# Supplementary material for: Efficacy and safety of Tuina (Chinese Therapeutic Massage) for chronic ankle instability: A systematic review and meta-analysis of randomized controlled trials
Source: PLoS One. 2025 Jun 6;20(6):e0321771. doi: 10.1371/journal.pone.0321771 (PMC12143534; doi:10.1371/journal.pone.0321771)
Supplement: S1 Table — (PDF) [file pone.0321771.s001.pdf]

Question: Should Tuina be used for Chronic Ankle Instability?

Bibliography: . Tuina for Chronic Ankle Instability. Cochrane Database of Systematic Reviews [Year], Issue [Issue].

Quality assessment							Summary of Findings				
**Participants (studies) Follow up**	**Risk of bias**	**Inconsistency**	**Indirectness**	**Imprecision**	**Publication bias**	**Overall quality of evidence**	**Study event rates (%)**		**Relative effect** (95% CI)	**Anticipated absolute effects**	
							**With Control**	**With Manipulation**		**Risk with Control**	**Risk difference with Manipulation** (95% CI)
**Clinical Effective Rate** (CRITICAL OUTCOME)											
607 (8 studies)	serious[1]	no serious inconsistency	no serious indirectness	no serious imprecision	undetected	⊕⊕⊕⊖ **MODERATE**[1] due to risk of bias	219/302 (72.5%)	288/305 (94.4%)	**OR 6.51** (3.76 to 11.28)	**Study population**	
										**725 per 1000**	**220 more per 1000** (from 183 more to 242 more)
										**Moderate**	
										**763 per 1000**	**191 more per 1000** (from 161 more to 210 more)
**Visual Analogue Scale** (IMPORTANT OUTCOME; Better indicated by lower values)											
611 (10 studies)	serious[1]	very serious[2]	no serious indirectness	no serious imprecision	undetected	⊕⊖⊖⊖ **VERY LOW**[1,2] due to risk of bias, inconsistency	297	314	-		The mean visual analogue scale in the intervention groups was **1.83 lower** (2.59 to 1.07 lower)
**Baird-Jackson Ankle Score** (CRITICAL OUTCOME; Better indicated by lower values)											
464 (7 studies)	serious[1]	serious[2]	no serious indirectness	no serious imprecision	undetected	⊕⊕⊖⊖ **LOW**[1,2] due to risk of bias, inconsistency	231	233	-		The mean baird-jackson ankle score in the intervention groups was **8.85 higher** (8.03 to 9.67 higher)
**AOFAS Ankle Hindfood Scale** (CRITICAL OUTCOME; Better indicated by lower values)											
215 (3 studies)	serious[1]	very serious[2]	no serious indirectness	serious[3]	undetected	⊕⊖⊖⊖ **VERY LOW**[1,2,3] due to risk of bias, inconsistency, imprecision	99	116	-		The mean aofas ankle hindfood scale in the intervention groups was **14.52 higher** (9.81 to 19.23 higher)

[1] Randomization and blinding are not adequate or appropriate
[2] High heterogeneity
[3] Insufficient sample size
