# Supplementary material for: Efficacy and safety of Tuina (Chinese Therapeutic Massage) for chronic ankle instability: A systematic review and meta-analysis of randomized controlled trials
Source: PLoS One. 2025 Jun 6;20(6):e0321771. doi: 10.1371/journal.pone.0321771 (PMC12143534; doi:10.1371/journal.pone.0321771)
Supplement: S2 File — (ZIP) [file pone.0321771.s004.zip › 1.正骨手法与中药熏洗治疗陈旧性踝关节扭伤的临床疗效比较_李西晋.pdf]

·中医·中西医结合·

# 正骨手法与中药熏洗治疗陈旧性踝关节扭伤的临床疗效比较

李西晋

【摘　要】　目的　正骨手法与中药熏洗治疗陈旧性踝关节扭伤的临床疗效比较。方法　选择天水市中西医结合医院 2011 年 5 月—2013 年 5 月收治的陈旧性踝关节扭伤患者 158 例，采用随机数字表法分为观察组和对照组，每组 79 例。对照组患者给予中药熏洗治疗，观察组患者给予正骨手法治疗，比较两组患者临床疗效及踝关节功能评分情况。结果　观察组患者的总有效率高于对照组，踝关节功能评分高于对照组，差异有统计学意义（$P < 0.05$）。结论　正骨手法治疗陈旧性踝关节扭伤的临床疗效优于中药熏蒸，可有效促进关节功能恢复。

【关键词】　损伤和劳损；踝关节；正骨手法；治疗结果
【中图分类号】　R 873　【文献标识码】　B　【文章编号】　1674-3296（2016）01C-0127-02
doi：10.15887/j.cnki.13-1389/r.2016.03.066

踝关节扭伤是四肢关节扭伤中常见的一种创伤，是患者在日常生活或锻炼过程中导致的一种损伤，当急性扭伤后未能及时有效治疗及引起足够重视，患者继续长期负重及运动，导致踝关节出现以酸痛、不能久行、久行无力及功能受限的一种疾病[1-3]。急性踝关节扭伤接受不恰当的正骨及其他物理、药物治疗亦能导致陈旧性踝关节扭伤的出现。踝关节出现陈旧性扭伤后关节活动受限、导致患者患侧小腿无力，严重影响患者日常生活及工作[4-6]。研究发现中医学在治疗陈旧性关节扭伤方面具有良好的效果，如外用膏药、中药熏洗、正骨手法等治疗措施。本研究旨在比较正骨手法与中药熏洗治疗陈旧性踝关节扭伤的临床疗效，现报道如下。

## 1　资料与方法

1.1　一般资料　选择天水市中西医结合医院 2011 年 5 月—2013 年 5 月收治的陈旧性踝关节扭伤患者 158 例，采用随机数字表法分为观察组和对照组，每组 79 例。对照组中男 42 例，女 37 例；年龄 22 ~ 56 岁，平均（34.5 ± 2.3）岁；病程 1 个月 ~ 2 年，平均（2.6 ± 1.2）个月；踝关节功能平均评分为（71.3 ± 4.4）分。观察组中男 43 例，女 36 例；年龄 21 ~ 52 岁，平均（34.3 ± 2.3）岁；病程 1 个月 ~ 2 年，平均（2.6 ± 1.3）个月；踝关节功能平均评分为（71.4 ± 4.5）分。两组患者一般资料比较，差异无统计学意义（$P > 0.05$），具有可比性。纳入标准：（1）患者入院后经诊断符合“中医病症诊断疗效标准”中陈旧性踝关节扭伤的相关诊断标准[7-9]，即患者既往有明显的踝关节扭伤史，扭伤时间至少在 1 个月及以上，主要临床表现为一定程度的踝部疼痛、无力，久行后症状更为明显，影响患者生活工作；（2）X 线检查示无脱位及骨折；（3）患者内踝或外髁前下方检查可见一定程度的肿胀及按压痛，部分患者可触及痛性筋结；（4）患者具有良好的依从性，自愿签署知情同意书，积极配合治疗及研究工作。排除标准：（1）患者既往存在风湿性关节炎、类风湿关节炎、痛风、强直性脊柱炎等关节炎症性疾病，关节肿瘤；（2）严重全身性器质性疾病如心力衰竭、肝肾功能障碍者，（3）对本研究使用中药存在过敏情况者。

1.2　方法　对照组患者接受中药熏洗治疗，熏洗使用药物包括乳香、苏木、没药、红花、当归、川断、土鳖虫、伸筋草、透骨草、（酒）大黄各 20g，苍术、天南星、海桐皮、路路通、五加皮、忍冬藤各 15g，以上药物加水浸泡 1h 后煎煮 0.5h，将药汁倒处后再加水煎煮 1 次，两次所得药汁混合使用。治疗前先将药汁煮沸，为患者进行熏蒸，当药汁冷却至合适温度后患者再将患足伸入进行浸泡，需注意药液平面应高于踝关节水平，1 剂/d，1 次/d，0.5h/次。观察组患者接受正骨手法治疗，当患者扭伤部位为外踝时嘱患者侧卧且上肢在上。助手用双手固定患者伤侧远端，操作者双手虎口相对，使用双手大拇指按住外踝间隙处，其余四肢握住患者伤足，并转动足环将之摇晃 6 ~ 7 次[10-12]并和助手相对拔伸，操作者继续将足内翻，内翻后外翻，同时使用双手拇指向下戳按，以上操作可重复，但不能超过 3 次。结束时于患侧外踝位置下方肿胀的软组织进行揉捻，缓解肿胀。当患者为内踝扭伤时保持患者侧卧且伤肢在下，助手同样固定伤侧小腿避免晃动，操作者双手拇指握住内踝间隙位置且其余四指握住伤足，沿足环转动 6 ~ 7 次并与助手对拔，完成后进行足外翻、内翻操作，同时双手拇指向下按压，以上操作不超过 3 次，结束时于内踝前下方对压痛、肿胀部位揉捻促进消散。

1.3　观察指标　观察两组患者临床疗效及踝关节功能评分情况。临床疗效依据“中医病症诊断疗效标准”[13-15]判定。痊愈：治疗后患者踝关节可进行各方向活动，局部静态及动态疼痛消失；显效：治疗后患者踝关节无痛活动范围达 80% 及以上，局部静态疼痛消失；有效：治疗后患者踝关节活动情

作者单位：741020 甘肃省天水市中西医结合医院

(C)1994-2024 China Academic Journal Electronic Publishing House. All rights reserved.　http://www.cnki.net

况未达 80%，疼痛情况有所缓解；无效：治疗后患者病情未见明显好转，甚至发生恶化。总有效率 = 痊愈率 + 显效率 + 有效率。踝关节功能评分使用 Baird - Jackon 踝关节评分进行评价。

1.4 统计学方法 采用 SPSS 19.0 统计软件进行数据处理，计数资料采用 $\chi^2$ 检验；计量资料以 $\bar{x} \pm s$ 表示，采用 $t$ 检验。以 $P<0.05$ 为差异有统计学意义。

## 2 结 果

2.1 两组患者临床疗效比较 观察组总有效率高于对照组，差异有统计学意义（$\chi^2=12.45$，$P<0.05$，见表 1）。

表 1 两组患者临床疗效比较

| 组别 | 例数 | 痊愈 | 显效 | 有效 | 无效 | 总有效率 |
|---|---|---|---|---|---|---|
| 观察组 | 79 | 38(48.10) | 34(43.04) | 6(7.59) | 1(1.27) | 78(98.73)* |
| 对照组 | 79 | 26(32.91) | 15(18.99) | 24(30.38) | 14(17.72) | 65(82.28) |

注：与对照组比较，* $P<0.05$

2.2 两组患者踝关节功能比较 观察组患者踝关节功能评分为（93.2 ±4.9）分，对照组为（84.8 ±6.5）分；观察组患者踝关节功能评分高于对照组，差异有统计学意义（$t=9.18$，$P<0.05$）。

## 3 讨 论

踝关节为下肢的承重关节，主要由胫骨下端及距骨体上侧构成，该关节于胫腓骨下端有内外踝及周围韧带保持关节稳定[16-17]。临床常见的损伤部位为外侧副韧带，其次为内侧副韧带、踝关节关节囊、胫腓横韧带等损伤[18-19]。如果采取及时的制动、外固定、药物及理疗等手法可痊愈，但若延误治疗或早期治疗不当可导致局部反复水肿，甚至出现局部组织粘连等问题，造成陈旧性踝关节扭伤[20]。陈旧性踝关节扭伤在中医学属于“伤筋”范畴，一般由于运动过度或用力不当等原因导致局部瘀血未化、气血运行不畅，最终导致关节功能丧失。中医熏洗是通过煮沸的药物作用及温热作用刺激局部血管、神经及皮肤，最终达到疏通筋络、促进血液循坏的作用。正骨手法是通过正骨、理顺筋节及分筋等作用于局部使病变部位痉挛的肌肉韧带达到松弛效果，纠正踝关节扭转、错缝等情况，是一种治疗陈旧性踝关节的重要手段。

本研究结果显示，观察组患者总有效率明显高于对照组，说明在临床陈旧性踝关节治疗中，正骨手法与中药熏蒸均为治疗的有效方法，且正骨手法的治疗效果优于中医熏蒸效果；观察组患者踝关节功能评分高于对照组，说明通过正骨手法治疗后踝关节功能恢复情况优于对照组，正骨手法治疗后除缓解患者疼痛及气血运行不畅等症状外促进关节功能恢复，效果较中药熏蒸更为明显。

综上所述，正骨手法治疗陈旧性踝关节扭伤的临床疗效优于中药熏蒸，可有效促进关节功能恢复，值得临床推广使用。

## 参考文献

[1] 李俊海，王庆甫，黄沪. 正骨手法与中药熏洗治疗陈旧性踝关节扭伤的病例对照研究 [J]. 中国骨伤，2012，25（2）：113-115.
[2] 齐越峰，赵兴玮，陈福林，等. 正骨散治疗急性软组织损伤的临床观察 [J]. 北京中医药，2013，27（8）：587-590.
[3] 刘文志. 踝关节扭伤的治疗体会 [J]. 实用骨科杂志，2011，11（6）：890.
[4] 王亦璁. 骨与关节损伤 [M]. 4 版. 北京：人民卫生出版社，2007：1512-1516.
[5] 杨春花. 针刺配合小针刀治疗陈旧性踝关节扭伤 30 例 [J]. 浙江中医杂志，2012，47（3）：198.
[6] 木荣华. 针刀配合手法治疗陈旧性外踝关节扭伤 120 例 [J]. 中国骨伤，2002，15（11）：685.
[7] 管玉杰. 医用臭氧配合针刀治疗 m 级及以下膝关节骨性关节炎 327 例疗效分析 [J]. 健康大视野，2013，21（6）：174.
[8] 卢文，刘淳. 火针、刺血治疗退行性膝关节炎临床疗效观察 [J]. 中国中医骨伤科杂志，2009，17（10）：52.
[9] 彭程，王进. 推拿配合铺灸治疗增生性膝关节炎 50 例 [J]. 云南中医中药杂志，2010，31（3）：39-40.
[10] 朱启明. 手法治疗腰源性膝关节痛 56 例 [J]. 中国疗养医学，2010，19（5）：436.
[11] 焦秋里，徐东. 脊源性膝关节疼痛临床分析 [J]. 中国社区医师：医学专业，2011，15（13）：278.
[12] 张农. 龟鹿二仙胶治疗老年增生性骨关节炎的临床观察 [J]. 中医临床研究，2012，4（13）：79-80.
[13] 郭敏. 三瘦汤配合针刺疗法治疗膝关节骨性关节炎 30 例临床观察 [J]. 中医临床研究，2012，4（16）：21-22.
[14] 史中亚，胡奋强，陈勇. 动态针刺配合功能训练治疗膝痛症疗效观察 [J]. 中医药临床杂志，2010，22（1）：54-55.
[15] 张素玲，王舰. 董氏奇穴刺络拔罐法治疗膝骨性关节炎 30 例 [J]. 中国针灸，2010，30（5）：358.
[16] 谢传钞，吴镇阳. 董氏奇穴配合关节松动术治疗膝关节骨性关节炎临床研究 [J]. 按摩与康复医学，2012，3（8）：28-29.
[17] 陈刚，曹怀焱. 关节镜治疗膝关节骨性关节炎 56 例 [J]. 中外医疗，2011，30（19）：103.
[18] 张素玲，王舰. 董氏奇穴刺络拔罐法治疗膝骨性关节炎 30 例 [J]. 中国针灸，2010，30（5）：358.
[19] 张美花. 推拿辅以超声波疗法治疗陈旧性踩关节扭伤的疗效观察及护理 [J]. 内蒙古中医药，2014（2）：44-45.
[20] 陈俊辉，陈铜，孙琪，等. 非留体抗炎药的胃肠副作用 4417 例临床分析 [J]. 中华风湿病杂志，2001，5（3）：194-196.

（收稿日期：2015-10-11）

(C)1994-2024 China Academic Journal Electronic Publishing House. All rights reserved. http://www.cnki.net
