# Supplementary material for: Efficacy and safety of Tuina (Chinese Therapeutic Massage) for chronic ankle instability: A systematic review and meta-analysis of randomized controlled trials
Source: PLoS One. 2025 Jun 6;20(6):e0321771. doi: 10.1371/journal.pone.0321771 (PMC12143534; doi:10.1371/journal.pone.0321771)
Supplement: S2 File — (ZIP) [file pone.0321771.s004.zip › 2.正骨手法与中药熏洗治疗陈旧性踝关节扭伤的病例对照研究_李俊海.pdf]

·临床研究·

正骨手法与中药熏洗治疗陈旧性踝关节扭伤的病例对照研究

李俊海，王庆甫，黄沪
（北京中医药大学第三附属医院骨科，北京 100029）

【摘要】 **目的**：比较正骨手法与中药熏洗治疗陈旧性踝关节扭伤的临床效果。**方法**：2008 年 3 月至 2011 年 5 月，将 76 例患者分为治疗组 39 例和对照组 37 例。治疗组：男 15 例，女 24 例；年龄 20~59 岁，平均（42.97±9.21）岁；病程 1~60 个月；踝关节功能评分平均（71.27±4.50）分。对照组：男 11 例，女 26 例；年龄 25~57 岁，平均（41.29±8.77）岁；病程 1~36 个月，平均（8.47±7.37）个月；踝关节功能评分平均（71.45±4.61）分。治疗组采用正骨手法治疗，每周 2 次；对照组采用中药熏洗治疗，每日 1 次。采用 Baird-Jackson 踝关节功能评分，3 周后比较 2 组患者的踝关节功能评分及其治疗效果。**结果**：治疗 3 周后，治疗组踝关节评分为（93.44±4.91）分，对照组踝关节评分为（85.81±6.57）分，差异有统计学意义（$P<0.05$），治疗组评分优于对照组。治疗组治疗前后的踝关节平均分差异有统计学意义（$P<0.05$），对照组治疗前后的踝关节平均分差异有统计学意义（$P<0.05$）。治疗组优 16 例，良 18 例，可 3 例，差 2 例；对照组优 9 例，良 14 例，可 5 例，差 9 例，治疗组疗效优于对照组。**结论**：正骨手法与中药熏洗治疗陈旧性踝关节扭伤均有一定的疗效，且前者优于后者。

【关键词】 踝关节； 扭伤和劳损； 正骨手法； 中草药； 病例对照研究
DOI：10.3969/j.issn.1003-0034.2012.02.008

Case-control study on therapeutic effects between bone-setting and herbal fumigation for the treatment of the obsolete malleolus joint sprains

LI Jun-hai, WANG Qing-fu, HUANG Hu. Department of Orthopaedics, the Third Hospital Affiliated to Beijing University of Traditional Chinese Medicine, Beijing 100029, *China*

ABSTRACT Objective: To compare the clinical effects of bone-setting technique and herbal fumigation for the treatment of the obsolete malleolus joint sprains. **Methods**: From March 2008 to May 2011, 76 patients were divided into treatment group (39 cases) and control group (37 cases). In the treatment group: 15 males and 24 females; the age ranged from 20 to 59 years with an average of (42.97±9.21) years; the course of disease ranged from 1 to 60 months; the average score of ankle joint function was (71.27±4.50). In the control group: 11 males and 26 females; the age ranged from 25 to 57 years with an average of (41.29±8.77) years; the course of disease ranged from 1 to 36 months with an average of (8.47±7.37) months; the average score of ankle joint function was (71.45±4.61). The patients in the treatment group were treated with bone-setting technique two times a week, and the patients in the control group were treated with herbal fumigation once a day. The ankle joint function scores and treatment effects of the two groups were compared after 3 weeks by using Baird-Jackson ankle function score. **Results**: After 3 weeks of the treatment, the average score of ankle joint function of the treatment group was (93.44±4.91), and in the control group was (85.81±6.57), the difference has statistical significance. The treatment group score was better than that of the control group. Before and after treatment, the average ankle score of the treatment group was (71.27±4.50) and (93.44±4.91), the difference has statistical significance. Before and after treatment, the average ankle score of the control group was (71.45±4.61) and (85.81±6.57), the difference has statistical significance. In the treatment group, 16 cases got an excellent result, 18 good, 3 fair, 2 poor; in the control group, 9 cases got an excellent result, 14 good, 5 fair, 9 poor. The difference has statistical significance. **Conclusion**: The bone-setting techniques and herbal fumigation treatment of obsolete malleolus joint sprains both have a certain effect, and the former is better than the latter.

KEYWORDS Ankle joint; Sprains and strains; Bone setting manipulation; Drugs, Chinese herbal; Case-control studies

Zhongguo Gu Shang/China J Orthop Trauma, 2012, 25(2): 113-115 www.zggszz.com

陈旧性踝关节扭伤是急性扭伤后因重视不够，没有及时治疗，伤后仍长期负重活动，而导致踝关节酸痛无力、不能久行、功能受限的一种常见病。因症状缠绵不愈，严重影响日常生活和工作，尽管中药熏

通讯作者：李俊海 E-mail：ljy621229@sina.com

万方数据

洗有一定疗效，但有些患者嫌麻烦，不愿使用。笔者在临床实践中发现，正骨手法治疗该病疗效显著。为进一步探讨其治疗效果，自 2008 年 3 月至 2011 年 5 月，采用中药熏洗与正骨手法治疗相对照，比较其疗效。

1　资料与方法

1.1　临床资料与分组方法　符合纳入标准的 76 例分为治疗组 39 例和对照组 37 例，均来自本院门诊。其中治疗组 39 例，年龄 20~59 岁，病程 1~60 个月；对照组 37 例，年龄 25~57 岁，病程 1~36 个月。2 组患者临床资料比较见表 1，2 组具有可比性。

1.2　诊断、纳入及排除标准　诊断标准：参照中华人民共和国中医药行业标准《中医病证诊断疗效标准》制定[1]：①有明确的踝部扭伤史；②扭伤时间在 1 个月以上；③踝关节疼痛、无力，不能久行，影响生活、工作和运动；④内踝或外髁前下方处可有不同程度的肿胀和压痛，或可触及痛性"筋节"；⑤X 线片未见骨折和脱位。纳入标准：①符合诊断标准；②年龄 20~60 岁；③同意参加本研究，并签署知情同意书。排除标准：①过敏体质，既往对中药外敷或外洗过敏；②患有关节炎症表现的疾病，如类风湿关节炎、强直性脊柱炎、痛风（发作期）等。

1.3　治疗方法　治疗组采用正骨手法治疗，每周 2 次；对照组采用中药熏洗治疗，每日 1 次。

1.3.1　手法治疗[2]　外踝扭伤的治疗：患者侧卧，伤肢在上。助手双手握住伤侧小腿远端固定，勿使摇动。医者两虎口相对，双手拇指按住外踝间隙处，余 4 指拿住伤足，将足环转摇晃 6~7 次后，与助手相对拔伸，并将足内翻，接着再外翻，并双手拇指向下戳按。整个操作可再重复 1 次，最多 3 次。最后在外踝前下方处，对肿胀、压痛的软组织或筋结进行揉捻治疗，使其变软、变小甚至消失为止。

内踝扭伤的治疗：患者侧卧，伤肢在下。助手双手握住伤侧小腿远端固定，勿使摇动。医者两虎口相对，双手拇指按住内踝间隙处，余 4 指拿住伤足，将足环转摇晃 6~7 次后，与助手相对拔伸，并将足外翻，接着再内翻，并双手拇指向下戳按。整个操作可再重复 1 次，最多 3 次。最后在内踝前下方处，对肿胀、压痛的软组织或筋结进行揉捻治疗，使其变软、变小甚至消失为止。

1.3.2　中药熏洗　熏洗药物组成：乳香 20 g，没药 20 g，苏木 20 g，红花 20 g，当归 20 g，土鳖虫 20 g，川断 20 g，酒大黄 20 g，苍术 15 g，天南星 15 g，路路通 15 g，海桐皮 15 g，忍冬藤 15 g，五加皮 15 g，伸筋草 20 g，透骨草 20 g。方法：将上述药物加水适量浸泡 60 min，加水煎煮 30 min 后，取汁。原药再加水煎煮 1 次，最后将全部滤液合在一起。每次治疗时将药液煎沸，先熏蒸，待药液温度合适时再浸泡患足。药液量要超过踝关节，每日 1 次，每次 30 min，每日 1 剂。

1.4　疗效评定标准　按照 Baird-Jackson[3]踝关节评分，3 周后比较 2 组患者的踝关节功能评分及其治疗效果。Baird-Jackson 踝关节评分系统最高评分 100 分，优 96~100 分，良 91~95 分，可 81~90 分，差 0~80 分。

1.5　统计学处理　应用 SPSS 13.0 统计软件包进行统计学分析，定量资料以均数±标准差（$\bar{x}\pm s$）表示，采用成组设计定量资料的 t 检验，定性资料采用 χ^2 检验，等级资料采用秩和检验，以 $P<0.05$ 为差异有统计学意义。

2　结果

治疗 3 周后评价 2 组的疗效。2 组踝关节评分比较见表 2，除放射学评分（2 组患者治疗前、后均无异常）外，分项和总平均分差异均有统计学意义，治疗组评分优于对照组，提示正骨手法在改善踝关节功能方面优于中药熏洗。治疗后 2 组踝关节平均分均较治疗前提高，差异有统计学意义（见表 2），提示正骨手法治疗和中药熏洗 2 种治疗方法都有效。治疗后治疗组优 16 例，良 18 例，可 3 例，差 2 例；对照组优 9 例，良 14 例，可 5 例，差 9 例。2 组疗效比较，经秩和检验，u=2.429，P=0.007 6，2 组疗效差异有统计学意义，治疗组优于对照组。

3　讨论

3.1　正骨手法的渊源和特点　本组所用的正骨手法最早源于上驷院绰班处，上驷院为清朝内务府所属三院之一。主要任务是为清朝宫廷驯养马匹，为数众多的领侍卫衔的蒙古医生亦属上驷院管辖，其主

表 1　2 组临床资料比较

Tab.1　Comparison of clinical data between two groups before treatment

组别	例数（例）	年龄（$\bar{x}\pm s$，岁）	性别（例） 男	性别（例） 女	病程（$\bar{x}\pm s$，月）	踝关节评分（$\bar{x}\pm s$）
治疗组	39	42.97±9.21	15	24	7.65±9.43	71.27±4.50
对照组	37	41.29±8.77	11	26	8.47±7.37	71.45±4.61
检验值	–	t= 0.814	χ^2=0.643		t =0.435	t=0.172
P 值	–	0.419	0.423		0.664	0.864

万方数据

表 2 治疗后 2 组踝关节评分比较($\bar{x}\pm s$,分)

Tab.2 Comparison of malleolus joint grading between two groups after treatment($\bar{x}\pm s$, score)

组别	例数(例)	治疗前	疼痛	踝关节稳定性	行走能力	跑步能力	工作能力	踝关节活动范围	放射学结果	踝关节总平均分
治疗组	39	71.27±4.50	14.26±2.78	10.98±4.98	13.89±2.61	9.47±1.72	9.89±1.39	9.95±2.26	25.00±0.00	93.44±4.91*
对照组	37	71.45±4.61	12.99±2.36	8.64±4.91	12.56±2.82	8.64±1.69	9.19±1.46	8.79±2.41	25.00±0.00	85.81±6.57**
t 值	-	0.172 0	2.141 4	2.061 5	2.135 0	2.120 6	2.141 2	2.165 5	-	5.751 0
P 值	-	0.864 0	0.035 5	0.042 8	0.036 1	0.037 3	0.035 5	0.033 6	-	0.000 1

注:与治疗前比较,* t=20.788,P<0.05;**t=10. 883,P<0.05

Note: Compared to before treatment, *t=20.788, P<0.05; **t=10.883, P<0.05

要职责乃是为大内人员正骨医伤。"绰班"一词是满词,译成汉语就是"正骨医生","绰班处"即"正骨处"。据考,上驷院绰班处正式成立时间当在道光初年,后成为清廷大内惟一的骨科医疗机构,并进入全盛时期[4]。其手法治病的最大特点是"轻巧柔和,筋骨并重,治病使患者不知其苦"。

3.2 正骨手法治疗机制 根据本手法的操作方法推断治病机制为松解踝关节周围软组织的粘连和纠正踝关节的微细错位(骨错缝)。急性踝关节扭伤如果没有及时治疗或适当休息, 负重活动会使损伤的韧带不能修复,无菌性炎症反应长期存在,就会发生增生粘连,影响踝关节活动且遗留经常疼痛,而形成陈旧性伤[5]。此手法中对抗牵引和环转摇晃踝关节的目的就是松解踝关节的粘连,改善关节功能,达到舒筋通络、消肿止痛、气血运行通畅的作用。对局部软组织的肿胀和筋结进行揉捻的操作手法也是此目的。

踝关节扭伤中有相当一部分伴有骨错缝, 而这很少被 X 线检查所显示,由于骨错缝、筋出槽,如不整复,即骨不平、筋不顺,关节失去内平衡,临床上遇到久治不愈者即属此类, 这是因为在治疗时只注意到软组织的损伤而忽视了骨错缝内在的一面。外踝扭伤可引起踝关节外侧间隙的微细错位, 根据中医"欲合先离"的理论,复位时先顺扭伤机制使踝关节内翻,然后再逆扭伤机制使踝关节外翻,并双手拇指向下戳按将其复位;内踝扭伤同理。此种操作手法的运用就是为了纠正踝关节的微细错位, 使粘连得以松解,错位得以复正[6]。

参考文献

[1] 国家中医药管理局. 中医病证诊断疗效标准[M]. 南京:南京大学出版社,1994:201-202.
National Chinese Medicine Administrative Bureau. The standard of diagnosis and curative effect about Chinese medical disease and symptom[M]. Nanjing: Nanjing University Publishing House, 1994:201-202. Chinese.

[2] 北京中医药大学东直门医院. 刘寿山正骨经验[M]. 北京:人民卫生出版社,2006:361-363.
Dongzhimen Hospital of Beijing Chinese Medicine University. *Liu Shou-shan* bone-setting experience[M]. Beijing: People's Medical Publishing House, 2006:361-363. Chinese.

[3] Baird RA, Jackson ST. Fractures of the distal part of the fibula with associated disruption of the deltoid ligament. Treatment without repair of the deltoid ligament[J]. J Bone Joint Surg Am, 1987, 69(9):1346-1352.

[4] 张军,唐东昕,李俊海,等. 孙氏筋伤手法脉系源流追溯考究[J]. 中国中医骨伤科杂志,2007,15(3):51-52.
Zhang J, Tang DX, Li JH, et al. The source and course backward about the managing muscle technique of the *Sun*[J]. Zhongguo Zhong Yi Gu Shang Ke Za Zhi, 2007, 15(3):51-52. Chinese.

[5] 刘文志. 踝关节扭伤的治疗体会[J]. 实用骨科杂志,2005,11(6):550.
Liu WZ. The treatment experience about malleolus joint sprains[J]. Shi Yong Gu Ke Za Zhi, 2005, 11(6):550. Chinese.

[6] 方楚权. 踝部扭伤的中后期治疗[J]. 中国骨伤,2000,13(6):378.
Fang CQ. The treatment of ankle sprain in the late[J]. Zhongguo Gu Shang/China J Orthop Trauma, 2000, 13(6):378. Chinese.

(收稿日期:2011-09-05 本文编辑:连智华)

万方数据
