# Supplementary material for: Efficacy and safety of Tuina (Chinese Therapeutic Massage) for chronic ankle instability: A systematic review and meta-analysis of randomized controlled trials
Source: PLoS One. 2025 Jun 6;20(6):e0321771. doi: 10.1371/journal.pone.0321771 (PMC12143534; doi:10.1371/journal.pone.0321771)
Supplement: S2 File — (ZIP) [file pone.0321771.s004.zip › 3.正骨理筋手法联合体外冲击波...性踝关节扭伤的临床疗效观察_张博.pdf]

分类号：　　　　　　　　　　　　　　　　　　　　密级：

甘肃中醫藥大學
GANSU UNIVERSITY OF CHINESE MEDICINE

硕 士 研 究 生 学 位 论 文

论文题目（中文）正骨理筋手法联合体外冲击波治疗陈旧性踝关节扭伤的临床疗效观察

论文题目（外文）Observation on the clinical effect of bone-setting manipulation combined with extracorporeal shock wave in the treatment of chronic ankle sprain

研 究 生 姓 名　张博

学　科、专　业　中医骨伤科学

研　究　方　向　中医药治疗骨伤科疾病

导师姓名、职称　姜劲挺 教授 主任医师

论　文　工　作
起　止　年　月　2019 年 12 月 至 2021 年 2 月

论文提交日期　2021 年 3 月

论文答辩日期　2021 年 5 月

学位授予日期　2021 年 6 月

中国知网 https://www.cnki.net

中国知网 https://www.cnki.net

目 录

摘要 1
ABSTRACT 3
英文缩写词表 6
前言 7
第一部分 临床研究 9
一、临床对象 9
1 病例来源 9
2 诊断标准 9
2.1 中医诊断标准 9
2.2 西医诊断标准 9
3 纳入标准 9
4 排除标准 10
5 脱落剔除标准 10
6 脱落病人处理 10
二、研究方法 10
1 分组方法 10
2 治疗设备 10
3 治疗方案 10
3.1 联合组 10
3.2 手法组 11
3.3 冲击波组 11
4 观察指标 11
4.1 一般资料 11
4.2 VAS 疼痛评分 11
4.3 Baird-Jackson(1987)踝关节评分 12
4.4 踝关节活动度（ROM）评分 12

中国知网 https://www.cnki.net

4.5 肿胀程度......12
5 疗效评价标准......12
6 观察时间点......12
7 不良事件处理......13
8 统计分析......13
第二部分 结果......14
1 纳入病例完成情况......14
2 一般资料分析......14
3 三组治疗前各指标组间对比......15
4 三组患者治疗前后各指标变化......16
4.1 VAS 评分比较......16
4.2 Baird-Jackson 踝关节评分比较......17
4.3 ROM 踝关节活动度评分比较......18
4.4 肿胀程度比较......19
5 三组总体疗效对比......21
6 不良事件......21
第三部分 讨论......22
1 现代医学对陈旧性踝关节扭伤的认识......22
1.1 解剖、稳定性及扭伤机制......22
1.2 症状、诊断及常见治疗......23
2 祖国医学对陈旧性踝关节扭伤的认识......24
2.1 筋骨病机与伤筋和错缝......24
2.2 痹症......24
3 正骨理筋手法......26
3.1 正骨理筋手法作用......26
3.2 正骨理筋手法的要求及操作......26
3.3 正骨理筋手法治疗陈旧性踝关节扭伤的临床研究......26
4 体外冲击波......27
中国知网 https://www.cnki.net

4.1 体外冲击波的作用......27
4.2 体外冲击波治疗肌腱、韧带方面的研究进展......28
4.3 体外冲击波治疗陈旧性踝关节扭伤的临床研究......28
5 正骨理筋手法联合体外冲击波治疗......29
6 临床研究数据及疗效分析......29
6.1 VAS 评分比较......30
6.2 Baird-Jackson 踝关节评分比较......30
6.3 ROM 踝关节活动度评分比较......30
6.4 肿胀程度比较......31
6.5 总体疗效对比......31
第四部分 结语......33
1 结论......33
2 问题及展望......33
2.1 问题......33
2.2 展望......33
参考文献......34
文献综述......39
中西医治疗陈旧性踝关节扭伤研究概况......39
1 中医药治疗......39
1.1 中药治疗......39
1.2 针灸治疗......40
1.3 手法治疗......41
2 西医治疗......42
2.1 药物治疗......42
2.2 非手术物理治疗......42
2.2.1 功能锻炼......43
2.2.2 器具辅助疗法......43
2.2.3 体外冲击波......43

中国知网 https://www.cnki.net

2.3 手术治疗......44
2.3.1 对韧带起止点的原位解剖缝合手术......45
2.3.2 韧带重建手术......45
参考文献......46
附录......50
附录 1 知情同意书......50
附录 2 体外冲击波及治疗图片......51
附录 3 病例资料采集册......52
附录 4 VAS 疼痛评分量表......53
附录 5 Baird-Jackson(1987)踝关节评分量表......54
附录 6 Kofoed 评分量表......56
附录 7 肿胀程度......57
附录 8 医学伦理审查表......58
致谢......59
研究生学习期间主要研究成果......60

中国知网 https://www.cnki.net

摘 要

目的：观察正骨理筋手法联合体外冲击波治疗陈旧性踝关节扭伤的临床疗效，对比手法联合体外冲击波与单独用手法或体外冲击波治疗陈旧性踝关节扭伤的优势，为治疗此种疾病提供新的思路和可行性方法。

方法：2020 年 01 月到 2020 年 12 月期间，在甘肃中医药大学附属医院宋氏正骨科门诊就诊的陈旧性踝关节扭伤患者，将合乎本次研究标准的 90 例患者随机分为 3 组：①联合组 30 例，采用正骨理筋手法联合体外冲击波治疗；②手法组 30 例，采用正骨理筋手法治疗；③冲击波组 30 例，采用体外冲击波治疗。所有患者医治时间为 2 周，在治疗前、治疗 2 周后及治疗后 1 个月（随访）三个时间点上记录患者的视觉模拟疼痛评分（Visual analogue scale，VAS）、Baird-Jackson 踝关节评分、ROM 踝关节活动度评分及踝关节肿胀程度的情况来评估和量化疾病的缓解程度，最后评定三组的总体疗效。所有数据应用 SPSS 26 软件包进行统计学处理。

结果：

1. VAS 评分比较

组内比较：三组治疗 2 周后和治疗后 1 个月 VAS 评分比较均有统计学差异，且评分降低。组间比较：三组治疗前 VAS 评分比较无统计学差异。治疗 2 周后，三组比较有统计学差异，且评分降低，三组组间多重对比，P 值均小于 0.05，有统计学差异；治疗后 1 个月，三组比较有统计学差异，且评分降低，LSD 法两两比较，手法组和冲击波组 $P=0.503>0.05$。

2. Baird-Jackson 踝关节评分比较

组内比较：三组治疗 2 周后和治疗后 1 个月 Baird-Jackson 踝关节评分均有统计学差异，且评分升高。组间比较：三组治疗前 Baird-Jackson 踝关节评分无统计学差异。治疗 2 周后和治疗后 1 个月三组比较有统计学差异，且评分升高，LSD 法两两比较，P 值均小于 0.05，有统计学差异。

3. ROM 踝关节活动度评分比较

组内比较：联合组和手法组在治疗 2 周后和治疗后 1 个月 ROM 踝关节活动度评分有统计学差异，且评分升高，冲击波组在治疗 2 周后与治疗前比较 $P=0.000<0.05$，治疗后 1 个月与治疗 2 周后比较 $P=0.129>0.05$。组间比较：三组治疗前 ROM 踝关节活动度

中国知网 https://www.cnki.net

评分无统计学差异。治疗 2 周后，三组比较有统计学差异，且评分升高，LSD 法两两比较，联合组和手法组比较：P=0.111>0.05；治疗后 1 个月，三组比较有统计学差异，且评分升高，三组组间多重对比，P 值均小于 0.05。

4. 肿胀程度比较

组内比较：联合组和手法组在治疗 2 周后和治疗后 1 个月肿胀程度比较有统计学差异，且程度减轻，但在治疗后 1 个月与治疗 2 周后比较 P 值均大于 0.05；冲击波组组内比较 P=0.267>0.05，差异无统计学意义。组间比较：三组治疗前肿胀程度无统计学差异。治疗 2 周后和治疗后 1 个月，三组比较有统计学差异，且程度减轻，三组组间多重对比，联合组和手法组比较，P 值均大于 0.05。

5. 总体疗效比较

总有效率联合组、手法组、冲击波组分别为 93.10％、86.67％、75.86％，三组比较有统计学差异，组间多重对比，P 值均小于 0.05。

结论：

1. 正骨理筋手法联合体外冲击波、正骨理筋手法和体外冲击波对陈旧性踝关节扭伤均有疗效。

2. 正骨理筋手法联合体外冲击波治疗陈旧性踝关节扭伤好于单纯正骨理筋手法和体外冲击波疗法，且正骨理筋手法治疗陈旧性踝关节扭伤好于体外冲击波疗法。

关键词：正骨理筋手法；体外冲击波；陈旧性踝关节扭伤；临床疗效

中国知网 https://www.cnki.net

ABSTRACT

Objectives: Observe the clinical efficacy of bone and tendon manipulation combined with extracorporeal shock wave in the treatment of old fractures and sprains, and compare the advantages of manipulation combined with extracorporeal shock wave and separate treatment or extracorporeal shock wave in the treatment of old torsion sprains, and provide new ideas and feasibility for treating diseases method.

Methods: From January 2020 to December 2020, 90 patients with old ankle sprains who were treated at the Song's orthopedics clinic of the Affiliated Hospital of Gansu University of Traditional Chinese Medicine were randomly divided into 3 groups: ①Combined group Thirty cases were treated with osteotomy and tendon manipulation combined with extracorporeal shock wave treatment; ②30 cases in the manipulation group were treated with osteotomy and tendon manipulation; ③30 cases in the shock wave group were treated with extracorporeal shock wave. The treatment time for all patients was 2 weeks, and the visual analogue scale (VAS) and Baird-Jackson ankle joints were recorded at three time points before treatment, 2 weeks after treatment, and 1 month after treatment (follow-up). ROM ankle range of motion score and ankle swelling to evaluate and quantify the degree of disease relief, and finally evaluate the overall efficacy of the three groups. All data were statistically processed using SPSS 26 software package.

Results:

1. VAS score comparison

Intra-group comparison: The VAS scores of the three groups were statistically different after 2 weeks of treatment and 1 month after treatment, and the scores were reduced. Comparison between groups: There was no statistical difference in the VAS scores of the three groups before treatment. After 2 weeks of treatment, there was a statistical difference between the three groups, and the score was reduced. Multiple comparisons between the three groups, the *P* value was less than 0.05, there was a statistical difference; 1 month after the treatment, there was a statistical difference between the three groups, and the scores were reduced, and the

中国知网 https://www.cnki.net

LSD method was compared in pairs. P=0.503>0.05 in the manipulation group and the shock wave group.

2. Baird-Jackson ankle score comparison

Intra-group comparison: The Baird-Jackson ankle scores of the three groups were statistically different after 2 weeks of treatment and 1 month after treatment, and the scores increased. Comparison between groups: There was no statistical difference in Baird-Jackson ankle scores before treatment in the three groups. There was a statistical difference between the three groups after 2 weeks of treatment and 1 month after treatment, and the scores increased. The LSD method was compared in pairs, and the P values were all less than 0.05, which was statistically different.

3. Comparison of ROM ankle range of motion score

Intra-group comparison: The combination group and the manipulation group had statistical differences in the ROM ankle range of motion scores after 2 weeks of treatment and 1 month after treatment, and the scores increased. The shock wave group was compared with before treatment after 2 weeks of treatment, P=0.000<0.05, P=0.129>0.05 compared with 1 month after treatment and 2 weeks after treatment. Comparison between groups: There was no statistical difference in the ROM ankle range of motion scores between the three groups before treatment. After 2 weeks of treatment, there was a statistical difference between the three groups, and the scores increased. LSD method was used to compare pairwise, the combination group and the manual group were compared: P=0.111>0.05; 1 month after treatment, there was a statistical difference between the three groups, And the score increased, multiple comparisons between the three groups, P values were all less than 0.05.

4. Comparison of swelling

Intra-group comparison: There is a statistically significant difference between the combined group and the manipulation group in the degree of swelling after 2 weeks of treatment and 1 month after treatment, and the degree is reduced, but the P value is greater than 0.05 between 1 month after treatment and 2 weeks after treatment; Compared with the shock wave group, P=0.267>0.05, the difference was not statistically significant. Comparison between

中国知网 https://www.cnki.net

groups: There was no statistical difference in the degree of swelling before treatment in the three groups. After 2 weeks of treatment and 1 month after treatment, there was a statistical difference between the three groups, and the degree was reduced. Multiple comparisons between the three groups, the combination group and the manipulation group, *P* values were all greater than 0.05.

5. Comparison of overall efficacy

The total effective rate was 93.10%, 86.67%, and 75.86% in the combination group, manipulation group, and shock wave group, respectively. There were statistical differences among the three groups. For multiple comparisons between the groups, the *P* values were all less than 0.05.

Conclusions:

1. The combination of bone and tendon manipulation combined with extracorporeal shock wave, bone and tendon manipulation and extracorporeal shock wave have curative effects on old ankle sprains.

2. The combination of bone and tendon manipulation combined with extracorporeal shock wave is better than the simple bone and tendon manipulation and extracorporeal shock wave therapy, and the bone and tendon manipulation is better than extracorporeal shock wave therapy for old ankle sprains.

Key Words: bone-setting manipulation; extracorporeal shock wave; chronic ankle sprain; clinical efficacy

中国知网 https://www.cnki.net

英文缩写词表

缩写词	英文全称	中文全称
AOFAS	American Orthopedic Foot and Ankle Society	美国足与踝关节协会
CAS	Chronic ankle sprain	陈旧性踝关节扭伤
ESWT	Extracorporeal shock wave therapy	体外冲击波疗法
MRI	Magnetic Resonance Imaging	磁共振成像
ROM	Range of motion	关节活动度
VAS	Visual analogue scale	视觉模拟评分法
VEGF	Vascular endothelial growth factor	血管内皮生长因子

中国知网 https://www.cnki.net

前 言

踝关节扭伤占全身关节扭伤的 80%以上[1]，是日常生活与运动中的常见疾病。本病可发生于任何年龄，女性多见，多因行走于不平路面、下楼梯或者跑跳、穿高跟鞋、体育训练时动作不协调、行走不稳导致踝跖屈位时，骤然足强力内翻或外翻，造成外侧副韧带或者内侧副韧带牵拉伤，急性期以踝关节明显肿胀疼痛、跛行、功能障碍、皮肤青紫为特征，大部分患者经初始保守治疗，如早期活动指导、理疗、绷带、支具等，可完全恢复；如未能得到及时治疗或治疗方法不得当，使损伤的韧带不能及时恢复，会造成组织的粘连、增生和慢性炎症的存在，患者踝关节出现慢性疼痛、酸困无力、肿胀、不稳定感、担心扭脚、活动不利等症状，发展为陈旧性踝关节扭伤。

本病归属中医“踝缝伤筋[2]”、“筋伤”或者“痹症”范畴，因外伤导致筋脉受损，淤血内滞，久病入络，经络气血不通，风寒湿邪乘隙而入，进一步加重筋脉失养，内外邪凝结，引起局部麻木、隐痛、软组织结节等症状。早期治宜活血祛瘀、消肿止痛，中后期宜温经止痛、舒筋活络。陈旧性踝关节扭伤常见治疗方法分为保守治疗和手术治疗，韧带损伤较轻时，临床上常口服非甾体抗炎药、进行康复锻炼、体外冲击波等理疗，或运用正骨、理筋、中药熏洗、外敷、推拿、针灸等方法治疗。当反反复复扭伤致使韧带过于松弛引起关节不稳定、韧带严重撕裂或者断裂时，应手术治疗。

正骨理筋手法[3]是基于骨伤科“筋骨并重”和“骨正筋柔”的手法。陈旧性踝关节扭伤属于中医“筋伤”范畴，即骨错缝、筋出槽，其主要病机为经络闭阻、气滞血瘀，筋脉失养，正骨理筋手法治疗陈旧性踝关节扭伤能起到活血化瘀、消肿止痛，剥离粘连、滑利关节的作用，是最为有效的传统中医药疗法。

近年来随着对体外冲击波疗法（extracorporeal shock wave therapy，ESWT）的深入研究，ESWT 已成为肌肉骨骼系统疾病的保守替代疗法，如股骨外上髁炎、肌腱炎、肩周炎、足底筋膜炎、骨不连或愈合延迟、股骨头坏死、膝骨关节炎等，具有非侵入性、安全、有效、副作用小的优势。目前在骨伤科的应用也越来越广泛，它可以抑制疼痛信号的传递，减轻韧带、肌腱起止点炎症，促进微小血管再生、扩张，改善微循环，消除水肿，松解组织粘连，从而缓解疼痛、改善关节功能[4]。

基于以上背景和医疗工作中手法联合体外冲击波治疗骨伤科伤筋病的优良效果。本课题对正骨理筋手法联合体外冲击波治疗陈旧性踝关节扭伤的临床疗效进行研究，以期

中国知网 https://www.cnki.net

进一步明确该治疗方法对陈旧性踝关节扭伤的的疗效，为该方法在临床中用于治疗陈旧性踝关节扭伤的可能性、可靠性提供依据。

中国知网 https://www.cnki.net

第一部分 临床研究

一、研究对象

1 病例来源

在2020年1月到12月期间，就诊于甘肃中医药大学附属医院宋氏正骨科门诊的陈旧性踝关节扭伤的患者，共计纳入病例数为90例，均符合本研究纳入标准，对90例患者依照就诊时间顺序随机分为3组，每组30例。本研究合乎伦理学要求。

2 诊断标准

2.1 中医诊断标准

采用《中医诊疗方案》（2011年国家中医药管理局颁布）[5]，该诊疗方案将踝关节扭伤分为两个证型：气滞血瘀证（急性）和筋脉失养证（陈旧性）。本研究属于后者证型筋脉失养证：扭伤后期，关节持续隐痛，轻度肿胀，或可触及硬结，步行欠力。舌淡，苔白，脉弦细。

2.2 西医诊断标准

参照《实用骨伤科手册》中该病诊断标准[6]。①明确外伤史；②踝关节肿胀、酸痛乏力，关节活动时可有摩擦感，久行久立后及阴雨天时加重；③外踝前下方及内踝前外侧有肿胀、压痛，内翻、跖屈时活动可受限；④检查未发现骨折和脱位，X线摄片检查未见骨折迹象；⑤病程＞20d 未愈者。符合①②⑤或者①③⑤或者①③④⑤或者①②④⑤，可诊断为该病。

3 纳入标准

（1）就诊于甘肃中医药大学附属医院宋氏正骨科门诊第一诊断陈旧性踝关节扭伤患者；

（2）合乎以上诊断，病程超过20d，为单侧踝关节扭伤，且为外翻型；

（3）经X线、高频超声检查无骨折和韧带完全断裂；

（4）既往无皮肤病病史；

（5）年龄满足16周岁以上（包括16岁），65周岁以下（包含65周岁）；

（6）近期无口服药物及其他任何方式的治疗；

（7）可配合进行正骨理筋手法治疗及体外冲击波疗法治疗。

中国知网 https://www.cnki.net

4 排除标准

（1）合并有风湿、痛风、类风湿等疾病，踝关节活动被受到影响者；

（2）合并有特别严重的、危及生命的心、脑、肝、肾等原发疾病；

（3）治疗局部皮肤有伤口或破损，影响实施手法或冲击波治疗者；

（4）患者有出血性、严重心律失常、高血压等冲击波治疗禁忌症者；

（5）患者不能配合治疗，有认知障碍或精神疾病者。

5 脱落剔除标准

（1）医治期间，患者发生过敏、出血等不良反应或其他不可测情况，因而不能完成治疗者；

（2）患者中途因各种主观、客观原因要求退出治疗者；

（3）患者治疗期间未按研究要求，影响有效性评价者；

（4）资料不全，影响疗效评价者。

6 脱落病人的处理

当病人脱落时，尽可能与病人进行沟通，详细询问病人脱落原因及有无不良反应，并记录在病例资料采集册中，统计时按照脱落病例处理。

二、研究方法

1 分组方法

依照就诊顺序对 90 例患者随机分为 3 组：联合组、手法组、冲击波组，每组 30 例；分别采用正骨理筋手法联合体外冲击波治疗、正骨理筋手法治疗、体外冲击波治疗。

2 治疗设备

产品名称：体外冲击波治疗机；

规格型号：Swiss Dolor Clast；

注册证编号：国械注进 20162260354。见附录 2。

3 治疗方案

3.1 联合组

先采用体外冲击波进行治疗。见附录 2。

中国知网 https://www.cnki.net

首先冲击波参数设定为：压强 1.0-1.5 kPa，频率 13-15 Hz，冲击量 2000 次。患者坐于治疗床上，患肢踝关节充分暴露放在床上，在外踝前下方痛点、筋结及周围肌腱韧带等处涂抹耦合剂，准备开始治疗。首先在以上部位进行轻强度的击打，以让患者适应冲击波的作用方式，缓解紧张心理，然后根据患者忍受程度加强冲击波强度进行击打（以患者能忍受为度），每个部分 200 次，冲击总量到达 2000 次后完成治疗。

后采用正骨理筋手法进行治疗。见附录 2。

①患者坐于治疗床上，患肢踝关节充分暴露放在床上；医者与患者面对，坐于方凳上，一手自外踝后侧托住踝后部，一手拇指置于外踝前下方，余指放于内踝旋转按揉，拇指按揉部位为外踝前下方痛点、筋结及周围肌腱韧带，放松踝关节周围软组织约 7min。

②医者与患者面对而坐，以一手由下方托住足跟，以另一手握住患足跖跗部，两手徐徐用力牵拉，并缓缓地作旋转、内外翻及伸屈活动。令患者放松肌肉确感关节放松时，在屈伸活动中足部极度背伸时，骤然牵拉，有时可听到响声，以纠正由于外踝扭伤并发的踝关节错缝。

③患者伤肢在上侧卧在治疗床上，助手两手紧握伤侧小腿下部使脚踝固定不移动，医者两手相对，拇指在上拿住足部，作踝关节摇法，在牵引下先使足跖屈内翻，再将足背屈外翻，同时双手拇指向下按压伤处，最后以手拇指沿肌腱韧带走形作捋顺法，完成治疗。

疗程：每周一、三、五上午各一次，3 次/每周；治疗时间为 2 周。

3.2 手法组

采用正骨理筋手法进行治疗，方法同联合组手法治疗。见附录 2。

疗程：每周一、三、五上午各一次，3 次/每周；治疗时间为 2 周。

3.3 冲击波组

采用体外冲击波进行治疗，方法同联合组体外冲击波治疗。见附录 2。

疗程：每周一、三、五上午各一次，3 次/每周；治疗时间为 2 周。

4 观察指标

4.1 一般资料

性别、年龄、病位、病程。

4.2 VAS 疼痛评分

中国知网 https://www.cnki.net

视觉模拟评分法（visual analogue scale/sore，简称 VAS）评估患者疼痛程度灵活、方便、直观、可比性强。见附录 4。

4.3 Baird-Jackson(1987)踝关节评分

Baird-Jackson(1987)踝关节评分总分 100 分，将踝关节评分分为 7 个小项，每项又下列 4 级或 5 级的程度评分，评估较为细致，能比较客观评估患者的踝关节功能情况。见附录 5。

4.4 踝关节活动度（ROM）评分

踝关节活动度（ROM）评分 20 分。此评分参照 Kofoed 评分量表中的活动度选项。伸、屈各 5 分；旋后、旋前各 3 分；负重时内翻、外翻各 2 分。踝关节各种评分量表中，Kofoed 评分量表中的活动度最为详细，因此参照此量表中的该选项。见附录 6。

4.5 肿胀程度

参照《中药新药临床研究指导原则》[7]中软组织损伤症状分级量化表中的肿胀症状评估。测量方法为：用游标卡尺测量患侧和健侧踝关节直径，取其差值平均值。见附录 7。

5 疗效评价标准

疗效评价参照《中医病证诊断疗效标准》[8]制定。

①治愈：踝关节疼痛、肿胀等症状消失，活动功能正常；

②显效：肿胀消失，活动功能正常，内外踝处有轻度压痛，久行或阴雨天时偶有轻度不适；

③有效：肿胀部分时有消失，活动功能尚未正常，内外踝处有压痛，久行或阴雨天时有轻度不适；

④无效：疼痛、肿胀等症状存在，行动受限，阴雨天经常发作。

有效率=（治愈＋显效＋有效）／ n ×100%。

6 观察时间点

在治疗前、治疗 2 周后及治疗后 1 个月（随访）三个时间点上记录患者的视觉模拟疼痛评分（Visual analogue scale，VAS）、Baird-Jackson 踝关节评分、ROM 踝关节活动度评分及踝关节肿胀程度的情况来评估和量化疾病的缓解程度，最后评定三组的总体疗效。

中国知网 https://www.cnki.net

7 不良事件的处理

本研究中手法和体外冲击波治疗中，如出现皮肤破损、皮下淤青等任何不良反应，立即进行干预措施，必要时终止治疗。观察患者病情变化，指导合理用药，并做详细记录。

8 统计分析

①等级资料、计数资料分别采用多样本秩和检验、卡方分析；

②计量资料符合正态分布，采用单因素方差分析，组间计量资料比较有统计学差异，采用 LSD 法两两比较；不符合，采用多样本秩和检验；

③组内计量资料符合正态分布，采用配对样本 t 检验；不符合，采用配对样本秩和检验；

④所有数据运用$\bar{x} \pm s$表示，P＜0.05 有统计学差异。

中国知网 https://www.cnki.net

第二部分 结 果

1 纳入病例完成情况

纳入病例 90 例，手法组全部完成，联合组和冲击波组各有 1 例脱落，脱落原因均为治疗中因事不能完成治疗。

2 一般资料分析

88 例患者中男 30 例，女 58 例，年龄范围为 16-65 岁；损伤部位为右踝 46，左踝 42；病程范围为 3 周到 24 个月。经检验分析三组在性别、年龄、病位、病程分布情况无统计学差异（P＞0.05）。详情见表 1、表 2、表 3、表 4。

表 1 三组性别比较（例）

组别	例数	男性	女性	χ^2	P
联合组	29	10	19		
手法组	30	9	21	0.416	0.812
冲击波组	29	11	18		

注：经卡方检验，P＞0.05，无统计学差异，具有可比性。

表 2 三组年龄比较（$\bar{x} \pm s$，岁）

组别	例数	年龄（岁）	F	P
联合组	29	43.52±13.71		
手法组	30	40.10±13.91	0.461	0.632
冲击波组	29	41.31±13.84		

注：经单因素方差分析，P＞0.05，无统计学差异，具有可比性。

表 3 三组病位比较（例）

组别	例数	右	左	χ^2	P
联合组	29	14	15		
手法组	30	16	14	0.297	0.862
冲击波组	29	16	13		

注：经卡方检验，P＞0.05，无统计学差异，具有可比性。

表 4 三组病程比较（$\bar{x} \pm s$，月）

中国知网 https://www.cnki.net

组别	例数	病程（月）	Z	*P*
联合组	29	6.41±7.73		
手法组	30	6.15±7.86	0.421	0.810
冲击波组	29	6.36±8.95		

注：经多样本秩和检验，*P*＞0.05，无统计学差异，具有可比性。

3 三组治疗前各指标组间对比

三组治疗前 VAS 疼痛评分、Baird-Jackson 踝关节评分、ROM 踝关节活动度评分符合正态分布，且方差齐，采用单因素方差分析，肿胀程度采用多样本秩和检验；结果 *P* 值均＞0.05，表明治疗前以上观察指标无统计学差异。详情见表 5、表 6、表 7、表 8。

表 5 三组治疗前 VAS 疼痛评分比较（$\bar{x} \pm s$，分）

组别	例数	治疗前 VAS 疼痛评分	F	*P*
联合组	29	5.25±0.63		
手法组	30	5.21±0.56	0.643	0.528
冲击波组	29	5.38±0.55		

注：经单因素方差分析，*P*＞0.05，无统计学差异，具有可比性。

表 6 三组治疗前 Baird-Jackson 踝关节评分比较（$\bar{x} \pm s$，分）

组别	例数	治疗前 Baird-Jackson 踝关节评分	F	*P*
联合组	29	66.79±3.28		
手法组	30	67.90±2.82	1.091	0.340
冲击波组	29	67.55±2.68		

注：经单因素方差分析，*P*＞0.05，无统计学差异，具有可比性。

表 7 三组治疗前 ROM 踝关节活动度评分比较（$\bar{x} \pm s$，分）

组别	例数	治疗前 ROM 踝关节活动度评分	F	*P*
联合组	29	11.21±1.61		
手法组	30	11.10±1.58	0.143	0.867
冲击波组	29	11.31±1.49		

注：经单因素方差分析，*P*＞0.05，无统计学差异，具有可比性。

中国知网 https://www.cnki.net

表 8 三组治疗前肿胀程度比较（例）

组别	例数	无或者轻度	中度	重度	Z	*P*
联合组	29	18	8	3		
手法组	30	17	11	2	0.220	0.896
冲击波组	29	16	10	3		

注：经多样本秩和检验，*P*＞0.05，无统计学差异，具有可比性。

4 三组患者治疗前后各指标变化

4.1 VAS 评分比较

组内比较：三组组内 VAS 评分均不符合正态分布，采用配对样本秩和检验。①治疗后 2 周与治疗前比较：联合组 Z=-4.709、*P*=0.000，手法组 Z=-4.603、*P*=0.000，冲击波组 Z=-4.295、*P*=0.000；*P* 值均小于 0.05，有统计学差异。②治疗后 1 个月与治疗 2 周后比较：联合组 Z=-4.755、*P*=0.000，手法组 Z=-4.150、*P*=0.000，冲击波组 Z=-4.295、*P*=0.000；*P* 值均小于 0.05，有统计学差异。见表 9。

表 9 三组治疗前后 VAS 组内比较情况（$\bar{x} \pm s$，分）

组别	治疗前	治疗 2 周后	治疗后 1 个月
联合组	5.25±0.63	2.10±0.28	1.16±0.23
P		0.000#	0.000*
手法组	5.21±0.56	3.02±0.48	1.78±0.23
P		0.000#	0.000*
冲击波组	5.38±0.55	2.53±0.25	1.81±0.16
P		0.000#	0.000*

注：治疗 2 周后与治疗前比较，#*P*<0.05（联合组 Z=-4.709、*P*=0.000，手法组 Z=-4.603、*P*=0.000，冲击波组 Z=-4.295、*P*=0.000）；治疗后 1 个月与治疗 2 周后比较，**P*<0.05（联合组 Z=-4.755、*P*=0.000，手法组 Z=-4.150、*P*=0.000，冲击波组 Z=-4.295、*P*=0.000）。

组间比较：①三组治疗 2 周后 VAS 评分均不符合正态分布，经多样本秩和检验，*P*=0.000<0.05，有统计学差异。三组组间多重比较：联合组和手法组 Z=-6.846、*P*=0.000，联合组和冲击波组 Z=-3.475、*P*=0.002，手法组和冲击波组 Z=3.342、*P*=0.002；*P* 值均小于 0.05，有统计学差异。②三组治疗后 1 个月 VAS 评分均符合正态分布且方差齐，经

中国知网 https://www.cnki.net

单因素方差分析，F=88.762、*P*=0.000<0.05，有统计学差异。LDS 法两两比较：联合组和手法组 *P*=0.000<0.05、联合组和冲击波组 *P*=0.000<0.05，有统计学差异；手法组和冲击波组 *P*=0.503>0.05，无统计学差异。见表 10。

表 10 三组治疗前后 VAS 组间比较情况（$\bar{x}\pm s$，分）

组别	治疗前	治疗 2 周后	治疗后 1 个月
联合组	5.25±0.63	2.10±0.28◎	1.16±0.23&
手法组	5.21±0.56	3.02±0.48※	1.78±0.23▲
冲击波组	5.38±0.55	2.53±0.25®	1.81±0.16□
P	0.528	0.000#	0.000*

注：治疗 2 周后三组组间比较，#*P*<0.05，组间多重比较，◎※*P*、◎®*P*、※®*P* 均小于 0.05；治疗后 1 个月三组组间比较，**P*<0.05，LDS 法两两比较，&▲*P*<0.05、&□*P*<0.05、▲□*P*>0.05。

4.2 Baird-Jackson 踝关节评分比较

组内比较：三组组内 Baird-Jackson 踝关节评分均不符合正态分布，采用配对样本秩和检验。①治疗后 2 周与治疗前比较：联合组 Z=4.737、*P*=0.000，手法组 Z=4.819、*P*=0.000，冲击波组 Z=4.719、*P*=0.000；*P* 值均小于 0.05，有统计学差异。②治疗后 1 个月与治疗 2 周后比较：联合组 Z=4.743、*P*=0.000，手法组 Z=4.319、*P*=0.000，冲击波组 Z=4.762、*P*=0.000；*P* 值均小于 0.05，有统计学差异。见表 11。

表 11 三组治疗前后 Baird-Jackson 踝关节评分组内比较情况（$\bar{x}\pm s$，分）

组别	治疗前	治疗 2 周后	治疗后 1 个月
联合组	66.79±3.28	86.31±3.29	93.10±3.15
P		0.000#	0.000*
手法组	67.90±2.82	82.10±3.19	86.93±2.84
P		0.000#	0.000*
冲击波组	67.55±2.68	77.13±2.71	82.10±2.73
P		0.000#	0.000*

注：治疗 2 周后与治疗前比较，#*P*<0.05（联合组 Z=4.737、*P*=0.000，手法组 Z=4.819、*P*=0.000，冲击波组 Z=4.719、*P*=0.000）；治疗后 1 个月与治疗 2 周后比较，**P*<0.05（联合组 Z=4.743、*P*=0.000，手法组 Z=4.319、*P*=0.000，冲击波组 Z=4.762、*P*=0.000）。

中国知网 https://www.cnki.net

组间比较：三组治疗后组间 Baird-Jackson 踝关节评分均符合正态分布，且方差齐，采用单因素方差分析比较：治疗 2 周后 F=64.767、*P*=0.000，治疗后 1 个月 F=103.909，*P*=0.000；*P* 值均小于 0.05，有统计学差异。LSD 法两两比较，均得出 *P*=0.000<0.05，有统计学差异。见表 12。

表 12 三组治疗前后 Baird-Jackson 踝关节评分组间比较情况（$\bar{x} \pm s$，分）

组别	治疗前	治疗 2 周后	治疗后 1 个月
联合组	66.79±3.28	86.31±3.29$^{◎}$	93.10±3.15$^{\&}$
手法组	67.90±2.82	82.10±3.19$^{※}$	86.93±2.84$^{▲}$
冲击波组	67.55±2.68	77.13±2.71$^{®}$	82.10±2.73$^{□}$
P	0.340	0.000$^{\#}$	0.000*

注：治疗 2 周后三组组间比较，$^{\#}P$<0.05，LDS 法两两比较，$^{◎※}P$、$^{◎®}P$、$^{※®}P$ 均小于 0.05；治疗后 1 个月三组组间比较，$^{*}P$<0.05，LDS 法两两比较，$^{\&▲}P$、$^{\&□}P$、$^{▲□}P$ 均小于 0.05。

4.3 ROM 踝关节活动度评分比较

组内比较：三组组内 ROM 踝关节活动度评分均不符合正态分布，采用配对样本秩和检验。①治疗后 2 周与治疗前比较：联合组 Z=-4.669、*P*=0.000，手法组 Z=-4.873、*P*=0.000，冲击波组 Z=-5.176、*P*=0.000；*P* 值均小于 0.05，有统计学差异。②治疗后 1 个月与治疗 2 周后比较：联合组 Z=-3.729、*P*=0.001<0.05，手法组 Z=-4.920、*P*=0.000<0.05，有统计学差异；冲击波组 Z=-1.853、*P*=0.129>0.05，无统计学差异。见表 13。

表 13 三组治疗前后 ROM 踝关节活动度评分组内比较情况（$\bar{x} \pm s$，分）

组别	治疗前	治疗 2 周后	治疗后 1 个月
联合组	11.21±1.61	15.76±1.24	18.14±1.06
P		0.000$^{\#}$	0.001$^{\&}$
手法组	11.10±1.58	15.13±1.76	17.07±1.46
P		0.000$^{\#}$	0.000$^{▲}$
冲击波组	11.31±1.49	14.34±1.42	15.28±1.19
P		0.000$^{\#}$	0.129$^{□}$

中国知网 https://www.cnki.net

注：治疗 2 周后与治疗前比较，#*P*<0.05（联合组=Z-4.669、*P*=0.000，手法组 Z=-4.873、*P*=0.000，冲击波组 Z=-5.176、*P*=0.000）；治疗后 1 个月与治疗 2 周后比较，&*P*<0.05（联合组 Z=-3.729、*P*=0.001），▲*P*<0.05（手法组 Z=-4.920、*P*=0.000），□*P*>0.05（冲击波组 Z=-1.853、*P*=0.129）。

组间比较：①三组治疗 2 周后组间 ROM 踝关节活动度评分符合正态分布，且方差齐，经单因素方差分析：F=6.536、*P*=0.002<0.05，有统计学差异。LSD 方法两两比较：联合组和手法组 *P*=0.111>0.05，无统计学差异；联合组和冲击波组 *P*=0.001<0.05、手法组和冲击波组 *P*=0.046<0.05，有统计学差异。②三组治疗后 1 个月组间 ROM 踝关节活动度评分不符合正态分布，经多样本秩和检验：*P*=0.000<0.05，有统计学差异。三组组间多重比较：联合组和手法组 Z=2.562、*P*=0.031，联合组和冲击波组 Z=6.436、*P*=0.000，手法组和冲击波组 Z=3.928、*P*=0.000；*P* 值均小于 0.05，有统计学差异。见表 14。

表 14 三组治疗前后 ROM 踝关节活动度评分组间比较情况（$\bar{x} \pm s$，分）

组别	治疗前	治疗 2 周后	治疗后 1 个月
联合组	11.21±1.61	15.76±1.24◎	18.14±1.06&
手法组	11.10±1.58	15.13±1.76※	17.07±1.46▲
冲击波组	11.31±1.49	14.34±1.42®	15.28±1.19□
P	0.867	0.002#	0.000*

注：治疗 2 周后三组组间比较，#*P*<0.05，LSD 法两两比较，◎※*P*>0.05、◎®*P*<0.05、※®*P*<0.05；治疗后 1 个月三组组间比较，**P*<0.05，组间多重比较，&▲*P*、&□*P*、▲□*P* 均小于 0.05。

4.4 肿胀程度比较

联合组和手法组组内比较：采用多样本秩和检验，联合组 Z=21.323、*P*=0.000<0.05，手法组 Z=15.648、*P*=0.000<0.05，有统计学差异。治疗后 2 周与治疗前比较：联合组 Z=3.804、*p*=0.000<0.05，手法组 Z=3.259、*p*=0.003<0.05，有统计学差异。治疗后 1 个月与治疗前比较：联合组 Z=4.169、*P*=0.000<0.05，手法组 Z=3.572、*P*=0.001<0.05，有统计学差异。治疗后 1 个月与治疗 2 周后比较：联合组 Z=0.365、*P*=1.000>0.05，手法组 Z=0.313、*P*=1.000>0.05，无统计学差异。见表 15、表 16。

表 15 联合组组内肿胀程度比较（例）

时间	无或者轻度	中度	重度	Z	*P*
治疗前	18	8	3		

中国知网 https://www.cnki.net

治疗 2 周后	28	1	0	21.323	0.000
治疗后 1 个月	29	0	0		

注：经多样本秩和检验，*P*<0.05，有统计学差异。

表 16 手法组组内肿胀程度比较（例）

时间	无或者轻度	中度	重度	Z	*P*
治疗前	17	11	2		
治疗 2 周后	27	3	0	15.648	0.000
治疗后 1 个月	28	2	0		

注：经多样本秩和检验，*P*<0.05，有统计学差异。

冲击波组组内比较：采用多样本秩和检验，Z=2.641、*P*=0.267>0.05，无统计学差异。见表 17。

表 17 冲击波组组内肿胀程度比较（例）

时间	无或者轻度	中度	重度	Z	*P*
治疗前	16	10	3		
治疗 2 周后	19	10	0	2.641	0.267
治疗后 1 个月	21	8	0		

注：经多样本秩和检验，*P*>0.05，无统计学差异。

治疗 2 周后三组组间比较：采用多样本秩和检验得出 Z=11.495、 *P*=0.003<0.05，有统计学差异。三组组间多重比较：联合组和手法组 Z=-0.684、*P*=1.000>0.05，有统计学差异；联合组和冲击波组 Z=-3.213、*P*=0.004<0.05，手法组和冲击波组 Z=-2.556、*P*=0.032<0.05，无统计学差异。见表 18。

表 18 三组治疗 2 周后组间肿胀程度比较（例）

组别	例数	无或者轻度	中度	重度	Z	*P*
联合组	29	28	1	0		
手法组	30	27	3	0	11.495	0.003
冲击波组	29	19	10	0		

注：经多样本秩和检验，*P*<0.05，有统计学差异。

中国知网 https://www.cnki.net

治疗后 1 个月三组间比较：采用多样本秩和检验得出 Z=11.816、 P=0.003<0.05，有统计学差异。三组组间多重比较：联合组和手法组 Z=-0.802、P=1.000>0.05，无统计学差异；联合组和冲击波组 Z=-3.291、P=0.003<0.05，手法组和冲击波组 Z=-2.517、P=0.036<0.05，有统计学差异。见表 19。

表 19 三组治疗后 1 个月组间肿胀程度比较（例）

组别	例数	无或者轻度	中度	重度	Z	P
联合组	29	29	0	0		
手法组	30	28	2	0	11.816	0.003
冲击波组	29	21	8	0		

注：经多样本秩和检验，P<0.05，有统计学差异。

5 三组总体疗效对比

三组总有效率联合组 93.10％，手法组 86.67％，冲击波组 75.86％。采用多样本秩和检验 Z=23.792、P=0.000<0.05，有统计学差异。三组组间多重比较：联合组和手法组 Z=-2.402、P=0.049，联合组和冲击波组 Z=-4.878、P=0.000，手法组和冲击波组 Z=-2.496、P=0.038；P 值均小于 0.05，有统计学差异。见表 20。

表 20 三组患者治疗后 1 个月临床疗效比较（例）

组别	例数	痊愈	显效	有效	无效	总有效率（％）
联合组	29	8	13	6	2	93.10
手法组	30	5	11	10	4	86.67
冲击波组	29	3	9	10	7	75.86

注：等级资料多样本比较，经 Kruskal-Wallis 检验，P<0.05（联合组和手法组 Z=-2.402、P=0.049<0.05，联合组和冲击波组 Z=-4.878、P=0.000<0.05，手法组和冲击波组 Z=-2.496、P=0.038<0.05）。

6 不良事件

三组患者治疗期间均未出现严重的不良事件。

中国知网 https://www.cnki.net

第三部分 讨 论

1 现代医学对陈旧性踝关节扭伤的认识

踝关节扭伤占全身关节扭伤的 80%以上[1]，是日常生活与运动中的常见疾病。本病可发生于任何年龄，女性多见，多因行走于不平路面、下楼梯或者跑跳、穿高跟鞋、体育训练时动作不协调、行走不稳导致踝跖屈位时，突然足强力内翻或外翻，引起外侧副韧带或者内侧副韧带牵拉伤，急性期以踝关节明显肿胀疼痛、跛行、功能障碍、皮肤青紫为特征，大部分患者经初始保守治疗，如早期活动指导、理疗、绷带、支具等，可完全恢复；如未能得到及时治疗或治疗方法不得当，使损伤的韧带不能及时恢复，会造成组织的粘连、增生和慢性炎症的存在，患者踝关节出现慢性疼痛、酸困无力、肿胀、不稳定感、担心扭脚、活动不利等症状，发展为陈旧性踝关节扭伤。

1.1 解剖、稳定性及扭伤机制

踝关节是滑车关节，连接小腿和足，由胫腓骨远端及距骨构成。胫腓骨远端组成下胫腓联合，下胫腓联合并非是真正的关节，它不具有关节软骨和关节囊，是由纤维较粗，韧性较强的胫腓前后韧带及骨间膜所连接，故未称关节，而叫联合。胫骨远端关节面呈凹形，逐渐向内凸起，延续向下形成内踝，腓骨远端与胫骨远端组成下胫腓联合，被坚强的韧带所约束，再向外下方隆起，形成外踝。胫骨远端后唇较长，称后踝，能防止胫骨向前滑出。距骨分为体、颈、头三部分，体与胫腓骨下端组成踝关节，体的中央凹下，两边凸起成鞍形，称滑车，前面宽而后面窄，防止距骨后移。

维持踝关节前后方向的稳定包括关节囊后部及侧副韧带后部纤维等。维持踝关节水平的稳定包括完整的踝与胫腓下韧带，强有力的内、外侧副韧带等。当踝关节背屈运动时，距骨体沿胫骨远端之嵴向背侧滑动，自内下滑向外上，待距骨体宽头完全进入踝穴后，约背屈 25 度，至极限时，踝关节最稳定。当踝关节跖屈运动时，约自中立位到 45 度左右，距骨体较窄部分进入踝穴，但由于距骨远较踝穴窄，活动度和松动程度非常明显，踝关节不稳定[9]。

踝关节放松时，踝关节呈轻微跖屈反向位置，在走路迈步阶段到踝关节移动到该位置时，踝关节内翻扭伤危险性较高。此时，踝关节的稳定性主要靠外侧副韧带提供，如踏空或遇微小阻力便会踝关节进一步跖屈内翻造成外侧的软组织如肌腱、韧带等的过度牵拉，引起扭伤。足的跖屈内翻中，最先最严重的损伤是距腓前韧带，如若力量过大，

中国知网 https://www.cnki.net

跟腓韧带也会损伤，距腓后韧带损伤极少。如果踝关节发生过扭伤并且治疗不彻底，其再发的危险性会增加，这是因为以前踝关节扭伤会导致部分传人神经的阻滞，这会减弱踝关节的神经肌肉控制能力，也就是本体感觉产生了一定程度的损伤。本体感觉能使人体精确地感受位置，它的损伤增加踝关节扭伤的危险因素，对于突发踝关节内翻的保护性反射反应不能及时发生[10]，这是导致习惯性踝关节扭伤或者慢性踝关节不稳的重要因素。

踝关节外侧副韧带易伤的主要原因是外踝较内踝长，外侧副韧带较内侧副韧带弱[11]，距骨前宽后窄，足跖屈时关节的活动度大并且不稳定，并且足屈与内翻的肌力大于足伸与外翻，再加上足底受到刺激后出现跖屈和必伴有内翻的防御反射（无条件反射），因此足易内翻扭伤外侧副韧带[12]。

1.2 症状、诊断及常见治疗

由于慢性炎症的存在，陈旧性踝关节扭伤的患者踝关节会反复疼痛、酸困无力、甚至肿胀，尤其是长时间步行或者跑步时更加明显，同时会有关节不稳定、运动不利及不同程度的活动受限。如若患者不及时治疗，反复的活动障碍、关节磨损会使踝关节关节面的软骨破坏，造成创伤性关节炎，进一步会加快关节退行性改变，骨赘形成，最终发展为骨性关节炎[13]。

陈旧性踝关节扭伤的诊断要从病史、症状、查体、辅助检查等方面作综合判断。病史方面往往可追溯到患者有一次甚至多次的踝关节扭伤经历，症状上基本有踝关节慢性疼痛、酸困无力、肿胀、不稳定感、担心扭脚、活动不利等。查体时可在踝关节周围触及压痛、泥样肿胀或是筋结，周围肌肉或有僵硬、紧张感，踝关节的内外翻试验阳性、抽屉试验阳性等。辅助检查对于陈旧性踝关节扭伤的诊断很重要，主要有 X 线片、肌骨彩超、MRI 等。X 线片除了可排除骨折、脱位等，还可以观察胫距关节内外侧间隙是否一致，对于陈旧性损伤，通常选择踝关节正位内翻应力位，但 X 线片不能直接显示韧带损伤情况；肌骨彩超价格低廉，且为无创性检查，用于陈旧性踝关节扭伤可以诊断韧带的损伤情况，尤其对韧带的完全撕裂可靠性高；MRI 检查对诊断软组织的损伤优于其他辅助检查，但其价格相对较高，且耗时长，门诊上处理病人一般很少应用。

陈旧性踝关节扭伤的现代医学治疗分为保守和手术两种方式。保守治疗针对侧副韧带未完全撕裂者，主要有非甾体抗炎药、封闭疗法、制动休息、康复锻炼、理疗、低能

中国知网 https://www.cnki.net

量的体外冲击波等疗法。对于踝关节侧副韧带撕裂严重或者完全断裂者，采用手术治疗，主要有踝关节韧带解剖缝合术或者韧带重建手术，重建手术又有非解剖重建和解剖重建，重建移植来的肌腱有自体肌腱和异体肌腱之分[14]。

2 祖国医学对陈旧性踝关节扭伤的认识

中医学认为，本病属“筋伤、痹症”范畴，多因跌仆闪扭或外力直接打击造成足踝部经筋扭挫、气血凝滞，因失治误治，复感外邪，造成踝部邪瘀交阻，气滞血瘀，痹阻经脉，筋肉失养发为本病，这与骨伤科的“伤筋和错缝”关系密切，后期转化为慢性筋伤与“痹症”相关。下面主要从筋骨病机、伤筋和错缝、痹症来论述陈旧性踝关节扭伤的祖国医学认识。

2.1 筋骨病机与伤筋和错缝

肢体的运动是依靠筋骨协同完成，筋与骨相互依存，相互为用。筋束骨、护骨、依骨、养骨，在维系骨骼关节的完整、健康与功能方面，起着主要作用；骨张筋，为筋提供动力支点和滋养，骨强髓充则筋壮。肝主筋，筋有连缀四肢百骸、主司关节运动的作用。肾主骨，藏精，而精能生髓，髓居骨中，充养骨，骨有支持躯体、保护内脏的功能，筋骨依赖肝肾和气血的滋养而发挥作用[15,16]。

中医“伤筋”中的“筋”是对软组织的统称[17]，包括了肌腱、韧带、滑膜、滑囊、软骨等，因此中医的伤筋病很广泛；错缝主要指骨关节的轻度错位和对合不齐。伤筋和错缝往往相互关联、密不可分，一般来说，伤筋不一定错缝，但错缝一定有筋伤。治疗上，伤筋中如果有肌腱、韧带的大部分断裂，这往往需要手术缝合或者修复，一般的损伤如有骨错缝，应该先纠正骨关节的微小错缝，这样可以为筋的恢复提供良好的外部坏境，促进伤筋病的快速恢复。错缝的治疗一般是正骨手法的运用，正骨手法可以滑利关节，纠正微小错缝，它的作用是直接而立杆见影的[18]。

2.2 痹症

痹症是人体正气不足，又感受风、寒、湿、热等外邪引起躯体四肢疼、肿、酸、麻、困，甚或活动不利的病证[19]。痹症病因主要有正虚、外邪和痰瘀。

正虚乃是人体正气不足，不能抵御风寒湿等外部邪气而导致的痹症[20]。这其中大体上有三个原因，其一是先天禀赋不足。比如骨伤科小儿膝痹鹤膝风的治疗，清代名医喻昌在其所著的《医门法律》中有曰：“非必为风寒湿所痹，多因先天所禀，肾气衰薄，……”。

中国知网 https://www.cnki.net

其二是劳逸过度。持续行走或长期站立则伤筋损骨，经常躺卧或持续端坐则伤气损肉，这些行为易导致颈腰膝踝等部位痹症的发生。其三是大病产后。罹患重病后，体虚易感各种病邪，妇女胎产后，行经不调，情志不舒，亏肝损脾，也易感外邪杂气，此时风寒湿邪乘机而入发为痹症。

外邪的侵袭，是人体痹症发生的主要外在原因。其一、四时气候出现异常，当热不热，当寒不寒，或当热过热，当寒过寒，人体不能及时适应而为痹症，如太阳在泉时，寒淫旺盛，寒气长久客居人体，导致腰部及以下股胫足膝疼痛、屈伸不利。其二、居处环境不佳、生活起居不慎而为痹症，如居所环境寒冷、潮湿，不见日光，则易得寒痹和湿痹，居所环境炎热似火，热多寒少，则易得热痹。另外还有风痹，若坐卧当风，长久迎风而吹，会导致局部皮肤的血液脉络不通，血凝于肤而为风痹。清代医家叶天士最早提出“暑伤气，湿热邪气入络为痹”的概念，被称作“暑痹”。就陈旧性踝关节扭伤所导致的痹症而言，寒湿痹为其主要外邪。

痰瘀致痹症，饮食所伤，嗜食肥甘厚味，为导致痰浊的重要原因，脾主运化，胃主受纳，脾胃所伤，水湿阻滞，凝结为痰浊，痰浊再阻塞经络，使经络闭塞不通，不通则痛，最终发为痹症。踝关节扭伤就是日常生活中最常见的外伤致瘀成痹，如果意外跌扑闪挫某个部位，气血在此部位必定受扰壅聚，气聚血亦聚，进而成瘀为痹。

痹症的病机概括为“正虚、邪侵、不荣、不通”这八个字。正虚起决定作用，邪侵是必要条件，不荣、不通是结果产物。正虚的过程包括营气和卫气失去各自的运行规律，不能密腠理、抗外邪；气血虚弱不能行其濡养机体的作用；在这里，营气可以泌津注脉为血，气虚则卫气必虚，他们不可分割，相互影响。营卫失调气血不足会导致肝肾脾三脏衰弱，肝主筋、骨主骨、脾主肌肉四肢，筋骨肌肉四肢失去濡养，此时若出现外邪侵袭人体这一条件，则导致气血经络闭塞不通，不通则痛，最终发为痹症。痹症的发展转变，风寒湿热之邪侵袭人体，根据人的先天禀赋不同，或从阳化热，或从阴化寒，分为寒热。《素问·痹论》有提到寒者，阳少阴多，与病相益故寒，热者，阳多阴少，阳遭阴故热。热炼津、寒凝津、湿停聚，皆可成痰；进一步邪胜正虚，不荣不通并现；痹久正气更虚，痰饮更甚，瘀血更瘀，这正如清代王清任《医林改错》曰：“元气即虚……必停留而瘀”[21]。陈旧性踝关节扭伤患者脚踝酸困、乏力，走则加重，休息好转，患病愈久，虚与瘀夹杂愈重，恢复愈慢。

中国知网 https://www.cnki.net

3 正骨理筋手法

正骨理筋手法[3]是基于骨伤科“筋骨并重”和“骨正筋柔”的手法。它广泛运用于临床，是最为有效的传统中医药疗法，具有疗效好，痛苦小，方法简便的特点，深受广大患者的欢迎。

3.1 正骨理筋手法的作用

正骨理筋手法主要有以下作用[22,23]：①正骨复筋作用，通过手法的作用使发生错缝的骨关节对合如初，使移位的筋健恢复如旧，为疾病的快速愈合提供良好的恢复坏境。②活血理气作用，筋骨的损伤会导致气血瘀滞，如《正体类要》所云：“且肢体损于外，则气血伤于内……”。正骨理筋手法可以使疼痛肿胀减轻或消失，有着有效的活血化瘀、理气散滞的作用。③通络舒筋作用，正骨理筋手法可以解除瘀闭、舒筋解挛，对肌肉的筋结凝聚、关节的屈伸不利有良好的治疗作用。④消肿散结作用，正如《正骨心法要旨》云：“按其经络，以通郁闭之气；摩其壅聚，以散瘀结之肿”，正骨理筋手法可散瘀结肿胀。

3.2 正骨理筋手法的要求及操作

手法要求[24]均匀、有力、持久、柔和从而达到深透的目的，正如古人云：“筋喜柔而不喜刚，骨喜刚而不喜柔”。所以手法要以柔为主，柔中有刚，刚柔相济稳准熟练，才能起到治疗作用，否则粗暴的手法只会加重损伤，增加患者痛苦。手法操作，临床一般分为三个阶段：①准备阶段主要选用揉法、按法、摩法等具有行气活血、镇静止痛的轻柔手法，以放松肌肉、松弛关节，缓解紧张的状态，为第二阶段的手法作准备。②正骨理筋阶段应根据诊断选用一些针对病症的治疗手法，如摇法、旋转法、板法、弹筋法等。③结束阶段一般运用轻柔舒筋的手法以缓解第二阶段强刺激后的局部反应，如抖散手法等。

3.3 正骨理筋手法治疗陈旧性踝关节扭伤的临床研究

陈旧性踝关节扭伤又称“踝缝伤筋”，因胫距关节受外力而造成微小移位，且周围韧带、肌腱等损伤引起部分功能障碍和疼痛。正骨理筋手法使关节错移回归的同时，筋腱的移位也多能同时回归，关节、筋腱的回归，有利于损伤的快速恢复。陈兆军等[25]研究表明清宫外踝理筋手法在减缓疼痛、改善踝关节功能方面疗效明显，且好于功能疗法锻炼治疗组，但二者均在距骨倾斜度改善方面与治疗前相比虽然有改善，但无统计学意义，

中国知网 https://www.cnki.net

说明其在改变踝关节解剖结构方面效果不确切，手法针对功能性的陈旧性踝关节不稳效果更佳。梁国富[26]等治疗陈旧性踝关节扭伤干预组给予正骨理筋手法结合针刺，常规组给予电磁波结合针刺治疗。结论为运用理筋正骨手法联合针刺治疗陈旧性踝关节扭伤，效果明显，有效率为 90％。翟东旺[27]运用理筋手法联合中药熏洗治疗陈旧性踝关节扭伤，联合治疗方法为治疗组，对照组采用中医熏洗，结果前者各项观察指标优于后者。俞益火[28]等运用中医理筋正骨手法治疗慢性踝关节损伤 53 人，常规康复组 26 例采用功能锻炼、关节松动术、物理因子疗法，联合组 27 例在常规康复组的基础上施加中医理筋正骨手法，连续治疗 1 个月后，观察治疗前后的疼痛评分、AOFAS 评分、关节肿胀值及活动度，结果显示联合组各项指标均好于常规康复组，中医理筋正骨手法可以有效缓解肿胀、疼痛，改善活动度及功能，疗效好。

4 体外冲击波

体外冲击波疗法作为西医的治疗手段。ESWT 已成为肌肉骨骼系统疾病的保守替代疗法，如股骨外上髁炎、肌腱炎、肩周炎、足底筋膜炎、骨不连或愈合延迟、股骨头坏死、膝骨关节炎等，具有非侵入性、安全、有效、副作用小的优势。目前在骨伤科的应用也越来越广泛，它可以抑制疼痛信号的传递，减轻韧带、肌腱起止点炎症，促进微小血管再生、扩张，改善微循环，消除水肿，松解组织粘连，从而缓解疼痛、改善关节功能[4]。

4.1 体外冲击波的作用

体外冲击波主要有以下作用：①高密度组织裂解作用[29]，冲击波具有能量，它在不同介质中传播时会产生应力，在应力作用下可以使高密度组织发生裂解。②组织损伤再修复作用[30-34]，体外冲击波可以增加损伤软组织的愈合能力，刺激血管再生，增加营养供给，加快软组织损伤的修复作用。③镇痛及神经末梢封闭作用[35-37]，体外冲击波可直接抑制神经末梢细胞，改变其周围化学介质的组成，影响痛觉感受器的接收频率，这样使它的敏感性降低，神经传导受阻，缓解疼痛。④炎症及感染控制作用[38-41]，体外冲击波可以增加细胞内外离子的移动速度，加快代谢反应，炎性因子在代谢过程中被清除和吸收，慢性炎症可以减轻消退，另外有研究发现低强度冲击波对细菌有抑制作用，在一定程度上可以控制感染。⑤组织粘连松解作用[42]，当冲击波在不同的介质（肌腱、韧带、脂肪、骨骼）中传播时可以生产应力作用，该应力方向不同，有挤压组织的压应力和拉

中国知网 https://www.cnki.net

伸组织的拉应力，它们的共同作用下可以引起组织间的松解，毛细血管循环的加快及增加组织细胞的摄氧量和细胞弹性形变，最终达到分离粘连，伸展肌肉挛缩的目的。⑥扩张血管和血管再生作用[43-44]，通过松解组织粘连，疏通闭塞的微细血管，来促进血液流动，提高代谢能力，加快炎症吸收，消退肿胀，减轻疼痛。体外冲击波可通过对血管内皮细胞生长因子（VEGF）的作用，促进血管再生，增加组织血供，减轻或缓解相关疾病。

4.2 体外冲击波治疗肌腱、韧带方面的研究进展

体外冲击波治疗肌腱、韧带的临床报道很多，不少临床研究都肯定了其在治疗肌腱、韧带损伤方面有较好效果[45-49]。体外冲击波治疗肌腱、韧带损伤，不仅通过改善循环代谢、释放镇痛因子起到镇痛效果，还可刺激骨骼表面成骨细胞的生长成熟，促进软骨等组织再生[50]。研究发现冲击波可降低如白细胞介素-1β（IL-1β）和肿瘤坏死因子-α（TNF-α)等炎症因子的水平从而起到抗炎作用[51,52]；还可通过上调血管内皮生长因子（VEGF）、骨形态发生蛋白 2（BMP-2）的表达促进缺血坏死股骨头中新生血管和软骨下骨的形成[53,54]；通过干预骨髓间充质干细胞合成、分泌具有活性的胰岛素样生长因子（IGF-1）、转录生长因子（TgF-β）在不同增殖时期的表达而促进成骨细胞的成熟以促进骨、软骨组织的生长发育和重建[55]。为了进一步证明其对肌腱的治疗作用，有学者通过动物实验研究[56]发现经体外冲击波治疗后，大鼠肌腱细胞明显增加，肌腱内 VEGF 含量在 2 周时达到最高峰，修复过程中未见明显瘢痕组织形成，表明体外冲击波能最大程度地修复肌腱功能。另有实验证明体外冲击波可以增加胶原蛋白I、III[57]，下调基质金属蛋白酶（MMPs-1、3、13）水平[58,59]，从而发挥修复组织、抗炎作用。综上，我们可以推测体外冲击波可以作为力学信号通过刺激肌腱、韧带组织使得物理信号转化为生物化学信号从而发挥治疗作用。

4.3 体外冲击波治疗陈旧性踝关节扭伤的临床研究

体外冲击波可以抑制疼痛信号的传递，减轻韧带、肌腱起止点炎症，促进微小血管再生、扩张，改善微循环，消除水肿，松解组织粘连，从而缓解疼痛、改善关节功能，鉴于此，体外冲击波疗法已经逐渐运用于治疗踝关节疾病方面。刘辉[60]等用冲击波、关节粘连松解术和肌肉能量技术治疗踝关节僵硬的 26 例患者，治疗 1 个月后，Barthel 指数、AOFAS 评分和关节活动度均明显好转，表明冲击波可以改善软组织深层的黏弹性、

中国知网 https://www.cnki.net

松解组织粘连、促进局部的血液循环，可以作用于软组织深层，弥补手法的不足。李祥雨等[61]对姜劲挺教授治疗踝关节外侧副韧带损伤应用冲击波经络击打法进行探讨，将正骨理筋手法与体外冲击波相联合，为临床治疗本病提供了新的切实可行的疗法。周家彬[62]运用体外冲击波治疗 7 位踝关节陈旧性扭伤患者，均来自散打运动员，7 位患者踝关节损伤后因治疗不善遗留有疼痛症状，7 位患者治疗 1 个月后疼痛均减轻， 活动情况均改善。乔雅馨[63]等根据对 400 例踝关节外侧副韧带损伤患者的超声诊断，依据对侧副韧带的损伤程度制定体外冲击波治疗方案，评估疗效，来说明体外冲击波对韧带损伤治疗的可行性。刘怡梦[64]等运用体外冲击波对 58 例陈旧性踝扭伤患者进行治疗，对照组采用超声波治疗，治疗组运用体外冲击波疗法，在治疗前、治疗 2 周后、治疗 1 个月后评估踝部 VAS 评分、Kofoed 评分和肿胀程度，结果治疗组优于对照组，体外冲击波治疗可以有效缓解慢性疼痛并可改善踝关节功能。都本才[65]对 40 名踝关节软组织撞击综合征的陆军士兵运用瑞士 EMS Dolor Clast 体外冲击波进行治疗，评估治疗前后的 VAS 及 AOFAS 评分情况，结果显示体外冲击波可以缓解疼痛，恢复踝关节功能，疗效比封闭疗法好，且无副作用。王荷琴[66]等对郑大五附院的 80 例急性踝关节扭伤 24 小时后采用美国 DJO 低能量冲击波治疗，患者经 1-6 天治疗后，总有效率为 100%。结论低能量冲击波、蜡疗有利于踝关节扭伤后的康复。王筱锋[67]等用发散式冲击波治疗郑州市中医院陈旧性踝关节扭伤 38 例，每周治疗 2 次，总共治疗 1 个月，结果治愈 27 例，总有效率为 92.1%。

5 正骨理筋手法联合体外冲击波治疗

正骨理筋手法和体外冲击波治疗陈旧性踝关节扭伤各有优势，前者可以纠正踝关节的微小骨错缝和肌腱、韧带的移位，为踝关节恢复提供更好的环境，另外手法还可以松解粘连组织，活血化瘀，消肿散结，舒筋通络。后者可以镇痛，消除炎症，裂解筋结，松解组织粘连，促进微小血管生成，加快软组织修复。前者属于传统的中医药疗法，后者属于最新的西医治疗手段。将两者结合起来治疗疾病是一种简单便捷、行之有效的方式。两者同属外治法，且都属于无创治疗，简单便捷，效果好且副作用少，同比针灸、封闭、手术等其他有创治疗有独特的优势，且相比口服非甾体抗炎药无胃肠道副作用，安全性更高。

6 临床研究数据及疗效分析

中国知网 https://www.cnki.net

6.1 VAS 评分比较

组内比较：三组治疗 2 周后和治疗后 1 个月 VAS 评分比较均有统计学差异，且评分降低。说明三组治疗陈旧性踝关节扭伤在疼痛方面均有效果。

组间比较：三组治疗前 VAS 评分比较无统计学差异。治疗 2 周后，三组比较有统计学差异，且评分降低，三组组间多重对比，*P* 值均小于 0.05，有统计学差异；治疗后 1 个月，三组比较有统计学差异，且评分降低，LSD 法两两比较，手法组和冲击波组 $P=0.503>0.05$。说明治疗 2 周后三组在疼痛方面均有疗效，且联合组>手法组>冲击波组；治疗后 1 个月三组在疼痛方面均有疗效，但联合组和手法组在疼痛方面的差异不明显，不具有统计学差异。

分析比较表明在治疗期间联合组在疼痛改善方面优于手法组，而在停止治疗后 1 个月，联合组和手法组在疼痛改善方面效果一样。这可能是治疗期间体外冲击波在镇痛方面起了良好的作用。

6.2 Baird-Jackson 踝关节评分比较

组内比较：三组治疗 2 周后和治疗后 1 个月 Baird-Jackson 踝关节评分均有统计学差异，且评分升高，说明三组治疗陈旧性踝关节扭伤在改善踝关节综合疗效方面均有效果。

组间比较：三组治疗前 Baird-Jackson 踝关节评分无统计学差异。治疗 2 周后和治疗后 1 个月三组比较有统计学差异，且评分升高，LSD 法两两比较，*P* 值均小于 0.05，有统计学差异。说明治疗 2 周后和治疗后 1 个月三组在踝关节综合疗效方面均有疗效，且联合组>手法组>冲击波组。

分析比较表明在改善踝关节综合疗效方面无论是治疗期间还是停止治疗后，正骨理筋手法联合体外冲击波治疗陈旧性踝关节扭伤好于单独正骨理筋手法治疗，单独正骨理筋手法治疗又好于单独体外冲击波治疗。

6.3 ROM 踝关节活动度评分比较

组内比较：联合组和手法组在治疗 2 周后和治疗后 1 个月 ROM 踝关节活动度评分有统计学差异，且评分升高，冲击波组在治疗 2 周后与治疗前比较 $P=0.000<0.05$，治疗后 1 个月与治疗 2 周后比较 $P=0.129>0.05$。说明联合组和手法组治疗陈旧性踝关节扭伤

中国知网 https://www.cnki.net

在改善踝关节活动度方面均有效果，冲击波组治疗期间效果好，但是停止治疗后效果欠佳。

组间比较：三组治疗前 ROM 踝关节活动度评分无统计学差异。治疗 2 周后，三组比较有统计学差异，且评分升高，LSD 法两两比较，联合组和手法组比较：P=0.111>0.05；治疗后 1 个月，三组比较有统计学差异，且评分升高，三组组间多重对比，P 值均小于 0.05。说明治疗的 2 周期间三组在踝关节活动度方面均有疗效，但联合组和手法组在踝关节活动度方面的差异不明显；治疗后 1 个月三组在踝关节活动度方面均有疗效，且联合组>手法组>冲击波组。

分析比较表明在改善踝关节活动度方面，治疗期间联合组和手法组疗效差别不大，停止治疗后，冲击波组效果欠佳；这可能是治疗期间由于联合组和手法组均有实施正骨理筋手法，这在一定程度上纠正了踝关节错缝和肌腱的微小移位，活动度恢复较好，故差别不大，但远期疗效上联合组由于体外冲击波的疗效加持表现更好；而体外冲击波在治疗期间患者由于症状的缓解而有一定的活动度改善，停止治疗后，症状恢复没有之前的快，表现在活动度方面也改善不明显。

6.4 肿胀程度比较

组内比较：联合组和手法组在治疗 2 周后和治疗后 1 个月肿胀程度比较有统计学差异，且程度减轻，但在治疗后 1 个月与治疗 2 周后比较 P 值均大于 0.05；冲击波组内比较 P=0.267>0.05，差异无统计学意义。说明治疗期间，联合组和手法组都能改善踝关节肿胀程度，但在治疗结束后肿胀方面效果欠佳，而冲击波组在改善肿胀程度方面不明显。

组间比较：三组治疗前肿胀程度无统计学差异。治疗 2 周后和治疗后 1 个月，三组比较有统计学差异，且程度减轻，三组组间多重对比，联合组和手法组比较，P 值均大于 0.05。说明治疗期间联合组和手法组在改善肿胀程度方面均有效果，但差异不明显。

分析比较表明肿胀程度方面，在治疗期间联合组和手法组均有改善，但两者差别不大，停止治疗后改善不明显；冲击波组对肿胀程度改善不明显。这可能是由于正骨理筋手法的活血化瘀、消肿散结作用要好于体外冲击波的消肿作用，体外冲击波本身的消肿作用并不明显。

6.5 总体疗效对比

中国知网 https://www.cnki.net

三组总有效率联合组、手法组、冲击波组分别为93.10％、86.67％、75.86％，三组比较有统计学差异，组间多重对比，P 值均小于0.05。说明总体疗效方面：联合组>手法组>冲击波组。

分析比较表明患者的总体有效率同踝关节的综合疗效比较相一致。这充分说明了治疗陈旧性踝关节扭伤，正骨理筋手法联合体外冲击波要好于单纯正骨理筋手法，单纯正骨理筋手法要好于单纯体外冲击波疗法。

中国知网 https://www.cnki.net

第四部分 结 语

1 结论

①正骨理筋手法联合体外冲击波、正骨理筋手法和体外冲击波对陈旧性踝关节扭伤均有疗效。②正骨理筋手法联合体外冲击波治疗陈旧性踝关节扭伤好于单纯正骨理筋手法和体外冲击波疗法，且正骨理筋手法治疗陈旧性踝关节扭伤好于体外冲击波疗法。

2 问题及展望

2.1 问题

①本研究由于实际条件的限制，观察指标多为主观指标，缺少客观评价指标；②治疗时间只有 2 周，随访时间 1 个月，治疗和随访时间较短，缺乏远期疗效观察；③本研究纳入病例数较少，临床研究结果可能存在偏差；④目前体外冲击波治疗陈旧性踝关节扭伤的相关研究较少。

2.2 展望

正骨理筋手法和体外冲击波疗法是治疗慢性软组织损伤的常用方法，前者属于中医疗法，后者属于西医疗法，两者结合，可以取长补短，提高治疗效果，今后我们应该用中西汇通的方式思考问题。对于体外冲击波疗法治疗韧带方面的疾病，缺乏研究，这是今后研究的一个方向。

中国知网 https://www.cnki.net

参考文献

[1]尹彦,罗冬梅,刘卉,等.踝关节扭伤流行病学的研究进展[J].体育科技,2019,40(06):18-21+24.

[2]叶宜颖.基于"骨正筋柔"理论探讨摇拔戳手法治疗外侧踝关节扭伤机制的研究[D].中国中医科学院,2019.

[3]宋志靖,宋敏.宋敏教授采用正骨理筋手法治疗椎动脉型颈椎病经验[J].甘肃中医药大学学报,2020,37(03):15-17.

[4]邢更彦,张浩冲,刘水涛,等.中国骨肌疾病体外冲击波疗法指南(2019 年版)[J].中国医学前沿杂志(电子版),2019,11(04):1-10+6.

[5]踝关节扭伤中医诊疗方案,24 个专业 105 个病种中医诊疗方案. 国家中医药管理局医政司，2011:143-144.

[6]实用骨伤科手册[M].湖南:湖南科技出版社,2009:10.

[7]郑筱英.中药新药临床研究指导原则（试行）[M].北京:中国医药科技出版社,2002.342-345.

[8]国家中医药管理局.中医病症诊断疗效标准[S].南京:南京大学出版社,1991:33.

[9]苏应军,童新延,胡力.以踝关节解剖结构及生物力学特征分析慢性踝关节不稳[J].中国组织工程研究,2015,19(15):2415-2419.

[10]陈玉潇,刘姣姣,伊长松,等.本体感觉训练对运动员踝关节扭伤康复效果 Meta 分析[J].康复学报,2020,30(06):489-496.

[11]王贵忻,何锦泉.踝关节三角韧带损伤的诊断和治疗[J].中国组织工程研究,2019,23(20):3235-3241.

[12]赵勇,王钢.踝关节扭伤的生物力学与运动学研究进展[J].中国骨伤,2015,28(04):374-377.

[13]李永恒,陈兆军.踝关节陈旧性损伤的诊断和治疗进展[J].中华骨与关节外科杂志,2017,10(02):173-177.

[14]陈明亮,谷成毅,徐留海,等.踝关节外侧副韧带损伤诊断治疗研究进展[J].中国运动医学杂志,2019,38(02):152-158.

[15]郑扬康,詹杰,侯蕾,等."筋骨理论"对肌腱病的临床诊治指导探讨[J].中国运动医学杂志,2019,38(05):434-436.

[16]李西海.基于中和思想初探慢性筋骨病的防治新策略[J].中华中医药杂志,2020,35(04):1651-1653.

[17]刘迪一. 中医"筋"理论研究[D].中国中医科学院,2019.

[18]张明才,詹红生,石印玉,等."骨错缝、筋出槽"理论梳理[J].上海中医药杂志,2009,43(11):59-62.

中国知网 https://www.cnki.net

[19]张伯礼,吴勉华.中医内科学:全国中医药行业高等教育“十三五”规划教材[M].北京:中国中医药出版社,2017:363.

[20]吴国琳,李天一,范永升.全国名中医范永升教授运用虫类药治疗痹病经验探析[J].中国中药杂志,2019,44(04):845-848.

[21]窦志芳.《医林改错》注释及临床应用[M].太原:山西科技出版社,2006:124.

[22]顾骐,李金学,朱立国,等.中医正骨推拿手法分类的现状与分析[J].中医正骨,2011,23(08):74-77.

[23]徐志为,刘建航.推拿手法作用机制的研究进展[J].湖南中医杂志,2014,30(06):185-187.

[24]殷京,孙树椿,赵宝力,等.清宫正骨流派传承与其特色理筋手法运用探析[J].中华中医药杂志,2021,36(01):267-271.

[25]陈兆军,孙树椿,等.清宫外踝理筋手法治疗陈旧性踝关节损伤的临床研究[J].中国中医骨伤科杂志,2017,25(09):30-34.

[26]梁国富.理筋正骨手法联合针刺治疗陈旧性踝关节扭伤的疗效观察[J].临床医药文献电子杂志,2019,6(58):27-28.

[27]翟东旺.外踝理筋手法联合中药熏洗治疗陈旧性踝关节扭伤 42 例临床观察[J].中国民族民间医药,2019,28(08):94-95.

[28]俞益火,谢嫚花,周文军,等.中医理筋正骨手法治疗慢性踝关节损伤的临床研究[J].中医正骨,2019,31(03):20-27.

[29]Ogden JA,Tóth-Kischkat A,Schultheiss R.Principles of shock wave therapy[J].Clin Orthop Relat Res,2001(387):8-17.

[30]Speed CA.Extracorporeal shock-wave therapy in the management of chronic soft-tissue conditions[J].J Bone Joint Surg Br,2004,86(2):165-171.

[31]Chen YJ,Kuo YR,Yang KD,et al.Shock wave application enhances pertussis toxin protein-sensitive bone formation of segmental femoral defect in rats[J].J Bone Miner Res,2003,18(12):2169-2179.

[32]Notarnicola A,Tamma R,Moretti L,et al.Effects of radial shock waves therapy on osteoblasts activities[J].Musculoskelet Surg,2012,96(3):183-189.

[33]Zhao Z,Ji H,Jing R,et al.Extracorporeal shock-wave therapy reduces progression of knee osteoarthritis in rabbits by reducing nitric oxide level and chondrocyte apoptosis[J].Arch Orthop Trauma Surg,2012,132(11):1547-1553.

中国知网 https://www.cnki.net

[34]Zhai L,Ma XL,Jiang C,et al.Human autologous mesenchymal stem cells with extracorporeal shock wave therapy for nonunion of long bones[J].Indian J Orthop,2016,50(5):543-550.

[35]Ohtori S,Inoue G,Mannoji C,et al.Shock wave application to rat skin induces degeneration and reinnervation of sensory nerve fibres[J].Neurosci Lett,2001,315(1-2):57-60.

[36]Ochiai N,Ohtori S,Sasho T,et al.Extracorporeal shock wave therapy improves motor dysfunction and pain originating from knee osteoarthritis in rats[J].Osteoarthritis Cartilage,2007,15(9):1093-1096.

[37]Takahashi N,Ohtori S,Saisu T,et al.Second application of low-energy shock waves has a cumulative effect on free nerve endings[J].Clin Orthop Relat Res,2006,443:315-319.

[38]Foldager CB,Kearney C,Spector M.Clinical application of extracorporeal shock wave therapy in orthopedics:focused versus unfocused shock waves[J].Ultrasound Med Biol,2012,38(10):1673-1680.

[39]Mariotto S,de Prati AC,Cavalieri E,et al.Extracorporeal shock wave therapy in inflammatory diseases:molecular mechanism that triggers anti-inflammatory action[J].Curr Med Chem,2009,16(19):2366-2372.

[40]Mariotto S,Cavalieri E,Amelio E,et al.Extracorporeal shock waves:from lithotripsy to anti-inflammatory action by NOproduction[J].Nitric Oxide,2005,12(2):89-96.

[41]Holfeld J,TepeköylüC,Kozaryn R,et al.Shockwave therapy differentially stimulates endothelial cells:implications on the control of inflammation via toll-Like receptor 3[J].Inflam mation,2014,37(1):65-70.

[42]Speed CA.Extracorporeal shock-wave therapy in the management of chronic soft-tissue conditions[J].J Bone Joint Surg Br,2004,86(2):165-171.

[43]Ma HZ,Zeng BF,Li XL.Upregulation of VEGF in subcho ndral bone of necrotic femoral heads in rabbits with use of extra corporeal shock waves[J].Calcif Tissue Int,2007,81(2):124-131.

[44]Wang CJ,Wang FS,Yang KD,et al.Shock wave therapy induces neovascularization at the tendon-bone junction.A study in rabbits[J].J Orthop Res,2003,21(6):984-989.

[45]唐贻贤,肖文武,张晓松,等.超声定位下中能量聚焦式体外冲击波治疗钙化性冈上肌腱炎的临床疗效[J].中华物理医学与康复杂志,2020,42(11):1023-1025.

[46]俞诗威,邢丹谋,冯伟,等.超声介导注射联合体外冲击波治疗冈上肌钙化性肌腱炎的临床疗效[J].中华手外科杂志,2020,36(05):358-362.

中国知网 https://www.cnki.net

[47]冯刚,许红霞,卯姣娜.痛点注射疗法和冲击波疗法对肌腱炎的治疗效果比较[J].四川解剖学杂志,2020,28(01):101-102.

[48]秦建忠,董启榕,范志英,等.体外冲击波治疗运动员踝部韧带损伤的前瞻性研究[J].中国康复医学杂志,2015,30(04):355-358.

[49]饶毅,肖京,曹昺焱,等.基于经筋理论指导下的冲击波治疗腰背部棘上韧带炎的疗效观察[J].中国中西医结合杂志,2019,39(06):753-755.

[50]Dizon JN,Gonzalez-Suarez C,Zamora MT,et al.Effectiveness of extracorporeal shock wave therapy in chronic plantar fasciitis:a meta-analysis[J].Am J Phys Med Rehabil,2013,92:606-620.

[51]刘洪柏,张鸣生,区丽明,等.体外冲击波对大鼠膝骨关节炎白细胞介素-1β 及肿瘤坏死因子-α 表达的影响[J].中国康复医学杂志,2014,29(03):208-211+217.

[52]郭江,陈本华.体外冲击波治疗IIIB 型慢性前列腺炎患者的疗效及机制[J].实用医学杂志,2017,33(17):2900-2903.

[53]汪喆,彭昊,刘世清,等.体外冲击波治疗兔缺血性股骨头坏死过程中血管内皮生长因子的表达[J].中华实验外科杂志,2009(05):639-640.

[54]赵子星,李宏宇,席立成,等.体外冲击波疗法联合仙桃草口服用于兔桡骨骨不连临床效果观察[J].山东医药,2016,56(36):31-33.

[55]王五洲,邢更彦,叶启彬,等.冲击波干预成人骨髓间充质干细胞后 IGF-I和 TGF-β1 的表达及意义[J].中国矫形外科杂志,2006(13):1009-1012.

[56]马玲,贾子善,肖红雨,等.体外冲击波干预治疗对大鼠肌腱损伤修复的观察[J].解放军医学院学报,2015,36(09):932-934+962.

[57]Vetrano M,d'Alessandro F,Torrisi MR,et al.Extracorporeal shock wave therapy promotes cell proliferation and collagen synthesis of primary cultured human tenocytes[J].Knee Surg Sports Traumatol Arthrosc.2011 Dec;19(12):2159-68.

[58]刘洪柏,张鸣生,区丽明.体外冲击波对大鼠膝骨性关节炎中基质金属蛋白酶-1、3、13 表达的影响[J].中国康复医学杂志,2014,29(11):1016-1019+1030.

[59]金先跃,李宏宇,田文,等.独活寄生汤联合体外冲击波对膝骨关节炎患者 IL-1β 和 SOD 及 MMP-3 表达的影响[J].中国临床新医学,2017,10(09):838-842.

中国知网 https://www.cnki.net

[60]刘辉,刘波,张鑫,等.传统关节粘连松解术联合冲击波和肌肉能量技术治疗踝关节僵硬[J].中医正骨,2019,31(12):59-62.

[61]李祥雨,马喜凤,张伦广,等.姜劲挺应用冲击波经络击打法治疗踝关节外侧副韧带损伤探讨[J].新中医,2018,50(03):180-183.

[62]周家彬.冲击波治疗散打运动员踝关节陈旧性扭伤的应用[J].双足与保健,2019,28(02):9-10.

[63]乔雅馨,席占国,张浩良,等. 踝关节外侧副韧带损伤的超声诊断结合冲击波治疗的可行性分析[C].中国超声医学工程学会.中国超声医学工程学会第七届全国肌肉骨骼超声医学学术会议论文汇编.中国超声医学工程学会:中国超声医学工程学会,2019:11

[64]刘怡梦. 体外冲击波治疗对陈旧性踝扭伤的疗效观察[C]. 中国中西医结合学会骨伤科专业委员会.2019 楚天骨科高峰论坛暨第二十六届中国中西医结合骨伤科学术年会论文集.中国中西医结合学会骨伤科专业委员会:中国中西医结合学会,2019:601.

[65]都本才,杨晓明,管清丽,等.士兵军事训练致踝前外侧软组织撞击伤综合征的体外冲击波治疗效果[J].实用医药杂志,2017,34(08):678-680.

[66]王荷琴,李哲,郭钢花.蜡疗、低能量冲击波治疗急性踝关节扭伤[J].世界最新医学信息文摘,2016,16(32):158+161.

[67]王筱锋,朱倩.发散式冲击波治疗陈旧性踝关节扭伤 38 例临床观察[J].中国民间疗法,2015,23(12):72-73.

中国知网 https://www.cnki.net

文献综述

中西医治疗陈旧性踝关节扭伤研究概况

摘要：陈旧性踝关节扭伤是临床常见疾病，由踝关节扭伤后失治、误治而来，踝关节出现隐痛、酸困、活动受限、踝关节不稳等症状，影响日常工作、生活。通过阅读文献，本文就陈旧性踝关节扭伤的中西医治疗现状进行综述。

关键词：中西医结合治疗；陈旧性踝关节扭伤；研究概况

踝关节扭伤是一种临床很常见的肌肉骨骼损伤疾病，又称踝关节软组织损伤、踝关节扭挫伤、跂骨离位伤筋、踝关节韧带损伤等，可发生于任何年龄，女性多见，多因行走于不平路面、下楼梯或者跑跳、穿高跟鞋、体育训练时动作不协调、行走不稳导致踝跖屈位时，突然足强力内翻或外翻，引起外侧副韧带或者内侧副韧带牵拉伤，由于踝关节的解剖特点，临床以足内翻伴外侧副韧带损伤多见，占全身关节扭伤的 80%以上，急性期以踝关节明显肿胀疼痛、跛行、功能障碍、皮肤青紫为特征，排除严重急性踝扭伤需要手术外，约 36%-85%的患者通过初始保守治疗，如早期活动指导、理疗、绷带、支具等，可完全恢复[1]；如未能得到及时治疗或治疗方法不得当，日久愈合欠佳，则引起患处纤维组织增生、局部组织粘粘、软骨损伤、关节面毛糙、筋伤筋结等改变，病程超过 3 周者，则称为陈旧性踝关节扭伤，以踝关节局部隐痛、酸楚、麻木、组织发硬、活动受限、踝关节不稳为特征，上述症状尤在天阴下雨、寒冷潮湿环境中明显加重，关节损伤日久不愈则易并发滑膜炎、软骨损伤、剥脱性骨软骨炎、外侧关节内撞击综合征、胫骨前方骨赘、关节内游离体等，危害极大，影响日常工作、生活。故在此将近年来中西医治疗陈旧性踝关节扭伤的疗法总结如下，以期为临床优化治疗方案提供依据。

1 中医药治疗

1.1 中药治疗

陈旧性踝关节扭伤因外伤致瘀血内阻，气血运行不畅，加之皮肤肌肉腠理不密，风寒湿邪乘虚而入，内外邪合阻肌肉、经络，气滞血瘀，“不通则痛，不荣则痛”，气血失养则麻木、隐痛，治当散寒逐湿，活血化瘀，理气和血，因病灶局限，故临床多采用中药外敷、外洗、熏蒸的方法局部治疗。中药熏蒸治疗将“热效”与“药效”相结合，利用热能以疏通腠理、舒张血管，疏通血脉，促进血液循环，改善皮肤的吸收功能，进一步使复方中药渗透入里发挥舒筋活络、止痛、活血化瘀的疗效，同时热效应可助阳散寒、增

中国知网 https://www.cnki.net

强活血化瘀、疏通筋脉的作用，使疗效更加突出持久。实践证明，中药外用法作用直接、疗效明显，无明显毒副作用。郝金林等[2]观察中药熏蒸联合针刺治疗陈旧性踝关节扭伤，处方为：延胡索、制川乌、制草乌各 20 g 祛风除湿，温经止痛，大黄 15 g 荡涤瘀血，川楝子 20g、鸡血藤、芍药、土鳖虫、伸筋草各 30 g 活血舒筋通络，结果显示联合治疗方案能显著降低患者 VAS 评分，提高 AOFAS 评分及关节活动度评分，较单用针刺疗法更能减轻患者疼痛感，改善关节活动度，且治疗安全性好。王冠军[3]观察中药熏蒸联合小针刀治疗陈旧性踝关节扭伤，所用中药处方为：防风 15g、荆芥 10 g 祛风解痉，散寒胜湿止痛，刘寄奴、大黄各 15 g 疗伤止血、破血通经，宽筋藤、忍冬藤、钩藤、王不留行各 30 g 舒筋通络，结果显示相较单纯小针刀组，观察组疼痛评分降低，Kofoed 评分升高，表明联合治疗方法效果更佳。

1.2 针灸治疗

针灸作为一种古老而又生机勃勃的治疗方法，以针为代表，包括了灸、拔罐、按摩等体表刺激法，具有疏通经络、调和阴阳、扶正祛邪的作用。随着针灸在世界范围内受瞩目，其与现代医学的联系亦越来越紧密，其疗效机理更为清楚，治法更加多样化，适应范围更广阔。在普通针刺、温针、火针、灸法、刺络拔罐法等传统治法的基础上，现代医学发展了电针、穴位注射、激光穴位疗法、低频磁疗法、针刀等新的疗法。研究表明，针刺具有抗炎[4]、麻醉镇痛[5]、调节代谢[6]、机体免疫及微循环[7]等多种疗效，由此看出，现代机理研究与传统治疗作用是一致的。

陈旧性踝关节扭伤归属中医“伤筋”、“痹症”范畴，因外伤致筋脉受损，淤血内滞，久病入络，经络气血不通，风寒湿邪乘隙而入，进一步加重筋脉失养，内外邪凝结，引起局部麻木、隐痛、软组织结节等症状；瘀血作为病理因素及致病因素，在整个病程中起主要作用，故可运用针灸“补虚泻实、陈菀则除之”的治则以扶正祛邪、活血化瘀、通络止痛。陈壮娜等研究表明[8]，电温针丘墟、阳陵泉穴治疗陈旧性踝关节扭伤，与普通针刺丘墟、申脉、解溪和阿是穴、阳陵泉相对比，电温针对疼痛及关节功能的临床疗效更佳；周彬等[9]研究亦表明电针联合火针治疗对踝关节功能的改善优于只运用电针治疗；这与李军[10]的研究结果一致，上述研究均表明，联合使用现代针刺方法将热效、针效及电生理刺激作用相结合，从风寒瘀虚、神经肌肉刺激等多方面治疗陈旧性踝关节扭伤的效果更佳，而穴位多以踝三针：解溪、昆仑、太溪为主。

中国知网 https://www.cnki.net

另外，针灸、针刀可运用刺、割、剥等手法松解组织、解除粘连、改善血运，使关节恢复活动功能[11]。对于陈旧性踝关节扭伤，近年来也有个别新型疗法的报道，如超激光疼痛治疗仪局部照射解溪、昆仑、太溪等穴位[12]；踝关节周围痛点及静脉曲张处刺络放血法以达到祛瘀生新，改善局部微循环，促进损伤组织修复的作用[13]；张茂亮[14]观察手法治疗配合穴位注射治疗 23 例陈旧性踝关节扭伤患者，手法理筋以去病疾，强筋以防反复，穴位注射以活血化瘀，VitB1 营养神经，诸多方法合用以增加疗效，防止复发，总有效率可达 94%；以上疗法虽具创新性，但近 10 年所能查阅到的文献甚少，研究中观察样本量过少，存在证据不足问题，其疗法仍需临床反复验证。

1.3 手法治疗

伤科手法治疗历史悠久，《医宗金鉴》曰：“法之所施，使患者不知其苦，方称为手法也”。推拿即按摩，通过推拿者的手或者其他辅助器材，运用按、摩、揉、抖、推、拿等手法技巧在人体一定穴位或部位上防治疾病的一种方法，在中医学理论的指导下，其具有活血化瘀、消肿止痛，剥离粘连、滑利关节、平衡阴阳、调整人体器官功能的作用。

中医认为，“骨正筋柔，筋骨合和”，筋是柔顺而有弹性的，它维系着骨与骨间的稳固联结，并辅助完成各种关节动作，它是筋骨系统保持正常生理状态极为重要的部分。推拿则是在筋伤处顺着肌纤维用推、按、揉、拨等手法以理顺肌纤维，使筋结松解，瘀血散开，气血畅通，关节滑利，达到治疗目的。除此外，陈旧性踝关节扭伤还存在细微的骨错缝问题，而正骨理筋手法可通过牵、理、摇、推、揉等法整复筋骨关节，使之恢复正常生理功能。陈旧性踝关节扭伤中医亦可称之为痹症，“风寒湿三邪合而为痹”，风邪易夹它邪而致病，损伤后期，久病正气虚，卫气不固，机体易受风寒湿外邪侵袭，寒邪凝滞，湿性泥滞，凝聚于患处，气血不通、失荣则肢体酸楚，麻木沉重，手法治疗可促进局部血液循环，温热肌表以驱散风寒，温通经络，促进症状消散。

实践证明，手法整复治疗陈旧性踝关节扭伤疗效确切。奚小冰[15]观察以魏氏手法+特色中药熏洗治疗患足踝 6 周，外用中药处方：伸筋草、川牛膝、老鹳草、海桐皮、桑寄生、川羌活、川当归、泽兰叶、乳香、没药各 12g、川木瓜 9 g、山慈菇 15 g；结果显示，6 周时 AOFAS 踝-后足评分较扶他林软膏联合功能锻炼的对照组无差异，而 6 月后两者评分方具有明显差异。舒文韬等[16]观察杜氏拔伸摇踝手法配合香独活血散外敷治疗患足踝 2 周，其 VAS 评分较对照组（TDP 照射配合香独活血散外敷）明显下降，Biard-

中国知网 https://www.cnki.net

Jackson 踝关节评分明显升高，总有效率达 95%，明显高于对照组（78%）。陈兆军等[17]经肌骨超声检查证实外踝理筋手法治疗后患侧损伤的韧带厚度回声变薄，18 月随访时患侧韧带厚度与健侧基本相同，说明理筋手法不仅能使患者疼痛、关节活动不利等症状减轻或消失，且能通过改善踝关节周围韧带的厚度、宽度、张力弹性起治疗作用。陈兆军等[18]研究表明清宫外踝理筋手法在缓解疼痛、改善踝关节功能方面疗效显著，且优于功能锻炼组，但二者均在距骨倾斜度改善方面与治疗前相比虽然有改善，但无统计学意义，说明其在改变踝关节解剖结构方面效果不确切，手法针对功能性的陈旧性踝关节不稳效果更佳。

综上所述，正骨理筋手法治疗是中医治疗慢性伤科病的一大特色，有确切疗效，亦可避免手术之痛苦，而综合近年来的研究表明，手法联合针刺[19]、中药熏洗[20, 21]以及活血化瘀药物等均较单纯针灸、中药以及磁疗联合针刺等方法的治疗效果更好；手法常用按揉类、活动关节类、弹拨拔伸类、摇戳类等，按、揉以行气活血散结，去瘀生新，气血调和则可发挥濡养作用；弹拨拔伸类手法以松解粘连，扩大关节间隙，恢复正常解剖结构，起到整复细微骨错缝的作用；中药多以活血化瘀、通络止痛类药物外敷、外洗、熏蒸或以膏药摩擦治疗患部；针灸以踝周穴、阿是穴为主。中医治疗陈旧性踝关节扭伤临床疗效佳，但观察指标多为患者主观感受，研究存在客观证据不足问题，对其治疗机理仍需做进一步深入研究。

2 西医治疗

2.1 药物治疗

药物治疗以对症治疗为主，常用药物有非甾体类抗炎药、活血化瘀类中成药以及糖皮质激素、利多卡因、维生素 B1 局部封闭治疗[22]，但口服药物短期具有消炎镇痛、活血化瘀作用，长期使用胃肠刺激大，有肝肾功损害等较多副作用，有消化系统基础疾病者，更有消化道出血风险，故该类药物不可作为主要治疗方案；局部封闭治疗使用糖皮质激素抗炎、减少炎性渗出，利多卡因局部麻醉镇痛，维生素 B1 营养神经，三者合用患部注射具有消肿止痛、营养受损神经作用，部分踝关节扭伤伴神经受损的患者经封闭治疗后疼痛可消失，该类方法临床多用，但文献研究甚少，临床发现局部封闭治疗后易复发，反复使用有耐药、增加软骨脆性、晶体性关节炎风险。

2.2 非手术物理治疗

中国知网 https://www.cnki.net

2.2.1 功能锻炼

功能锻炼主要是通过指导患者进行患足踝周肌肉力量以及平衡能力、本体感觉等对症的运动训练以达到改善症状，治疗并预防疾病复发的一种康复治疗手段。研究发现[23]通过对患足背曲、跖曲、内旋、外旋功能锻炼结合带状训练、重量训练、改善本体感觉的训练，康复运动 12 周后，双侧跖曲强度、同侧肌力比、等长内翻强度和等长外翻强度在统计学上显著改善，说明康复功能锻炼能够有效增强踝关节肌力，改善下肢平衡能力及踝关节本体感觉，进而减少疼痛，促进关节功能恢复。庞红梅等[24]给予踝关节反复扭伤患者增加踝关节活动度、负重训练、踝周肌肉韧带力量和协调性训练、踝关节本体感觉训练、抗阻力背屈、跖屈、内翻训练以及平衡稳定性训练，发现经以上渐进性训练 4 个阶段（连续 6 天为 1 阶段，6-10min/d），与未训练者相比观察组 AOFAS 评分明显升高，且疗效随时间而累积增加，说明针对踝关节肌肉、韧带力量、协调性的康复训练对防治反复踝关节扭伤有显著疗效，且该方法是一个循序渐进的过程，不可一蹴而就。Lee Dong-Rour[25]研究得出了类似结果，足部肌肉锻炼能够有效改善慢性踝关节不稳患者足部肌肉功能及平衡能力。

2.2.2 器具辅助疗法

临床常用器具有肌内效贴和支具、矫形鞋。裴子文等[26]研究发现在常规功能锻炼基础上使用肌内效贴贴扎治疗能即刻增强慢性踝关节不稳患者踝关节稳定性，提升平衡能力，改善自觉症状，进一步改善关节功能障碍。张弓皓等[27]大数据分析发现踝关节支具可有效提升患者的平衡能力、关节位置觉，较功能锻炼更能有效地降低扭伤患者再扭伤的发生率，而半硬材质支具，如马镫样结构和束带样结构支具较软材质更有优势。对于踝关节骨折手术患者，支具固定配合功能锻炼较石膏固定功能锻炼者更能安全有效促进踝关节术后早期功能的康复[28]；同样，对于早期踝关节侧副韧带损伤，DARCO 踝关节 U 型支具治疗在总有效率、治愈率和满意度方面均优于 U 型石膏托固定者[29]。

2.2.3 体外冲击波

近年来随着对体外冲击波疗法的深入研究，ESWT 已成为肌肉骨骼系统疾病的保守替代疗法，如股骨外上髁炎、肌腱炎、肩周炎、足底筋膜炎、骨不连或愈合延迟、股骨头坏死、膝骨关节炎等，具有非侵入性、安全、有效、副作用小的优势。

中国知网 https://www.cnki.net

体外冲击波（ESW）[30]是一种机械能量波，冲击波的能量能在小于 10 ns 的极短时间内释放高达 100 MPa 的峰值压力。它针对作用组织产生机械效应、空化效应、热效应，从而使组织内压强、温度、密度等物理性质发生显著的、跳跃式的改变，产生治疗作用。具体有以下几点：①当冲击波在不同的介质（肌腱、韧带、脂肪、骨骼）中传播时可以生产应力作用，该应力方向不同，有挤压组织的压应力和拉伸组织的拉应力，它们的共同作用下可以引起组织间的松解，毛细血管循环的加快及增加组织细胞的摄氧量和细胞弹性形变，最终达到分离粘连，伸展肌肉挛缩的目的。②声学效应：冲击波在不同密度的组织间传播速度不同，声阻抗的变化导致组织表面及内部产生应力作用，可促进细胞增殖分化。③热效应：冲击波在传播中会产生热能，热能具有加强组织代谢，增加局部供血供氧，改善组织营养，降低肌张力、缓解痉挛、镇痛作用。

杨金宇等[31]使用体外冲击波治疗距骨骨软骨损伤，治疗 3 月后与非甾体类抗炎药相比，观察组 MRI 显示损伤面积明显缩小，骨髓水肿减轻，说明 ESWT 可通过促进毛细血管再生，改善微循环，促进局部致炎因子代谢，减轻疼痛。国外研究亦表明[32]，ESWT 治疗可增加腱-骨连接处的新生血管数量，增加 I 型和 III 型胶原、转化生长因子（TGF-β）和胰岛素生长因子（ILGF-1），促进韧带修复。苏宏图等[33]观察 ESWT（1.8-2.5 bar，频率 8-10 Hz，冲击次数 2500-3000 次，1 周治疗 1 次，4 次 1 疗程）联合下肢洗伤方治疗陈旧性踝部软组织损伤，治疗 2 周后比较各组 VAS 及 AOFAS 评分，结果显示联合组均优于单一治疗组，而冲击波组优于中药熏洗组，说明该方法临床疗效确切，能够有效改善症状，促进组织功能的恢复。周家彬研究[34]也证实 ESWT 治疗陈旧性踝关节扭伤可取得理想的效果，与传统药物及手术治疗相比有很大的优越性。研究表明[35]在前交叉韧带（ACL）重建中，ESWT（0.15 mJ/mm2，1000 次脉冲，4 Hz）能增强 ACL 残余细胞的活力，其增殖与迁移率、胶原基因、TGF-β、血管内皮生长因子（VEGF）的表达均显著增加，并且可增强周围骨髓基质细胞（BMSCs）的增殖与迁移，提高 BMSCs 成腱分化的能力，促进韧带移植成功。

以上研究表明 ESWT 可通过减轻疼痛、改善患部微循环，修复损伤韧带起治疗作用，ESWT 治疗陈旧性踝关节扭伤在临床实践中具有确切疗效，但其理论机制仍需进一步大数据研究。

2.3 手术治疗

中国知网 https://www.cnki.net

2.3.1 对韧带起止点的原位解剖缝合手术

该方法用于外侧副韧带尚完整或残端较完整的患者，开放术式以 Broström 术式为代表，手术要点是将断裂的韧带缩紧、重叠后缝合，但开放手术血管神经损伤、术后韧带瘢痕化、皮缘愈合不佳、感染等缺点，随着近年来关节腔镜的发展，目前采用全关节镜下锚钉修复术[36]，该术式仅选择辅助前外侧和内侧中线作为手术入路，切口小，全程能直观的尽最大可能的规避以上风险，最大程度保护关节囊结构，维持皮肤感觉及踝关节原有机械感受器作用，进而改善踝关节功能。大量文献研究[37-39]表明关节腔镜下锚钉修复术创伤小、疼痛轻、术后恢复快，有很好的疗效。

2.3.2 韧带重建手术

韧带重建用于修补术失败、韧带严重缺损、肥胖、重体力劳动和高强度运动的患者，分为非解剖重建、解剖重建。

非解剖重建手术指使用腓骨肌肌腱等重建外踝韧带。该类手术相对简单，恢复快，近期效果好，但临床发现由于改变生物力学的原因，远期会出现不同程度的踝关节、距下关节功能受限，加速关节退行性改变，新发关节不稳等并发症[40,41]，因此目前临床多使用解剖原位重建。

解剖原位重建手术指用自体、异体或人工肌腱原位固定重建外踝韧带，优点是无需牺牲其他有用的组织，通过移植韧带以恢复解剖结构，进一步维持踝关节的稳定，恢复其功能；相比非解剖重建该方法疗效确切，创伤小、并发症少[42，43]，且关节腔镜技术的发展更是减少了手术并发症[44]；不同移植物材料的并发症亦不同，自体肌腱廉价、骨腱愈合快、本体感觉恢复快，但需另取切口和组织、患者不易接受。异体肌腱手术创伤小，远期疗效好，价格高，存在感染、排异、本体感觉重塑缓慢等缺点。目前手术方式的选择无统一定论，临床可根据患者自身情况选择最佳术式。

综上所述，慢性踝关节扭伤的治疗，首选中医正骨理筋手法，联合针灸、活血化瘀药物患部外用、体外冲击波等疗法效果更佳，这与西医治疗理念是一致的，因此对于缺少中医治疗条件者，建议长时间的严格康复训练[45]；对病程 3～6 个月以上、经保守治疗效果欠佳或对关节功能要求高的机械性踝关节不稳者，建议手术治疗，以解剖原位修复或重建为主，术后康复训练对功能的恢复尤为重要。

中国知网 https://www.cnki.net

参考文献

[1]van Ochten John M,van Middelkoop Marienke,Meuffels Duncan,et al.Chronic complaints after ankle sprains: a systematic review on effectiveness of treatments.[J].The Journal of orthopaedic and sports physical therapy,2014,44(11).

[2]郝金林,徐芳华.中药熏蒸联合针刺治疗陈旧性踝关节扭伤疗效观察[J].新中医,2018,50(02):119-122.

[3]王冠军.中药熏蒸配合小针刀治疗陈旧性踝关节扭伤疗效观察[J].新中医,2015,47(05):265-266.

[4]李媛,吴凡,沈雪勇,劳力行.膝关节炎模型及针灸对其抗炎镇痛机制的研究进展[J].中国中西医结合杂志,2019,39(12):1523-1529.

[5]韩济生.针麻镇痛研究[J].针刺研究,2016,41(05):377-387.

[6]虞莉青,曹莲瑛,谢菁,等.电针治疗胰岛素抵抗型多囊卵巢综合征疗效与作用机制研究[J].中国针灸,2020,40(04):379-383.

[7]朱凤亚,江泰君,汤莉洁,等.针灸治疗慢性疲劳综合征肠道菌群-免疫功能的作用机制[J].中国中医基础医学杂志,2020,26(09):1354-1358.

[8]陈壮娜,熊峻,黄石龙.不同针灸疗法治疗陈旧性踝关节扭伤的疗效观察[J].内蒙古中医药,2020,39(02):91-92.

[9]周彬,金瑛,曹莉.电针结合火针治疗陈旧性踝关节内翻扭伤的临床疗效观察[J].时珍国医国药,2018,29(01):121-122.

[10]李军.不同针灸疗法治疗陈旧性踝关节扭伤临床效果研究[J].足踝外科电子杂志,2019,6(04):39-42.

[11]付解辉,陈长兴,李雁婷,等.小针刀联合理筋法对陈旧性踝关节扭伤患者踝关节功能的影响[J].中外医学研究,2020,18(12):53-54.

[12]金志超,郑晓辉,刘芳芳,等.“踝三针”联合超激光治疗陈旧性踝关节扭伤的疗效观察[J].中医药导报,2018,24(18):79-80+85.

[13]赵天杰,王武超,杨琼.刺血法治疗陈旧性踝扭伤遗留踝关节行走痛的疗效[J].中国中医药现代远程教育,2016,14(18):125-127.

[14]张茂亮.手法配合穴位注射治疗陈旧性踝关节扭伤疗效观察[J].中医临床研究,2013,5(18):49.

[15]薛彬,万世元,李飞跃,等.魏氏伤科法治疗陈旧性踝关节扭伤的临床分析[J].中成药,2014,36(08):1612-1615.

中国知网 https://www.cnki.net

[16]舒文韬,欧阳松,等.杜氏拔伸摇踝手法配合香独活血散外敷治疗陈旧性踝关节扭伤疗效观察[J].四川中医,2020,38(11):158-160.

[17]陈兆军,常青,吴俊德,等.肌骨超声观察外踝理筋手法治疗陈旧性踝关节扭伤 39 例[J].中国中医骨伤科杂志,2018,26(07):42-46.

[18]陈兆军,孙树椿,等.清宫外踝理筋手法治疗陈旧性踝关节损伤的临床研究[J].中国中医骨伤科杂志,2017,25(09):30-34.

[19]梁国富.理筋正骨手法联合针刺治疗陈旧性踝关节扭伤的疗效观察[J].临床医药文献电子杂志,2019,6(58):27-28.

[20]翟东旺.外踝理筋手法联合中药熏洗治疗陈旧性踝关节扭伤 42 例临床观察[J].中国民族民间医药,2019,28(08):94-95.

[21]薛保才.正骨手法联合熏洗方治疗陈旧性踝关节扭伤 32 例[J].光明中医,2017,32(22):3262-3263.

[22]苏展,李焱,刘竹.踝关节扭伤合并神经损伤的诊治体会[J].黑龙江医学,2009,33(06):446-447.

[23]Kim Kewwan,Jeon Kyoungkyu.Development of an efficient rehabilitation exercise program for functional recovery in chronic ankle instability.[J].Journal of physical therapy science,2016,28(5).

[24]庞红梅,韩庆林.早期康复训练用于足踝韧带扭伤患者中的效果[J].南昌大学学报(医学版),2016,56(04):52-55.

[25]Lee Dong-Rour,Choi Young-Eun.Effects of a 6-week intrinsic foot muscle exercise program on the functions of intrinsic foot muscle and dynamic balance in patients with chronic ankle instability.[J].Journal of exercise rehabilitation,2019,15(5).

[26]裴子文,汪冕,言功立,等.肌内效贴治疗慢性踝关节不稳的即刻及短期疗效观察[J].中国康复,2020,35(09):463-466.

[27]张弓皓,曹圣轩,石家齐,等.踝关节支具对功能性踝关节不稳的作用[J].国际骨科学杂志,2019,40(01):29-33.

[28]刘艳,谭艳庆,谭彩霞,等.支具辅助旋后-外旋型IV度踝关节骨折术后早期功能康复的疗效观察[J].现代医院,2018,18(11):1661-1664.

[29]豆勇刚,黎健伟,余斌.U 型支具治疗早期踝关节侧副韧带损伤的疗效分析[J].中华关节外科杂志(电子版),2012,6(04):568-573.

中国知网 https://www.cnki.net

[30]邢更彦,张浩冲,刘水涛,等.中国骨肌疾病体外冲击波疗法指南(2019 年版)[J].中国医学前沿杂志(电子版),2019,11(04):1-10+6.

[31]杨金宇,王雪菲,李少迪,等.基于步态分析的距骨骨软骨损伤的冲击波治疗[J].足踝外科电子杂志,2020,7(01):39-42.

[32]Notarnicola A,Moretti B.The biological effects of extracorporeal shock wave therapy (eswt) on tendon tissue[J].Muscles Ligaments Tendons J,2012,2(1):33-37.

[33]苏宏图,林坤山.体外冲击波结合下肢洗伤方治疗踝部陈旧性软组织损伤[J].中国骨与关节损伤杂志,2017,32(12):1327-1329.

[34]周家彬.冲击波治疗散打运动员踝关节陈旧性扭伤的应用[J].双足与保健,2019,28(02):9-10.

[35]Lu Cheng Chang,Chou Shih Hsiang,Shen Po Chih,et al.Extracorporeal shock wave promotes activation of anterior cruciate ligament remnant cells and their paracrine regulation of bone marrow stromal cells' proliferation, migration, collagen synthesis, and differentiation.[J].Bone&joint research,2020,9(8).

[36]李国胜.全关节镜下锚钉修复对慢性踝关节不稳距腓前韧带损伤术后患者踝关节功能的影响[J].中国疗养医学,2021,30(03):309-311.

[37]王国强,屈建国,渠海波,等.关节镜下 Brostr(o)m-Gould 术治疗慢性踝关节外侧不稳[J].中国内镜杂志,2021,27(01):28-33.

[38]徐柯烽,林平,涂迎春,等.踝关节镜下修复距腓前韧带治疗慢性踝关节不稳[J].中华关节外科杂志(电子版),2020,14(05):636-639.

[39]蒋宏魁,樊力,冯亮,等.全镜下无结锚钉技术修复距腓前韧带治疗慢性踝关节外侧不稳定的临床疗效[J].临床和实验医学杂志,2020,19(21):2332-2335.

[40]李永恒,陈兆军.踝关节陈旧性损伤的诊断和治疗进展[J].中华骨与关节外科杂志,2017,10(02):173-177.

[41]Noailles Thibaut,Lopes Ronny,Padiolleau Giovanni,et al.Non-anatomical or direct anatomical repair of chronic lateral instability of the ankle: A systematic review of the literature after at least 10 years of follow-up.[J].Foot and ankle surgery:official journal of the European Society of Foot and Ankle Surgeons,2018,24(2).

中国知网 https://www.cnki.net

[42]尚林,王翔宇,王爱国,等.距腓前韧带重建联合踝关节镜术治疗慢性踝关节外侧不稳[J].中国矫形外科杂志,2019,27(08):744-748.

[43]刘建永.慢性踝关节外侧不稳定距腓前韧带和跟腓韧带解剖重建[J].中国运动医学杂志,2016,35(02):126-131.

[44]张跃钟,胡跃林,江东.关节镜在慢性踝关节不稳治疗中的应用[J].中国微创外科杂志,2016,16(09):845-847.

[45]外踝韧带损伤的中西医结合治疗专家共识[J].中华骨科杂志,2019(11):653-659.

中国知网 https://www.cnki.net

附 录

附录 1

知情同意书

本人已仔细阅读“正骨理筋手法联合体外冲击波治疗陈旧性踝关节扭伤的临床疗效观察”，已了解这是一项安全性及有效性评价的临床研究，临床试验研究者已就此研究的特点和可能存在的不良反应向我做了详细解释，并对有关问题给予了解答。我在充分了解受试者须知的全部内容以及参加受试者带来的利弊后，志愿参加本实验，我已充分理解：

1、此项研究是甘肃中医药大学附属医院宋氏正骨科参与进行的临床试验。

2、作为受试者，将遵守受试者须知要求，志愿参加本实验，愿意自费承担自己治疗及相关检查。并与研究人员充分合作，如实、客观地向研究人员提供参加本研究前的健康状态以及相关情况。

3、本临床试验的结果只用于科研目的，除外研究者及监查员等，参加试验及试验中的个人资料均属保密，将依照法律规定得到保护。

4、自愿参加本研究，如果在临床试验中出现不可预见的不良反应，将得到医生及申办单位妥善积极的治疗，如果发生与治疗有关的严重不良事件，除得到妥善积极的治疗外，申办者将会负责由此引起的相关治疗费用及赔偿。

5、参加本临床试验完全是自愿的，可以拒绝参加或在任何时间退出试验，而不会遭到歧视和报复，医疗待遇与权益亦不会受影响。

受试者签名： 研究者签名：

日期： 年 月 日

中国知网 https://www.cnki.net

附录 2

体外冲击波及治疗图片

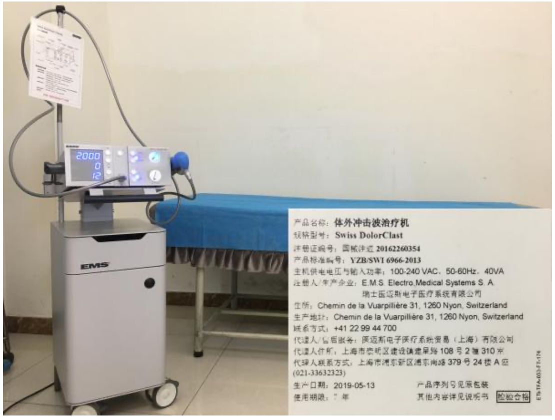

体外冲击波治疗机

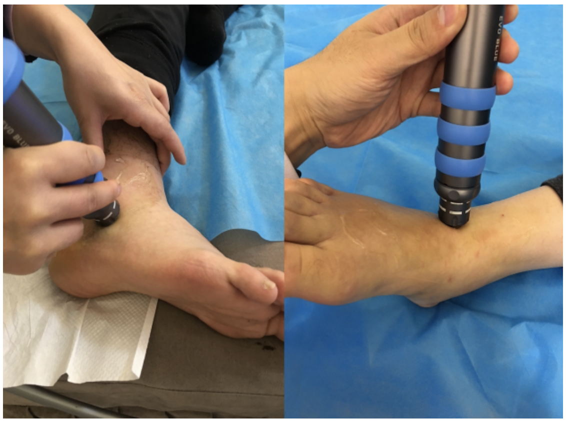

体外冲击波治疗图示 1

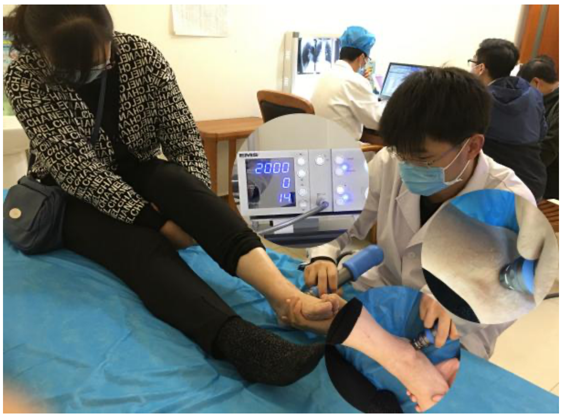

体外冲击波治疗图示 2

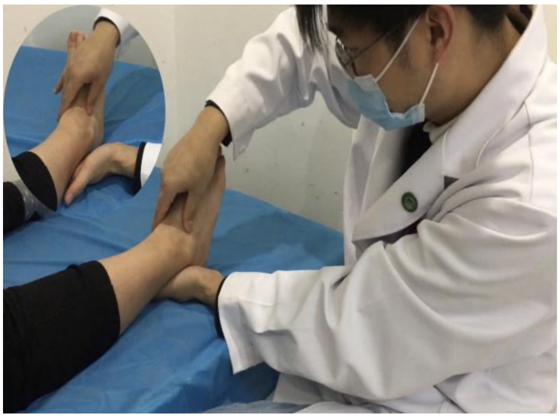

正骨理筋手法步骤 1

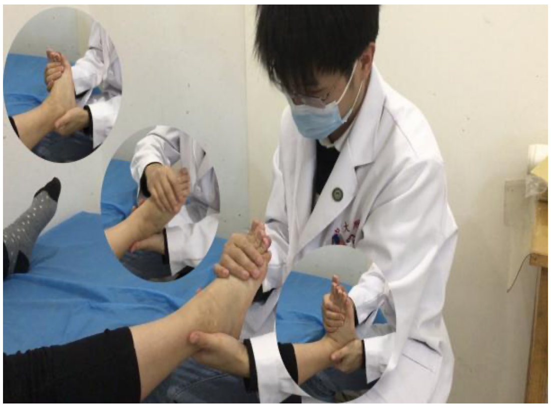

正骨理筋手法步骤 2

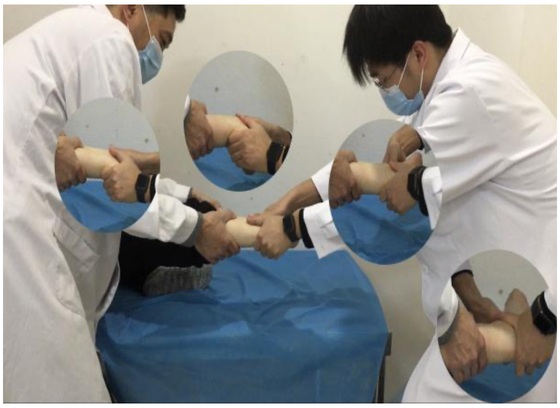

正骨理筋手法步骤 3

中国知网 https://www.cnki.net

附录 3

病例采集册

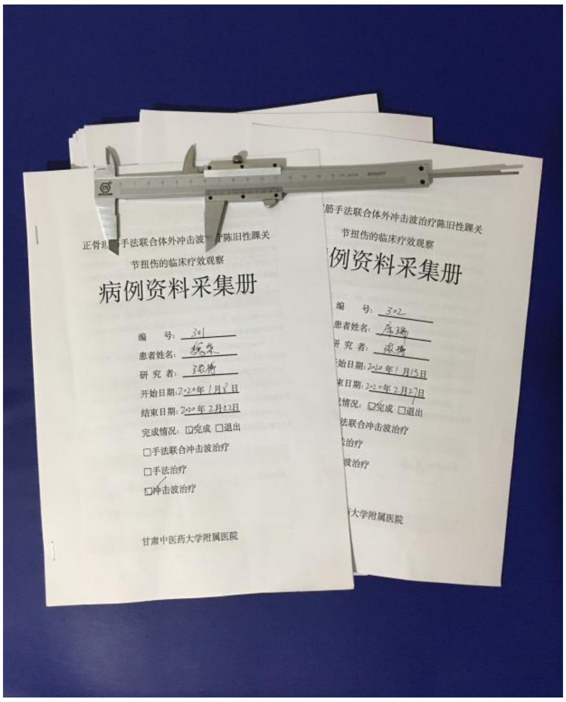

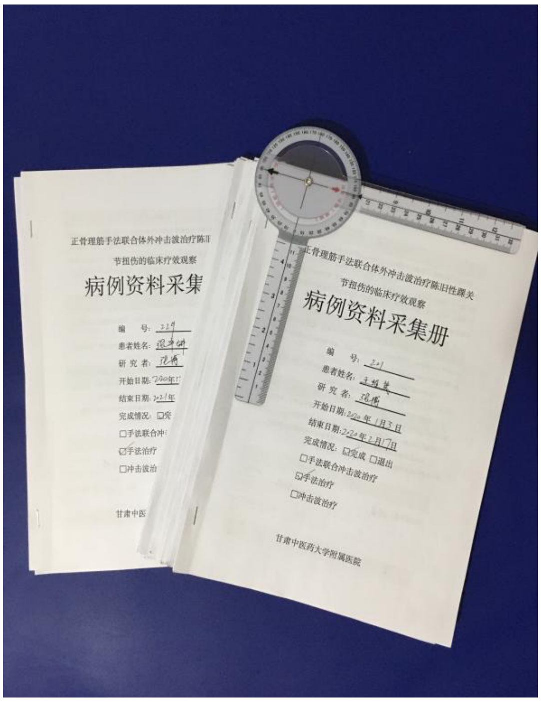

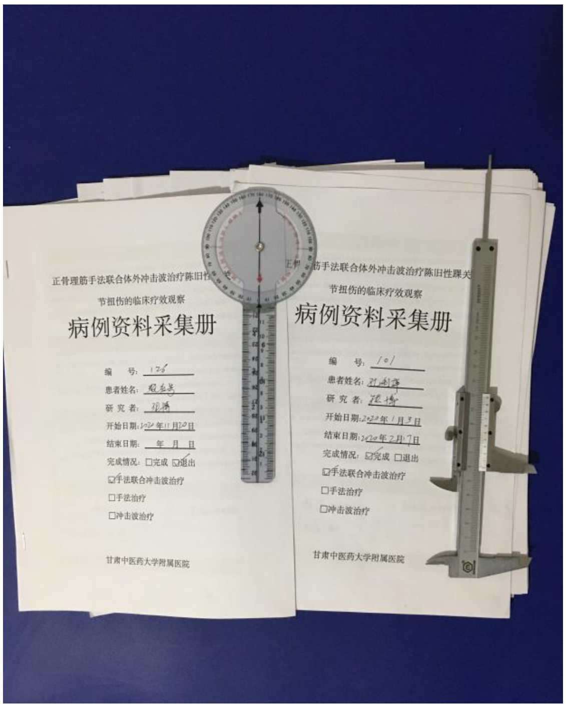

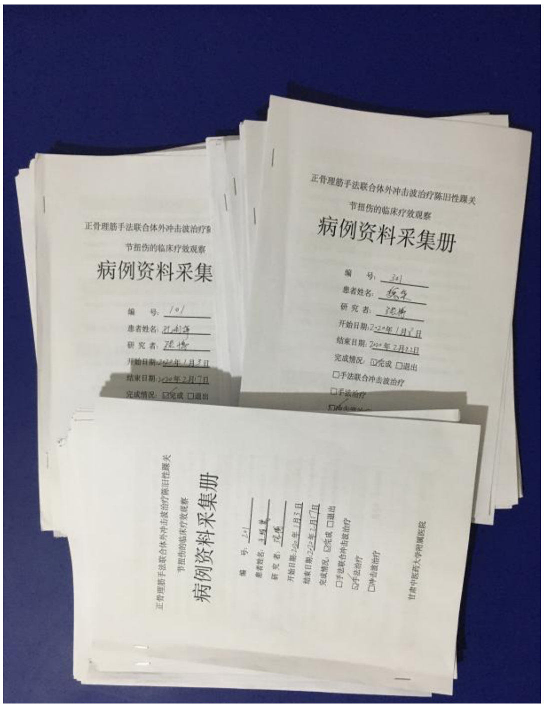

中国知网 https://www.cnki.net

附录 4

VAS 疼痛评分量表

姓名： 性别： 年龄： 编号：

无痛 最痛

0 1 2 3 4 5 6 7 8 9 10

在 VAS 疼痛评分卡标有 10cm 的刻度，两端分别表示无痛和最痛。分别记作 0 和 10。病人依据自己的主观感觉进行评分，研究者记录静止状态下 VAS 疼痛评分。

评分标准如下：
无疼痛： 0，无任何疼痛
轻度疼痛：1-3 微痛，时痛时止
中度疼痛：4-6 痛重，呈持续性
重度疼痛：7-9 痛剧，需服止痛药
极度疼痛： 10 痛极，无法忍受

治疗前 日期： 年 月 日

无痛 最痛

0 1 2 3 4 5 6 7 8 9 10

治疗 2 周后 日期： 年 月 日

无痛 最痛

0 1 2 3 4 5 6 7 8 9 10

治疗后 1 个月 日期： 年 月 日

无痛 最痛

0 1 2 3 4 5 6 7 8 9 10

中国知网 https://www.cnki.net

附录 5

Baird-Jackson(1987)踝关节评分量表

姓名:　　性别:　　年龄:　　编号:　　日期:　　年　　月　　日

Baird-Jackson(1987)踝关节评分	分数	计分
1.疼痛		
（1）无痛。	15 分	
（2）剧烈活动时轻微疼痛。	12 分	
（3）日常活动时轻微疼痛。	8 分	
（4）负重时疼痛。	4 分	
（5）静息时疼痛。	0 分	
2.踝关节稳定性		
（1）无临床不稳定。	15 分	
（2）体育运动时不稳定。	5 分	
（3）日常活动时不稳定。	0 分	
3.行走能力		
（1）能行走，距离不受限制，无跛行、无疼痛。	15 分	
（2）能行走，距离不受限制，有轻度跛行或疼痛。	12 分	
（3）行走能力中度受限。	8 分	
（4）仅能行走较短距离。	4 分	
（5）不能行走。	0 分	
4.跑步能力		
（1）能跑步，距离不受限制，无疼痛或跛行。	10 分	
（2）能跑步，距离不受限制，有轻微疼痛。	8 分	
（3）跑步能力中度受限，有轻度疼痛。	6 分	
（4）仅能跑较短距离。	3 分	
（5）不能跑步。	0 分	

中国知网　https://www.cnki.net

5.工作能力		
（1）能完成一般职业工作，工作不受限。	10 分	
（2）能完成一般职业工作，但剧烈活动时受限。	8 分	
（3）能完成一般职业工作，但明显受限。	6 分	
（4）部分残疾，仅能选择性工作。	3 分	
（5）不能工作。	0 分	
6.踝关节活动范围		
（1）低于正常踝关节的 10 以内。	10 分	
（2）低于正常踝关节的 15 以内。	7 分	
（3）低于正常踝关节的 20 以内。	4 分	
（4）低于正常踝关节的 50%，或背屈小于 5°。	0 分	
7.放射学结果		
（1）踝关节恢复解剖对位关系，内侧关节间隙正常，踝穴上关节间隙正常，无距骨倾斜。	25 分	
（2）基本结果与上述一致，但关节边缘有轻度增生反应性改变。	15 分	
（3）踝穴上关节间隙轻度变窄，但踝穴上关节间隙仍大于 2mm，或距骨倾斜大于 2mm。	10 分	
（4）踝穴上关节间隙中度变窄，在 1-2mm。	5 分	
（5）踝穴上关节间隙重度变窄，小于 1mm；内侧关节间隙变宽有重度增生反应性改变（软骨下骨质硬化骨赘形成）。	0 分	

结果:优，96-100 分；良，91-95 分；可，81-90 分；差，0-80 分。

中国知网 https://www.cnki.net

附录 6

Kofoed 评分量表

姓名：　　性别：　　年龄：　　编号：　　日期：　　年　　月　　日

疼痛（满分 50 分，为基本分）		功能（满分为 30 分）	
无疼	50	足趾行走	3
行走开始时疼痛	40	足趾行走	3
行走时疼痛	35	正常节律上下楼梯	6
偶尔负重时都有疼痛	25	单腿站立	6
每次负重时都有疼痛	15	无辅助性行走	6
检查时疼痛或自发疼痛	0	不用骨科足支具	6
活动度（满分 20 分）			
伸＞10°	5	旋后＞30°	5
5-9°	3	15-29°	3
＜5°	1	＜15°	1
屈＞30	5	旋前＞20°	5
15-29°	3	10-19°	3
小于 15°	1	＜10°	1
负重时外翻＜5°	2	负重时内翻＜3°	2
5-10°	1	4-7°	1
＞10	0	＞7	0
总分			

（分数低于 70 分者为差；70-74 分为及格；75-80 分为良好；85-100 分为优）

中国知网 https://www.cnki.net

附录 7

肿胀程度

姓名：　　性别：　　年龄：　　编号：

	访视一	访视二	访视三	访视四
轻度肿胀				
中度肿胀				
重度肿胀				

按照《中药新药临床研究指导原则》，并参照软组织损伤症状分级标准（尺标法健侧对比），采用健侧和患侧测量对比的方法，用游标卡尺测量双侧踝关节中心 3 次并取其差值的平均值。

轻度：双侧宽度差值小于等于 0.5cm；

中度：双侧宽度差值介于 0.5cm 和 1.0cm 之间；

重度：双侧宽度差值大于等于 1.0cm。

治疗前　　日期：　　年　月　日

肿胀程度：

治疗 2 周后　　日期：　　年　月　日

肿胀程度：

治疗后 1 个月　　日期：　　年　月　日

肿胀程度：

中国知网 https://www.cnki.net

附录 8

医学伦理审查表

甘肃中医药大学附属医院伦理委员会

甘中附院伦理委员会发[2019]62 号

关于审查“正骨理筋手法联合体外冲击波治疗陈旧性踝关节扭伤的临床疗效观察”项目的通知

2019 年 12 月 2 日医院伦理委员会对“正骨理筋手法联合体外冲击波治疗陈旧性踝关节扭伤的临床疗效观察”的项目进行了详细的伦理审查，认为该项目符合《临床研究伦理审查管理规范》等各项相关规定。

甘肃中医药大学附属医院伦理委员会

二〇一九年十二月二日

主题词：审查 正骨理筋手法 体外冲击波 陈旧性踝关节扭伤

伦理委员会办公室 2019 年 12 月 2 日印发

中国知网 https://www.cnki.net

致 谢

时光荏苒，转眼三年研究生生活结束，即将进入人生下一阶段，回首往昔历历在目，有奋斗、有蹉跎，有欢颜、有泪目，构成这段时光最美好的记忆。

首先以最诚挚的心感谢导师姜劲挺教授，感谢恩师在学习上孜孜不倦的教诲，在生活中无微不至的关怀。跟随恩师学习，受益颇深，必将终生铭记！

感谢甘肃中医药大学附属医院宋氏正骨科宋鹏程主任，跟随宋主任学习收益匪浅，在宋主任的门诊上完成病例搜集，得以完成毕业论文的撰写，感谢宋主任的照顾！

感谢甘肃中医药大学中医临床学院宋敏教授、曹林忠教授、秦大平班主任对 2018 级全体中医骨伤研究生的关心、教导！

感谢甘肃中医药大学附属医院宋氏正骨科诸位老师，在临床中的言传身教，让我对专业知识有更深入的了解，感谢你们的关爱照顾，感谢你们创造了一个学习进步的好空间、好氛围！

感谢李祥雨、徐欣、王强强师兄在我学习过程中不吝指导及生活中的关怀！感谢同窗及室友在学习生活中的帮助、鼓励！

感恩父母家人，感谢你们的辛勤付出，今后我定将努力拼搏，更进一步！

感谢各位专家教授在百忙之中来参加我的论文答辩并予以指导，在此谨向全体参加论文工作、并予以指导的专家教授表示衷心感谢！

中国知网 https://www.cnki.net

研究生学习期间主要研究成果

1 发表论文情况

[1]张博,姜炫合,郑吉元,马宏武,窦中信,徐欣,王强强,指导：姜劲挺.基于聚类分析总结姜劲挺治疗腰痛用药规律研究[J].新中医,2021,53(8):201-205.

[2]张博,姜炫合,姜劲挺,郑吉元,马宏武,窦中信.姜劲挺教授治疗骨伤科痹证经验总结[J].中医临床研究,（已录用，2021 年 4 月 12 期刊载）.

[3]徐欣,姜劲挺,王强强,马理元,李祥雨,杨波,张博.中医骨伤科对慢性踝关节不稳定的认识及治疗进展[J].中国医药科学,2019,9(19):55-58.

[4]王强强,徐欣,马理元,李祥雨,杨波,张博.姜劲挺治疗椎动脉型颈椎病(肾虚肝郁型)经验总结[J].陕西中医药大学学报,2020,43(06):19-21.

中国知网 https://www.cnki.net
