# Supplementary material for: Efficacy and safety of Tuina (Chinese Therapeutic Massage) for chronic ankle instability: A systematic review and meta-analysis of randomized controlled trials
Source: PLoS One. 2025 Jun 6;20(6):e0321771. doi: 10.1371/journal.pone.0321771 (PMC12143534; doi:10.1371/journal.pone.0321771)
Supplement: S2 File — (ZIP) [file pone.0321771.s004.zip › 4.詹氏正骨手法配合中药薰药治...陈旧性踝关节扭伤的临床研究_范青红.pdf]

同等学力申请硕士学位

浙江中醫藥大學

硕士学位论文

论文题目：詹氏正骨手法配合中药薰药治疗陈旧性踝关节扭伤的临床研究

作者姓名：范青红

指导教师：王人彦教授

学科专业：中医学中医骨伤科学

提交日期 2016年11月

中国知网 https://www.cnki.net

中国知网 https://www.cnki.net

目 录

中文摘要……I
ABSTRACT……III
前 言……1
一、临床资料……3
(一)病例来源……3
(二)病例选择……3
1.诊断标准……3
2.纳入标准……4
(三)排除标准……4
(四)脱落标准……4
二、研究方法……5
(一)分组方法……5
(二)治疗设施……5
1.熏药处方……5
2.仪器……5
(三)治疗方法……5
1.治疗组……5
2.对照组……7
(四)观察指标……7
1.VAS 疼痛评分……7
2.Kofoed 关节功能评分量表……7
3.疗效评定标准……7
4.不良事件观察……7
(五)统计方法……7
三、研究结果……9
(一)局部压痛情况……9
(二)Kofoed 关节功能评分……9

中国知网 https://www.cnki.net

1.关节疼痛评分......9
2.功能活动度评分......10
(三)治疗前后两组疗效评价......10
(四)不良反应观察......11
四、分析与讨论......13
(一)詹氏正骨手法治疗陈旧性踝关节扭伤......13
1.詹氏正骨手法治疗陈旧性踝关节扭伤的应用原则原理......13
2.詹氏正骨手法治疗陈旧性踝关节扭伤......13
3.詹氏正骨手法治疗陈旧性踝关节扭伤的优点优势......15
(二)詹氏正骨手法在其他筋伤疾病中的应用......15
1.詹氏正骨手法治疗筋伤疾病治疗前准备......15
2.詹氏正骨手法治疗筋伤疾病的特点......16
3.詹氏正骨手法治疗筋伤疾病的作用原理......17
4.詹氏正骨手法治疗筋伤疾病的选用原则......18
5.詹氏正骨手法的使用原则......18
(三)中药熏药及选方依据......19
1.传统医学对中药薰药的理论认识......19
2.选方依据......19
结论......21
参考文献......22
附　表......24
致谢......27
文献综述......28

中国知网　https://www.cnki.net

中文摘要

詹氏正骨手法配合中药熏药治疗陈旧性踝关节扭伤的临床研究

目的：通过詹氏正骨手法配合中药薰药治疗与单纯中药薰药治疗陈旧性踝关节扭伤的对比研究，综合比较詹氏正骨手法配合中药熏药治疗与单纯中药熏药治疗陈旧性踝关节扭伤之间的疗效差异，从疗效指标，多角度、多环节的评价詹氏正骨手法配合中药熏药的疗效。通过临床实践的研究、总结，研究手法治疗机理，建立詹氏正骨手法治疗陈旧性踝关节扭伤的诊疗规范，制定詹氏正骨手法治疗陈旧性踝关节扭伤的操作流程。以期为临床治疗提供参考依据。

方法：根据纳入标准及排除标准，借助 SPSS19.0 统计分析系统对 60 例来源于詹氏医院门诊的陈旧性踝关节扭伤（筋脉失养型）患者随机分为 2 组，每组各 30 例。其中治疗组采用詹氏正骨手法配合中药薰药治疗，对照组单纯采用中药薰药治疗，对照组：中药薰药治疗方剂为来源于《中医伤科讲义》经验方“四肢损伤洗方”，使用 XZQ-III型中药熏蒸器治疗，熏药时间为 20 分钟，每日一次。7 次为一疗程，共治疗两个疗程。治疗组 30 例同样使用四肢损伤洗方中药熏药后，再采用詹氏正骨手法治疗。每天一次，每次 15 分钟，每 7 天为 1 个疗程，共治疗两个疗程。然后进行治疗前后 VAS 疼痛评分和 Kofoed 关节功能评分及根据《中医病证诊断疗效标准》对疗效进行评价，进行数据统计分析，来制定手法治疗的诊疗规范，形成詹氏正骨手法治疗陈旧性踝关节扭伤的标准操作流程。

结果：

(1) 总体疗效：治疗组（詹氏正骨手法配合中药薰药治疗）30 例，治愈 13 例，好转 16 例，未愈 1 例。对照组（单纯中药薰药治疗）30 例，治愈 5 例，好转 20 例，无效 5 例。经卡方检验，两组差异有统计学意义（$P<0.05$），表明两种方法治疗陈旧性踝关节扭伤均有效，但詹氏正骨手法配合中药薰药治疗疗效优于单纯中药薰药治疗。

(2) VAS：两组受试者治疗前后局部压痛分值比较，治疗前两组局部压痛积分 $P>0.05$，差异无统计学意义，具有可比性。治疗组与对照组治疗前后局部压痛分值相比，治疗后一周开始往后所有 P 值均<0.05，差异有统计学意义。可见从治疗后一周起治疗组疼痛减轻方面就优于对照组。

(3) 症状积分：治疗前两组症状积分 $P>0.05$，差异无统计学意义，具有可比

中国知网 https://www.cnki.net

性。治疗组与对照组评分相比，治疗后一周开始往后所有 P 值均<0.05，差异有统计学意义。可见从治疗后一周起治疗组症状减轻方面就优于对照组。

(4)疼痛、功能活动度积分：两组患者治疗前疼痛积分与功能活动积分比较 $P>0.05$，差异无统计学意义，具有可比性。治疗组与对照组疼痛积分比较，两组治疗一周后组间疼痛评分比较($P>0.05$)差异无统计学意义；治疗两周及三个月后随访两组间比较差异有统计学意义（$P<0.05$），说明治疗组与对照组在治疗一周后疼痛评分改善差异不明显，治疗两周及三个月后治疗组疼痛评分改善方面优于对照组。治疗组与对照组功能活动积分相比，治疗后一周开始往后所有 P 值均<0.05，差异有统计学意义。可见从治疗后一周起治疗组功能活动度改善方面就优于对照组。

结论：本研究治疗组采用詹氏正骨手法配合中药薰药治疗陈旧性踝关节扭伤，对照组单纯使用中药薰药治疗陈旧性踝关节扭伤。受试者经治疗后在症状、体征改善上均比较明显，两种治疗方案对陈旧性踝关节扭伤均有显著疗效。治疗组的疼痛、功能和活动度的改善好于对照组；在关节功能上面治疗组明显优于对照组。而且在总体疗效上治疗组明显好于对照组。可见詹氏正骨手法配合中药薰药与单纯中药薰药治疗陈旧性踝关节扭伤相比在疗效的改善方面更具优势，对于陈旧性踝关节扭伤患者的总体疗效更佳，值得推广使用。

主题词 詹氏正骨手法，中药薰药，陈旧性踝关节扭伤

中国知网 https://www.cnki.net

Abstract

Jane bonesetting skill with herbal fumigation medicine research new bed for the treatment of chronic ankle sprain

Objective: By Jane bonesetting skill with traditional Chinese medicine fumigation therapy and pure Chinese medicine fumigation in the treatment of old ankle sprain are comparative study,comprehensive comparison of Jane bonesetting skill with traditional Chinese medicine fumigation treatment and pure Chinese medicine fumigation treatment the curative effect of the difference between old ankle sprain,from curative effect,muiti-angle,much link to evaluate the effect of Jane bonesetting skill with herbal fumigation medicine.Through the study of clinical practice,summed up,the research technique treatment mechanism,estabish a specification of Jane bonesetting skill treatment of old ankle sprain are diagnosis and treatment,make Jane bonesetting skill operation process for the treatment of old ankle sprain.In order to provide reference basis for clinical treatment.

Methods: According to the inclusion criteria and exclusion criteria,using SPSS19.0 statistical analysis system of 60 cases from hangzhou Jane hospital outpatient service in patients with old ankle sprain(channels alongwith type) are randomly divided into 2 groups,each group 30cases.Among them with Chinese medicine treatment group use technique of Jane fumigation treatment,the control group only with Chinese medicine fumigation treatment, the control group:herbal fumigation medicine treatment formula is derived from experience in teaching materials on Chinese medicine& experience part"limbs damage the party".Steam use XZQ-ⅢChinese medicine fumigation device, fumigation time for 20 minutes,once a day.7 times for a period of treatment, treatment of two period of treatment.Treatment group of 30 patients using the same limb injury party after Chinese medicine fumigation washing,use technique of Jane treatment again.Once a day,15 minutes every time,every 7 days for a period of treatment,a total of two courses of treatment.The pain VAS scores before and after treatment and Kofoed joint function score and according to the "standard of disease diagnosis curative effect of traditional

中国知网 https://www.cnki.net

Chinese medicine "to evaluate the curative effect,statistical and analysis,to make a diagnosis and treatment for the treatment of Jane technique standard operating procedure for the treatment of old ankle sprain.

Results:

(1)The overall effect:the treatment group,30 cases,13 cases were cured,16cases were markediy effective , 1 case had no effect.Control group of 30 cases,5 cases were cured,20 cases were markediy effective, 5 cases had no effect .By chi-square test,the differences between the two groups have statistical significance(P<0.05).On the curative effect of treatment group was obviously better than the control group.

(2)VAS:Local tenderness score comparison before and after treatment,the two groups before treatment in the two groups of local tenderness points P>0.05,no statisically significant difference,comparable. Local tenderness score in treatment group and ontrol group before and after treatment,compared to a week after treatment began to back all P values<0.05,with statistical significance.See from the reduction in pain a week after the treatment,the treatment group was better than that in control group.

(3)Symptom score:The treatment of the two groups before symptom score P>0.05, no statisically significant difference, comparable.The treatment group compared with the control group scores,compared to a week after treatment began to back all P values<0.05,with statistical significancedifference.See the symptom is reduced from a week after the treatment,the treatment group was better than that in control group.

(4)Pain,functional activity Points:In the two groups before treatment inpatients with pain and functional activity integral comparison P>0.05,no statistical significancedifference,comparable.The treatment group compared with the control group pain points,pain score comparison between the two group a week after treatment (P<0.05) no statistically significant difference.Two weeks and three months after follow-up of comparative difference between the two groups have statistical significance(P<0.05), treatment group and control group no obvious difference in the treatment of pain score improved after one week, Two weeks and three months after treatment the treatment group pain score improved better than the control group.

中国知网 https://www.cnki.net

Treatment compared with the integration of the activities and functions with the control,a week after the start back all *P* values<0.05,with statistical significancedifference. Visible from a week treatment group after treatment function improved activity is superior to the control group.

Conclusion: This study with Chinese medicine treatment group use technique ofJane smoked medicine treatment of chronic ankle sprain,control group simply use herbal fumigation medicine treatment of chronic ankle sprain.The subjects on the signs and symptoms improved after treatment are relatively obvious,two kinds of treatment for chronic ankle sprains are all have remarkable curative effect.The pain in treatment group,the improvement of function and activity is better than the control group;On the joint function in treatment group was better than the control group.And on the overall effect treament group significantly better than the control group.VisibleJane bonesetting skill with herbal fumigation medicine compared with pure herbal fumigation medicine treatment of chronic ankle sprains are more advantages in the improvement curative effect,for the overall efficacy of patients with chronic ankle sprains are much better,is worthy of popularization and application.

Subjectwords Jane bonesetting skill,Herbal fumigation medicine,old ankle sprain

中国知网 https://www.cnki.net

前　言

踝关节扭伤为骨科门诊常见病、多发病，可见于各个年龄段。由于踝关节韧带损伤早期在X线片上往往不能显示[1]，导致患者和医生对其给予的重视不够，处理不及时或治疗不恰当，最终发展为关节反复肿胀疼痛，关节僵硬，活动不利甚者功能障碍，即发展为陈旧性踝关节扭伤。或遇阴雨天气，关节酸痛重着，由于外伤瘀血凝结，积久不散，或外感风寒湿邪，痹阻经络，以致伤处气血滞涩，血不养筋，筋肉挛缩[2]。

随着人们参加体育活动的增加，近年来踝关节损伤的人数有逐步上升的趋势。西医治疗踝关节陈旧性扭伤分为保守治疗和手术治疗，保守治疗方法比较单一，主要有固定和功能治疗如不同的外部支持、石膏固定或胶布固定等，局部外用镇痛类膏剂外涂或内服镇痛类药物，平衡训练康复等[3,4]。手术治疗过程中手术的指征、手术的利弊以及远期效果难以权衡。中医治疗由于其安全性、经济性而有着良好的发挥空间，而且方法很多。其中传统中医手法治疗陈旧性踝关节扭伤疗效比较确切，但对手法的规范化使用及诊疗规范尚不十分明确。本项目通过詹氏正骨手法配合中药薰药与单纯中药薰药治疗陈旧性踝关节扭伤的对比研究，在治疗陈旧性踝关节扭伤方面进行规范化研究，建立手法治疗的诊疗规范，同时研究其治疗机理，指导临床，利于教学与推广。

中国知网 https://www.cnki.net

中国知网 https://www.cnki.net

一、临床资料

(一)病例来源

研究对象来源于我院 2014 年 5 月至 2015 年 5 月中医骨伤科、康复医学科及针灸推拿科门诊的陈旧性踝关节扭伤患者，共计 60 例。所有病例均为单侧踝关节扭伤。

(二)病例选择

1. 诊断标准

1.1 中医诊断标准：参照 2011 年国家中医药管理局制定的踝关节扭伤中医诊疗方案[5]，诊断和症候分类为：

(1)血瘀气滞：损伤早期，踝关节疼痛，活动时加剧，局部明显肿胀及皮下瘀斑，关节活动受限；舌红有瘀点，脉弦；

(2)筋脉失养：损伤后期，关节持续隐痛，轻度肿胀，或可触及硬结，步行乏力；舌淡，苔薄，脉弦细。

其中，陈旧性踝关节扭伤属于筋脉失养型。

1.2 西医诊断标准：参照《实用骨伤科手册》中关于陈旧性踝关节扭伤诊断标准[6]

(1)有明显外伤史；

(2)踝关节肿胀、酸痛乏力，关节活动时可有摩擦感，久行、阴雨天时加重；

(3)外踝前下方及内踝前外侧有肿胀、压痛，内翻、屈伸时活动可受限；

(4)检查未发现骨折和脱位，X 线摄片检查未见骨折；

(5)病程超过 20 天未愈者；

注：综合临床、实验室及 X 线检查，符合 1、2、5 条或 1、3、5 条或 1、3、4、5 条或 1、2、4、5 条，可诊断为陈旧性踝关节扭伤。

1.3 踝关节韧带损伤分度[7,8]：

Ⅰ度损伤：韧带拉伤，关节无不稳：韧带有所松弛；

Ⅱ度损伤：韧带部分断裂，轻度不稳；

Ⅲ度损伤：韧带完全断裂，明显不稳，一般的 X 光片可发现骨折。

研究对象为Ⅰ度韧带损伤患者。

2. 纳入标准

中国知网 https://www.cnki.net

(1)来源于我院中医骨伤科、康复医学科及针灸推拿科门诊陈旧性踝关节扭伤患者；

(2)符合上述陈旧性踝关节扭伤的诊断标准，且均为单踝扭伤，均为外侧韧带损伤，病程超过20天未愈者；

(3)近期未用过药物或其他方法治疗；

(4)既往无接触性皮炎等皮肤病病史；

(5)签署知情同意书；

(6)伦理管理委员会同意。

(三)排除标准

(1)对所用中药熏药方中药物过敏者或多敏体质患者；

(2)妊娠期及哺乳期妇女；

(3)局部皮肤软组织破损或有伤口感染者；

(4)不符合陈旧性踝关节扭伤诊断标准或纳入标准；

(5)合并有恶性肿瘤、骨折、骨髓炎等其它踝关节病变患者；

(6)有严重心脑、肝、肾疾病或者精神病病史患者；

(7)糖尿病患者；

(8)凝血功能障碍的患者；

(9)治疗过程中不合作或者同时在进行其它治疗的患者；

(10)踝关节韧带Ⅱ度及Ⅲ度损伤患者。

注：符合上述排除标准中任一项者即要终止临床研究。

(四)脱落标准

(1)纳入病例在研究期间出现严重不良反应或并发症不能完成治疗的状况；其他不可预测情况发生使治疗不能继续。

(2)治疗中自行要求退出或者未能完成整个治疗过程的病例。

中国知网 https://www.cnki.net

二、研究方法

(一)分组方法.

根据病例纳入标准及排除标准，确定受试者，借助 SPSS19.0 统计分析系统进行随机分组，将 60 例受试者分为治疗组（詹氏正骨手法配合中药熏药）和对照组（单纯使用中药熏药），每组 30 例。受试者年龄在 18-53 岁之间，病程为 20-60 天之间，其中治疗组男 13 例，女 17 例，右踝 18 例，左踝 12 例，平均年龄（30.60±8.07)岁，平均病程（31.60±9.97）天；对照组男 10 例，女 20 例，右踝 19 例，左踝 11 例，平均年龄（28.50±7.16）岁，平均病程（32.96±8.50）天。治疗组与对照组基本资料比较见下表 1，经统计分析，两组在性别、损伤部位、年龄和病程构成方面差异无统计学意义（P>0.05)，具有可比性。

表 1 两组基本资料比较

组别	性别（例）		部位（例）		年龄（岁）	病程（天）
	男	女	左踝	右踝		
治疗组	13	17	12	18	30.60±8.07	31.60±9.97
对照组	10	20	11	19	28.50±7.16	32.96±8.50
检验值	X^2=0.533		X^2=1.200		t=1.159	t=-0.677
P 值	1.000		0.063		0.256	0.504

(二)治疗设施

1.熏药处方：熏药处方使用来源于《中医伤科讲义》的经验方“四肢损伤洗方”，组成为原方，剂量由作者自行设定。组方[9]为：桑枝、桂枝、伸筋草、透骨草各 25 克，怀牛膝、木瓜各 15 克，乳香、没药、红花各 30 克，羌活、独活、落得打各 20 克，补骨脂、淫羊藿、萆薢各 30 克。以上各药切成片，混合均匀，分装成袋，每袋 125 克。每袋中药，煎煮为 150 毫升药液装为一袋备用。

2.仪器：XZQ-III型中药熏蒸器，产地：常州。

(三)治疗方法

1.治疗组

先加适量的温水，再将煎好的药液一袋（150 毫升）加入治疗仪器的容器内，接通电源，打开总开关，根据要求在控制面板上设定各参数，温度设定在 38-42 度之间，时间为 20 分钟，加温到指定温度后，嘱患者患踝充分暴露，并检查治疗部位皮肤有无破损。操作床上垫一次性中单，嘱患者侧卧于操作床上，若外踝损伤健侧卧位，健侧下肢屈曲，患侧下肢伸直；若内踝损伤患侧卧位，健侧下肢

中国知网 https://www.cnki.net

屈曲，患侧下肢伸直，将熏蒸探头对准患处，探头与患处之间间隔 20-30cm，治疗开始，询问病人温度是否合适，体位是否舒服。治疗过程加强巡视，密切观察患者有无不适，熏蒸温度有无异常等等。治疗完毕关闭仪器，用毛巾擦拭熏蒸部位，同时观察局部皮肤情况。每日一次，每次 20 分钟。

中药熏药后使用詹氏正骨手法进行治疗。具体手法操作步骤如下：

(1)舒筋。患者仰卧于操作床上，双下肢平伸，全身放松，呼吸自然。医者左手扶住患踝，使患踝保持中立位，右手拇指及其余四指由远及近揉捏小腿肌肉三头肌及胫前肌等。然后用右手拇食指沿着内外踝的边缘做缓慢的推法。若发现肌肉、筋膜、肌腱等软组织增粗、变硬、挛缩、粘连时，予以按压、拨推手法，手法作用力偏重以病人能忍受为度，来回 10 次。

(2)点按阿是穴、足三里、丰隆、承山、阳陵泉、解溪、昆仑、悬钟、丘墟、申脉等穴。每个穴位点压 10-15 秒。

(3)按摩足背及足底。术者用左手固定足部，即用左手拇指及其余四指捏握住患足足趾端，用右手拇指关节沿足背肌腱走行方向由远及近，从外侧到内侧缓慢的推。然后使用左右两手分别拿捏足的内外侧，用双拇指的罗纹面自足背的中央分别向两边分开移动，来回 5 次，即使用分推法，要求两手用力均匀动作柔和，协调一致。然后用右手食指第二关节沿足底外侧缘、足底中线、足底内侧缘从足趾向足跟部缓慢的使用推法，其余四指呈握拳状，来回 5 次。

(4)牵引拔伸运踝。术者一手按住患踝关节前方，一手牵拉患足拇趾，患足其余 4 趾放松，牵拉时要静止用力，持续用力 10 秒钟，然后依次进行牵拉其余四趾。然后术者用右手拇指及其余四指捏握住患足足趾端，左手托住在足跟处，同时左手拇、食指分别压在内、外踝前下方的间隙处，在夹持踝关节的同时，右手在牵引拔伸下作顺时针运踝，然后在跖屈踝关节到最大范围下，再背伸踝关节至最大范围。最后放松足部及小腿周围肌肉。手法的整个过程用时 15 分钟左右。

治疗期间，还应嘱受试者配合，避免患侧大强度的运动，如避免剧烈的跑跳、爬山等活动，避免踝关节再次扭伤。

治疗时间为 2 个疗程，每个疗程 7 天。2 疗程治疗后，仍继续观察，定期追踪随访三个月。

中国知网 https://www.cnki.net

2. 对照组

对照组单纯使用中药薰药治疗。熏药处方及熏药方法同治疗组。治疗期间，注意事项同治疗组。治疗时间为2个疗程，7天为一疗程。2疗程治疗后，仍继续观察，定期追踪随访三个月。

(四)观察指标

1. 局部压痛 VAS 疼痛评分

对踝关节压痛点进行压痛检查，观察患者疼痛情况，采用 VAS 疼痛评分，即使用一根长10cm的不透明直尺，直尺两端 “0”和“10”刻度，分别代表不痛和疼痛无法忍受[10]。检查时医师将直尺没有刻度的一面对着病人，让病人凭主观感觉指出疼痛程度的相应部位，根据病人的指出位置进行评分[11]。(如附录1所示)

2. Kofoed 关节功能评分量表

此评分量表[12]对疼痛，功能，活动度三部分进行评分，总分为100分，评分标准详见附录2。

3. 疗效评价标准

参照《中医病证诊断疗效标准》[13,14]

(1)治愈：踝关节肿痛消失，关节稳定，踝关节活动功能正常；

(2)好转：踝关节疼痛减轻，轻度肿胀或皮下瘀斑，关节欠稳，步行欠力，酸痛；

(3)未愈：踝关节疼痛无改善，关节不稳定，活动受限。

4. 不良事件观察：详细记录出现不良事件的起始时间、采取的措施及转归，判断不良事件与治疗是否有关，是否纠正治疗、是否退出试验。

(五)统计方法

分别在治疗前、治疗一周后、治疗两周后和治疗三个月后随访时，进行 VAS 疼痛评分、Kofoed 关节功能评分和整体疗效进行评价。借助 SPSS19.0 统计分析系统进行统计分析，计数资料采用 X^2 检验，计量资料采用 t 检验；以 $P<0.05$ 作为具有统计学意义的标准，统计总结、评价其疗效由专人负责，统计者不参与实验观察，以排除主观偏倚。

中国知网 https://www.cnki.net

中国知网 https://www.cnki.net

三、研究结果

(一)局部压痛情况

局部压痛采用 VAS 疼痛评分，经 t 检验结果见表 2，治疗前治疗组与对照组比较差异无统计学意义(*P*>0.05)，具有可比性。治疗前后两组内局部压痛差异有统计学意义（*P*<0.05)，表明治疗后压痛均有明显改善，两种方法治疗本病均有效。治疗后两组间比较各观察时段压痛 VAS 评分差异有统计学意义(*P*<0.05)，治疗组的疗效明显优于对照组。

表 2 治疗前后两组组间局部压痛评分比较（VAS 法）

组别	治疗前	一周后	两周后	三个月后
治疗组	5.70±1.32	3.07±0.91	1.23±0.77	0.63±0.72
对照组	5.67±1.15	3.63±0.72	2.60±0.81	2.23±1.17
检验值	t=0.095	t=-0.538	t=-5.761	t=-5.442
P 值	0.925	0.017	0.000	0.000

(二)Kofoed 关节功能评分

总症状积分采用 Kofoed 关节功能评分，采用 t 检验结果见表 3：治疗前两组比较差异无统计学意义(*P*>0.05)，具有可比性。治疗前后（一周、两周、三个月随访）两组内总症状积分比较差异有统计学意义（*P* <0.05)，说明两种方法治疗本病均有效。治疗后两组间比较差异有统计学意义（*P*<0.05)，说明治疗组疗效明显优于对照组。

表 3 治疗前后两组组间症状积分比较

组别	治疗前	一周后	两周后	三个月后
治疗组	63.7±7.97	74.86±5.61	84.3±8.82	85.8±8.84
对照组	65.6±8.53	69.70±6.78	76.43±6.47	78.2±7.15
检验值	t=-0.896	t=3.707	t=4.261	t=4.341
P 值	0.378	0.001	0.000	0.000

1. 关节疼痛评分

在 Kofoed 关节功能评分中，对踝关节疼痛评分进行比较，采用 t 检验结果见表 4：治疗前两组间踝关节疼痛评分比较差异无统计计意义(*P*>0.05)，具有可比性。两组组内治疗前和治疗一周后、两周后及三个月后随访比较差异均具有统计

中国知网 https://www.cnki.net

学意义（$P<0.05$），说明两种方法治疗本病均能有效改善踝关节疼痛。治疗一周后两组组间比较差异无统计学意义($P>0.05$)；治疗两周及三个月后随访两组间比较差异具有统计学意义（$P<0.05$），说明治疗组与对照组在治疗一周后对踝关节疼痛改善差异不明显，治疗两周及三个月后治疗组踝关节疼痛症状改善方面优于对照组。

表 4 治疗前后两组组间及组内疼痛评分比较

组别	治疗前	治疗一周后	治疗两周后	三个月后
治疗组	35.33±6.01	38.17±2.45	43.17±5.49	43.83±5.68
对照组	35.83±4.56	37.50±2.54	40.00±3.22	41.17±4.29
检验值	t=-0.356	t=1.072	t=2.726	t=2.333
P 值	0.725	0.293	0.011	0.027

2. 功能活动度评分

在 Kofoed 关节功能评分中，对踝关节活动度评分进行比较，采用 t 检验结果见表 5：治疗前两组踝关节活动度评分差异无统计学意义($P>0.05$)，具有可比性；治疗前后两组内比较差异均具有统计学意义($P<0.05$)，说明两种方法治疗本病均有效。治疗后（一周、两周、三个月随访）两组组间比较差异均具有统计学意义($P<0.05$)，表明治疗组在治疗后期改善踝关节活动度方面治疗组优于对照组。

表 5 治疗前后两组组间及组内功能活动度评分比较

组别	治疗前	治疗一周后	治疗两周后	三个月后
治疗组	18.20±2.61	22.00±2.98	23.80±3.24	24.20±2.94
对照组	18.20±3.60	19.70±3.41	21.70±3.02	21.80±2.94
检验值	t=0.000	t=2.841	t=2.841	t=3.378
P 值	1.000	0.008	0.006	0.002

(三)治疗前后两组疗效评价

参照《中医病证诊断疗效标准》进行疗效评价见表 6，经卡方检验，两组差异有统计学意义（$P<0.05$），表明两种方法治疗陈旧性踝关节扭伤均有效，但詹氏正骨手法配合中药薰药治疗疗效优于单纯中药薰药治疗。

中国知网 https://www.cnki.net

表 6 中医病证诊断疗效评定结果

组别	例数	治愈	好转	未愈	X^2	*P* 值
治疗组	30	13	16	1	120.00	0.00
对照组	30	5	20	5		

(四)不良反应观察

治疗组在整个治疗观察过程中无明显与治疗有关的不良反应发生。对照组其中 1 例患者在中药熏药后局部皮肤有灼痛感，皮肤较红。考虑与中药熏药时温度过高有关。治疗过程中予以调整熏药温度，并予以湿润烧伤膏外涂，经休息 3 个小时后恢复正常。没有影响实验的正常进行。

中国知网 https://www.cnki.net

中国知网 https://www.cnki.net

四、分析与讨论

陈旧性踝关节扭伤为慢性筋伤的一种。本研究选用詹氏正骨手法配合中药熏药治疗陈旧性踝关节扭伤。詹氏正骨手法是由詹氏骨伤创始人詹庄锡名老中医在传统推拿手法基础上自创的，一套讲究中医整体观的治疗筋伤疾病的手法。治疗了大量的陈旧性踝关节扭伤，取得了较好的治疗效果。

（一）詹氏正骨手法治疗陈旧性踝关节扭伤

1. 詹氏正骨手法治疗陈旧性踝关节扭伤的应用原则原理

陈旧性踝关节扭伤为中医慢性筋伤疾病，詹氏正骨手法治疗筋伤的作用原理为：通过不同力的作用使损伤病变组织的形态发生不同的变化，从而改善其结构，恢复其功能。在治疗陈旧性踝关节扭伤过程中詹氏正骨手法遵循以下选用和使用原则：

1.1 因人因病制宜：急性踝关节扭伤常发生于青壮年，陈旧性踝关节扭伤往往由扭伤早期治疗不当所致，是一种慢性筋伤疾病，故一般选用深透性较强的手法，每次 15 分钟左右为宜，每日一次。但治疗过程中需观察病人的反应，强度以病人能够耐受为度，以病人第二天感觉舒适为度。

1.2 宜大不宜小：该病作为一种慢性软组织损伤损伤牵涉的范围较广，不但牵涉局部组织的损伤，同时往往影响到邻近的关节和肌肉，所以手法的使用范围宜大，但治疗时仍需分清主次，既抓主要矛盾，又兼顾次要矛盾。如使用推、按、揉捏等手法。

1.3 宜深不宜浅：陈旧性踝关节扭伤的病机为：损伤日久，损伤局部气血运行不畅，筋脉失养。同时常伴发组织粘连、硬结、条索状物和筋骨的微小错位。一般选用斜向作用力或对称作用力治疗，损伤部位较深，手法宜重不宜轻，作用力要直达病所。常用手法有推、拨、点、按、拔伸法（拉法）等。手法的施用过程中还注意邻近关节和组织的损伤治疗。

2. 詹氏正骨手法治疗陈旧性踝关节扭伤

根据上述选用及使用原则，詹氏正骨手法在治疗陈旧性踝关节扭伤中选取揉捏、按、拨、推、拔伸、摇晃等手法进行治疗。手法施用过程如下：

2.1 舒筋。遵循詹氏正骨手法使用原则中的宜大不宜小、宜深不宜浅的原则，使用揉捏放松小腿及其周围肌肉。使用按压、拨推等手法，松解因损伤日久形成

中国知网 https://www.cnki.net

的肌肉韧带粘连和硬结。

2.2 点按相关穴位。根据推拿选穴[15,16]的原则：

(1)近部选穴

近部选穴是以腧穴的近治作用为依据就近取穴。陈旧性踝关节扭伤选取踝关节周围的解溪、昆仑、悬钟、丘墟、申脉等穴，进行穴位按摩。解溪主治脚踝痛及下肢萎痹；昆仑穴，主治足踝肿痛；悬钟又称绝骨，是八会穴之髓会，常与足三里、申脉、公孙等配伍使用主治脚弱无力；丘墟穴为胆经原穴，主治下肢萎痹，外踝肿痛等；申脉为足太阳阳跷脉之会，有补阳益气，疏导水湿的作用，可治疗足踝关节痛[17]。

(2)远部选穴

远部选穴是指在离病变部位较远的部位选取腧穴。通常选取肘膝关节以下的五腧穴，取其对本经循行所经过的远部组织有远治的作用[18]。陈旧性踝关节扭伤选取膝关节周围的五腧穴：阳陵泉、足三里等穴位进行治疗。阳陵泉又名筋会，为胆经的合穴和下合穴，为筋之会穴,主治下肢萎痹，是治疗筋病的要穴；足三里为足阳明胃经之合穴，是五腧穴之一，阳明筋脉总会于宗筋，宗筋具有约束关节和滑利关节的作用；中医有“治萎独取阳明”的说法[19]。

(3)对症取穴

对症取穴是根据疾病的特殊症状而选取穴位的原则。如陈旧性踝关节扭伤后常存在关节酸痛重着，乏力；选取足三里、阳陵泉、丰隆、承山等穴，丰隆穴为足阳明络穴，主治下肢萎痹[17]。承山是一个有效的解乏穴，可舒筋活络、壮筋补虚。

2.3 按摩足背及足底。詹氏正骨手法善于创新，遵循中医整体观，注重局部与整体结合，认为足部和踝关节是一个统一的整体，认为踝关节损伤或多或少都会伴有足部关节和组织的损伤或关节和肌腱的微小错位。通过指推法和分推法起到舒筋通络，整形复位的作用。

2.4 牵引拨伸运踝。在依次牵拉足五趾后，在左手夹持踝关节的同时，右手在牵引拔伸下作顺时针运踝，然后在跖屈踝关节到最大范围下，再背伸踝关节至最大范围。最后放松足部及小腿周围肌肉。进一步使踝关节各筋骨各归其位，司其职，以便恢复关节的正常功能。

根据詹氏正骨手法使用原则中的因人因病制宜原则，整个手法治疗过程的施

中国知网 https://www.cnki.net

治时间 15 分钟左右，频率为每日一次。

3. 詹氏正骨手法治疗陈旧性踝关节扭伤的的优点优势

3.1 讲究力与技巧的紧密配合

詹氏正骨手法根据病变部位不同在使用过程选择作用力方向不同。其目的是使作用力直达病所，从而达到治疗的目的。其中斜向用力在詹氏正骨手法中使用较多。陈旧性踝关节扭伤发生筋骨错位的部位往往不易显露，詹氏正骨手法常使用斜向用力，使力能作用于病变部位。同时通过牵引拔伸、抖摇等达到整复筋骨错缝的目的。

3.2 注重中医整体观

足部和踝关节作为一个统一的整体，踝关节扭伤往往涉及到足部软组织及关节的损伤。足部解剖较为复杂，有 26 块骨，形成众多的关节。同时足部足弓还分为三个弓即内、外侧纵弓及内外侧方向的一个横弓[20]。涉及的肌肉及肌腱也较多。踝关节扭伤后易造成足部软组织损伤及相关关节正常解剖结构的变化。查体时通过“触摸”足部各关节和肌肉韧带位置与健侧对比，发现陈旧性踝关节扭伤经常有筋不顺、骨不平的现象存在，即所谓的筋骨错位，弥补了现代医学 X 线片无法显示的不足。治疗上根据中医整体观原则，认为治疗踝关节扭伤应足踝并重，在治疗踝关节的同时，注意足部损伤的治疗。使用指推法、分推法及拨伸等手法对足背部及足底进行治疗，从而改善损伤软组织的血循，使错位的筋骨恢复正常的解剖结构。同时根据踝关节扭伤的受伤机制不同，采用不同的手法，逆其道而行。使筋骨各归其位，各司其职。达到治病求本的作用。

（二）詹氏正骨手法在其他筋伤疾病中的应用

詹氏正骨手法治疗筋伤疾病，在长期的临床实践过程中形成了其一套完整的诊断和治疗操作规范，具有自身的特点及优势，在手法的原理，选用原则和治疗原则上都有其独特的认识。

1. 詹氏正骨手法治疗筋伤疾病治疗前准备

1.1 病史的询问

一般在疾病治疗前，需要详细询问病人的病史，职业，既往有无外伤史，有无劳损史，有无其他疾病，之前有无进行过治疗，诊断为什么，怎么治疗，治疗的效果，疾病发生前有无特殊饮食，病情的变化与天气变化有无关系，与运动量有无关系，损伤后有无过早负重史等等。在治疗过程中，通过不时的查体和医患

中国知网 https://www.cnki.net

的沟通，追问完善相关病史及其病人治疗前后病情的变化情况。从而最终确认疾病的诊断和发生机制，治疗的效果如何，指导治疗方案的确立和调整。

1.2 查体

在查体时注重局部与整体相结合，避免“头痛医头，脚痛医脚“的发生，兼顾损伤相邻关节和周围组织的损伤情况，注重整体观，达到治病求本，标本兼治的目的。如陈旧性踝关节扭伤的患者，在常规查体的基础上，同时检查足部有无压痛，有无筋不顺，骨不平，负重有无疼痛，活动有无受限。注重健侧肢体与患侧肢体的对比，尤其讲究筋伤视、触、叩、听检查里面的触诊。除了踝关节的触诊检查，同时注重足部的触诊检查。由近及远、从内向外的触摸跗骨间关节、跗跖关节、跖骨间关节和趾骨间关节，在触摸到感觉有筋不顺、骨不平的地方，与对侧的相应关节对比，从而诊断出足部有无筋骨错缝，弥补了筋骨错缝时 X 线无法显示的缺陷。詹氏正骨手法在长期的治疗陈旧性踝关节扭伤的临床实践过程中发现内侧的跗骨间关节及外侧的跗跖关节处在踝关节扭伤时容易发生筋骨错缝。

1.3 辅助检查

同时借助辅助检查的方法，协助疾病诊断。如陈旧性踝关节扭伤，在查体的同时，注重与影像学检查相结合。常规 X 线片，或根据受伤机制拍摄应力下 X 线片，症状严重者配合 MRI 检查。借助血常规、风湿三项和 CRP 检查，排除痛风或踝关节感染性疾病等可能。

1.4 确认诊断

根据病史、查体及辅助检查，确认疾病的诊断。

1.5 建立治疗处方

治疗处方的建立需根据疾病的诊断、患者的基本情况和体质的强弱等最终确定。

2. 詹氏正骨手法治疗筋伤疾病的特点

在筋伤疾病治疗中，往往采用詹氏正骨手法治疗，同时配合中药内服外用。达到动静结合，标本兼治的作用。骨伤与筋伤的关系是“骨伤必定有筋伤，筋伤未必有骨伤”；詹氏正骨手法在筋伤治疗中遵循中医的整体观，讲究“筋骨并重”的治疗原则[9]。陈旧性踝关节扭伤作为一种慢性筋伤疾病，其治疗过程中也遵循这一原则。

中国知网 https://www.cnki.net

2.1 讲究手法的应用技巧

詹氏正骨手法使用强度的大小，持续的时间和频率安排与患者体质的强弱、病程的长短、疾病发生的病因病机直接相关。患者体质弱、疾病病程短、发病原因由外伤等因素引起的，手法使用宜轻，主张以轻柔手法为主，手法常以摩法、揉法为主，注意不宜过早使用手法，一般在损伤后 24 到 48 小时使用，以免加重局部软组织的损伤，持续时间一般以 10 分钟左右为宜。若患者体质强，疾病病程长，以慢性损伤为主者，手法使用应针对局部损伤部位的治疗，同时兼顾相邻的关节和组织损伤的治疗，此时手法强度要求宜重不宜轻，但同时要兼顾患者的承受能力施行，以病人能承受为度。常使用揉捏、推、拨、牵引拔伸等手法治疗，治疗时间以 15 分钟左右为宜。若患者体质弱，疾病病程长，以慢性劳损为主的损伤，需使用温养的手法进行治疗，改善损伤局部的血液循环，手法以温补手法为主，即操作时应舒缓，持久，以病人舒适为度，切忌使用过重过快的手法，以免加重病情，使旧伤再添新伤，治疗时间以 20 分钟左右为宜。

2.2 善于创新

詹氏正骨手法在手法的使用上灵活多变，要求在掌握各种基本手法的操作要领和手法熟练的基础上，认为只要能达病所解决病痛的手法就是好手法，同时把中医的整体观、辨证论治很好的应用于各种疾病的诊断和治疗过程中。认为足部和踝关节作为一个统一的整体，踝关节扭伤往往涉及到足部软组织及关节的损伤。在手法使用上注重足部手法的使用，使因踝关节扭伤引起的筋出槽，骨错缝得以各归其位，行其职。

3. 詹氏正骨手法治疗筋伤疾病的作用原理

詹氏正骨手法在手法的使用过程中，手法的选择与疾病的病因病机密切相关。其作用原理是运用不同的手法，通过使用力的大小不同、方向不同、强度不同，引起损伤部位组织的不同形变，通过改变损伤组织的形变使组织恢复正常形态和功能，达到人体运动力学结构的新平衡。故手法运用的正确与否与治疗的结果直接相关。在使用上注重力与技巧的相互配合，手法要求“刚柔相济，柔中有刚，刚中有柔”，“动静结合，筋骨并重”。同时不同的损伤外力作用于组织，可以引起组织的不同损伤形变[21]。詹氏正骨手法是根据损伤组织的不同形变及其局部特有的解剖结构来确定具体使用手法作用力的大小、方向及强度。如使用斜向用力作用于筋骨错缝的部位。轻度及急性损伤，手法选择一般以轻柔为主，作用

中国知网 https://www.cnki.net

力方向一般选用平面用力起到舒筋活络，缓解痉挛的作用[22]；对于常见的软组织扭伤，手法应轻重适中；对慢性损伤引起的粘连、硬结、条索，则需选用垂直用力的手法，使用按、拨等手法，用力宜偏重，以达到剥离粘连、解痉止痛的作用[23]。对于因损伤导致的筋骨错位，往往选择使用斜向用力，使作用力直达病所，再使用拔伸、摇晃等手法，以达到顺筋正骨、整形复位的作用[24]。

4. 詹氏正骨手法治疗筋伤疾病的选用原则

4.1 急性筋伤：一般选用比较轻柔的手法，作用力以水平用力为主。手法要求宜轻不宜重，手法使用过程中的强度一般由轻到重再到轻。如先抚摸，后揉法，最后摩法。筋伤早期不主张过早使用手法治疗，以免加重组织的损伤，影响组织的修复。施术一般建议在伤后 24-48 小时以后。

4.2 慢性筋伤：往往为急性筋伤治疗不当或处理不及时所致，损伤日久，损伤局部气血运行不畅，筋脉失养。同时常伴发组织粘连、硬结、条索状物和筋骨的微小错位。一般选用斜向作用力或对称作用力治疗，手法宜重不宜轻，常用手法有推、按、拔伸法（拉法）等。手法的施用过程中还注意邻近关节和组织的损伤治疗。

4.3 疲劳性损伤：需使用温养的手法进行治疗，改善损伤局部的血液循环，手法以温补手法为主，即操作时应舒缓，持久，以病人舒适为度，切忌使用过重过快的手法，以免加重病情，使旧伤再添新伤。

5. 詹氏正骨手法治疗筋伤疾病的使用原则

詹氏正骨手法的使用原则可归纳为“六宜四不宜”。

5.1 因人制宜：手法可以使用于不同年龄，詹氏正骨手法在使用过程中，要根据病人的性别、年龄、体质的强弱来选择手法的作用力大小、强度、持续时间和频率。一般体质弱的手法力度宜轻，持续时间宜短一般 10-15 分钟每次，频率可每日一次或隔日一次。同时观察病人的反应，强度以病人能够耐受为度，以病人第二天感觉舒适为度。

5.2 因病制宜：根据疾病的病因病机和疾病的病程长短，来进行手法选择和确定手法的强度、持续时间和频率。急性病一般选用轻柔的手法，持续时间每次 10 分钟左右为宜，慢性病一般选用深透性较强的手法，每次 15 分钟左右为宜，根据情况可适当延长。劳损类疾病一般选用温养的手法一般以 20 分钟每次为宜。

5.3 宜小不宜大：局部损伤较轻，一般使用以局部取穴按摩的手法主，确保

中国知网 https://www.cnki.net

手法持续作用的时间，尽快的收到疗效。常用的手法有点、按、揉法等。

5.4 宜大不宜小：损伤牵涉的范围较广的慢性软组织损伤，不但牵涉局部组织的损伤，同时往往影响到邻近的关节和肌肉，所以手法的使用范围宜大，但治疗时仍需分清主次，既抓主要矛盾，又兼顾次要矛盾。常用的有推、按、揉捏等。

5.5 宜深不宜浅：若损伤部位较深，使用手法进行治疗时为达到治疗的效果，要求手法宜沉不宜浮，作用力要直达病所。常用手法有弹、拨、点、按等。

5.6 宜浅不宜深：若损伤后疼痛的部位比较表浅，手法使用则宜浅不宜深，宜柔不宜刚。以免造成深部组织新的损伤发生。常用手法有摩法、檫法等。

詹氏正骨手法治疗筋伤疾病就是根据病人体质的强弱，疾病的类型及病因病机，遵循上述选用原则和使用原则，选择合适的手法、合适的强度、合适的持续时间、合适的频率来治疗各种筋伤疾病的。

(三)中药熏药及选方依据

1. 传统医学对中药熏药治疗的理论认识[25]

1.1 发汗透表、调节阴阳

《素问》“阳气怫郁在表当解当熏之”，认为对于外邪袭表导致阳气郁闭于内，可熏蒸先治其外。张锡纯“ 出汗之道，在调其阴阳”，指出了熏蒸治疗通过发汗透表达到调节机体阴阳平衡的作用。

1.2 活血化瘀、散寒止痛

《素问.至真要大论篇》中提出“寒者热之”及“寒胜则痛”，指出疼痛多因寒邪侵犯机体所致；另《素问.痹论》言“凡痹之类，逢寒则虫，適热则纵”，其中“痹者，闭也”为气血感受寒凉，导致气血流通运行不畅所致。

1.3 调补气血

中药熏药药方中常配伍以补气活血的药物，同时通过蒸汽的温热作用，能很好的有助于损伤部位的气血流通，调补卫阳不足。“外治者，气血流通即是补”，中药熏蒸治疗通过使气血流通达到调补卫阳之不足 ，和《内经》“损者温之”之意相通，对于气血不足者熏蒸具有调补气血的作用 。

2. 选方依据

陈旧性踝关节扭伤根据中医辨证属于筋脉失养型。本研究选方来源于《中医伤科讲义》的经验方“四肢损伤洗方”，方中桑枝与桂枝合用，温经散寒、通络止痛、祛风除痹效佳，常用于各种风寒湿痹证的治疗；伸筋草、透骨草合用进一

中国知网 https://www.cnki.net

步增强散寒活血止痛之功；牛膝善于补肝肾、强筋骨同时能引血下行，常用于下肢痹证的治疗；木瓜善治拘痉挛，具有舒筋活络之效；乳香、没药合用，为宣通脏腑、流通经络之要药，为止痛之专药，治疗痹证多用；红花常用于治疗各种跌打损伤，为瘀血肿痛之要药；羌活、独活配伍具有较强的祛风散寒，胜湿止痛的功效，两者在治疗痹证上分别善于上肢和下肢，两者配伍使用可治疗各种四肢痹证；落得打：清热利湿，解毒消肿；萆薢祛风湿；利湿浊；补骨脂、淫羊藿均具有温肾助阳之功治疗寒痹结合补阳，遵循“阳气足则阴寒自散”的治疗原则[26-28]。诸药配合共同起到温经通络、活血祛风的作用，用于四肢骨折、脱位及各种扭挫伤后筋络挛缩酸痛等。

中国知网 https://www.cnki.net

结 论

第一，本临床研究表明，两种方法治疗陈旧性踝关节均有效果，詹氏正骨手法配合中药薰药治疗陈旧性踝关节扭伤能明显改善受试者的临床症状、局部压痛指标、疼痛指标、功能活动度指标，疗效优于单纯中药薰药治疗。詹氏正骨手法配合中药薰药治疗陈旧性踝关节扭伤比单纯中药薰药治疗陈旧性踝关节扭伤效果更好，疗效更佳，值得推广使用。

第二，詹氏正骨手法在筋伤疾病治疗过程中，在传统手法的基础上，形成了自己的独有特色，注重查体和治疗过程中局部与整体的结合。认为治疗疾病是一个过程，而这一过程不是一层不变的，它随着治疗的变化而变化，治疗的主次也会发生变化，要求手法的治疗在总的手法选择好之后，在施治时也要不断进行调整；同时配合中药薰药治疗能更好的的达到治疗效果。

中国知网 https://www.cnki.net

参考文献

[1]王智勇,吴启六.正骨手法治疗踝关节扭伤 80 例报告[J].中国中医骨伤科杂志,2006,14(5):57.

[2] 欧云娜,张珑琼,秦乐等.浅谈痹证从瘀辨治[J].湖南中医杂志,2014,5(30):116-117.

[3]Sanjay Mattam,Panduranga Rao KR,Chinthaparthi Mallikarjuna Reddy.non operative treatment of chronic ankle sprain:a study conducted in teaching general hospital,Telangana,India[J].International Journal of Research in Medical Sciences,2015,3(3): 635-639.

[4]Wolf Petersen,Ingo Volker Rembitzki,Andreas Gsele Koppenburg,et al.treatment of acute ankle ligament injuries:a systematic review[J].Archives Of Orthopaedic And Traumatic Surgery,2013,133(8):1129-1141.

[5]踝关节扭伤中医诊疗方案,24 个专业 105 个病种中医诊疗方案（试行）[S].国家中医药管理局医政司,2011:143-144.

[6]彭力平.实用骨伤科手册[M].湖南:湖南科技出版社,2009:10.

[7]Crichton KJ,Fricker PA,Purdam C,et al.Injuries to the pelvis and lower limb.In Bloomfield J,Fricker PA and Fitch KD.Science and Medicine in Sport 2nd ed[J].Blackwell Science Pty Ltd,Victoria,1995:463-467.

[8] 许涛.踝关节外侧韧带与踝间后韧带的解剖学与生物力学研究[D] 吉林大学.2009:6.

[9]田佳.中医筋伤学[M].成都:成都体育学院教材审定委员会,1996:1,29,319.

[10]程志安,骆惠明,谢水安等.227 例急性腰扭伤临床分析与手法治疗疗效评价[J].中国中医骨伤科杂志,2007,15(12):57-59.

[11]曹卉娟，邢建民，刘建平.视觉模拟评分法在症状类结局评价测量中的应用[J].中医杂志.2009(07):600-602.

[12]KOFOED H.Comparison of ankle arthroplasty and arthrodesise prospective series with long term follow-up[J].Foot,1994,(4):6-9.

[13]国家中医药管理局.中医病证诊断疗效标准[S].南京:南京大学出版社,1994:186-187.

中国知网 https://www.cnki.net

[14]林建达. 浅谈推拿治疗踝关节扭伤[J]. 按摩与导引,2007,23(5):32-33.

[15]魏连海,王卫,孟向文等.针灸处方的选穴原则和组方规律[J]天津中医药大学学报,2008,27(2):59-60.

[16]房敏,刘长信,王立新等. 推拿医疗技术[C]. 中华中医药学会推拿分会第十四次推拿学术交流会论文汇编.2013:44.

[17]梁繁荣,沈学勇.针灸学[M].中国中医药出版社,2012:45-46,71-72,95-96.

[18]石学敏.针灸学[M].中国中医药出版社,2015:23.

[19]彭支莲,黄剑,韦桂勇等.电针对兔膝骨关节炎家兔模型软骨细胞凋亡的影响[J]中国中医急症,2014,23(8):1428-1430.

[20]朱晞,人体解剖学[M]浙江大学出版社,2003:33-35.

[21]秦渭志.推拿治疗软组织损伤的机制研究[C]全国第十五届传统医学手法学术交流大会、广东省中医药学会推拿分会学术年会、广东省针灸学会手疗法医学专委会成立大会暨第一届委员选举大会论文集,2009:8-9.

[22] 尹春英,曹中兵,伊友明.浅析推拿手法治疗踝关节扭伤的机理[J]时珍国医国药,2006,17(2):283-284.

[23]蔡金林.推拿疗法应用发展浅析[J].陕西中医,2008,29(9):1214-1215.

[24]胡军飞.范炳华运动推拿手法介绍[J].浙江中医杂志,2006,41(5):294-295.

[25]陆继娣.中药熏蒸治疗关节炎及其抗炎机理的研究[D].安徽中医学院,2006:12.

[26]高学敏,中药学[M].中国中医药出版社,2012:53-499.

[27]李世峰,易洪城.活血化瘀法在骨伤科的应用[C].第五届中国整脊学学术交流大会论文集,2009,10(2):118-119

[28]贺新勇,蔡明,贾小平.闭合复位外固定配合中药治疗尺桡骨双骨折 [J].中医正骨，2004，16(6):36.

中国知网 https://www.cnki.net

附表

附表 1　VAS 疼痛评分

随机号：　　　姓名：　　　性别：　　年龄：　访视时间：　　年　月　日

备注：

VAS 疼痛评分，如图所示：即使用一根长 10cm 的不透明直尺，直尺两端“0”和“10”刻度，分别代表不痛和疼痛无法忍受。检查时医师将直尺没有刻度的一面对着病人，让病人凭主观感觉指出疼痛程度的相应部位，根据病人的指出位置进行评分。

0	1	2	3	4	5	6	7	8	9	10cm

结果：VAS 局部压痛评分 0 代表无疼痛，1-3 代表轻度疼痛，4-6 代表中度疼痛，7-10 代表重度疼痛（主观症状评分）。

中国知网　https://www.cnki.net

附表 2 Kofoed 评分量表

随机号: 姓名: 性别: 年龄: 访视时间: 年 月 日

备注:

Kofoed 评分量表:(低于 70 分为差;70-74 分为及格;75-85 分良;85-100 分为优)

(1)疼痛(满分 50 分，为基本分)		(2)功能(满分为 30 分)	
①无疼	50	①足趾行走	3
②行走开始时疼痛	40	②足跟行走	3
③行走时疼痛	35	③正常节律上下楼梯	6
④偶尔负重时都有疼痛	35	④单腿站立	6
⑤每次负重时都有疼痛	15	⑤无辅助性行走	6
⑥检查时疼痛或自发疼痛	0	⑥不用骨科足支具	6
C3)活动度(满分 20 分)			
伸$>10^0$	5	旋后$>30^0$	3
$5-9^0$	3	$15-29^0$	2
$<5^0$	1	$<15^0$	1
屈$>30^0$	5	旋前$>20^0$	3
$15-29^0$	3	$10-19^0$	2
$<15^0$	1	$<10^0$	1
负重时外翻$<5^0$	2	负重时内翻$<3^0$	2
$5 — 10^0$	1	$4-7^0$	1
$>10^0$	0	$>7^0$	0

中国知网 https://www.cnki.net

附表 3

知情同意书同意签字页

临床研究项目名称：詹氏手法配合中药熏药治疗陈旧性踝关节扭伤的临床研究

同意声明

我已经阅读了上述有关本研究的介绍，而且有机会就此项研究与医生讨论并提出问题。我提出的所有问题都得到了满意的答复。

我知道参加本研究可能产生的风险和受益。我知晓参加研究是自愿的，我确认已有充足时间对此进行考虑，而且明白：

●我可以随时向医生咨询更多的信息。

●我可以随时退出本研究，而不会受到歧视或报复，医疗待遇与权益不会受到影响。

我同样清楚，如果我中途退出研究，特别是由于药物或手法治疗的原因使我退出研究时，我若将我的病情变化告诉医生，完成相应的体格检查和理化检查，这将对整个研究十分有利。

如果因病情变化我需要采取任何其他的药物治疗，我会在事先征求医生的意见，或在事后如实告诉医生。

我将获得一份经过签名并注明日期的知情同意书副本。

最后，我决定同意参加本项研究，并保证尽量遵从医嘱。

患者签名：　　　　　　　　　　　　年　　月　　日

联系电话：

我确认已向患者解释了本试验的详细情况，包括其权力以及可能的受益和风险，并给其一份签署过的知情同意书副本。

医生签名：　　　　　　　　　　　　年　　月　　日

医生的工作电话：

中国知网 https://www.cnki.net

致 谢

三年寒暑，转瞬即逝，当我完成学业即将告别校园之际，首先要感谢导师王人彦教授一直以来对我的悉心指导和谆谆教诲。感谢导师王人彦教授对我论文设计、构架和写作的指导。王人彦教授在学习、工作及生活中都给了我极大的关怀和支持。导师严谨的治学态度、开阔的思维、高度的敬业精神、正直豁达的人生态度给我留下了难以磨灭的印象。必将对我经后的学习生活造成良好的影响。

衷心感谢全体授课老师，感谢他们传授我关于文献检索方面的技巧、立题的技巧，以及统计学知识及其统计工具的使用。

衷心感谢浙江中医药大学尹航教授、吴连国教授在论文设计上给予的指导。

衷心感谢杭州市中医院潘浩主任、浙江省中山医院姚新苗主任、浙江省中医院卢建华主任、江干区人民医院金信良主任对论文的撰写及修改过程中的指导。

衷心感谢杭州詹氏中医骨伤医院非物质文化遗产项目小组及院方领导对本论文的支持。

衷心感谢杭州詹氏中医骨伤医院詹新宇主任、李明主任、朱建华主任、邓正平主任在课题施行期间给予的指导和帮助。

衷心感谢科室成员在病例收集及数据收集中给予的帮助。

最后，感谢亲爱的父母及亲友对我一如既往的关心与支持，你们是我一切力量的源泉和动力。

中国知网 https://www.cnki.net

文献综述

手法治疗陈旧性踝关节扭伤研究进展

摘要：踝关节扭伤是运动中最容易发生的关节部损伤。陈旧性踝关节扭伤为临床常见病，多发病，严重影响患者的日常生活。西医治疗方法单一，疗效一般。中医治疗该病方法较多，如手法、针刺、中药熏蒸、灸法、火针等，现已发现传统中医手法治疗陈旧性踝关节损伤疗效确切。从近年来文献研究中，笔者就手法治疗为主治疗陈旧性踝关节扭伤的临床进展进行总结，以期为临床治疗提供参考依据。

关键词：陈旧性踝关节扭伤，手法，临床研究，综述

1.病理基础

踝关节是人体负重最大的屈戍关节，站立时全身重量均落在踝关节上，行走时的负荷值为体重的 5 倍。踝关节扭伤是最常见的关节部位的损伤。踝关节扭伤分为急性和陈旧性，急性踝关节扭伤是骨科急诊中最常见的关节部位的损伤[1]。陈旧性踝关节扭伤多由急性踝关节扭伤疾病失治、误治或积劳成疾所致。中医理论认为：踝关节扭伤是踝关节平衡关系失调，致使筋出槽，骨错缝，不通则痛。陈旧性踝关节扭伤的病理机制为：瘀血未化，经络闭阻，气血运行不畅，导致踝关节周围软组织筋脉失养。手法推拿具有活血化瘀、消肿散结、疏通经络、滑利关节的功效。“轻巧柔和，筋骨并重，治病使患者不知其苦”是推拿手法治病的最大特点。

2.治疗方法

2.1 单纯手法治疗

踝关节的急性损伤如未及时处理或处理不彻底，常因局部纤维组织增生、粘连及关节间微小错缝，导致患踝长期肿胀、疼痛、僵硬及功能障碍。治疗重在松解粘连、滑利关节、整复错缝，以恢复其功能。手法治疗可松解病变关节周围痉挛的肌肉韧带，使踝关节卷缩、错缝、扭转的筋归其位、行其槽、司其职，同时可起到活血化瘀，消肿散结，疏通经络，疏利关节的作用，为陈旧性踝关节扭伤的传统重要治疗手段。

吴斌[2]采用俯卧位踝关节拔伸法配合理筋手法治疗陈旧性踝关节扭伤 50 例，

中国知网 https://www.cnki.net

经过 3 个疗程的治疗统计疗效，总有效率为 98%。高景华等[3]采用摇拔戳手法治疗陈旧性踝关节扭伤 34 例，治疗 3 个疗程总有效率为 91.2%。何永瑞[4]分期推拿治疗踝关节扭伤 40 例根据踝关节局部的肿胀情况，采用不同的推拿手法进行治疗。结果：治愈 39 例，好转 1 例，有效率为 100%。李俊海等[5]比较正骨手法与中药熏洗治疗陈旧性关节扭伤的临床效果。治疗组采用正骨手法即摇拔戳手法治疗，每周 2 次，对照组采用中药熏洗治疗，每日 1 次。结果治疗三周，治疗组总有效率为 87.2%，明显高于对照组的 62.1%。高波[6]使用四步手法治疗单纯性踝关节运动扭伤 92 例，方法：随机将患者分为治疗组和对照组，分别采用推拿手法与口服中华跌打丸结合正红花油外搽进行 15 天的治疗。结果：治疗组治愈率和总有效率显著高于对照组。结论：四步推拿手法通过放松、理筋、整复、整理 4 大步骤治疗，在促进踝部水肿、血肿吸收的基础上，能迅速解除损伤局部肌肉韧带痉挛，促进炎症介质分解稀释和组织修复，故对单纯性踝关节扭伤有显著疗效。宋贺卫[7]探讨三步推拿法治疗踝关节扭伤的临床效果。方法：踝关节扭伤患者 60 例根据治疗方法的不同分为治疗组与对照组各 30 例，对照组运用足背伸外翻位包扎固定治疗，治疗组采用三步推拿法治疗。结果：治疗组的总有效率为 93.3%，对照组为 73.3%。

2.2 手法配合中药熏洗治疗

陈旧性踝关节扭伤患者因瘀血内阻，经络闭阻，气血运行不畅，导致踝关节周围软组织筋脉失养。腠理不密，风寒湿邪易乘虚而入，导致气血凝滞，则肿痛难消，功能受限，不通则痛。中药熏洗由于药与热发挥作用，与手法配合治疗陈旧性踝关节扭伤，可以达到更好地活血化瘀、温经通脉、疏利关节的作用。

刘瑞钦等[8]观察推拿联合中药熏洗治疗陈旧性踝关节扭伤疗效，对照组采用局部痛点封闭，治疗组采用推拿即揉捏弹拨、按揉周围肌肉韧带后，在拨伸下做踝关节屈伸，内外翻及环转运动。同时配合中药熏洗，熏洗处方组成为：伸筋草 50 克，延胡索、白芷各 30 克，威灵仙、当归、鸡血藤、丹参各 50 克，乳香、没药、透骨草、木瓜各 30 克。经治疗治疗组总有效率为 96.32%，对照组总有效率为 33.25%。冯前等[9]推拿配合中药药熏治疗踝关节扭伤 81 例，在采用推拿手法放松周围肌肉韧带的基础上，再采用牵拉矫正的手法，达到纠正关节错缝，滑利关节，增强关节活动度的作用。配合使用中药熏洗，中药熏洗方组成：杜仲 20 克、川芎 20 克、伸筋草 20 克、透骨草 20 克、丹参 20 克、防风 20 克、威灵

中国知网 https://www.cnki.net

仙 15 克、延胡索 20 克、桑寄生 20 克、桂枝 20 克、红花 10 克。结果临床治愈 62 例，占 76.5%，好转 17 例，占 20.9%，无效 2 例，占 2.4%，总有效率为 97.5%。郭峪城[10]中药熏洗联合推拿治疗踝韧带扭伤 64 例，对照组采用舒筋通络，理筋整复手法治疗，治疗组在使用舒筋通络，理筋整复手法配合使用中药熏洗，熏洗方组成为川椒 30 克、归尾 15 克、红花 15 克、伸筋草 15 克、透骨草 15 克、三棱 15 克、莪术 15 克、川乌 15 克、草乌 15 克、威灵仙 15 克、海桐皮 15 克、土茯苓 25 克。并嘱患者自行功能锻炼。对照组总有效率 71.88%，治疗组总有效率为 90.63%。谢君等[11]观察温养手法推拿结合中药熏洗治疗陈旧性踝关节扭伤 30 例，选择拿、点、揉、搓等舒筋手法，辅以分筋、摇扳关节等运筋手法。对治疗部位及穴位选择，首先从整体上选择总督一身阳气的督脉以及膀胱经上腧穴进行滚揉，其次选择局部的丘墟、申脉、昆仑穴和关节周围的解溪、太溪、照海等穴，以及关节所布经络临近的承山、足三里、三阴交、 阳陵泉、悬钟等穴位，外用中药自拟温经舒筋汤熏洗，处方：艾叶 100g，川芎 30g，独活 30g，木瓜 30g，伸筋草 30g，舒筋草 30g，松节 50g，怀牛膝 30g。30 例中优 20 例，良 7 例，一般 2 例．差 1 例，优良率为 90.0%。刘海全[12]调经筋手法配合中药熏洗治疗陈旧性踝关节扭伤 72 例临床观察，均选用解溪穴、丘墟穴、阳陵泉穴作为基础穴位，施以轻手法按摩，按摩以患者感觉酸胀为度。按损伤部位及临床症状，根据足部经筋走行调整相应经筋。踝外侧者调整足太阳、足少阳经筋；踝前者调整足少阳、足阳明经筋；踝内侧者调整足太阴、足少阴和足厥阴经筋。以及经筋取穴手法配合自拟舒筋活络汤熏洗，观察治疗 1 疗程后的疗效情况。参照 Baird-jackson 踝关节评分系统，优 15 例，良 38 例，可 37 例，差 2 例，优良率 73.6%。

2.3 手法配合针刺治疗

针刺对人体的整体功能与局部功能均具有良好的调节作用，针刺可舒筋通络、行气活血使局部血液循环改善，肿痛消失，修复组织[13]；手法整复是通过扳、摇、牵伸、关节松动等改善踝关节周围血液循环，加速扭伤后血肿及渗出液的吸收，解除肌肉痉挛疼痛，防止软组织粘连，预防关节僵硬[14]；配合主动功能锻炼，可避免因瘀肿的消退而产生粘连的发生，并加速病程的痊愈。

王傅等[15]观察针刺配合手法整复治疗踝关节扭伤的疗效，方法:85 例踝关节扭伤患者分为综合组 53 例和对照组 32 例，均采用针刺治疗，综合组同时配合手法整复治疗。结果:治疗 20 天后，综合组治愈率明显优于对照组(分别为 69.8%、

中国知网 https://www.cnki.net

40.6%, P<0.05)。结论:针刺配合手法整复治疗踝关节扭伤的疗效优于单一的治疗方法。针刺取穴（解溪、丘墟、商丘）和阿是穴。祝增奇等[16]使用针刺配合手法整复治疗踝关节扭伤的疗效研究，观察组和对照组均给予针刺，观察组同时使用手法整复，结果：观察组治愈率 70%，总有效率 97.5%。对照组治愈率 40.5%，总有效率 92.9%。曹均[17]针刺配合推拿手法治疗踝关节扭伤 36 例，将 72 例患者随机分为对照组和治疗组各 36 例，对照组采用传统针刺治疗。治疗组在对照组基础上配合推拿手法治疗。治疗 2 周后比较两组患者症状体征积分及临床疗效。结果治疗组患者踝关节评分及总有效率明显高于对照组。针刺方法采用传统针刺治疗，取患侧阳陵泉、昆仑、解溪、足临泣等穴。李博识[18]手法整复配合针刺治疗踝关节扭伤 40 例，治疗方法：先手法整复，首先手法对抗牵引 1-2min，以松解粘连的软组织，，缓缓背屈、跖屈及内翻外翻踝关节。然后进行复位手法治疗。针刺治疗选穴：外踝扭伤取阳陵泉、悬钟、丘虚、昆仑、足临泣、阿是穴；内踝扭伤取三阴交、太溪、太白(均取患侧)。40 例患者经 1 个疗程治疗后，治愈 32 例占 80%；好转 8 例占 20%；总有效率为 100%。

2.4 手法配合其他方法治疗

刘保新等[19]观察小针刀疗法配合运动理筋疗法治疗陈旧性踝关节扭伤的临床疗效。选取陈旧性踝关节扭伤患者 70 例，随机分为治疗组和对照组，每组 35 例，治疗组采用小针刀+运动理筋疗法治疗，对照组采用小针刀+局部推拿理筋治疗。治疗 4 周后，治疗组复发情况及进一步的恢复情况优于对照组。刘克龙[20]拔罐配合推拿治疗踝关节扭伤 78 例，瘀肿局部消毒，再用消毒用三棱针对准瘀肿局部快速散刺出血，用火罐拔于针刺处。后采用药酒或按摩乳涂于患处，予以局部点穴按摩，然后通过拔伸踝关节并作小幅度内外旋被动动作，突然被动背伸跖屈踝关节，可听到“咯噔”响声；最后按丘墟、阳陵泉酸胀为度，用绷带固定躁关节用绷带固定躁关节本组 78 例，治愈 76 例，占 97.44%;好转 2 倒， 占 2.56%有效翠为 100%。张茂亮[21]手法配合穴位注射（行苍龟探穴法并注射药液）治疗陈旧性踝关节扭伤，取得良好效果。邱华平[22]隔姜灸结合自我拉伸法治疗陈旧性踝关节扭伤 36 例。36 例患者中治愈 26 例，好转 10 例，无效 0 例，总有效率达 100%。张美花[23]观察推拿辅以超声波治疗陈旧性踝关节扭伤的效果。治疗组予以推拿手法辅助超声波治疗和护理，对照组单纯予以推拿手法治疗，治疗组总有效率 93.33%，对照组总有效率 73.33%。

中国知网 https://www.cnki.net

3. 结论

中医治疗陈旧性踝关节扭伤的方法有很多，手法治疗在陈旧性踝关节扭伤治疗中虽然取得较为满意的疗效。各种研究表明手法治疗可促进血液循环，有效减轻静脉淤滞 ，提高痛阈值，恢复踝关节正常功能，并能解除肌肉痉挛，营养肌组织，防止肌肉萎缩。单纯手法治疗踝关节损伤，具有痛苦小、副作用少、费用低、易于普及推广、患者易接受等优点，具有推广应用价值。在使用手法治疗的同时辅助以其他疗法，对缩短踝关节扭伤的病程有着一定的意义。但在其研究过程中确实也存在一些明显不足，主要表现在：第一：对踝关节扭伤的诊断标准不统一，临床研究为案例式，缺乏科学严谨性对照，疗效评价标准以主观性为多，缺乏客观性评价标准。第二：大多研究对于踝关节扭伤的病例的选择标准不够明确，对不同损伤时期和损伤程度的踝关节扭伤手法的使用没有进行规范，即对手法的规范化使用及诊疗规范尚不十分明确。如使用什么手法，手法使用频次及强度都没有进行规范。操作步骤缺乏规范。我们应在研究使用中医手法治疗踝关节扭伤疗效的同时，研究中医手法治疗机理，建立手法治疗踝关节扭伤的技术操作规范。

参考文献

[1]Susan R R,Donald A,Deanna K K, et al.Nutrition support in the intensive care unit [J].Nutr Crit Care.2005,23(6):49-57.

[2]吴斌.俯卧位踝关节拔伸法配合理筋手法治疗陈旧性踝关节扭伤 50 例[J]. 中国高等医学教育,2015,(1):131.

[3]高景华,高春雨,孙树椿等.摇拔戳手法治疗陈旧性踝关节扭伤 34 例[J].世界中医药,2011,3(6):214-215.

[4]何永瑞.分期推拿治疗踝关节扭伤 40 例[J].上海中医药杂志,2002,(9):39.

[5]李俊海,王庆甫,黄沪.正骨手法与中药熏洗治疗陈旧性踝关节扭伤的病例对照研究[J].中国骨伤,2012,25(2):113-115.

[6]高波.四步手法治疗单纯性踝关节运动扭伤 92 例[J].陕西中医，2014，35(7):873.

[7]宋卫贺.三步推拿法治疗踝关节扭伤的临床分析[J].中国现代药物应用，

中国知网 https://www.cnki.net

2013,7(23):222-223.
[8]刘瑞钦,张光亚,张红纪.推拿联合中药熏洗治疗陈旧性踝关节扭伤随机平行对照研究[J].实用中医内科杂志,2014,28(8):25-27.
[9]冯前，梁恒晔.骆红剑推拿配合中药药熏治疗踝关节扭伤 81 例[J],中国中医骨伤科杂志,2012,20(9):59-60.
[10] 郭峪城 . 中药熏洗联合推拿治疗踝韧带扭伤 66 例 [J], 光明中医,2015,30(1):117-118.
[11]谢君,游富贵.温养手法推拿结合中药熏洗治疗陈旧性踝关节扭伤 30 例[J]. 国医论坛,2013,28(1):25-26.
[12]刘海全.调经筋手法配合中药熏洗治疗陈旧性踝关节扭伤 72 例临床观察[J].新中医,2009,41(12):62-63.
[13]王俊华,付立勇,郑明双等.物理疗法配合消瘀止痛膏治疗急性踝关节扭伤[J].中国康复,2001,16(4):230-230.
[14]南登昆.物理治疗手册[M].北京:人民军医出版社,2001.
[15]王傅,高春华,陈丽珍等.针刺配合手法整复治疗踝关节扭伤[J].中国康复.2006,21(4):262.
[16]祝增奇,邓德.针刺配合手法整复治疗踝关节扭伤的疗效研究[J].青海医药杂志,2013,43(4):73-74.
[17]曹均.针刺配合推拿治疗踝关节扭伤 36 例临床研究[J].亚太传统医药，2015,11(14):76-77.
[18]李博识.手法整复配合针刺治疗踝关节扭伤临床体会[J].中华中西医学杂志2009,7(9):20.
[19]刘保新,关俊辉,蔡迎峰等.小针刀配合运动理筋疗法治疗陈旧性踝关节扭伤的临床研究[J].辽宁中医杂志,2015,42(5):1071-1073.
[20]刘克龙.拔罐配合推拿治疗踝关节扭伤 78 例[J].中医外治杂志,2002,11(2):45.
[21]张茂亮.手法配合穴位注射治疗陈旧性踝关节扭伤疗效观察[J].中医临床研究,2013,5(18):49-50.
[22]邱华平.隔姜灸结合自我拉伸法治疗陈旧性踝关节扭伤 36 例[J].浙江中医杂志,2013,48(11):851.
[23]张美花.推拿辅以超声波疗法治疗陈旧性踝关节扭伤的疗效观察及护理[J].

中国知网 https://www.cnki.net

内蒙古中医药,2014,(5):44-45.

中国知网 https://www.cnki.net

詹氏正骨手法配合中药熏药治疗陈旧性踝关节扭伤

技术操作规范

踝关节扭伤为骨科门诊常见病、多发病，可见于各个年龄，尤以青壮年多见。由于踝关节韧带损伤早期在X线片上往往不能显示，导致患者和医生对其给予的重视不够，处理不及时或治疗不恰当，最终发展为关节反复肿胀疼痛，关节僵硬，活动不利甚者功能障碍，即发展为陈旧性踝关节扭伤。中医认为陈旧性踝关节扭伤的病理机制为：瘀血未化，经络闭阻，气血运行不畅，导致踝关节周围软组织筋脉失养[1]。以关节反复肿胀、疼痛、关节功能障碍为主要表现。本病近年来随着人们健康意识的不断提高，参加各种体育运动和锻炼人数的逐年上升，其发病率也有所增高，已经给患者的生活和工作带来严重影响。本病属于中医“筋伤”和“痹症”的范畴，也称为“踝缝伤筋”。

一、西医诊断

（一）西医诊断标准

参照《实用骨伤科手册》中关于陈旧性踝关节扭伤诊断标准[2]

(1)有明显外伤史；

(2)踝关节肿胀、酸痛乏力，关节活动时可有摩擦感，久行、阴雨天时加重；

(3)外踝前下方及内踝前外侧有肿胀、压痛，内翻、屈伸时活动可受限；

(4)检查未发现骨折和脱位，X线摄片检查未见骨折；

(5)病程超过20天未愈者；

注：综合临床、实验室及X线检查，符合1、2、5条或1、3、5条或1、3、4、5条或1、2、4、5条，可诊断为陈旧性踝关节扭伤。

（二）疾病损伤分类[2]

结合骨伤理论、外伤机制和临床四诊表现，可分为：

(1)内翻损伤：此型临床最多见，这是与踝关节的解剖特点有关。维持踝关节内侧稳定的三角韧带远比维持踝关节外侧稳定的跟腓韧带、距腓前韧带、距腓后韧带结实的多，而且外踝要比内踝长1-2cm。受伤时踝关节极度内翻，踝关节外侧疼痛、肿胀、皮下青紫，外踝前缘、下缘压痛明显，踝关节活动受限，X线片有时可见到外踝尖处有小骨片撕脱。

(2)外翻损伤：踝关节极度外翻位损伤，踝关节内侧处疼痛、肿胀、皮下青

中国知网 https://www.cnki.net

紫，内踝周围压痛明显，踝关节活动受限，X 线片踝关节多无异常，有时需要加照外翻应力位片。

（三）踝关节韧带损伤分度[3-4]：

Ⅰ度损伤：韧带拉伤，关节无不稳，韧带有所松弛；

Ⅱ度损伤：韧带部分断裂，轻度不稳：

Ⅲ度损伤：韧带完全断裂，明显不稳，一般的 X 光片可发现骨折。

本研究对象为Ⅰ度损伤损患者。

二、中医诊断

（一）中医诊断标准[5]

参照中华人民共和国中医药行业标准 《中医病证诊断疗效标准》制定 ：

①有明确的踝部扭伤史 ；

②扭伤时间在 1 个月以上 ；

③踝关节疼痛、无力，不能久行，影响生活、工作和运动；

④内踝或外髁前下方处可有不同程度的肿胀和压痛，或可触及痛性“筋节”；

⑤X 线片未见骨折和脱位。

(二)中医辩证分型[6]

参照 2011 国家中医药管理局制定的踝关节扭伤中医诊疗方案，中医辩证分型为

(1)血瘀气滞证：损伤早期，踝关节疼痛，活动时加剧，局部明显肿胀及皮下瘀斑，关节活动受限。舌红边瘀点，脉弦。

(2)筋脉失养证：损伤后期，关节持续隐痛，轻度肿胀，或可触及硬结，步行欠力。舌淡，苔白，脉弦细。

其中，陈旧性踝关节扭伤属于筋脉失养型。

三、适应症和禁忌症

(一）适应症：

符合上述中西医诊断，中医诊断中的筋脉失养型，踝关节韧带Ⅰ度损伤患者，有踝关节疼痛和活动受限的症状。

(二） 禁忌症：

1. 严重心肝肾重要脏器疾患；药物过敏者；孕妇及哺乳期妇女；

2. 皮肤病，感染者，发热病人，血小板功能不全者；

中国知网 https://www.cnki.net

3. 结核病，恶性肿瘤患者等不适合小针刀治疗患者；

4. 糖尿病患者；

5. 局部皮肤软组织破损或有伤口感染者。

四、操作步骤和技术要领

在门诊使用詹氏正骨手法配合中药熏药治疗陈旧性踝关节扭伤，每日 1 次，7 次为 1 疗程，治疗周期为 2 疗程。2 疗程治疗后，仍继续观察，定期追踪随访三个月。

1、中药熏药方法：

(1)准备物品：

中熏药处方使用来源于《中医伤科讲义》的经验方“四肢损伤洗方”，组成为原方，剂量由作者自行设定。组方为：桑枝、桂枝、伸筋草、透骨草各 25 克，怀牛膝、木瓜各 15 克，乳香、没药、红花各 30 克，羌活、独活、落得打各 20 克，补骨脂、淫羊藿、萆薢各 30 克。以上各药切成片，混合均匀，分装成袋，每袋 125 克。每袋中药，煎煮为 150 毫升药液装为一袋备用。见图 1。

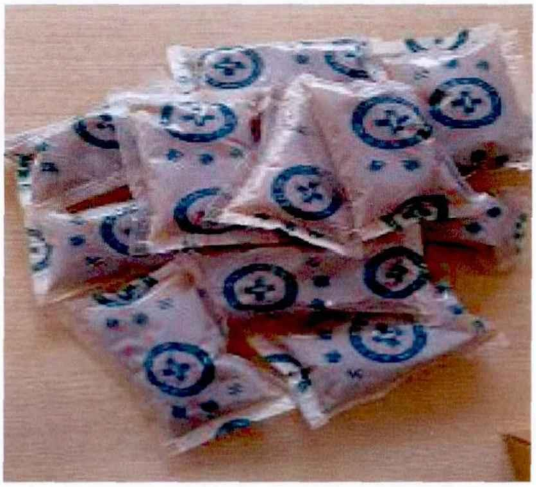

图 1

中国知网 https://www.cnki.net

2 仪器：XZQ-III型中药熏蒸器，产地：常州。图 2，图 3。

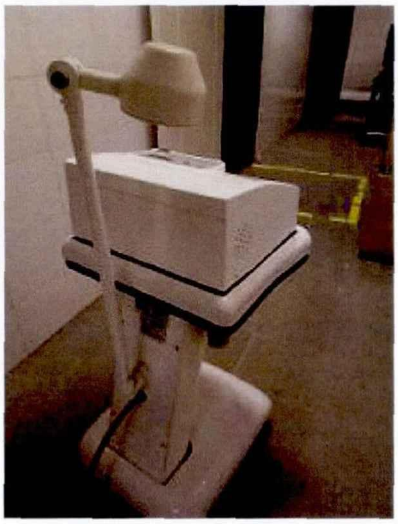

图 2

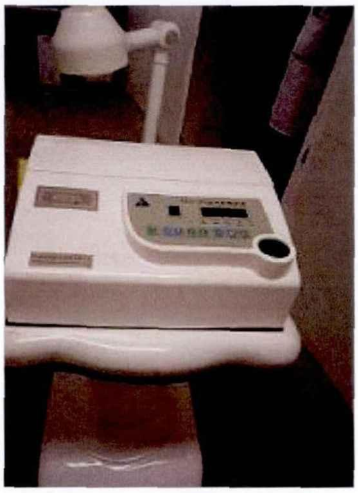

图 3

3）熏蒸方法：先加适量的温水，再将煎好的药液一袋（150 毫升）加入治疗仪器的容器内，接通电源，打开总开关，根据要求在控制面板上设定各参数，温度设定在 38-42 度之间，时间为 20 分钟，加温到指定温度后，嘱患者患踝充分暴露，并检查治疗部位皮肤有无破损。操作床上垫一次性中单，嘱患者侧卧于操作床上，若外踝损伤健侧卧位，健侧下肢屈曲，患侧下肢伸直；若内踝损伤患侧卧位，健侧下肢屈曲，患侧下肢伸直，将熏蒸探头对准患处，探头与患处之间间隔 20-30cm，治疗开始，询问病人温度是否合适，体位是否舒服。治疗过程加强巡视，密切观察患者有无不适，熏蒸温度有无异常等等。治疗完毕关闭仪器，用毛巾擦拭熏蒸部位，同时观察局部皮肤情况。每日一次，每次 20 分钟。见图 4

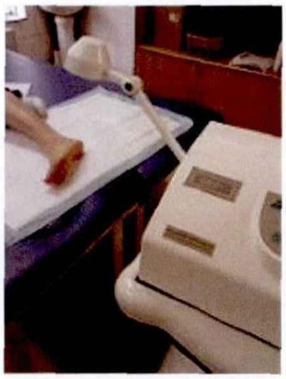

图 4

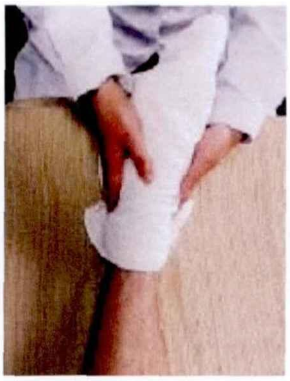

图 5

4 用毛巾擦拭干净患踝。见图 5。

中国知网 https://www.cnki.net

2. 詹氏正骨手法的使用

中药熏药完成后使用詹氏正骨手法进行治疗。具体手法操作步骤如下：

①体位：患者仰卧于操作床上，双下肢平伸，全身放松，呼吸自然。见图5。

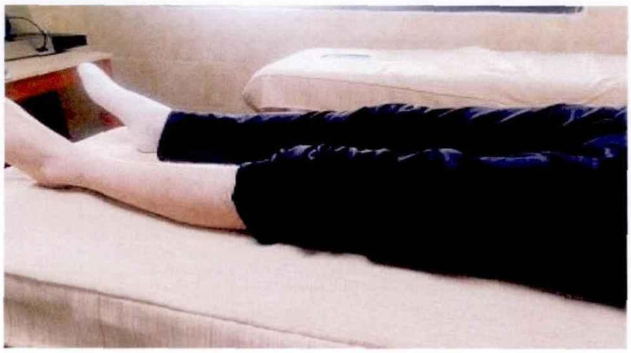

图6

②舒筋：医者左手扶住患踝，使患踝保持中立位，右手拇指及其四指由远及近揉捏小腿肌肉三头肌及胫前肌等。然后用右手拇食指沿着内外踝的边缘做缓慢的推法。若发现肌肉、筋膜、肌腱等软组织增粗、变硬、挛缩、粘连时，予以按压、拨推手法，手法作用力偏重以病人能忍受为度，来回10次。见图7，图8。

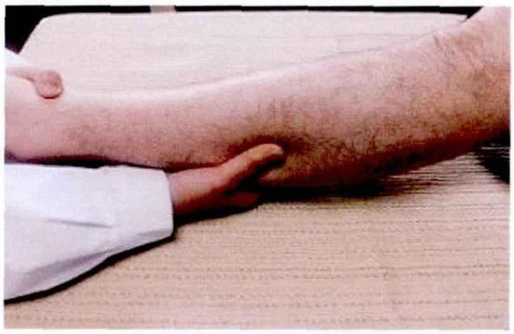

图7

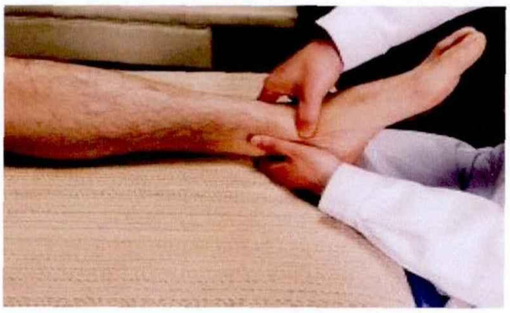

图8

③定点经穴：阿是穴、足三里[1]、丰隆[2]、承山[3]、阳陵泉[4]、解溪[5]、昆仑[6]、悬钟[7]、丘墟[8]、申脉[9]等穴。每个穴位点压10-15秒。见图9，10。

中国知网 https://www.cnki.net

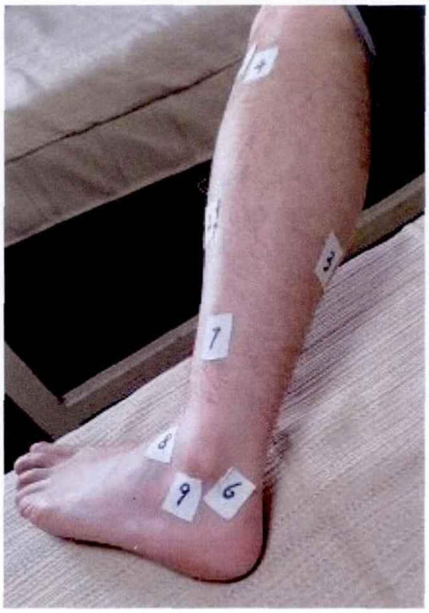

图9

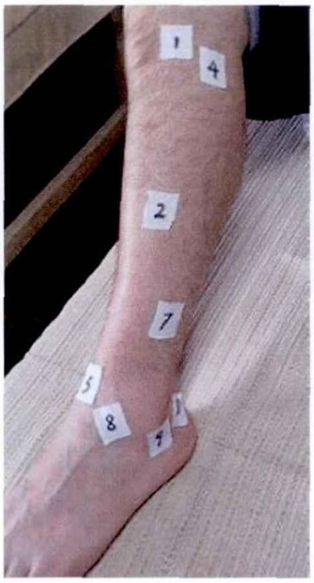

图10

(4)按摩足背及足底。术者用左手固定足部，即用左手拇指及其余四指捏握住患足足趾端，用右手拇指关节沿足背肌腱走行方向由远及近，从外侧到内侧缓慢的推。然后使用左右两手分别拿捏足的内外侧，用双拇指的罗纹面自足背的中央分别向两边分开移动，来回5次，即使用分推法，要求两手用力均匀动作柔和，协调一致。然后用右手食指第二关节沿足底外侧缘、足底中线、足底内侧缘从足趾向足跟部缓慢的使用推法，其余四指呈握拳状，来回5次。见图11，图12，图13。

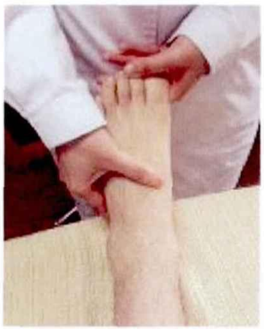

图11

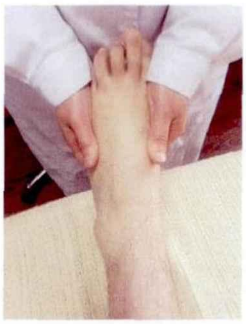

图12

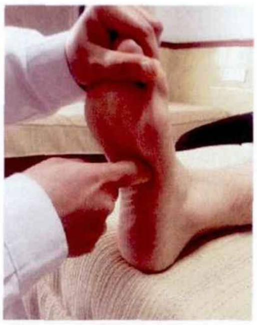

图13

(5)牵引拨伸运踝。术者一手按住患踝关节前方，一手牵拉患足拇趾，患足其余4趾放松，牵拉时要静止用力，持续用力10秒钟，然后依次进行牵拉其余四趾。然后术者用右手拇指及其余四指捏握住患足足趾端，左手托住足跟处，同时

中国知网 https://www.cnki.net

左手拇、食指分别压在内、外踝前下方的间隙处，在夹持踝关节的同时，右手在牵引拔伸下作顺时针运踝，然后在跖屈踝关节到最大范围下，再背伸踝关节至最大范围。最后放松足部及小腿周围肌肉。见图 14，15，16,17。手法治疗整个过程用时 15 分钟左右。

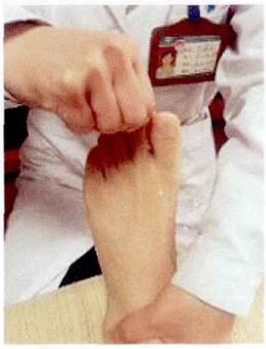

图 14

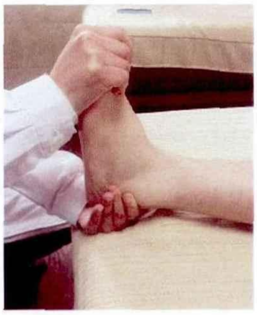

图 15

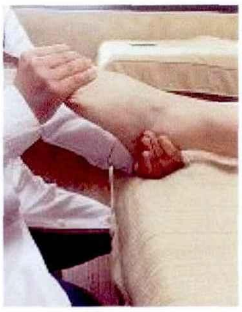

图 16

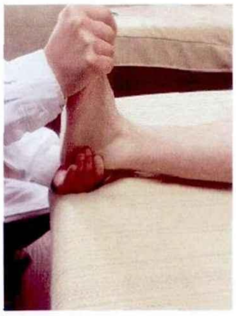

图 17

3. 注意事项：

① 避免过饥、过饱、疲劳、精神紧张时立即进行治疗；

② 患者有思想过虑时，或畏惧情绪时，应事先做好解释工作；

③ 在手法使用前，应根据个体情况选择适当的体位，施治前问患者有无不良不适。

④施术前医生要做好自己的自身清洁工作，指甲的清理，六步洗手法洗手，术后医生同样需做好清洁工作。

⑤ 擦拭毛巾做到一人一块，使用后进行消毒。避免交叉感染。

⑥中药熏药的温度控制一般在 38-42 度之间，熏药时间一般不宜过长，以 20 分钟左右为宜。

五、可能的意外情况及处理方案

1. 中药熏药过程中，需注意观察局部皮肤有无过敏现象发生和熏药温度过

中国知网 https://www.cnki.net

高引起烫伤，嘱患者若发现熏药局部有不适反应及时告知医生。有过敏反应发生应停用中药熏药，局部或口服使用抗过敏药物。若有烫伤，局部使用湿润烧伤膏外涂，若 6 小时后烫伤无好转，暂停后续中药熏药和手法治疗；若短时间内烫伤恢复，进行评估是否继续治疗。同时总结经验控制好熏药温度，加强巡视，防止类似事件的发生。

2. 在患者自身体质较弱，处于过饥、过饱、精神紧张或疲劳的情况下，使用手法过重，容易引起晕厥即头晕眼花、心慌、胸闷等症状的发生；解决类似事件发生的方法是：首诊施术之前评估患者的体质，询问患者有无进食，进食的时间是什么时候，有无过饥过饱及疲劳等现象，同时嘱咐患者精神放松，在综合评估的基础上，若患者未进食，可进食后在进行治疗，但注意进食不要过多。过饱患者和疲劳患者在手法施治时尽量轻柔，嘱咐回家注意休息，和患者说明下次治疗需避免疲劳、过饥或过饱的现象，尽量保持心情放松。治疗过程中不断观察病人反应，调节治疗力度，以病人能承受为度，治疗时间一般掌握在 15 分钟左右。若晕厥已经发生，立即停止治疗，将患者平躺去枕，给患者进服糖水、吸氧等对症处理，尽量保持环境安静，同时请内科协助处理。

3. 疼痛加重：一般由于手法施治时用力过度或用力时间过久引起。故治疗过程中要不断询问病人的感受。一个部位的施治时间不宜过长，用力以患者能承受为宜，时间控制在 15 分钟左右。治疗力度从轻到重再从重到轻。以患者第二次治疗没有发生疼痛加重为宜。

六、康复护理和健康宣教

治疗期间，嘱受试者配合，避免患侧大强度的运动，如避免剧烈的跑跳、爬山等活动，避免踝关节再次扭伤。尽量穿球鞋，不穿高跟鞋和不合脚的鞋。指导患者进行足踝部肌肉训练。

七、不良反应

治疗组在整个治疗观察过程中无明显与治疗有关的不良反应发生。对照组其中 1 例患者在中药熏药后局部皮肤有灼痛感，皮肤较红。考虑与中药熏药时温度过高有关。予以湿润烧伤膏外涂，经休息 3 个小时后恢复正常。没有影响实验的正常进行。同时治疗过程中予以调整熏药温度。

八、疗效

詹氏正骨手法配合中药薰药治疗陈旧性踝关节扭伤后，患者 VAS 评分和

中国知网 https://www.cnki.net

Kofoed 关节功能评分均较治疗前明显改善，而且能维持疗效较长时间（三个月）。

九、本技术卫生经济学分析

詹氏正骨手法配合中药薰药治疗陈旧性踝关节扭伤，在门诊即可开展，无需住院，每日 1 次，每次不足一小时，较方便，痛苦小，病患容易接受。本疗法属于外治疗法，对患者胃肠道疾病和心血管干扰少，禁忌少，适应治疗的患者较多。本疗法费用低廉，经济而实惠，从而缓解看病贵和看病难的问题。另外，本法在操作上简单易学，无需昂贵设备，无需病房配备，适合基层医院、卫生院推广应用。如能在基层开展应用，对于促进基层卫生医疗事业的发展，建构和谐社会，将产生巨大作用。

参考文献

[1]林志斌.陈旧性踝关节扭伤临床研究进展[J].亚太传统医药,2014,10(22):33-34.

[2]实用骨伤科手册[M],湖南:湖南科技出版社,2009:10.

[3]Crichton KJ,Fricker PA,Purdam C,et al.Injuries to the pelvis and lower limb.In Bloomfield J,Fricker PA and Fitch KD.Science and Medicine in Sport 2nd ed[J].Blackwell Science Pty Ltd,Victoria,1995:463-467.

[4] 许涛.踝关节外侧韧带与踝间后韧带的解剖学与生物力学研究[D] 吉林大学,2009:6.

[5]彭力平.实用骨伤科手册[M].湖南:湖南科技出版社,2009:10.

[6]踝关节扭伤中医诊疗方案,24 个专业 105 个病种中医诊疗方案（试行）[S].国家中医药管理局医政司,2011:143-144.

[7]田佳.中医筋伤学[M].成都:成都体育学院教材审定委员会,1996:1,319.

中国知网 https://www.cnki.net
