# Supplementary material for: Efficacy and safety of Tuina (Chinese Therapeutic Massage) for chronic ankle instability: A systematic review and meta-analysis of randomized controlled trials
Source: PLoS One. 2025 Jun 6;20(6):e0321771. doi: 10.1371/journal.pone.0321771 (PMC12143534; doi:10.1371/journal.pone.0321771)
Supplement: S2 File — (ZIP) [file pone.0321771.s004.zip › 5.外踝理筋手法治疗陈旧性踝关节扭伤临床疗效观察_李永恒.pdf]

北京中醫藥大學

BEIJING UNIVERSITY OF CHINESE MEDICINE

硕士研究生学位论文

THESIS OF MASTER’S DEGREE

题目：外踝理筋手法治疗陈旧性踝关节扭伤临床疗效观察

专　　业：中西医结合骨伤科学

研究方向：中西医结合治疗足踝筋伤疾病

学位类型：专业型

硕 士 生：李永恒

导　　师：陈兆军 主任医师

二〇一七年五月

中国知网 https://www.cnki.net

中国知网 https://www.cnki.net

目 录

摘 要 1
ABSTRACT 3
符号说明 5
文献综述 6
综述一 中医对陈旧性踝关节扭伤的认识及治疗进展 6
1. 中医对筋、筋骨、筋伤、筋结的认识 6
2. 中医对陈旧性踝关节扭伤的认识 7
3. 中医对陈旧性踝关节扭伤的治疗进展 8
综述二 现代医学对陈旧性踝关节扭伤的认识 12
1 踝关节的解剖 12
2 陈旧性踝关节扭伤的认识与诊断 13
3 陈旧性踝关节扭伤的治疗进展 15
前 言 18
临床资料 19
1 病例来源 19
2 病例选择 19
3 排除标准 20
4 脱落标准 20
研究方法 21
1 分组方法 21
2 治疗方法 21
3 观察指标 22
4 统计方法 27
研究结果 28
1 一般情况 28

中国知网 https://www.cnki.net

2.疼痛视觉模拟标尺法（Visual analogue scales,VAS）评分 31
3 肿胀程度 33
4 美国足与踝关节协会踝与后足功能评分(AOFAS Ankle-Hindfoot Scale) 34
5 距骨倾斜角 36
6 PANAS-X 具体情绪量表 37
7 总体疗效评定 41
8 不良事件观察 41
讨论 **42**
1 陈旧性踝关节扭伤产生的机制 42
2 陈旧性踝关节扭伤的发病特点 42
3 中医手法治疗陈旧性踝关节扭伤的疗效 43
4 展望 45
结论 **46**
参考文献 **47**
致谢 **52**
个人简历 **53**

中国知网 https://www.cnki.net

摘 要

研究目的：科学地观察中医正骨手法中外踝理筋手法治疗陈旧性踝关节扭伤的临床疗效，较为客观地评估、验证外踝理筋手法的有效性，以期为陈旧性踝关节扭伤的临床治疗提供参考依据。**研究方法：** 2015年9月至2016年12月，临床收集内收型单侧踝关节扭伤患者74例，最终由66例符合标准并资料完整，其中左侧33例，右侧33例。其中男性29例，女性37例，年龄16~64岁，平均36岁。扭伤到治疗时间最短者4周，最长者14个月，平均6.8个月。所有病例采用单盲法，随机分配至手法治疗组（治疗组）与功能锻炼组（对照组）。治疗组进行外踝理筋手法治疗，具体手法要点是：1，手摸心会，寻找点按筋结；2，手法理筋，行摇、拔、戳手法；3，捋顺脉络，轻捋收工。对照组行功能锻炼治疗，具体包括：1，足背身锻炼；足跖屈锻炼；3，踝关节内翻、外翻锻炼；4，提踵锻炼等。对两组患者治疗前后对疼痛学 VAS 评分、踝关节肿胀程度、AOFAS 的 Baird-Jackson 踝关节评分、距骨倾斜角测量、PANAS-X 具体情绪量表等指标进行监测。

研究结果：1.74例患者中66例得到随访,随访时间最短者1个月，最长者6个月，平均4.2个月。2.VAS评分情况:治疗前两组VAS评分差异无统计学意义,具有可比性($p>0.05$)。治疗后，治疗组组内比较，治疗后较治疗前VAS评分下降，差异有统计学意义($p<0.01$)，对照组组内比较治疗后较治疗前 VAS 评分下降，差异有统计学意义（$p<0.01$）；组间比较治疗组 VAS 评分降低程度明显优于比对照组（$p<0.01$），差异有统计学意义。3.踝关节肿胀情况:治疗前两组患者的患侧踝关节肿胀情况无统计学差异（$p>0.05$），具有可比性;治疗后两组患者的患侧踝关节肿胀情况无统计学差异($p>0.05$)。4.AOFAS评分情况：治疗前两组患者的 AOFAS 评分情况无统计学差异（$p>0.05$）；治疗后两组患者组内比较 AOFAS评分均较治疗前有升高趋势且均有统计学差异（$p<0.05$），治疗后两组患者组间比较 AOFAS 评分发现治疗组评分显著高于对照组，差异有统计学意义（$p<0.05$）。5.距骨倾斜角变化情况：治疗前两组患者患侧距骨倾斜角无统计学差异，具有可比性($p>0.05$)；治疗后，治疗组组间治疗前后距骨倾斜角变化无统计学差异（$p>0.05$），对照组组间治疗前后距骨倾斜角变化无统计学差异($p>0.05$)，两组组内比较同样无统计学差异($p>0.05$)。6. PANAS-X 具体情绪量表评分情况：治疗前，两组患者的该评分无统计学差异，具有可比性（$p>0.05$）；治疗组组内比较治疗前后发现，正性情绪评分升高，负性情绪评分下降，与治疗前相比有统计学差异（$p<0.05$）；对照组组内比较治疗前后发现，正性情绪评

中国知网 https://www.cnki.net

分升高，负性情绪评分下降，与治疗前相比有统计学差异（$p<0.05$）;组间比较，治疗后对照组正性情绪评分升高较对照组明显，负性情绪评分下降较对照组明显，差异有统计学意义（$p<0.05$）。

研究结论：1.外踝理筋手法治疗陈旧性踝关节扭伤在缓解疼痛、改善患者踝关节功能方面临床疗效明显，并且优于目前西医常用的踝关节单纯功能疗法锻炼组。2.外踝理筋手法和踝关节功能疗法锻炼均不能改善踝关节的解剖关系，所以对于功能性踝关节不稳定疗效明显，对于机械性踝关节不稳定治疗效果一般。3.治疗组和对照组对改善踝关节肿胀程度无显著性差异。4.外踝理筋手法和功能锻炼治疗陈旧性踝关节扭伤再改善症状的同时，均对患者的情绪有积极作用，但是中医手法治疗组效果明显优于功能锻炼对照组。

关键词：陈旧性踝关节扭伤；外踝理筋手法；功能锻炼疗法；　正性负性情绪量表

中国知网　https://www.cnki.net

ABSTRACT

Objective: Scientific observation of traditional Chinese medicine orthopedic treatment of old ankle sprain clinical efficacy, more objective assessment, verify the effectiveness of traditional Chinese medicine orthopedic techniques, in order to provide a reference for the clinical treatment of old ankle sprain.

Method: From September 2015 to December 2016, 74 patients with unilateral ankle sprain were collected from the clinic, and 66 patients met the criteria and the data were complete. 33 cases on the left, 33 cases on the right side. Including male patients in 29 cases, 37 cases of female. Aged 16 to 64 years, mean 36 years old. Sprained to the shortest treatment time of 4 weeks, the longest 14 months, an average of 6.8 months. All cases were randomized to the surgical treatment group (treatment group) and functional exercise group (control group). The treatment group for traditional Chinese medicine orthopedic treatment, the specific methods are: 1, hand touching heart, looking for points according to tendons; 2, tactical reinforcement, line shake, pull, poke method; 3, stroke along the line, light stroke work. The control group of functional exercise treatment, including: 1, foot back exercise; foot plantar flexion exercise; 3, ankle varus, valgus exercise; 4, heel training and so on. The VAS score of pain, the degree of ankle swelling, the Baird-Jackson ankle score of AOFAS, the measurement of talus tilt angle and the specific mood scale of PANAS-X were monitored before and after treatment.

Result: 1.Of the 74 patients, 66 were followed up for one month and the longest 6 months with an average of 4.2 months.2.VAS score: There was no significant difference in VAS score between the two groups before treatment (P> 0.05). (P <0.01). Compared with the control group, the VAS score of the control group was lower than that of the control group (p <0.01), and the difference was statistically significant (p <0.01). The degree of VAS score in the treatment group was significantly better than that in the control group (p <0.01), the difference was statistically significant. 3. Ankle joint swelling: the two groups of patients before treatment of the affected side of the ankle joint swelling was no statistically significant (p> 0.05), comparable; after treatment, the two groups of patients with ipsilateral ankle joint swelling was no statistically significant P> 0.05). 4.AOFAS Rating: There was no significant difference in AOFAS scores between the two groups before treatment (p> 0.05). The AOFAS scores of the two groups were significantly higher than those before treatment (p <0.05) The scores of the AOFAS scores in the two groups were significantly higher than those in the control group (p <0.05).5. Talus tilt angle situation: There was no significant difference (P> 0.05) between the two groups before and after treatment, and there was no significant difference between the two

中国知网 https://www.cnki.net

groups (p> 0.05). There was no significant difference between the two groups before and after treatment (p> 0.05) (P> 0.05). There was no significant difference between the two groups (p> 0.05). There was no significant difference between the two groups (p> 0.05).6. PANAS-X specific emotional scale score: Before and after treatment, the scores of the two groups were not statistically significant (p> 0.05). In the treatment group, the scores of positive emotion were decreased and the negative emotion score decreased compared with before treatment (P <0.05). Compared with the control group, there were significant differences (P <0.05) between the control group and the control group (P <0.05). There was significant difference between the control group and the control group (P <0.05). The scores of positive emotion scores in the control group were significantly higher than those in the control group (P <0.05).

Conclusion: 1. The use of traditional Chinese medicine orthopedic treatment of old ankle sprain in relieving pain, improve the patient's ankle function in clinical efficacy is obvious, and better than the current Western medicine commonly used ankle function alone exercise group. 2.Orthopedic and ankle exercises can not improve the anatomical relationship between the ankle joint,so the functional ankle instability is obvious, for the mechanical ankle instability treatment effect in general.3.There was no significant difference between the treatment group and the control group in improving the degree of ankle swelling. 4.TCM orthopedic and functional exercise treatment of old ankle sprain and then improve the symptoms at the same time, have a positive effect on the patient's mood, but the effect of traditional Chinese medicine treatment group was significantly better than the functional exercise control group.

Key wotrds: Old ankle sprain, Traditional Chinese medicine orthopedic technique of Lateral ankle，Functional exercise therapy，PANAS-X

中国知网 https://www.cnki.net

符号说明

英文缩写	英文全称	中文全文
CAS	Chronic ankle sprains	陈旧性踝关节扭伤
VAS	Visual Analogue Score	视觉模拟评分法
AOFAS	American Orthopedic and Ankle Society	美国足与踝关节协会
ATFL	Anterior Talofibular Ligament	距腓前韧带
PTL	posterior talofibular ligament	距腓后韧带
CFL	calcaneofibular ligament	跟腓韧带
PANAS-X	Positive and Negative Affect Scale	正性负性情绪量表

中国知网 https://www.cnki.net

文献综述

综述一 中医对陈旧性踝关节扭伤的认识及治疗进展

踝关节是人体的重要结构，是一种高度适配的鞍状负重关节。踝关节扭伤临床非常多见,各年龄组均可发生单纯踝关节韧带损伤往往得不到重视,日后容易导致踝关节不稳[1]。人体关节扭伤中，踝关节扭伤发病率为第一位，踝关节韧带扭伤约占全身韧带扭伤的80%[2]。祖国医学认为，急性筋伤两周后，疼痛渐不明显，瘀肿大部分消退，瘀斑转为黄褐色，功能轻度障碍，如失治误治，即此转为陈旧性筋伤[3]。中医对陈旧性踝关节扭伤的治疗有其独特的优势，现综述如下。

1.中医对筋、筋骨、筋伤、筋结的认识

在祖国医学的伤科领域里，陈旧性踝关节扭伤属于筋伤的范畴。

1.1 筋

筋的含义相当广，它概括了除骨以外的皮肉、筋（筋膜、筋络、筋腱)、脉等组织，相当于现代医学中的肌筋膜、韧带、关节囊、肌肉、肌腱、神经、血管等骨周围一切软组织的统称[4]。中医对筋的认识十分广博。《素问·痿论》中提到“宗筋主束骨而利机关也”[5]，说明筋的功用为束骨利机关,即表示筋对骨关节的功能活动及稳定性有其重要的意义。《灵枢·经筋》中提到“筋为刚”[6]，说明筋具有使骨关节刚强有力的作用，即表明了筋不仅在提供骨关节稳定性方面的重要作用，又表明了筋在骨关节活动中提供动力重要作用。筋在带动骨关节运动的同时，也起到约束骨关节过度运动的作用，如《灵枢·经筋》:“小指次指支转筋，引膝外转筋，膝不可屈伸，腘筋急，前引髀，后引尻”[7]。

1,2 筋骨

筋骨是一个整体，两者相辅相成、相互依赖。筋骨是组成人体运动系统的功能和支持保护机体脏器。筋骨相关，筋束骨而利关节，筋是附着于骨并依靠骨的支架和杠杆作用带动关节运动；骨是靠筋的动力来维持正常的结构和产生关节运动。

筋骨的强弱往往可以表现人体生长、发育、衰老等状态。如《素问·上古天真论》:“女子……四七筋骨坚，……丈夫……三八肾气平均，筋骨劲强，故真牙生而长极；四八筋骨隆盛，肌肉壮满；……肾者主水，受五脏六腑之精而藏之，故五脏盛乃能泻。今五脏皆衰，筋骨解堕，天癸尽矣，故发鬓白，身体重，行步不正，而无子耳”[7]。又如

中国知网 https://www.cnki.net

《灵枢·根结》："血气皆尽，五脏空虚，筋骨髓枯，老者灭绝，壮者不复矣"[7]。均说明人的筋骨于人体生长壮老的内在联系。

筋骨不仅可以反映人体生长壮老矣的自然状态，筋骨还可以表现人体健康与疾病的状况及严重程度。如《素问·气交变大论》[8]："藏气不政，肾气不衡，……民病寒疾于下，……筋骨并辟"；"……民病飧泄霍乱，体重腹痛，筋骨繇复"。又如《素问·至真要大论》[8]："是故百病之始生也，必先于皮毛，邪中之腠理开，开则入客于络脉，留而不去，传入于经，……其留于筋骨之间，寒多则筋挛骨痛，热多则筋弛骨消"。说明筋骨异常是内在脏腑系统疾病的外在表现。

1.3 筋伤

筋伤指的是筋的损伤。筋伤分两种，一种为筋的组织受致病因素损害，一种为筋的功能受致病因素损害。前者为器质性损伤，后者为功能性损伤，统称为筋伤。筋伤病因多为急性外伤或慢性病损。六淫、饮食也可间接导致筋伤的发生。如《素问·宣明五气篇》[8]："五劳所伤：久行伤筋"，说明慢性劳损是筋伤产生的原因之一。又如《素问·阴阳应象大论大论》[8]："酸伤筋""风伤筋"，说明饮食与六淫也是筋伤发生的原因。再就是《素问·刺要论》[8]："刺脉无伤筋，筋伤则内动肝"和《诸病源候论·金疮病诸侯》[9]："金疮伤筋断骨候""腕折破骨伤筋候"以及《诸病源候论·金疮伤筋断骨候》[9]："夫金疮始伤之时，半伤其筋，荣卫不通，其疮虽愈合，后仍令痹不仁也"等，说明外伤也是筋伤产生的重要病因。综上所述，外感六淫、饮食、外伤等都能导致筋伤的发生，有的表现为急性筋伤，而有的则表现为慢性病损。

1.4 筋结

筋结一词，见于《医宗金鉴·正骨心法要旨·踝骨》[10]："用手扶筋，再以手指点按其筋结之处，必令端平。"结，有屈曲、凝结、收敛、凸出之意，如《广雅·释诂》："结，曲也"。故筋结是指筋伤后，气血凝滞，出现局限性结块而高突体表之上，或者筋结掩藏与皮下骨缝之上，虽外观无明显异常，但用手巡按，便可触及。本人结合临床学习中逐渐体会到，陈旧性踝关节扭伤出现的筋结多为手摸筋伤之处，有抚之碍手，推之缠跳，粒粟大小。

本研究中，患者在伤侧踝部特定部位多可触及筋结。

2. 中医对陈旧性踝关节扭伤的认识

踝关节扭伤的病因主要为不慎跌仆或外界暴力，以致筋肉损伤，出现局部疼痛、肿胀、皮下淤血、活动不利等症状。人体遭受外力，血脉经络受损而挫伤，气运行不畅，

中国知网 https://www.cnki.net

气机阻滞不通，不通则痛，局部气机闭塞，可致瘀血，故踝关节扭伤的病机为气滞血瘀。如清代医家沈金鳌在《杂病源流犀烛·卷三十》中云："跌仆闪挫，卒然身受，由外及内，气血俱伤病也"。

陈旧性踝关节扭伤属于筋伤范畴。祖国医学认为急性筋伤两周以后，疼痛渐不明显，瘀肿大部分消退，瘀斑转为黄褐色，但在失治误治的情况下，患处仍旧出现隐痛、酸楚、麻木等症状，并且迁延日久病症无明显改善，并在体力劳动或体育运动过程中症状加剧。此时急性筋伤便转化为了陈旧性损伤[3]。

急性踝关节扭伤后如治疗不及时或处理不得当，迁延日久，可造成踝关节不稳定，继发创伤性关节炎，严重影响踝关节的功能，导致陈旧性踝关节扭伤的产生，严重影响患者的日常生活。

目前对于陈旧性踝关节扭伤诊断和治疗方面的认识，尚无统一的规范和标准。

3. 中医对陈旧性踝关节扭伤的治疗进展

中医治疗陈旧性踝关节扭伤方面，主要是以行气活血、化瘀止痛为治疗大法，总结出动静结合、内外兼治等指导方针，形成了分期辨证用药、中医综合治疗的体系。包括手法按摩、针灸、中药外敷、熏洗等。在此将近年来中医治疗陈旧性踝关节扭伤的进展综述如下。

3.1 单纯中医手法在治疗陈旧性踝关节扭伤的应用

陈旧性踝关节扭伤临床治疗中，中医手法是重要的手段之一，以达到"理筋整复，活血化瘀"的目的。临床骨科医师大多认可采用中医手法治疗陈旧性踝关节扭伤。陈兆军[11]运用孙氏外踝理筋手法治疗陈旧性踝关节 110 例，采用随机对照单盲设计方法进行了分组研究，并统计治疗前后 AOFAS Baird-Jackson 评分、距骨倾斜角、疼痛学 VAS 评分等指标。最终发现，孙氏手法在治疗陈旧性踝关节扭伤方面，对缓解疼痛以及改善踝关节功能方面有显著疗效。高景华[12]应用中医手法治疗陈旧性踝关节扭伤 34 例，治疗前后统计得总有效率为 91.2%。阿伍提·艾克木的研究表明[13]，对 54 例陈旧性踝关节扭伤的患者外踝手法治疗，治愈率达到 85.2%，优良率为 92.6%，疗效显著。陈立等人的研究表明[14]，采用中医推拿的方法治疗陈旧性踝关节扭伤 38 例，治疗两个疗程，治愈率为 73.7%，总有效率为 100%。另有学者乔欣军[15]同样采用中医手法治疗该病，共收集 52 例，治疗前后对比，统计得总优良率为 92%。吴山等学者[16]研究发现中医理筋手法治疗陈旧性踝关节扭伤经 10 次治疗后，总有效率同样可达为 100%。

综上所述，在临床报道的相关文献中体现出，单纯中医手法治疗陈旧性踝关节扭伤

中国知网 https://www.cnki.net

疗效显著，值得临床推广应用。

3.2 单纯中药熏洗在治疗陈旧性踝关节扭伤中的应用

陈旧性踝关节扭伤患侧踝关节局部气血瘀滞、经脉阻塞，故治疗上，应以活血化瘀，疏通经脉为法。中药熏洗既有药物的药效作用，温热的熏洗环境又起到活血化瘀的作用，临床疗效显著，临床医师多倾向应用中药熏洗治疗陈旧性踝关节扭伤。

刘照富[17]应用中药熏洗治疗陈旧性踝关节扭伤 60 例，并将其随机分成治疗组和对照组。治疗组采用中药洗药泡足，具体药物组成为：当归、川牛膝、红花、五加皮、木瓜、续断、鸡血藤、透骨草、伸筋草、川芍各 24g，生艾叶 12g 煎汤外洗，一天 2 次，一次 30 分钟。对照组采用吲哚美辛巴布膏外贴患处。治疗后，两组有效率分别为 93.33%、73.33%，差异有统计学意义(P<0.05)。说明该方法疗效显著,值得临床推广应用。王伟红[18]根据活血化瘀、舒经通络的治疗原则，拟定熏洗方剂：荆芥 20g 、川乌 15g 、草乌 15g、牛膝 20g 、伸筋草 20g 、透骨草 20g 、川断 20g 、当归 20g 、木瓜 20g 、桑寄生 20g 、五加皮 20g ，煎汤熏洗，一日 2 次，一次 30 分钟，10 日一个疗程。其报道结果：临床治愈 20 例，占比 66.67%，显效 5 例，占比 16.7%，有效 3 例，占比 10% 无效 2 例。总有效率达到 93.3 %。说明中药熏洗治疗陈旧性踝关节扭伤疗效显著，值得临床推广。

文献报道中，单纯中药熏洗治疗陈旧性踝关节扭伤的中药方剂具体药物并非完全相同，但多为活血化瘀、舒经活络类药物，临床疗效多显著。

3.3 针灸、针刀疗法在治疗陈旧性踝关节扭伤中的应用

针灸、针刀是中医特色的治疗方法，其治疗的有效性已经被世界认可。临床报道中，有许多医家采用针灸、针刀的方法治疗陈旧性踝关节扭伤，疗效满意。

杨雅琴[19]通过受试者治疗前后的 AOFAS 评分、VAS 评分、ROM 关节活动度变化对常规针刺、电针以及温针灸三种针灸疗法进行了随机对照研究，研究结果表明，电针和温针的治疗方法在改善陈旧性踝关节疼痛方面明显优于常规针刺组，说明电针和温针在治疗陈旧性踝关节扭伤方面疗效显著，值得推广。杨春花[20]收集非洲纳米比亚共和国卡图图拉医院门诊陈旧性踝关节扭伤病例 30 例，采用针刺配合小针刀治疗，疗效满意。秦民安[21]运用小针刀治疗陈旧性踝关节扭伤共收集病例 25 例，全部采用小针刀松解局部组织。术后嘱患者休息三天，三天后疼痛消失，关节活动正常者占 72%；疼痛明显减轻，关节活动正常者占 16%，总体优良率达到 88%，疗效显著，此方法值得临床推广。蔡三金[22]临床报道应用药物电针刀治疗陈旧性踝关节扭伤 117 例，治愈率为 33.33%，

中国知网 https://www.cnki.net

显效及有效率共计66.67%，总有效率达到100%，表明，药物电针刀治疗陈旧性踝关节扭伤疗效显著，值得临床广泛采用。阮炳炎[23]采用毫火针治疗陈旧性踝关节扭伤32例，并和常规毫针针刺进行临床疗效对比观察，治疗两个疗程后，使用Ridit统计分析，治疗组和对照组的95%可信区间分别为（0.5188,0.7230）和（0.2655,0.4763），两组的可信区间没有重叠，且治疗组R＞对照组R。说明毫火针在临床治疗陈旧性踝关节扭伤中疗效显著。

针灸、针刀是中医的特色治疗手段，其对陈旧性踝关节扭伤的治疗疗效得到广泛的认可。但应注意避免在治疗过程中损伤血管、神经、韧带等组织，避免造成医源性损伤。

3.4 中医综合疗法在治疗陈旧性踝关节扭伤中的应用

中医治疗陈旧性踝关节扭伤多是采用两种或两种以上的方法综合治疗，多为中医手法配合中药熏洗、针灸针刀配合中药熏洗、中医手法配合外用膏药等方法。中医综合疗法取各种方法的优势，相得益彰，综合治病，临床疗效得到众多医师的认可。

刘海全[24]运用调经筋手法配合中药熏洗治疗72例陈旧性踝关节扭伤，其中中药熏洗的药物组成为：细辛10g，怀牛膝30g，宽筋藤30g，两面针30g，威灵仙30g，川芎20g，透骨草30g，桂枝15g，常规熏洗。治疗一个疗程后，参照Baird-Jackson踝关节评分系统，统计得疗效优良率为73.6%，说明其所采用的调筋经手法配合中药熏洗治疗该病的方法临床疗效满意。陈可飞[25]采用手法整复配合中药熏洗治疗陈旧性踝关节扭伤23例，其中药熏洗方剂组成为：苏木15g，红花15g，花椒9g，土茯苓15g，当归15g，制乳香9g，透骨草30g，炒川楝子15g，当归15g，白芷15g，牛膝15g，海桐皮15g，羌活15g，五加皮15g，姜黄15g，威灵仙15g，常规熏洗。应用治疗前后Baird—Jackson踝关节评分和ROM评分，统计结果有差异(P<0.01)，其研究证明此方法疗效可靠。刘瑞钦[26]等对推拿联合中药熏洗治疗陈旧性踝关节扭伤进行了临床研究，并将其疗效和封闭联合药物治疗进行了随机对照试验。其中，中药熏洗的药物组成为：伸筋草50g，乳香、没药、透骨草、木瓜各30g，威灵仙、当归、鸡血藤、丹参各50g，延胡索、白芷各30g，常规熏洗。研究结果表明，其采用的推拿手法联合中药熏洗方法临床总有效率为96.32%，明显高于封闭配合口服药物组的32.25%。故该方法疗效显著，值得推广。还有很多临床报道结果相似[27][28][29]，均证明了中医手法配合中药熏洗在治疗陈旧性踝关节扭伤中的确切疗效。

刘保新[30]采用小针刀配合理筋疗法治疗该病70例，研究发现应用小针刀配合理筋疗法临床疗效确切。说明对于陈旧性踝关节扭伤的治疗，小针刀配合理筋疗法有其优势，

中国知网 https://www.cnki.net

值得推广。王敏[31]研究发现中医药联合臭氧对陈旧性踝关节扭伤有效。马美子[32]的一项研究表明，电针结合中药熏洗治疗陈旧性踝关节扭伤疗效显著。范青红[33]研究表明正骨手法配合中药熏洗治疗该病有效。同样，还有大量文献资料显示，中医综合疗法治疗陈旧性踝关节扭伤临床疗效显著，值得推广。

由于踝关节韧带对维持踝关节稳定的重要作用，所以手法与中药熏洗等保守疗法更值得推广应用。虽有医师报道针刀疗法在治疗陈旧性踝关节扭伤中的独特疗效，但由于针刀操作技术要求更高、危险性更大的原因，年轻医师临床中慎重选择。

中国知网 https://www.cnki.net

综述二 现代医学对陈旧性踝关节扭伤的认识

踝关节扭伤临床发病率很高，青少年多见，虽然发病率高，但无论是医生还是患者对此病的重视程度仍然不够[1]。踝关节扭伤多伴随踝关节周围韧带的损伤，踝关节韧带扭伤发病率在全身韧带扭伤中占首位[2]，据统计美国每天踝关节扭伤约发生23000例[34]。踝关节的功能活动离不开踝关节的稳定，然而踝关节的关节囊比较薄弱，踝关节的稳定主要靠踝关节的韧带来维持[35]，踝关节的稳定踝关节扭伤后，如果不能及时有效的治疗，常遗留病痛，多会反复扭伤，迁延日久，形成踝关节不稳，严重者形成踝关节骨性关节炎，导致不可逆的病理变化，严重影响人们的生活和工作。目前学术界对陈旧性踝关节扭伤诊断和治疗尚存争议。

1 踝关节的解剖

人体踝关节又被称为距小腿关节，是人体最大的屈戌关节，行走时负重可达体重的5倍以上[36]。踝关节主要由胫骨和腓骨的下端以及距骨滑车、关节囊、踝关节周围的韧带构成。

1.1 踝关节的骨性结构及其对踝关节稳定性的影响

踝关节的骨性结构主要由为胫骨下端、腓骨下端以及距骨滑车构成。胫骨的内关节面和腓骨的外关节面一同构成了踝穴，距骨滑车嵌合在踝穴中。距骨滑车关节面呈现前宽后窄的形态，当踝关节背伸时，距骨宽部进入踝穴窄部，使得距骨内踝面及外踝面与胫腓骨内外踝上的关节面间隙变小，距骨体在很难向两侧移动，所以，在踝关节背伸时，踝关节相对稳固，不容易发生扭伤。当踝关节跖屈时，距骨体的窄部进入踝穴的宽部，使得距骨内踝、外踝面与胫腓骨关节面之间的间隙变大，距骨体可以在踝穴中发生侧方运动，使得踝关节处于不稳定状态。所以，踝关节在跖屈位时更容易发生扭伤[37]。

外踝的关节面呈三角形，内踝的关节面呈逗号状，并且外踝比内踝要低，在踝关节跖屈位时，外踝面有脱离踝穴的可能，而内踝面仍然可以与踝穴保持接触，使得内踝和外踝受力不均匀，踝关节产生不稳定状态，又结合其结构特点，易发生内翻扭伤[38]。

综上所述，从踝关节骨性结构方面体现出踝关节更容易产生跖屈内翻位损伤，故踝关节扭伤多发生在下楼梯、下坡、下山等情况中。

1.2 踝关节周围韧带及其对踝关节稳定性的影响

踝关节周围韧带对维持踝关节的稳定起到重要的作用[35]。踝关节内侧有三角韧带，其为坚韧的三角形纤维索。踝关节外侧韧带由三条不连续的独立韧带组成，分别为：前

中国知网 https://www.cnki.net

侧的距腓前韧带、中间的跟腓韧带、后方的距腓后韧带。内侧三角韧带较外侧韧带坚强，内侧韧带的坚强使得踝关节外翻时受到的阻力大于踝关节内翻时的阻力，故踝关节更易发生内翻损伤。

1.2.1 踝关节内侧副韧带

踝关节内侧副韧带即三角韧带是维持踝关节内侧稳定的重要结构[39][40]主要由胫距前韧带、胫舟韧带、胫跟韧带及胫距后韧带构成。有研究表明[41]可分为深、浅两层,其中有的三角韧带两层之间有脂肪组织分离。浅层韧带束主要为胫跟韧带，其起点为内踝前丘的前下界，止连与背内侧韧带，形成胫侧弹性束；深层韧带主要为胫距前韧带深层以及胫距后韧带深层，其中胫距前韧带的走行为斜行向前下，胫距后韧带的走行为斜向后下。

由于浅层胫跟韧带前界及深层胫距韧带后界具有等长性，踝关节跖屈时,三角韧带前方由后至前逐渐发生紧张，踝关节背伸时，三角韧带后方由前向后逐渐紧张，为踝关节提供了强有力的防止外翻的稳定力量。

1.2.2 踝关节外侧副韧带

踝关节的结构特性，使得踝关节内翻扭伤发病率远远高于踝关节外翻扭伤[42],踝关节扭伤中，有约90%是外侧副韧带损伤，即踝关节扭伤多为内翻性扭伤。

踝关节外侧副韧带由跟腓韧带(calcaneal fibular ligament,CFL)、距腓后韧带(posterior talofibular ligament,PTFL)、距腓前韧带(anterior talofibular ligament,ATL)组成。三条韧带的主要作用各有不同[43]。跟腓韧带主要的作用主要是阻止足内翻，间接限制距骨倾斜，同时跟腓韧带还有维持距下关节稳定的作用。距腓后韧带是踝关节外侧副韧带中相对最强硬的韧带，其主要作用是阻止距骨向后移位。距腓前韧带是三条韧带中最薄弱的韧带，距腓前韧带的是防止足内翻的最重要的韧带，并且，距腓前韧带向前逐渐与踝关节外侧的关节囊密切相连，故有学者认为距腓前韧带是关节囊的增厚部分[44]。由于踝关节的结构特性，踝关节内翻型扭伤远远多于外翻型损伤，而踝关节内翻时，距腓前韧带首先起到限制作用，当内翻力量过大时，距腓前韧带首先破裂[45]。

综上所述，踝关节扭伤中，多发生内翻位的外侧副韧带损伤，其中又以距腓前韧带的损伤最为多见。

2 陈旧性踝关节扭伤的认识与诊断

陈旧性踝关节扭伤多由急性踝关节扭伤失治误治发展而来，有报道显示，急性踝关

中国知网 https://www.cnki.net

节扭伤转变为陈旧性踝关节扭伤的比例高达 20%至 40%[46]。陈旧性踝关节扭伤者多伴有踝关节外侧副韧带处疼痛、压痛和青肿，尤以距腓前韧带处多见。诊断本病时，应该常规注意收集病史以及临床症状体征，并应考虑到特殊职业的踝关节较正常人松弛的情况（如杂技演员、舞蹈演员、体操运动员等）。

多数学者认为，陈旧性踝关节扭伤最显著的特征是慢性踝关节不稳定（chronic ankle stability，CAI）的存在[47][48]，患者多表现为行走或运动时的恐惧感或不稳定感，过量行走运动后出现关节酸胀疼痛，这些症状，均为踝关节韧带撕裂后踝关节松弛所致[49]。

踝关节不稳的机制，有观点认为，人体感受器受损是主要原因[50]，并进一步将踝关节不稳定分为机械性不稳定（mechanical ankle instability，MAI）和功能性不稳定（functional ankle instability，FAI），前者又细分为骨性不稳定和软组织性不稳定。机械性不稳定指的是在借助 X 线观察到有骨折、先天性骨骼发育异常以及借助 MRI 检查观察到有明显的韧带断裂、关节囊撕裂等器质性病变。功能性不稳定主要是指各种原因引起外踝反复疼痛、压痛、肿胀甚至出现硬结，但关节活动度不一定超出正常生理极限，并在 X 线及 MRI 等辅助检查下午明显的骨折及软组织断裂情况，但会伴有完全或不完全失去自主控制[51]。有学者认为踝关节韧带或踝关节韧带上存在机械感受器，本病就是该感受器受损，导致传导障碍所致[52]。有学者通过动物实验证实，在猫的踝关节韧带上可以监测到电生理反应，其发现感受器多在韧带两端，毗邻骨附着[53]，但是未进一步说明人体踝关节是否存在此感受器。

对于陈旧性踝关节扭伤的诊断，以及踝关节不稳定的诊断，需要从病史、临床表现、体格检查、辅助检查几个方面综合考虑，其中 X 线在诊断本病的过程中十分重要。正常的踝关节正位内翻应力位 X 线应显示为胫距关节内侧间隙和胫距关节外侧间隙等宽。有学者提出[54]在髋、膝关节屈曲 90°、踝关节跖屈 30° 时，拍摄踝关节正位内翻应力位片应显示距骨斜角在 0° 至 5° 范围内。另有学者指出[55]内翻应力位距骨倾斜角大于 10° 时提示存在踝关节外侧韧带损伤；当此角度大于 15° 时，提示距腓前韧带损伤；当角度介于 15° 与 30° 之间时提示距腓前韧带损伤合并跟腓韧带损伤；大于 30° 时提示韧带断裂可能。有学者认为，足前抽屉位 X 线对诊断慢性踝关节扭伤病理状态有诊断价值，当此位置踝关节向前方半脱位 3mm 至 6mm 被认为病理情况存在[43]，然而，正常人双侧距骨倾斜角可相差 5° 或 6°，甚至在 4%至 5%的正常人群众存在双侧距骨倾斜角相差近 19° 而不伴有踝关节不稳定症状，故主张 X 线诊断陈旧性踝关节扭伤存在较大概率的误

中国知网 https://www.cnki.net

诊。有学者主张三维成像 MRI 对诊断合并韧带断裂或损伤的踝关节扭伤临床意义[56][57]，故此可排除因韧带断裂而引起的踝关节不稳定。有学者[58][59]发现可以通过高分辨率的 MRI 检查显示距腓前韧带和跟腓韧带，从而对诊断该病提供帮助。白万山等[60]同意上述观点，并认为 MRI 在诊断踝关节扭伤伴随韧带损伤方面明显优于其他辅助检查手段。但是无论是 X 线还是 MRI 均不能动态观察，也不能满足对踝关节拍摄体位的要求，并存在 MRI 的临床检查费昂贵，耗时久等缺点[61]。

在诊断陈旧性踝关节扭伤的辅助检查手段中，除了 X 线及 MRI 外，肌骨超声的应用也在逐渐增多。林发俭[62]认为，超声是诊断踝关节韧带损伤的可靠方法，并对诊断完全撕裂的韧带有很高的准确性。另有关节造影诊断，但其为有创检查方法，且假阳性率较高，临床难以推广。

综上所述，陈旧性踝关节扭伤的诊断主要以症状体征为主，以明确踝关节扭伤病史为前提，伴随踝关节疼痛、压痛、肿胀、打软腿、踝关节反复扭伤以及对行走的不信任感，并结合 X 线、高频超声、MRI 等检查可初步诊断。

3 陈旧性踝关节扭伤的治疗进展

现代医学认为陈旧性踝关节扭伤的治疗主要是慢性踝关节不稳定的治疗。本人通过查阅相关文献，现将慢性踝关节不稳定的治疗进展综述如下。

3.1 保守治疗

当有慢性踝关节不稳定症状出现时，应该首先考虑保守治疗。女性患者可以通过降低鞋跟的高度或者增宽鞋跟的宽度从而起到保护作用。慢性踝关节不稳定患者在行走或进行重体力劳动之前，可以选择绑束带、护踝从而起到减轻症状的作用。有的患者通过肌肉力量的锻炼，从而治愈慢性踝关节不稳定。保守治疗方案多为对功能性患者有效。

有研究发现，踝关节功能性不稳定与人体平衡控制技能关系重大。踝关节不稳定的保守治疗主要是针对踝关节本体感觉、平衡、肌力方面等[63]。马雪等[64]一项研究显示，其收集慢性踝关节不稳定患者 62 例，随机分为功能锻炼和对照组，通过连续 4 个阶段的功能锻炼，结果显示治疗后 AOFAS 评分显著提高，说明功能锻炼疗法对慢性踝关节不稳定的治疗有临床推广价值。Clark 等[65]通过应用平衡板训练的方法，对 19 例踝关节不稳定的患者进行临床治疗观察，并通过测定胫前肌、腓骨长肌表面肌电反应，结果显示，通过 4 周的功能锻炼后，踝关节功能评分显著改善，说明功能锻炼中的平衡板训练队治疗慢性踝关节不稳定有效。Eils 等[66]的一项研究同样证实了功能锻炼对慢性踝关节不稳的有效性，其采用的功能锻炼方案是通过系统的热身、单腿站立、弹力带外展练习、

中国知网 https://www.cnki.net

平衡练习（气垫、倾斜板、biodex 稳定系统等）等，6 周后，腓骨肌反应时间显著缩短。多数学者认为功能锻炼疗法对慢性踝关节不稳定患者的恢复有积极作用[67]。

3.2 手术治疗

机械性踝关节不稳定通过保守治疗往往疗效不显著，需通过手术治疗。通过手术，对韧带进行复位或者重建十分重要，治疗后应给予康复标准的运动功能恢复和神经肌肉系统功能恢复[68]。手术治疗慢性踝关节不稳定的术式很多[69][70]，具体可分为三大类：1，原位缝合类；2，非解剖重建类；3，解剖重建类。现分述如下。

3.2.1 原位缝合治疗慢性踝关节不稳定

治疗慢性踝关节不稳定的原位缝合重建手术可分为韧带紧缩术和韧带皱缩术。韧带紧缩术一般在开放下进行，但近年有学者[71]主张在踝关节镜下进行韧带紧缩术，其认为踝关节镜的治疗手段相对于开放式手术在创伤更小的前提下，有恢复快以及可以达到开放手术的等同术后效果的特点。开放式的原位缝合韧带术式以 Brostrom 术式为代表[72]，具体操作方法是在外侧韧带体部切断之后进行紧缩缝合。近年有学者利用铆钉将此术式进行了改进[73],临床效果满意。韧带皱缩术是在关节镜操作下，利用射频技术皱缩踝关节韧带，达到改善踝关节稳定性的作用[74]。其原理和肩关节囊皱缩原理相同，使软组织在皱缩的同时保持一定的生物活性，但可造成软组织的破坏和吸收，故临床逐渐放弃使用。

3.2.2 非解剖重建治疗慢性踝关节不稳定

利用肌腱非解剖重建外踝韧带的手术主要应用于断裂的韧带残端吸收、韧带过多松弛等，临床采用较多。代表术式有 Evans 术式、Watson-Jones 术式、Chrisman-Snook 术式等。

Evans 术式是利用外踝切口，暴露腓骨肌腱，切取腓骨短肌腱并通过外踝钻孔形成骨隧道，将腓骨短肌腱穿过，最终与骨膜缝合，短期疗效可，长期疗效欠佳[75]，并存在皮肤瘢痕长、愈合时间长、腓骨肌力下降等缺点。Watson-Jones 术式是对 Evans 术式的改良和补充，切口暴露同 Evans 术式，并在外踝尖近侧开一斜行骨道，再于距骨颈外侧开第二个骨道，将腓骨短肌腱从后方穿过第一个骨道再从下方穿过第二个骨道，并最终与骨膜缝合。有学者报道，通过 22 年的随访发现，采用 Watson-Jones 术式治疗慢性踝关节不稳定疗效不满意[76]。但更多的学者报道了此方法临床效果良好[77][78]。故认为有临床推广价值。Chrisman-Snook 术式是通过多个骨隧道重建距腓前韧带的方法，报道称此方法可靠[79]。

中国知网 https://www.cnki.net

3.2.3 解剖重建治疗慢性踝关节不稳定

改良的 Brostrom 手术是一种解剖型的重建手术，对于任何决定手术的患者而言，都是首选[80]。术者在术前应做好其他术式的准备，因为术中如发现韧带明显回缩或钙化则此术式不在适合应用，而应当改用肌腱移位或肌腱移植手术。改良的 Brostrom 手术可以维持踝关节稳定的同时，可以恢复踝关节的功能。

华英汇[81]报道应用异体半腱肌腱对该病患者进行解剖重建，并进行平均 37.9 个月的随访，AOFAS 评分、Karlsson 评分显著提高，临床疗效满意。有学者采用自体趾伸肌腱同样疗效满意[82]。

综上所述，西方医学对慢性踝关节不稳定的治疗术式复杂多样，且疗效并不都十分理想。特别是由于术式多样及术者的个人常用术式等不同，使得慢性踝关节不稳定的手术术式选择存在一定争议。

中国知网 https://www.cnki.net

前言

踝关节是人体站立、行走、运动等的重要结构，是一种高度适配的鞍状负重关节。踝关节扭伤临床非常多见，各年龄组均可发生单纯踝关节韧带损伤往往得不到重视，日后容易导致踝关节不稳[1]。人体关节扭伤中，踝关节扭伤发病率为第一位，踝关节韧带扭伤约占全身韧带扭伤的80%[2]。祖国医学认为，急性筋伤两周后，疼痛渐不明显，瘀肿大部分消退，瘀斑转为黄褐色，功能轻度障碍，如失治误治，即此转为陈旧性筋伤[3]。西医治疗陈旧性踝关节扭伤多采取手术治疗，且术式多样，没有统一的术式标准，且不同学者对同一术式报道的临床疗效也存在差异，有学者推崇原位缝合修复韧带损伤，有学者推崇解剖复位缝合韧带，有学者推崇非解剖复位的韧带重建修复韧带损伤，临床争议较大。中医骨伤科治疗陈旧性踝关节扭伤有其独特优势，特别是中医正骨手法治疗该病的临床疗效得到广大学者的认可，其中又以摇拔戳手法最为令人称道，因此我设计了本项目课题的应用研究，使用单盲随机对照的研究方法，来观察中医正骨手法中的外踝理筋治疗陈旧性踝关节扭伤的临床疗效。将外踝理筋手法组设置为治疗组，功能锻炼组设置为对照组。采用的评价指标如下：1.疼痛视觉模拟标尺法（Visual analogue scales,VAS）评分；2.肿胀程度；3.美国足与踝关节协会踝与后足功能评分(AOFAS Ankle-Hindfoot Scale)；4.距骨倾斜角；5.PANAS-X具体情绪量表评价治疗前后患者情绪心理变化；6.总体疗效评价。并对不良事件进行观察记录。本研究的目的是通过病例对照研究，客观的验证中医手法治疗陈旧性踝关节扭伤的有效性，为临床医生提供治疗参考。具体研究过程如下。

中国知网 https://www.cnki.net

临床资料

1 病例来源

自 2015 年 9 月 1 日至 2016 年 12 月 31 日，收集来源于北京中医药大学第三附属医院手足外科门诊及金盏社区卫生服务中心门诊就诊的该病患者病例共计 74 例。均为单侧陈旧性踝关节损伤。本课题病例选择均为内翻型损伤即踝关节外侧副韧带陈旧性损伤患者。

2 病例选择

2.1 诊断标准

2.1.1 中医诊断标准

陈旧性踝关节扭伤的中医诊断标准采用 2011 年颁布的中医诊疗方案[83]，该诊疗方案将踝关节扭伤分为两个证型。其中，血瘀气滞型的临床症状体征表现为：踝关节扭伤早期出现的踝关节疼痛、活动加剧，局部肿胀显著及可见明显皮下瘀斑、关节功能活动受限、舌红有瘀点、脉弦；筋脉失养型的临床症状体征表现为：踝关节扭伤后期，关节持续性隐痛、可见轻度肿胀、无明显瘀斑、踝关节局部可触及硬结、步行受限、舌淡苔薄白、脉弦细。

故本课题所涉及的陈旧性踝关节扭伤辩证分型属于踝关节扭伤筋脉失养型。

2.1.2 西医诊断标准

西医诊断标准参考《实用骨伤科手册》[84]。

（1） 明显外伤史；

（2） 踝关节肿胀、酸痛乏力，关节活动时可有摩擦感，久行、阴雨天时加重；

（3） 外踝前下方及内踝前外侧有肿胀、压痛，内翻、屈伸时活动可受限；

（4） 检查未发现骨折和脱位，X 线检查未见骨折；

（5） 病程超过 20 天未愈。

符合 1、2、5 或者 1、3、5 或者 1、3、4、5 或者 1、2、4、5，即可诊断该病。

2.2 纳入标准

（1）就诊于北京中医药大学第三附属医院手足外科门诊或北京市朝阳区金盏社区服务中心门诊的第一诊断陈旧性踝关节扭伤患者；

（2）符合上述诊断，并为单侧踝关节内翻型扭伤或主要症状表现为单侧外踝损伤，病程超过 20 天；

中国知网 https://www.cnki.net

（3）经 X 线检查无骨折病史、经高频超声检查无踝关节副韧带完全断裂；

（4）不伴有其他足部畸形；

（5）近期未进行口服药物以及其他相关保守或非保守治疗；

（6）年龄满足 16 周岁以上（包含 16 岁），65 周岁以下（包含 65 周岁）；

（7）患者无既往皮炎、皮肤病等病史，无外伤手术史等；

（8）可配合进行中医手法治疗及功能锻炼疗法治疗；

（9）伦理委员会同意，签署知情同意书。

3 排除标准

（1）年龄小于 16 周岁或者年龄大于 65 周岁；

（2）合并有危及生命的原发病，如心、脑、肝、肾以及造血系统等原发病。

（3）合并局部皮肤软组织破损或伴有感染者；

（4）合并风湿性关节炎、类风湿性关节炎、滑膜炎等影响踝关节活动者；

（5）有明确韧带断裂或骨折（新鲜骨折或陈旧骨折）；

（6）合并精神疾病以及阿尔茨海默病等无法配合治疗者；

（7）妊娠及哺乳期妇女。

4 脱落标准

（1） 治疗过程中患者出现严重不良反应和（或）其他不可预测的情况，患者无法配合继续进行研究者；

（2） 治疗过程中患者主动要求停止治疗或中途强烈要求选择其他治疗方案者；

（3） 在治疗过程中患者因出现其他疾病，需要诊治他病，不再适合进行本研究者；

（4） 治疗过程中发现韧带断裂或其他需要手术治疗从而必须停止保守治疗者。

中国知网 https://www.cnki.net

研究方法

1 分组方法

本人经过前期的临床观察并进行了初步的文献研究，文献来源于 CNKI、万方数据库，关键词为陈旧性踝关节扭伤、中医正骨手法、功能锻炼。查阅相关资料后并根据样本量计算公式：$n=[(Z_{1-\alpha/2}+Z_{1-\beta})\times(\sigma_1^2+\sigma_2^2)]/\delta^2$[85]得出样本量为 64 例，按照 15%脱落计算，得最终样本量为 74 例。每组应观察 37 例。收集北京中医药大学第三附属医院手足外科门诊和北京金盏社区卫生服务中心门诊符合本研究纳入标准及排除标准要求的陈旧性踝关节扭伤病例，确定受试者，并借助统计软件 spss19.0，应用随机数字表法，随机分组，将 74 例患者随机分为治疗组和对照组，每组 37 例。治疗组采用中医正骨手法中的外踝理筋手法治疗；对照组采用系统功能锻炼疗法治疗。

2 治疗方法

2.1 治疗组

治疗组采用中医正骨手法的外踝理筋手法治疗该病。外踝理筋手法治疗陈旧性踝关节扭伤有一套完整的操作流程[11]，具体操作如下：

（1） 按揉筋结：令患者侧卧于治疗床上，患侧在上，健侧在下，令助手握持患肢踝部上端，施手法者用拇指指腹在解剖距腓前韧带处轻巧柔和寻找筋结，多可触及一粟粒大小的韧性结节，按揉此处 10 余次。

（2） 摇：施手法者两手掌虎口相对持住踝关节，拇指轻柔按压住踝关节的踝缝，其余手指握住患足，摇晃环绕 7 次。

（3） 拔：施手法者和助手配合，并保持患足跖屈内翻位，进行轻柔拔伸。

（4） 戳：施手法者将患足置于足背伸外翻位，双手拇指向踝缝下轻按，双手合力戳按踝关节。

（5） 轻捋收功：施手法者对患者轻捋患肢，捋顺筋脉。

备注：每次治疗进行摇拔戳 7 次，每隔 2 天治疗 1 次，共计治疗 6 次。

2.2 对照组

对照组采用踝关节功能锻炼疗法，具体治疗流程如下[86]：

（1） 足背伸功能锻炼：足背伸功能锻炼可以起到锻炼胫骨前肌的作用。动作要领如下：身体保持站立体位，健侧下肢负重，患侧足背伸位保持 20 秒，5 次一组，每次治疗做 2 组。

中国知网 https://www.cnki.net

（2） 足跖屈功能锻炼：足跖屈功能锻炼可以起到锻炼胫骨后肌和小腿三头肌的作用。动作要领如下：身体保持站立体位，健侧下肢负重，患侧足跖屈位保持20秒，5次一组，每次治疗做2组。

（3） 踝关节内翻、外翻功能锻炼：踝关节内翻、外翻功能锻炼可以起到锻炼腓骨长短肌和胫骨前肌的作用。动作要领如下：身体保持站立体位，健侧下肢负重，患侧足内翻位及外翻位个保持20秒，5次一组，每次治疗做2组。

（4） 提踵功能锻炼：提踵功能锻炼的可以起到锻炼小腿三头肌的作用。动作要领如下：身体保持站立体位，双足自然分开与肩同宽，原地提踵，脚尖负重，过程缓慢进行，10次一组，每次治疗做2组。

备注：每隔2天治疗1次，共计治疗6次。

3 观察指标

3.1 随访流程

患者按研究要求入组后，无论是治疗组还是对照组，第一次治疗前进行CRF表的填写及治疗前入组访视一，并按流程进行访视二（入组第1天第一次治疗后）、访视三（入组第16天第六次治疗后）。按疗程治疗后，进行治疗后的随访，分别进行治疗后1周（访视四）、1个月（访视五）、3个月（访视六）随访，访视视窗为±2天。具体各项访视指标统计分述如下：

3.2 一般项目

一般项目包含入组患者的受试编码、姓名、入组日期、家庭住址、联系方式、身份证编号。

3.3 一般生物学指标

一般指标：性别、年龄、身高、体重。

生命体征：体温、血压、呼吸、心率。

3.4 疼痛视觉模拟标尺法（Visual analogue scales,VAS）评分

疼痛学指标采用疼痛视觉模拟评分法（Visual analogue scales,VAS）评分。在CRF表中做一个10厘米的标尺，左端为“0分”表示不痛，右端为“10分”表示剧痛，让患者在每次访视中划线选择。每次访视结果记录于CRF表中。

中国知网 https://www.cnki.net

VAS评分

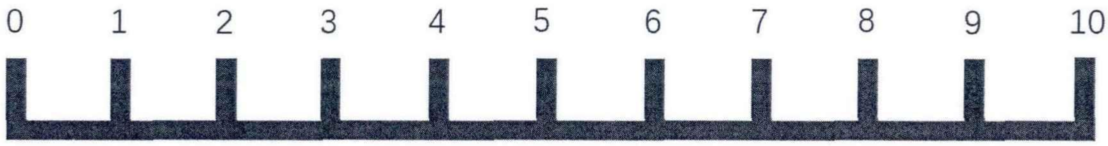

*疼痛视觉模拟评分法（Visual Analogue Scale.VAS）.是将疼痛的程度用0至10共11个数字表示．0代表无痛．10代表剧痛难忍．请根据自身疼痛在这11个数字中选择一个．代表自己目前的疼痛状态

3.2 肿胀程度

按照《中药新药临床研究指导原则》[57]，并参照软组织损伤症状分级标准（尺标法健侧对比），采用健侧和患侧测量对比的方法，用游标卡尺测量双侧踝关节中心三次并取其差值的平均值。轻度：双侧宽度差值小于等于 0.5cm；中度：双侧宽度差值介于 0.5cm 和 1.0cm 之间；重度：双侧宽度差值大于等于 1.0cm。每次访视结果记录于 CRF 表中。

肿胀程度

	访视一	访视二	访视三	访视四	访视五	访视六
轻度肿胀						
中度肿胀						
重度肿胀						

*按照《中药新药研究指导原则》，并参照软组织损伤症状分级标准（尺标法健侧对比），采用健侧和患侧对比的方法，用游标卡尺测量双侧踝关节中心三次并取其差值的平均值。轻度：双侧宽度差值小于等于0.5cm；中度：双侧宽度差值介于0.5cm和1cm之间；重度：双侧宽度差值大于等于1.0cm。

3.3 美国足与踝关节协会踝与后足功能评分(AOFAS Ankle-Hindfoot Scale)

本研究的踝关节功能性评价采用 AOFAS 踝与足功能评分。本评分共计分为九个部分，分别对疼痛（40 分）、功能和自主活动、支撑情况（10 分）、最大步行距离（街区数）（5 分）、地面步行的困难程度（5 分）、反常步态（8 分）、前后活动（屈曲加伸展）（8 分）、后足活动（内翻加外翻）（6 分）、踝-后足稳定性（前后，内翻-外翻）（8 分）、足部对线（10 分）等进行了评分评价。并根据最终得分评定优：90 ～ 100 分；良：75 ～ 89 分；可：50 ～ 74 分；差：50 分以下。每次访视在 CRF 表中记录此评分。

中国知网 https://www.cnki.net

AOFAS踝-后足评分系统（AOFAS Ankle Hindfoot Scale）

	评 分
疼痛（40分）	
无	40
轻度，偶尔	30
中度，常见	20
严重，持续	0
功能和自主活动、支撑情况（10分）	
不受限，不须支撑	10
日常活动不受限，娱乐活动受限，需扶手杖	7
日常和娱乐活动受限，需扶手杖	4
日常和娱乐活动严重受限，需扶车、扶拐、轮椅、支架	0
最大步行距离（街区数）（5分）	
大于6个	5
介于4 ~ 6个	4
介于1 ~ 3个	2
小于1个	0
地面步行（5分）	
任何地面无困难	5
走不平地面、楼梯、斜坡、爬梯时有困难	3
走不平地面、楼梯、斜坡、爬梯时很困难	0
反常步态（8分）	
无、轻微	8
明显	4
显著	0
前后活动（屈曲加伸展）（8分）	
正常或轻度受限（>30°）	8
中度受限（15° -29°）	4
重度受限（<15°）	0
后足活动（内翻加外翻）（6分）	
正常或轻度受限（75%-100%正常）	6
中度受限（25%-74%正常）	3
重度受限（<25%）	0
踝-后足稳定性（前后，内翻-外翻）（8分）	
稳定	8
明显的不稳定	0
足部对线（10分）	
优：跖行足，踝-后足排列正常	10
良：跖行足，踝-后足明显排列成角，无症状	5
差：非跖行足，严重排列紊乱，有症状	0

优：90 ~ 100分；良：75 ~ 89分；可：50 ~ 74分；差：50分以下

中国知网 https://www.cnki.net

3.4 距骨倾斜角

内翻应力位的距骨倾斜角测量对慢性踝关节不稳定意义重大[illegible]，具体操作方法如下：患者屈髋屈膝，小腿内旋 30°，踝关节跖屈 20°，检查者一手持住踝关节，另一手持住跟骨并使患者踝关节处于极度内翻不可推动，维持此应力拍摄 X 线。由于此方法存在检查者需暴露于放射线下的缺点，故借助科室研发的足踝内翻应力位拍摄设备。

拍摄正位 X 线片后，取胫骨远端和距骨上端的连线夹角，正常为 0° 至 5°。治疗前拍摄一次，全部治疗后拍摄第二次，共计两次。

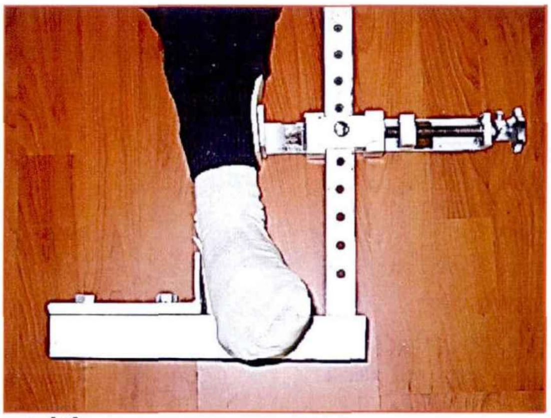

3.5 PANAS-X 具体情绪量表[89]

本人在临床中发现，陈旧性踝关节扭伤会不同程度地给患者带来心理上的困扰，尤其是对走路的不安全感，会严重影响患者的生活质量。更有甚者，本人在临床中发现，有一部分陈旧性踝关节扭伤患者存在焦虑状态。

故本研究引入 PANAS-X 具体情绪量表来评价患者治疗前后情绪的情绪变化。本量表包含正性情绪和负性情绪两个部分，能有效反映出积极情绪及消极情绪两个方面，相对于汉密顿抑郁量表（SAS）、抑郁自评量表（SDS）等单方面的情绪量表，本量表更能客观全面反映治疗前后患者的情绪变化[illegible]。

每次访视让患者自行打分，并记录于 CRF 表中。

得分统计

时间	正性情绪平均分分	标准差	负性情绪平均分分	标准差
今天				
这一个月				

中国知网 https://www.cnki.net

积极情绪消极情绪量表（PANAS-X）

（Watson et al.）

请阅读每一个词语并根据你近"1-2星期"的实际情况在相应的答案上打"√"

词	1	2	3	4	5	词	1	2	3	4	5
欢乐的						不友善					
伤心的						收到惊吓					
活跃的						轻蔑的					
对自己生气						孤独的					
遭人讨厌的						自豪的					
平静的						惊讶的					
内疚的						放松的					
热情的						警觉的					
注意的						战战兢兢的					
害怕的						有兴趣的					
有乐趣的						易怒的					
消沉的						苦恼的					
害羞的						活泼的					
疲劳的						厌恶					
焦虑的						喜悦的					
懦弱的						愤怒的					
无精打采的						感到羞愧					
吃惊的						自信					
寂寞的						有灵感的					
痛苦的						冒失的					
大胆的						自在的					
虚弱的						精力充沛的					
昏昏欲睡的						勇敢的					
应受谴责的						忧郁的					
感到惊讶						恐惧的					
快乐的						集中注意力的					
兴奋的						厌恶自己					
坚决的						胆怯的					
坚强的						昏昏欲睡					
胆小的						不满自己					

*备注 项目分析

1、一般特征量表
负性情绪(10)害怕的,恐惧的,焦虑的,战战兢兢的,易怒的,不友善,内疚的,感到羞愧,苦恼的,痛苦的
正性情绪(10)活跃的,警觉的,注意的,坚决的,热情的,兴奋的,有灵感的,有兴趣的,自豪的,坚强的
2、基本负性情绪量表
害怕（6）害怕的,恐惧的,受到惊吓,焦虑的,战战兢兢的,虚弱的
敌意（6）愤怒的,不友善,易怒的,轻蔑的,厌恶自己,厌恶
内疚（6）内疚的,感到羞愧,应受谴责的,对自己生气,厌恶自己,不满自己
悲哀（5）伤心的,忧郁的,消沉的,孤独的,寂寞的
基本正性情绪量表
愉快（8）快乐的,有乐趣的，喜悦的,欢乐的,兴奋的,热情的,活泼的,精力充沛的
自信（6）自豪的,坚强的,自信,冒失的,大胆的,勇敢的
关心（4）警觉的,注意的,集中注意力的,坚决的
4、其他情绪状态
胆怯（4）胆怯的,害羞的,懦弱的,胆小的
疲劳（4）昏昏欲睡,疲劳的,无精打采的,昏昏欲睡
安静（3）平静的,放松的,自在的
惊奇（3）吃惊的,感到惊讶,惊讶的

中国知网 https://www.cnki.net

3.7 总体疗效评定

本研究的总体疗效评定采用《中医病症诊断疗效标准》中的相关内容[91]。

（1） 治愈：踝关节局部肿痛消失，踝关节不稳定感消失，踝关节功能活动恢复正常。

（2） 好转：踝关节局部肿痛明显减轻，可存在轻度肿胀或轻度瘀斑，踝关节稳定性欠佳，行动活动力量欠佳，踝关节可存在轻微酸痛。

（3） 未愈：踝关节不稳定症状无明显改善，存在局部明显肿痛，关节不稳，活动受限。

疗程结束后记录于 CRF 表中。

3.8 不良事件观察

本研究治疗组与对照组治疗观察过程中均未出现明显不良事件。

4 统计方法

本研究后期将数据收集后，采用 SPSS19.0 进行统计学处理。为保证临床研究和统计学研究的一致性，咨询相关统计学老师后拟定统计学方法选择：用非参数检验的方法统计计数资料;用 t 检验或方差分析的方法统计计量资料;用 Ridit 分析统计临床疗效。具体统计结果细则见研究结果部分。

中国知网 https://www.cnki.net

研究结果

1 一般情况

经过筛选，最终纳入病例 74 例，其中我院手足外科门诊 58 例，金盏社区门诊 16 例。其中 4 例患者因自身其他疾病情况中途终止治疗，2 例患者因出国等事宜终止治疗，2 例对照组患者未能坚持功能锻炼疗法，中途自行停止，剔除脱落率为 10.8%，本研究最终得到 66 例资料完整的病例，随访率为 89.2%，治疗组 34 例，对照组 32 例。

治疗组 34 例患者中，男性患者 14 例，女性患者 20 例。均为单侧内翻型损伤，男性患者中左踝关节陈旧性扭伤 8 例，右踝关节陈旧性扭伤 6 例；女性患者中左踝关节陈旧性扭伤 11 例，右踝关节陈旧性扭伤 9 例。对照组 32 例患者中，男性患者 15 例，女性患者 17 例。均为单侧内翻型损伤，男性患者左踝关节陈旧性扭伤 8 例，右踝关节陈旧性损伤 7 例。

本研究中的收集的病例，患者就诊时间距扭伤时间最短者 3 周，最长者 8 个月，平均 2.8 个月。患者年龄分布最小者 17 岁，年龄最大者 58 岁。两组患者经统计学检验，在性别、年龄、患侧分布、疗程等基线资料方面差异无统计学意义（$p>0.05$），具有可比性。

1.1 性别

本研究中，治疗组男性患者 14 例，女性患者 20 例；对照组男性患者 15 例，女性患者 17 例，经过 x^2 检验，x^2 等于 0.217，p 值等于：0.641（$p>0.05$），治疗组和对照组性别无显著差异，有统计学意义，具有可比性。见表一。

表一：两组性别比较（X^2）

组别	N	男	女	X^2	P
治疗组	34	14（41.2%）	20（58.8%）	0.217	0.641
对照组	32	15（46.9%）	17（53.1%）		

1.2 年龄

本研究中，治疗组年龄最小者 17 岁，年龄最大者 58 岁，平均 34.6 岁；对照组年龄最小者 18 岁，年龄最大者 58 岁，平均 36.7 岁。其中治疗组年龄符合正态分布，对照组年龄不符合正态分布，故两者对比选用非参数检验，统计得 Z=-0.308，p=0.758（p

中国知网 https://www.cnki.net

＞0.05）,两组年龄无差异，有统计学意义，故可比。见表二。

年龄分布情况如下:治疗组小于20岁3人，20-30岁11人，31-40岁10人，41-50岁8人，51岁以上2人；对照组小于20岁1人，20-30岁11人，31-40岁10人，41-50岁7人，51岁以上4人。两组患者年龄分布情况经统计学比较，差异无统计学意义（p>0.05）。见图一。

表二：两组年龄比较（$\bar{X}$±S）

组别	N	平均（岁）	Z	P
治疗组	34	34.63±1.83	-0.308	0.758
对照组	32	36.72±2.16		

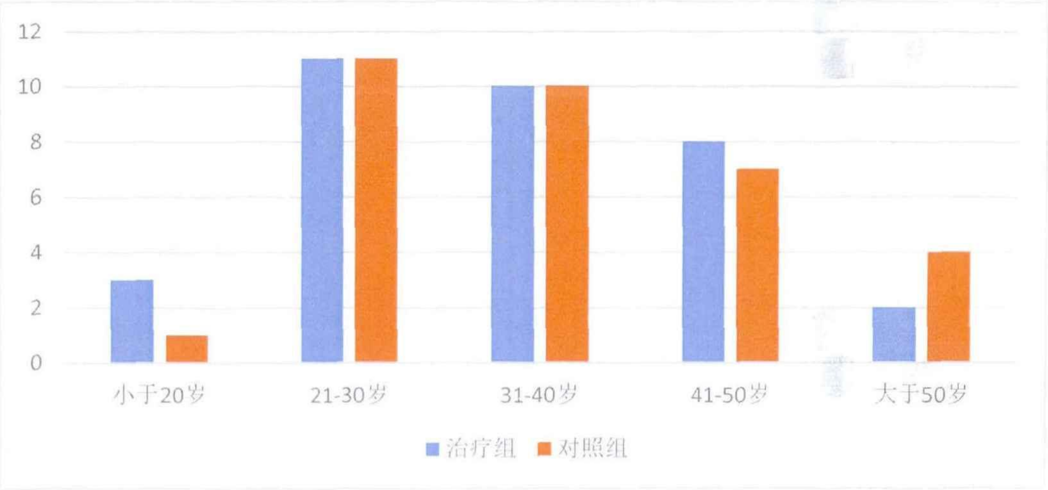

Figure 1 两组患者年龄分布情况

1.3 患侧

本研究中，治疗组左侧陈旧性踝关节扭伤18例，右侧陈旧性踝关节扭伤16例；对照组左侧陈旧性踝关节扭伤15例，右侧陈旧性踝关节扭伤17例。两组患侧例数经χ^2检验，χ^2等于0.323，p值等于：0.570（p＞0.05），有统计学意义，故可比。见表三及图二。

中国知网 https://www.cnki.net

表三：两组患侧比较（X^2）

组别	N	左侧	右侧	X^2	P
治疗组	34	18（52.9%）	16（47.1%）	0.323	0.570
对照组	32	15（46.9%）	17（53.1%）		

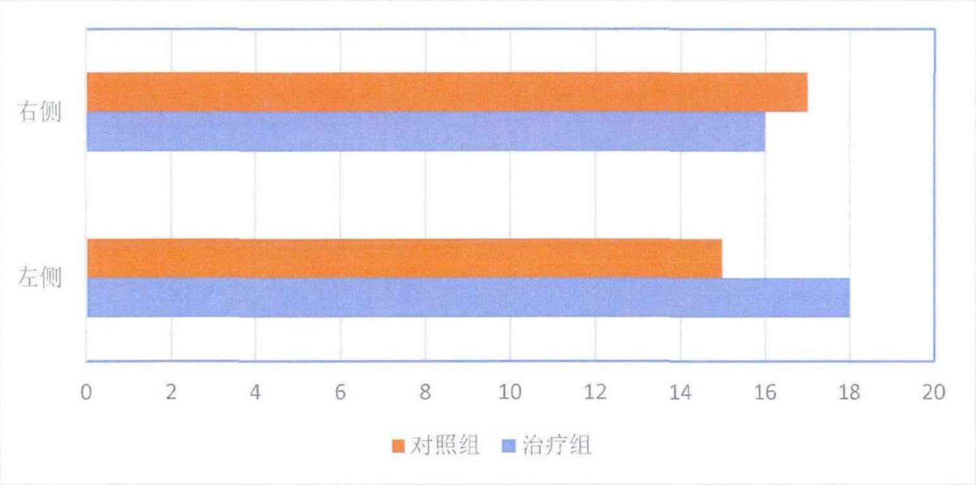

Figure 2 两组患者患侧分布情况

1.4 病程

本研究中，治疗组病史最短者 3 周，病史最长者 16 个月，其中 3-6 周者 7 人，4-8 周 5 人，8-16 周者 13 人，16 周以上者 9 人，平均 13.9 周；对照组病史最短者 4 周，病史最长者 18 个月，其中 3-6 周者 6 人，4-8 周 5 人，8-16 周者 14 人，16 周以上者 7 人，平均 12.1 周经统计学两组病史分布均成正态分布，故采用 t 检验，检验得 t=0.049，p=0.414（p＞0.05），故可比。见表四及图三。

表四：两组病程比较（$\overline{X}$±S）

组别	N	平均（月）	t	P
治疗组	34	13.9±2.83	0.049	0.758
对照组	32	12.1±1.16		

中国知网 https://www.cnki.net

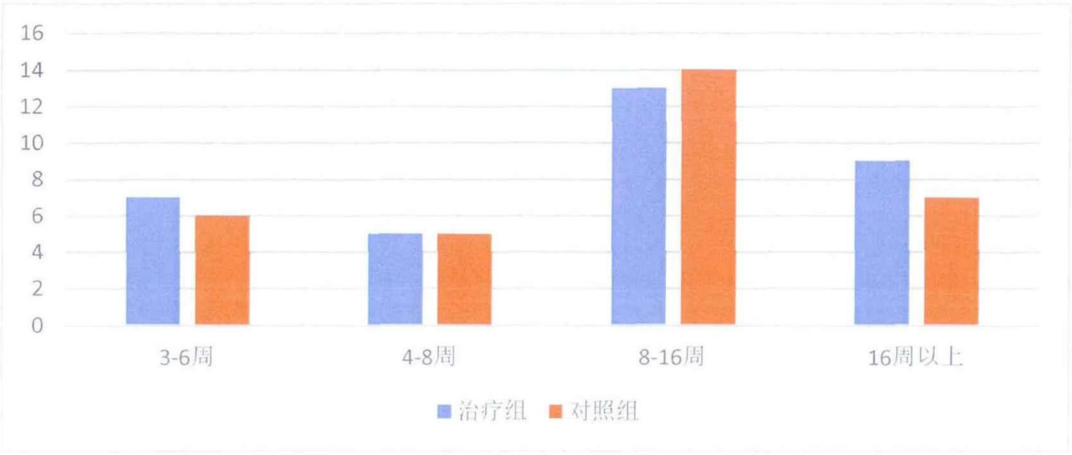

Figure 3 两组患者病史分布情况

2.疼痛视觉模拟标尺法（Visual analogue scales,VAS）评分

本研究中，治疗组与对照组治疗前对比，经统计学检验两组VAS评分均为非正态分布，采用非参数检验，得到Z=-0.245, p=0.806(p>0.05)，说明治疗前两组VAS评分无统计学差异，可比。见表五。

表五：两组治疗前VAS评分比较（X±S）

组别	N	平均值	Z	P
治疗组	34	7.56±0.12	0.245	0.806
对照组	32	7.47±0.10		

两组第一次治疗后，比较VAS评分，统计结果如下：治疗组VAS评分平均值为5.47，标准差为0.66；对照组VAS评分平均值为6.66，标准差为0.48，且两者均不符合正态分布，经非参数检验得到：Z=-5.933, p=0.019(p<0.05)，有差异，存在统计学意义。且治疗组和对照组组内比较两者p值均小于0.01。见表六。

表六：两组第一次治疗后VAS评分比较（X±S）

组别	N	治疗前	治疗一次后	组内p	组间p
治疗组	34	7.56±0.12	5.47±0.66	< 0.01	0.019
对照组	32	7.47±0.10	6.66±0.48	< 0.01	

中国知网 https://www.cnki.net

两组疗程结束当日，比较 VAS 评分，统计结果如下：治疗组 VAS 评分平均值为 1.53，标准差为 0.76；对照组 VAS 评分平均值为 4.59，标准差为 0.84，且两者不符合正态分布，经非参数检验，检验得到：Z=-7.101，p<0.01，有显著性差异。且两组组内比较，经配对 T 检验，两组 p 值均小于 0.01。有意义见表七。

表七：疗程结束当日VAS评分比较（$\overline{X}$±S）

组别	N	治疗前	疗程结束当日	组内p	组间p
治疗组	34	7.56±0.12	1.53±0.76	＜0.01	＜0.01
对照组	32	7.47±0.10	4.59±0.84	＜0.01	

两组治疗后 1 周、1 个月、3 个月随访，治疗组 VAS 评分分别为：1.13、0.81、0.47；标准差分别为 0.66、0.54、0.57；。对照组 VAS 评分分别为：3.72、3.47、2.86；标准差分别为：0.68、0.51、0.49。且统计，所有数据均为非正态分布，经非参数检验，治疗结束后 1 周、1 个月、3 个月组间比较 VAS 评分 Z 值分别为：-7.059、-7.271、-7.241；P 值均小于 0.01，有显著统计学差异，见表八、九、十。

两组患者整个治疗过程的 VAS 评分变化趋势见图四。

表八：疗程结束1周VAS评分比较（$\overline{X}$±S）

组别	N	治疗前	疗程结束1周	组内p	组间p
治疗组	34	7.56±0.12	1.13±0.81	＜0.01	＜0.01
对照组	32	7.47±0.10	3.72±0.64	＜0.01	

表九：疗程结束1月VAS评分比较（$\overline{X}$±S）

组别	N	治疗前	疗程结束1月	组内p	组间p
治疗组	34	7.56±0.12	0.81±0.54	＜0.01	＜0.01
对照组	32	7.47±0.10	3.47±0.51	＜0.01	

中国知网 https://www.cnki.net

表十：疗程结束3月VAS评分比较（$\bar{X}\pm S$）

组别	N	治疗前	疗程结束3月	组内p	组间p
治疗组	34	7.56±0.12	0.47±0.57	＜0.01	＜0.01
对照组	32	7.47±0.10	2.86±0.49	＜0.01	

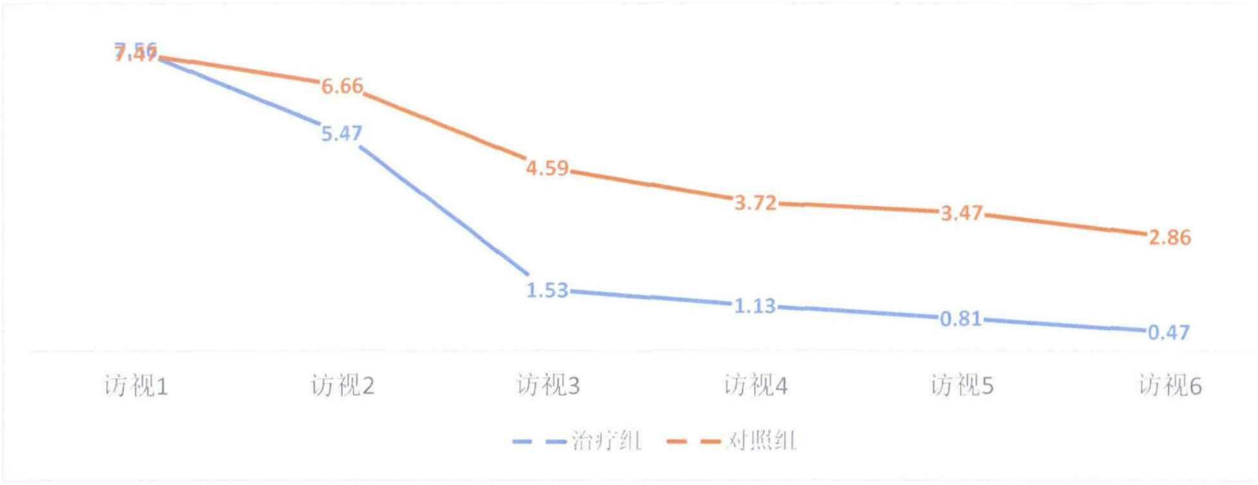

Figure 4 两组 VAS 评分变化趋势

3 肿胀程度

本研究中，治疗前治疗组轻度肿胀 17 例，中度肿胀 12 例，重度肿胀 5 例；治疗前对照组轻度肿胀 16 例，中度肿胀 11 例，重度肿胀 5 例。经非参数检验，Z=-0.035, p=0.972(p>0.05)，两组数据无统计学差异，故可比见表十一及图五。

表十一：两组治疗前肿胀程度比较（非参数检验）

组别	N	无或轻度	中度	重度	Z	P
治疗组	34	17（50%）	12（35%）	5（15%）	-0.035	0.972
对照组	32	16（50%）	11（34%）	5（16%）		

两组疗程结束后当日测量肿胀程度结果如下：治疗组轻度肿胀 6 例，中度肿胀 1 例，重度肿胀 0 例，其余 27 例无肿胀。对照组轻度肿胀 5 例，中度肿胀 2 例，重度肿胀 0 例，其余 25 例无肿胀。经非参数检验，治疗组疗程结束后当日和治疗前对比，组内 Z=-4.364, p＜0.01，具有显著性差异。对照组疗程结束后当日和治疗前对比，经非参数检

中国知网 https://www.cnki.net

验，组内 Z=-3.905,p＜0.01，具有显著性差异。治疗后组间对比结果为：Z=-0.640,p=0.522(p＞0.05),故说明两者结果无统计学差异。见表十二及图6。

表十二：两组治疗前后肿胀程度比较

组别	N		无或轻度	中度	重度	P 治疗前	P 组间	P 组内
治疗组	34	治疗前	17	12	5	0.972	＜0.01	0.522
		治疗后	33	1	0			
对照组	32	治疗前	16	11	5		＜0.01	
		治疗后	30	2	0			

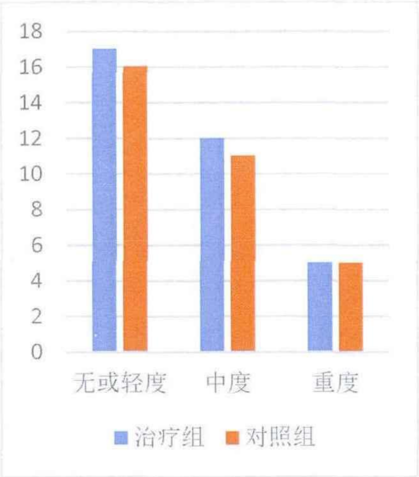

Figure 5 两组治疗前肿胀程度情况

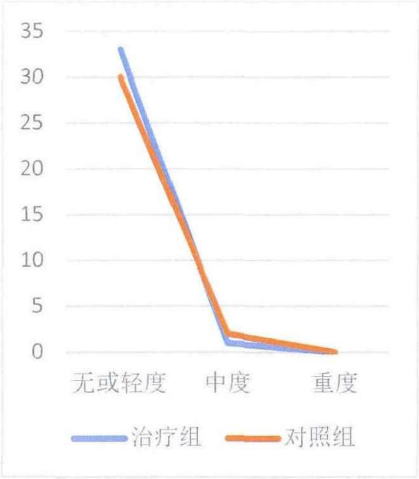

Figure 6 两组治疗后肿胀程度情况

4 美国足与踝关节协会踝与后足功能评分(AOFAS Ankle-Hindfoot Scale)

本研究结果显示，两组患者治疗前两组患者 AOFAS 踝与后足功能评分经正态性检验后，发现治疗组数据不符合正态分布，故采用非参数检验，检验结果示：Z=-1.936，p=0.053(p＞0.05),故两组数据治疗前有可比性。见表十三。

中国知网 https://www.cnki.net

表十三：两组治疗前AOFAS评分比较（$\bar{X}$±S）

组别	N	平均值	Z	P
治疗组	34	35.84±6.11	-1.936	0. 053
对照组	32	33.53±5.42		

本研究结果显示，治疗组治疗前后数据经配对 t 检验得治疗前得分平均值为：35. 84 分，治疗后得分平均值为：82. 53 分；治疗前标准差为：6. 11，治疗后标准差为：3. 23。并且 t=-40. 464，p＜0. 01。有统计学差异。对照组治疗前后数据经配对 t 检验得治疗前得分平均值为：33. 53 分，治疗后得分平均值为：62. 24 分；治疗前标准差为：5. 42，治疗后标准差为：8. 05。并且 t=-16. 870，p＜0. 01。有统计学差异。治疗后治疗组和对照组 AOFAS 评分经正态性检验发现不符合正态分布，故采用非参数检验，得到 Z=-7. 021，p＜0. 01，两者存在显著性差异。具体见表十四。

末次随访与治疗前相比，经配对 t 检验，末次随访治疗组得分平均值为：91. 44；标准差为：3. 17。并且 t=-44. 132，p＜0. 01，有显著性差异。对照组得分平均值为：68. 97；标准差为 8. 15：。并且 t=-20. 668，p＜0. 01，有显著性差异。末次随访治疗组和对照组组间对比，经正态性检验发现不符合正态分布，故采用非参数检验，得到 Z=-7. 012，p 小于 0. 01，两者有显著性差异。具体见表十五。

两组 AOFAS 评分变化趋势见图七。

表十四：两组末次治疗后AOFAS评分比较（$\bar{X}$±S）

组别	N	治疗前	末次治疗后	组内p	组间p
治疗组	34	35.84±6.11	82.53±3.23	< 0.01	< 0.01
对照组	32	33.53±5.42	62.24±8.05	< 0.01	

中国知网 https://www.cnki.net

表十五：两组末次随访后AOFAS评分比较（$\overline{X}$±S）

组别	N	治疗前	末次治疗后	组内p	组间p
治疗组	34	35.84±6.11	91.44±3.17	< 0.01	< 0.01
对照组	32	33.53±5.42	68.97±8.15	< 0.01	

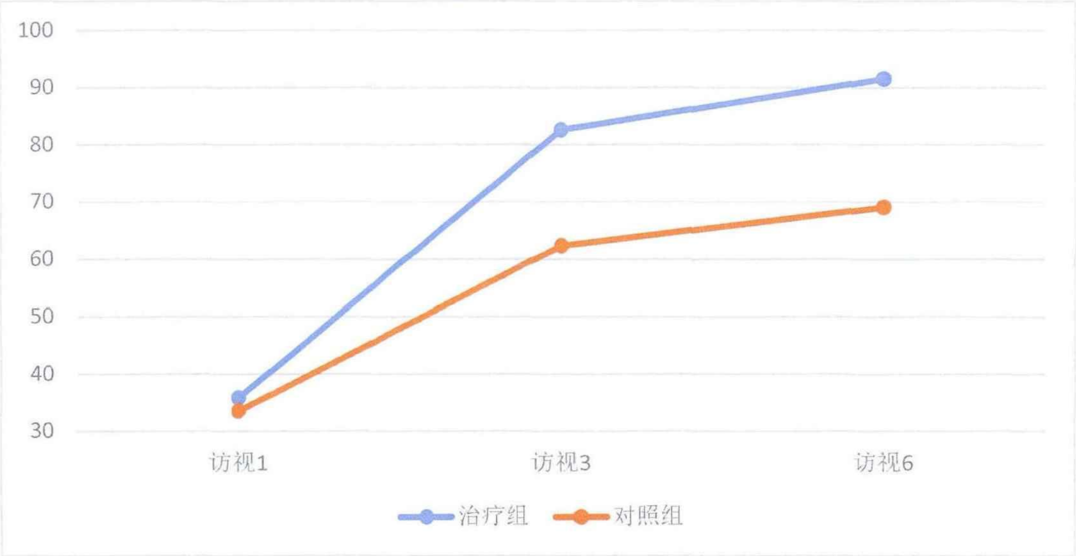

Figure 7 两组患者AOFAS评分变化趋势

5 距骨倾斜角

通过正态性检验，治疗组和对照组患者治疗前，两组数据符合正态性分布(p1=0.638, p2=0.099)，故采用独立样本t检验，检验结果示：t=1.781, p=0.08(p＞0.05)，故治疗前治疗组合对照组距骨倾斜角数据无差异，有可比意义。治疗前，治疗组距骨倾斜角平均为：6.25，标准差为：1.38；对照组距骨倾斜角平均为：5.63，标准差为：1.43。见表十六。

表十六：两组治疗前距骨倾斜角比较（$\overline{X}$±S）

组别	N	平均值(度)	t	P
治疗组	34	6.25±1.38	1.781	0. 08
对照组	32	5.63±1.43		

末次治疗后，治疗组距骨倾斜角平均值为：6.23，标准差为：1.39。对照组距骨倾斜角平均值为：5.62，标准差为：1.43。治疗组治疗前后组内比较，经配对t检验，得

中国知网 https://www.cnki.net

到 t=1.469，p=0.151(p＞0.05)，故无差异。对照组治疗前后组内比较，经配对 t 检验，得到 t=0.991，p=0.329(p＞0.05)，故无差异。

治疗后，治疗组合对照组距骨倾斜角组间比较，经正态性检验治疗组 p=0.758(p＞0.05)，对照组 p=0.198(p＞0.05)，故符合正态分布，采用独立样本 t 检验，得到 t=1.744, p=0.086(p＞0.05)，故两者无差异。具体见表十七图八。

表十七：两组末次治疗后距骨倾斜角比较（X±S）

组别	N	治疗前（度）	末次治疗后（度）	组内p	组间p
治疗组	34	6.25±1.38	6.23±1.39	0.151	0.086
对照组	32	5.63±1.43	5.62±1.43	0.329	

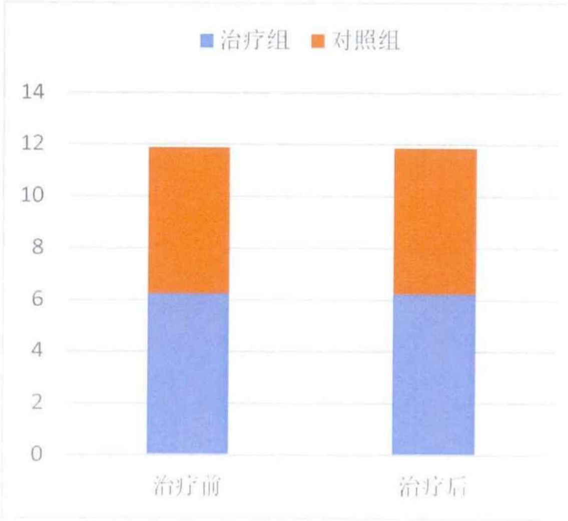

Figure 8 两组患者治疗前后距骨倾斜角情况

6 PANAS-X 具体情绪量表

通过正态性检验，两组治疗前 PANAS-X 量表正性情绪评分不符合正态分布，故选择非参数检验，统计得 Z= 0.777, p=0.437（p>0.05），两组无差异，可比。治疗组均值：25.94 分，标准差为：0.64。对照组均值为：24.84 分，标准差为：0.36。见表十八。

通过正态性检测，两组治疗前 PANAS-X 量表负性情绪评分同样不符合正态分布，故采用非参数检验，统计得 Z=-1.618, p=0.106(p＞0.05)，无差异，可比。治疗组平均值：32.38 分，标准差为：0.88。对照组均值为：30.75 分，标准差为：0.49。见表十九。

末次治疗后正性情绪方面：见表二十。

(1) 治疗组组内比较：治疗前后正性情绪评分经配对 t 检验，得到：t=-23.926, p<

中国知网 https://www.cnki.net

0.01，两者有显著性差异。末次治疗后正性情绪评分均值为：42.29 分，标准差为：2.91。

② 对照组组内比较：治疗前后情绪评分经 t 检验，得到：t=-17.066，p<0.01，具有显著差异，对照组评分均值为：34.06 分，标准差为：2.93。

③ 治疗组对照组组间比较：经正态性分布验证，发现不符合正态分布，采用非参数检验得到：Z=-6.515，p<0.01，故具有显著性差异。

末次治疗后负性情绪方面：见表二十一。

① 治疗组组内比较：治疗前后负性情绪评分经配对 t 检验，得到：t=20.944，p<0.01，两者有显著差异。末次治疗后治疗组负性情绪评分均值为：11.44 分 ，标准差为：2.16。

② 对照组组内比较：治疗前后负性情绪评分经配对 t 检验，得到：t= t=-17.066，p<0.01，具有显著差异。末次治疗后对照组负性情绪评分均值为：22.25 分，标准差为：2.03。

③ 治疗组和对照组组间比较：经正态性分布验证，发现不符合正态分布，采用非参数检验得到：Z=-6.993，p<0.01，故具有显著性差异。

表十八：两组治疗前正性情绪评分比较（$\overline{X}$±S）

组别	N	平均值	Z	P
治疗组	34	25.94±0.64	-0.777	0.437
对照组	32	24.84±0.36		

表十九：两组治疗前负性情绪评分比较（$\overline{X}$±S）

组别	N	平均值	Z	P
治疗组	34	32.38±0.88	-1.618	0.106
对照组	32	30.75±0.49		

中国知网 https://www.cnki.net

表二十：两组治疗后正性情绪评分比较（X±S）

组别	N	治疗前	治疗后	组内p	组间p
治疗组	34	25.94±0.64	42.29±2.91	< 0.01	< 0.01
对照组	32	24.84±0.36	34.06±2.93	< 0.01	

表二十一：两组治疗后负性情绪评分比较（X±S）

组别	N	治疗前	治疗后	组内p	组间p
治疗组	34	32.38±0.88	11.44±2.16	< 0.01	< 0.01
对照组	32	30.75±0.49	22.25±2.03	< 0.01	

治疗前后正性情绪评分变化趋势见图 9，治疗前后负性情绪评分变化趋势见图 10。

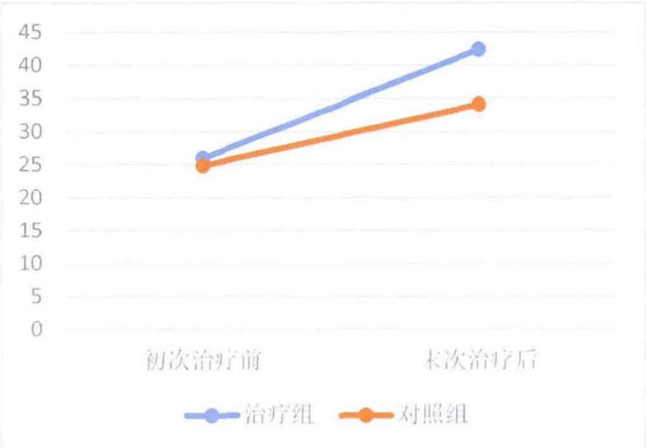

Figure 9 两组患者治疗前后正性情绪变化情况

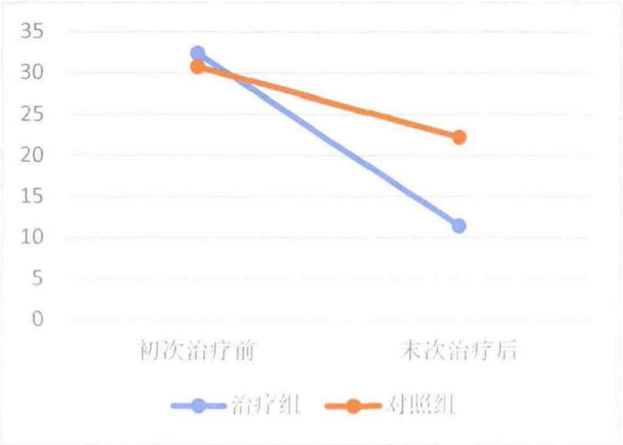

Figure 10 两组患者治疗前后负性情绪变化情况

本研究同时统计了本量表中的害怕、敌意、内疚、悲哀、愉快、自信、关心等七个方面内容。其中害怕情绪包含害怕的、恐惧的、受到惊吓、焦虑的、战战兢兢的、虚弱的；敌意情绪包含愤怒的、易怒的、轻蔑的、厌恶自己的、厌恶（其他的）；内疚的情绪包含内疚的、感到羞愧的、应收谴责的、对自己生气、厌恶自己、不满自己；悲哀的情绪包含伤心的、忧郁的、消沉的、孤独的、寂寞的；愉快情绪包含快乐的、有乐趣的、喜悦的、欢乐的、兴奋的、热情的、活泼的、精力充沛的；自信情绪包含自豪的、坚强的、自信的、冒失的、大胆的、勇敢的；关心情绪包含警觉的、注意的、集中注意力的、坚决的。具体可见下图 11-图 17。

中国知网 https://www.cnki.net

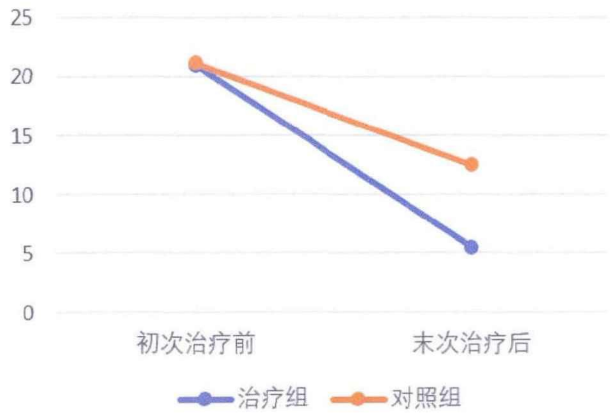

Figure 11 两组患者治疗前后害怕情绪变化情况

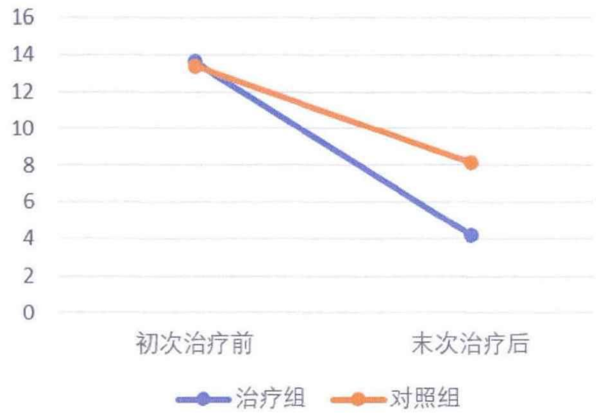

Figure 12 两组患者治疗前后敌意情绪变化情况

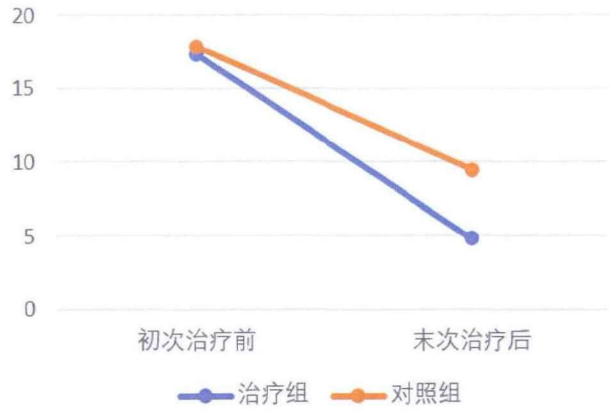

Figure 13 两组患者治疗前后内疚情绪变化情况

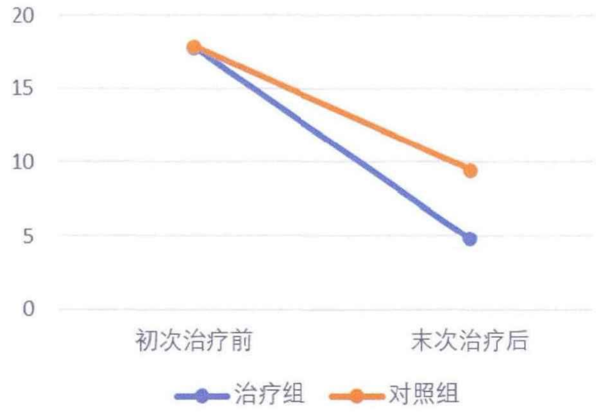

Figure 14 两组患者治疗前后悲哀情绪变化情况

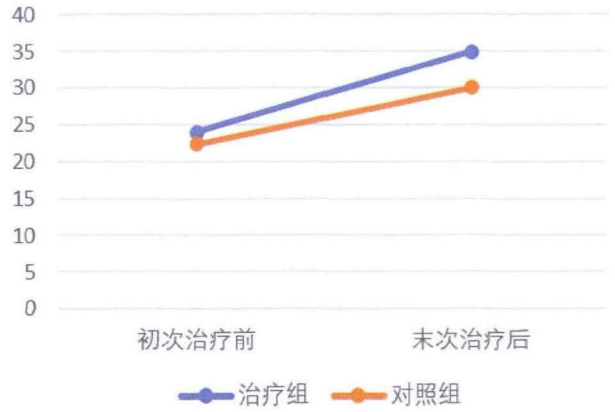

Figure 15 两组患者治疗前后愉快情绪变化情况

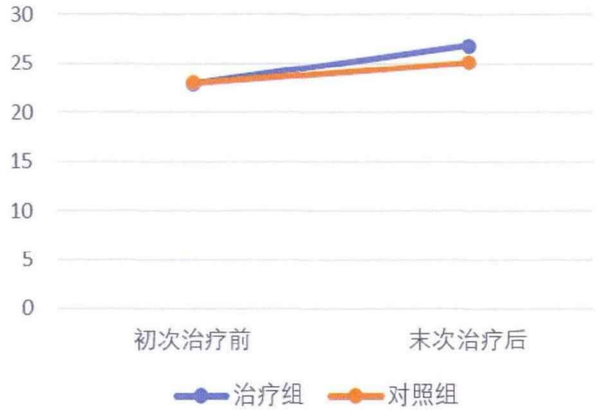

Figure 16 两组患者治疗前后自信情绪变化情况

中国知网 https://www.cnki.net

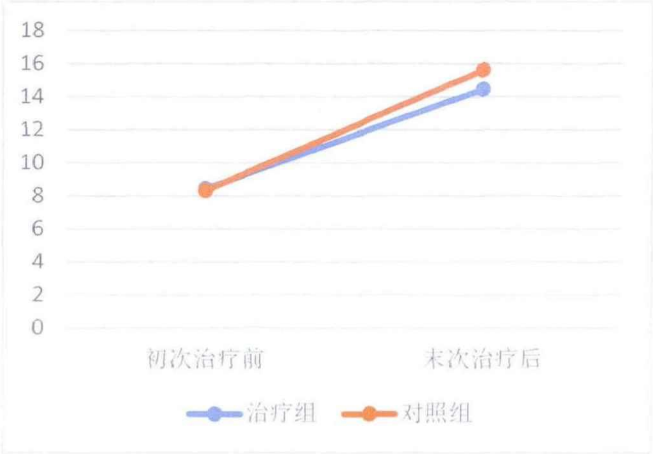

Figure 17 两组患者治疗前后关心情绪变化情况

7 总体疗效评定

本研究结果显示治疗组治疗组治愈 26 例，好转 5 例，未愈 3 例，治愈率 76.5%，有效率 91.1%；对照组治愈 18 例，好转 6 例，未愈 8 例，治愈率 56.3%，有效率 75%。治疗组治愈率和有效率明显高于对照组。

8 不良事件观察

本研究治疗组与对照组治疗观察过程中均未出现明显不良事件。对照组有 1 例患者出现小腿前侧轻微疼痛，考虑为功能锻炼时胫前肌肉略有过度用力，指导患者后症状消失。

中国知网 https://www.cnki.net

讨论

1 陈旧性踝关节扭伤产生的机制

陈旧性踝关节扭伤是临床较为多见，尤其青少年多见，虽然发病率高，但是任然存在医生或患者对此病的重视程度仍然不够的情况[1]。踝关节扭伤多伴随踝关节周围韧带的损伤，踝关节韧带扭伤发病率在全身韧带扭伤中占首位[2]，据统计美国每天踝关节扭伤约发生 23000 例[92][93]。踝关节的功能活动离不开踝关节的稳定，然而踝关节的关节囊比较薄弱，踝关节的稳定主要靠踝关节的韧带来维持，踝关节的稳定踝关节扭伤后，如果不能及时有效的治疗，常遗留病痛，多会反复扭伤，迁延日久，形成踝关节不稳，严重者形成踝关节骨性关节炎，导致不可逆的病理变化，严重影响人们的生活和工作。

本病发病率较高与踝关节的解剖特点及其功能特点有一定的关系。

解剖特点方面，踝关节的骨性结构、踝关节内侧副韧带、外侧副韧带以及踝周的其他软组织都对踝关节的稳定性起到一定的作用，当踝关节相关解剖结构发生骨折、韧带断裂等损伤时，必然导致踝关节不稳定出现，此时行动过程中极易再次发生踝关节扭伤，从而恶性循环，逐渐导致陈旧性踝关节扭伤的产生。

功能特点方面，临床中存在许多不伴有骨折、韧带断裂等器质性损伤的陈旧性踝关节扭伤的患者，此类患者多存在行走活动时对自身踝关节稳定性的不信任感。有学者认为踝关节囊或踝关节周围韧带上存在着机械感受器，该病所表现出的症状即为机械性感受器受损或缺乏导致的[94]。本研究所收集的病例均为陈旧性踝关节扭伤后产生功能性踝关节不稳定症状的患者，并且通过检测指标发现，中医外踝理筋手法对功能性踝关节不稳定患者的症状有明显的治疗意义。近年学者的研究发现，单侧踝关节不稳定，会导致对侧踝关节疼痛等症状[95]。这可能与患者行走时对患侧缺乏信任感，自觉或者不自觉地在行走活动的过程中将重心偏向健侧，长时间的非正常步态，导致健侧逐渐出现疼痛。本人认为，对陈旧性踝关节扭伤的后续研究中，可以引入步态分析的技术手段，从而验证上文提到的试验假设，从而更客观的阐明陈旧性踝关节扭伤的健侧出现疼痛的原因。

2 陈旧性踝关节扭伤的发病特点

临床实践中，中医外踝理筋手法治疗陈旧性踝关节扭伤疗效显著，故我设计了本研究，欲客观量化中医手法治疗陈旧性踝关节扭伤的临床疗效，以期为临床治疗陈旧性踝关节扭伤提供一种选择。

通过前期的文献研究，选取了 74 例患者，最终 66 例资料完整。在统计数据中发现，

中国知网 https://www.cnki.net

无论是治疗组还是对照组，女性患者均略多于男性患者，但是统计结果显示，治疗组和对照组组内比较性别无显著性差异。考虑到本研究样本量较小，存在不足以完全反映出该病在性别方面的发病趋势的可能。本研究中的女性患者多于男性的原因可能是女性穿高跟鞋等穿鞋选择多样化，导致踝关节不能得到有效的支撑，从而发病较男性多。

年龄方面，无论是治疗组还是对照组，本组研究中，40 岁以上的患者所占比例较 40 岁以下的患者多占比例较大。本研究中初步反应出了中老年女性患者陈旧性踝关节扭伤发病率较多，而本研究的前期文献整理中显示，踝关节扭伤青少年发病率更高。本人分析认为，产生青少年踝关节扭伤发病率高而中老年女性陈旧性踝关节扭伤发病率高的原因是可能为如下几点：1.本院青少年患者就诊比例较少，青少年特别是少年儿童，多去积水潭就诊或北京儿童医院等有小儿骨科专科的医院就诊，故本研究中很少收集到青少年的病例。2.存在踝关节扭伤后中老年人较青少年更容易损伤踝周的机械性感受器，且损伤后，中老年人较青少年更难恢复。但限于样本量及取样范围的因素，并不能说明陈旧性踝关节扭伤中老年女性发病率最高。

3 中医手法治疗陈旧性踝关节扭伤的疗效

本研究结果发现，中医正骨手法中的外踝理筋手法在治疗陈旧性踝关节扭伤方面疗效显著，具体如下。

VAS 评分统计方面：本研究结果中显示，中医手法治疗组的 VAS 评分从治疗前的；平均 7.56 分下降到治疗后的平均 1.53 分。而对照组从治疗前的平均 7.47 分下降到治疗后的 4.59 分。且经过统计，两者组间比较，治疗前无统计学差异，治疗后存在统计学差异。说明中医手法治疗组在改善陈旧性踝关节扭伤的 VAS 评分方面明显优于功能锻炼对照组。此结果和本人查阅文献及临床实践中的结果相对应。说明中医手法中的外踝理筋手法治疗陈旧性踝关节扭伤疾病中的疼痛方面的疗效是值得认可与值得推广的。

踝关节肿胀程度方面：本研究结果显示，治疗组治疗前后的踝关节肿胀程度明显改善，但是对照组同样出现了治疗前后踝关节肿胀程度明显改善的情况。并且研究结果显示，治疗前两组组间比较踝关节周围肿胀程度没有差异，治疗后，治疗组和对照组两组的组间比较同样没有差异。说明在改善踝关节肿胀方面，中医手法中的外踝理筋手法和功能锻炼疗法都能达到改善局部肿胀的效果，并且两者不存在差异性。说明两者在改善局部血液循环及调畅局部气血运行方面有相似疗效。

AOFAS 评分方面:治疗前治疗组和对照组 AOFAS 评分没有统计学差异,具有可比性。治疗后，治疗组的 AOFAS 评分由治疗前的平均 35.84 分上升治疗后的平均 82.53 分。说

中国知网 https://www.cnki.net

明中医手法中的外踝理筋手法治疗陈旧性踝关节扭伤在改善踝关节 AOFAS 评分方面有显著的疗效。对照组中，该 AOFAS 评分从治疗前的平均 33.53 分上升到治疗后的 62.24 分，说明功能锻炼对改善踝关节 AOFAS 评分有一定的临床疗效。于此同时，通过治疗后的组间比较不难发现，治疗组的 AOFAS 评分升高明显高于对照组。说明在改善踝关节功能方面，外踝理筋手法的效果明显优于功能锻炼。

距骨倾斜角方面：本研究通过分别测量两组治疗前后的距骨倾斜角发现，组内比较中治疗组和对照组治疗前后距骨倾斜角无显著性改变，且通过统计分析得出治疗前后两组组内比较距骨倾斜角两组数据无统计学差异，即手法组和功能锻炼组均不没有减小距骨倾斜角的作用。产生此种结果的原因说明，中医外踝理筋手法组和功能锻炼组均不能改善踝关节的骨性结构，对机械性的踝关节不稳定不会起到治疗效果。但是实际临床中，陈旧性踝关节扭伤中的单纯的机械性踝关节不稳定十分罕见，其中机械性的踝关节不稳定多同时伴有功能性损伤，故在外踝理筋手法治疗陈旧性踝关节扭伤过程中，多会发现对其症状有不同程度的改善作用。而通过本研究距骨倾斜角的治疗前后测量得知，此症状的改善多是改善了踝关节功能性不稳定，而不是改善了踝关节的机械性不稳定。同时我们进一步得知，当出现踝关节韧带断裂、踝关节骨折等机械性因素时，应该选择手术治疗。

PANAS-X 情绪评分方面：本研究引入了正性负性情绪量表来统计治疗前后患者的情绪变化，以期对实际临床中采用该手法后多数患者正性情绪改变的程度进行一个量化。临床实践中，多数患者在临床症状改善的同时，多有对治疗效果满意度高，情绪由原来的相对低落消极改善为积极乐观。本指标不仅量化了正性情绪，于此同时也量化了负性情绪。本研究发现，治疗组的

临床实践中，往往会遇到患者治疗后情绪有很大改观，故本研究引入了正性负性情绪量表，从而统计治疗前后患者的情绪变化。研究发现，陈旧性踝关节扭伤患者治疗前后负性情绪变化较明显，往往负性情绪相关评分有显著下降，这和临床中陈旧性踝关节扭伤的患者多存在低落情绪、焦虑情绪以及治疗后情绪改善的情况相统一。其中治疗组的结果显示，该组的基本正性情绪评分由治疗前的平均 25.94 分上升到治疗前的 42.29 分；该组负性情绪由治疗前的 32.38 分下降为治疗后的 11.44 分。说明外踝理筋手法治疗陈旧性踝关节扭伤改善其临床症状的同时，有效地改善了患者的情绪，使其正向积极的情绪增加的同时，又明显的减少了其负性消极的情绪。对照组的结果显示，功能锻炼组的正性情绪由治疗前的平均 24.84 分上升为治疗后的 34.06 分；负性情绪评分由治疗

中国知网 https://www.cnki.net

前的平均 30.75 分下降为治疗后的 22.25 分。说明功能锻炼在改善陈旧性踝关节扭伤症状的同时，也起到了改善情绪的作用。但是组间比较，通过统计学分析发现，治疗组无论是在改善正性情绪方面，还是改善负性情绪方面都显著优于对照组。

此结果和中医认为人是一个统一的整体、中医在治疗的过程中是整体论治的观点相符合。

统计结果还发现，在正性情绪方面，愉悦类情绪改善较信心类和关心类改善明显，这和临床患者治疗一段时间后对疗效满意所表现出的明显高兴愉悦相符合。负性情绪方面害怕、内疚、恐惧等情绪都有一定的改善。

从本研究中我认为，治疗疾病不仅仅是针对患者身体疾病的单纯治疗，而应该认同治疗方案对患者情绪治疗的效果以及其临床意义。患者情绪的改善不仅能给患者自身的心理状态带来积极一面，同样情绪的改善会一定程度上促进机体免疫力、抵抗力、康复能力等。这正是中医治疗方法的优越之处。

4 展望

本研究结果显示，中医手法治疗陈旧性踝关节扭伤总体疗效评价方面显示，总体有效率可达 91.1%，并且有操作简单、费用经济、患者接受程度高等优点，值得临床推广使用。

人体是一个有机整体，在面对疾病的治疗过程中，要从整体论治。

本研究的后续研究中，可以将步态分析纳入到治疗前后的检测指标中，因为步态分析可以反映出人体在行进过程中的步态变化以及足部的着力点推进过程，可以借助步态分析来进一步量化中医手法中的外踝理筋手法治疗陈旧性踝关节扭伤的临床疗效。

陈旧性踝关节扭伤临床发病率较高，给患者带来很大的生活不便，严重的患者虽能行走，但处处小心谨慎，严重影响患者的身心健康，我希望通过对该病的不断研究与临床实践，以期更好的治疗该病，为临床医师提供一种治疗陈旧性踝关节扭伤的选择，争取让该病的患者恢复身心健康。

中国知网 https://www.cnki.net

结论

中医手法中的外踝理筋手法治疗陈旧性踝关节扭伤具有显著改善踝关节周围疼痛、消除局部软组织肿胀、改善踝关节自主活动、支撑、步行间距、步行姿态等踝关节功能方面的作用，并有操作简单、费用经济、对患者依从性要求相对较低等优点。值得临床推广。

但是本研究存在样本量不够大，最终收集完整病例 66 份，由于例数有限，未设计空白对照，无法得到更客观的结果。并且远期疗效观察也需要进一步完善与研究。

中国知网 https://www.cnki.net

参考文献

[1] 陈兆军,唐凡启,林顺福,等. 踝关节韧带损伤的早期诊治[J]. 中国骨伤,2007,05:330-331.

[2] 王亦璁.骨关节与损伤[M].北京:人民卫生出版社,2007.1498-1514.

[3] 樊粤光.中医骨伤科学[M].北京:人民卫生出版社，2012.191-192.

[4] 韦以宗.中医骨伤科学辞典[M].北京:中国中医药出版社，2001.4.474-475.

[5] 虞舜，于莉英.四库全书黄帝内经素问[M].南京:江苏科学技术出版社，2008.1.228-229.

[6] 虞舜，于莉英.四库全书黄帝内经灵枢经[M].南京:江苏科学技术出版社，2008.01.48-52.

[7] 牛兵占.中医经典通释黄帝内经[M].石家庄:河北科学技术出版社，1994.3.

[8] 马烈光.黄帝内经素问[M].成都：四川科学技术出版社，2008.6.

[9] 丁光迪.诸病源候论校注[M].北京：人民卫生出版社，1992.1

[10] 吴谦著；张年顺等校注.医宗金鉴[M].北京：中国医药科技出版社，2011.8

[11] 陈兆军. 孙树椿教授外踝理筋手法治疗陈旧性踝关节扭伤临床观察及机理初探[D].中国中医科学院,2016.

[12] 高景华.摇拔戳手法治疗陈旧性踝关节扭伤 34 例[J]. 世界中医药,2011,(03):214-215.

[13] 阿伍提・艾克木,李俊海,林留洋. 宫廷正骨手法治疗陈旧性踝关节扭伤疗效观察[J]. 现代中医临床,2016,(01):44-46.

[14] 陈立. 推拿治疗陈旧性踝关节扭伤 38 例[J]. 现代中西医结合杂志,2002,(18):1795.

[15] 乔欣军. 手法治疗陈旧性踝关节扭伤 52 例报告[J]. 中医正骨,1999,(01):26.

[16] 吴山,马友盟,林应强. 挤压法治疗陈旧性踝关节扭伤 47 例[J]. 新中医,2000,(07):31.

[17] 刘照富,张振南. 中药洗药治疗陈旧性踝关节扭伤的临床疗效观察[J]. 中医临床研究,2014,(20):46-47.

[18] 王伟红,高丽丹. 中药熏洗治疗慢性踝关节扭伤 30 例[J]. 黑龙江医学,2002,(03):238.

[19] 杨雅琴. 不同针灸疗法治疗陈旧性踝关节扭伤临床观察[D].广州中医药大学,2015.

[20] 杨春花.针刺配合小针刀治疗陈旧性踝关节扭伤 30 例[J]. 浙江中医杂志,2012,(03):198.

[21] 秦民安,陈建鸿.小针刀治疗陈旧性踝关节扭伤 25 例临床体会[A]. .针刀医学论文精选[C].:,1999:2.

[22] 蔡三金,程传国. 药物电针刀疗法为主治疗陈旧性踝关节扭挫伤 117 例临床疗效观察[J]. 九江医学,2001,(02):82-83.

[23] 阮炳炎.毫火针治疗陈旧性踝关节扭伤 32 例[J]. 中国医药科学,2013,(19):107-108.

[24] 刘海全. 调经筋手法配合中药熏洗治疗陈旧性踝关节扭伤 72 例临床观察[J]. 新中医,2009,(12):62-63.

[25] 陈可飞,李刚. 手法整复配合中药熏洗治疗陈旧性踝关节扭伤 23 例[J]. 实用中医药杂志,2016,(01):27-28.

[26] 刘瑞钦,张光亚,张红纪. 推拿联合中药熏洗治疗陈旧性踝关节扭伤随机平行对照研究[J]. 实用中

中国知网 https://www.cnki.net

医内科杂志,2014,(08):25-27.

[27] 谢君,游富贵.温养手法推拿结合中药熏洗治疗陈旧性踝关节扭伤 30 例[J].国医论坛,2013,(01):25-26.

[28] 马德刚,赵秀华.中药熏洗配合手法治疗陈旧性踝关节扭伤 55 例报告[J].齐齐哈尔医学院学报,2008,(11):1346.

[29] 张世鹏.二乌散熏洗配合手法治疗陈旧性踝关节扭伤 197 例[J].中医药学报,2004,(04):42.

[30] 刘保新,关俊辉,蔡迎峰等. 小针刀配合运动理筋疗法治疗陈旧性踝关节扭伤的临床研究[J]. 辽宁中医杂志,2015,(05):1071-1073.

[31] 王敏,卢振和,陈来,陈筱. 中医药联合臭氧治疗陈旧性踝关节扭伤[J]. 现代中西医结合杂志,2012,(24):2669-2671.

[32] 马美子. 电针结合中药熏洗治疗陈旧性踝关节扭伤 77 例[J]. 中国实用医药,2007,(07):77-78.

[33] 范青红. 詹氏正骨手法配合中药薰药治疗陈旧性踝关节扭伤的临床研究[D].浙江中医药大学,2016.

[34] Pijnenburg A C M, Van Dijk C N, Bossuyt P M M, et al. Treatment of ruptures of the lateral ankle ligaments: a meta-analysis[J]. The Journal of Bone & Joint Surgery, 2000, 82(6): 761-761.

[35] 王玉玺,岳学强,韩琛等. 踝关节韧带解剖学观测[J]. 新乡医学院学报,2009,(06):565-567.

[36] 王正义.足踝外科学[M].北京:人民卫生出版社,2014.12.10-12.

[37] 贾林,李国平,许铮铮,艾康伟. 踝关节损伤研究现状(综述一上)[J]. 体育科学,1996,(06):62-67.

[38] 杨松鹤,杨振军. 构成踝关节各关节面的形态对踝关节稳定性的影响[J]. 承德医学院学报,2005,(01):11-12.

[39] Schuberth J M, Collman D R, Rush S M, et al. Deltoid ligament integrity in lateral malleolar fractures: a comparative analysis of arthroscopic and radiographic assessments[J].

[40] The Journal of foot and ankle surgery, 2004, 43(1): 20-29.

[41] 王晨. 踝关节三角韧带解剖、损伤诊断与动态生物力学研究[D].复旦大学,2014.

[42] Becker H P, Rosenbaum D. Chronic, recurring ligament instability in the lateral ankle[J]. Der Orthopäde, 1999, 28(6): 483-492.

[43] 王金辉,蒋协远,武勇,王满宜. 慢性踝关节外侧不稳定[J]. 中华创伤骨科杂志,2006,(05):468-471.

[44] Bahr R, Pena F, Shine J, et al. Ligament force and joint motion in the intact ankle: a cadaveric study[J]. Knee Surgery, Sports Traumatology, Arthroscopy, 1998, 6(2): 115-121.

[45] Bahr R, Pena F, Shine J, et al. Mechanics of the anterior drawer and talar tilt tests: a cadaveric study of lateral ligament injuries of the ankle[J]. Acta Orthopaedica Scandinavica, 1997, 68(5): 435-441.

[46] Jackson W, McGarvey W. Update on the treatment of chronic ankle instability and syndesmotic injuries[J]. Current Opinion in Orthopaedics, 2006, 17(2): 97-102.

[47] 施建东,翟文亮,庄泽民. 陈旧性踝关节外侧副韧带损伤的治疗[J]. 临床骨科杂志,2010,(04):479.

中国知网 https://www.cnki.net

[48] 杨珍,胡亚哲. 慢性踝关节不稳的诊断与修复[J]. 中国组织工程研究,2014,(09):1434-1440.

[49] Anderson M E. Reconstruction of the lateral ligaments of the ankle using the plantaris tendon[J]. J Bone Joint Surg Am, 1985, 67(6): 930-934.

[50] Hintermann B. Biomechanics of the unstable ankle joint and clinical implications[J]. Medicine and science in sports and exercise, 1999, 31(7 Suppl): S459-69.

[51] Delahunt E. Neuromuscular contributions to functional instability of the ankle joint[J]. Journal of Bodywork and Movement Therapies, 2007, 11(3): 203-213.

[52] Ozeki S, Yasumura K. Ligament injuries in the ankle joint[J]. Current Opinion in Orthopaedics, 1998, 9(3): 24-29.

[53] Takebayashi T, Yamashita T, Minaki Y, et al. Mechanosensitive afferent units in the lateral ligament of the ankle[J]. J Bone Joint Surg Br, 1997, 79(3): 490-493.

[54] Cox J S, Hewes T F. " Normal" talar tilt angle[J]. Clinical orthopaedics and related research, 1979, 140: 37-41.

[55] 杨志. 踝关节周围韧带损伤[J]. 广西医学,2004,(04):465-467.

[56] Verhaven E F C, Shahabpour M, Handelberg F W J, et al. The accuracy of three-dimensional magnetic resonance imaging in the diagnosis of ruptures of the lateral ligaments of the ankle[J].

[57] The American journal of sports medicine, 1991, 19(6): 583-587.

[58] 汪学松,邱贵兴,翁习生,高增鑫,翟吉良. 踝关节内外侧韧带损伤的诊断和治疗[J]. 中国矫形外科杂志,2008,(04):269-272.

[59] Farooki S, Sokoloff R M, Theodorou D J, et al. Visualization of ankle tendons and ligaments with MR imaging: influence of passive positioning[J]. Foot & ankle international, 2002, 23(6): 554-559.

[60] 白万山,赵辉,邱晓华,杨述华. 磁共振成像在踝关节韧带损伤诊断中的作用[J]. 国外医学(骨科学分册),2005,(02):112-115.

[61] 李莉,王彦,俞东,王少特. 高频超声在急性踝关节韧带损伤分型诊断中的应用[J]. 武警医学,2006,(11):854-856.

[62] 林发俭,冉维强,黄曼维,王金锐. 踝关节侧副韧带损伤超声检查[J]. 中国医学影像技术,2002,(12):1298-1299.

[63] Balduini F C, Vegso J J, Torg J S, et al. Management and rehabilitation of ligamentous injuries to the ankle[J]. Sports medicine, 1987, 4(5): 364-380.

[64] 马雪,杨珍,胡亚哲. 功能锻炼对慢性踝关节不稳定的临床疗效研究[J]. 华南国防医学杂志,2016,(09):571-573.

[65] Clark V M, Burden A M. A 4-week wobble board exercise programme improved muscle onset latency and perceived stability in individuals with a functionally unstable ankle[J]. Physical therapy in sport, 2005, 6(4): 181-187.

中国知网 https://www.cnki.net

[66] Eils E, Rosenbaum D. A multi-station proprioceptive exercise program in patients with ankle instability[J]. Medicine and science in sports and exercise, 2001, 33(12): 1991-1998.

[67] 李坤,王予彬. 踝关节不稳与本体感觉研究现状[J]. 中国微创外科杂志,2010,(09):851-854.

[68] Yamamoto H, Yagishita K, Ogiuchi T, et al. Subtalar instability following lateral ligament injuries of the ankle[J]. Injury, 1998, 29(4): 265-268.

[69] Baltopoulos P, Tzagarakis G P, Kaseta M A. Midterm results of a modified evans repair for chronic lateral ankle instability[J]. Clinical orthopaedics and related research, 2004, 422: 180-185.

[70] De Vries J S, Krips R, Blankevoort L, et al. Arthroscopic capsular shrinkage for chronic ankle instability with thermal radiofrequency: prospective multicenter trial[J]. Orthopedics, 2008, 31(7).

[71] Acevedo J I, Mangone P. Ankle instability and arthroscopic lateral ligament repair[J]. Foot and ankle clinics, 2015, 20(1): 59-69.

[72] Brostrom L.Sprained ankles.Part [illegible].Clinical observations in recent ligament reputures.Acat Chir Scand.1965,130:560

[73] Giza E, Nathe R, Nathe T, et al. Strength of bone tunnel versus suture anchor and push-lock construct in Broström repair[J]. The American journal of sports medicine, 2012, 40(6): 1419-1423.

[74] De Vries J S, Krips R, Blankevoort L, et al. Arthroscopic capsular shrinkage for chronic ankle instability with thermal radiofrequency: prospective multicenter trial[J]. Orthopedics, 2008, 31(7).

[75] 徐琦,张立新. 改良 Brostrom 术式治疗慢性踝关节外侧不稳 21 例疗效分析[J]. 中国骨与关节损伤杂志,2011,(02):164-165.

[76] van der Rijt GA.The long-term results of Watson-Jones tenodesis.J Bone Joint Surg(Br),1984,66:371-375.

[77] Morelli F, Perugia D, Vadalà A, et al. Modified Watson-Jones technique for chronic lateral ankle instability in athletes: clinical and radiological mid-to long-term follow-up[J]. Foot and Ankle Surgery, 2011, 17(4): 247-251.

[78] Sugimoto K, Takakura Y, Akiyama K, Kamei S, Kitada C, Kumai T. Long-term results of Watson-Jones tenodesis of the ankle. Clinical and radiographic findings after ten to eighteen years of follow-up. J Bone Joint Surg Am. 1998; 80(11):1587-1596

[79] 周建刚. 改良 Chrisman-Snook 手术治疗慢性踝关节不稳定[J]. 现代医药卫生,2005,(20):2792-2793.

[80] 王正义.足踝外科学[M].北京:人民卫生出版社,2014.12.584-594.

[81] Hua Y, Chen S, Jin Y, et al. Anatomical reconstruction of the lateral ligaments of the ankle with semitendinosus allograft[J]. International orthopaedics, 2012, 36(10): 2027-2031.

[82] Vega J, Golanó P, Pellegrino A, et al. All-inside arthroscopic lateral collateral ligament repair for ankle instability with a knotless suture anchor technique[J]. Foot & ankle international, 2013, 34(12): 1701-1709.

中国知网 https://www.cnki.net

[83] 踝关节扭伤中医诊疗方案，24 个专业 105 个病种中医诊疗方案[S].国家中医药管理局医政司，2011:143-144.

[84] 彭力平.实用骨伤科手册[M].湖南：湖南科技出版社，2009:10.

[85] 方积乾.生物医学研究的统计方法[M].北京：高等教育出版社，2007:06.

[86] Chinn L, Hertel J. Rehabilitation of ankle and foot injuries in athletes[J]. Clinics in sports medicine, 2010, 29(1): 157-167.

[87] 郑筱萸.中药新药临床研究指导原则[M].北京：中国医药科技出版社，2002:5.

[88] 何伟华,黄昌林,李珂,胡书鸿. 慢性外踝不稳定的距骨斜角测量[J]. 西南国防医药,2005,(03):298-300.

[89] Watson D, Clark L A, Tellegen A. Development and validation of brief measures of positive and negative affect: the PANAS scales[J]. Journal of personality and social psychology, 1988, 54(6): 1063.

[90] 黄丽,杨廷忠,季忠民. 正性负性情绪量表的中国人群适用性研究[J]. 中国心理卫生杂志,2003,(01):54-56.

[91] 国家中医药管理局.中医病证诊断疗效标准[S].南京：南京大学出版社，1994:186-187

[92] Pijnenburg A C M, Van Dijk C N, Bossuyt P M M, et al. Treatment of ruptures of the lateral ankle ligaments: a meta-analysis[J].

[93] The Journal of Bone & Joint Surgery, 2000, 82(6): 761-761.

[94] Ozeki S, Yasumura K. Ligament injuries in the ankle joint[J]. Current Opinion in Orthopaedics, 1998, 9(3): 24-29.

[95] Chen H, Li H Y, Zhang J, et al. Difference in postural control between patients with functional and mechanical ankle instability[J]. Foot & ankle international, 2014: 1071100714539657.

中国知网 https://www.cnki.net

致谢

在此由衷地感谢我的导师陈兆军教授。陈教授在学业上指导我认真求学，孜孜进取，培养我严谨的治学态度；在临床实践中培养我客观仔细、治病求因的临床思维，使我对临床治病思路有了深刻的认识；在医德医风方面，陈教授教导我为人谦和，想他人之所想，急他人之所急的大医精诚的精神。陈教授是我终身学习的楷模。

我还要特别感谢北中医三附院骨科李昕宇、祁印泽老师三年来对我的关心和培养，以及吴俊德、马占华、潘旭月师兄对我的精心指导与帮助。

最后感谢我的家人以及朋友对我求学的帮助和支持。

中国知网 https://www.cnki.net

中国知网 https://www.cnki.net
