# Supplementary material for: Efficacy and safety of Tuina (Chinese Therapeutic Massage) for chronic ankle instability: A systematic review and meta-analysis of randomized controlled trials
Source: PLoS One. 2025 Jun 6;20(6):e0321771. doi: 10.1371/journal.pone.0321771 (PMC12143534; doi:10.1371/journal.pone.0321771)
Supplement: S2 File — (ZIP) [file pone.0321771.s004.zip › 6.推拿配合祛瘀止痛散治疗陈旧性踝关节扭伤的临床研究_刘松刚.pdf]

分类号：　　　　　　　　　　　　　　　单位代码：10441

密　级：　　　　　　　　　　　　　　　学　　号：201502206

山东中医药大学

硕士学位论文

中文题目：推拿配合祛瘀止痛散治疗陈旧性踝关节扭伤的临床研究

英文题目：Clinical Study on Tuina combined with Quyu Zhitong Powder in the treatment of Chronic ankle sprain

申请人姓名　刘松刚

入学年月　2015年9月

学科专业　针灸推拿学

指导教师　李华东

学位类型　中医学专业学位

2018年6月15日

中国知网　https://www.cnki.net

中国知网 https://www.cnki.net

提 要

目的：本研究旨在以陈旧性踝关节扭伤患者为观察对象，通过观察推拿配合祛瘀止痛散外敷和单用祛瘀止痛散外敷治疗陈旧性踝关节扭伤的临床疗效（踝关节局部疼痛、活动度的改善程度）对比，进一步探讨推拿配合祛瘀止痛散治疗陈旧性踝关节扭伤的作用机理。

方法：将符合纳入标准的 60 例患者按随机数字表法分为治疗组（推拿配合祛瘀止痛散外敷）和对照组（单纯使用祛瘀止痛散外敷），每组各 30 例。推拿隔日 1 次，每周 3 次，6 次为 1 个疗程；中药外敷每周 6 次，每次 6~8 小时，12 次为 1 个疗程。2 个疗程结束后，对两组患者治疗前后症状体征综合评分，完成疗效评价。

结果：两组患者均取得明显的临床疗效（踝关节局部疼痛、活动度的改善程度方面），推拿配合祛瘀止痛散外敷组和单用祛瘀止痛散外敷组有效率分别为 90%和 76.7%，具有统计学差异（$P<0.05$）。

结论：推拿配合祛瘀止痛散外敷治疗陈旧性踝关节扭伤可以明显改善患者踝关节局部疼痛和活动度的范围，并且比单用祛瘀止痛散外敷治疗本病疗效更加显著。推拿和祛瘀止痛散两种疗法相辅相成，有利于提高临床治疗效果，并且安全可靠，值得推广。

关键词：推拿；祛瘀止痛散；陈旧性踝关节扭伤

中国知网 https://www.cnki.net

Clinical Study on Tuina combined with Quyu Zhitong Powder in the treatment of Chronic ankle sprain

Speciality: Acupuncture and Tuina

Author：Liu Songgang

Tutor：Professor

Abstract

Objective: The aim of this study was to observe the clinical effect of tuina combined with Quyu Zhitong Powder and Quyu Zhitong Powder in the treatment of chronic ankle sprain (local pain of ankle joint). The effect mechanism of tuina combined with Quyu Zhitong Powder in the treatment of chronic ankle sprain was discussed.

Methods: 60 patients who met the inclusion criteria were randomly divided into treatment group (Tuina combined with Quyu Zhitong powder external application group) and control group (30 cases in each group, each group was treated with Quyu Zhitong Powder, 30 cases every other day) and control group (30 cases in each group). Three times a week, six times a week, six times a week, six times a week, six hours and twelve times a course of treatment. After two courses of treatment, the patients in the two groups were evaluated comprehensively before and after treatment, and the symptoms and signs of the two groups were evaluated.

Results: The effective rates of Tuina combined with Quyu Zhitong Powder and Quyu Zhitong Powder were 90% and 76.7%, respectively. There was significant difference between the two groups ($P < 0.05$).

Conclusion: The treatment of chronic ankle sprain with tuina combined with Quyu Zhitong Powder can obviously improve the degree of local pain and motion of ankle joint. Tuina and Quyu Zhitong Powder two kinds of therapy complement each other, which is beneficial to improve the clinical treatment effect, and is safe and reliable, worthy of promotion.

中国知网 https://www.cnki.net

Key words : Tuina; Quyu Zhitong Powder;Chronic ankle sprain

中国知网 https://www.cnki.net

目　录

引　言……1
临床研究……2
1 临床资料……2
1.1 一般资料……2
1.2 病例选择……2
2.研究方法……3
2.1 病例分组……3
2.2 治疗方法……3
3.临床观察……4
3.1 基础资料……5
3.2 观察指标……5
3.3 疗效标准……5
3.4 统计方法……5
3.5 统计与分析……5
3.6 疗效分析……7
讨　论……8
1 祖国医学对陈旧性踝关节扭伤的认识……8
1.1 祖国医学对陈旧性踝关节扭伤的病名及病因病机的认识……8
1.2 祖国医学对陈旧性踝关节扭伤的治疗……8
2 现代医学对陈旧性踝关节扭伤的认识……10
2.1 骨骼……10
2.2 韧带……10
2.3 推拿配合祛瘀止痛散治疗陈旧性踝关节扭伤的原理探讨……11
2.4 推拿的现代医学作用原理……12
2.5 祛瘀止痛散方药分析……13
结　语……15
参考文献……16
综　述……19

中国知网　https://www.cnki.net

附　录......26
致　谢......28

中国知网　https://www.cnki.net

引 言

踝关节，作为人体负重最大的关节之一，极易受损。临床可见于各个年龄段的损伤，但以青壮年多见。多发于青壮年的主要原因是青壮年喜好运动 。多以剧烈无氧运动为主，如足球、篮球等，所以来就诊的患者当中以青壮年居多。本病的发生主要为急性踝关节扭伤后，由于治疗不及时或反复扭伤，导致踝关节扭伤部位肿胀疼痛难消，活动不利，最终发展成为陈旧性踝关节扭伤。

陈旧性踝关节扭伤归于祖国医学“痹证”、“伤筋”的范畴，祖国医学认为本病多是由外伤等因素所导致。踝关节扭伤，局部组织受损，从而引发脉络血溢，影响气血周流。血瘀气滞，从而引发踝部肿胀和疼痛，进而影响踝关节的正常活动。本病多为实证，故治疗以舒筋活血，消肿止痛，理筋整复为准则。现代医学认为踝关节易扭伤大多由于其特殊生理结构所导致。踝关节是人体活动最频繁又是活动范围较大的屈戌关节，踝关节主要靠韧带、肌腱和关节囊保护，其中韧带的主要作用是维持踝关节稳定，由于关节囊较薄弱，同时又缺乏强有力肌肉保护，急性踝关节扭伤后，若治疗不当或医治不及时，损伤的软组织得不到修复，反应一直存在，软组织逐渐增生粘连，血肿吸收不良，渗出物纤维化，血液循环障碍，不利于炎性物质吸收，从而导致慢性无菌性炎症的发生。加上经常性扭伤，踝关节失稳，以上诸因素均可导致本病的发生。

目前，治疗本病的方法很多，中医学和现代医学各有其代表性的治疗方法。祖国医学主要采用针刺、推拿、艾灸、熏洗、刺络放血等外治法进行治疗。现代医学主要采用保守治疗和手术这两种方式。保守治疗多采用口服镇痛消炎药和以超短波为主的物理疗法等。手术主要针对踝关节不稳进行固定和韧带撕裂进行修复等，但是手术效果并不十分明确。在临床上，中医治疗本病的疗效十分突出，推拿配合自拟祛瘀止痛散治疗本病是导师 教授多年临床经验的总结，并且取得显著疗效。推拿和祛瘀止痛散的两者结合，充分发挥舒筋活血、消肿止痛、理筋整复之效，为以后临床治疗本疾病提供一个崭新的方向。

中国知网 https://www.cnki.net

临床研究

1 临床资料

1.1 一般资料

本研究所选择的病例均来自于山东中医药大学附属医院 2017 年 4 月至 2018 年 1 月前来推拿科和骨科门诊就诊的患者，共计 60 例。随机分为对照组和治疗组。

1.1.1 性别

组别	男（例）	女（例）	合计（例）
治疗组	17	13	30
对照组	20	10	30

经卡方检验，χ^2＝0.635，p＞0.05，两组在性别方面无明显差异，具有可比性。

1.1.2 年龄

组别	15-30 岁	31-40 岁	41-65 岁	平均年龄
治疗组	17	9	4	31.50±10.97
对照组	15	10	5	32.27±10.61

经 t 检验，p=0.97＞0.05，两组在年龄方面无明显差异，具有可比性。

1.1.3 病史

组别	3-8 周	9-12 周	大于 12 周	平均病史
治疗组	7	15	8	74.77±22.86
对照组	6	18	6	73.50±20.44

经 t 检验，p=0.44＞0.05，两组在病史方面无明显差异，具有可比性。

1.2 病例选择

1.2.1 陈旧性踝关节扭伤的诊断标准

依据《中医病证诊断疗效标准》[1]和《实用骨伤科手册》[2]制定：

（1）明显外伤史；

（2）踝部肿、痛，关节摩擦音，活动无力，阴雨天、久行加重；

（3）内外踝前下方压痛，内外翻及跖屈、背伸受限；

（4）X 线未见骨折和脱位；

中国知网 https://www.cnki.net

（5）病程在 3 周以上。

注：综合临床、实验室及 X 线检查，满足第（1）（5）条，其余 3 项满足任意 1 项即可诊断为陈旧性踝关节扭伤。

1.2.2 纳入标准

（1）符合上述本病的诊断标准；

（2）年龄 14 岁~65 岁（含 65 岁）；

（3）对本次研究有良好的依从性；

（4）经 X 线检查无骨折病史、经核磁共振检查无踝关节韧带完全断裂者；

（5）既往无接触性皮炎等皮肤病史；

（6）同意参加本次研究，且签署知情同意书。

1.2.3 排除标准

（1）年龄＜14 和＞65；病史＜3 周；

（2）局部皮肤有破损或有伤口感染者；

（3）合并风湿、类风湿、痛风等累及踝关节活动的患者；

（4）合并有严重危及生命的患者；

（5）正在接受其它相关治疗，对本研究相关效应指标有影响的患者；

（6）经核磁共振检查诊断为Ⅲ度韧带损伤（韧带完全撕裂、断裂的患者）。

1.2.4 剔除与脱落标准

（1）依从性差，对本研究的疗效评价有影响的患者；

（2）患者发生过敏等事件，不愿意继续治疗者；

（3）因各种原因中途退出者；

（4）未按规定治疗，无法判定疗效或资料不全等影响疗效判断者；

（5）属于观察对象，但因观察资料不全而影响疗效和安全性判断者。

2. 研究方法

2.1 病例分组

将符合条件的 60 例病例，按照随机数字表法随机分为治疗组（推拿配合祛瘀止痛散外敷）和对照组（单纯使用祛瘀止痛散外敷），每组各 30 例。

中国知网 https://www.cnki.net

2.2 治疗方法

治疗组：推拿配合祛瘀止痛散外敷。

治疗原则：舒筋活血，消肿止痛，理筋整复。

取穴：阿是穴、阳陵泉、解溪、太溪、昆仑、照海。

手法：㨰法、拿法、揉法、摇法、拔伸法、擦法。

（1）推拿操作：

①局部放松：患者取仰卧位，在患侧踝关节下垫大约10cm的填充物，使其充分放松。取适量凡士林均匀涂于患侧施术部位。首先，术者采用以小鱼际为着力点的㨰法施术于足背及踝关节周围及小腿前侧，然后用拇指与其余四指相配合，先拿揉患侧的小腿后侧肌群3~5min，然后用一指禅偏峰推法施术于踝关节周围，重点在其胫腓韧带、外踝的前下方及其跟腱两侧。

②点揉穴位：依次按揉阳陵泉、解溪、太溪、昆仑、照海等穴。仔细寻找患者的压痛点，并用拇指对其压痛的软组织和筋结重点按揉，约5min，对其局部疼痛的韧带按照其走向进行梳理，约3min。

③踝关节拔伸：术者先用右手将患足推向背伸，左手同时顺势将其足跟向下拉伸，并将其保持，接着右手用力向下将足拉向跖屈，即可使踝关节受力，拉宽其关节间隙。

④术者双手握住踝关节如抱球状，按揉踝关节5~6次，行踝关节擦法，以透热为度。

（2）祛瘀止痛散外敷：

祛瘀止痛散是山东中医药大学附属医院推拿科主任李华东教授几十年来治疗陈旧性踝关节的临床经验结晶，在消肿止痛方面疗效极其突出。它主要由血竭、生大黄、生栀子、艾叶炭、三七、川芎、红花等药物组成，诸药研细末。过60目筛。

患者根据踝关节疼痛肿胀面积大小取适量药粉，以温开水调成糊状，均匀的涂抹在患侧踝关节表面，以保鲜膜缠绕包裹，以防药汁四溢，于每晚睡前敷上，次日醒后揭去保鲜膜，以温水洗净药渣，每次外敷6~8小时。

推拿隔日1次，每周3次，6次为一个疗程，中药外敷每周6次，12次为一个疗程，两个疗程后观察疗效。

（3）中药外敷注意事项

中国知网 https://www.cnki.net

如患踝局部皮肤出现过敏情况，可适当缩短外敷时间，改为隔日 1 次，每次不超过 6 小时。

对照组

治疗方法：单纯中药外敷（具体操作同治疗组）

3.临床观察

3.1 基础资料

患者的一般情况如年龄、性别、病程及踝关节扭伤症状、体征轻重程度积分等。

3.2 观察指标

疼痛视觉模拟评分法 (VAS)和Kofoed评分量表[2]见附录。治疗 2 个疗程结束后，对两组患者治疗前后症状体征综合评分，完成疗效评价。

3.3 疗效标准

参照《中医病证诊断疗效标准》、相关文献及其结合临床后拟定

临床治愈：症状体征消失，疗效指数较治疗前减少≥95%。

显效：70%≤症状体征明显改善，疗效指数较治疗前减少＜95%。

有效：30%≤症状体征有所缓解，疗效指数较治疗前减少＜70%。

无效：症状体征无改善或很小，疗效指数较治疗前减少＜30%.

注：计算公式（尼莫地平法）：[（治疗前积分-治疗后积分）/（治疗前积分）]×100%

3.4 统计方法

本研究结束后，用 SPSS22.0 版统计软件对所收集的数据进行分析。计量资料采用均数±标准差（ $\bar{x}\pm s$）表示，计数资料用 χ^2 检验，等级计数资料用 Wilcoxon 秩和检验。p＜0.05 说明两组数据具有统计学意义，p＜0.01 差异具有显著统计学意义。

中国知网 https://www.cnki.net

3.5 统计与分析

治疗前 VAS 评分比较

组别	例数	VAS 评分治疗前	P
治疗组	30	5.23±1.07	0.369
对照组	30	5.00±1.05	

经统计学分析，p=0.369＞0.05。由表中可知，治疗前 VAS 评分无明显差异，具有可比性。

治疗前后两组患者 VAS 评分比较

组别	例数	治疗前	治疗后
治疗组	30	5.23±1.07	2.00±1.02
对照组	30	5.00±1.05	3.00±1.15

由表中可知，在 VAS 评分方面，两组均有疗效，但是治疗组（推拿配合祛瘀止痛散）的疗效明显优于对照组（单纯外敷祛瘀止痛散）。

治疗前两组患者 kofoed 评分量表疼痛表比较

组别	例数	kofoed 疼痛治疗前	P
治疗组	30	17.67±13.88	0.751
对照组	30	18.67±13.06	

经统计学分析，p=0.751＞0.05。由表中可知，治疗前两组患者在 kofoed 评分量表疼痛表比较方面无明显差异，具有可比性。

治疗前后两组患者 kofoed 评分量表疼痛比较

组别	例数	治疗前	治疗后
治疗组	30	17.67±13.88	38.00±11.03
对照组	30	18.67±13.06	31.67±14.04

由表中可知，在 kofoed 评分量表疼痛表比较方面，治疗组和对照组均有疗效，但是其治疗组（推拿配合祛瘀止痛散）的疗效明显优于对照组（单纯外敷祛瘀止痛散）。

治疗前两组 kofoed 评分表活动度比较

组别	例数	kofoed 活动度治疗前	P
治疗组	30	12.90±2.98	0.632
对照组	30	12.60±2.54	

经统计学分析，p=0.632＞0.05。由表中可知，治疗前两组患者在 kofoed 评分

中国知网 https://www.cnki.net

量表疼痛表比较方面无明显差异，具有可比性。

治疗前后两组 kofoed 评分表活动度比较

组别	例数	治疗前	治疗后
治疗组	30	12.90±2.98	16.23±2.58
对照组	30	12.60±2.54	14.07±2.92

由表中可知，在 kofoed 评分表活动度上，治疗组和对照组均有疗效，但是其治疗组（推拿配合祛瘀止痛散）的疗效明显优于对照组（单纯外敷祛瘀止痛散）。

治疗前后两组总有效率

组别	痊愈	显效	有效	无效	总有效率
治疗组	11	9	7	3	90%
对照组	6	8	9	7	76.7%

由表中可知，治疗组总有效率为达 90%，对照组总有效率为 76.7%，经统计学分析，治疗组优于对照组（p＜0.05）。

3.6 疗效分析

两组受试者治疗前后在 VAS 评分和在 kofoed 评分表疼痛、活动度中，两组均有减轻其疼痛，增加其活动度，改善其活动障碍的作用，但是推拿配合祛瘀止痛散的治疗组改善程度更大，疗效更佳。通过多次比较证实了推拿配合自拟祛瘀止痛散比单纯的使用祛瘀止痛散外敷在治疗此病上更具优势。

综上所述，推拿配合祛瘀止痛散和单纯使用祛瘀止痛散外敷治疗陈旧性踝关节扭伤均有疗效，但是推拿配合祛瘀止痛散无论在其疼痛还是活动度方面改善更加明显，疗效更突出，值得广泛推广和应用。

中国知网 https://www.cnki.net

讨 论

1 祖国医学对陈旧性踝关节扭伤的认识

1.1 祖国医学对陈旧性踝关节扭伤的病名及病因病机的认识

陈旧性踝关节扭伤为西医学上的名称，根据其临床表现和病因病机，归于祖国医学“伤筋”、“痹证”范畴。“伤筋”和“痹证”在祖国医学几千年的发展历程中，其相关的病因病机在古代医学著作中有着大量的记载，对其认识非常丰富。

古代医家对于痹证的病因病机记载非常详尽。“痹”最早见于《黄帝内经》。《扁鹊心书•痹病》亦认为，风寒湿气相合，“走注疼痛”，导致“寒邪凑于分肉之内”，谓之白虎历节风，“痹者，合而发病。”经过几千年的沉淀，现代医家对于痹证的认识及定义做出了更加规范的描述，认为痹证是由于风、寒、湿、热等邪气侵袭经络气血，导致肢体关节肌肉等处痛、麻、酸、重或活动不利，甚则形态改变的一种疾病。

“伤筋”一词，最早见于战国时期，出自《足臂十一脉灸经》。中医骨伤学中的筋，涵盖了解剖学中的肌腱、韧带、关节囊等软组织。《圣济总录・伤折恶血不散》认为，因于伤折，动伤经络，血道不得宣通，“瘀结不散，则为肿为痛。”《太平圣惠方•治一切伤折淋熨诸方》指出：“治伤折，踠损蹉跌，筋骨俱伤，黯肿疼痛，无疮口，宜用熨药方。”淋熨主要是指根据中药方剂的组成，把若干药物煎熬好后浇熨于痛处以通和血脉，这和现在所说的中药熏洗外敷非常相似。

综上可知，陈旧性踝关节扭伤的病因病机是外感风寒湿等邪气及跌打损伤等引起踝部筋脉肌肉的损伤，局部气血运行不畅，导致气滞血瘀，经脉痹阻，不通则痛。

1.2 祖国医学对陈旧性踝关节扭伤的治疗

陈旧性踝关节扭伤属实证，总的治疗大法是舒筋活血，消肿止痛，理筋整复。中医学治疗陈旧性踝关节扭伤的方法比较多，主要以中医外治法（针刺、推拿、艾灸、小针刀、拔罐、刺络放血和中药外敷）为主。

对于骨伤科疾病的治疗，中医外治法有其独到的见解，经过几千年的历史沉淀，经过历代医家的发展，治疗体系日臻完善，并且疗效突出，是中医在临床上治疗骨伤科疾病的主要方法。中医外治法能够直接作用于损伤局部，有其独特的优势。

推拿直接作用于损伤局部，以手法治疗本病，增加局部组织痛阈，促进瘀血和炎性物质的吸收，增加肌力改善踝部韧带、关节囊的弹性，松解粘连[3]，促进损伤

中国知网 https://www.cnki.net

组织的修复，恢复踝关节的稳定性。王琳等[4]等采用的推拿手法不但可以改善足部血液流通，还可以促进血液中沉积于足部的滞留物的排泄，并且可以促进组织损伤释放的儿茶酚胺、组织胺等物质的排泄，消除其疼痛。

针刺治疗，在五版教材《针灸治疗学》中也提到踝关节扭伤，则以阿是穴及邻近取穴为主，如阳陵泉、昆仑、绝骨等。针灸大家贺普仁提出的“病多气滞，法用三通”学说[5]可以概括为毫针微通针刺法可以起到行气活血导滞的功效；刺络放血的强通针法可以起到破血祛瘀之效；火针温通针法可以起到温经通络之功效。因此，针刺腧穴，通过疏经通络、活血镇痛的作用，从而取得良好疗效的目的。

灸法，灸法的种类繁多，既有单纯的艾灸，也有将艾灸与其他方法相结合（如温针灸），将灸法的温热作用与艾草的温经散寒的药性相结合作用于损伤部位是本法的主要作用原理。檀占娜[6]利用数据挖掘技术对 1949 年至 2012 年灸法的临床应用病种与特点的研究检索中发现灸法对临床各个病种都有较高的治愈率，其中外科总有效率高达 95.48%；统计文献发现灸法应用治疗腰腿痛频次为 107 次，位居第二位。可见灸法在治疗此病上有独特的优势。

刺络放血，是以特殊针具在损伤部位点刺瘀络，将局部瘀血祛除消散，气血运行得以畅通的一种疗法。此为“瘀血不去，新血不生”。《黄帝内经》一书中详细记载了此疗法。王焘在《外台秘要》也写到“先以针刺螫处出血，然后角之[7]”这是对拔罐和刺络放血联合应用的较早记载。现代医学对其机理进行了更深入的研究后发现刺络放血疗法可以使血液中的微循环中粒线流加速，降低红细泡的聚集，增加其血氧含量[8]，降低血液的黏稠度[9]，从而微循环中的血液瘀滞情况得到改善。

拔罐，罐的材质多种多样，如玻璃制、竹制、陶土制、塑料制。在临床上多采用玻璃罐，利用火罐在损伤局部、阳陵泉等腧穴进行操作，通过其温经通络的作用，能够改善局部微循环，增加局部的血流量[10]，加快代谢，促进炎性物质的吸收，加快组织修复。现代医学对其机理进行了更深入的研究后发现，此疗法可以使其施术的部位痛阈明显提高[11]。

中药外敷，以活血化瘀类中药为主，研末外敷，以醋或者温水为调和剂，在损伤局部治疗，作用更加直接。有研究表明[12]，中药外敷可以改善损伤局部微循环的通透性，减少炎性的渗出，加快损伤局部瘀血的吸收。

小针刀疗法，依据针刀医学原理，使损伤的韧带等软组织粘连处得以松解，恢

中国知网 https://www.cnki.net

复其关节功能[13]，促进损伤部位肉芽组织的转化[14]，增强组织再生能力[15]，从而使疾病得到治愈。

综上所述，祖国医学对于本病的治疗方式颇多，在疾病的不同阶段，采用相对应的治疗手段进行治疗，对症下药，往往取得较好的疗效。

2 现代医学对陈旧性踝关节扭伤的认识

踝关节扭伤是临床上常见的运动损伤类疾病，特别是在经常运动的人群中，占所有运动相关损伤的20%[16]，该病的发生与踝关节特殊的解剖结构以及在人体运动时所承担的作用密切相关。人在运动或行走或上下楼梯踩空，极易发生踝部的扭伤。虽然踝关节扭伤在运动损伤类疾病中所占比例很高，但是在临床上尚未引起足够重视。当发生扭伤后，踝部周围损伤的韧带，影响关节的内外侧平衡，继而造成踝关节的不稳，由于治疗不及时或踝关节反复性扭伤，最终造成陈旧性踝关节扭伤，更为严重的患者还需要进行手术治疗，不仅在身体上造成伤害，也对患者的心理造成一定的影响，严重影响患者的日常生活。由于现在医学界对本病的诊断标准存在较大分歧，因此在治疗方面也存在着争议。

2.1 骨骼

作为人体承重最大的屈戍关节，踝关节由上位的胫腓骨下端和下位的距骨关节面组成，其中腓骨下端骨突、胫骨下端向下及向后的骨突分别称为外踝、内踝及后踝。外踝较内踝相比位置更低。

2.2 韧带

2.2.1 内侧副韧带

内侧副韧带，又称三角韧带，起自内踝下缘，止于跟骨、距骨及足舟骨，呈扇形向下覆盖于足内侧面，坚韧而富有弹性，是保护足内侧缘的唯一韧带。根据其附着部位的不同，分为胫跟韧带、胫舟韧带、胫距前韧带及胫距后韧带。胫跟韧带与胫舟韧带位于内侧副韧带的后方和前方，分别连接于后踝和跟骨内上方以及内踝与舟骨内上方，其中胫跟韧带防止足部向后脱位；胫距前、后韧带分别起于内踝，分别止于距骨颈和距骨后内侧，其中胫距前韧带浅层为舟胫韧带，附于距骨颈后部。以上共同构成内侧副韧带，面积宽广，防止足过度外翻和背屈为其主要作用。

2.2.2 外侧副韧带

外侧副韧带位于足外侧，主要分为距腓前、后韧带和跟腓韧带，三条韧带相互

中国知网 https://www.cnki.net

独立，均起自外踝，分别向前、后、下止于距骨和跟骨。与内侧副韧带相比，面积狭小，力量薄弱。跟腓韧带主要作用是在踝关节 90 度位时防止发生足过度内翻。距腓前韧带可防止踝关节在跖屈状态下的过度内翻，并可对抗距骨在中立位状态下的向前移位。距腓后韧带的主要作用则是防止踝关节发生过度背伸活动。

踝关节主要靠其周围的韧带、肌腱、关节囊的保护，缺乏肌肉和其他软组织的覆盖。背屈、跖屈以及负重是踝关节的基本功能，其关节囊两侧紧而前后松，前后韧带亦较薄弱。由于踝关节特殊解剖结构决定踝关节容易发生内翻扭伤，当发生扭伤时，以外侧副韧带损伤多见，尤其是以距腓前韧带损伤最为常见。

2.3 推拿配合祛瘀止痛散治疗陈旧性踝关节扭伤的原理探讨

2.3.1 推拿的中医作用机理

推拿以手法以及操作部位的特异性而对机体产生整体性调节，进而影响人体的生理、病理进程。概括起来，推拿具有舒筋通络，行气活血，理筋整复，滑利关节等作用。

（1）舒筋通络，行气活血

经络，内属脏腑而调阴阳，外络肢节而利关节，通达表里而行气血，贯穿上下而强筋骨。是人体气血运行的通路。当足踝部强力向内、向外翻扭伤后，踝部气血失和，痹阻经脉，不通则痛。特异性推拿手法直接作用于患处体表，可加快血行，加速血液流通。正如《素问•血气行志》采用按摩醪药以治疗由于“行数惊恐”引起的“不仁”病。现代研究发现，在肌肉收缩过程中,代谢失衡等因素使神经末梢敏感增加从而引发疼痛,强直性的肌肉痉挛会加剧疼痛[17]，而适当的推拿可以提高局部的痛阈，解除肌肉痉挛[18]。经过推拿治疗能量由机械能向热能转换，产生较高的热能，间接加快患处血液流通。现代研究证实[19]推拿手法能使局部毛细血管扩张，加快血液循环，加快患处破损的小血管愈合[20]，活血化瘀，促进血肿及其水肿吸收[21]，推拿手法使踝关节得到适当的锻炼，从而可以加强踝关节周围肌肉和韧带弹性和力量[22]。

（2）理筋整复，滑利关节

筋骨、关节是人体的运动器官。气血调和，人体关节才能滑利。如《灵枢•本藏》所主张的“血和”是“经络流利”、“营复阴阳”、“筋骨劲强”、“关节清利”的首要条件。踝部扭伤后，损伤其周围筋骨，导致络脉血溢，影响气血周流。

中国知网 https://www.cnki.net

继而为肿为痛，必然影响肢体活动。《医宗金鉴•正骨心法要旨》采用按摩法，按经络，摩壅聚，来“通郁闭之气”、“散瘀结之肿”，以治疗跌扑闪挫引起的骨缝开错，气血郁滞等诸肿痛症，印证了手法的理筋整复，滑利关节作用。这体现在三个方面：首先，手法的局部治疗作用可促进气血运行，理气止痛，祛瘀消肿；其次，通过整复力的直接作用起到理筋整复的作用，以纠正骨错缝、筋出槽；最后通过主动性的功法和被动性的手法相辅相成，以松解局部粘连，滑利关节。

2.4 推拿的现代医学作用原理

2.4.1 选穴分析

阿是穴：出自《备急千金要方》，以疼痛局部或者其他反应点作为施术的一类腧穴。尤其擅长治疗瘀血引起的疼痛。李悦芳[23]采用阿是穴放血疗法治疗瘀血疼痛，在毫针疗法的基础上，配合局部刺络放血，可以有效祛除瘀滞的病邪，改善局部气血运行的状况，疏通经络，提高疗效。

阳陵泉：足少阳胆经的合穴。为八会穴之筋会，主要用于治疗全身筋伤、筋痿的病症。闫博景[24]等采用巨刺法，以巨刺健侧阳陵泉为主治疗肩周炎，配合患侧“肩三针”等腧穴，共奏舒筋通络之功。肩周炎和本病同属于筋伤类的疾病，因此阳陵泉穴亦是治疗陈旧性踝关节扭伤的重要腧穴。

昆仑：足太阳膀胱经的经穴。用于治疗踝肿痛等痛症。杨志新[25]采用太溪透昆仑针法治疗急性踝关节扭伤，阮炳炎[26]采用火针刺昆仑、阿是穴等腧穴治疗本病，均取得理想疗效，昆仑穴位于踝关节附近，根据腧穴近治作用特点，昆仑穴具有治疗踝关节附近病变的作用。

照海：足少阴肾经腧穴，八脉交会穴。王子臣[27]等采用丘墟透照海治疗因脑卒中引起的足下垂，有效改善足下垂步态，丘墟透照海，能够运行气血、疏经活络。

解溪：足阳明胃经经穴。主要用于治疗下肢和踝关节疾病。金文燕[28]采用解溪穴傍针刺的疗法治疗足下垂患者，另配合常规针刺方法可以有效改善卒中后足下垂患者的步行能力。

2.4.2 手法分析

摇法：对关节进行被动性的活动，使关节沿着各运动轴，在摇动区位之间做环转运动的手法。本手法重在活动关节，手法操作要和缓，用力要稳，主要作用于关节及其周围的软组织，具有滑利关节、松解粘连的作用。摇法作用于踝关节，有利

中国知网 https://www.cnki.net

于维持其稳定性，增强并且恢复其运动功能。

拔伸法：关节一端固定，术者沿关节纵轴方向施加拉伸力，以增宽关节间隙的手法。本手法重在增宽关节间隙，手法操作动作要稳而和缓，用力要持续而均匀，要用“巧力”，具有疏通滑利、增宽关节间隙的作用。拔伸法作用于踝关节，要求熟练操作，仔细体会踝关节间隙被拉宽的感觉。

拿揉法：将拿法和揉法相结合的一种复合手法。拿揉法在操作时，以拿法为主，揉法为辅，拿法具有疏通经络、行气活血的作用，揉法具有温经通络、活血散瘀的作用。拿法和揉法相结合，作用力更加柔和深透，增强活血的作用。

一指禅推法：以拇指指端、螺纹面着力于治疗部位，以周期性的肘关节屈伸，带动腕关节的往返摆动，使所产生的的功力通过拇指持续不断地作用于治疗部位的一种手法。本手法着力面小、深透性强，具有舒筋活血、疏通经络、消积导滞的作用。

点揉法：是结合点法与揉法的复合手法。本手法属于重刺激手法，作用层次比较深，在操作过程中，力量主要集中在治疗部位上，适用于久痹、陈伤等病症，具有破瘀、止痛之效。

擦法：以手掌掌面、指面及大小鱼际附着在一定部位上，做快速的直线来回运动的手法。本手法摩擦力强，具有明显的温热效应。在运动系统疾病治疗过程中，具有温经止痛、行气活血、消肿散结、蠲痹胜湿等作用

㨰法：以小鱼际及手背尺侧在治疗部位进行连续的滚动的手法。本手法接触面大，平和舒缓，具有舒筋活络之效。

2.5 祛瘀止痛散方药分析

方中艾叶炭、三七、川芎、红花性温，味辛、苦，温可祛寒活血脉，并抑制诸寒药之凝滞，味苦涌泄破瘀血，辛散行血气。其中，艾叶炭，主归肝、脾、肾、经，为温经止血之要药，可暖气血温经脉；三七归肝、胃经，属活血药兼具止血功效，其活血具有化瘀不伤正的特点，止血具有止血不留瘀的特点，为伤科之要药；川芎归肝、胆经，为血中之气药，可活血止痛，行气散瘀；红花归心、肝经，善通利血脉，为瘀滞肿痛要药；生大黄、生栀子味苦性寒，善通脉祛瘀，取其苦泻通经功效。其中生大黄归肝、心包经，生栀子归心、肺、三焦经，走气分而泻火，皆善治瘀血肿痛诸证；血竭性平味甘，归肝经，入血分，可化瘀止血，为伤科要药。

中国知网 https://www.cnki.net

现代药理研究表明，血竭可促进造血恢复[30]、镇痛[31]、抗菌[32]；生大黄有抗炎、抑菌、活血[33]之效，生栀子有镇静、抗菌消炎[34]的作用；艾叶炭有止血、镇痛[35]的作用；三七具有镇痛、止血[36]等作用；川芎有解痉、抗菌、镇静[37]的作用，还可加速骨折局部血肿吸收，促进骨痂形成；红花对中枢神经系统有镇痛、镇静[38]作用。

综上研究，通过中药及推拿两者作用有机结合，加速了扭伤的踝部皮肤对药物有效成分的吸收，清除了逐渐衰退的上皮细胞，缓解了扭伤局部的肌肉痉挛，解除了血管挛缩，减低了肌肉粘滞性，加速了踝关节扭伤部位的血液微循环及淋巴循环，加快扭伤部位的水肿和渗出物的吸收，活跃周围组织的代谢，从而使本病得到有效治疗。

中国知网 https://www.cnki.net

结 语

踝关节扭伤在临床上比较多见。由于大部分急性踝关节扭伤不伴有骨折、脱位，所以往往没有引起患者及临床医生的足够重视，或者由于医疗条件影响，没有得到及时的治疗，从而演变成陈旧性踝关节扭伤，有的甚至还会造成踝关节不稳从而引发踝关节骨性关节炎，给患者身体带来长期疼痛和不便，也在心理上造成一定影响。由于病情的演变，最后不得不进行踝关节融合或踝关节人工关节置换，从而加重了患者的经济负担。踝关节扭伤在中医辨证中属“痹证”、“伤筋”的范畴。中医治疗该病有显著的优势和疗效。在本课题及其大量文献和实验中已经得到证实。推拿配合祛瘀止痛散和单纯的使用祛瘀止痛散外敷在治疗陈旧性踝关节扭伤均有疗效，但是推拿配合祛瘀止痛散无论在其疼痛还是活动度方面改善更加明显，疗效更突出，值得广泛推广和应用。

1 本研究创新点

祛瘀止痛散是导师李华东教授几十年临床经验的总结，再配合其独特的推拿手法，在治疗陈旧性踝关节扭伤病上取得显著的成效。

2 本研究结果

本研究表明无论是推拿配合祛瘀止痛散和单纯的使用祛瘀止痛散外敷在治疗陈旧性踝关节扭伤均有疗效，但是推拿配合祛瘀止痛散无论在疼痛还是活动度方面改善都更加明显，疗效更突出，值得广泛推广和应用。

3 本研究不足

因本次研究受课题经费、人力等各方面条件的限制，临床部分只进行了 2 个量化表 3 个部分的研究，收纳病例人数过少，并未对远期疗效进行观察，使结论具有一定的片面性。且本课题仅是对所纳患者进行疼痛和活动度评分的统计、观察，没有权威的症状体征观察标准和统一的诊断标准，以后可在此方面加强探讨与研究，使结论更加客观。

中国知网 https://www.cnki.net

参考文献

[1] 国家中医药管理局.中医病证诊断疗效标准[Ｍ].南京：南京大学出版社，1994，64-65.

[2]实用骨伤科手册[M].湖南：湖南科技出版社，2009:10.

[3]KOFOED H.Comparison of ankle arthroplasty and arthrodesise prospective series with long term follow-up[J].Foot,1994,(4):6-9.

[4]王若丞.中医推拿的作用[A].中国传统医学手中国传统医学手法研究会第十六届、中港第五届骨伤手法全国学术交流会论文汇编[C].2010 年.

[5]王琳，李心沁，李晓燕.浅论足部推拿作用原理[J].山东中医药大学学报，1998，22（6）：423.

[6]贺普仁. 普仁明堂示三通［Ｍ］. 北京：科学技术文献出版社.2011：15.

[7]贺普仁. 国医大师贺普仁针灸三通法概述［Ｊ］. 上海针灸杂志，2010，29（4)：205-206.

[8]（唐）王焘. 外台秘要〔M〕. 北京：人民卫生出版社，1955. 9

[9]吴峻，沈晓柔.刺血治疗前后微循环变化 33 例对照观察[J].中国针灸，2001，21（9）：42-43.

[10]牛乾,刘立公,梁子钧.刺血过程中血液流变学指标的即时效应[J].上海针灸杂志，2011，30（7）：477-478.

[11]田宇瑛，秦丽娜，张维波. 不同拔罐负压对皮肤血流量影响 的 初 步 观 察[J]. 针刺研究，2007，32（3）：184-185.

[12]辛英，王书萍，房秀凤，等.单次拔罐前后的痛阈及皮温观察[J]. 中华理疗杂志，1994，17（3）：173-174.

[13]顾晶亮.双柏膏外敷治疗跟骨骨折早期软组织肿胀的临床研究[D].新疆：新疆医科大学，2017.

[14]杨米雄，朱胜良，等.小针刀疗法[J].浙江创伤杂志，1994，1（1）：44-47.

[15]赵斌，刘玉倩。小针刀治疗肌肉损伤的组织学和生物力学研究[J].体育科学，2004,9（24）：28.

[16]乔晋琳，王健瑞。针刀疗法对 L3 横突综合征兔血浆血栓素 B2 及 6-酮-前列腺

中国知网 https://www.cnki.net

素水平的影响[J].中国骨伤，2004,17（5）:257.

[17]Gerber JP，Williams GN，Scoville CR，et al.Persistent disability associated with ankle sprains：a prospective eamination of an athletic population[J].Foot Ankle Int，1998,19（10）：653-660.

[18]张春阳,张婕.运动性延迟性肌肉酸痛的研究进展[J].2007，28（5）:398-400.

[19]胡精超，周军.推拿对延迟性肌肉酸痛作用机制的研究进展[J].中国康复医学杂志，2009，24（1）：89.

[20]陆珍千,费季翔，等从血浆与尿中儿茶酚胺和它的代谢产物含量说明推拿的镇痛作用[J].颈腰痛杂志，1994，15（4）:199-200.

[21]朱亚林,李子让.试论特殊按摩手法对早期急性闭合性软组织损伤的治疗[J].体育科学.2001，21（4）：61.

[22]吴贵根，李军，刘川. 推拿按摩治疗踝关节损伤的研究进展［Ｊ］. 光明中医，2010，25（8）：1536-1537.

[23]杨定产.踝关节韧带损伤的治疗与功能恢复训练［Ｊ］.长沙人学学报，2002，16（4）：90-91.

[24]李悦芳.阿是穴放血疗法治疗瘀血疼痛疗效观察[J].青海医药杂志，2017,47（5）：77-78.

[25]闫博景，许军峰，李美玲，等.巨刺阳陵泉治疗肩关节周围炎30例[J].河南中医，2014,34（4）：679-680.

[26]杨志新. 相对穴位的临床应用－太溪、昆仑穴的应用[J].中国临床医生，2003,31（3）：52-53.

[27]阮炳炎.毫火针治疗陈旧性踝关节扭伤32例[J].中国医药科学，2013,03(19):107-108.

[28]王子臣，王声强.丘墟透照海治疗脑卒中足下垂60例疗效观察[J].河北中医药学报，2009,24（3）：40-41.

[29]金文燕，赵军.解溪穴傍针刺治疗卒中足下垂40例临床观察[J].河北中医，2015,37（4）：567-569.

[30]张子龙，梁 奇，谢 月等龙血竭的药效物质基础及经皮渗透的研究进展[J].海峡药学.2017，29（12）：39-41.

中国知网 https://www.cnki.net

[31]陈玉立，陈素，刘向明. 龙血竭镇痛和阻滞神经传导作用的实验研究[J].时珍国医国药，2010，21(10) : 2446.

[32]Luo Y，Dai H F，Wang H，et al. Chemical Constituents from Dragon's Blood of Dracaena cambodiana〔J〕. Chin J Nat Med，2011，9(2) : 112.

[33]魏江存，陈勇，谢臻等.中药大黄炮制品的化学成分及药效研究进展[J].中国药房，2017，28（25）：3569-3574.

[34]李兆星，申洁。毕武等.中国栀子属植物资源及利用研究进展[J].中药材，2017，40（2）：498-503.

[35]瞿燕，秦旭华，潘晓丽.艾叶和醋艾叶炭止血、镇痛作用比较研究[J].中药药理与临床，2005，21（4）：46-47.

[36]徐倩，王巨鑫，邓同乐.三七的现代功用研究概况[J].科技视界，2012，28:17.

[37]王永忠，童树洪.川芎的传统用法与现代药理研究[J].中国药业，2012，21(7)：95-96.

[38]付萍.中药红花的现代药理作用分析及新用[J].世界最新医学信息文摘，2015，15（51）：141.

中国知网 https://www.cnki.net

综 述

陈旧性踝关节扭伤的临床治疗研究概况

1.陈旧性踝关节扭伤的中医药治疗研究进展

踝关节扭伤一般指踝关节在跖屈位状态下的强力内翻，造成相应踝外侧韧带的损伤[1]。急性损伤后，如若处理不当，重视不够，反复损伤，导致韧带松弛，引起踝关节失稳，最终导致陈旧性踝关节扭伤。在踝关节扭伤中，外侧副韧带最易引起损伤。陈旧性踝关节扭伤常伴有患侧的疼痛和肿胀，不少患者在劳累、寒冷、潮湿的状态下，疼痛和肿胀的程度加重。对于陈旧性踝关节扭伤的治疗，中医药疗效更加突出，经济适用。代表性的中医治疗方法如下。

1.1 中药外敷熏洗

中药治疗本病以外敷熏洗为主，中药以伸筋草、透骨草等活血化瘀，以及生川乌草乌、生白芷等温经止痛祛风散寒除湿的中药为主。秦纲[2]拟损伤熏洗液治疗陈旧性踝关节外侧韧带扭伤，损伤熏洗液组成：防风、桂枝、伸筋草、透骨草、灵仙各 30g，制川草乌各 15g，苏木 24g，灵仙 30g，三棱 24g，莪术 24g，当归尾 24g，红花 18g，川牛膝 24g，桑枝 24g，千年健 20g，刘寄奴 20g。一日 3 次，每剂药可反复使用 6 次。结果：治愈率 100%，平均治愈时间为 10.1 天。组成损伤熏洗液的中药以活血化瘀、舒筋通络的中药为主，同时配合熏洗液的热力，能够加速患处气血运行，加快瘀肿消散，有利于致痛物质快速消散和患处组织的修复。刘照富[3]采用中药洗液治疗陈旧性踝关节扭伤，对照组采用吲哚美辛巴布膏，结果显示：治疗组与对照组有效率分别为 93.33%、73.33%，组间比较差异具有显著性。讨论：方药方药组成为当归、透骨草、伸筋草、川芎、五加皮、木瓜、续断、鸡血藤、川牛膝、红花各 24g，生艾叶 12g，具有热、药双重疗效，诸药配伍，热能松弛肌筋，活血通络；药可疏经通络，消肿止痛，祛寒除湿。

1.2 推拿

推拿以手法以及操作部位的特异性而对机体产生整体性调节，进而影响人体的生理、病理进程，以达到舒筋骨、通经络、活血脉、化瘀血、利关节、理筋骨、整错复的作用。高景华[4]运用孙树椿教授传授的摇拔戳手法治疗本病 34 例，治疗 3 次

中国知网 https://www.cnki.net

后和一个月后有效率均为91.2%，并且总体疗效无差异。在研究中指出，摇拔戳手法能够明显改善骨错缝和筋出槽的状况，摇拔戳手法讲究“轻巧柔和”，诸法配合应用，相辅相成，摇晃手法能够使筋归其位，戳按手法能够使关节复位，最终到达通则不痛、踝关节功能恢复的目的。宋贺卫[5]用三步推拿法治疗踝关节扭伤，对照组采用足背伸外翻位包扎固定治疗，结果显示治疗组疗效明显高于对照组，治疗后疼痛评分治疗组明显低于对照组，具有统计学意义。三步推拿法是指首先采取局部按揉、伤处擦法和理筋法，能够有效提高治疗效果，缓解疼痛症状。陈立[6]用推拿治疗陈旧性踝关节扭伤，治疗5次后，38名患者中治愈21例，治疗10次后，治愈28例，显效10例，治疗方法：首先点按舒筋法，其次环摇屈伸法，最后拔伸牵引法和环摇屈伸法，推拿手法环环相扣，点按舒筋法，使局部气血畅通，粘连松解，环摇屈伸法和拔伸牵引法能够使错缝整复，关节滑利，粘连松解，均有利于损伤的踝关节功能恢复。陈立[6]用推拿治疗陈旧性踝关节扭伤，经过5次推拿后，在38名患者当中，有21名患者得到治愈。经过10次推拿后，有28名患者得到治愈，有10名患者有显著疗效。治疗方法：首先点按舒筋法，其次环摇屈伸法，最后拔伸牵引法和环摇屈伸法，推拿手法环环相扣，点按舒筋法，使局部气血畅通，粘连松解，环摇屈伸法和拔伸牵引法能够使错缝整复，关节滑利，恢复其功能。李俊海[7]在正骨手法配合中药熏洗治疗本病的文章中，治疗21天后结果显示：两者方法均有效，推拿治疗本病的疗效远远高于熏洗的疗效。刘汉云[8]用一指禅推法治疗陈旧性踝关节扭伤，一，此法着力面小，压强大，具有通经活络、祛瘀散结之功，一指禅推法配合擦法作用于损伤的踝关节周围，可以化瘀止痛。吴山[9]运用挤压法治疗本病的47例患者，治愈率68.1%也是远远高于对照组34.0%，总有效率100%高于对照组85.1%，采用挤压法主要整复分离的胫腓关节和距骨关节，有利于经过治疗后各个骨关节的紧密结合，同时配合固定法，能够减少后遗症，规避了传统治疗手段的弊端，使疗程更短，方便快捷。王波[10]采用理筋正骨手法结合易化牵伸术治疗本病30例，与对照组采用电磁波治疗仪治疗本病，结果显示：经治疗后，进行比较，各评分标准，两个组和治疗前相比症状均有很大好转，手法组的疗效远远好于对比的仪器组（P＜0.05），中医传统手法和西医手法相结合疗效确切。

1.3 针刺

倪兴平[11]将123例患者分为两组，治疗组针刺以小节穴为主的腧穴，对照组采

中国知网 https://www.cnki.net

用冷敷和药物。方法：治疗组组以针刺扭伤处相对应对侧腕处的小节穴为主，辅以疼痛点。进针方向直刺，得气为度。10 分钟行针一次，同时配合病人主动活动其扭伤部位。行针 4 次，即可出针。对照组则在前 24 小时内用冷敷的物理疗法，24 小时后用热敷，并口服名为独一味胶囊的药物，3 天后若为愈，则改为只服药物加红外线治疗仪。结果显示：治疗组大部分 5min 之内疼痛缓解，其中有 13 例患者 1 次痊愈。经统计学，治疗组的疗效远高于对照组。小节穴出自董氏奇穴，根据全息理论，此穴与手足太阴经想通，故效甚。

段玲[12]将收录的 80 例患者随机分成两组，每组各 40 例患者。治疗组采用针刺阿是穴、丘墟等穴，针法采用泻法，不留针。在扭伤肿胀处放血，血变则止。用自拟消肿膏涂抹扭伤处。对照组武汉体育医院“新伤 1 号”作对比。治疗组的有效率 100%，而对照组则为 95%。结果显示：诸法并用疗效远远大于单一中药外敷。

阮炳炎[13]将 62 例患者随机分为两组，取穴相同。治疗组采用毫火针，直刺，刺入即出。对照组采用普通针刺，留针 30 分钟。2 疗程后观察疗效。结果显示：治疗组只有 1 例患者无效，对照组则有 6 例患者，由此可知，火针的针和灸双重功效高于单纯的针刺。

杨春花[14]将 60 例患者随机分为两组，每组各 30 例。治疗组采用针刺加小针刀疗法。常规针刺；小针刀则是以损伤的韧带施术为主。当处于韧带附着处时，则纵向剥离，反之则横向。对照组则用自配中药煎水熏洗患处。结果显示治疗组 29 例患者有效，对照组 22 例患者有效。

1.4 综合疗法

采用中医综合疗法治疗本病，主要以手法、针刺、灸法和中药外用等疗法的互相结合。楚德升[11]采用推拿配合中药热敷治疗 30 例陈旧性踝关节内翻位扭伤，结果显示：治愈人数高达 26 人，只有一名患者未治愈，总有效率达 96.7%。讨论：推拿手法以点按和增力点压法为主，中药热敷主要采用葛根、秦艽、防风等中药，手法操作主要在其扭伤部位相对较远的肌肉起止点处进行施术，手法操作能够加快损伤组织的血液循环，促进无菌性炎症的消散吸收，软化消除病灶，使痉挛的肌肉得以放松，而中药热敷能够发挥镇痛、软化瘢痕、消除水肿的作用。卢振中[12]用针灸配合刺血疗法治疗陈旧性踝关节扭伤 33 例，结果显示：经治疗后，治疗组愈显率为 93.5%，对照组为 70%。讨论：针刺取穴采用经络辨证和远近配穴法，以阳陵泉、

中国知网 https://www.cnki.net

阿是穴、昆仑、解溪和丘墟为主，同时配合艾火之温，能够行气活血，通络止痛，同时配合患部活动，能够舒筋通络，减少局部粘连。赵媛[13]针刺配合中药电离子导入治疗36例患者，经治疗后，治愈率达77.8%，有效率高达97.2%。邱华平[14]用隔姜灸法结合自我拉伸法治疗36例陈旧性踝关节扭伤患者，结果显示：治愈26例，好转10例，总有效率100%。讨论：局部隔姜灸中生姜含有姜辣素和多种挥发油，艾灸能够温经散寒，生姜和艾灸相结合，能够直达病所，自我拉伸法是患者的主动活动，讲求患者对“度”的把握，一方面可以防止患者再次损伤，另一方面能够松解粘连的软组织，最终实现对疾病的治愈。张爱玲[15]通过针刺配合按推压痛点并结合患者运动治疗踝关节陈旧性损伤，结果显示：治愈63.9%，好转36.1%，无效0.00%，总有效率100%。讨论：针刺对侧外关穴，并透刺至内关穴，行捻转手法平补平泻，行针时配合主动运动踝关节，同时刺激外关和内关，可使气血调和，外关、内关通阳维脉、阴维脉，外关透内关能够通经活络，按推臀部足太阳经循行压痛点，可起到解痉止痛的作用，下病上治，可以减轻患者痛苦，患者更易接受。刘保新[16]小针刀疗法配合运动理筋治疗本病35例，结果显示：治疗组疗效优于对照组，治疗组在关节活动评分和是否复发等方面均优于对照组。讨论：小针刀疗法能够松解因损伤引起的瘢痕黏连，运动理筋疗法中踢足跟运动和踝关节肌筋收缩疗法相结合，患者积极正确主动的运动，能够减缓损伤局部的软组织粘连，加快病情的好转，维持关节的稳定性。王冠军[17]用中药熏蒸联合小针刀疗法治疗陈旧性踝关节扭伤，治疗后观察组Kofoed踝关节功能评分显著高于对照组，压痛VAS评分显著低于对照组。讨论：小针刀疗法能够松解粘练，解痉止痛，配合中药熏蒸，中药的药力和热力能够直达病灶，发挥其作用，两者结合具有较高的临床价值，也为其以后研究本病提供的崭新方向。

2.陈旧性踝关节扭伤的西医治疗研究进展

现代医学对于陈旧性踝关节扭伤的治疗，主要是针对损伤的韧带和踝关节不稳，主要分为保守治疗和手术治疗。保守治疗主要采用力量训练、固定、药物等，相对于手术治疗痛苦小，更易被接受。陈立峰[18]等通过弹性绷带加压固定，并外用止痛消炎膏治疗53例踝副韧带损伤，对照组采用石膏固定，结果显示治疗2周和治疗4周后，治疗组总有效率均高于对照组，随访18个月，治疗组踝关节外侧不稳定发生率15.38%，对照组14.89%，两组比较无显著性差异（P＞0.05）。治疗组的治疗

中国知网 https://www.cnki.net

方法比石膏固定更有优势，同时避免了石膏固定带来的不便。闫亚新[19]等采用本体感觉与肌内效贴相结合的方法治疗慢性踝关节不稳，对照组单纯采用肌内效布贴治疗，观察指标为疼痛程度、踝-后足功能康复情况、踝关节平衡康复情况，治疗后均有统计学意义（P＜0.05）。肌内效贴对踝关节的具有稳定和支持作用，配合进行本体感觉训练，对于维持踝关节的稳定性具有非常重要的作用，加强对姿势和平衡的控制，有利于康复。闫亚新[20]等采用功能性力量训练对大学生慢性踝关节不稳进行康复，对照组采用传统康复方法，结果显示：两组 CAIT 评分、T 型敏捷性测试均有明显改善，且实验组优于对照组（P 均＜0.05），萨金特纵跳测试两组比较无统计学差异（P＞0.05）。功能性力量训练有利于患者肌力和本体感觉的提高，同时练习方式多样，比传统力量训练的效果更好，更易被接受。王亚军[21]等采用外侧副韧带重建手术结合关节镜检查治疗慢性踝关节不稳，常规康复组采用常规康复治疗，结果显示手术组与康复组患者优良率分别为 88.24%、73.53%，差异明显，外侧副韧带重建手术主要针对外侧副韧带明显松弛的患者，配合关节镜检查，可以有效清除骨赘，有效改善慢性踝关节外侧不稳。

目前，陈旧性踝关节扭伤的治疗方法众多，对本病的病因病机的认识比较系统全面，但是治疗方式以被动类手法为主，对于后期功能的恢复缺乏足够的保障，启示我们在本病的诊疗过程中，重视中医和西医相结合，主动和被动相结合，手法和其他疗法相结合，对于解除患者的痛苦，更加迅速有效，同时可以保证患者踝关节的活动度和稳定性的恢复，疗效更加突出。

中国知网 https://www.cnki.net

参考文献

[1]樊粤光.中医骨伤学[M].北京：人民卫生出版社，2012.191-192.

[2]秦纲.损伤熏洗液治疗陈旧性踝关节外侧韧带损伤47例[J].中国民间疗法，2002,10（2）：28.

[3]刘照富，张振南.中药洗药治疗陈旧性踝关节扭伤的临床疗效观察[J].中医临床研究，2014,6（20）：46-47.

[4]高景华，高春雨，孙树椿，等.摇拔戳疗法治疗陈旧性踝关节扭伤34例[J].世界中医药，2011,6（3）：214-215.

[5]宋贺卫.三步推拿法治疗踝关节扭伤的临床分析[J].中国现代药物应用，2013,7（23）：222-223.

[6]陈立.推拿治疗陈旧性踝关节扭伤38例[J].现代中西医结合杂志,2002,11(18),1795.

[7]李俊海，王庆甫，黄沪.正骨手法与中药熏洗治疗陈旧性踝关节扭伤的病例对照研究[J].中国骨伤，2012，25（2）：113-115.

[8]刘汉云.一指禅推法治疗陈旧性踝关节扭伤[J].按摩与导引，2004,20（3）：51.

[9]吴山，马友盟，林应强.挤压法治疗陈旧性踝关节扭伤47例[J].2000,32(7):31.

[10]王波，陈朝晖，程露露，等.理筋正骨手法联合易化牵伸术治疗陈旧性踝关节扭伤30例[J].安徽中医药大学学报，2017,36（2）：38-41.

[11]楚德升，王海燕，牛俊革.手法配合中药热敷治疗陈旧性踝关节内翻位损伤30例[J].河南中医，2013,33（9）：1499-1500.

[12]卢振中.针灸配合刺血疗法治疗陈旧性踝关节扭伤疗效观察[J].上海针灸杂志，2016,35（3）：329-331.

[13]赵媛针刺配合中药电离子导入治疗陈旧性踝关节扭伤36例[J].上海针灸杂志,2010，29（5）：313.

[14]邱华平.隔姜灸结合自我拉伸法治疗陈旧性踝关节扭伤36例[J].浙江中医杂志，2013,48（11）：851.

[15]张爱玲，杨国超，张姝.针刺运动配合按推压痛点治疗陈旧性踝关节扭伤[J].中国针灸，2013，33(2)：116.

[16]刘保新，关俊辉，蔡迎峰，等.小针刀配合运动理筋疗法治疗陈旧性踝关节扭

中国知网 https://www.cnki.net

伤的临床研究[J].辽宁中医杂志，2015,42（5）：1071-1073.
[17]王冠军.中药熏蒸配合小针刀治疗陈旧性踝关节扭伤疗效观察[J]，新中医，2015,47（5）：河南中医，2012,32（5）：:265-266.
[18]陈立峰，王微.弹性绷带固定配合止痛消炎膏治疗踝关节外侧副韧带损伤疗效观察[J].浙江中医杂志，2008,43（1）：43.
[19]闫亚新，杨建全，陈亮，等.本体感觉结合肌内效贴对慢性踝关节不稳的康复作用[J].海南医学，2017,28（15）：2467-2469.
[20]闫亚新，杨建全.功能性训练对大学生慢性踝关节不稳的康复研究[J].中国临床研究，2017,30（2）：245-247.
[21]王亚军，张春芬，封亮亮，等.外侧副韧带重建手术结合关节镜检查治疗慢性踝关节不稳的疗效分析[J].当代医学，2017,23（4）：107-108.

中国知网 https://www.cnki.net

附 录

附表一

随机号： 姓名： 访视时间： 年 月 日

无痛 最痛

0 1 2 3 4 5 6 7 8 9 10

在 VAS 疼痛评分卡标有 10cm 的刻度，两端分别表示无痛和最痛。分别记作 0 和 10。病人依据自己的主观感觉进行评分，研究者记录静止状态下 VAS 疼痛评分。

治疗前

无痛 最痛

0 1 2 3 4 5 6 7 8 9 10

治疗后

无痛 最痛

0 1 2 3 4 5 6 7 8 9 10

评分标准如下：

无疼痛：0，无任何疼痛

轻度疼痛：1~3 微痛，时痛时止

中度疼痛：4~6 痛重，呈持续性

重度疼痛：7~9 痛剧，需服止痛药

极度疼痛：10 痛极，无法忍受

中国知网 https://www.cnki.net

附表二

姓名：　　　　性别：　　　年龄：　　　　编号：

日期：　　年　　月　　日

Kofoed 评分量表

疼痛（满分 50 分，为基本分）		功能（满分为 30 分）	
无疼	50	足趾行走	3
行走开始时疼痛	40	足跟行走	3
行走时疼痛	35	正常节律上下楼梯	6
偶尔负重时都有疼痛	35	单腿站立	6
每次负重时都有疼痛	15	无辅助性行走	6
检查时疼痛或自发疼痛	0	不用骨科足支具	6
活动度（满分 20 分）			
伸＞10°	5	旋后＞30°	3
5-9°	3	15-29°	2
＜5°	1	＜15°	1
屈＞30°	5	旋前＞20°	3
15-29°	3	10-19°	2
小于 15°	1	＜10°	1
负重时外翻＜5°	2	负重时内翻＜3°	2
5-10°	1	4-7°	1
＞10°	0	＞7°	0

（分数低于 70 分者为差；70-74 分为及格；75-80 分为良好；85-100 分为优秀）

中国知网　https://www.cnki.net

致 谢

本课题是在导师李华东教授的指导下完成的，导师不仅在课题设计、实施、论文写作、临床实践、科研能力方面给予指导，也在生活、为人等方面给予教诲。恩师严谨的治学态度、精湛的临床技术、高尚的医德是我学习的榜样，让我终身受益。培育之恩，难以忘怀，在此致衷也感谢！感谢二十多年来辛勤培育我的父母，感谢他们无论在物质还是心理上给与我极大帮助支持，使我积极进取，不断攀升。感谢在本课题研究中无私帮助我的人。

最后向参加评审论文和答辩指导的各位专家表示由衷的感谢！

中国知网 https://www.cnki.net

发表论文

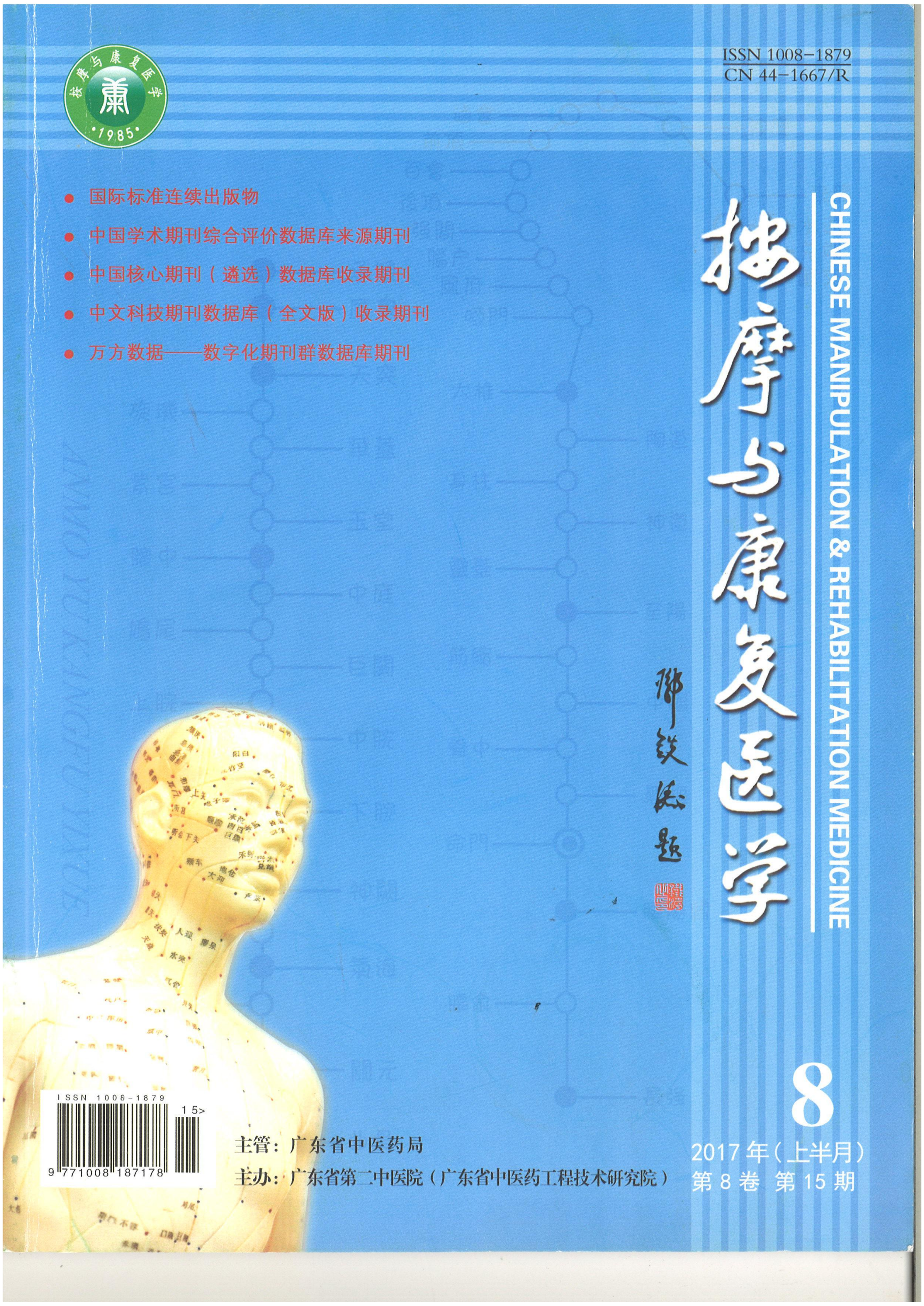

中国知网 https://www.cnki.net

按摩与康复医学

Chinese Manipulation & Rehabilitation Medicine

半月刊　1985年创刊　2017年8月第8卷第15期　Aug.2017 Vol.8 No.15

目　次

●论　著●

蒙特利尔认知评估量表在轻度认知损害诊断中的应用分析*……石义容，胡　慧，王　凌等(1)
侵袭性肺曲霉菌病的诊断研究进展……黄锦焕，陈潮钦(5)
简析血液透析饮食管理APP的开发方向……方坤洋，曾霞芳(7)
创伤后凝血功能障碍机制及临床分析……王光红，黄国军，李张维，等(8)

●医学康复●

放射式冲击波配合针刺治疗网球肘临床疗效观察……刘丽明，冯　前，王　静，等(10)
深层肌肉刺激仪联合电针治疗梨状肌综合征疗效观察……龙贤亮，伍琼英，王　理，等(11)
三伏天穴位贴敷配合中药泡脚治疗冬季手足冰凉60例的临床观察……林　超，高文锦(13)
穴位贴敷联合放松训练对高血压患者睡眠质量的影响……杨　燕，邓筱娟，盛晓萍(15)
表面肌电生物反馈在下背痛患者康复治疗中的应用*……王泽熙，姜贵云(17)
表面肌电在康复医学领域运用及脑卒中患者评定治疗中的应用探讨……任小红(19)
吞咽功能训练配合非营养吮吸对早产儿经口喂养的影响……黄珠莲(22)

●针灸康复●

电针治疗面肌痉挛临床疗效观察……张　威(24)
分步针灸合并理疗加血塞通等针剂静脉输注治疗神经根型颈椎病的临床疗效……欧荣军(25)
空心针治疗外伤后周围型动眼神经麻痹一例……范勇杰，叶光明，高　杨，等(27)

●推拿按摩●

激痛点推拿治疗斜方肌劳损的临床观察……刘松刚，李华东(28)
健翔理筋推拿治疗腰椎髓核摘除术后残余神经症状疗效观察……苏　嘉，李明潭，龙翔宇，等(29)
推拿治疗慢性浅表性胃炎验案……王培超，季　远，李法会，等(32)
推拿手法治疗DPN的机理探讨*……杜正强，石江龙，王可天等(33)

●临床研究●

醒脑静联合纳洛酮治疗脑血管病意识障碍的Meta分析……陈　垚，张　瑾(35)
通冠复脉汤治疗冠心病(CHD)室性早搏对血管内皮功能的影响分析……彭　建，徐　苹，朱　彬，等(39)
米索前列醇经阴道用药在初次妊娠早期无痛人流患者中的效果……黄　静(41)
全腔镜手术与传统手术治疗早期分化型甲状腺癌的临床应用对比分析……张思远，卿伯华，段群欢(43)
40例后路钉棒系统内固定结合后外侧植骨治疗胸腰段脊椎骨折……韩学忠(45)
按摩手法配合三七血竭胶囊治疗血瘀型腰椎间盘突出症的疗效研究……梅中军，张冬生，刘　波(47)
脉冲枪治疗肩袖损伤的临床疗效观察……张　洲，伍　丹(49)
复合乳酸菌对抗生素相关性胃肠病的疗效观察……韦信安(50)
佛山人参再造丸联合康复手法治疗脑卒中后吞咽障碍40例临床疗效观察*……叶宇铁，王宜健(52)

中国知网　https://www.cnki.net

- 28 - 《按摩与康复医学》2017年第8卷第15期 Chinese Manipulation & Rehabilitation Medicine,2017,Vol.8No.15

● 推拿按摩 ●

激痛点推拿治疗斜方肌劳损的临床观察

刘松刚[1],李华东[2△]

(1.山东中医药大学,山东 济南 250355;2.山东中医药大学附属医院,山东 济南 250011)

[摘　要] **目的**:观察推拿治疗斜方肌劳损的临床疗效。**方法**:采用激痛点推拿治疗30例斜方肌劳损患者,施术手法包括㨰法、按揉、拿法、弹拨、叩击等,观察其临床疗效。**结果**:30例患者中,治愈22例、好转6例、未愈2例,总有效率为为93.3%。**结论**:推拿能够缓解斜方肌的痉挛和紧张,从而达到缓解疼痛的治疗目的。

[关键词] 斜方肌劳损;推拿;激痛点;疗效

[中图分类号] R244.1 [文献标识码] B [文章编号] 1008-1879(2017)15-0028-02

随着科技的发展、社会的进步,电脑和手机随处可见,"低头族"、"伏案族"也随处可见;久而久之,颈肩部出现疼痛,更有甚者彻夜难眠,特别是斜方肌区域尤为明显。推拿疗法作为中华传统医学的瑰宝,具有疏通经络、滑利关节、整复止痛的作用,同时具备操作简便、疗效显著、无毒副作用等特点,能够快速缓解肌肉痉挛,从而达到治愈目的。本文采用激痛点推拿治疗30例斜方肌劳损患者取得了满意疗效,现报道如下。

1 资料与方法

1.1 *一般资料* 30例斜方肌患者均来源于2015年10月~2016年10月来我院推拿科就诊的患者,其中男20例、女10例,年龄23~48岁,平均28.3岁,病程3个月~7年,平均1.5年;单侧劳损10例,双侧劳损20例。

1.2 *诊断标准*[1] ①颈肩背部酸胀不适,沉重感,患者头部略向患侧偏歪。②枕外隆凸下稍外部肌肉隆起处压痛,肌纤维变性,弹性减退;颈根部和肩峰之间及肩胛冈上、下缘可触及条索状物,压之酸胀或疼痛,可牵及患肩或患侧头枕部。③固定患肩向健侧旋转患者头项部可引起疼痛。④X线片一般无明显变化,病程长者枕后肌肉在骨面附着处可有骨赘生成。

1.3 *治疗方法* ①患者取坐位,术者站于患者身后方,先用㨰法在斜方肌处来回㨰动,约3~5min,使斜方肌得到放松;②拿揉颈项部及肩部肌肉2~3min,使斜方肌充分放松;③用拇指点压、按揉斜方肌起点处风池、天柱两个穴位和斜方肌止点处肩胛骨内上角阿是穴和曲垣、巨骨等穴位,以酸胀感为度;④找到斜方肌的激痛点,在其激痛点上用拇指弹拨,每处弹拨3~5次;⑤先作颈部屈伸及旋转活动,使颈部充分放松,然后施以颈部斜扳法;⑥最后用小鱼际或空拳叩击项背部,结束治疗。推拿时间约20min,隔日一次。

1.4 *疗效标准* 参照1994国家中医药管理局《中医病证诊断疗效标准》[2]中的相关疗效标准拟定。治愈:颈肩部疼痛消失,活动自如,无复发;好转:颈肩部疼痛减轻,活动时稍有不适;未愈:症状无明显改善。

2 结果

30例患者经过2个疗程的治疗后,治愈22例、好转6例、未愈2例,总有效率为为93.3%。

3 讨论

斜方肌是一块位置表浅但分布面积比较大的肌肉,其位于项部和背部的皮下。根据其肌纤维走向分为三部分[3],其中起于上项线内1/3、枕外隆凸、项韧带的游离缘,止于锁骨外侧1/3后缘的肌束,称为斜方肌上部分纤维;起自第7颈椎到第3胸椎的全部棘突,止于肩峰内侧缘称为斜方肌中部分纤维;第4胸椎到第12胸椎全部棘突于肩胛冈内侧端所形成的三角区域称为斜方肌下部分纤维。因其肌质较薄弱、神经丰富、支配复杂,因此很容易受到损害。随着"低头族"和"伏案族"人数的增加,该病发病率出现明显增高趋势并呈现年轻化。患者颈肩背部酸胀不适,可触及条索状物,更有甚者可出现头晕、头疼,严重影响了人们的身心健康和正常生活。推拿作为以神经、循环、运动等系统的解剖生理学为基础,结合中国的经络和穴位等理论指导手法操作治疗疾病的一门学科,在治疗这类疾病中有得天独厚的优势。美国David G. Simons[4]在其著作中指出了当斜方肌受损时,可找到7个激痛点,其位置分别位于:第一激痛点位于约C_4~C_5横突附近;第二激痛点位于第一激痛点下外侧,约肩胛内上角附近;第三激痛点位于斜方肌下部分纤维的外侧缘;第四激痛点位于

作者简介:刘松刚(1990-),男,在读硕士,研究方向:推拿手法的机理研究。

通讯作者:李华东(1966-),男,博士,主任医师,研究方向:推拿手法的机理研究、推拿治疗伤科及内科疾病的临床研究。

中国知网 https://www.cnki.net
