# Supplementary material for: Efficacy and safety of Tuina (Chinese Therapeutic Massage) for chronic ankle instability: A systematic review and meta-analysis of randomized controlled trials
Source: PLoS One. 2025 Jun 6;20(6):e0321771. doi: 10.1371/journal.pone.0321771 (PMC12143534; doi:10.1371/journal.pone.0321771)
Supplement: S2 File — (ZIP) [file pone.0321771.s004.zip › 7.孙树椿教授外踝理筋手法治疗...关节扭伤临床观察及机理初探_陈兆军.pdf]

中国中医科学院
CHINA ACADEMY OF CHINESE MEDICAL SCIENCES

临床医学博士专业学位论文

DISSERTATION OF DOCTOR OF MEDICINE

孙树椿教授外踝理筋手法治疗陈旧性踝关节扭伤临床观察及机理初探

The clinical observation and discussion on mechanism of Sunshi's manipulation in treating chronic ankle sprain

申 请 人: 陈兆军

专 业: 中医骨伤科学

研究方向: 足踝筋伤疾病

导 师: 孙树椿 教授

2016 年 5 月 18 日

中国知网 https://www.cnki.net

密　级：__________　　　　　　　　单位代码：84502

分类号：__________　　　　　　　　学　　号：B2016055

中国中医科学院

临床医学（中医师承）专业博士学位论文

孙树椿教授外踝理筋手法治疗陈旧性踝关节扭伤临床观察及机理初探

The clinical observation and discussion on mechanism of Sunshi's manipulation in treating chronic ankle sprain

姓　　名：　陈兆军

专　　业：中医骨伤科学

研究方向：足踝筋伤疾病

导　　师：孙树椿教授

中国中医科学院望京医院

2016年 5月　北京

中国知网　https://www.cnki.net

中国知网 https://www.cnki.net

目录

中文摘要 1

ABSTRACT 4

英文缩略语 9

第一部分 文献综述 10

综述一 孙树椿教授学术渊源 10

1 孙树椿教授小传 10

2 孙树椿教授学术源流 11

3 清代上驷院绰班处宫廷正骨的起源和历史沿革 11

4 清代上驷院绰班处学术思想的历史发展过程 12

参考文献 14

综述二 中医骨伤科对踝关节扭伤的认识与治疗 16

1 筋骨相关，筋束骨而利关节 16

2 “筋出槽”、“骨错缝”与踝关节扭伤 17

3 中医手法在治疗踝关节扭伤中的应用 18

4 中医综合疗法在治疗踝关节扭伤中的应用 19

参考文献 20

综述三 现代医学对踝关节扭伤的诊断和治疗进展 22

1 踝关节的解剖特点 22

2 急性踝关节扭伤的认识与诊断 23

3 陈旧性踝关节扭伤的认识与诊断 25

4 急性踝关节扭伤及陈旧性踝关节扭伤的治疗 26

参考文献 33

第二部分孙树椿教授学术思想和临床经验的整理与研究 40

1 中医骨伤科治病的主体是人而不是各个器官，所以治病要求本，以人为主40

2 骨伤科是中医骨伤科，辨证施治是根本，但是也要辨病、辨证相结合 41

中国知网 https://www.cnki.net

3 骨伤科治病首重手法，而手法治疗要寻找“筋结”，并讲究轻、巧、柔、和 42
4 骨伤科疾病首重气血，但瘀血为因、气滞为果，血瘀才是病机根本 43
5 筋伤疾病也讲动静结合，尤其重视功能锻炼的重要性 43
6 手法与药物并重，内外用药结合 44
参考文献 46
第三部分　孙树椿教授外踝理筋手法治疗陈旧性踝关节扭伤的临床研究 47
1. 前言 47
2 资料和方法 48
2.1 病例及课题来源 48
2.2 样本量计算 48
2.3 诊断、纳入及排除标准 48
2.4 治疗方案 49
2.5 随访方法 51
2.6 数据统计 54
3 结果 54
3.1 一般情况 54
3.2 两组患者疼痛评分情况比较 57
3.3 治疗前后 AOFAS 评分情况 57
3.4 患者治疗前后应力位 X 片测量情况 58
3.5 严重不良事件分析 58
4 讨论 58
4.1 踝关节扭伤产生的机制 58
4.2 中医手法治疗踝关节扭伤的优势与不足 60
4.3 孙氏外踝理筋手法的学术特色 61
4.4 展望 63
参考文献 63
第四部分　孙树椿教授外踝理筋手法治疗陈旧性踝关节扭伤的机理初探 66

中国知网 https://www.cnki.net

1.前言 66
2 材料与方法 67
2.1 临床资料 67
2.2 实验仪器与材料 67
2.3 实验方法 68
2.4 症状评定标准 70
2.5 统计学分析 71
3 结果 71
3.1 一般情况 71
3.2 肌骨超声检查结果 71
3.3 足底重心及应力分布的变化比较 73
4 讨论 74
4.1 运用中医理论解释手法作用机制及其局限性 74
4.2 肌骨超声技术在踝关节扭伤中的应用 74
4.3 步态变化与陈旧性踝关节损伤 75
4.4 结语 77
参考文献 78
小　结 80
致　　谢 82
个人简历 83
附录：病例观察表 85

中国知网　https://www.cnki.net

中文摘要

背景：

踝关节扭伤临床非常多见。对于不伴有骨折、脱位的单纯踝关节扭伤，则往往被忽视，由此会演变成为陈旧性踝关节扭伤。陈旧性踝关节扭伤会导致踝关节周围的慢性疼痛、踝关节不稳，最后还可能发生严重的踝关节骨性关节炎，不得不进行关节融合或关节置换，从而产生严重的社会和经济负担。清宫正骨派的代表孙树椿教授运用独特的外踝理筋手法治疗陈旧性踝关节扭伤，临床疗效明显。本研究共分四个部分，第一部分为综述，其中包括孙树椿教授小传及清宫正骨学术源流，中医骨伤科对踝关节扭伤的认识和治疗，现代医学当前对踝关节扭伤的认识与治疗。第二部分为孙树椿教授学术思想和临床经验整理与研究。共有 7 个方面，主要包括孙树椿教授对骨伤科筋伤疾病的认识和治疗疾病首重手法、辨证与辨病结合、手法与用药结合等学术特色以及踝关节外踝理筋手法的渊源与特色。第三、第四部分为运用孙树椿教授清宫外踝理筋手法治疗临床常见病踝关节陈旧性扭伤的临床观察及有关机理初探。

目的：

1.科学观察、评估清宫正骨外踝理筋手法对陈旧性踝关节扭伤的临床疗效，较为客观地验证、判定中医手法治疗临床常见病的有效性，进一步提高踝关节陈旧性扭伤的临床诊治能力。

2.通过肌骨超声技术、足底动态应力分析系统初步探讨清宫正骨外踝理筋手法治疗陈旧性踝关节扭伤的机理。

方法：

1.临床观察：经过筛选符合纳入标准的病例 110 例患者，最终有 89 例符合标准并资料完整，采用随机对照单盲设计方法进行分组，随机分为手法治疗组（简称治疗组）与对照组，其中治疗组 52 例，对照组 37 例。治疗组男 13 例、女 39 例，对照组男 14 例、女 23 例。所有患者踝关节扭伤全部为单侧，且都为内翻损伤，均有不同程度的外踝处疼痛，右侧 66 例，左侧 23 例。扭伤到治疗时间最短者 3 周，最长者 11 月，平均 4.7 月。患者年龄最小者 18 岁，最大者 62 岁。

中国知网 https://www.cnki.net

治疗组进行外踝理筋手法治疗，具体手法要点如下：①手摸心会：在外踝处寻找“筋结”。②手法理筋：轻巧点柔“筋结”，待“筋结”由僵硬变软后以踝关节拔、摇、戳手法理筋。③轻捋收功：最后沿着肌腱韧带走行方向捋顺伤处。

对照组进行功能锻炼治疗，具体包括①足背伸锻炼；②足跖屈锻炼；③踝关节内外翻锻炼；④提踵锻炼等。对两组患者治疗前后进行疼痛学 VAS 评分、AOFAS的 Baird-Jackson 踝关节评分、距骨倾斜角测量等的对比观察。

2.机理初探：上述入组患者中病史 6 个月以上的陈旧性踝关节扭伤患者共 36 例，其中男 14 例，女 22 例，平均 6.3 个月随访时对清宫外踝理筋手法治疗前及治疗后进行如下对比研究。

研究 1：踝关节外侧副韧带肌骨超声检查：对治疗前后的踝关节距腓前韧带、跟腓韧带、距腓后韧带进行高频超声检查，①记录踝关节外侧副韧带的厚度；②观察韧带纤维的连续性；③踝关节运动过程中的韧带张力；④外侧副韧带周围血肿面积；⑤踝关节内积液量等。

研究 2：观察手法治疗前后患者的足底重心分布及其行走步态变化等。

结果：

1.临床结果：本组患者随访`时间最短者 3 月，最长者 2 年，平均 13.6 月。疼痛学评分：组内比较；两组患者治疗后与末次统计疼痛评分均低于治疗前，差异有统计学意义（P<0.05，P＜0.01）；两组患者末次统计疼痛评分与治疗后相比，治疗组呈升高趋势，对照组呈降低趋势，差异有统计学意义（P<0.05）。

组间比较：两组患者治疗前疼痛评分差异无统计学意义（P＞0.05）；治疗后与末次统计，治疗组疼痛评分低于对照组，差异有统计学意义（P<0.05，P＜0.01）。

AOFAS评分情况：两组患者治疗后 AOFAS评分情况相比治疗前呈升高趋势，差异有统计学意义（P<0.05，P＜0.01）。治疗组治疗前 AOFAS 评分情况与对照组差异无统计学意义（P＞0.05）；治疗后治疗组 AOFAS 评分情况高于对照组，差异有统计学意义（P＜0.05）。

应力位 X 片距骨倾斜度：两组患者治疗前后，治疗组与对照组在距骨倾斜度情况方面，差异均无统计学意义（P＞0.05）。

2.机理探讨结果：本组患者 36 例全部得到随访，随访方式均为门诊随访。末次随访到受伤时间平均为 6.3 个月。手法治疗次数平均为 7.8 次，每次治疗不

中国知网 https://www.cnki.net

超过10分钟。

肌骨超声检查：本组病例选择均为踝关节外侧副韧带部分断裂或部分松弛的患者。追踪观察36例踝关节损伤治疗前及治疗后平均6.3个月的情况可见在踝关节各侧副韧带中距腓前韧带是最常损伤、最难恢复的韧带。跟腓韧带、距腓前韧带易同时损伤并损伤程度相仿,但能通过手法保守治疗较快恢复。本组患者损伤可分为挫伤和部分断裂，治疗前所有韧带均连续性好；韧带张力表现为松弛；副韧带周围有面积大小不等的血肿；韧带周围有明显的积液。经手法治疗一段时间后运用业界公认的疗效评价标准-超声进行综合疗效评价。距腓前韧带的有效率为97.2%、跟腓韧带有效率为88.9%、距腓后韧带的有效率为83.3%。并对超声机器所测各韧带厚度值进行统计学分析，清宫外踝手法治疗前后踝关节外侧各副韧带厚度均较治疗前有所恢复，有显著性差异（$P<0.05$）。

足底重心分布及其行走步态变化：本组患者的测试结果显示踝关节陈旧性损伤患者明显有对踝关节外侧不信任感，患足足底外侧压力增加，足底压力中心的连线出现明显外移。测量患足较健足压力中心摆动的距离明显变大。经过手法治疗后患者无论足底压力中心还是重心摆动距离均很快恢复到健侧水平。

结论：

1、运用孙氏“清宫正骨”外踝理筋手法治疗陈旧性踝关节扭伤在缓解疼痛、改善患者踝关节功能方面临床常有立竿见影的明显疗效，优于目前西医常用的踝关节单纯功能疗法锻炼组。

2、“清宫正骨”外踝理筋手法和踝关节功能疗法锻炼均不能改善踝关节的解剖关系，所以对于功能性踝关节不稳定疗效明显，对于机械性踝关节不稳定治疗效果一般。

3、运用“清宫正骨”外踝理筋手法治疗陈旧性踝关节扭伤经过肌骨超声检查证实可以对踝关节外副韧带张力、厚度、消除周围血肿、积液等方面有所改善，并且通过对全踝关节的手法调整对足底重心位移、足部步态等方面有所改善，从而运用现代技术证实了中医手法的临床疗效和机理。

关键词：陈旧性踝关节扭伤；肌骨超声；清宫外踝理筋手法；步态分析。

中国知网 https://www.cnki.net

Abstract

Background:

Ankle sprains are common in clinic. In anterior dislocation of pure ankle sprains, fractures, is often neglected, which would evolve into chronic ankle sprain. Chronic ankle sprains can lead to chronic pain around the ankle and ankle instability, the last may also lead to severe ankle osteoarthritis, that must be joint fusion or joint replacement, causing serious social and economic burden. Professor shu-chun sun's bonesetting method is very effective. This study is divided into four parts. The first part is the review. Including the professor shu-chun sun biographies and bonesetting qing academic origin and traumatology understanding of ankle sprains and treatment, the current understanding of ankle sprains and modern medicine treatmen. The second part is the academic thoughts and clinical experience of professor shu-chun sun. This part of a total of seven aspects. Mainly includes shu-chun sun professor of orthopedics muscle injury disease the understanding and treatment of diseases of the first heavy technique, dialectical and combined disease, technique combined with medication, and academic characteristic and the origin and characteristics of ankle external ankle manage reinforcement technique. The third and the fourth part for our use the professor shu-chun sun external ankle qing manage reinforcement technique to treat clinical common disease clinical observation and the mechanism of chronic ankle sprain.

Objectives:

1.To observe and evaluate the clinical curative effect of external ankle qing manage reinforcement technique.Objectively evaluate and validate the effectiveness of the technique in the treatment of clinical common disease of traditional Chinese medicine.To improve the clinical curative effect of chronic ankle injury(CAS).

2.Through musculoskeletal ultrasound(MUSU) and foot pressure measurement(FPM) system to explore external ankle qing reinforcement technique for the treatment of chronic ankle injury mechanism.

中国知网 https://www.cnki.net

Methods:

1.Clinical observation: Select 110 cases accordance with the inclusive criteria, Eventually 89 cases conform to the standard and have complete information. A random, single-blind parallel study was conducted. 89 cases were randomly divided into treatment group and control group. The treatment group of 52 cases (13 males and 39 females), control group of 37 cases (14 males and 23 females). Patients were unilateral ankle injury, all is the internal injury, has a different degree of external ankle pain. The right side of the 66 cases, 23 cases of the left. History of the shortest is 3 weeks, the longest history is 11 months, an average of 4.7 months. The minimum age 18, maximum age 62 years old. The treatment group specific implementation points: ①With the hand massage, feel with heart: In external ankle place to find and soft light point "tendons knot".②Handle the reinforcement: Wait until " tendons knot" become soft,treating with "Shake、Pull、Poke". ③Brush to close work: Walked along the tendon ligament line direction last sequence channels. In the control group, patients were treated with Functional exercise. Includes the following several aspects.①Dorsalis pedis stretching exerc.②Foot plantar flexion exercise.③Inside and outside the ankle exercise.④Lift heel exercise. Two groups of patients before and after treatment for Visual Analogue Score(VAS), Baird - Jackson ankle American Orthopedic Foot Andankle Society(AOFAS) score, talus slope Angle measurement.

2.Discuss the pathogenesis: A total of 36 patients with history of six months or more（14 males and 22 females）. For external ankle qing manage reinforcement technique as compared before and after treatment. The average follow-up of 6.3 months. Study 1: Musculoskeletal ultrasound ankle joint lateral collateral ligament. For before and after treatment of ankle talofibular ligaments, before with after ligament and talofibular ligaments to high frequency ultrasound. To observe:① Record the thickness of the ankle joint lateral collateral ligament.② Observe the continuity of ligament fiber. ③ Ankle ligament tension in the process of movement.④ The lateral collateral ligament around hematoma area.⑤ In the amount of fluid in the ankle. Study 2: Were observed before and after treatment in

中国知网 https://www.cnki.net

patients with plantar focus distribution and the walking gait changes.

Results:

1.Clinical outcome: Minimum 3 months follow-up time, the longest follow-up for two years。An average of 13.6 months. Visual analogue scale: Intra-group comparison：Two groups of patients after treatment and final statistics Visual analogue scale were lower than before treatment. The difference have statistically significant(P<0.05，P＜0.01); Two groups of patients with final statistics Visual analogue scale shows the following results：The treatment group showed a trend of rise and the control group showed a trend of reduce，The difference have statistical sense（P<0.05）. Comparison between the two groups: No statistical difference was found between two groups of patients before treatment(P＞0.05), After treatment and at the end of the time, the treatment group pain score lower than the control group .The difference have statistical sense(P<0.05，P＜0.01). AOFAS score situation: AOFAS score after treatment in both groups compared with before treatment showed a trend of rise The difference have statistical sense(P<0.05，P＜0.01). Before the treatment, There was no statistically significant difference in treatment group and control group(P＞0.05). After treatment, Treatment group is higher than the control group in AOFAS score, the difference have statistically significant（P＜0.05）. An X-ray stress the talus slope: The treatment group and control group in the aspect of the talus slope do not have statistically significant（P＞0.05）.

2.Pathogenesis of discussion result: 36 patients were followed up, For outpatient follow-up way. The last follow-up to injury time averaged 6.3 months. Number of manipulation treatment with an average of 7.8 times. Treatment time not more than 10 minutes at a time. Musculoskeletal ultrasound: Incidence of ankle part of the lateral collateral ligament rupture or part of a relaxation of the patients.36 cases before and after treatment the average follow-up of 6.3 months. Found the anterior talofibular ligament is the most common injuries. It is the most difficult to cure. It always injuries combined injuries of anterior talofibular ligament and calcaneofibular ligament. Calcaneofibular ligament can be cured by manipulation treatment. This group of patients can be divided into contusion and

中国知网 https://www.cnki.net

part of the fracture. All good continuity ligament before treatment. Ligament tension perfor- mance for relaxation. Ligament with different area around the hematoma. Ligament around has obvious effusion. In the treatment of after a period of time, using the ultrasonic industry recognized comprehensive curative effect evaluation ofcura- tive effect evaluation standard. Its effectiveness is 97.2% for anterior talo-fibular ligaments. 88.9% for calcaneofibular ligament.83.3% for posterior talo- fibular ligament. To various ligament thickness values measured by ultrasound and statistical analysis. Treatment group of thickness of ankle recovered before the treatment. There are significant differences ($P<0.05$). Foot focus distribution and walking gait: Chronic ankle injury in patients with plantar lateral pressure increased significantly. Plantar pressure center offshoring appear. With suff- icient distance with foot-massage center of pressure oscillation significantly bigger. After manipulation treatment in patients with plantar pressure center and center of gravity swing quickly restored to health side level distance.

Conclusions:

1.Sun-style external ankle qing manage reinforcement technique treatment of chronic ankle sprain in pain relief and improve the function of ankle joint clinical curative effect is distinct, treatment and curative effect is superior to the simple function.

2.Because external ankle qing reinforcement technique and ankle function exercise therapy can improve the ankle anatomy relationship, so this technique has a good effect for functional ankle instability, curative effect for mechanical ankle instability.

3.Musculoskeletal ultrasound confirmed: technique improved ligament tension and thickness, the function of eliminating surrounding hematoma and effusion. Sun-style technique improved sole focus displacement and the effect of gait. Use modern technology to prove the clinical curative effect mechanism of the old style of traditional Chinese medicine

中国知网 https://www.cnki.net

Key words:

Chronic ankle sprains;Musculoskeletal ultrasound; Inheritance of traditional Chinese medicine; Qing dynasty palace external ankle reinforcement technique; Center of gravity and the gait analysis

中国知网 https://www.cnki.net

英文缩略语

CAS	Chronic ankle sprains	陈旧性踝关节扭伤
MSUS	Musculoskeletal ultrasound	肌骨超声
FPM	Foot pressure measurement	足底应力测试
VAS	Visual Analogue Score	视觉模拟评分法
AOFAS	American Orthopedic Foot Andankle Society	美国足与踝关节协会

中国知网 https://www.cnki.net

第一部分 文献综述

综述一 孙树椿教授学术渊源

1 孙树椿教授小传

孙树椿，男，1939年7月1日出生，汉族，河北省蠡县人，中国共产党党员。现任中国中医科学院首席研究员、主任医师、博士生导师，中国中医科学院科学技术委员会委员，国家级名老中医，中央保健会诊专家，国家药典委员会委员，国家中医药管理局重点学科(骨伤科)学术带头人，国家中医药管理局重点专科(骨伤科)专科带头人，中华中医药学会副会长，世界中医药学会联合会骨伤专业委员会会长，中华中医药学会骨伤科分会主任委员，北京中医药学会骨伤专业委员会主任委员，全国高等中医院校骨伤科系列教材编委会主任委员、《中国骨伤》杂志副主编、《中国中医骨伤科》杂志主任委员、执行主编、《中医正骨》杂志副主任委员、副主编。是第一批国家级非物质文化遗产“中医正骨”传承人。曾任国家发明奖医药卫生组委员、中国中医研究院骨伤科研究所所长、北京针灸骨伤学院骨伤系主任等职，1993年获国务院政府特殊津贴。

孙树椿老师五十年来一直在骨伤科临床、科研和教学工作的第一线，擅长治疗各种骨伤科疾病，尤其对中医骨伤手法的钻研更深，毕业工作后，得到了北京骨伤名医刘寿山先生的亲授真传，对“宫廷正骨”学派要义体会颇深。成为“清宫正骨流派”的传承人。同时又博采了大江南北诸家名医之长。积极提倡运用中医手法治疗，努力挖掘和发扬祖国传统医学特色，形成具有独具特色的筋伤治疗方法（筋伤手法、专病专方及练功疗法）。指出“筋喜柔不喜刚”，在手法运用上尤其强调轻柔和缓、外柔内刚，使患者在没有痛苦感觉的情况下获得症状的缓解或痊愈。形成了“入其法而又出其法”的独特手法，真正体现了“机触于外、巧生于内、手随心转、法从手出”的正骨手法要旨。在临床上对患者认真负责，除注重影像学诊断外，更注重临床检查，务必在诊断明确基础上方采取治疗，对于骨伤科的一些“疑难疾病”，常有“手到病除”的效果，深受广大患者欢迎，我国骨伤界誉称“国内一把手”、“手上有眼的人”。 如对颈椎病诊断和治疗，所开展的“不定点旋转手法（孙氏手法）治疗颈椎病”的方法，在国内中医学术界独树一帜，经多年临床应用表明，该疗法安全可靠，

中国知网 https://www.cnki.net

疗效明显，已被国家中医药管理局列为推广项目。

2 孙树椿教授学术源流

孙树椿教授为国家非物质文化遗产---清宫正骨派的代表人物，其学术源流要追溯到清朝初年的专为皇家治病的蒙古正骨大夫。道光初年正式成立上驷院绰班处，“绰班”一词是满词，译成汉语就是“正骨医生”，“绰班处”即“正骨处”。清《太医院志》记载：“旨以正骨科划归上驷院，蒙古医生长兼充。”目前，有文字记载的《绰班处花名册》记录的第一名绰班御医是清·咸丰九年七月二十三日被挑选入绰班处学习正骨的“正白旗满州如壁佐领下蓝翎侍卫杯塔布”。从这时起上驷院绰班处成为清廷大内唯一的骨科医疗机构，并进入全盛时期。

据张军教授等[2]考证，清宫正骨派祖师爷为德寿田。老先生为满族人，生卒年月不祥，道光中期受业于上驷院绰班处，为正五品衔御医。由于治疗骨折、关节脱臼、软组织损伤及其他慢性疾患有独到之处而名噪京城，人称“绰班德”。后技艺传至桂祝峰、怀塔布、崔海映等人，桂祝峰门下弟子有文佩亭，连坠等。孙树椿教授的恩师为刘寿山老先生，其即为文佩亭门下弟子。

3 清代上驷院绰班处宫廷正骨的起源和历史沿革

明末清初，战乱频频，在同明王朝作战中，满蒙八旗兵常发生坠扑跌折、关节脱臼及跌打损伤，在这种情况下，善于接骨按摩的骨伤科蒙古医生应运而生，并积累了宝贵的经验，据《清史稿》记载[3]：“善治伤，有中矢垂毙为拢镞，敷良药，寻伤愈。”（《清史稿》卷二百八十九）。又“天命中有患臂屈不能仲者，令先以热镬熏蒸然后斧椎其骨，操之有声即愈。”绰尔济是当时最著名的蒙古医生，他将其特效医术传授给广大的满蒙八旗士兵，培养了大批的满蒙八旗骨伤科医生，满语称之为“绰班”。

清代顺治初年设有御马监，顺治十八年改阿敦衙门，至“康熙十六年改为上驷院。雍正六年定卿为三品。”（《清朝文献通考》卷八十三职官七）。当时上驷院的主要任务是为清朝宫廷及骑兵驯养马匹，因满蒙八旗绰班医生主要随同骑兵一起调动，并为受伤的将士治伤，所以为数众多的领侍卫衔的蒙古绰班医生属上驷院管辖。据《清史稿》记载[4]：“上驷院兼管大臣，无员限。卿二人，正三品。其属……蒙古医生长三人，正六品。副蒙古医生长二人，八品。绰班长二人，初无品级。雍正元年定正七品……”，这段记述说明了从顺治年间到康

中国知网 https://www.cnki.net

熙、雍正年间上驷院内一直设有“绰班”御医职位。此时尚未形成正式的医疗机构，医学理论也未统一，手法亦未形成统一流派。

至乾隆年间，朝廷对医疗机构进行整顿，尤其对上驷院管辖内负责正骨按摩的蒙古绰班医生给予了高度重视，并对医生的选拔、教学、官职、责任方面进行了明确的规定，据《钦定大清会典事例》内务府官制卷一千一百七十一记载：“乾隆六年奏准（上驷院）额定阿敦侍卫二十一人”，“十一年奏准，于蒙古医内拔选医道优长，堪充教习者，授为蒙古医生头目二人。给予八品虚衔顶戴，令其教习蒙古医生。”当时朝廷的制度是在三旗的士卒中挑选懂得正骨技术者，每旗选十名，由上驷院管理，叫“蒙古医士”，晋升的最职称叫“蒙古医生长”。乾隆七年由吴谦、杨裕铎等人编辑的《医宗金鉴》终于刊行，《医宗金鉴·正骨心法要旨》则被上驷院绰班医生视为金科玉律，它所阐述的学术思想使得上驷院绰班医生在医学理论上得到统一，也标志着上驷院满蒙绰班医生“正骨心法学派”的诞生。

至嘉庆末年、道光初年，朝廷对太医院作出整顿，据清《太医院志》记载：“旨以正骨科划归上驷院，蒙古医生长兼充”，从这时起，上驷院绰班处正式成立，并成为清朝宫廷大内唯一的骨科医疗机构，开始进入全盛时期，学术思想和医疗技术日臻成熟，涌现出大批的满蒙汉优秀的骨科和按摩医生。

4 清代上驷院绰班处学术思想的历史发展过程

4.1 萌芽时期

这时期的代表人物是绰尔济，其学术思想主要体现了蒙古骨伤医生的接骨、外伤兼治的特点，医治方法辅以刀、锤、特效蒙医药物，依重秘方，手法、药物难分伯仲。

4.2 形成时期

《医宗金鉴·正骨心法要旨》的出现代表着上驷院绰班处学术思想的初步形成，它也成为了上驷院绰班处的手法宗旨。这时期的代表人物是伊桑阿，《清史稿》记载：“觉罗伊桑阿，乾隆中，以正骨起家，至巨富。其授徒法，削笔为数段，包以纸，摩裟之，使其节节皆结合，如未断者然。乃如法接骨，皆奏效。”由此可见，伊桑阿非常重视接骨的手法，对《医宗金鉴·正骨心法要旨》中正骨八法中“接”法的使用技巧颇为娴熟，他在承袭了绰尔济的学术思想后，更

中国知网 https://www.cnki.net

加注重手法的治伤作用，这也是受《医宗金鉴·正骨心法要旨》中“手法者，诚正骨之首务哉”观念的影响。

4.3 成熟前期

这时期的代表人物是德寿田（绰班德），他在功法、手法、器具、方药等方面的传授中，一方面宗《医宗金鉴·正骨心法要旨》为经典；又一方面更加注重摸法的传授。德氏要求上驷院绰班处的医生和学员们必须真正领悟到“摸”法中的奥妙，真正做到“则骨之截断、碎断、斜断，筋之弛、纵、卷、挛、翻、转、离、合，虽在肉里，以手扪之，自悉其情[5]。”可以看出德寿田的学术思想较绰尔济和伊桑阿更加注重手法的作用，强调“手巧”。上驷院绰班处学术思想在这一时期发展较快，进入了成熟前期。

4.4 成熟期

这时期的代表人物是夏锡五，夏氏认为要想做到“一旦临证，机触于外，巧生于内，手随心转，法从手出”，及“盖正骨者，须心明手巧，既知其病情，复善用夫手法，然后治自多效”，首先医生自己要做到“心明”，“无心则无法，心不明则法必乱”，即要“以心法统手法”，代表着上驷院绰班处学术思想成熟期的到来。夏氏对骨折的医治方法提出了正、整、接、实的治疗思想，对筋伤治法归纳为立、盘、旋、背、合、推、摇、摆、提等，充实和发展了《医宗金鉴·正骨心法要旨》中手法的内容。

4.5 成熟后期至今

这时期的代表人物是孙树椿教授，孙氏继承了师傅的学术思想、功法、手法真谛，潜心钻研，师古而不泥古，结合自己的临床经验，特别是对筋伤手法进行了系统的研究和整理，形成了具有孙氏特点的筋伤手法治疗体系，逐渐形成了“辨证论治、病证结合”、“气血辨证、以血为先”、“手法治疗、轻巧柔和”、“重视练功、自我康复”的学术思想，强调在治疗骨伤科疾病应在中医理论指导下辨证论治，在临床诊治时，既要辨病，又要辨证，只有病、证合参，才能选用适当方药，恰当的手法。经过大量的临床诊疗实践并结合中、西医理论，明确指出髓型颈椎病患者感觉以“疼、麻、凉”为其特点，属“痹证”者，运用舒筋活络、活血止痛、理筋整复的手法和药物等，使局部气血通畅，改变脊髓与致压物的解剖关系，松解局部粘连，减轻脊髓受到的压迫和刺激，完全可

中国知网 https://www.cnki.net

以通过非手术治疗达到目的；明确提出了”腰椎间盘突出”不等于“腰椎间盘突出症”；“骨质疏松”不等于“骨质疏松症”；　“骨质增生”不等于“骨性关节病”。这些观点的提出极大地减少了过度医疗的情况，既减轻了广大患者的痛苦，又减轻了国家的医疗支出，发挥了极大的经济效益和社会效益[6]。

孙树椿教授明确提出骨伤疾病无论伤筋还是伤骨，离经之血形成的“瘀”才是病机之初的根本，所以患者早期的表现主要以疼痛为主，痛有定处，痛处拒按，性质为针刺样、刀割拉样。日久瘀血影响气机条达，然后才出现气滞。因此，对骨伤科疾患辨证并不应是 “气滞血瘀”，而应该是“血瘀气滞”。

在手法运用上讲究轻柔和缓、外柔内刚，真正体现了“机触于外、巧生于内、手随心转、法从手出”的正骨手法要旨。使患者在没有痛苦感觉的情况下获得疾病的缓解或痊愈。轻：主要指动作要轻，使患者在心理上易于接受。巧：巧妙，一方面是指手法运用的技巧，另一方面是指用“巧劲”。柔：是手法用力要柔和，不能粗暴、生硬，强调刚中有柔，柔中有刚，刚柔相济。手法的力量要根据患者病情，并结合医生自身功力运用。对新伤用力要轻，动作要缓，而陈旧伤则可逐步加重用力。对于体质较弱、病情较重的患者治疗时要徐徐用力，以能耐受为限。对于身体强壮、病情较轻的患者，用力时使患者感到患处有沉重感或酸痛，但能忍受即可。和：就是心、手相合[6]。医者用手“体会”病患损伤的具体情况，取得对疾病的正确诊断是治疗的基 础，用“心”指导双手施术是治疗的目的。筋伤手法不是简单重复的机械运动，而是在“心”的指导下做的一种能量的输出。

参考文献

[1]高景华，张军. 孙树椿筋伤疾病诊治经验. 北京：中国中医药出版社，2014,1.
[2]张军 唐东昕 李俊海，等.孙氏筋伤手法脉系源流追溯考究.中国中医骨伤科杂志，2007，15（3）：56-57.
[3]赵尔巽撰.清史稿（卷五百二·列传二百八十九）[M].北京：中华书局，1977,3672---3678.
[4]赵尔巽撰.清史稿（卷一百十八·志九十三）[M].北京：中华书局，1977，169

中国知网 https://www.cnki.net

8-1899.

[5]吴谦等.医宗金鉴（第六册）.北京：人民卫生出版社，1957,278.

[6]孙树椿.骨伤名师二十三讲，北京：人民卫生出版社，2008：149-161.

中国知网 https://www.cnki.net

综述二 中医骨伤科对踝关节扭伤的认识与治疗

踝关节是人体重要的负重关节，损伤机会较多，各年龄组均可发生[1]。踝关节韧带为维持踝关节稳定的重要结构，踝关节扭伤往往会先伤及韧带。踝关节韧带损伤的发生率在全身关节韧带损伤中居第一位，约占80%以上[2]。

祖国医学认为无论急性踝关节扭伤还是慢性损伤在中医骨伤科辨证中都属"筋伤"的范畴。要了解踝关节扭伤必须先了解中医的"筋"与"骨"。现综述如下。

1 筋骨相关，筋束骨而利关节

在中医骨伤科"筋"的含义相当广，它概括了除内脏与骨以外的组织，相当于现代医学中的肌肉、肌腱、神经、血管、骨周围一切软组织的统称。早在《黄帝内经》里就有一些相关论述，认为骨性坚刚，是人体的支架，起支撑作用，而筋则是人体强健力量之源[3]。清代医家高士赚认为"大筋连于骨内，小筋络于骨外"，提出筋的作用是连接骨骼与关节，并支持肢体的运动[4]。

中医认为筋骨与五脏六腑都有联系，但关系最为密切的不过于肝肾。肝主筋，主藏血，一身之筋依赖肝血的滋养，而人体之活动，虽是筋之用，却关系到肝血的盛衰。只有肝血充盈，才能"淫气于筋"，使筋有所养，筋壮才能"束骨利机关"[5]。肾主骨，生髓。"肾藏精，精生髓，髓养骨"[6]。肾精的充盈与否能影响骨的生长发育、壮健与否和损伤的修复再生。另外，中医还认为脾主肌肉，为后天之本。人体肌肉的壮实与否，与脾胃的运化功能相关。脾气旺盛，气血充足，则肌膄壮实，反之则四肢无力消瘦或虚肥，甚或大肉尽脱。肝肾脾的亏虚可表现出衰老状态，表现为筋的运动不灵活，因此肝血不足，血不养筋，即可出现手足拘挛，肢体麻木，屈伸不利等症。骨是支持人体的支架，骨的坚硬依赖肾气的濡养和脾气荣华，肾精脾气不足，则骨髓空虚，肌萎无力，即可出现腿足痿软不能行动，腰瘦背痛活动不便等症。肝肾脾亏虚主要发生在久病之后或年老体弱的患者，临床上对肝肾亏虚的筋伤患者，要注意调养肝肾脾。

《灵枢·本脏》曰："经脉者，所以行气血而营阴阳，濡筋骨，利关节也"[7]。中医认为，经脉和利、气血通畅，则关节通利。如因外伤而致筋的损伤，则气血不循常道而溢于脉外，为肿为痛。如《圣济总录·伤折恶血不散》中云：

中国知网 https://www.cnki.net

“若因伤折，内动经络，血行之道不得宣通，瘀结不散，则为肿为痛” [8]。《灵枢·本脏》亦指出:“是故血和则经脉流行，营复阴阳，筋骨劲强，关节清利矣”。

踝关节扭伤主要是因不慎跌仆或外界暴力，以致筋肉损伤，出现局部疼痛、肿胀、皮下瘀血、活动不利等症状。如果损伤日久，气血运行不畅，气机阻滞不通，不通则痛，局部气机闭塞，可致瘀血，故病机为气滞血瘀。如清代医家沈金鳌在《杂病源流犀烛·卷三十》中云:“跌仆闪挫，卒然身受，由外及内，气血俱伤病也” [9]。中医治疗方面主要以活血化瘀止痛为治疗大法，并总结出动静结合、内外兼治等指导方针，形成了分期辨证用药、综合治疗的体系[10]。其中尤以外治法在治疗踝关节扭伤方面有独特疗效。

2 “筋出槽”、“骨错缝”与踝关节扭伤

中医骨伤科认为:筋槽是在肢体关节的关键部位的特有结构，起到保护、容纳、连结等作用;骨缝是骨与骨相接处的的一种特有结构，有时指的是关节，有时说的是骨间缝隙。唐代《仙授理伤续断秘方》有:“凡左右损处，只相度骨缝，仔细捻捺，忖度便见大概” [11]，提出“骨缝”概念。筋出槽是中医骨科的习惯用语，在各种文献中没有这一病名，但对筋损伤的病理改变文献中确有不少论述。《医宗金鉴·正骨心法要旨·手法释义》中说[12]:"筋之驰、纵、卷、挛、翻、转、离、合，"以及"筋歪"，"筋走"等等都属于"筋出槽"的范畴。

骨错缝是中医骨伤科疾病的传统病名，历代医家多有论述。《医宗金鉴·正骨心法要旨·手法释义》:“或因跌扑闪失，以致骨缝开错，气血郁滞，为肿为痛，宜用按摩法，按其经络，以通郁闭之气，摩其壅聚，以散瘀闭之肿，其患可愈” [12]。这里不但提出了骨缝开错这一名称，而且明确指出了骨错缝的原因和治疗方法。

中医骨伤科认为踝关节扭伤就属于“筋出槽”与“骨错缝”的范畴，骨错缝与筋出槽两者之间有密切的关连，有时筋出槽可单独出现，有时或合并骨错缝。踝关节扭伤后使踝关节周围的肌腱、韧带部分撕裂或断裂，脱离了正常的舒缩轨道，而导致筋伤出槽;筋出槽后不能维持原来正常的解剖位置，骨失去筋的牵拉维系处于非生理、不正常吻合状态，表现为骨错缝。相反，关节扭伤使小关节在外力的作用下出现了微细的错缝，关节周围的关节囊、韧带等软组织，也可相应的发生改变，如关节囊的破裂，韧带、筋膜的撕裂等。

中国知网 https://www.cnki.net

总之，在人体的踝关节骨和筋是相辅相成的一个整体，有时骨错缝与筋出槽是同一损伤的两部分或者是不同阶段，骨错缝必然导致踝关节周围的筋出槽，而筋出槽的发生也可以引起踝关节部位的骨错缝。在治疗时要二者兼顾，在纠正了骨错缝之后筋则可自然恢复正常的位置。同样，出槽的筋恢复了其正常舒缩轨道也可以使错缝的骨复位，从而使疼痛等症状自然会马上消失。

3 中医手法在治疗踝关节扭伤中的应用

运用中医手法治疗踝关节扭伤是临床重要的手段之一，临床常用的手法按摩乃取其“理筋整复，活血化瘀”的原则来治疗踝关节扭伤后导致的韧带损伤。对于陈旧性踝关节扭伤大多中医骨伤科医师主张可以进行手法治疗，但对于急性踝关节扭伤是否能够采用手法治疗存在争议。潘志雄等[13]以手法治疗急性踝关节韧带损伤 486 例，治愈 467 例，占 96.1%，好转 19 例，占 3.9%，无效 0 例。认为此方法治疗该病简单易行，操作方便，疗效可靠。许多医家同意此观点[14]。但是赵勇等[15]认为踝关节损伤 24 小时内禁止按摩，否则会加重损伤，主张用冰敷或外敷中药消肿。刘艳等[16]亦持类似的观点，并认为不仅早期不宜手法治疗，就连早期外敷活血性药物也不主张应用，以免促进局部出血，使肿胀加重。但主张扭挫伤 24 小时后，可以内服与外敷活血性药物会起到良好的治疗效果。

孙树椿教授通过自己多年的临床经验得出无论踝关节急性损伤还是慢性损伤均可进行手法治疗，关键是什么时期采取何种手法，急性期不宜应用按揉等手法治疗踝关节局部，但可以采取摇、拔、戳等运动关节类手法[17]。吕燃等[18]应用孙树椿教授的手法，对 32 例急性踝关节扭伤患者进行治疗，治疗前后均对患者的疼痛进行评分，采用自身前后对照的方法，得出治疗后的疼痛分值明显低于治疗前，并认为孙氏手法对急性踝关节扭伤患者的疼痛症状有明显改善作用。笔者也同样运用该类手法治疗急性踝关节扭伤疗效与上基本一致。孙宝玲等 [19]采用推、摩、滚、揉足三里、解溪、阿是穴等穴位，配合活动踝关节治疗急性踝关节扭伤 73 例，疗效显著。李金宝等[20]以挤压类手法治疗该病 30 例，治愈 20 例，显效 6 例，有效 2 例，无效 2 例。林应强等[21]应用挤压手法加“8”字绷带外固定踝关节于中立位治疗急性踝关节扭伤 60 例，临床也收到满意疗效。可见，治疗急性踝关节扭伤关键不是在于能否采用手法治疗，而是采用何种手法。手法的治疗也和疾病的分期息息相关。对于陈旧性踝关节扭伤大多数医家

中国知网 https://www.cnki.net

都主张可以采用手法治疗，而手法又各有特色。但通过手法复位错缝的关节，理顺局部筋络，恢复原有正常解剖关系的治疗原则是一致的。

4 中医综合疗法在治疗踝关节扭伤中的应用

在临床中医外治法治疗踝关节扭伤种类繁多，各具优点，有单纯采用某一种方法治疗而取得疗效者，亦有综合两种甚至多种方法而治疗取得满意疗效者。中医综合疗法就是在中医药理论的指导下，根据临床需要，将中药外敷、熏洗、针灸以及手法等结合应用治疗踝关节扭伤的疗法，目前已越来越广泛地应用于临床。当前文献报道较多的是手法配合外敷药物治疗踝关节扭伤具有治疗方法简便，疗效确切，疗程较短，治愈率高等优点[22、23、24]。陈泽林等[25]报道采用手法配合外敷中药治疗急性踝关节扭伤患者并与单纯采用中药外敷作为对照，治疗共 70 例患者，治疗组有效率为 88.58%，对照组为 71.43%，有显著性临床意义。谢凯[26]报道采用针刺后用中药外敷治疗踝关节扭伤患者 80 例，取穴以局部为主，如取丘墟、解溪、悬钟、昆仑、太溪等，出针后以中药“四黄散”(生大黄、生黄栀、黄柏、生黄芩各等份，研碎组成)适量水调，外敷于患处，并给以绷带外固定，结果显示针药组治愈率明显高于单纯针灸或单纯外敷药物组（$P<0.01$）。

根据文献数据分析显示大部分的文献报道中医综合疗法主要集中于手法与外用药物配合或针灸与外用药物配合方面[27]，当然也有少部分学者主张三种以上的综合疗法治疗踝关节扭伤[28]。但是孙树椿教授主张如果能用一种方法取得比较满意的疗效，不建议运用多种方法叠加，这样不仅减少了患者负担，也降低了患者的痛苦[29]。笔者更同意此观点，临床医生由于害怕一种方法不能取得满意疗效，而选择两种甚至两种以上的治疗方法叠加，至于何种方法起到了真正疗效不做进一步探究。

总而言之，中医外治方法治疗踝关节扭伤方法众多，并有使用简便，疗效确切，毒副作用低等优点，值得临床应用推广，但是尚还存在许多的问题和不足。如相当一部分研究来自临床观察或个人的经验总结，样本量较少，缺乏科学严谨的对照，观察指标也比较主观，评估也缺乏统一的标准，因而疗效难以被认可。而且相关的实验研究相对较少，客观指标不够，因而缺少具有可比性的数据，从而使其可信度降低。另外，大部分外用药存在配置较为繁琐、应用

中国知网 https://www.cnki.net

复杂、污染衣物、不便于携带等缺点。因此，临床更应大力推广行之有效的手法治疗该病。

参考文献

[1] 孙树椿. 清宫正骨手法图谱. 北京：中国中医药出版社，2012,209-217.

[2]王正义.足踝外科学[M].北京：人民卫生出版社，2006，205-210.

[3]程士德.黄帝内经.北京：人民卫生出版社，2006,207-208.

[4]姜鹏飞，王培民.急性软组织损伤中医外治的研究现状[J].中国医药导报，2009,6（1）：86-87.

[5]孙树椿、孙之镐. 临床骨科学（第二版）. 北京：人民卫生出版社，2014，29-40.

[6]程士德.黄帝内经.北京：人民卫生出版社，2006,100-102.

[7]王庆其.内经选读.北京.中国中医药出版社，2007,76-77.

[8]赵佶.（郑金生整理）. 圣济总录. 北京.人民卫生出版社,，2013，1627-1628.

[9]沈金鳌.杂病源流犀烛.北京：人民卫生出版社,2006,1026-1071.

[10]黄桂成、王庆甫. 中医正骨学. 北京：人民卫生出版社，2012,1-3.

[11]蔺道人.（胡晓峰整理）.仙授理伤续断秘方.北京：人民卫生出版社，2006,18.

[12]吴谦等.医宗金鉴（第六册）.北京：人民卫生出版社，1957,278.

[13]潘志雄,何家雄,徐志强等.手法治疗急性踝关节韧带损伤 486 例疗效分析[J].甘肃中医, 2006,19(1):24-25.

[14]陈兆军、唐凡启、林顺福，等.踝关节韧带损伤的早期诊治[J].中国骨伤，2007,20（5）：330-331.

[15]赵勇.手法配合外敷止痛消炎软膏治疗踝关节扭伤 50 例[J].中医外治杂志,2006，15(4):46.

[16]刘艳,马珍珍.足踝关节扭挫伤的处理与预防[J].河南外科学杂志,2010,16(5)：87-88.

[17] 高景华、高春雨、孙树椿，等.摇拔戳手法治疗陈旧性踝关节扭伤 34 例.世界中医药，2011,6（3）：214-215.

[18]吕燃.孙氏手法对缓解急性踝关节扭伤疼痛症状的疗效观察[J].按摩与导引,20

中国知网 https://www.cnki.net

08，24(6):37-38.

[19]孙宝玲.按摩治疗急性踝关节扭伤 73 例[J].按摩与导引,2001,17(6):49.

[20]李金宝.挤压手法治疗急性踝关节扭伤30例疗效观察[J].中华实用中西医杂志，2003, 3(06):851-852.

[21]林应强，吴山，马友盟.挤压手法治疗急性踝关节扭伤 60 例疗效观察[J]. 中医正骨，2000,12(07):21.

[22]孙琦、王丹、刘海兵，等. 复合手法结合中药熏洗治疗陈旧性踝关节扭伤疗效观察. 浙江中医杂志，2015,50（4）：281.

[23]李俊海、王庆甫、黄沪. 正骨手法与中药熏洗治疗陈旧性踝关节扭伤的病例对照研究. 中国骨伤，2012,25（2）：113-115.

[24]张继明.手法配合中药外敷综合治疗踝关节扭伤 63 例[J].中国初级卫生保健，2008，2(2):88-89.

[25]陈泽林,洪文.手法配合中药外敷治疗急性踝关节扭伤临床研究[J]. 中医学报，2011, 26(2):245-246.

[26]谢凯.针药结合治疗急性踝关节扭伤 80 例[J].上海针灸.2002,21(3):32.

[27]黄向群.针药并用治疗陈旧性踝关节扭伤 23 例临床观察.四川中医，2010，28（8）： 122-123.

[28]李景元.手法结合针刺及中药外用治疗陈旧性踝关节扭伤 164 例临床观察.中国中医基础医学杂志，2013,19（4）：480-481.

[29]吴山，马友盟，林应强.挤压法治疗陈旧性踝关节扭伤 47 例. 新中医，2000, 32（7）：31.

中国知网 https://www.cnki.net

综述三　现代医学对踝关节扭伤的诊断和治疗进展

踝关节扭伤临床多见可发生于任何年龄，以青少年多见，可分为急性和陈旧性损伤。尽管该病在临床有较高的发病率，但是无论医生还是患者对此损伤的重视仍然不够[1]。该病若早期处理不当，常遗留疼痛，严重者可影响踝关节功能，甚至后期发生踝关节不稳。若反复踝关节扭伤，并可继发踝关节骨性关节炎等，形成不可逆的病理变化。目前学术界对于踝关节扭伤特别是陈旧性踝关节扭伤的诊断和治疗尚无统一的规范和标准，还存在较大的差异及一些争论。

1 踝关节的解剖特点

人体的踝关节是屈戌关节，周围有韧带包绕，是踝关节的稳定机制。踝关节周围韧带主要分为内、外侧副韧带和前后侧韧带，前后侧韧带分别位于踝部关节囊的前、后部，较为薄弱，这样有利于踝关节的屈伸运动。人体踝关节主要功能就是背伸和跖屈，只允许少量的内外翻运动，因此临床上的踝关节扭伤主要发生于内外翻活动时，又由于外踝比内踝低，踝关节扭伤主要是内翻所引起。因此踝关节扭伤主要损伤的是内外侧韧带，其中又以外侧韧带损伤更多见。

1.1 内侧副韧带

踝关节内侧副韧带又名三角韧带，分为深、浅两层。该韧带由胫距前韧带、胫舟韧带、胫跟韧带及胫距后韧带组成。三角韧带较踝关节的外侧韧带坚强，其主要生理作用为从内侧加强踝关节。当踝关节受到由内向外的暴力时，其前部内侧附着点处可发生撕裂。三角韧带的胫距后韧带与外侧的距腓后韧带相对应，起于内踝后缘，止于距骨后部，靠近踝关节的运动轴，正常运动时维持紧张状态[2]。

1.2 外侧副韧带

外侧副韧带不如三角韧带坚强，外踝也比内踝较长。外侧副韧带由前向后依次为距腓前韧带、跟腓韧带、距腓后韧带。在踝关节外侧副韧带中距腓前韧带薄弱，走向几乎成水平方向。在踝关节跖屈位时，此韧带的主要作用就是限制足内翻活动，而在踝关节中立位时，距腓前韧带还有对抗距骨向前移位的作用。跟腓韧带在踝关节外侧的三个韧带中属于中等坚强，并有腓骨长、短肌越过。由于踝关节运动轴线位于此韧带前方，所以跟腓韧带在踝部背伸时紧张，在跖屈时则松弛。在踝关节中立位时跟腓韧带有限制足内翻的作用。距腓后韧

中国知网　https://www.cnki.net

带为踝关节外侧副韧带三束韧带中最坚强者，其有部分纤维与拇长屈肌腱想融合。距腓后韧带主要功能就是限制踝关节的过度背伸，临床很少发生损伤。

在临床上，在踝关节各组韧带中，以外侧副韧带损伤最为常见，约占急诊运动损伤的16%[3]。主要原因是踝关节内踝较外踝位置高，外侧三束副韧带较内侧的三角韧带薄弱，并且引起足内翻肌群的肌力较外翻肌群强大。当人体快速行走或参加各种体育运动时，若足来不及协调好位置，容易造成内翻跖屈位着地。当踝关节跖屈位受到内翻应力时，距腓前韧带最为紧张，故首先发生距腓前韧带损伤[4]。Ozeki 等[5]通过研究发现当踝关节跖屈 16.4° 时，距腓前韧带达到最长、张力最大，当足部内翻、跖屈位着地时，距腓前韧带最易发生断裂。这从形态学上解释了距腓前韧带容易发生损伤的原因。另外，在踝关节外侧副韧带的急性损伤的患者中 10%～30%会发展成慢性踝关节不稳定[6]，严重者还可合并外踝尖部骨质撕脱。

2 急性踝关节扭伤的认识与诊断

急性踝关节扭伤是指踝关节内翻或外翻损伤后不伴有骨折、脱位的单纯踝关节韧带损伤的总称。临床如何明确诊断该病至关重要。临床上一般认为诊断该病必须综合考虑损伤病史、临床表现，体格检查、以及 X 线片、CT 或 MRI 辅助检查等多因素综合判断才能明确诊断急性踝关节扭伤[7]。

2.1 病史及临床表现

要重视病史的采集，有助于对踝关节周围韧带及损伤机制的判断 [8]，临床上，急性踝关节扭伤多存在踝关节内外翻扭伤史，局部的疼痛、肿胀、青紫瘀斑、活动时疼痛加剧等，其中局部肿痛为主要临床表现[9]。

2.2 体格检查

检查时足部，踝部和小腿应特别注意让患者充分放松，另外仔细触诊踝关节周围各韧带起点和终点的位置也同等重要，还应同时评估踝关节主动活动范围、感觉神经是否正常、肌肉肌力等，如腓骨肌无力常提示可能存在踝关节慢性不稳等[10]。

在临床上踝关节前抽屉试验是应用最多的检查方法。其最早由 Dehne 在 1934 年所描述，主要是用于判断是否存在距腓前韧带的损伤[10,11]。目前临床上常用踝内翻与外翻应力试验、前后抽屉试验等来综合判断踝关节侧副韧带损伤

中国知网 https://www.cnki.net

及踝关节不稳定的程度[4]。

2.3 X 线检查

X 线虽然是临床分辨有无骨折的有效辅助检查方法，但是对于踝关节有无韧带损伤同样有临床意义。唐三元[12]认为所有的踝关节扭伤有疑问的情况下，前抽屉试验，距骨倾斜试验以及拍摄应力位 X 射线等即可确诊。陈兆军等[1]建议双侧踝关节 X 线片进行对比，以便作出及时的诊断。Black[13]认为在应力位 X 片上当距骨倾斜大于等于 10° 时可判定距腓前韧带存在损伤。如果倾斜角度>20° ，则提示踝关节外侧韧带全部撕裂。杨志[14]亦提出摄片应力位踝关节 X 线片对判断踝周韧带的损伤程度有重要价值，应以健侧作为比较，在内翻应力位时：距骨倾斜若大于 10° 则提示踝外侧韧带有损伤；若大于 15° 表明存在距腓前韧带的不同损伤；而 15～30° 提示距腓前韧带、跟腓韧带同时有损伤；若超过 30° ，则表示踝关节外侧韧带遭受结构损伤。

2.4 CT 及 MRI 检查

CT 及 MRI 检查对踝关节副韧带损伤有重要的参考价值，陈兆军等[1]认为如果患者经济条件允许双侧踝关节对比 CT 或 MRI 检查对诊断踝关节韧带损伤很有必要。但是大多数学者更推崇将 MRI 应用于踝关节韧带损伤程度的诊断中[15]。周捷等认为[7]MRI 是检查外侧副韧带有无断裂的好方法，并且对于职业运动员伴有内踝压疼的病例，应使用 MRI 除外关节软骨是否有损伤。Verhaven 等[16]观察发现，三维快速成像 MRI 检查对发生两条韧带撕裂的运动损伤有很大的临床意义。许多学者[17][18]研究发现高分辨率的 MRI 可以很清楚的显示跟腓韧带和距腓前韧带。白万山等［19］认为 MRI 检查韧带损伤和定性损伤方面，明显优于其他检查手段。

2.5 关节造影

Brostrom1965 年将造影用于诊断脚踝韧带损伤[20]，他将造影剂注射关节后拍摄 X 线，观察是否造影剂从内部或外部溢流，可以帮助诊断韧带有无损伤。Miyagi 等[21] 认为造影术完全可以作为诊断急性踝关节韧带损伤的有效辅助检查。然而持反对观点的学者认为腓骨肌腱鞘和潜在滑液囊可以相通，假阳性结果很高。周捷等[7]认为踝关节造影是一种有创操作，没有必要用关节造影来证实外踝韧带断裂，关节造影剂还有可能再次刺激踝周的滑膜组织，造成进一步损

中国知网 https://www.cnki.net

伤。

2.6 超声检查

林发俭等[22]认为超声是一种诊断踝韧带的可靠方法，特别是对完全撕裂的韧带损伤的诊断，具有很高的准确性。也有人认为单纯的韧带挫伤，在超声中很难诊断清楚，急性韧带损伤时，多为血肿回声，对声音信号有干扰 [22]。有人认为踝关节损伤在超声检查中可以通过分度来了解损伤的程度[23]。周杰等[8]在临床实践中结合高频超声检查，将踝关节外侧韧带损伤程度分为Ⅰ度：外侧韧带拉伤；Ⅱ度：外侧韧带部分断裂；Ⅲ度：两个或两个以上踝关节外侧韧带断裂。贺小兵等[24]采用超声下 3 度划分法描述踝关节侧副韧带损伤：Ⅰ度，轻微韧带损伤；Ⅱ度，韧带不完全损伤；Ⅲ度，韧带完全撕裂或断裂。Ⅲ度损伤时通常会提示日后可能导致踝关节不稳。而唐农轩[25]将韧带损伤分为四度，即Ⅰ度：距腓前韧带部分撕裂；Ⅱ度：更多的纤维断裂，但距腓前韧带尚连续；Ⅲ度：距腓前韧带完全断裂；Ⅳ度：距腓前韧带及跟腓韧带完全断裂。但是许多临床医生对于踝关节韧带损伤的分度，大多还是采用三度划分的方法。

3 陈旧性踝关节扭伤的认识与诊断

陈旧性踝关节扭伤多由急性踝关节扭伤疾病失治、误治或积劳成疾所致[26]。其最主要的危害就是后期造成的踝关节不稳，相关文献显示高达 20%~40%的踝关节扭伤将演变成慢性踝关节不稳定[27, 28]。大多数学者认为陈旧性踝关节扭伤的伤害是踝关节内外侧韧带在松弛位愈合后造成的踝关节不稳，其中尤以踝关节外侧不稳为最多见[29, 30]。患者多表现为行走不平地面时的恐惧感或不稳定感，长时间行走时关节的酸痛及酸胀感，以及踝关节活动受限[31]。

踝关节不稳可以分为机械性不稳定和功能性不稳定，而机械性不稳定又可以分为骨性不稳定和软组织性不稳定。机械性不稳定是指由于各种原因造成的关节活动范围超出了正常生理极限的疾患。由于该病多伴有关节或骨骼的发育异常问题，在此不做特殊讨论。功能性不稳定是指由于各种原因导致关节活动不一定超出正常生理极限，但却完全或不完全失去自主控制的疾患[32]。有学者研究表明[33]，在解剖学和病理学方面看，功能性不稳定的踝关节和正常关节没有区别。有的学者认为踝关节囊或者韧带上存在机械感受器，该病就是机械感受器缺乏或损伤所致[34]。在人体膝关节交叉韧带上存在机械感受器已经被证实。Takebayashi 等[35]在猫的踝关节韧带上证明有电生理反应，发现绝大多数（93%）

中国知网 https://www.cnki.net

的机械感受器单位是处于韧带的远、近两端，毗邻于骨附着处。但在人体踝关节韧带上是否存在尚不能肯定。

外踝关节的被动稳定主要靠关节面及韧带的完整，而动态稳定则有赖于重力作用、肌肉活动及足与地面相互作用等的综合因素[36]。为了了解韧带的功能和在踝关节不稳中的作用，Bahr 等[37]设计了一种小型传感器安装在韧带上，并安装同时具有三维活动的角度测量器，测量踝关节活动度，通过分别切断不同的外侧副韧带来了解那根韧带在踝关节活动中的作用最大。结果显示：距腓前韧带在跖屈下所受应力最大，跟腓韧带则在背伸下应力最大，并发现单独的距腓韧带损伤仅引起较轻的韧带松弛，而在距腓前韧带和跟腓韧带同时损伤下韧带松弛显著增加。在踝关节的韧带损伤中非负重下相比负重状态更容易引起韧带损伤，关节的完全接触提供了 100%的内翻稳定性和 30%的旋转稳定性。但在实际的临床上踝关节的损伤既可以发生于非负重位也可以发生于负重位[38]。

对于陈旧性踝关节扭伤以及是否导致踝关节是否稳定的诊断，同样需要从病史、临床表现、体格检查、辅助检查几个方面综合考虑。在临床上踝关节局部肿痛以及反复发生的踝关节扭伤是陈旧性踝关节扭伤的主要表现。查体距骨相对胫骨前后方向的移动被用作判断距腓前韧带有无损伤的重要手段[39]。而距骨倾斜实验则用于判断距腓前韧带有无合并跟腓韧带损伤。诊断陈旧性踝关节扭伤的辅助检查包括应力位 X 片、关节造影、MRI、关节镜检查等。在这些检查中应力位 X 片最低廉临床也最实用，在正位 X 线上测量胫骨下关节面与距骨上关节面的成角、一般认为>10° 即有临床意义。成角越大、韧带损伤越重[40]。

4 急性踝关节扭伤及陈旧性踝关节扭伤的治疗

4.1 急性踝关节扭伤的治疗

对于急性踝关节扭伤早期是否需要治疗，当前临床还存在着较大的争议[1]。就治疗方法而言，主要分为非手术与手术方法两种。

4.1.1 非手术治疗

在非手术方法治疗中，多数学者强调早期诊断韧带有无损伤以及及时早期处理的重要性。并且主要以早期固定受损的韧带为主。唐三元认为[12]多数单纯急性踝关节韧带损伤的踝关节扭伤均可采用非手术方法治疗，其方式包括固定、功能治疗(指采用弹性绷带外固定、控制下主动及被动活动或行走)以及辅以药物

中国知网 https://www.cnki.net

治疗。陈兆军等[1]认为急性踝关节扭伤无论韧带损伤的程度严重与否，早期固定对恢复踝关节功能均有好处。固定可以为韧带修复创造有利的条件，不仅能够稳定踝关节，还可以最大限度的避免出现踝关节不稳。王三好[41]认为踝关节韧带急性损伤只有及时的早期处理，合理的固定和有效的康复结合起来，才能达到预期的治疗效果。并且认为无论弹性绷带外固定，还是运用护踝、胶布固定等来治疗韧带损伤，应该都属于固定的范畴。并且认为弹力绷带外固定不仅能起到固定踝关节的作用，而且在绷带的缠绕过程中，从外踝斜向前或向后的两组绷带的走向就类似于距腓前、后韧带的走行方向，而两者的合力方向大致为跟腓韧带的走行方向一致。通过这种固定方式，可保证踝关节外侧韧带中的任意一束韧带在修复过程中不再受到损伤。Karlsson 等[42]认为，踝关节用绷带外固定支持后，可加快本体感受信号传入，这样可以使腓骨肌的反应时间缩短，而后者正是维持踝关节稳定的重要结构。Feuerbach 等[43]研究发现，受到损伤的踝关节在用绷带外固定后，并不是真正固定了踝关节而是主要使皮肤感受器的传入反馈得到了改善，进而改善了踝关节的位置觉，使踝关节得到了保护。但也有学者持相反的观点。Freeman[44]提出对于急性踝关节的损伤，早期活动关节比制动固定关节更为重要，并主张早期踝关节功能活动的重要性。Pijnenburg 等[45]研究结果显示：踝关节功能治疗无论在恢复工作、费用、优良率上均优于石膏固定组。因此也有学者采取了折中的治疗方法。黄涛等[46]的研究认为对踝关节进行有限固定比行石膏托牢固外固定踝关节功能恢复更优良。韩军良等[47]采用胶布外固定治疗军训所致的踝关节外侧副韧带损伤 78 例，也同样取得了满意疗效。一般认为韧带的修复通常是通过瘢痕愈合的方式进行，韧带在无张力的情况下修复可使形成的瘢痕少，韧带愈合后不松弛，从而起到稳定踝关节的作用。唐剑邦等[48]认为在急性踝关节韧带损伤的治疗过程中，护踝的作用也非常重要。佩戴质量优良的护踝可一定程度上限制患侧踝关节的活动，拥有一定的制动作用；同时又可使对抗内翻的力量增强，避免了在恢复期间的再次损伤，起到相应的保护作用。护踝可以起到类似于踝周韧带的伸缩特性，使踝关节固定而不“僵”、动中有制，动静结合，允许踝关节能在的适当范围内具有一定的活动度，有助于创伤的修复。

4.1.2 手术治疗

对于是否需要早起手术治疗急性踝关节韧带损伤，需要根据韧带损伤程度

中国知网 https://www.cnki.net

来综合考虑。王正义等[2]认为对于比较严重的韧带损伤或开放性损伤，必须手术修补韧带。也有学者[6]主张如果仅仅单纯距腓前韧带损伤，可通过足外翻位、踝背屈位 8 字绷带加压固定即可；若为韧带部分断裂早期可采用石膏固定；而韧带完全断裂则建议手术修复。有学者认为只有在距腓前韧带及跟腓韧带两束韧带完全破裂时才选择手术修复。也有人认为外侧副韧带断裂，早期行手术修复可使韧带获得良好愈合，更有利于恢复韧带功能。王敏等[49]提出对于踝关节外侧副韧带III度损伤的患者，单纯通过外固定治疗易产生踝关节外侧不稳。其对 11 例踝关节外侧副韧带III度损伤患者进行了早期手术修复，并根据 AOFAS（美国足与踝关节协会）踝与后足的功能评分进行疗效评定，总分 85～96 分，平均 93.6 分。并且认为III度以上损伤的患者距腓前韧带、跟腓韧带一般均已断裂，对较年轻的患者应积极手术治疗并进行解剖性修复。郑金荣[50]等通过观察不同治疗方法后期出现踝关节不稳、疼痛等后遗证，得出III度损伤患者手术治疗组治愈率明显高于单纯石膏固定组。有学者主张不用考虑踝关节外侧副韧带损伤程度全部选择手术治疗。Pijnenburg 等[51]将踝关节外侧韧带损伤的患者分为两组，一组用标准的外科修补，另一组做功能锻炼治疗，随访 7.7 年，结果发现外科修补组患者踝关节稳定性较功能治疗组好，踝关节抽屉试验阳性率较功能治疗组低。Lacko 等[52]的研究也支持这样的观点。然而，也有人的研究结果与上述学者相反。Hazanas 等[53]的研究结果显示：采取功能治疗与手术修复治疗之间的踝关节功能恢复优良率无明显差异。Kaikkonen 等[54]回顾性随访了 100 例应用一期外科治疗的外侧踝关节损伤病例的结果，随访时间 6～10 年，并与采用功能治疗的病例比较，发现手术组优良率仅为 65%，其认为对急性外侧踝关节韧带损伤早期当首选功能治疗。

由此可见，对于急性踝关节侧副韧带损伤，是否采取手术或非手术治疗，目前争议仍较大。洪文跃[55]提出早期的踝关节韧带损伤国内多采用保守的非手术治疗，而手术治疗可获得良好的稳定，但恢复期较长。陈兆军等[1]认为临床应根据患者的具体病情、相关职业、功能需求及经济条件等来综合考虑，决定是否采取手术治疗；对于严重的踝关节扭伤，如果是年轻的患者或者从事某些特殊职业者，如舞蹈员、运动员等要求较高，需要患侧踝关节今后比较耐用、功能较为良好，则即可选用手术方法治疗；而对于一般患者大都可以选择性应用相关保守治疗手段，因为这些治疗方法具有治疗简便、患者受痛苦小、费用低

中国知网 https://www.cnki.net

廉等优点。

总的来说，对于急性踝关节扭伤越来越多的学者逐步认识到早期固定制动对于踝关节功能恢复的重要性。至于是否选择手术治疗该病，当前临床尚存有争论，但本人认为应该根据患者年龄、职业、损伤程度等实际情况，恰当选择不同的治疗方法，以期使损伤能在早期得到妥当地处理，尽量减少其发展为慢性踝关节不稳定的可能。

4.2 陈旧性踝关节扭伤及踝关节不稳的治疗

对于陈旧性踝关节扭伤及踝关节不稳的治疗也存在着非手术和手术治疗之分。

4.2.1 非手术治疗

陈旧性踝关节扭伤及功能性踝关节不稳患者若症状轻微对生活或工作影响不大者可采用加强外翻肌肌力锻炼、支具、矫形鞋等保守治疗。根据研究发现踝关节功能性不稳与平衡控制技能有重要关系[56]。此类疾病的非手术治疗主要针对踝关节的本体觉及平衡、肌力训练方面[57]。何伟华等[58]采用肌肉功能锻炼的方法对慢性外踝关节不稳的40例患者，随机分为3组，A组进行踝背屈肌功能锻炼、B组进行患踝外翻肌肌力锻炼、C组同时进行背屈、外翻肌肌力锻炼，并都在锻炼的同时以医用橡皮驱血带作为训练器材进行包括肌力、肌耐力、肌爆发力等训练，结果显示C组患者的AOFAS评分明显高于前两组，通过12周的观察发现背屈、外翻肌力功能锻炼均能有效改善慢性踝关节不稳患者症状，但以两者组合锻炼者为最优。Clark等[59]对19例踝关节功能性不稳定的患者进行了数周的平衡板摇摆训练，每周3次，分别训练2周和4周时测定胫前肌、腓骨长肌表面肌电反应，结果显示训练2周后胫前肌、腓骨长肌反应潜伏期明显缩短，训练4周后踝关节功能评分显著改善。Eils等[60]对20例功能性踝关节不稳定患者进行每周1次的康复训练，包括热身、在移动平台上单腿站立、利用弹力带进行外展练习，并在气垫、倾斜板、水平或垂直移动平台、Biodex稳定系统等装置或仪器上进行单腿或双腿平衡训练，每个项目20分钟，6周后发现受试者晃动明显改善，并且腓骨肌反应时间显著缩短。当前多数学者认为平衡、本体感觉以及肌力等方面的训练对功能性踝关节不稳定患者恢复稳定和功能恢复有积极作用[61]。在中国大多数学者主张将中医的理筋手法加入踝关节韧带损伤或踝关节功能性不稳定的治疗中，并且发现手法加平衡、本体感觉以及

中国知网 https://www.cnki.net

肌力锻炼可以明显提高临床疗效[62]。有人认为通过特定手法推拿可以提高下丘脑内啡呔的含量，降低缓激肽、5-羟色胺等炎性介质的含量，促使内外神经根内外水肿吸收，发挥消炎镇痛的作用[63]。吴贵根等[64]认为推拿对肿胀的组织具有良好的活血化瘀作用，加快静脉和淋巴液向心回流，有利于水肿吸收。再加上专业的康复训练可加强踝关节磨合，增加踝关节周围韧带的柔韧性和协调性，从而恢复踝关节的稳定[65]。

4.2.2 手术治疗

踝关节机械性不稳定往往由于踝关节结构异常所致，大多都需要手术重建，不在此讨论的范围内，而对于各种踝关节陈旧性韧带损伤或踝关节功能性不稳定，应以非手术治疗为主，效果不良者可以我考虑进行手术治疗。手术解剖的复位和重建是非常重要的，但是康复的标准需要兼顾运动功能恢复和神经肌肉系统功能恢复[66]。

该类手术术式繁多，大约有 50 余种手术可供选择[67、68]，但大体可以归纳为以下三类：①对韧带起止点的原位解剖缝合重建手术。②使用部分或全部腓骨肌肌腱重建外踝韧带的手术(非解剖重建)。③使用自体、异体或人工肌腱非原位固定重建外踝韧带的手术（解剖重建）。

4.2.2.1 对韧带起止点的原位解剖缝合重建手术

对韧带起止点的原位解剖缝合重建手术又分为韧带皱缩手术和韧带紧缩缝合手术。前者多用于踝关节外侧陈旧性韧带损伤的治疗，适合于外踝副韧带在松弛的位置上愈合，但韧带序列尚完整者。具体方法多采用关节镜下操作选择低温等离子射频技术皱缩踝关节外侧副韧带，主要是距腓前韧带应用较多。目的是恢复韧带张力，改善踝关节稳定性[69]。该方法的理论依据是希望通过射频冷凝技术是韧带组织在紧缩的同时仍能保持组织的活性。但是该手术方法存在疗效不确定、组织破坏吸收、韧带紧缩过度或不足等缺点，临床已较少采用。对于外侧副韧带残端较完整的患者，可以采用韧带紧缩缝合术来改善踝关节的稳定性。该手术一般都在开放下进行，但近些年有越来越多的学者主张在关节镜下进行创伤更小、恢复更快，还会获得开放手术类似的效果[70、71]。开放术式以 Brostrom 术式[72]为代表，主要是将陈旧性撕裂的外侧副韧带在体部切断，将断端重叠缝合，达到紧缩韧带的目的。近些年随着铆钉技术的发展，该手术方式又有了许多改良[73、74]。该类术式操作简单、创伤小、疗效可靠，本人运用该

中国知网 https://www.cnki.net

技术治疗陈旧性踝关节扭伤后踝关节功能性不稳定患者多例，均收到满意疗效。

4.2.2.2 非解剖重建手术

对于损伤时间长，损伤较重的患者可能出现韧带残端吸收，或韧带松弛、肥胖等特殊患者则需要进行肌腱重建外踝韧带的手术[75]。目前临床重建手术应用最多的就是非解剖重建手术。在该类手术中最有代表的手术是 Evans、Watson-Jones、Chrisman-Snook、术式及其改良手术[76]。Evans 手术方法为切取腓骨短肌腱通过外踝骨隧道环绕最后与骨膜缝合，以起到控制踝关节内翻和距骨前移的作用。该手术操作简单，对术者要求相对较低，短期疗效尚可，长期疗效欠佳[77]。Watson-Jones 手术是对 Evans 手术的改良和补充，具体做法是切去更长的腓骨短肌腱固定于腓骨后继续向前最后固定于距骨颈上重建距腓前韧带的方法。Chrisman-Snook 手术是通过多个骨隧道切取较长的腓骨短肌腱的一部分即重建距腓前韧带又重建跟腓韧带的手术方法，据报道该方法疗效可靠[78]。改良手术中典型的是 BrostrÖm-Gould 手术，即利用伸肌支持带修复距腓前韧带或跟腓韧带的方法。在这些手术中应用最广泛的是 Watson-Jones 手术，其优良率为 80~93%，Evans 手术的优良率为 85%左右，Chrisman-Snook 手术的优良率仅为 82%[79]。虽然该类手术具有操作简单，患者近期满意率较高等优点，尤其近年关节镜技术大量用于该手术操作更是对该类手术微创、患者恢复快等光环[80]。但是大量的临床实践证实该类手术都不同程度的控制了踝关节、距下关节的生理活动。有学者报道该类术式可以导致 35%患者的中度后足内翻受限，在观察的 28 例患者中 11%的患者存在前抽屉实验大于 8mm，19%的患者存在轻度关节退变及行走困难[81]。

4.2.2.3 解剖重建手术

为了减少和避免非解剖重建带来的并发症，规避该类手术的上述缺点，当前越来越多的的医生主张采用解剖重建外侧副韧带，尽可能的将移植物固定于原有韧带止点位置，以获得良好的手术效果[81]。解剖修复的优点是无需牺牲其他有用的组织，通过移植韧带的解剖结构恢复其张力，这种术式不仅可以维持踝关节的稳定，而且恢复踝关节功能，手术操作相对简单，并发症少。尤其腔镜技术的发展，使得该类手术完全可以在关节镜下进行。华英汇等[82]采用异体半腱肌腱对 36 例慢性踝关节不稳的患者进行了外侧副韧带解剖重建，平均 37.9 个月的随访，AOFAS 评分从术前的 42.3 分提高到 90.4 分，Karlsson 评分从术前

中国知网 https://www.cnki.net

的 38.5 分提高到术后的 90.1 分，收到比较满意的临床疗效。Takahashi 等[83]则采用移植自体趾伸肌腱解剖重建踝关节外侧副韧带，观察了 13 例患者，经过 7 年的随访发现，Karlsson 评分平均为 95.4 分，同样效果满意。Vega 等[84]利用关节镜技术行全关节内、无结缝合锚钉技术治疗外侧副韧带导致的踝关节不稳 16 例患者，其中 13 例单纯距腓前韧带损伤，3 例距腓前韧带、跟腓韧带均有损伤。结果所有患者的踝关节不稳感觉均有改善，AOFAS 评分从术前的 67 分增加到术后的 97 分。因此作者认为对于踝关节不稳患者全关节镜下韧带修复是一种安全，可靠，重现性的技术，同时保留了关节镜技术的所有优点，既提供外侧副韧带复杂的解剖修复和恢复踝关节的稳定性。

该类手术移植物的选择范围较广，可以自体包括腓骨肌腱、股薄肌腱、半腱半膜肌腱、跖肌腱等、异体、人工肌腱等。自体肌腱有廉价、骨腱愈合快、本体感觉重塑迅速等优点，但也存在还需另取切口和组织、患者不易接受等缺点。异体肌腱随着低温冷冻技术的进步近年来也被越来越多用于临床，但也有产生额外费用，存在传染病毒、艾滋病等风险。人工肌腱技术也是近些年韧带修复发展的趋势之一，Usami 等[85]利用聚酯纤维的人工韧带对 436 例患者 451 个足的踝关节外侧韧带进行重建手术，经过平均 5 年 8 个月的随访。距骨倾斜从 16 度降至 4 度，前抽屉从前移 11 毫米至降至 4 毫米。在这项研究中患者在较长时间内没有出现关节的运动范围降低，骨关节炎的进一步发展等。得出结论，踝关节的韧带使用人造韧带重建经长期随访疗效优异。其的缺点是价格较高、增加患者费用、不易在基层开展、本体感觉重塑缓慢等。

综上所述对于陈旧性踝关节扭伤和由此所导致的踝关节功能性不稳定无论非手术疗法还是手术疗法，采取何种手术治疗都有一定的局限性，并且各自有着自己的优缺点。在此问题上中国的患者是幸运的，因为既可以享受中医手法的治疗又可以接受现代医学的手术治疗。中医手法治疗该类疾病虽然临床疗效明显[86, 87]，但是手法应用繁杂，缺乏规范深入细致的研究。评价标准也大都以患者的疼痛、肿胀感觉为主，没有用国际通用的评价标准和评价方法进行客观的评价。《医宗金鉴·正骨心法要旨》云："夫手法者……患者不知其苦，方称为手法也"。因此，寻找一种临床疗效明显，经得起国际通用评价标准评价，患者又没有什么痛苦的治疗方法才是我们医生的最大追求。

中国知网 https://www.cnki.net

参考文献

[1]陈兆军，唐凡启，林顺福等.踝关节韧带损伤的早期诊治[J].中国骨伤. 2007，(05):330-331.

[2]王正义.足踝外科学.北京:人民卫生出版社，2006.205-210.

[3]Maehlum S,Daljord OA. Acute sports injurise in Oslo:a one year study[J]. Am J Sports Med. 1984,18(3):181-185.

[4]王亦璁.骨关节与损伤.北京:人民卫生出版社，2007.1498-1514.

[5]Ozeki S，Yasumra K，Kaneda K,et al. Simultaneous strain measurement wit h determination of a zero strain reference for the medial and lateral ligament of the ankle[J].Foot ankle Int,2002,23(9):825-832.

[6]毛宾尧.踝关节不稳[J].中华关节外科杂志(电子版).2009(3):82-85.

[7]周捷，曲绵域，田得祥等.急性外踝韧带和关节囊撕裂[J].中国运动医学杂志.1 998(17): 307-308.

[8]孙材江.实用骨伤科手册[M].长沙：湖南科学技术出版社，2000:339.

[9]谭小平.肿痛消汤治疗早期踝关节外侧韧带损伤 90 例临床观察[J].中医药导报. 2011， (17):63-65.

[10]田伟.实用骨科学.北京：人民卫生出版社，2008:1033-1035.

[11]Marder RA.Current methods for the evaluation of ankle ligament injuries[J]. J Bone Jiont Surg(Am)，1994，76:1103-1111.

[12]唐三元.踝关节韧带损伤[J].中国矫形外科杂志.2002,11(10):1013-1014.

[13]BlackH.Roentgenographic considerations. Am J SportsMed，977，5:238-240.

[14]杨志.踝关节周围韧带损伤[J].广西医学.2004，(4):465-467.

[15]Aerts P,Disler D G. Abnormalities of the foot and ankle:MR imaging findi ng[J].Am J Ronentg 1995,165(1):119-124

[16]Verhaven EF，ShahabpourM，Handelberg FW，et al. The accuracy of three dimensional magnetic resonance imaging in the diagnosis of ruptures of the l ateral ligaments of the ankle. Am J SportsMed，1991，19:583-587.

[17]汪学松，邱贵兴，翁习生等.踝关节内外侧韧带损伤的诊断和治疗[J].中国矫形外科杂志.2008，17(04):269-271.

[18]Farooki S，Sokoloff RM，Theodorou DJ，et al. Visualization of ankle tend ons and ligaments with MR imaging: influence of passive positioning[J].Foot

中国知网 https://www.cnki.net

Ankle Int，2002，23(6):554-559.

[19]白万山，赵辉，邱晓华等.磁共振成像在踝关节外侧副韧带损伤诊断中的作用［J］.国外医学:骨科学分册，2005，26(2):112-115.

[20]Mrder RA.Current methods for the evaluation of ankle ligament injuries[J]. J Bone joint Surg(Am)，1994，76:1103-1111.

[21]Miyagi S，Tokunaga J.Stress peroneal tenography in the diagnosis of the lateral ligament ruptures of the ankle joint[J].J Jpn Soc Surg Foot，2004，25: 118-121.

[22]林发俭，冉维强，黄曼维等.踝关节侧副韧带损伤超声检查[J]. 中国医学影像技术. 2002，(18):1298-1299.

[23]Kannus P， Renstrom P. Treatment for acute tears of the lateral ligaments of the ankle. Operation， cast，or early controlled mobilization [J]. J Bone Joint Surg Am，1991，(73):305-312.

[24]贺小兵，卢卫庆，朱文忠等.手术治疗III度踝关节外侧副韧带损伤的长期随访结果［J］.中华创伤杂志，2003，19(4):241-242.

[25]唐农轩，范清宇.踝足伤病诊疗手册.人民军医出版社，2004:62.

[26]林志斌.陈旧性踝关节扭伤临床研究进展。亚太传统医药，2014,10（22）：33-34.

[27]Baumhauer JF,O'Brien T. Surgical Considerations in the Treatment of ankle instability[J]. J Athl Train, 2002,37(4):458-462.

[28]Jackson W, McGarvey W. Update on the treatment of chronic ankle instabilityand syndesmotic injuries. Ankle and foot[J].Current Opinion inOrthopedics,2006,17(2):97-102.

[29]施建东、翟文亮、庄泽民.陈旧性踝关节外侧副韧带损伤的治疗.临床骨科杂志，2010,13（4）：479.

[30]杨珍、胡亚哲. 慢性踝关节不稳的诊断与修复. 中国组织工程研究.2014,18（19）： 1434-1440.

[31]华英汇、陈世益. 慢性踝关节不稳定的外科治疗进展. 中国医学前沿杂志（电子版）. 2013,5（3）：8-11.

[32]Eamonn Delahunt. Neuromuscular contributions to functional instability of the ankle joint. Journal of Bodywork and Movement Therapies. 2007,11(3) : 20

中国知网 https://www.cnki.net

3.
[33]Birmingham TB,Chesworth BM,Hartsell HD,et al. Peak passive resistive tor que at maximum inversion range of motion in subjects with recurrent ankle in versionsprains.Journal of Orthopaedic and Sports physical Therapy. 1997,25(5):3 42.
[34]Ozeki S , Yasumura K .Ligament injuries in the ankle join t[J] .Current Opinion in Orthopedics. 1998,9(3):24 ～ 29.
[35]Takebayashi T , Yamashita T , Minaki Y , et al .Mechanosenstive af-feren t units in lateral ligament of the ankle[J] .J Bone Joint Surg (Br), 1997 , 79 : 490 ～ 493 .
[36]于涛、俞光荣.踝关节外侧不稳定的生物力学研究进展.中国骨与关节损伤杂志.2010,25（1）：94-96.
[37]Bahr R , Pena F , Shine J , et al.Biomechanics of ankle ligamentreconstru ction :an in vitro comparison of the Brostrom repair, WatsonJonesreconstruction, and a new anatomic reconstruction technique[J].AmJSportsMed. 1997, 25:424 ～ 432.
[38]Pijnenburg ACM , VanDijk CN , Bossuyt MM , et al .Treatment of ruptur esof the lateral ankle ligaments :A meta-analysis[J] . J Bone Joint Surg (Am). 2000,82:761 ～ 773.
[39]Marder RA .Current methods for the evaluat ion of ankle ligament injuries [J] .J Bone Jiont Surg(Am), 1994,76:1103 ～ 1111.
[40]Jarde O.Havet E, Gabrion A.et al. Long-term outcome following surgical o f ruptures of the fibular collateral ligament of the ankle[J]. ActaOrthop Belg. 1999,65(3):340 ～ 345.
[41]王三好.绷带固定与康复治疗踝关节损伤［J］.中国中医药现代远程教育.2009,(7):302.
[42]Karlsson J， LansingerO. Lateral instability of the ankle joint. ClinOrthop RelatRes，1992，276:253-361.
[43]Feuerbach JW， GrabinerM， Koh T， et a.l Effect of ankle orthosis and ankle ligament anesthesia on ankle joint proprioception. Am J Sports Med，1994，22:223-239.
[44]Freeman MA. Instability of the footafter injuries to the lateral ligament of

中国知网 https://www.cnki.net

the ankle. J Bone JointSurg(Br)，1965，47:669-677.

[45]Pijnenburg ACM，VanDijk CN， Bossuyt MM， et al. Treatment of ruptures of the lateral ankle ligaments: A meta-analysis[J]. J Bone Joint Surg (Am)，2000，82:761-773.

[46]黄涛，张钢林.功能治疗在运动员距腓前韧带损伤中的应用[J].福建中医药，2007，38(6):18-19.

[47]韩军良，葛勤，郭清旭.胶布固定治疗军训所致踝部外侧副韧带损 78 例[J].临床军医杂志，2004，32(6):96.

[48]唐剑邦，苏培基.跌打镇痛液治疗急性踝关节韧带损伤的临床疗效观察[J].广州中医药大学学报，2011，28(3):232-235.

[49]王敏，石仕元，赖震.踝关节外侧副韧带损伤的手术治疗[J].中国骨伤，2010，23(7): 522-523.

[50]郑金荣，郭中林.踝关节外侧韧带损伤治疗方法探讨[J]. 实用骨科杂志，2002，8(2): 136-137.

[51]Pijnenburg ACM， Bogaard K， Krips R， et al. Operative and functional treatment of rupture of the lateral ligament of the ankle. A randomised， prospective trial [J].J Bone Joint Surg Br，2003，85(4):525-530.

[52]Lacko M， Sidor Z， Stolfa S， et al. Acute injuries of lateral ankle joint ligaments [J]. Rozhl Chir，2010，89(7):461-465.

[53]Hazanas S，Galvez L，Cepes JA. Functional stabilization versus orthopedic immobility in grade Ⅰ-Ⅱ ankle spain [J]. Aten Primaria，1999，23(7):452-458.

[54]Kaikkonen A，Hyppanen E，Kannus P，et al. Long-term functional outcome after primary repair of the lateral ligaments of the ankle[J]. Am J Sports Med，1997，25:150-155.

[55]洪文跃.踝关节镜探查并距腓前韧带重建治疗习惯性踝关节扭伤[J]. 浙江创伤外科， 2011，16(1):63-64.

[56]张阳、张秋霞.功能性踝关节不稳定者的静态平衡能力[J].中国组织工程研究，2013,17（35)：6287-6292.

[57]Balduini F、Vegso J、Torg J.et al. Management and rehabilitation of ligaMentous injuries to the ankle[J]. Sports Med,2007,4:364-380.

中国知网 https://www.cnki.net

[58]何伟华、李珂、独建库，等.肌肉功能锻炼对慢性外踝关节不稳的治疗效果.实用医学杂志，2014,30（9）：1514-1515.

[59]Clark VM,A-4week wobble board exercise programme improved muscle on set latency and perceived stability in individuals with a functionally unstable a nkle.Physical Therapy in Sports.2005,6(4):181-187.

[60]Eils E,Rosenbaum D.A multi-station proprioceptive exercise program in pati ents with ankle instability. Med Sci Sports Exerc.2001,33(12):1991-1998.

[61]李坤，王予彬.踝关节不稳与本体感觉研究现状[J]. 中国微创外科杂志， 20 10,15(9): 851-854.

[62]黎奇峰.陈旧性踝关节运动损伤手法配合康复训练治疗 30 例疗效观察.中医临床研究， 2011,3（23）：77-78.

[63]胡精超，周军.推拿对延迟性肌肉酸痛作用机制的研究进展[J]. 中国康复医学杂志， 2009,24（1）：89.

[64]吴贵根，李军，刘川.推拿按摩治疗踝关节损伤的研究进展[J].光明中医，20 10,25（8）：2536-1537.

[65]杨定产.踝关节韧带损伤的治疗与功能恢复训练[J].长沙大学学报，2002,16（4）：90-91.

[66]Yamamoto H,Kazuyoshi Y,Ogiuchi T, et al. Subtalar instability following la teral ligament injuries of the ankle.1998.29(4):265.

[67]Sammarco Ⅵ.Complications of lateral ankle ligament reconstruction. Clin Orthop Relat Res.2001.397:123-132.

[68]Baltopoulos p. Midterm results of a modified Evans repair for chronic late ral ankle instability. Clin Orthop Relat Res.2004.422:180-185.

[69]Vries JS.KRIPS r.Blankevoort L.et al. Arthroscopic capsular shrinkage for c hronic ankle instability with thermal radiofrequency; prospective multicenter tri al[J]. Orthopedics. 2008,31(7):655.

[70]Nery C, Raduan F, Del Buono.et al. Arthroscopic-assisted BrostrÖm-Gould for chronic ankle instability; a long-term follow-up[J].Am J Sports Med.2011, 39(11):2381-2388.

[71] Acevedo J I, Mangone P.Ankle. Instability and Arthroscopic Lateral Liga ment Repair[J]. Foot and ankle clinics. 2015, 20(1): 59-69.

[72]Brostrom L. Sprained ankles. Part Ⅲ.Clinical observations in recent ligame

中国知网 https://www.cnki.net

nt ruputures. Acta Chir Scand.1965,130:560.
[73]Giza E,Nathe R,Nathe T,et al.Strength of bone tunnel versus suture anchor and push-lock construct in BrostrÖm repair[J].Am J Sports Med. 2012,40(6):1419-1423.
[74]Aydogan U,Glisson RR,NunleyJA. Extensor retinaculum augmentation reinforces anterior talofibular ligament repair[J]. Clin Orthop Relat Res.2006,422:210-215.
[75]Karlsson J,Bergsten T,Lansinger O,et al. Reconstruction of the lateral ligaments of the ankle for chronic lateral instability[J]. J Bone Joint Surg.1988,70A;581-588.
[76]Sammarco VJ. Complications of lateral ankle ligament reconstruct-tion[J].Clin Orthop Relat Res.2001,397:123-132.
[77]徐琦，张立新.改良 Brostrom 术式治疗慢性踝关节外侧不稳 21 例疗效分析[J].中国骨与关节损伤杂志.2011,26（2）：164-165.
[78]周建刚.改良 Chrisman-Snook 手术治疗慢性踝关节不稳[J]. 现代医药卫生. 2005,21(20):2792-2793.
[79]王金辉，蒋协远，武勇等. 慢性踝关节外侧不稳定.中华创伤骨科杂志，2006,8（5）：468-471.
[80]Klammer G, Schlewitz G,Stauffer C,et al. Percutaneous lateral ankle stabilization: an anatomical investigation[J]. Foot Ankle Int.2011,#@(1)66-70.
[81]Baltopoulos P,Tzagarakis GP,Kaseta MA. Midterm results of a modified Evans repair for chronic lateral ankle instability[J]. Clin Orthop Relat Res.2004,422:180-185.
[82]Hua Y,Chen S Jin Y,et al.Anatomical reconstruction of the lateral ligaments of the ankle with semitendinosus allograft[J]. Int Orthop. 2012, 36(10): 2027-2030.
[83]Takahashi T,Nakahira M,Kaho K,et al.Anatomical reconstruction of chronic lateral ligament injury of the ankle using pedicle tendon of the extensor digitorum longus[J].Arch Orthop Trauma Surg.2003,123(4):175-179.
[84]Vega J, Golanó P, Pellegrino A, et al. All-inside arthroscopic lateral collateral ligament repair for ankle instability with a knotless suture anchor technique [J]. Foot & ankle international, 2013, 34(12): 1701-1709.
[85] Usami N, Inokuchi S, Hiraishi E, et al. Clinical application of artificial li

中国知网 https://www.cnki.net

gament for ankle instability—long-term follow-up[J]. Journal of long-term effects of medical implants, 2000, 10(4).
[86]高景华、高春雨、孙树椿，等.摇拔戳手法治疗陈旧性踝关节扭伤 34 例. 世界中医药，2011,6（3）：214-215.
[87] 李俊海、王庆甫、黄沪. 正骨手法与中药熏洗治疗陈旧性踝关节扭伤的病例对照研究. 中国骨伤，2012,25（2）：113-115.

中国知网 https://www.cnki.net

第二部分孙树椿教授学术思想和临床经验的整理与研究

孙树椿教授是著名骨伤科专家，国家骨伤科重点专科学科带头人，第一批国家级非物质文化遗产-----中医正骨疗法的传承人，其手法主要源于清宫廷正骨派，师出京城著名宫廷正骨中医刘寿山老先生[1]。孙树椿教授长期从事中医骨伤科医疗、科研、教学一线工作，在多年的临床工作中，孙树椿老师尊古而不泥古，博采大江南北诸家名医之长，骨伤治疗手法真正做到了“机触于外、巧生于内、手随心转、法从手出”，形成了鲜明学术风格，主要表现在以下几点：

1 中医骨伤科治病的主体是人而不是各个器官，所以治病要求本，以人为主

中医骨伤科学是研究人体各部位损伤和运动系统疾病的预防、诊断、治疗和康复的一门临床医学学科。它承载着中国古代人民同疾病作斗争的经验和理论知识，是在古代朴素的唯物论和辨证法思想指导下，通过长期医疗实践逐步形成并发展成的医学理论体系。中医认为人与自然界是一个统一的整体，即“天人合一”、“天人相应”。人的生命活动规律以及疾病的发生等都与自然界的各种变化（如季节气候、地区方域、昼夜晨昏等）息息相关，因此在诊断、治疗同一种疾病时，多注重因时、因地、因人制宜，并非千篇一律。孙老认为[2]人体各个组织、器官共处于一个统一体中，不论在生理上还是在病理上都是互相联系、互相影响的。因此不能孤立地看待某一生理或病理现象，头痛医头，脚痛医脚，而应多从整体的角度来对待疾病的治疗与预防，特别要强调“整体观”。

孙树椿老师认为中医的“中”不只是一个相对于西医的方位、国别或民族的概念，而应是《中庸》里所说的“致中和”的“中”[3]。中医的最高境界就是对“中”的理解和运用。中医用精气学说、阴阳学说和五行学说，来解释生命的奥秘。中医治病就是调理阴阳平衡的过程，阴阳平衡是世界万物存在的最理想状态。中医骨伤科是在中医理论指导下的一门学科。虽然我们接触的是运动系统的骨骼、筋脉，但要明白这些都是人体的一部分，人才是疾病的主体，人是活体有自身修复能力，医生只是为人体自身修复创造最有利的条件，千万不能把人体当做机器进行部件的修理。疾病的治疗除要求恢复解剖位置外，更期望人的运动功能恢复正常。比如肢体骨折的处理，应该首重伤肢的功能恢复，而不是为了达到所谓的骨的“解剖对位”而不顾可能会影响功能的其他因素。

中国知网 https://www.cnki.net

另外，还要看到整体与局部的关系问题，人体全身强壮有利于局部骨折的愈合，肢体局部功能的恢复正常又会反过来促进机体的更加健康。

2 骨伤科是中医骨伤科，辨证施治是根本，但是也要辨病、辨证相结合

骨伤科学是在中医理论指导下的一门学科，辨证施治是根本，但是又有其自己独特的诊病方法，孙老认为有病就有证，辨证才能识病，两者是密不可分的。临床诊治时，既要辨病，又要辨证，只有病、证合参，才能选用适当方药，恰当的手法。辨证是在中医学的基本理论指导下,对疾病发展过程中某阶段的病位、病性等本质的概括,揭示病因、病位、病性、病势,为治疗提供依据。例如脊髓型颈椎病，一般临床医生大都主张手术治疗［4、3]，脊髓型颈椎病虽然都有走路踩棉感、束带感等症状和病理反射阳性、霍夫曼氏症阳性等体征，但中医骨伤科根据患者的临床表现不同而分为痿证和痹证，并采取不同的治疗方法。临床上患者感觉以“疼、麻、凉”为其特点，属“痹证”，运用舒筋活络、活血止痛、理筋整复的手法和药物等，使局部气血通畅，改变脊髓与致压物的解剖关系，松解局部粘连，减轻了脊髓受到的压迫，症状可缓解或消失，完全可以非手术治疗。而肌肉萎缩、麻痹者属“痿证”，“痿证”型则建议手术治疗[6]。

在学术上孙老提出：“腰椎间盘突出”不等于“腰椎间盘突出症”，腰椎间盘突出以往都是手术治疗，大量临床实践证明，运用中医的手法、中药等治疗方法解除了神经根的压迫和刺激，一大批患者的临床症状随之减轻或消除了，影像学上虽然有髓核突出，但临床上已没有症状，使患者免除了手术的痛苦。另外，“骨质疏松”也不等于“骨质疏松症”，人变老或因雌性激素水平下降，就会“骨质疏松”，但临床上大多数的人并没有症状，骨质疏松重在预防，骨质疏松症才须要治疗，预防并不是要补钙，而是应主动的功能锻炼。

“骨质增生”、“骨刺”不等于骨性关节炎，随着人们年龄不断老化，骨的退变、增生也会逐年加重，但这和“骨性关节炎”的发病率并不成正比，老化是不可逆的，我们治好了局部的炎症，骨刺还在，而产生的症状完全消失了。这些观点从中医角度提出了辨证、辨病的思路，为中医药治疗提供了理论依据，不仅得到中医骨伤界的广泛认同，也对西医骨科产生了深刻的影响，减少了过度医疗的情况。

中国知网 https://www.cnki.net

3 骨伤科治病首重手法，而手法治疗要寻找“筋结”，并讲究轻、巧、柔、和

骨伤科治病尤重手法，而手法治疗必须对人体正常筋骨的结构关系有一个清楚的了解，所以《医宗金鉴·正骨心法要旨》说：“必先知其体相，识其部位”。古代由于解剖学知识缺乏，为了解人体构造和病变部位，只能靠手来寻找，做到心中了然。现代医学的发展，解剖学知识的丰富，为“手摸心会”提供了可靠详实的基础。

孙树椿教授根据自己有10多年手术经历的特点，将中医骨伤科的“手摸心会”与现代解剖学相印证，提出手法治疗要找到病变部位，这和临床上查体不完全一致。病变部位一定有“筋结”的出现。“筋结”是筋伤以后，由于局部的出血、渗血、水肿、炎性细胞侵入，形成的“无菌性炎症”，随着组织的逐渐修复，纤维组织增生而形成的。不同的组织损伤，形成的痛性硬结，形状不一，但呈现规律性。在长期临床实践中，探索总结出了各种疾病筋结的位置和形状[7]。如神经根型颈椎病常可在相当于颈椎第5、6节水平的椎旁软组织；颈性眩晕常可在相当于颈椎第3、4节水平的椎旁软组织；腰肌劳损可在一侧或双侧第三腰椎横突处；腰椎间盘突出症可在病变节段的棘突旁等处找到筋结；针对“筋结”进行治疗，可取得事半功倍的效果。

孙树椿教授作为宫廷正骨手法的代表，治病手法讲究“轻、巧、柔、和”。老师最欣赏的一句话就是《医宗金鉴·正骨心法要旨》中的：“法之所施，使患者不知其苦，方称为手法也”。手法特别讲究力的运用，包含以下几个层面：第一是施术者要有功力、穿透力，具有聚全身气力于一点的威力，作用力要能深达患者的经穴、脉络、筋骨；注重以内心感觉引导动作，以丹田配合力的蓄发，施手法时虽出于手，实发以心，心手相合，从而提高以气催力的能力，做到手法稳健，柔中带刚，手到而病除。正所谓“机触于外，巧生于内，手随心转，发从手出”。第二是用力要轻巧，掌握好发力时机；在施治患者时，患者若有紧张对抗时，则应用一些技巧，转移其注意力，如指导患者行吐纳呼吸调整气息，放松情绪，当患者到吸气时，骤然发力。第三是要学会“借力”。如在做颈椎旋转手法时，术者要轻提患者颈部并让患者做颈部旋转动作，待旋转至某一侧有固定感时，以腰带肘，迅速发力旋转，借力发力，此时即可听到一连串的关节弹响声。手法临证，讲究“轻、巧、柔、和”[8]。

中国知网 https://www.cnki.net

4 骨伤科疾病首重气血，但瘀血为因、气滞为果，血瘀才是病机根本

治疗骨伤科疾病一般的医家都重视气血，大都认为气滞血瘀是病理基础，治疗也大都以行气活血为主。但是孙老通过大量的临床实践发现无论是疾病的急性期或慢性期，疼痛、肿胀往往是患者的主要症状，损伤后，由于血离经脉，血瘀筋脉，经络受阻，气血流通不畅，从而产生疼痛、肿胀。这说明无论伤筋还是伤骨，离经之血形成的瘀血才是病机之初的根本。所以患者早期的表现主要以疼痛为主，痛有定处，固定不移。日久瘀血影响气机条达，然后才出现气滞。因此，对骨伤科疾患辨证并不是传统意义的“气滞血瘀”，而应该是“血瘀气滞”。无论手法还是药物都重视瘀血的祛除，特别是通过一些特定的手法则可以促进局部血液和淋巴的循环，加速局部瘀血的吸收，改善局部组织代谢，理顺筋络，并可以提高局部组织的痛阈，使气血通畅，从而起到舒筋活络、消肿止痛的作用，这也就是“通则不痛”、“通则气机条达”的道理。正如《医宗金鉴·正骨心法要旨》所说：“为肿为痛，宜用按摩法，按其经络，以通郁闭之气，摩其壅聚，以散郁结之肿，其患可愈”。

5 筋伤疾病也讲动静结合，尤其重视功能锻炼的重要性

孙老主张在筋伤的治疗中，动静结合也同样有着实际的临床意义。伤后经络受阻，气血瘀滞，血肿形成，引起疼痛和功能障碍。因此筋伤的愈合局部需要固定一段时间，以利于筋伤的修复，这即是“静”。同时，由于血肿形成，若瘀血不去，日久气血凝滞，血不荣筋，容易导致筋肉挛缩、疼痛、活动受限等并发症。因此，除一定时间的“静”外，也需要注重局部及全身的功能锻炼，使气血畅通，筋肉得养，这即是“动”。人体是活体，活动是根本，静止固定只是治疗疾病的必要手段和阶段。 孙老在临床上还非常重视练功疗法，如在临床上第三腰椎横突综合征（腰肌劳损），除了手法和药物治疗之外，同时要求患者进行腰部练功以加强腰背肌功能。“燕飞”是腰背肌锻炼的主要方法。其具体动作是：患者俯卧位，四肢伸直，两腿并拢。头、上身及双下肢同时缓缓背伸，双手自然后伸，令整个身体后伸成一自然弧形线，同时吸气，其形如飞燕翔空。停留片刻后呼气还原。每日早晚各 1 次，以练后症状不加重为度，坚持半年至一年左右，临床效果良好。对颈椎病则提出“与项争力”、“哪吒探海”、“回头望月”、“以头

中国知网 https://www.cnki.net

书鳯”等锻炼方法。对于足跟“骨刺”，指导患者跺脚、用力要求稍有痛、麻的感觉，每次 81 下，每日二次，一般可达“痊愈”。这实际上是种自我按摩的方法。在治疗的同时，重视“功能锻炼”，强调“生命在于运动”，也充分体现了“治未病”的思想[9]。

6 手法与药物并重，内外用药结合

孙树椿老师在疾病强调手法治疗的同时，也应重视药物的配合应用。可从西医角度诊断其为何种疾病，然后按中医理论进行辨证，疾病不同、中医的证不同，药物的运用也有所不同。即使是同一损伤，也要根据患者的年龄、性别、体质灵活运用。例如神经根型颈椎病，中医辨证大多数属于血瘀气滞、脉络闭阻证。针对此病证，孙老配制了由三七、川芎、延胡索、白芍、威灵仙、葛根、羌活七味药物组成的颈椎Ⅱ号方。方中三七化瘀止血，活血定痛，以祛除在经之瘀血，为君药；川芎、延胡索活血行气，祛风止痛，“气行则血行”敌为臣药；白芍养血敛阴、柔肝止痛，威灵仙、羌活祛风湿、通经络、止痹痛，葛根发表解肌、升阳透疹，共为佐使之品。合方共奏活血化瘀、祛风除湿、行气止痛之功[10]。而对于椎动脉型颈椎病，其临床主要表现为眩晕及自主神经紊乱如恶心呕吐等症状，中医辨证多属于肝阳上亢。针对此病证，孙老制定了颈椎III号方。方中天麻、钩藤平抑肝阳、息风镇惊；川芎、延胡索、白芷、细辛活血行气、祛风止痛；葛根、黄芩清热解肌，诸药共奏平肝潜阳、祛瘀止痛之功。孙老还强调内服药物与手法治疗相结合，以达到局部与全身结合临床上大都收效甚佳。孙老非常重视中药外用熏洗的应用，其认为外用膏药、散剂、熏洗剂这些不仅是中医骨伤科的特色也是老祖宗留给我们的重要遗产。孙树椿教授应用中药外洗治疗膝关节炎、关节损伤等病每每 2 到 3 剂即可见效，其中奥妙就是辨证施治。外用药许多医生无论何病，也不管寒、热、虚、实均一个方子从之，临床收效甚微。孙老师告诫我们中医骨伤科外用药也讲辨证，寒者热之、热者寒之，不进行辨证，也不问寒热虚实，热衷于什么验方、秘方岂不是胡乱为之，南辕北辙。

7、外踝理筋手法渊源及特色

清宫正骨派理筋手法中认为，踝关节的前、后、左、右以及前外、前内、后外、后内存在八个面的“踝缝”。这些“踝缝”往往是踝关节扭伤后的发病点，

中国知网 https://www.cnki.net

也是手法治疗的着重点。因此手法围绕这八个踝缝展开，又俗称“八面缝”手法。具体分为踝前侧手法、踝内侧手法、踝外侧手法。踝前侧手法又分为踝前手法、踝前外手法、踝前内手法等[11]。

对于踝关节陈旧性损伤的治疗孙老师主张临床查体一定要和解剖知识相结合。踝关节陈旧性损伤最容易伤及的就是踝关节距腓前韧带，但是临床往往又不单纯是该韧带的损伤。踝关节一旦损伤外侧所有副韧带大都不能幸免，所以临床检查时一般在距腓前韧带周围可以触及豆粒样大小的“筋节”，在按压这些“筋节”时患者会感到剧烈的疼痛。但是这往往就是治疗该病的关键点所在。这些“筋节”需要施术者查体时要慢慢体会，也即是“手摸心会”。在踝关节扭伤的手法治疗中，孙氏手法不主张运用按压、滚揉等刺激局部软组织的手法，而主张在轻揉“筋节”的同时运用特色的“摇、拔、戳”手法进行治疗。“摇”法是指术者在助手的对抗牵引下，双手握住关节远端顺时针摇动关节的手法；“拔”法即牵引、拔伸的意思；“戳”法是宫廷正骨的独特手法，为术者握住肢体远端向关节内戳按的手法。摇法可以使因外伤导致的踝关节周围痉挛的软组织得到放松，从而使踝关节解除“交锁”；拔法通过拔伸、牵引局部痉挛的软组织，使迂曲、挛缩的毛细血管运行畅通，从而改善了局部的血液循环；戳法充分利用踝穴的作用，使发生轻微移位的踝关节复位，并能使卡压于关节内的滑膜等组织解除嵌顿。并且孙老师还主张好的理筋手法不需要施术很长时间。此外，孙老师在强调手法治疗的重要性的同时，绝不排斥西医手术治疗。选择何种治疗方法不应该分西医、中医，而是根据患者的临床需要而定。对于踝关节陈旧性损伤踝关节存在机械性不稳定者或经检查证实韧带有撕裂者孙老师同样也主张行手术治疗。

总之，孙老师近60年的临床一线工作经历，学术特色何止这区区几个方面，本人作为国家中医药管理局第 5 批名老中医传承人有幸跟随孙老学习，短短 3 年时间接触孙树椿教授的学术思想只不过是冰山一角，由于自己水平问题，对孙老师的学术经验总结还很片面，也难免挂一漏万，今后还需潜心学习进一步多跟师临床实践，在临床实践中总结发现老师的宝贵经验和学术思想，使老师的学术思想总结更加完善。

中国知网 https://www.cnki.net

参考文献

[1]北京中医药大学东直门医院.刘寿山正骨经验[M].北京：人民卫生出版社，2009:2.

[2] 孙树椿.中医药治疗颈痛.北京：人民卫生出版社，2002:81-84.

[3]高景华.以自然之道，养自然之身.中国中医药报，2010，12、23

[4]钟卓霖　胡建华 翟吉良，等. 伴肌萎缩颈椎病的手术治疗效果. 中华骨与关节外科杂志，2015,8（3）：209-213.

[5]戴力扬. 脊髓型颈椎病的病因与病理. 中国矫形外科杂志，2005,12（24）：1899-1901.

[6] 孙树椿.骨伤名师二十三讲.北京：人民卫生出版社，2008:159-161.

[7]高景华，张军. 孙树椿筋伤疾病诊治经验. 北京：中国中医药出版社，2014,12-13.

[8] 孙树椿. 清宫正骨手法图谱，北京：中国中医药出版社， 2012：:24-309.

[9] 孙树椿、孙之鎬. 临床骨科学（第二版）. 北京：人民卫生出版社，2014，144-148.

[10]陈兆军，王庆甫，王尚全.机处于外而巧生于内手随心转法从手出.现代中医临床，2014,21（1）：3538.

[11] 孙树椿. 清宫正骨手法图谱. 北京：中国中医药出版社，2012,209-217.

中国知网 https://www.cnki.net

第三部分 孙树椿教授外踝理筋手法治疗陈旧性踝关节扭伤的临床研究

1. 前言

踝关节是人体负重、行走的重要结构，人体之所以能够与凹凸不平的地面垂直，完全依赖于踝关节的特殊结构[1]。踝关节韧带是维持踝关节稳定的重要结构，其损伤在临床上非常多见，发生率在全身关节韧带损伤中居第一位[2]，约占80%以上，可发生于任何年龄，以青少年多见。该病若早期处理不当，常遗留疼痛，可严重影响踝关节功能，甚至成为慢性疾患，后期发生踝关节不稳，反复扭伤，并可继发骨关节炎等[3]，形成不可逆的病理变化，故应引起高度重视。

踝关节扭伤在中医伤科辨证中属“筋伤”的范畴。踝关节扭伤分为急性和陈旧性损伤。陈旧性损伤多为急性踝关节扭伤后失治、误治或积劳成疾所致[4]。中医认为踝关节陈旧性损伤的病理机制为瘀血未化、经络闭阻、气血运行不畅，导致踝关节周围软组织筋脉失养，以局部疼痛、肿胀为主要表现。中医骨伤科认为踝关节扭伤就属于“筋出槽”与“骨错缝”的范畴。踝关节扭伤后使踝关节周围的肌腱、韧带部分撕裂或断裂，脱离了正常的舒缩轨道，而导致筋伤出槽；筋出槽后不能维持原来正常的解剖位置，骨失去筋的牵拉维系处于非生理、不正常吻合状态，表现为骨错缝。因此，在临床上运用中医手法治疗该病是主要手段[5, 6, 7]。

孙树椿教授通过自己多年的临床经验得出对于陈旧性踝关节扭伤手法治疗不宜采取重力按揉等手法，因为手法使用不当不仅不会治疗该病，还会加重踝关节局部的瘀血阻络状态，而更加导致局部气血运行不畅，使患者局部疼痛、肿胀更加严重。孙老认为和新鲜踝关节扭伤不同的是，在陈旧性踝关节扭伤患者的踝关节周围，特别是外踝处一般可触及较硬的“筋结”，此处往往是患者的疼痛最明显处，这也正是治疗该病的关键点。建议采用轻、巧、柔、和的点揉手法对存在的“筋结”进行轻轻的按揉，然后进行踝关节拔、摇、戳外踝理筋手法治疗，往往可以收到临床满意疗效[8]。尽管临床比较满意，但有关该手法的规范研究还是空白。因此，我们设计了本项目课题的应用研究，拟采取随机对

中国知网 https://www.cnki.net

照的研究方法，设置扭伤后踝关节功能康复疗法为对照组，观察两组患者治疗前后疼痛 VAS 评分、AOFANS 评分、应力位踝关节 X 线片踝穴宽度等指标测评，目的在于通过科学观察、评估清宫外踝理筋手法对陈旧性踝关节扭伤的临床疗效，较为客观地判定、验证中医手法治疗临床常见病的有效性，进一步提高踝关节陈旧性扭伤的临床诊治能力。有关结果汇报如下。

2 资料和方法

2.1 病例及课题来源

从 2012 年 8 月～2015 年 10 月来孙老师门诊、我院骨科门诊及北京市朝阳区金盏社区卫生服务中心（本人每周在此出门诊）就诊的陈旧性踝关节扭伤患者。本研究作为首都卫生发展科研专项项目-----“肌骨超声监视下清宫外踝理筋手法治疗陈旧性外踝关节扭伤的临床应用研究”的部分内容。

2.2 样本量计算

经前期临床观察及相关文献查阅研究（文献来源为中国期刊网，检索主题为“踝关节扭伤”，关键词为“按摩手法”、“功能锻炼”，所得期刊文章及相关毕业论文共 69 篇），初步评估：对于陈旧性踝关节扭伤，功能锻炼治疗的有效率约为 70%，即 P2=0.7；清宫外踝理筋手法治疗的有效率约为 90%，即 P1=0.9，采用优效性、等试验样本大小的计算公式，按显著性检验 α =0.05，检验效能 1-β =0.9，临床意义的高限 δ =0.05,欲进行临床试验对比观察，计算样本量 94 例，考虑其他因素，按 15%脱落率，计算样本量为 108 例，则每组需要观察 54 例。

计算公式：$n=(Z1-\alpha+Z1-\beta)2^{[P1(1-P1)+P2(1-P2)]}/(\varepsilon-\delta)2$

2.3 诊断、纳入及排除标准

2.3.1 陈旧性踝关节扭伤诊断标准

参照中华人民共和国中医药行业标准《中医病证诊断疗效标准》[9]制定：

①有明确的踝部扭伤史；

②扭伤时间在 3 周以上；

③踝关节疼痛、无力，不能久行，影响生活、工作和运动；

④内踝或外髁前下方处可有不同程度的肿胀和压痛，或可触及痛性“筋结”。

⑤X 线片未见骨折和脱位。

中国知网 https://www.cnki.net

2.3.2 纳入标准

①第一诊断必须符合陈旧性踝关节扭伤的患者；

②经肌骨超声影像检查无踝关节韧带完全断裂；

③年龄 16 岁以上，65 岁以内（含 65 岁）；足部无其它畸形。

④当患者同时具有其他疾病诊断时，但在治疗期间既不需特殊处理，也不影响第一诊断的临床研究流程实施时，可以入组；

⑤患者适合并接受中医手法门诊或病房治疗；

⑥同意参加本研究，并签署知情同意书。

2.3.3 排除标准

①年龄在 16 岁以下或 65 岁以上者；

②合并有心、脑、肝、肾和造血系统等严重危及生命的原发性疾病；

③合并皮肤软组织破损的踝关节扭伤或皮肤病者；

④合并风湿、类风湿、痛风等疾病影响踝关节活动者；

⑤需手术修补的韧带断裂者或合并周围神经损伤者；

⑥合并精神疾病或老年痴呆患者。

2.3.4 剔除标准

①由于不符合纳入标准，而入选的患者；

②在治疗期间因其他疾病，不适合再接受手法治疗者；

③在治疗期间发现有其他部位损伤者；

④在治疗期间患者接受手术治疗者。

2.4 治疗方案

2.4.1 研究分组

采用随机对照单盲设计方法进行研究，将符合纳入标准病例随机分为手法组与对照组，其中对照组行功能疗法锻炼治疗。

2.4.2 手法组——采用清宫外踝理筋手法治疗

清宫外踝理筋手法为一套路手法，操作时患者侧卧，伤肢在上，助手握住伤侧肢体近端，勿使摇动，具体手法包括：

①手摸心会：在外踝处寻找 “筋结”（图 1）

②手法理筋：采用轻、巧、柔、和的手法对 “筋结” 进行按揉；待 “筋结”

中国知网 https://www.cnki.net

变软后以特色的踝关节拔、摇、戳手法理筋。

③拔法（图 2）：医者与助手相对拔伸，并将足跖屈内翻；摇法（图 3）：医者两虎口相对，双手拇指按住外踝缝，余四指拿住患足，将足环转摇晃 6 次；戳法（图 4）：再将足背伸外翻，双手拇指同时向下戳按。

④轻捋收功：最后沿着肌腱韧带走行方向捋顺筋脉。

手法操作每次摇拔戳 7 次，治疗隔 2 日 1 次，4 次为一疗程，共计 12 天。

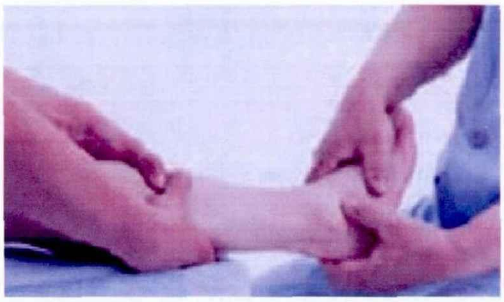

图1：按揉筋结

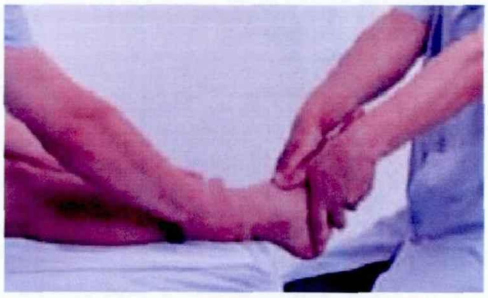

图2：摇法

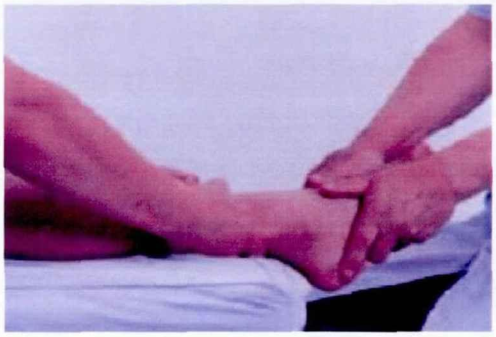

图3： 拔伸关节法

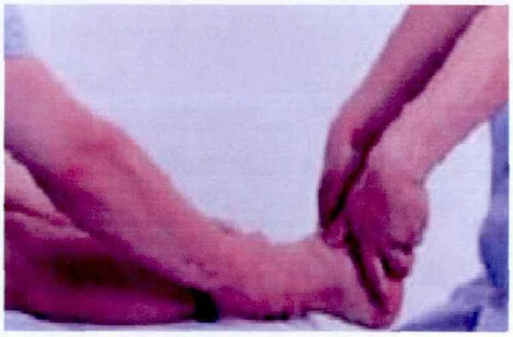

图4：戳按关节法

2.4.3 对照组 对照组采用踝关节功能疗法治疗，具体包括：

①足背伸

目的：锻炼胫骨前肌，胫骨前肌能背伸距小腿关节及内翻足。

动作要领：身体直立，以正常脚为支撑脚，让患肢足背伸至疼痛能够耐受处保持 20 秒，3-5 次/组，每日 3 组。

注意事项：支撑腿保持直立，患肢膝关节伸直，患肢足趾尽量向上翘起，足后跟用力下压。

②足跖屈

中国知网 https://www.cnki.net

目的：提高胫骨后肌、小腿三头肌的力量。

动作要领：身体直立，以正常脚为支撑脚，让患肢足跖屈至疼痛能耐受处保持60秒，3-5次/组，每日3组。

注意事项：支撑腿保持直立，患肢膝关节伸直，患肢足趾尽量下压。

③踝关节内外翻运动

目的：拉伸踝内外侧的韧带，提高腓骨长短肌及胫骨前肌的力量。

动作要领：身体直立，正常脚为支撑脚，让患踝内翻和外翻至疼痛处保持20秒，3-5次/组，每日3组。

注意事项：以足内侧缘和外侧缘为着力点,踝部用力向内和向外压，控制在疼痛的范围内。

④提踵练习

目的：主要是提高小腿三头肌肌群的力量。

动作要领：身体直立，双脚并拢，以脚尖为着力点，原地连续提踵,同时双手顺势前交叉摆动。双手收为一节，双手后展且踮脚为一节，两节为一次，连续10次为一组，5-7组，每组休息5秒。

注意事项：双手维持身体平衡，两腿膝关节伸直，脚尖用力，注意手与脚的配合。

以上功能疗法每次锻炼各动作也同样做7遍，隔2日锻炼1次，12日为一疗程。

2.5 随访方法

2.5.1 随访流程及要求

①于入组当天即进行各项治疗。对治疗前、手法治疗或功能疗法锻炼后即刻、一疗程治疗结束后及治疗后 1 个月、末次随访时，第三方进行患者问卷调查，填写病例观察表（CRF）、并进行疼痛VAS评分。访视视窗±2天。

②对治疗前、末次随访时，第三方进行患者问卷调查，完善病例观察表（CRF）、并进行AOFANS的Baird-Jackson踝关节评分[13]，访视视窗同样为±2天。

③对治疗前、末次随访时，由专人拍摄踝关节内翻应力位 X 线正位片，测量距骨斜角。进行治疗前后对比。

随访方式通过电话、微信、门诊随访、家中走访等。

中国知网 https://www.cnki.net

2.5.2 随访指标

2.5.2.1 一般项目

受试者编码、姓名和/或拼音首字母，试验开始日期，住址，联系电话，患者身份证号。

2.5.2.2 一般生物学指标

人口学特征：性别，年龄，身高，体重；

生命体征：体温，静息心率，呼吸，血压。

2.5.2.3 疗效性观测指标

①VAS 评分

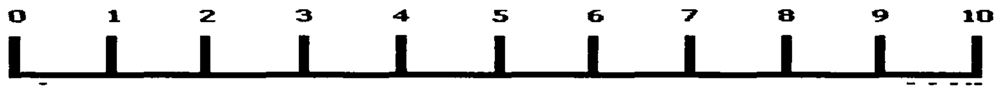

疼痛视觉模拟评分法(Visual Analogue Scale, VAS)是将疼痛的程度用 0 至 10 共 11 个数字表示，0 表示无痛，10 表示最痛，病人根据自身疼痛程度在这 11 个数字中挑选一个数字代表其疼痛程度。

所有患者治疗前均有不同程度的外踝疼痛，活动后疼痛更加加重，休息后少轻。在外踝前方均可触及大小不等的“筋结”，并且触碰“筋结”时疼痛会明显加重 2 个等级分。

② Baird-Jackson 踝关节评分[10]：

AOFAS踝关节 Baird-Jackson 踝关节评分系统包括踝关节疼痛评分满分 15 分、踝关节稳定性满分 15 分、踝部行走能力满分 15 分、跑步能力满分 10 分、工作能力满分 10 分、运动能力满分 10 分、放射线改变满分 25 分。这 7 方面是相辅相成的关系，所以需要综合评分才能够较客观的反应踝关节的功能情况。

Baird-Jackson 踝关节评分系统	分数	计分
1.踝关节疼痛（pain）：共 39 分		
A. 无痛(No pain)	15 分	
B. 大运动量活动时轻度疼痛(Mild pain with strenuous activity)	12 分	
C. 日常活动时疼痛(Mild pain with activities of daily living)	8 分	
D. 负重时疼痛(Pain with weight-bearing)	4 分	
E. 休息时疼痛(Pain at rest)	0 分	
2.踝关节稳定性（Stability of ankle）：共 20 分		
A. 无临床不稳定(No clinical instability)	15 分	
B. 运动时不稳定(Instability with sports activities)	5 分	
C. 日常活动时不稳定(Instability with activities of daily living)	0 分	

中国知网 https://www.cnki.net

3.行走能力（Ability to walk）：共 39 分		
A．随意行走时无跛行或无痛(Able to walk desired distances without limp or pain)	15 分	
B．随意行走时有轻度跛行或疼痛(Able to walk desired distances with mild limp or pain)	12 分	
C．行走能力中度受限(Moderately restricted in ability to walk)	8 分	
D．仅能走短距离(Able to walk short distances only)	4 分	
E．不能行走(Unable to walk)	0 分	
4.跑步能力（Ability to run）：共 27 分		
A．能无痛随意跑步(Able to run desired distances without pain)	10 分	
B. 随意跑步时轻度疼痛(Able to run desired distances with slight pain)	8 分	
C．跑步能力中度受限,轻度疼痛(Moderate restriction in ability to run, with mild pain)	6 分	
D．只能跑短距离(Able to run short distances only)	3 分	
E．不能跑步(Unable to run)	0 分	
5.工作能力（Ability to work）：共 27 分		
A．能从事一般职业(Able to perform usual occupation without restrictions)	10 分	
B．能从事一般职业,但某些强劳动受限(Able to perform usual occupation with restrictions in some strenuous activities)	8 分	
C．能从事一般职业,但某些受限(Able to perform usual occupation with substantial restrictions)	6 分	
D．部分残疾,只能选择工作(Partially disabled; selected jobs only)	3 分	
E．不能工作(Unable to work)	0 分	
6.踝关节运动（Motion of the ankle）：共 21 分		
A．无损伤踝的 10 度以内(Within 10° of uninjured ankle)	10 分	
B．无损伤踝的 15 度以内(Within 15° of uninjured ankle)	7 分	
C．无损伤踝的 20 度以内(Within 20° of uninjured ankle)	4 分	
D．小于无损伤踝 50%或背曲<5 度(<50% of uninjured ankle, or dorsiflexion <5°)	0 分	
7.放射线结果（Radiographic result）：共 55 分		
A．踝穴正常(内侧和关节上间隙正常,距骨无倾斜){Anatomical with intact mortise(normal medial clear space, normal superior joint space, no talar tilt)}	25 分	
B.关节边缘轻度创伤反应性改变,余同 A(Same as A with mild reactive changes at the joint margins)	15 分	
C．上关节间隙狭窄测量<2mm,或距骨切线>2mm （Measurable narrowing of the superior joint space,with superior joint space >2 mm, or talar tilt >2mm）	10 分	
D．上关节间隙中度狭窄,2~1mm 之间(Moderate narrowing of the superior joint space, with superior joint space between 2 and 1 mm)	5 分	

中国知网 https://www.cnki.net

E．上关节间隙严重狭窄<1mm,内侧间隙增宽严重创伤反应(软骨下骨硬化和骨赘形成){Severe narrowing of the superior joint space, with superior joint space < 1 mm, widening of the medial clear space, severe reactive changes (sclerotic subchondral bone and osteophyte formation)}	0 分	
能得到的最大分数 Maximum possible score	100 分	
注明:结果评定标准:优 96~100 分；良:91~95 分；可:81~90 分；差:0~80 分。<br>* Excellent = 96 to 100 points, good = 91 to 95 points, fair = 81to 90 points, and poor = zero to 80 points.		

③距骨斜角测量

本组患者全部在统一条件下有一名放射科医生进行摄片操作。特殊设备下让患者尽力内翻踝关节，以患者最大耐受为度。测量角度有计算机自动读出。具体拍摄方法：髋、膝关节屈曲 90°，踝关节跖屈 30°摄片，摄片条件:管电压 72kv，管电流 50mA.S，辅助内翻设备为我们自行设计（具体见下图），内翻最大角度为患者最大耐受疼痛为度。

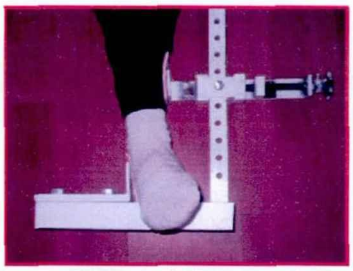

图 5　内翻应力拍摄 X 片设备

测量方法：在正位 X 片上，在胫骨远端划一直线，在距骨上端划一直线，两条直线相交于内侧形成一夹角，称为距骨斜角，此角度正常为 0~5°。

2.6 数据统计

后期对所有数据进行统计学处理，与北京中医药大学统计学专家一起对研究结果的全过程进行分析和总结。以期保证临床研究和统计学研究的一致性。所有数据的统计学处理均在 SAS 17 软件包上进行。其统计方法选择：计数资料用非参数检验；计量资料运用 t 检验或方差分析；临床疗效用 Ridit 分析或 CPD 分析。

3 结果

3.1 一般情况

经过筛选符合纳入标准的病例 110 例，其中望京医院东直门门诊 18 例（孙

中国知网 https://www.cnki.net

老师出诊处），北京中医药大学第三附属医院手足外科门诊 78 例，北京市朝阳区金盏社区 14 例。其中 3 例患者影像资料丢失，2 例患者因运动后再次受伤接受其他治疗，剔除脱落率为 4.5%。随访过程中因电话号码错误失去随访 5 例，患者未坚持功能疗法锻炼 11 例，最终有 89 例资料完整，随访率为 84.8%。其中治疗组 52 例，对照组 37 例。本组患者中男 27 例、女 62 例。治疗组男 13 例、女 39 例，对照组男 14 例、女 23 例。所有患者踝关节扭伤全部为单侧，且都为内翻损伤，均有不同程度的外踝处疼痛，右侧 66 例，左侧 23 例。扭伤到治疗时间最短者 3 周，最长者 11 月，平均 4.7 月。患者年龄最小者 18 岁，最大者 62 岁。随访时间最短者 3 月，最长者 2 年，平均 13.6 月。两组患者在在年龄、性别、疗程、患侧、病史等基线资料方面，经统计学检验，差异无统计学意义（P>0.05），具有可比性（见表 1-5）。

表 1　两组患者一般资料（$\bar{x}$ ±s）

组别	N	年龄（岁）	疗程（月）
治疗组	52	36.41±9.84	4.89±1.36
对照组	37	37.13±11.38	4.35±1.24

3.1.1 两组患者性别分布比较

两组患者中治疗组男 13 例、女 39 例，对照组男 14 例、女 23 例。经 X2 检验，X2 等于 1.686，p 值为：0.244。两组患者性别构成无显著性差异。但是该病在临床观察中女性多于男性。

表 2 两组患者性别比较

组别	N	男	女	X2	P
治疗组	52	13	39	1.686	0.244
对照组	37	14	23		

3.1.2 两组患者受伤肢体分布

两组患者中受伤踝关节右侧多于左侧，具体如下：

表 3 两组患者患侧比较

组别	N	左侧	右侧	X2	P
治疗组	52	13	39	0.046	0.830
对照组	37	10	27		

中国知网 https://www.cnki.net

3.1.3 两组患者年龄分布比较

治疗组患者小于 20 岁 3 人，20-30 岁 7 人，31-40 岁 12 人，41-50 岁 21 人，大于 60 岁 3 人；对照组患者小于 20 岁 2 人，20-30 岁 4 人，31-40 岁 6 人，41-50 岁 18 人，大于 60 岁 3 人；两组患者年龄分布情况经统计学比较，差异无统计学意义（P>0.05）（见表 4，图 6）。

表 4 两组患者年龄分布比较（岁）

组别	N	<20	20-30	31-40	41-50	51-60	>60	Z	P
治疗组	52	3	7	12	21	6	3	-0.731	0.465
对照组	37	2	4	6	18	4	3		

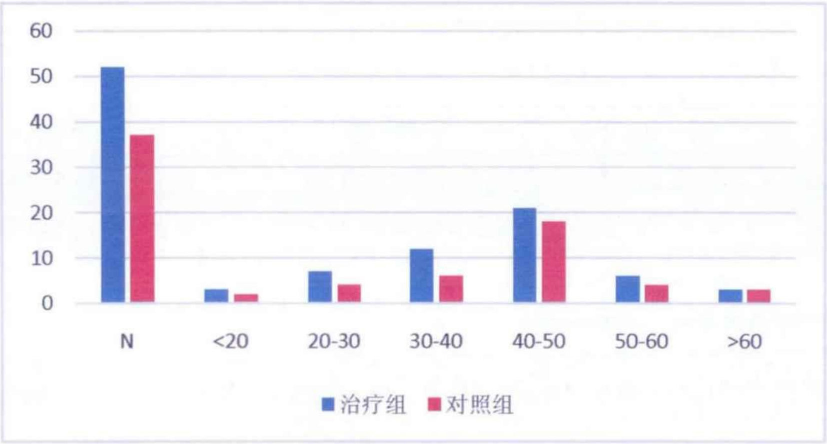

图 6 两组患者年龄分布情况

3.1.4 两组患者病史分布比较

治疗组患者病史 3-8 周 6 人，9-12 周 12 人，13-24 周 27 人，大于 24 周 7 人；对照组患者 3-8 周 7 人，9-12 周 10 人，13-24 周 14 人，大于 24 周 6 人；两组患者病史分布情况经统计学比较，差异无统计学意义（P>0.05）（见表 5，图 7）。

表 5 两组患者病史分布比较（周）

组别	N	3-8	9-12	13-24	＞24	Z	P
治疗组	52	6	12	27	7	-0.789	0.430
对照组	37	7	10	14	6		

中国知网 https://www.cnki.net

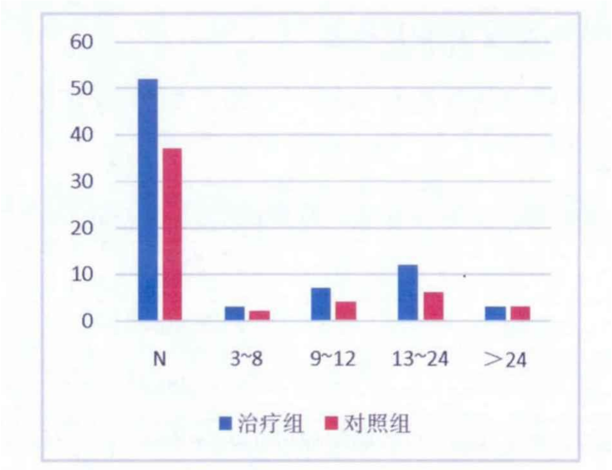

图 7 两组患者病史分布比较（周）

3.2 两组患者疼痛评分情况比较

组内比较：两组患者治疗后与末次统计疼痛评分均低于治疗前，差异有统计学意义（P<0.05，P＜0.01）；两组患者末次统计疼痛评分与治疗后相比，治疗组呈升高趋势，对照组呈降低趋势，差异有统计学意义（P<0.05）。组间比较：两组患者治疗前疼痛评分差异无统计学意义（P＞0.05）；治疗后与末次统计，治疗组疼痛评分低于对照组，差异有统计学意义（P<0.05，P＜0.01）（见表 6）。

表 6 两组患者疼痛评分情况比较（$\bar{x}$ ±s）

组别	N	治疗前	治疗后	末次
治疗组	52	7.24±3.12	2.1±1.68△△●●	3.44±0.96△△▲●
对照组	37	7.16±2.93	6.93±1.02△	5.29±1.34△△▲

注：与治疗前比较，△P<0.05，△△P<0.01；与治疗后比较▲P<0.05，▲▲P<0.01；与对照组比较，●P<0.05，●●P<0.01。

3.3 治疗前后 AOFAS 评分情况

两组患者治疗后 AOFAS 评分情况相比治疗前呈升高趋势，差异有统计学意义（P<0.05，P＜0.01）。治疗组治疗前 AOFAS 评分情况与对照组差异无统计学意义（P＞0.05）；治疗后治疗组 AOFAS 评分情况高于对照组，差异有统计学意义（P＜0.05）（见表 7）。

表 7 两组患者治疗前后 AOFAS 评分情况比较（$\bar{x}$ ±s）

组别	N	治疗前	治疗后
治疗组	52	67.21±3.05	92.3±2.98△△●
对照组	37	68.43±3.01	78.49±2.40△

注：与治疗前比较，△P<0.05，△△P<0.01；与对照组比较，●P<0.05，●●P<0.01。

中国知网 https://www.cnki.net

3.4 患者治疗前后应力位 X 片测量情况

两组患者治疗前后，治疗组与对照组在距骨倾斜度情况方面，差异均无统计学意义（P＞0.05）。

表 8 两组患者距骨倾斜度情况比较

组别	N	治疗前	治疗后
治疗组	52	7.22±0.23	6.85±0.36
对照组	37	7.41±0.31	6.76±0.61

注：与治疗前比较，P＞0.05；与对照组比较，P＞0.05。

3.5 严重不良事件分析

在本研究中严重不良事件定义为：①导致死亡；②威胁生命；③导致踝关节骨折或韧带断裂；④患者出现严重功能障碍，严重影响工作生活者；⑤发生医学伦理事件或引发医疗纠纷；发生以上情况之一。

本研究观察窗期间与随访期间，入组的 89 例患者以及脱落剔除的 21 例患者均未出现严重不良事件。

4 讨论

4.1 踝关节扭伤产生的机制

在临床上踝关节最易发生扭伤，之所以发生扭伤是与踝关节的解剖机构和生理功能息息相关。在人的日常行走或跑跳生理活动中，踝关节负责下肢与地面的接触。由于地面的不平整性，踝关节发生扭伤在所难免。但是并不是每一次的踝关节扭伤都会产生疾病发生。之所以如此是因为人体的距下关节帮助踝关节分担部分扭力，更重要的是踝关节周围有韧带包绕，韧带对踝关节起到了重要的保护作用，特别是内外侧的副韧带[11]。但当扭伤力足够大或应力过于集中于某一处时，则发生踝关节扭伤。一旦发病踝关节周围的韧带首当其冲。在踝关节各组韧带中，以外侧副韧带损伤最为常见。主要是因为踝关节外踝略成三角形，较内踝低，并且与内踝不在同一冠状面上。所以在日常生活中内翻损伤远远多于外翻损伤，而且外侧副韧带较内侧薄弱且足内翻肌群之肌力较外翻肌群强大，当快速行走时，足若来不及协调位置，容易造成内翻跖屈位着地，足受到内翻应力，使外侧副韧带受到牵拉直至损伤。在本组患者中全部为内翻损伤，女性多于男性，这可能与性别差异导致的肌肉韧带的类型不同有一定关

中国知网 https://www.cnki.net

系[12]。在临床上由于暴力大小不同,其损伤程度也不同，韧带可发生部分断裂，完全断裂；韧带完全断裂合并胫距关节脱位；及距跟关节外翻韧带断裂合并跗间关节脱位。当踝关节跖屈位受到内翻应力时，距腓前韧带最为紧张，故首先发生距腓前韧带损伤[13]。

踝关节韧带损伤可以由外伤引起，也可以是在持续运动过程中的过度疲劳所致[14]。踝关节韧带损伤后表现为踝部发红、灼热、肿胀、疼痛等，临床检查踝关节处压痛，并伴有皮肤瘀斑、活动受限等，在急性踝关节损伤期这些症状明显。但是急性踝关节损伤多不能得到有效的治疗而转为陈旧性踝关节损伤。据报道陈旧性踝关节韧带损伤多由急性踝关节扭伤疾病失治、误治或积劳成疾所致[15]。相关文献显示高达 20%~40%的踝关节扭伤将演变成慢性踝关节不稳定[16、17]。陈旧性踝关节扭伤虽然在临床症状上只表现为轻度疼痛和踝关节活动障碍，但是若急性踝关节扭伤不能得到及时和正确的诊治，会导致局部损伤的组织愈合不良，如关节囊、副韧带在松弛的位置愈合会导致踝关节不稳。患者会感觉在一般的工作强度下踝关节出现疼痛、肿胀，当行走在崎岖道路甚至在一般的平地时，会感到踝关节不稳而出现反复的“崴脚”。长期反复的此情况出现，患者会对踝关节有不信任感，不愿意在不平的道路上行走，久而久之会继发粘连性关节囊炎和创伤性骨关节病，以致长期性或永久性踝关节功能障碍[18]。

临床上一般踝关节不稳可以分为机械性不稳定和功能性不稳定，而机械性不稳定又可以分为骨性不稳定和软组织性不稳定。机械性不稳定是指由于各种原因造成的关节活动范围超出了正常生理极限的疾患。由于该病多伴有关节或骨骼的发育异常问题，在此不做特殊讨论。功能性不稳定是指由于各种原因导致关节活动不一定超出正常生理极限，但却完全或不完全失去自主控制的疾患[19]。有学者研究表明[20]，在解剖学和病理学方面看，功能性不稳定的踝关节和正常关节没有区别。有的学者认为踝关节囊或者韧带上存在机械感受器，该病就是机械感受器缺乏或损伤所致[21]。在我们的病例观察中发现所有陈旧性踝关节扭伤患者都有不平道路恐惧感和踝关节不信任病史存在，实验组的患者中通过手法治疗后患者的恐惧感都有不同程度的消除，AOFAS 评分较高这也是其中原因之一。有人已经在膝关节交叉韧带上面寻找到了机械感受器，在踝关节韧带上是否存在机械感受器还需进一步研究。

中国知网 https://www.cnki.net

4.2 中医手法治疗踝关节扭伤的优势与不足

中医骨伤科认为踝关节扭伤新鲜损伤属于"骨错缝"、"筋出槽"的范畴，而陈旧性损伤的病理机制为损伤日久、瘀血内阻、气血失和、腠理不密，导致瘀阻筋脉、肌肉等处，气血不通，不通则痛等。临床上无论新鲜损伤还是陈旧性损伤中医治疗可分为以下几类：①单纯中医手法治疗[22]；②中医手法配合外用中药治疗[23]；③中医手法配合针灸、理疗等治疗[24]；④中医综合方法治疗等[25]。在这些治疗方法中手法治疗是主要手段，也是中医骨伤科的特色之一。手法治疗的作用是活血化瘀、消肿止痛、舒利关节、整复错位、归顺经络等。手法种类有挤压类手法、按揉类手法、活动关节类手法、弹拨类手法、拔伸类手法等等。在这些手法中施术者认为按、揉可以调和气血、促进损伤处血肿炎症物质吸收，而弹拨类手法能使筋松结散、筋解脉通、使局部粘连的组织得以松解，拔伸牵张类手法可以扩大关节间隙，恢复踝关节的正常解剖关系，修复僵硬的踝关节功能。但是，临床医生为了增加临床疗效，大都会在手法的基础上结合外用中药或加用针灸、理疗等治疗。其目的是通过中药外敷可起到活血化瘀、消肿止痛的作用，可以促进血液循环，对局部水肿的吸收有利，不仅止痛迅速，还有利于局部组织的修复。

虽然手法与中药外洗、理疗、针灸等多种方法共用，医生的目的是多种方法协同作用共同提高临床疗效，但是也从另一个方面反映出施术者对手法的不自信。当前中医的临床手法治疗大都是根据医生的不同理念甚至是好恶而采取不同的方法治疗，而且大都是模糊概念，只管追求患者的所谓临床疗效，至于何种方法起到了何种疗效？其究竟对机体的何种组织起到的何疗效大都没有细化和规范化研究。

孙树椿教授提出对于踝关节扭伤无论新鲜损伤还是陈旧性损伤，只要你对手法的要点和施术部位掌握得当，单纯运用中医手法治疗即可取得非常好的临床疗效，完全无需配合其他治疗。我们通过该组病例的观察发现运用孙氏手法治疗踝关节陈旧性损伤疗效明确。其能够明显缓解踝关节局部的疼痛和肿胀，可以明显的改善踝关节功能评分（$p<0.05$）。但是其只适合于功能不稳的踝关节陈旧性扭伤患者，其可以改善患者的自我本体感觉和对路面的恐惧感，并且相对功能疗法锻炼组起效更快，患者也更愿意接受。通过我们的观察还发现无论

中国知网 https://www.cnki.net

手法治疗组还是功能锻炼治疗组对踝穴的胫距关节角虽然治疗前后有所改善，但没有统计学显著性差异。也就是说这两种治疗方法对踝关节机械性不稳定无明显治疗效果。

4.3 孙氏外踝理筋手法的学术特色

孙树椿教授悬壶行医60余年，运用手法治疗疾病是孙老师的一大特色。其认为手法开始的关键就是“手摸心会”，只有这样才能有的放矢，否则眉毛胡子一把抓，诊病不闻病之所在，胡乱从之，临床上是不可能真正治好疾病的。正如《医宗金鉴·正骨心法要旨》所云：“夫手法者，谓以两手安置所伤之筋骨，使乃复于旧也。但伤有重轻，而手法各有所宜。其痊可之迟速，及遗留残疾与否，皆关乎手法之所施得宜，或失其宜，或未尽其法也。盖一身之骨体，既非一致，而十二经筋之罗列序属，又各不同，故必素知其体相，识其部位，一旦临证，机触于外，巧生于内，手随心转，法从手出”。古代由于解剖学知识缺乏，为了解人体构造和病变部位，只能靠手来寻找，做到心中了然。当今现代医学的发展，解剖学知识的丰富，为“手摸心会”提供了可靠详实的基础。孙老师根据自己有10多年手术经历的特点，将中医骨伤科的“手摸心会”与现代解剖学相印证，提出手法治疗要找到病变部位，这和临床上查体不完全一致。病变部位一定有“筋结”的出现。“筋结”是筋伤以后，由于局部的出血、渗血、水肿、炎性细胞侵入，形成的“无菌性炎症”，随着组织的逐渐修复，纤维组织增生而形成的。不同的组织损伤，形成的痛性硬结，形状不一，但呈现规律性。在长期临床实践中，探索总结出了各种疾病筋结的位置和形状[26]。

对于踝关节陈旧性损伤的治疗孙老师主张临床查体一定要和解剖知识相结合。踝关节陈旧性损伤最容易伤及的就是踝关节距腓前韧带，但是临床往往又不单纯是该韧带的损伤，踝关节一旦损伤外侧所有副韧带大都不能幸免，所以临床检查时一般在距腓前韧带周围可以触及豆粒样大小的“筋结”，在按压这些“筋结”时患者会感到剧烈的疼痛。但是这往往就是治疗该病的关键点所在。这些“筋结”需要施术者查体时要慢慢体会，也即是“手摸心会”。在本组病例的观察中无论是手法组还是功能锻炼疗法组在外踝的前方，距腓前韧带的周围均可触及大小不等的“筋结”，按压触碰这些“筋结”部位时患者的疼痛VAS评分平均提高2个等级。但对这些相应部位施以相应手法治疗后，患者的疼痛情况明显改善，

中国知网 https://www.cnki.net

AOFAS评分得到明显提高。

孙老还主张临床治病光有"手摸心会"是远远不够的，治病手法也要讲究"轻、巧、柔、和"。轻：主要指动作要轻，不用暴力手法同样能达到治疗的目的，使患者在心理上易于接受。巧：巧妙，一方面是指手法运用的技巧，另一方面是指用“巧劲”。柔：是手法用力要柔和，不能粗暴、生硬，强调刚中有柔，柔中有刚，刚柔相济。手法的力量要根据患者病情，并结合医生自身功力运用。对新伤用力要轻，动作要缓，而陈旧伤则可逐步加重用力。对于体质较弱、病情较重的患者治疗时要徐徐用力，以能耐受为限。对于身体强壮、病情较轻的患者，用力时使患者感到患处有沉重感或酸痛，但能忍受即可。和：就是心、手相合。医者用手“体会”病患损伤的情况，取得对疾病的正确诊断是治疗的基础，用“心”指导双手施术是治疗的目的。筋伤手法不是简单重复的机械运动，而是在“心”的指导下做的一种能量的输出。老师最欣赏的就是《医宗金鉴·正骨心法要旨》中的：“法之所施，使患者不知其苦，方称为手法也……,盖正骨者，须心明手巧，既知其病情，复善用夫手法，然后治自多效。诚以手本血肉之体，其宛转运用之妙，可以一己之卷舒，高下疾徐，轻重开合，能达病者之血气凝滞，皮肉肿痛，筋骨挛折，与情志之苦欲也。较之以器具从事于拘制者，相去甚远矣”。手法特别讲究力的运用，包含以下几个层面：第一是施术者要有功力、穿透力，具有聚全身气力于一点的威力，作用力要能深达患者的经穴、脉络、筋骨；注重以内心感觉引导动作，以丹田配合力的蓄发，施手法时虽出于手，实发以心，心手相合，从而提高以气催力的能力，做到手法稳健，柔中带刚，手到而病除。正所谓“机触于外，巧生于内，手随心转，发从手出”。第二是用力要轻巧，掌握好发力时机；在施治患者时，患者若有紧张对抗时，则应用一些技巧，转移其注意力，如指导患者行吐纳呼吸调整气息，放松情绪，当患者到吸气时，骤然发力。

在踝关节扭伤的手法治疗中，孙氏手法不主张运用按压、滚揉等刺激局部软组织的手法，而主张在轻揉"筋结"的同时运用特色的"摇、拔、戳"手法进行治疗。“摇”法是指术者在助手的对抗牵引下，双手握住关节远端顺时针摇动关节的手法；“拔”法即牵引、拔伸的意思；“戳”法是宫廷正骨的独特手法，为术者握住肢体远端向关节内戳按的手法。摇法可以使因外伤导致的踝关节周围痉挛的软组织得到放松，从而使踝关节解除“交锁”；拔法通过拔伸、牵引局部痉

中国知网 https://www.cnki.net

挛的软组织，使迂曲、挛缩的毛细血管运行畅通，从而改善了局部的血液循环；戳法充分利用踝穴的作用，使发生轻微移位的踝关节复位，并能使卡压于关节内的滑膜等组织解除嵌顿。并且孙老师还主张好的理筋手法不需要施术很长时间。在本研究的这些病例中手法操作 5~7 次中病即止，每次手法操作时间不超过 3 分钟，所有患者手法后感觉踝关节温热舒适，患者没有任何痛苦，满意率极高。

此外，孙老师在强调手法治疗的重要性的同时，绝不排斥西医手术治疗。选择何种治疗方法不应该分西医、中医，而是根据患者的临床需要而定。对于踝关节陈旧性损伤踝关节存在机械性不稳定者或经检查证实韧带有撕裂者孙老师同样也主张行手术治疗。

4.4 展望

人体是一个有机整体，人体的各个器官和肌肉组织都各司其职，但也各有自己的解剖特点[27]。踝关节是临床重要的负重关节，当前踝关节发生损伤后，医生由于不能明确认识踝关节的解剖结构和受伤机制[28]，导致做出错误的诊断，以至于错过最佳的治疗时机，最终发展成陈旧性踝关节损伤，甚至是踝关节不稳定。另一方面，患者对第一次的踝关节扭伤也往往得不到重视，只有在踝关节转成陈旧性损伤出现临床症状后才引起关注。因此，如何对患者甚至是非足踝专业医生进行这方面的专业教育是今后的关键之所在。这也正是中医“治未病”思想的体现。中医手法治疗该病有自己独特的优势，但是临床评价标准五花八门，不能与国际接轨，疗效往往得不到国际上的承认。因此，如何规范的研究验证中医手法的临床疗效是中医科研的必经之路。

我们的此项研究即是在这方面的一个初步探讨，试图在如何科学传承名老中医临床经验方面进行一个有意义的尝试，有许多不足之处，还有许多问题值得我们今后做进一步深入研究。

参考文献

[1]王正义.足踝外科学[M].北京：人民卫生出版社，2006，205-210.

[2]王安利.运动医学.2007,7

[3]Pijnenburg ACM , VanDijk CN , Bossuyt MM , et al .Treatment of rupture

中国知网 https://www.cnki.net

s of the lateral ankle ligaments :A meta-analysis[J] .J Bone Joint Surg(Am), 2000 , 82 :761～773 .

[4]林志斌.陈旧性踝关节扭伤临床研究进展。亚太传统医药，2014,10（22）：33-34.

[5]谢君，游富贵.温养手法推拿结合中药熏洗治疗陈旧性踝关节扭伤 30 例[J].国医论坛，2013,1（28）：25.

[6]万里，王国新，卞荣.踝关节创伤后手法治疗效果评估[J].中国康复研究，2005,9（43）：126-127.

[7]孙琦、王丹、刘海兵，等. 复合手法结合中药熏洗治疗陈旧性踝关节扭伤疗效观察. 浙江中医杂志，2015,50（4）：281.

[8]高景华、高春雨、孙树椿，等.摇拔戳手法治疗陈旧性踝关节扭伤 34 例.世界中医药，2011,6（3）：214-215.

[9]国家中医药管理局.中医病证诊断疗效标准[M].南京：南京大学出版社，1994, 64-65.

[10]Baird RA.Jackson ST. Fractures of the distal part of the fibula with associated disruption of the deltoid ligament. Treatment without repair of the deltoid Ligamcnt[J]. J Bone Joint Surg Am,1987,69(9):1346-1352.

[11]Maehlum S,Daljord OA. Acute sports injurise in Oslo:a one yearstudy[J].Am J Sports Med. 1984,18(3):181-185.

[12]吴阳，陈世益，蒋佳等.性别差异在运动员前交叉韧带损伤中的影响. 中华创伤骨科杂志，2012，14（5）：447-449.

[13]王亦璁.骨关节与损伤.北京:人民卫生出版社，2007.1498-1514.

[14]潘玮敏，韩一生.运动性踝关节不稳的研究现状[J].中国医师进修杂志，2010, 33（14）：74-76.

[15]林志斌.陈旧性踝关节扭伤临床研究进展。亚太传统医药，2014,10（22）：33-34.

[16]Baumhauer JF,O'Brien T. Surgical Considerations in the Treatment of ankle instability[J]. J Athl Train, 2002,37(4):458-462.

[17]Jackson W, McGarvey W. Update on the treatment of chronic ankle instability and syndesmotic injuries. Ankle and foot[J].Current Opinion in Orthopedics, 2006,17(2):97-102.

中国知网 https://www.cnki.net

[18]李光宪，季永东，郭延章．踝关节损伤后不稳定的手术重建[J]．中国修复重建外科杂志，2003,17（7）：459-460.

[19]Eamonn Delahunt. Neuromuscular contributions to functional instability of the ankle joint. Journal of Bodywork and Movement Therapies. 2007,11(3): 203.

[20]Birmingham TB,Chesworth BM,Hartsell HD,et al. Peak passive resistive torque at maximum inversion range of motion in subjects with recurrent ankle inversionspr-ains.Journal of Orthopaedic and Sports physical Therapy. 1997,25(5): 342.

[21]Ozeki S , Yasumura K .Ligament injuries in the ankle join t[J] .Current Opinion in Orthopedics. 1998,9(3):24 ～ 29.

[22]陈立.推拿治疗陈旧性踝关节扭伤 38 例[J]. 现代中西医结合杂志，2002,18（11）： 1795.

[23]谢君，游富贵.温阳手法推拿结合中药熏洗治疗陈旧性踝关节扭伤 30 例[J]. 国医论坛，2013,1（28）：25.

[24]泮金亮. 针刺配合手法治疗陈旧性踝关节扭伤 30 例. 针灸临床杂志，2007,23（3）： 23-24.

[25]王敏，卢振和，陈来等. 中医药联合臭氧治疗陈旧性踝关节扭伤. 现代中西医结合杂志，2012,21（24）：2669-2670.

[26]高景华，张军. 孙树椿筋伤疾病诊治经验. 北京：中国中医药出版社，2014, 12-13.

[27]杨珍、胡亚哲. 慢性踝关节不稳的诊断与修复. 中国组织工程研究.2014,18（19）：1434-1440.

[28]牛中英.踝关节扭伤致慢性外侧不稳定的原因分析[J].内蒙古中医药，2002,21（5）：42

中国知网 https://www.cnki.net

第四部分 孙树椿教授外踝理筋手法治疗陈旧性踝关节扭伤的机理初探

1.前言

采用中医理筋手法治疗陈旧性踝关节扭伤临床疗效明显，但是相关研究大都从中医学的角度阐述手法作用的机理[1,2]。一般认为踝关节扭伤后局部损伤日久、瘀血内阻、气血失和、腠理不密，导致瘀阻筋脉、肌肉等处，气血不通，不通则痛等。至于何为气血失和？何为瘀阻筋脉？大都是通过临床症状推断所得。即便是中医骨伤科“筋出槽”、“骨错缝”理论也是古代医生对病理机制的一种个人相像和推断，都不能直观的反映出其手法作用机理，由此拿不出令人信服的直观证据，从而得不到国际上的承认。

肌骨超声技术是近几年兴起的一种无创的诊断手段，对于关节的辅助结构和邻近组织，包括韧带、肌肉、肌腱、滑囊、滑膜、神经、软骨等，因为其位置表浅的原因超声探头具有很高的分辨力，可以清楚显示上述组织结构和损伤程度[3]。既然踝关节扭伤主要损伤的是踝关节周围的肌腱等软组织，那么在各种治疗前可以通过肌骨超声技术诊断何部位出现损伤，在治疗后还可以了解治疗方法究竟对损伤部位是否存在疗效。我们正是基于此运用肌骨超声技术试图观察孙树椿教授清宫外踝理筋手法对陈旧性踝关节损伤的确切疗效。

另外，人体是一个有机整体，同样踝关节与周围的韧带、肌肉等软组织也是一个密不可分的整体。外踝关节损伤开始受伤的部位是外踝副韧带，但随着外踝韧带没有得到修复，会逐渐影响全踝关节和影响整个下肢的负重。因此，陈旧性踝关节损伤日久不愈，会影响整个踝关节功能和负重形态发生改变。足底压力测试和步态分析是一项基于生物力学原理，探测人体下肢结构状况，评估及预估未来足部疾病，提供科学康复治疗方法的国际先进技术。由于其方法简单，参数具体客观，科学性强，被广泛的用于骨科临床[4]。步态(Gait)，一般被定义为人或动物在地面上的移动模式[5]。对于人类而言，步态包括爬、走、跑、蹦、跳等，但一般研究中的步态通常所指的仅是行走。本研究中的步态也只是针对行走而言的。我们就是试图通过观察外踝理筋手法治疗前后患者的行走步

中国知网 https://www.cnki.net

态变化情况分析了解手法对全踝关节的影响。具体报告如下：

2 材料与方法

2.1 临床资料

2.1.1 研究对象

自2012年10月---2015年11月北京中医药大学第三附属医院手足外科门诊收治的陈旧性踝关节扭伤患者36例。其中男14例，女22例；所有患者均为单侧，左侧踝关节9例，右侧27 例；患者年龄21—58岁，平均39.7岁；病史最长者25月，最短者6月。

2.1.2 患者纳入标准

①第一诊断必须符合陈旧性踝关节扭伤的患者；

②经肌骨超声影像检查无踝关节韧带完全断裂；

③病史在6个月以上者；

④当患者同时具有其他疾病诊断时，但在治疗期间既不需特殊处理，也不影响第一诊断的临床研究流程实施时，可以入组；

⑤患者适合并接受中医手法门诊或病房治疗；

⑥同意参加本研究，并签署知情同意书。

2.1.3 排除标准

①年龄在16岁以下或65岁以上者；

②合并有心、脑、肝、肾和造血系统等严重危及生命的原发性疾病；

③合并皮肤软组织破损的踝关节扭伤或皮肤病者；

④合并风湿、类风湿、痛风等疾病影响踝关节活动者；

⑤需手术修补的韧带断裂者或合并周围神经损伤者；

⑥合并精神疾病或老年痴呆患者；

⑦局部皮肤对超生耦合剂过敏者。

2.2 实验仪器与材料

2.2.1 肌骨超声实验仪器

超声仪器：采用东芝APLIO790彩色多普勒超声诊断仪，线阵探头，探头频率12MHZ、耦合剂。

中国知网 https://www.cnki.net

2.2.2 步态分析仪器材料

比利时产 Foot-SCAN 步态分析仪、联想笔记本电脑、步态采集卡等（如下图）

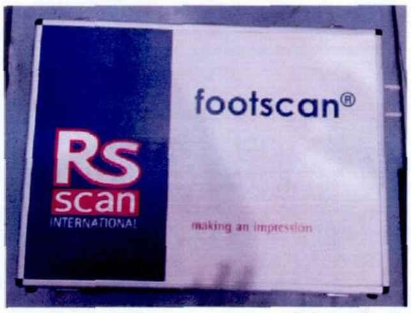

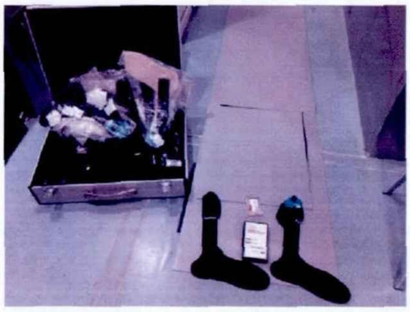

图 1 步态分析仪及测试鞋垫

2.3 实验方法

2.3.1 肌骨超声检查方法

2.3.1.1 超声操作[6]

由同一操作员对陈旧性踝关节扭伤患者于手法治疗前和末次随访时进行如下观察：扫查踝关节的外侧副韧带：距腓前韧带、 跟腓韧带、距腓后韧带。

a 距腓前韧带：侧卧位或平卧位,足部呈跖屈,探头水平置于外踝与距骨间。超声图像中骨性标志有外踝、距骨；距腓前韧带呈倒三角形均匀低回声结构。

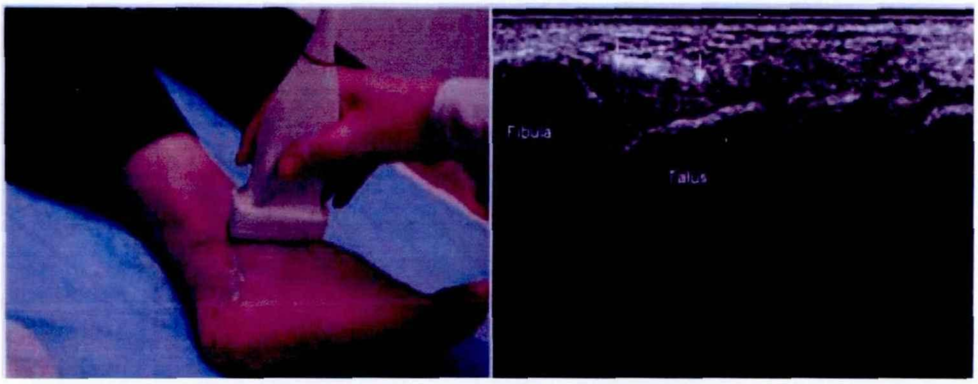

图 2 距腓前韧带 Fibula 外踝 Talus 距骨

b 跟腓韧带：侧卧位,足部呈背屈,探头置于外踝与跟骨间,超声图像骨性标志有外踝、跟骨；跟腓韧带为上细下宽呈中等回声较致密丝状结构,上连外踝,下附着跟骨外侧表面,上段可见与骨组织间有小空隙。前方浅层外踝旁可显示腓骨短肌键、腓骨长肌键的横切面。

中国知网 https://www.cnki.net

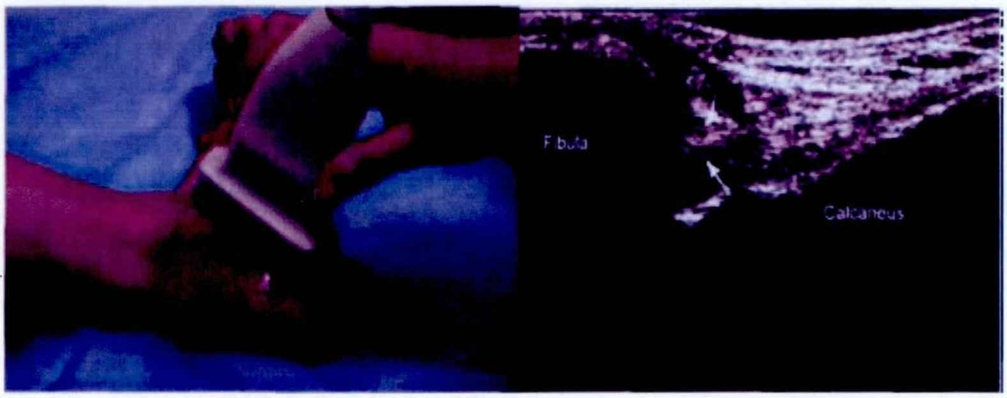

图 3 跟腓韧带 Fibula 外踝 Calcaneus 跟骨

c 距腓后韧带：俯卧位,足呈背屈,探头垂直皮肤置于外踝与距骨后方。超声图像骨性标志有外踝、距骨；距腓后韧带为附着在骨组织间低—中等回声,无明显丝状结构。

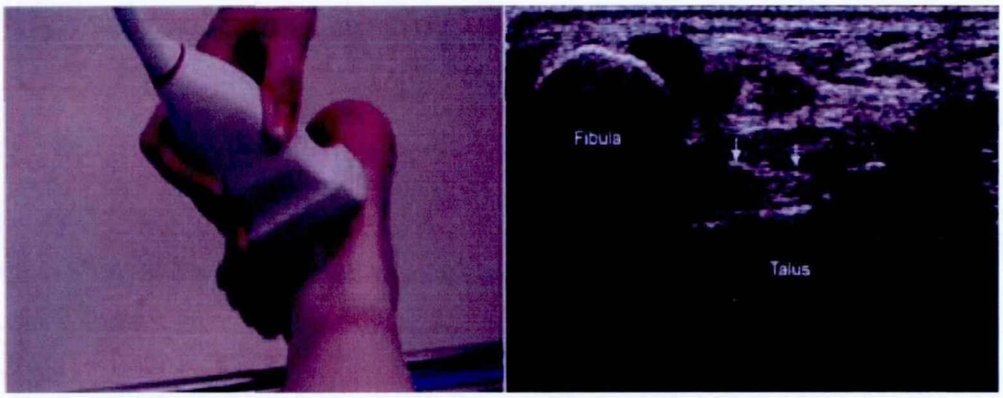

图 4 距腓后韧带 Fibula 外踝 Talus 距骨

2.3.1.2 记录内容

①记录踝关节外侧副韧带的厚度；②观察韧带纤维的连续性；③踝关节运动过程中的韧带张力；④外侧副韧带周围血肿面积；⑤踝关节内积液量等。

韧带厚度（mm）	距腓前韧带	
	跟腓韧带	
韧带的纤维连续性	□ 25% □ 50% □ 75% □ 100%	
韧带的张力	□ 松弛 □ 正常 □ 紧张	
韧带周围血肿（mm^2）	□ 无	
	□ 有	
踝关节内积液量（mm^3）	□ 不可探查	
	□ 可探查	

中国知网 https://www.cnki.net

2.3.2 步态分析测试

2.3.2.1 步态测试方法

先由工作人员向测试者讲解测试要点和方法，并观看演示。然后统一称量测试者体重，并输入电脑，并与测试结果对应，电脑进行自动矫正，进行如下测试：

先对患者进行行走步态训练，嘱患者放松自然行走。然后将步态分析足垫垫于患者的双侧鞋中，然后嘱患者先自行自然试行走，以适应测试环境。待患者行走自然后，开始进行双侧足底应力分布和步态信号采集，集中于采集器内。最后将数据输入电脑，专业软件进行分析。

2.3.2.2 记录内容

踝关节陈旧性损伤后踝关节功能性不稳定患者的步态变化是一个复杂的过程。步态的运动学特征可以从以下参数：步长、跨步长、步速、步频、步宽、步角、步态周期及各时期时相所占总周期的百分比、下肢三大关节在三个面内运动角度及角速度等方面进行反映。每个步态周期又分摆动相、支撑相等。因为时间和经费方面的考虑，更由于该研究的目的是了解孙氏特色外踝理筋手法治疗前后是否对患者的步态改变产生影响，我们没有对有关步态的所有因素进行观察，为了能够直观的说明问题此研究仅观察治疗前后足底重心及应力分布的变化，其中 COGY 代表重心在一个步态周期里 X 轴（前后方向）Y 轴（左右方向）移动幅度,测量治疗前后足底压力重心摆动的距离。

2.4 症状评定标准

本组病例选择均为踝关节外侧副韧带部分断裂或部分松弛的患者。追踪观察 36 例踝关节损伤治疗前及治疗后平均 6.3 个月的情况可见在踝关节各侧副韧带中距腓前韧带是最常损伤、最难恢复的韧带。跟腓韧带、距腓前韧带易同时损伤并损伤程度相仿,但能通过手法保守治疗较快恢复。具体情况：本组患者损伤可分为挫伤和部分断裂，治疗前所有韧带均连续性好；韧带张力表现为松弛；副韧带周围有面积大小不等的血肿；韧带周围有明显的积液。为了能运用超声技术以及统计学处理综合判断手法治疗的疗效性，参照文献[6]对患者症状体征进行分级处理，症状评定标准如下，并使用超声机器检测各韧带厚度。

高频肌骨超声对踝关节侧副韧带损伤症状评定标准：

中国知网 https://www.cnki.net

0 级:韧带肿胀明显，韧带连续性中断，断端挛缩，回声增强，关节腔见大面积积液，内翻实验无回声区间距明显增大。

Ⅰ级:韧带肿胀明显,回声仍不均匀或部分连续性仍中断,关节腔内见积液。

Ⅱ级:韧带稍肿胀,回声欠均匀,连续性好,关节腔内有或无积液。

Ⅲ级:韧带基本无肿胀,回声正常或有点状强回声,连续性好,关节腔内有或无积液。

2.5 统计学分析

采用 SPSS18.0 统计学软件进行分析。计量资料用（$\bar{x}$ ±s）表示，两组间组内比较采用 t 检验，计数资料采用卡方检验，等级计数资料采用非参数检验，非正态数据及方差不齐数据均采用非参数检验，以 P<0.05 表示差异具有统计学意义。

3 结果

3.1 一般情况

本组患者 36 例全部得到随访（为上一研究的部分病例），随访方式均为门诊随访。患者性别、年龄、扭伤等一般情况同临床观察部分。末次随访到受伤时间平均为 6.3 个月。手法治疗次数平均为 7.8 次，每次治疗不超过 10 分钟。

3.2 肌骨超声检查结果

3.2.1 距腓前韧带治疗前后症状评分比较

患者治疗前距腓前韧带症状评分 0 级 5 人，Ⅰ级 19 人，Ⅱ级 8 人，Ⅲ级 4 人，治疗后 0 级 0 人，Ⅰ级 1 人，Ⅱ级 12 人，Ⅲ级 23 人，经统计学检验，治疗前后差异有统计学意义（P＜0.01）（见表 1）。

表 1 距腓前韧带治疗前后症状评分比较

分期	0 级	Ⅰ级	Ⅱ级	Ⅲ级	Z	P
治疗前	5	19	8	4	-5.738	0.000
治疗后	0	1	12	23		

中国知网 https://www.cnki.net

3.2.2 跟腓韧带治疗前后症状评分比较

患者治疗前跟腓韧带症状评分 0 级 7 人，I 级 22 人，II 级 5 人，III级 2 人，治疗后 0 级 0 人， I 级 4 人， II 级 15 人，III级 2 人，经统计学检验，治疗前后差异有统计学意义（P＜0.01）（见表 2）。

表 2 跟腓韧带治疗前后症状评分比较

分期	0 级	I 级	II 级	III级	Z	P
治疗前	7	22	5	2	-5.795	0.000
治疗后	0	4	15	17		

3.2.3 距腓后韧带治疗前后症状评分比较

患者治疗前距腓后韧带症状评分 0 级 5 人， I 级 19 人， II 级 8 人，III级 4 人，治疗后 0 级 0 人， I 级 1 人， II 级 12 人，III级 23 人，经统计学检验，治疗前后差异有统计学意义（P＜0.05）（见表 3）。

表 3 距腓后韧带治疗前后症状评分比较

分期	0 级	I 级	II 级	III级	Z	P
治疗前	4	16	9	8	-3.311	0.001
治疗后	0	6	13	17		

3.2.4 踝关节外侧副韧带厚度治疗前后超声检测比较

患者距腓前韧带、跟腓韧带以及距腓后韧带厚度治疗后均低于治疗前，经统计学检验，差异有统计学意义（P＜0.05, P＜0.01）（见表 4）。

表 4 踝关节外侧副韧带厚度治疗前后超声检测比较（$\bar{x}$ ±s）

韧带	治疗前	治疗后
距腓前韧带	5.69±0.54	3.98±0.55△△
跟腓韧带	3.89±0.28	2.81±0.29△
距腓后韧带	3.92±0.41	2.71±0.42△

注：与治疗前比较，△P<0.05，△△P<0.01。

中国知网 https://www.cnki.net

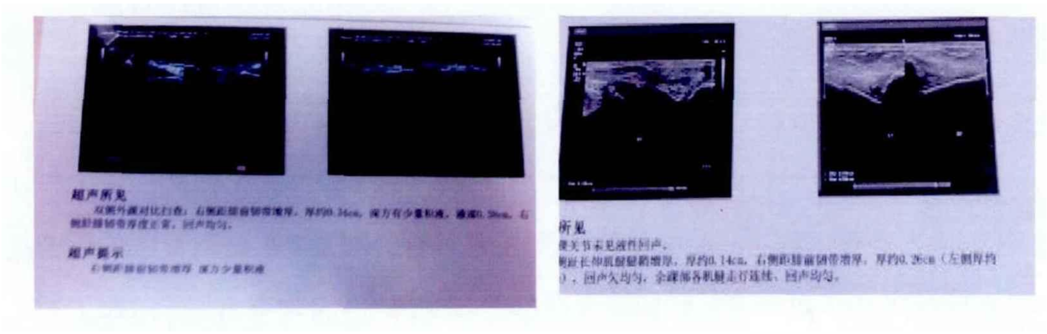

A 治疗前韧带变厚　　B 治疗后厚度接近正常

图 5 超生所见

3.3 足底重心及应力分布的变化比较

本组患者的测试结果显示踝关节陈旧性损伤患者明显对踝关节外侧不信任感，患足足底外侧压力增加，足底压力中心的连线出现明显外移。测量患足较健足压力中心摆动的距离明显变大。经过手法治疗后患者无论足底压力中心还是重心摆动距离均很快恢复到健侧水平。在足底重心及应力分布情况方面，治疗前后的 COGX 、COGY 经统计学检验，差异有统计学意义（P＜0.05, P＜0.01）（见表 5、图 6）。

表 5 足底重心及应力分布的变化比较（$\bar{x}$ ±s）

组别	COGX（mm）	COGY（mm）
治疗前	1499.04 ± 91.00●●	36.18 ± 15.22●
对照后	1599.100 ± 161.19	39.97 ± 18.09

注：治疗前后比较，●P<0.05，●●P<0.01。

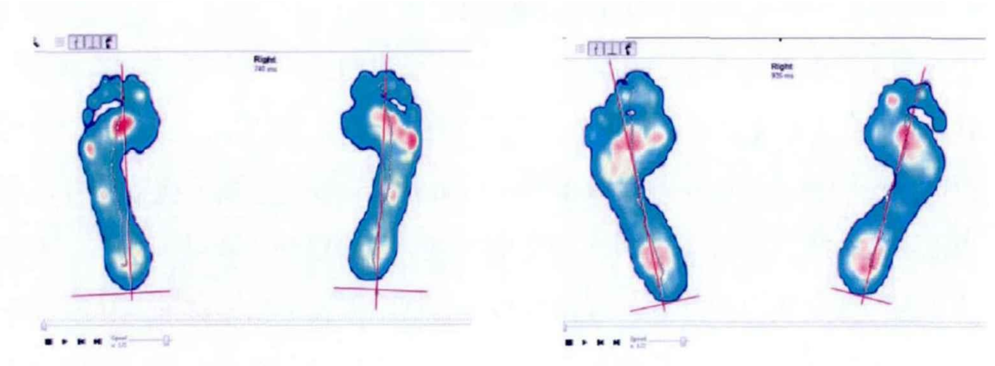

A 右踝关节扭伤治疗前　　B 治疗后两侧重心线基本一致

图 6 踝关节扭伤治疗前后足底应力分布变化图

中国知网 https://www.cnki.net

4 讨论

4.1 运用中医理论解释手法作用机制及其局限性

中医手法是骨伤科医生治疗踝关节扭伤的重要手段之一，临床常用的手法按摩乃取其“理筋整复，活血化瘀”的原则来治疗踝关节扭伤后导致的韧带损伤。大多数学者认为踝关节扭伤的病理机制为损伤日久、瘀血内阻、气血失和、腠理不密，导致瘀阻筋脉、肌肉等处，气血不通，不通则痛。中医手法之所以能够治疗该病，是因为手法能够调和气血、疏通筋脉、松筋散结等功效[7]。也有人试图通过骨伤科“筋出槽”与“骨错缝”的理论来解释该病[8]。踝关节扭伤后使踝关节周围的肌腱、韧带部分撕裂或断裂，脱离了正常的舒缩轨道，而导致筋伤出槽；筋出槽后不能维持原来正常的解剖位置，骨失去筋的牵拉维系处于非生理、不正常吻合状态，表现为骨错缝。相反，关节扭伤使小关节在外力的作用下出现了微细的错缝，关节周围的关节囊、韧带等软组织，也可相应的发生改变，如关节囊的破裂，韧带、筋膜的撕裂等。总之，骨错缝与筋出槽是相互影响的，骨错缝必然导致踝关节周围的筋出槽，而筋出槽的发生踝关节部位也可以引起骨错缝。在治疗时也往往是这样，在纠正了骨错缝之后筋则可自然恢复正常的位置，而使临床症状马上消失。但是这些有关手法作用的阐述大都是基于中医理论的角度，对于没有学习过中医理论的学者很难真正领会，并且其相关作用机制也大多数是操作者的个人相像和推断，都不能直观的反映出其手法作用机理，很难有较强的说服力。

4.2 肌骨超声技术在踝关节扭伤中的应用

踝关节侧副韧带是足部较细小的纤维增厚组织，特别是距腓前韧带。对于软组织损伤，X 线检查无能为力，只能观察关节间隙的变化；关节造影虽可用于韧带损伤的辅助诊断，但其假阳性及假阴性率较高，且属于有创检查，难以作为常规检查推广；CT 及 MRI 多用于关节及软组织肿瘤，在发现病变、显示病变特征及病变分级方面具有不可比拟的优越性，但由于受到踝关节体位和各韧带显示平面的影响，难以得到理想的图像，且不能动态观察，而且费用高昂，不能作为一项常规检查[9]。肌骨超声顾名思义就是指通过高清晰度超声技术观察肌肉、骨骼病理改变的新型检查技术。其有三个方面的优势：①是一种无创检查手段，扫查探头可以按要求放置，不受任何限制，灵活运用；②超生检查可以

中国知网 https://www.cnki.net

获得患者动态信息，获得的信息更接近患者的生理情况；③还可以作为引导、监视手段进行各种治疗操作。

高清晰超声能直接显示肌腱和膝关节韧带等浅表组织的观点已得到肯定，近年来国外学者也陆续对踝关节侧副韧带损伤的超声诊断有所报道[10]。国内林发俭等[11]认为超声是诊断和了解踝关节侧副韧带损伤治疗结果疗效的一种可靠方法，特别是对完全撕裂的诊断和验证具有很高的准确性。其分析 42 例踝关节侧副韧带不同类型损伤的声像图表现，并与临床及手术结果比较,得出 18 例完全撕裂超声诊断与手术结果符合,12 例挫伤、10 例部分撕裂及 2 例完全撕裂。经临床保守治疗前后超声对比观察证实超声检查结果与临床诊断符合率极高。何秀珍等[12]通过研究肌骨超声对踝关节侧副韧带损伤治疗效果观察,认为肌骨超声对踝关节侧副韧带损伤治疗效果评判是一种直接、无创、较廉价的客观影像手段，能较好显示踝关节各侧副韧带、较准确地观察踝关节侧副韧带的损伤情况，诊断其损伤程度，并能作为对踝关节侧副韧带损伤治疗效果观察的较理想的客观影像手段，具有较高的临床应用价值，值得临床广泛开展使用。

我们该研究正是基于高清晰肌骨超声观察骨骼周围软组织方面的优势，观察孙氏特色外踝理筋手法治疗前后的疗效机理进行初探。通过观察我们发现对于踝关节陈旧性损伤患者，超生检查证实虽然踝关节外侧韧带没有断裂，但是损伤导致了踝关节距腓前韧带、跟腓韧带均有不同程度的变宽、变厚，导致韧带水肿从而失去韧带很好的弹性，经手法治疗一段时间后上述韧带逐渐恢复对侧水平，患者的自我疼痛、活动不利等症状也随之减轻或消失。从而反证出我们采用的外踝理筋手法的作用机制是通过恢复踝关节副韧带的功能而起效的。同样也证实了手法仅对副韧带的损伤后功能恢复效果明显，而对于韧带断裂的机械性踝关节不稳定患者治疗意义不大。

4.3 步态变化与陈旧性踝关节损伤

未发生骨折、脱位及韧带断裂的单纯踝关节陈旧性损伤所造成的最大的损害就是后期的踝关节功能性不稳定。踝关节功能性不稳主要与踝关节扭伤时本体感受器损伤及损伤导致的关节周围相关肌群的力量缺失有关，表现为平衡协调能力差，空间位置觉判定能力明显下降[13]。踝关节是一个整体，外侧副韧带的损伤得不到及时的治疗和修复，日久会导致踝关节内侧，甚至全踝关节功能

中国知网 https://www.cnki.net

出现变化。本组病例中许多患者早期是踝关节外侧损伤，踝关节外侧疼痛、肿胀，久之踝关节内侧继而全踝关节周围出现疼痛，影响行走。而随着外踝副韧带周围经过外踝理筋手法治愈后，踝关节内侧、全踝关节症状也逐渐消失。

功能性踝关节不稳所导致的踝关节周围副韧带力量、弹性、柔韧性等方面的改变，继而导致本体感觉能力的下降，可能会导致行走步态的改变。但是影响的程度以及影响步态的哪些方面却无法进行主观判断，必须借助专业的实验设备和分析软件，才能量化功能性踝关节不稳者步态的特征。步态分析是利用力学原理及处理问题的手段，利用已经掌握的人体解剖学及生理学等结构和功能方面的知识，对人体行走时的行为方式及功能状态，进行对比分析，从而提取出与行走有关的生命活动信息的一种整体生物力学的研究方法。它是行走生物力学测量、分析、评价的专门技术[14]。

步态的运动学特征可以从以下参数：步长、跨步长、步速、步频、步宽、步角、步态周期及各时期时相所占总周期的百分比、下肢三大关节在三个面内运动角度及角速度等方面进行反映。而陈旧性踝关节损伤患者步态的运动学特征可能体现在步速、步角、各步态时相所占步态周期的比例、关节角度及关节运动范围、角速度等方面的改变。要想了解和测试该改变过程是一个繁杂而专业性很强的工程。所以，许多学者主张不对步态的所有变量都进行研究，而是对最能代表步态变化的因素加以观察分析。Becker 等[15]对 65 名踝关节陈旧性扭伤后导致踝关节行走不稳的患者和 100 名健康受试者进行行走过程中的足底压力测试，结果显示患足外侧压力明显增加。其提出可以用足外侧压力的增加来鉴别功能性踝关节不稳。钱龙杰[16]对 26 例单侧踝关节扭伤的患者进行步态分析平衡测试，测量患足与正常足压力中心摆动的距离(COF)，研究发现患侧的足底压力中心的摆动距离明显高于正常。 Nawata1 等[17]采用足底压力测试系统对 8 名患有功能性踝关节不稳的运动员进行了行走过程中的足底压力测试，试验要求受试者在长 6m、宽 0.6m 的轨道上进行赤足常速行走，行走过程中要求两足都能踩在预先安置的测力平板上仪。研究结果显示，与对照组相比，功能性踝关节不稳患者的足偏角明显偏小，足底压力中心的连线出现明显外移。受此启发，因为时间和经费方面的考虑，更由于该研究的目的是了解孙氏特色外踝理筋手法治疗前后是否对患者的步态改变产生影响，我们没有对有关步态的所有因素进行观察，为了能够直观的说明问题我们只进行了治疗前后患者足底重心

中国知网 https://www.cnki.net

改变情况的研究。结果发现所有半年以上陈旧性扭伤患者的行走步态都存在变化，足底负重点明显外移。这说明踝关节扭伤后由于踝关节外侧没有得到及时修复，踝关节外侧出现功能性不稳定，身体在行走时为了维持下肢平衡，不自觉会出现重心外移以维持下肢负重的平衡。这也解释了为什么踝关节陈旧性扭伤患者会出现踝关节外侧、小腿、大腿外侧疼痛不适的现象。孙氏清宫外踝理筋手法，虽然称之为外踝理筋手法，其作用的部位其实是全踝关节。按揉“筋结”是通过轻柔手法是受伤挛缩的局部疼痛炎性组织逐渐变软、恢复弹性。特色的“摇”、“拔”、“戳”手法无不作用于全踝关节。“摇”法可以使因外伤导致的踝关节周围痉挛的软组织得到放松，从而使踝关节解除“交锁状态”；“拔”法通过拔伸、牵引局部痉挛的软组织，使迂曲、挛缩的毛细血管运行畅通，从而改善了局部的血液循环；“戳”法充分利用踝穴的作用，使发生轻微移位的踝关节复位，并能使卡压于关节内的滑膜等组织解除嵌顿[18]。通过本组的观察发现，运用上述手法治疗 1-2 个疗程后经足底步态分析，足底重心位移点曲线与对侧接近，治疗前后重心点的恢复有显著性差异。

4.4 结语

踝关节扭伤是临床常见病，虽然是一个看似简单的扭伤，但是如果没有及时处理会给患者带来较大的痛苦。中医手法治疗该病有着操作简单、缓解疼痛快、价格低廉等临床优势。可是其如何起效的机理临床医家却大都不能明确表述清楚。中医手法治疗踝关节扭伤特别是陈旧性扭伤其治疗机理是一个复杂多因素起效的结果，绝不是仅仅利用肌骨超声、步态分析这么简单的两方面因素就能说的清楚地。我们进行上述研究仅仅是一个尝试，试图利用现代技术能够直观、浅显的来说明中医手法治疗该病的机理；试图运用现代技术理论来阐述名老中医临床经验，以便使中医技术能够被越来越多的医生所理解、所接受。尽管我们设计的试验研究还很简单，影响因素也较多，还存在许多不足，只是希望我们能够为如何继承发扬名老中医经验方面大家提供一种思路、一种有意义的探索。

中国知网 https://www.cnki.net

参考文献

[1] 李景元.手法结合针刺及中药外用治疗陈旧性踝关节扭伤 164 例临床观察.中国中医基础医学杂志，2013,19（4）：480-481.

[2] 高景华，张军. 孙树椿筋伤疾病诊治经验. 北京：中国中医药出版社，2014, 12-13.

[3]傅先水，张卫光.肌骨关节系统超生检查规范.第 1 版.北京：人民军医出版社. 2008,1.

[4]Philips D. Biomechanics. In:Vincent ed. Hallux valgus and forefoot surgery. Hetherington: Churchill Livingstone,1994,59:60.

[5]Adolph K.E.， Vereijken B.，Shrout P.E. What changes in infant walking a nd why? [J].child Dev.，2003，74 (2):475-479.

[6]何秀珍，梁峭嵘，石星等.正常成人踝关节侧副韧带超声扫查方法学探讨.中国超声诊断杂志，2006:7(9):679-681.

[7]孙琦、王丹、刘海兵，等. 复合手法结合中药熏洗治疗陈旧性踝关节扭伤疗效观察. 浙江中医杂志，2015,50（4）：281.

[8]高景华、高春雨、孙树椿，等.摇拔戳手法治疗陈旧性踝关节扭伤 34 例.世界中医药，2011,6（3）：214-215.

[9]Cerard Morvan，Jaeque BuSSon，Marc Wybier，etal. Ultrasound of the Ank le.European Journal of Ultrasound，2001,14(l):73-82.

[10]David.P，FeSSe LL，Marni XT，etal.Foot and ankle Sonography.The Radi oglogic Clinies of NA，1999;37(4):831-856.

[11]林发俭，冉维强，黄曼维等.踩关节侧韧带损伤超声检查.中国医学影像技术，2002:18(12):1298 一 1299

[12]何秀珍，梁峭嵘，石星等. 踝关节侧副韧带损伤的超声表现. 实用医学影像杂志，2007，6(8):188-192.

[13]Hertel J. Functional anatomy, pathomechanics and pathophysiology of latera l ankle instability[J]. Journal of athletic Training，2002,37:364-75.

[14]郑秀媛等.现代运动生物力学[M].第 1 版. 北京. 国防工业出版社. 2002：3 98-407.

[15]Becker HP,Rosenbaum D,Claes L, et al. Measurement of plantar pressure d

中国知网 https://www.cnki.net

istribution during gait for diagnosis of functional lateral ankle instability. Abstracts of the Fifth EMED User Meeting 1996.S19

[16] 钱龙杰.步态分析在踝关节功能性不稳定临床治疗的应用[D].上海交通大学硕士论文,2008.

[17]NawataK,Nishihara S,Hayashi I, et al. Plantar pressure distribution during gait in athletes with functional instability of the ankle joint:preliminary report[J]. J Orthop Sci,2005,10:298-301.

[18]陈兆军，孙树椿.清宫正骨手法治疗急性踝关节扭伤的体会.中国中医骨伤科杂志，2015,23（8）：70-71.

中国知网 https://www.cnki.net

小 结

1 概述：

踝关节扭伤临床非常多见。对于不伴有骨折、脱位的单纯踝关节损伤，则往往被患者和临床医生所忽视。从而演变成陈旧性疼痛性踝关节扭伤，有的甚至还会造成踝关节不稳引发踝关节骨性关节炎，给患者造成长期病痛，最后不得不进行踝关节融合或踝关节人工关节置换，从而产生严重的社会负担和经济负担。

踝关节扭伤在中医伤科辨证中属“筋伤”的范畴。中医骨伤科运用一些特色的手法治疗该病有较确切的疗效和优势。我们运用单纯的清宫正骨理筋拔、摇、戳手法治疗了大量急性踝关节扭伤患者，并通过专门的北京市科委的立项课题已经证明其有确切的疗效。而在临床中我们发现和新鲜踝关节扭伤不同的是，在陈旧性踝关节扭伤患者的踝关节周围，特别是外踝处一般可触及较硬的“筋结”，此处往往是患者的疼痛最明显处，采用轻、巧、柔、和的点揉手法对存在的“筋结”进行轻轻的按揉，然后进行踝关节拔、摇、戳外踝理筋手法治疗，患者的疼痛、肿胀症状明显得到缓解。因此我们试着采用该类特色手法治疗陈旧性踝关节扭伤临床收到满意疗效。手法包括：①手摸心会：在外踝处寻找“筋结”。②手法理筋：轻巧点柔“筋结”，待“筋结”变软后以特色的踝关节拔、摇、戳手法理筋。③轻捋收功：最后沿着肌腱韧带走行方向捋顺筋脉。虽然临床疗效明显，但是截至目前，并未有专门针对该类手法治疗陈旧性踝关节扭伤的相关规范研究。

鉴于此，并基于前期临床实践，以及相关研究基础，我们设计了本研究：

①拟采取随机对照的研究方法，设置目前国际通用的陈旧性踝关节扭伤保守治疗方法…踝关节功能康复疗法为对照组，借助踝关节疼痛学 VAS 评分、AOFAS 后足功能评分、应力位 X 片距骨倾斜角等手段评估清宫外踝理筋手法治疗踝关节陈旧性扭伤的临床有效性。

②通过肌骨超声、足底应力测试分析等现代技术手段初步探讨清宫外踝理筋手法治疗陈旧性踝关节扭伤的机理，以期能运用现代化手段更好的总结、继承名老中医经验，为让大家更直观的了解名老中医理筋技术做出有意义的探讨。

中国知网 https://www.cnki.net

2 本研究创新点：

1、首次运用国内、国际通用的观察指标规范验证孙氏清宫外踝理筋手法治疗陈旧性踝关节扭伤的临床疗效。

2、首次采用足底应力分析、局部肌骨超声等现代技术手段阐述清宫理筋手法的作用机理，使中医手法的治病机理可视化、浅显化。

3 本研究不足：

由于时间和自己水平的关系，临床部分只进行了 3 个方面的研究，今后还可以进行更多变量方面的深入探讨；机理研究方面肌骨超声、足底应力分析等技术只进行了一些浅显的观察，许多问题有待于进一步研究。比如：①在肌骨超声下如何分辨“筋结”。②“筋结”部位究竟是什么病理因素。③足底应力及重心位移是多种原因综合所致，踝关节扭伤后受干扰最多的是步态的什么期？对步长、步幅、步宽等因素有何影响等均有待于我们进行专业、细致、深入的研究。

中国知网 https://www.cnki.net

致　谢

唯有不断的总结与回顾才能使自己更加充实，唯有深深的感恩与谢意才能表达我对帮助过我的人的感激。

在三年的跟师岁月中孙树椿老师严肃的科学态度，严谨的治学精神，精益求精的工作作风，深深地感染和激励着我。孙老师不仅在学业上给我以精心指导，同时还在思想、生活上给我以无微不至的关怀，在此谨向孙老师致以诚挚的谢意和崇高的敬意。

恩师精湛的医术、严谨的治学态度、开阔的科研思路，无一不深深的激励着我，使我无论在学习还是在工作上都得到了长足的进步。唯有更加努力上进才能报答恩师对我的栽培之恩！

本人已近半百能够得到此次跟随孙老师学习的机会，非常不易，得此机会与中国中医院科学院望京医院朱立国院长、高景华、高云院长、张清处长、于忱忱老师的热情帮助分不开，在此表示衷心的感谢。三年跟师工作能较圆满的完成与张军、范东、王尚全、金天、叶宜颖、艾辉、于栋、张淳、罗杰、赵国东等师兄弟的帮助也分不开，在此一并致谢。

中国知网 https://www.cnki.net

中国知网 https://www.cnki.net

医结合理念处理骨科常见病、多发病。对手、足外科疾病有丰富的临床经验。最擅长治疗疾病：足内、外翻，平足症，高弓足，足拇内、外翻畸形矫正；足跟痛、胼胝体（脚垫）等各种顽固性疼痛；小儿麻痹后遗症，膝内、外翻畸形矫正等。2、对关节疾患有丰富临床经验，现每年从事膝、踝、距下关节镜技术操作 200 余例，全膝关节置换近 50 例。3、运用清宫手法、小针刀、微创手术等中西医结合方法治疗颈、肩、腰腿痛疾患。

获冶金部科技进步奖 2 项；主编骨科专著 2 部，合作编著骨科专著 6 部；在国内外杂志发表论文 30 余篇。

参与国家级课题 3 项；主持北京市科研课题 2 项；参与省部级科研课题 10 项。

中国知网 https://www.cnki.net

附录：病例观察表

入组序号 □□□	患者姓名拼音缩写<br>□□□□	病例观察表

清宫外踝理缝手法

治疗陈旧性踝关节扭伤的临床观察

（多中心随机对照临床研究）

研究病例报告表

（Case Report Form）

入 组 序 号：　　□　□　□

研 究 组 别：　　A 组□　　B 组□

患者姓名：_______________　　联系电话:_______________

临床观察医生:_____________　　临床研究单位:____________

研究负责人签字:____________

主持研究单位：01 北京中医药大学第三附属医院

02 朝阳区金盏第二社区卫生服务中心

03 骨研所门诊

中国知网 https://www.cnki.net

填写说明

1. 筛选合格者填写正式病例报告表。

2. 病例报告表应用请使用钢笔或签字笔填写，请勿使用圆珠笔或铅笔。

3. 病例报告表填写务必准确、清晰，不得随意涂改，错误之处纠正时需用横线居中划出，并签署修改者姓名及修改时间。举例：李明 2011 02 12。不要用任何方式（橡皮、涂改液等）涂抹原记录。

4. 患者姓名拼音缩写四格需填满，两字姓名填写两字拼音前两个字母；三字姓名填写三字首字母及第三字第二字母；四字姓名填写每一个字的首字母。

5. 各项目口内"肯定"划填"√"，否定划填"×"。如：√。表格中所有栏目均应填写相应的文字或数字，不得留空。如果此项"未做"则填入"ND"，"不知道"则填入"UK"，"不能提供"或"不适用"，则填入"NA"。

6. 临床试验期间应如实填写不良事件记录表。记录不良事件的发生时间、严重程度、持续时间、采取的措施和转归。如有严重不良事件发生，请及时报告课题研究负责单位 北京中医药大学第三附属医院，联系人：陈兆军，联系电话：13701356673，邮箱： zhaojunchen66@126.com.

中国知网 https://www.cnki.net

知情同意书

临床研究项目名称：清宫外踝理筋手法治疗陈旧性踝关节扭伤的临床观察

课题承担单位：北京中医药大学第三附属医院

患者同意声明：

我已了解有关本研究的介绍，而且有机会就此项研究与医生讨论并提出问题，我提出的问题都得到了满意的答复。

我知道参加本研究可能产生的风险和受益。我知晓参加研究是自愿的，我确认已有充足时间对此进行考虑，而且明白：

我可以随时向医生咨询更多的信息。

我可以随时退出本研究，而不会受到歧视或报复，医疗待遇与权益不会受到影响。

我同样清楚，如果我中途退出研究，特别是由于治疗的原因使我退出研究时，我若将病情变化

告诉医生，完成相应的体格检查和理化检查，这将对我本人和整个研究十分有利。

如果因病情变化我需要采取任何其他的治疗，我会在事先征求医生的意见，或在事后如实告诉医生。我同意科技管理部门、伦理委员会或研究者代表查阅我的研究资料。

我同意☐ 我拒绝☐ 除本研究意外的其他研究利用我的医疗记录和检查结果。

我已阅读了患者知情同意书，并得到了医生完整的解释，因此，我自愿参加本研究，我也同意有关方面对照我的原始医疗记录核对收集资料，并愿意按要求与研究者合作完成本研究。

患者签名： 患者联系电话:____________

医生签名： 医生联系电话：

年 月 日

中国知网 https://www.cnki.net

研究单位编号	入组序号	患者姓名拼音字母	就诊日期	
□□	□□□	□□□□	|_|_|_|_|/|_|_|/|_|_| 年 月 日	首次病例入组

一般资料及简要病史

性别：	年龄：	职业：	身高：cm	体重：kg	病程：天
家庭地址：					
病例来源	□门诊 □急诊 □住院		门诊号/住院号：		
病史摘要：					

中国知网 https://www.cnki.net

既往是否治疗过：□无　□有

若有，请说明治疗手段：□药物　□手法　□理疗　□其他

补充说明（药物名称及其他具体实施时间）

病例入选标准　　是　否

标准	是	否
1. 第一诊断必须符合陈旧性踝关节扭伤的患者.	□	□
2. 肌骨超声影像检查无踝关节韧带完全断裂。	□	□
3. 足部无其它畸形。	□	□
4. 年龄16岁以上，65岁以内（不含65岁）。	□	□
5. 当患者同时具有其他疾病诊断时，但在治疗期间既不需特殊处理，也不影响第一诊断的临床路径流程实施时，可以入组。	□	□
6. 患者适合并接受中医门诊治疗.	□	□
7. 同意参加本研究，并签署知情同意书。	□	□

如以上有一项为“否”，此患者不能进入临床研究

病例排除标准　　是　否

标准	是	否
1. 不符合临床研究方案规定的纳入标准者。	□	□
2. 年龄在16岁以下或65岁以上者.	□	□
3. 合并有心、脑、肝、肾和造血系统等严重危及生命的原发性疾病及精神病、老年痴呆、皮肤疾病、皮肤软组织破损的踝关节扭伤等影响治疗的疾病。	□	□
4. 合并风湿、类风湿、痛风等疾病影响踝关节活动者。	□	□
5. 需手术修补的韧带断裂者或合并周围神经损伤者。	□	□
6. 参加其它临床试验者或研究者认为不宜参加本试验者.	□	□
7. 难以对治疗有效性和安全性作出确切评价者。	□	□

以上有一项为“是”，此患者不能进入临床研究。

中国知网 https://www.cnki.net

研究单位编号	入组序号	患者姓名拼音字母	就诊日期	访视一
□□	□□□	□□□□	|_|_|_|_|/|_|_|/|_|_| 年 月 日	入组治疗前

踝部疼痛 VAS 评分

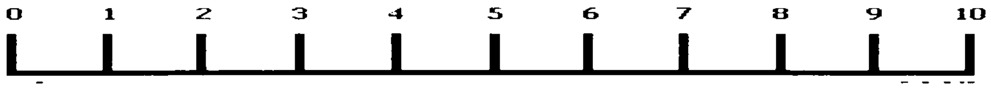

注：疼痛视觉模拟评分法(Visual Analogue Scale, VAS)是将疼痛的程度用 0 至 10 共 11 个数字表示，0 表示无痛，10 表示最痛，病人根据自身疼痛程度在这 11 个数字中挑选一个数字代表其疼痛程度。

	评分标准	记分
踝部疼痛	0 分：无疼痛<br>1-3 分：轻度疼痛，不影响工作、生活<br>4-6 分：中度疼痛，影响工作，不影响生活<br>7-9 分：重度疼痛，影响工作及生活<br>10 分：疼痛剧烈，无法忍受	

肌骨超声影像检查

韧带厚度（mm）	距腓前韧带	
	跟腓韧带	
韧带的纤维连续性	□25% □50% □75%□100%	

中国知网 https://www.cnki.net

韧带的张力	□松弛□正常□紧张	
韧带周围血肿（mm2）	□无	
	□有	
踝关节内积液量（mm3）	□不可探查	
	□ 可探查	

足底应力测试

患足静态足底应力	
患足动态足底应力	

Baird-Jackson 踝关节评分系统

指标	分数	计分
1.踝关节疼痛：共 39 分		
A．无痛	15 分	
B．大运动量活动时轻度疼痛	12 分	
C．日常活动时疼痛	8 分	
D．负重时疼痛	4 分	
E．休息时疼痛	0 分	
2.踝关节稳定性：共 20 分		

中国知网 https://www.cnki.net

A．无临床不稳定	15分	
B．运动时不稳定	5分	
C．日常活动时不稳定	0分	
3.行走能力：共39分		
A．随意行走时无跛行或无痛	15分	
B．随意行走时有轻度跛行或疼痛	12分	
C．行走能力中度受限	8分	
D．仅能走短距离	4分	
E．不能行走	0分	
4.跑步能力：共27分		
A．能无痛随意跑步	10分	
B．随意跑步时轻度疼痛	8分	
C．跑步能力中度受限,轻度疼痛	6分	
D．只能跑短距离	3分	
E．不能跑步	0分	
5.工作能力：共27分		

中国知网 https://www.cnki.net

A．能从事一般职业	10 分	
B．能从事一般职业,但某些强劳动受限	8 分	
C．能从事一般职业,但某些受限	6 分	
D．部分残疾,只能选择工作	3 分	
E．不能工作	0 分	
6.踝关节运动：共 21 分		
A．无损伤踝的 10 度以内	10 分	
B．无损伤踝的 15 度以内	7 分	
C．无损伤踝的 20 度以内	4 分	
D．小于无损伤踝 50%或背曲<5 度	0 分	
7.放射线结果：共 55 分		
A．踝穴正常(内侧和关节上间隙正常,距骨无倾斜)	25 分	
B．关节边缘轻度创伤反应性改变,余同 A	15 分	
C．上关节间隙狭窄测量<2mm,或距骨切线>2mm	10 分	
D．上关节间隙中度狭窄,2～1mm 之间	5 分	
E．上关节间隙严重狭窄<1mm,内侧间隙增宽严重创伤反应	0 分	

中国知网 https://www.cnki.net

能得到的最大分数	100 分	
注明:结果评定标准:优 96～100 分；良:91～95 分；可:81～90 分；差:0～80 分。		

观察医师签名　　日期|_|_|_|_年|_|_月|_|_

研究单位编号	入组序号	患者姓名拼音字母	就诊日期	访视
□□	□□□	□□□□	|_|_|_|_|/|_|_|/|_|_| 年　月　日	末次随访

踝部疼痛 VAS 评分

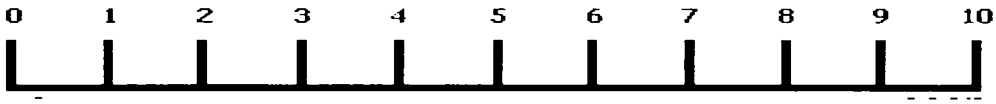

注：疼痛视觉模拟评分法(Visual Analogue Scale, VAS)是将疼痛的程度用 0 至 10 共 11 个数字表示，0 表示无痛，10 表示最痛，病人根据自身疼痛程度在这 11 个数字中挑选一个数字代表其疼痛程度。

	评分标准	记分
踝部疼痛	0 分：无疼痛<br>1-3 分：轻度疼痛，不影响工作、生活<br>4-6 分：中度疼痛，影响工作，不影响生活<br>7-9 分：重度疼痛，影响工作及生活<br>10 分：疼痛剧烈，无法忍受	

中国知网 https://www.cnki.net

肌骨超声影像检查

韧带厚度（mm）	距腓前韧带	
	跟腓韧带	
韧带的纤维连续性	□25% □50% □75%□100%	
韧带的张力	□松弛□正常□紧张	
韧带周围血肿（mm2）	□无	
	□有	
踝关节内积液量（mm3）	□不可探查	
	□ 可探查	

足底应力测试

患足静态足底应力	
患足动态足底应力	

Baird-Jackson 踝关节评分系统

指标	分数	计分
1.踝关节疼痛：共 39 分		
A．无痛	15 分	
B．大运动量活动时轻度疼痛	12 分	

中国知网 https://www.cnki.net

C．日常活动时疼痛	8分
D．负重时疼痛	4分
E．休息时疼痛	0分
2.踝关节稳定性：共20分	
A．无临床不稳定	15分
B．运动时不稳定	5分
C．日常活动时不稳定	0分
3.行走能力：共39分	
A．随意行走时无跛行或无痛	15分
B．随意行走时有轻度跛行或疼痛	12分
C．行走能力中度受限	8分
D．仅能走短距离	4分
E．不能行走	0分
4.跑步能力：共27分	
A．能无痛随意跑步	10分
B．随意跑步时轻度疼痛	8分

中国知网 https://www.cnki.net

C．跑步能力中度受限,轻度疼痛	6 分	
D．只能跑短距离	3 分	
E．不能跑步	0 分	
5.工作能力：共 27 分		
A．能从事一般职业	10 分	
B．能从事一般职业,但某些强劳动受限	8 分	
C．能从事一般职业,但某些受限	6 分	
D．部分残疾,只能选择工作	3 分	
E．不能工作	0 分	
6.踝关节运动：共 21 分		
A．无损伤踝的 10 度以内	10 分	
B．无损伤踝的 15 度以内	7 分	
C．无损伤踝的 20 度以内	4 分	
D．小于无损伤踝 50%或背曲<5 度	0 分	
7.放射线结果：共 55 分		
A．踝穴正常(内侧和关节上间隙正常,距骨无倾斜)	25 分	

中国知网 https://www.cnki.net

B．关节边缘轻度创伤反应性改变,余同 A	15 分	
C．上关节间隙狭窄测量<2mm,或距骨切线>2mm	10 分	
D．上关节间隙中度狭窄,2～1mm 之间	5 分	
E．上关节间隙严重狭窄<1mm,内侧间隙增宽严重创伤反应	0 分	
能得到的最大分数	100 分	
注明:结果评定标准:优 96～100 分；良:91～95 分；可:81～90 分；差:0～80 分。		

观察医师签名 日期|_|_|_|_年|_|_月|_|_日

中国知网 https://www.cnki.net

研究单位编号	入组序号	患者姓名拼音字母	就诊日期	
□□	□□□	□□□□	年 月 日	不良反应事件

不良反应事件

（用标准医学术语）记录所观察到的和用以下问句"自上次访视后，您有何不同的感觉？"直接询问得出的不良事件。尽量使用诊断名称而不使用症状名称。每一栏记录一个不良反应事件。如果在<br>试验期间有不良反应发生，请填写下表。无论有无不良反应事件发生均应在此表下方签名。<br>有无不良事件发生？ 有无			
不良事件名称			
开始发生日期	年月日	年月日	年月日
不良事件严重程度（*）	轻中重	轻中重	轻中重
是否采取措施	是否	是否	是否
处理措施	□继续治疗<br>□停止治疗	□继续治疗<br>□停止治疗	□继续治疗<br>□停止治疗
与研究药物或疗法的关系	□有关<br>□无关<br>□无法判定	□有关<br>□无关<br>□无法判定	□有关<br>□无关<br>□无法判定
根据研究者的判断是否符合严重不良事件定义？<br>1.导致死亡<br>2.威胁生命<br>3.导致住院或延长	是否<br>（如是，请立即电话报北京中医药大学第三附属医院）<br>报告日期：	是否<br>（如是，请立即电话报北京中医药大学第三附属医院）<br>报告日期：	是否<br>（如是，请立即电话报北京中医药大学第三附属医院）<br>报告日期：<br>年月日

中国知网 https://www.cnki.net

住院时间<br>4.导致持续或严重残疾/能力丧失	年月日	年月日	
所发生不良事件的结局	□仍存在<br>□已缓解<br>□不知道<br>缓解日期：<br>年月日	□仍存在<br>□已缓解<br>□不知道<br>缓解日期：<br>年月日	□仍存在<br>□已缓解<br>□不知道<br>缓解日期：<br>年月日
患者是否因此不良事件而退出试验？	是否	是否	是否

观察医师签名 日期|_|_|_|_年|_|_月|_|_日

中国知网 https://www.cnki.net

研究单位编号	入组序号	患者姓名拼音字母	就诊日期	
□□	□□□	□□□□	年 月 日	临床观察结束

完成情况总结

患者治疗时间：天
末次治疗日期：年月日　末次随访日期：年月日
患者研究期间是否发生不良事件：　□无　□有（如果有请填写不良事件表）
患者是否终止退出研究：□否　□是（如果是请填写病例退出表）

病例退出表

病例退出标准	
1.患者未能按时治疗.	☐
2.治疗期间，因并发危重内科疾病、以及其他皮肤疾病等导致治疗时间延长，治疗费用增加者，退出本研究。	☐
3.经治疗后踝关节疼痛加重、经 MRI 证实韧带断裂时，须改复杂手术治疗者，退出本研究.	☐
4.因患者及其家属意愿而影响本研究执行时，退出本研究.	☐
患者治疗时间：天。<br>患者退出日期：年月日。<br>退出原因（选择一项）：☐不良事件，☐违背研究方案，☐缺乏疗效，失访，☐其他（请注明）	

观察医师签名　　　　日期|_|_|_|_年|_|_月|_|_日

中国知网 https://www.cnki.net

病例报告表（CRF）

审核声明

本人作为该试验中心负责人特此声明：经审核，此病例报告表中所有项目的记录都是真实、完整和准确的。

签名：__________

中国知网 https://www.cnki.net

编号：CX2016-043

中医药科研项目查新报告书
（供本院博士论文盲审用）

项　目　名　称：孙树椿教授外踝理筋手法治疗陈旧性踝关节扭伤临床观察及机理初探
委托查新单位：中国中医科学院
通　讯　地　址：
邮　政　编　码：
委托查新日期：2016 年 01 月 07 日

查　新　单　位：国家中医药管理局
中国中医药文献检索中心

完　成　日　期：2016 年 02 月 01 日

中国知网　https://www.cnki.net

一、项目内容及技术要点

项目名称：孙树椿教授外踝理筋手法治疗陈旧性踝关节扭伤临床观察及机理初探

关 键 词：踝关节陈旧性损伤 清宫理筋手法

创新点：

1、运用规范的研究手段观察孙氏外踝理筋手法治疗陈旧性踝关节扭伤临床疗效。

2、试采用足底应力分析、局部肌骨超声影像监测等现代技术手段对“清宫理筋手法”的机理进行初步探讨。

要求查找国内与本项目相关的文献报道，并根据检索结果做出对比性结论。

二、文献检索范围及检索策略

1. 中国中医药期刊文献数据库（1984-2015）：
2. 中国生物医学文献数据库（1978-2016）：
3. CHKD 期刊全文数据库（1994-2016）：
4. 万方数据资源系统：

检索式：

1. 孙树椿
2. 扭伤 AND 踝 AND （理筋 OR 手法）

三、检索结果

根据用户提供的检索词和检索要求，采用上述检索策略，经查《中国中医药期刊文献数据库》、《中国生物医学文献数据库》、CHKD 期刊全文数据库、万方数据资源系统等，检出主要相关期刊文献 3 篇：

主要相关期刊文献：

1. 孙树椿治疗踝关节损伤的临床经验/范东;孙树椿/中央芭蕾舞团医务室,北京市西城区太平街 3 号 100050//世界中医药;2011;6(3):207-208

2. 孙树椿主任手法治疗踝足部筋伤经验撷菁/陈朝晖;唐东昕;王立恒等/安徽中医学院,合肥 230038//中国中医骨伤科杂志;2007;15(8):54

3. 摇拔戳手法治疗陈旧性踝关节扭伤 34 例/高景华,高春雨,孙树椿等/中国中医科学院望京医院脊柱二科//世界中医药,2011,03:214-215.

中国知网 https://www.cnki.net

四、查新结论

综合文献分析：

在查到的国内主要相关文献中，范东、陈朝晖等报道总结孙树椿治疗踝关节损伤的临床经验。

高景华等报道运用宫廷摇拔戳手法治疗陈旧性踝关节扭伤。

综上所述，国内文献中查到总结孙树椿治疗踝关节损伤临床经验的报道；查到运用宫廷摇拔戳手法治疗陈旧性踝关节扭伤的报道。未查到“孙氏外踝理筋手法”治疗陈旧性踝关节扭伤临床及机理研究的报道。

查新人：杨坤杰　技术职称：助理研究员

审核人：赵英凯　技术职称：研究员

国家中医药管理局

中国中医药文献检索中心

2016 年 02 月 04 日

中国知网　https://www.cnki.net
