# Supplementary material for: Efficacy and safety of Tuina (Chinese Therapeutic Massage) for chronic ankle instability: A systematic review and meta-analysis of randomized controlled trials
Source: PLoS One. 2025 Jun 6;20(6):e0321771. doi: 10.1371/journal.pone.0321771 (PMC12143534; doi:10.1371/journal.pone.0321771)
Supplement: S2 File — (ZIP) [file pone.0321771.s004.zip › 8.手法配合康复训练治疗陈旧性踝关节损伤的疗效分析_洪志群.pdf]

DOI:10.16662/j.cnki.1674-0742.2023.01.014

手法配合康复训练治疗陈旧性踝关节损伤的疗效分析

洪志群,谢志敏,沈锋,叶薇
联勤保障部队第九〇〇医院莆田医疗区骨二科,福建莆田 351100

[摘要] **目的** 探究手法配合康复训练治疗陈旧性踝关节损伤疗效。**方法** 随机选取2016年1月—2021年12月联勤保障部队第九〇〇医院莆田医疗区骨二科收治50例陈旧性踝关节损伤患者为研究对象,随机分为A、B组,各25例。A组采用常规康复治疗,B组在A组基础上采用手法配合康复训练治疗,比较两组患者视觉模拟评分(VAS)、关节功能优良率、症状改善、生活质量情况。**结果** 治疗前,两组VAS评分比较,差异无统计学意义(P>0.05)。治疗7、14 d,B组VAS评分低于A组,差异有统计学意义(t=5.298、10.462,P<0.05)。B组踝关节功能优良率(96.00%)高于A组(64.00%),差异有统计学意义(χ^2=8.000,P<0.05)。B组患者踝关节肿胀、压痛、活动受限症状评分低于A组,差异有统计学意义(t=10.506、8.333、7.337,P<0.05)。B组患者心理功能、生理功能、社会功能、躯干水平评分高于A组,差异有统计学意义(t=6.110、7.152、9.834、7.399,P<0.05)。**结论** 陈旧性踝关节损伤患者在疾病治疗过程中以手法按摩联合康复训练治疗,可有效改善疼痛情况,提高踝关节功能水平,改善疾病症状,对患者身体康复有显著作用。

[关键词] 手法配合康复训练;陈旧性踝关节损伤;踝关节功能
[中图分类号] R322 [文献标识码] A [文章编号] 1674-0742(2023)01(a)-0014-05

Analysis of Curative Effect of Manipulative Combined with Rehabilitation Training on Old Ankle Joint Injury

HONG Zhiqun, XIE Zhimin, SHEN Feng, YE Wei
Second Department of Orthopedics, Putian Medical District, 900th Hospital of Joint Logistics Support Force, Putian, Fujian Province, 351100 China

[Abstract] Objective To explore the curative effect of manipulation combined with rehabilitation training in the treatment of old ankle joint injuries. **Methods** Fifty patients with old ankle joint injury admitted to the 2nd Department of Bone, Putian Medical Area, 90th Hospital of Joint Logistic Support Force from January 2016 to December 2021 were randomly selected as the research objects, and randomly divided into group A and Group B, with 25 cases each. Group A received conventional rehabilitation treatment, group B received manipulation combined with rehabilitation training on the basis of group A. Visual Analogue Scale (VAS), excellent and good rate of joint function, symptom improvement and quality of life were compared between the two groups. **Results** Before treatment, there was no statistically significant difference in VAS scores between the two groups (P>0.05). After 7 d and 14 d of treatment, VAS score of group B was lower than that of group A, and the difference was statistically significant (t=5.298, 10.462, P<0.05). The excellent and good rate of ankle joint function in group B (96.00%) was higher than that in group A (64.00%), and the difference was statistically significant (χ^2=8.000, P<0.05). The scores of ankle swelling, tenderness and limited movement in group B were lower than those in group A, and the difference was statistically significant (t=10.506, 8.333, 7.337, P<0.05). The scores of psychological function, physiological function, social function and trunk level in group B were higher than those in group A, and the difference was statistically significant (t=6.110, 7.152, 9.834, 7.399, P<

[作者简介] 洪志群(1979-),男,本科,副主任医师,研究方向为关节和运动医学。
[通信作者] 叶薇(1981-),女,硕士,副主任医师,研究方向为骨与关节病理,E-mail:awei981186@126.com。

0.05). **Conclusion** In the process of disease treatment for patients with old ankle joint injury, manipulative massage therapy combined with rehabilitation training can effectively improve the pain, improve the ankle joint function level, improve the disease symptoms, and have a significant effect on the physical rehabilitation of patients.

[Key words] Manipulation and rehabilitation training; Old ankle joint injury; Ankle joint function

踝关节损伤是日常生活中最为常见的关节损伤性疾病，该病多由间接外力导致，患者在行走过程中踏入凹处使踝关节突然内翻、内收，严重情况下易造成踝关节骨折[1-2]。如果治疗不及时容易导致患者出现反复扭伤，形成陈旧性踝关节损伤，这种情况不仅会增加治疗难度，还会造成严重疼痛，影响踝关节功能。因此加大陈旧性踝关节损伤治疗研究是提升患者生活质量的关键[3-4]。临床中针对陈旧性踝关节损伤多以康复训练为主，通过康复训练可改善踝关节功能、减轻机体疼痛感。相关研究发现在康复训练基础上对患者采用手法治疗，能显著提高疾病治疗效果，对患者机体康复有重要积极意义[5-6]。为进一步观察手法配合康复训练的实施价值，本研究随机选取2016年1月—2021年12月联勤保障部队第九〇〇医院莆田医疗区骨二科收治的50例陈旧性踝关节损伤患者为研究对象，采用分组对比研究，观察手法配合康复训练在陈旧性踝关节损伤疾病治疗中的可实践性，现报道如下。

1 资料与方法

1.1 一般资料

随机选取本院收治的50例陈旧性踝关节损伤患者为研究对象，随机分为A、B组，各25例。A组中男15例，女10例；年龄28~48岁，平均(35.21±2.54)岁；病程5~10个月，平均(7.12±1.33)个月。B组中男14例，女11例；年龄27~49岁，平均(35.30±2.48)岁；病程4~11个月，平均(7.15±1.40)个月。两组一般资料比较，差异无统计学意义(P>0.05)，具有可比性。研究经医院医学伦理委员会批准，且患者家属知情，并在《知情同意书》签字。

1.2 纳入与排除标准

纳入标准：满足踝关节损伤疾病特点；年龄>20岁；可与人正常交流；认知状态良好；同意医学观察。

排除标准：精神性疾病者；口语表达障碍者；合并脏器损伤者；合并肿瘤者；妊娠、哺乳期女性；耐受性低者；研究期间参与其他项目研究者。

1.3 方法

A组采用常规治疗，布洛芬(国药准字H32023726，规格：0.1 g)口服，3次/d，0.4 g/次，同时进行基础康复训练，例如对患者踝关节进行基础按摩，帮助改善踝关节疼痛。

B组在A组基础上采用手法治疗，主要内容：(1)手法治疗。在患者治疗过程中，指导患者保持仰卧位姿势，并且将软枕垫于患者跟腱位置，使患者充分暴露膝盖及下肢部分，在患者踝关节周围组织部分涂抹依托芬那酯凝胶(注册证号HC20160035；规格：10%)，进行局部润滑，完成润滑后按揉踝关节局部，按揉时间控制在5 min，按揉有利于药膏吸收，有助于消除踝关节肿胀。完成按揉操作后以患者踝关节筋络为主，进行推法按揉，达到理筋骨、松骨膜作用，时间控制在5 min，推法完成后为患者进行穴位按摩，选择三阴交、悬钟、昆仑，太溪等穴位进行按摩，穴位按摩的同时开展踝关节牵引治疗。(2)康复训练。训练过程中需要依据患者实际的病情开展康复训练计划，训练内容主要包括3个部分：①训练初期需要指导患者单腿站立训练，每条腿均需要进行训练，1次/d，20 min/次，以此锻炼患者踝关节平衡能力。②当患者踝关节功能平衡训练结束后可以为患者增加康复训练难度，指导患者慢跑练习，慢跑训练1次/d，慢跑距离依据患者实际情况而定，以1 000 m为最佳，有助于提升患者踝关节耐性。③完成基础康复训练后需要指导患者踝关节负重练习，指导患者双手提沙袋，沙袋重量保持10 kg，使患者绷直双腿，以踝关节周围肌肉进行行走跟提，跟提时间控制在60 s，3次/d，跟提动作为20个/次。

1.4 观察指标

比较两组患者视觉模拟量表(Visual Analogue Scale, VAS)评分、关节功能优良率、症状改善、生活质量情况。

①视觉模拟量表(VAS)评分[7]：0~2分为无疼痛，3~5分为轻微疼痛，6~8分为中度疼痛，9~10分

为重度疼痛。

②踝关节功能优良率[8]：采用Kofoed量表进行评定，评定内容主要分为疼痛、功能、活动度3个部分，满分100分，0~59分为差，60~79分为良，80~100分为优。优良率=（优例数+良例数）/总例数×100%。

③症状改善情况：由本院自制调查问卷，问卷内容包括肿胀、压痛、活动受限等症状，各症状评分均为10分，分数越高患者症状越严重，分数越低患者症状越轻。

④生活质量判定[9]：采用健康状况调查问卷（36-Item Short-Form, SF-36）进行判定，观察患者生理功能、心理功能、社会功能、躯干水平。各维度满分100分，分数越好患者生活质量越好，反之分数越低患者生活质量越差。

1.5 统计方法

采用SPSS 22.0统计学软件进行数据处理，符合正态分布的计量资料以（$\bar{x}\pm s$）表示，组间差异比较采用t检验；计数资料以[n(%)]表示，组间差异比较采用χ^2检验，$P<0.05$为差异有统计学意义。

2 结果

2.1 两组患者治疗前后VAS评分比较

治疗前，两组患者VAS评分比较，差异无统计学意义（$P>0.05$）。治疗7、14 d，B组患者VAS评分低于A组，差异有统计学意义（t=5.298、10.462，$P<0.05$），见表1。

表1　两组患者治疗前后VAS评分比较[($\bar{x}\pm s$)，分]

Table 1　Comparison of VAS scores before and after treatment between the two groups of patients[($\bar{x}\pm s$),points]

组别	治疗前	治疗7 d	治疗14 d
A组（n=25）	5.14±1.01	4.58±0.87	4.01±0.64
B组（n=25）	5.12±1.03	3.41±0.68	2.01±0.71
t值	0.069	5.298	10.462
P值	0.945	<0.001	<0.001

2.2 两组患者踝关节功能优良率比较

B组踝关节功能优良率（96.00%）高于A组（64.00%），差异有统计学意义（χ^2=8.000，$P<0.05$），见表2。

2.3 两组患者症状改善情况比较

B组患者踝关节肿胀、压痛、活动受限症状评分均低于A组，差异有统计学意义（t=10.506、8.333、7.337，$P<0.05$），见表3。

表2　两组患者踝关节功能优良率比较[n(%)]

Table 2　Comparison of excellent and good ankle joint function rate between the two groups of patients[n(%)]

组别	例数	优	良	差	优良率
A组	25	6（24.00）	10（40.00）	9（36.00）	16（64.00）
B组	25	10（40.00）	14（56.00）	1（4.00）	24（96.00）
χ^2值					8.000
P值					0.005

表3　两组患者症状改善情况比较[($\bar{x}\pm s$)，分]

Table 3　Comparison of symptom improvement between the two groups of patients[($\bar{x}\pm s$),points]

组别	肿胀	压痛	活动受限
A组（n=25）	7.14±1.25	6.54±1.02	6.94±1.14
B组（n=25）	3.47±1.22	4.04±1.10	4.14±1.53
t值	10.506	8.333	7.337
P值	<0.001	<0.001	<0.001

2.4 两组患者生活质量比较

B组患者心理功能、生理功能、社会功能、躯干水平评分均高于A组，差异有统计学意义（t=6.110、7.152、9.834、7.399，$P<0.05$），见表4。

表4　两组患者生活质量比较[($\bar{x}\pm s$)，分]

Table 4　Comparison of quality of life between the two groups of patients[($\bar{x}\pm s$),points]

组别	心理功能	生理功能	社会功能	躯干水平
A组（n=25）	67.28±4.36	67.19±4.54	67.37±4.28	69.36±4.51
B组（n=25）	75.07±4.65	77.26±5.38	80.51±5.13	81.14±6.56
t值	6.110	7.152	9.834	7.399
P值	<0.001	<0.001	<0.001	<0.001

3 讨论

踝关节损伤是骨科疾病治疗中多发性疾病以及高发性疾病，导致疾病发生的原因有很多，例如走路不规范、日常生活中对踝关节保养不良、年龄过大身体素质较低等原因都可能在日常生活中引发患者踝关节损伤[10-11]。陈旧性踝关节损伤则是由于患者关节损伤后，未得到及时治疗导致踝关节再次出现损伤，严重影响患者关节功能，严重情况下出现踝关节骨折，因此陈旧性踝关节损伤患者需积极进行骨科治疗[12-13]。药物治疗是目前临床踝关节损伤常见的治疗措施，部分患者完全通过药物治疗减少自身关节疼痛。但由于药物治疗过程中患者会对药物产生依赖性，同时药物具有一定的不良反应，对机体的健康产生不良影响，且远期效果不理想，因此需要在药物治疗的同时辅助康复治疗，康复治疗可以在改善患者疾病症状的同时改善踝关节功能，对于患者疾病预后具有显著价值。

手法治疗及康复训练均是目前陈旧性踝关节损伤的主要治疗方法，前者利用手法按摩以及穴位按摩推拿等方法改善患者踝关节功能[14-15]。康复训练则是对患者踝关节进行功能训练，提高踝关节的平衡性以及站立持久性，将两种方法有效结合更加利于患者踝关节功能康复。本研究结果显示，B组患者治疗14 d的VAS评分（2.01±0.71）分低于A组（4.01±0.64）分，踝关节功能优良率高于A组，踝关节肿胀、压痛、活动受限症状评分低于A组（$P<0.05$），分析原因，利用推拿手法以及穴位按压手法，可以通过穴位的按摩有效改善患者肢体血液循环，帮助患者实现机体血气通畅，并达到舒筋活络的效果，改善患者踝关节肿胀情况。而且在手法治疗中利用拉伸可以保证踝关节间隙恢复正常，与此同时结合康复训练，恢复肢体的稳定性及运动协调性，可使患者在日常生活中更加灵活，降低由于关节不协调导致的运动受限，从而提升患者生活质量[16-17]。黄伟等[18]等在报道中针对65例陈旧性踝关节运动损伤患者为例，分别采用康复训练与康复训练联合手法进行治疗，结果显示使用联合治疗组患者VAS评分为（2.19±0.21）分低于单一治疗组的（3.92±0.25）分，且联合治疗患者踝关节功能优良率（93.94%）高于单一治疗组（78.13%）（$P<0.05$），与本研究结论高度相似，而且本研究中对患者症状改善情况进行比较，发现手法治疗联合康复训练可以显著改善陈旧性踝关节损伤症状，证实了陈旧性踝关节功能损伤患者在疾病治疗中使用手法治疗联合康复训练更加有利于自身病情好转。

现代医疗卫生发展过程中疾病治疗方法不仅仅包括药物治疗、手术治疗，同时还可以积极融入康复治疗，通过有效的康复治疗可以有效提高患者的康复速度，防止由于单一治疗过程中对患者的踝关节功能造成不利影响。在康复治疗过程中融合中西医结合更能体现现代化疾病治疗思想，有利于改善病症、提升生活质量。

综上所述，将手法治疗联合康复训练治疗应用于陈旧性踝关节损伤疾病治疗中，既可以改善患者关节症状，还可以降低患者关节疼痛，改善踝关节功能，提高患者生活质量，值得在临床疾病治疗中实践与推广。

[参考文献]

[1] 张佳翔,刘悦.浮针结合再灌注治疗陈旧性踝关节扭伤的疗效观察[J].按摩与康复医学,2020,11(14):10-13.
[2] 舒文韬,欧阳松,罗建平,等.杜氏拔伸摇踝手法配合香独活血散外敷治疗陈旧性踝关节扭伤疗效观察[J].四川中医,2020,38(11):158-160.
[3] 谭伟,孙妍,徐鹏陶.踝关节运动损伤的机制及康复训练治疗效果分析[J].中华养生保健,2020,38(8):25-26,36.
[4] 汤周泉,刘惠宇,鲍晓,等.浮针疗法配合早期康复训练治疗急性踝关节扭伤的临床疗效[J].内蒙古中医药,2021,40(8):106-108.
[5] 罗树雄,薛爱国.推拿手法配合平衡训练治疗陈旧性踝关节扭挫伤临床观察[J].实用中医药杂志,2020,36(12):1634-1635.
[6] 胡浩.陈旧性踝关节扭伤的手法治疗的对比研究[J].体育风尚,2021(7):139-140.
[7] 陈张,王晓东,寇智君,等.理筋手法联合探穴针罐法治疗陈旧性踝关节扭伤的临床研究[J].中医正骨,2021,33(3):20-25.
[8] 张博.正骨理筋手法联合体外冲击波治疗陈旧性踝关节扭伤的临床疗效观察[D].兰州:甘肃中医药大学,2021.
[9] 陈张,王晓东,寇智君,等.推拿手法联合探穴针罐法治疗陈旧性踝关节扭伤1例[J].中国乡村医药,2021,28(14):43.
[10] 金祥雨,陈朝晖,江雨,等.理筋正骨手法联合消瘀接骨散治疗陈旧性踝关节扭伤临床观察[J].安徽中医药大学学报,2021,40(5):14-18.
[11] 陈善创,苏海涛,黄永铨,等.抗阻训练结合舒筋活络洗剂熏洗治疗陈旧性踝关节内翻型扭伤临床疗效观察[J].辽宁中医药大学学报,2020,22(4):196-198.
[12] 刘路平,朱兰然,张卫,等.2种不同手术方法治疗陈旧性踝关节骨折合并下胫腓联合损伤的对比[J].昆明医科大学学报,2021,42(10):106-111.
[13] 陈张,孙佳蕾,王晓东,等.摩伸揉手法联合探穴针罐法治疗陈旧性踝关节扭伤30例[J].中国中医骨伤科
（下转第22页）

心血管事件患者预后不良因素分析[J].内科急危重症杂志,2021,27(2):138-141.

[20] Silversides JA, Emmet M, Ferguson AJ, et al. Conservative fluid management or deresuscitation for patients with sepsis or acute respiratory distress syndrome following the resuscitation phase of critical illness: a systematic review and meta-analysis[J]. Intensive care medicine,2020,43(2):155-170.

[21] 王彦芹.急危重症患者急诊呼吸阶梯性治疗的临床疗效分析[J].中国实用医药,2020,15(8):41-42.

[22] 何俊,许冀.急诊呼吸阶梯性疗法对急危重症患者进行呼吸复苏治疗的价值体会[J].中国社区医师,2020,36(18):17-18.

（收稿日期:2022-10-08）

（上接第17页）

杂志,2021,29(5):59-61,65.

[14] 田帼,刘悦.经筋恢刺法治疗陈旧性踝关节扭伤的临床疗效观察[J].广州中医药大学学报,2021,38(10):2158-2162.

[15] 赵焕东,王勤俭,王燕.筋骨消痛散贴敷配合红外线照射治疗陈旧性踝关节扭伤的疗效观察[J].中医临床研究,2019,11(27):110-112.

[16] 熊峻,陈壮娜,黄石龙,等.齐刺联合电温针疗法治疗陈旧性踝关节扭伤的效果观察[J].当代医药论丛,2020,18(19):134-135.

[17] 苏嘉,沈新升.踝关节骨折手术治疗和手法复位治疗效果分析[J].中国全科医学,2021,24(S1):120-122.

[18] 黄伟,肖婷婷,李雁冰,等.手法联合康复训练在治疗陈旧性踝关节运动损伤中的应用价值[J].世界最新医学信息文摘:连续性电子期刊,2021,21(10):204-205.

（收稿日期:2022-10-09）
