# Supplementary material for: Efficacy and safety of Tuina (Chinese Therapeutic Massage) for chronic ankle instability: A systematic review and meta-analysis of randomized controlled trials
Source: PLoS One. 2025 Jun 6;20(6):e0321771. doi: 10.1371/journal.pone.0321771 (PMC12143534; doi:10.1371/journal.pone.0321771)
Supplement: S2 File — (ZIP) [file pone.0321771.s004.zip › 9.清宫外踝理筋手法结合中药熏...疗陈旧性踝关节扭伤疗效观察_吴俊德.pdf]

清宫外踝理筋手法结合中药熏洗治疗陈旧性踝关节扭伤疗效观察

吴俊德　马占华　潘旭月　祁印泽　陈兆军
（北京中医药大学第三附属医院手足外科，北京 100029）

【摘要】目的　评价清宫外踝理筋手法治疗陈旧性踝关节扭伤的临床疗效及安全性。**方法**　选择2017年1月—2020年6月就诊于北京中医药大学第三附属医院的陈旧性踝关节扭伤患者96例，采用随机数字表法分为观察组、对照组各48例。对照组运用踝关节功能疗法结合中药熏洗治疗，观察组运用清宫外踝理筋手法结合中药熏洗进行治疗，14 d为1个疗程。对比2组疗效及治疗前后疼痛视觉模拟评分（VAS）、Baird-Jackson踝关节评分距骨倾斜角。**结果**　观察组总有效率高于对照组（$P<0.05$）。治疗后、随访时，2组VAS评分均低于治疗前（$P<0.05$），且观察组VAS评分均低于对照组（$P<0.05$）。治疗后、随访时，2组Baird-Jackson踝关节评分均高于治疗前（$P<0.05$），且观察组Baird-Jackson踝关节评分均高于对照组（$P<0.05$）。治疗前后2组组间及组内距骨倾斜角比较，差异均无统计学意义（$P>0.05$）。2组治疗期间及随访期间均未出现施治部位皮肤损伤、肿痛及皮下出血等不良反应。**结论**　清宫外踝理筋手法结合中药熏洗治疗陈旧性踝关节扭伤疗效确切，安全性高，效果优于踝关节功能锻炼疗法结合中药熏洗治疗。

【关键词】陈旧性踝关节扭伤；清宫外踝理筋手法；中药熏洗
DOI：10. 16025/j. 1674-1307. 2022. 12. 027

踝关节扭伤指踝关节在内翻位或外翻位受伤，但未出现踝部骨折、脱位等，属单纯的踝部韧带等软组织损伤，是常见的运动损伤，约占25%[1]。由于踝部损伤不伴骨折、脱位，往往被忽视，在损伤早期因失治、误治等逐渐演变为陈旧性踝关节扭伤[2]，遗留复发性损伤，甚至出现慢性踝关节不稳，最终导致踝骨关节炎的发生[3]。陈旧性踝关节扭伤患者的主要临床表现为在长时间站立或行走时踝关节酸痛、肿胀、无力，在不平的路面行走，患者会有恐惧感或踝关节不稳感以及踝关节活动受限等，影响患者的日常生活。有效缓解患者疼痛及功能障碍是临床治疗中亟需解决的问题，近年来本团队运用清宫外踝理筋手法结合中药熏洗治疗此损伤并观察临床效果，现报告如下。

1　临床资料

1.1　一般资料

选择2017年1月—2020年6月北京中医药大学第三附属医院收治的陈旧性踝关节扭伤患者96例，采用随机数字表法分为2组，各48例。观察组男27例，女21例；年龄18～62岁，平均（34.23±4.25）岁；病程1～30个月，平均（3.51±0.35）个月；踝关节损伤：左侧22例，右侧26例。对照组男25例，女23例；年龄17～64岁，平均（33.67±3.83）岁；病程1.5～27个月，平均（3.78±0.57）个月；踝关节损伤：左侧20例，右侧28例。2组一般资料比较，差异无统计学意义（$P<0.05$），具有可比性。本研究经医院临床研究伦理委员会批准，所有患者均签署知情同意书。

1.2　诊断标准

参照《中医病证诊断疗效标准》[4]制定陈旧性踝关节扭伤诊断标准：①有明确的踝关节扭伤史；②损伤时间>3周；③踝关节酸痛、肿胀、无力，不能长时间站立、行走，可有关节不稳感，影响日常行走和运动；④踝关节内外侧可出现不同程度的肿胀和压痛，在局部可能会触摸到细小“筋结”；⑤查X线片未见踝部骨折、脱位和其他骨质病变。满足4项即可确诊。

1.3　纳入标准

①患者的第一诊断需与以上关于陈旧性踝关

作者简介：吴俊德，男，36岁，硕士，主治医师。研究方向：足踝部骨关节疾病的防治。
通信作者：陈兆军，E-mail：chenzhaojun@sina. com
引用格式：吴俊德，马占华，潘旭月，等. 清宫外踝理筋手法结合中药熏洗治疗陈旧性踝关节扭伤疗效观察[J]. 北京中医药，2022，41(12)：1433-1436.

(C)1994-2023 China Academic Journal Electronic Publishing House. All rights reserved.　http://www.cnki.net

节扭伤的诊断标准相符；②经肌骨超声探查患踝，除外踝部内外侧副韧带的完全撕裂；③患踝内翻应力位X线片上的距骨倾斜角<15°；④年龄16~65岁，足部无其他畸形。

1.4 排除标准

合并心脑血管、肝、肾和造血系统等严重疾病者；合并患踝局部皮肤有破损，或者局部伴有皮肤疾病者；合并骨病而影响踝关节活动者；合并周围神经损伤者；合并精神疾病者；无法按时治疗，依从性差或无法坚持随访者。

2 治疗与观察方法

2.1 治疗方法

2.1.1 中药熏洗：予苏红汤方[5-6]，药物组成：苏木、红花、伸筋草、透骨草、羌活、独活、路路通各30 g，五加皮、煅自然铜、大黄、制乳香、制没药各15 g，牛膝20 g，川乌、草乌各10 g。将药物放于约2 000 mL冷水中浸泡30 min，煮沸，煎取约1 500 mL药液，置于足浴盆中，待温度适宜后，对患踝进行熏蒸（45～60 ℃）并浸泡（38～42 ℃）20 min。早晚各1次，持续14 d为1个疗程。

2.1.2 对照组：踝关节功能疗法[7-9]结合中药熏洗治疗。操作：患者以健侧脚为支撑保持直立；患侧膝关节保持伸直位，如觉身体不稳双手可轻扶墙面。①背伸足踝锻炼胫骨前肌，让患肢足踝背伸至最大程度保持20 s，10次/组，背伸时患肢足趾尽量向上翘起，足后跟用力下压。②跖屈足踝锻炼胫骨后肌、小腿三头肌，使患肢足跖屈至最大程度保持20 s，10次/组，跖屈时患肢足趾尽量下压。③主动内翻足踝以拉伸踝外侧副韧带，锻炼胫骨后肌、小腿三头肌，在患踝内翻至最大程度保持20 s，10次/组，锻炼时注意将着力点放在足外侧缘，向踝外侧发力压低踝部，控制在疼痛可耐受范围内。④主动外翻足踝以拉伸踝内侧副韧带，锻炼腓骨长短肌、胫骨前肌，在患踝外翻至最大程度保持20 s，10次/组，锻炼时注意将着力点放在足内侧缘，向踝内侧发力压低踝部，控制在疼痛可耐受范围内。⑤提踵练习锻炼小腿三头肌肌群，身体站直，双脚并拢，重心适当前移，以脚尖为着力点，缓缓提起双足跟，头上顶，稍停，双足跟下落，轻震地面，一起一落为1次，10次/组，注意以双手维持身体平衡，以防摔倒。以上整套踝关节功能疗法锻炼完成为1遍，每组动作间休息5 s，嘱患者每日早晚各锻炼1遍，且每周均由专人监督指导，连续锻炼14 d为1个疗程。

2.1.3 观察组：清宫外踝理筋手法[10]结合中药熏洗治疗。操作：患者向健侧侧卧，患肢在上并伸出床外，助手双手握住患侧小腿中下段，勿使晃动，并便于与施术者对抗用力。具体步骤：①手摸心会，仔细寻找患踝的痛点及“筋结”。②手法理筋：用指腹适度按揉痛点或“筋结”，力量取刚刚感觉到手指下有“筋结”的存在即可，待按揉后感觉到局部病变组织比较松软时，对患踝施以摇、拔、戳手法。③摇法：施治者一手托拿住患者足跟，另一手拿捏其足背，拇指按在伤处，摇晃足踝6次；拔法：施术者与助手以适中力量相对拔伸，稍停，在拔伸情况下跖屈内翻足踝，稍停；戳法：随之反向背伸外翻足踝，并以拇指顺势戳按伤处；拔、戳操作连续做2次，连贯施以摇、拔、戳手法为1次完整操作。④轻捋收功：用拇指沿着外踝缝及外踝韧带走行区进行推捋。在进行上述治疗操作时，整套动作需衔接连贯，力量柔和适度，并注意询问患者感受，禁忌粗暴用力。每次手法治疗施行摇、拔、戳完整操作5次，专人施行，隔日治疗1次，连续治疗7次，14 d为1个疗程。

2.2 观察指标及方法

2.2.1 疼痛视觉模拟评分（VAS）：2组分别在治疗前、最后一次治疗后即刻（治疗后）、治疗结束4周后（随访）时采用VAS法对患踝疼痛情况进行评价，分值越高疼痛越剧烈。

2.2.2 Baird-Jackson踝关节评分[11]：2组分别于治疗前、治疗后、随访时对患踝进行功能评价，分值越高踝关节功能越好。

2.2.3 距骨倾斜角：2组分别于治疗前、治疗后、随访时经同一技师照摄患踝内翻应力位X线片，测量距骨倾斜角角度。

2.3 统计学方法

全部资料采用SPSS 20.0统计软件进行数据分析。计量资料符合正态分布用均数±标准差（$\bar{x}\pm s$）表示，组内比较采用配对样本t检验方法，组间比较采用独立样本t检验；计数资料用例数和百分率（%）表示，比较采用χ^2检验。$P<0.05$为差异有统计学意义。

3 疗效观察

3.1 疗效判定标准

参考《中医病证诊断疗效标准》[4]拟定。治愈：治疗后症状体征消失或基本消失，Baird-

(C)1994-2023 China Academic Journal Electronic Publishing House. All rights reserved. http://www.cnki.net

Jackson踝关节评分增加≥70%；显效：治疗后症状体征有显著改善，50%≤Baird-Jackson踝关节评分增加<70%；有效：治疗后症状体征有一定程度改善，30%≤Baird-Jackson踝关节评分增加<50%；无效：治疗后症状体征无明显改善或加重，Baird-Jackson踝关节评分增加<30%。总有效率=（治愈例数+显效例数+有效例数）/总例数×100%。

3.2 结果

3.2.1 2组临床疗效比较：观察组治愈8例、显效29例、有效9例、无效2例，总有效率95.8%；对照组治愈3例、显效19例、有效21例、无效5例，总有效率89.6%。2组总有效率比较，差异有统计学意义（P<0.05）。

3.2.2 2组治疗前后VAS评分比较：治疗后、随访时，2组VAS评分均低于治疗前（P<0.05），且观察组VAS评分均低于对照组（P<0.05）。见表1。

表1 2组治疗前后VAS评分比较（分，$\bar{x}\pm s$）

组别	例数	治疗前	治疗后	随访
观察组	48	6.83±3.03	3.13±1.54*△	1.42±0.48*△
对照组	48	6.95±2.11	3.75±1.33*	2.06±1.63*

与治疗前比较，*P<0.05；与对照组比较，△P<0.05

3.2.3 2组治疗前后Baird-Jackson踝关节评分比较：治疗前，2组Baird-Jackson踝关节评分差异无统计学意义（P>0.05）；治疗后、随访时，2组Baird-Jackson踝关节评分均高于治疗前（P<0.05），且观察组Baird-Jackson踝关节评分均高于对照组（P<0.05）。见表2。

表2 2组治疗前后Baird-Jackson踝关节评分比较（分，$\bar{x}\pm s$）

组别	例数	治疗前	治疗后	随访
观察组	48	67.34±3.08	82.13±1.85*△	93.42±0.48*△
对照组	48	66.95±3.51	72.75±1.94*	84.22±1.63*

与治疗前比较，*P<0.05；与对照组比较，△P<0.05

3.2.4 2组治疗前后距骨倾斜角比较：2组治疗前后距骨倾斜角组间比较，差异均无统计学意义（P>0.05）；治疗后、随访时距骨倾斜角与治疗前比较，差异亦无统计学意义（P>0.05）。见表3。

表3 2组治疗前后距骨倾斜角比较（°，$\bar{x}\pm s$）

组别	例数	治疗前	治疗后	随访
观察组	48	10.35±1.03	9.83±1.54	9.14±0.48
对照组	48	10.69±2.11	9.72±1.33	9.03±1.63

3.3 安全性评价

研究期间2组均未出现施治部位皮肤损伤、肿痛及皮下出血等不良反应。

4 讨论

陈旧性踝关节扭伤的成因大多为对急性踝关节扭伤治疗不当、不彻底而逐渐形成[12]。伤后踝关节周围韧带、关节囊等失去其正常的解剖形态，局部软组织发生瘢痕替代、纤维化等病理变化，导致该结构正常的弹性张力下降，踝关节的稳定性减弱，当行走活动时易反复损伤，后期造成踝关节不稳，如此形成恶性循环，部分患者甚至会发展为踝关节退行性病变，形成踝骨关节炎等。有20%～40%的踝关节扭伤会逐渐进展成为慢性踝关节外侧不稳定（chronic lateral ankle instability，CLAI）[13-14]，主要临床表现为在长时间站立或行走时踝关节酸痛、肿胀、无力，可出现习惯性踝关节扭伤。

踝关节扭伤可归为中医学“伤筋”范畴，病机主要是气滞血瘀，并可用“筋出槽”和“骨错缝”阐释，指筋骨受损后人体正常解剖结构的改变。《医宗金鉴·正骨心法要旨》曰：“或因跌扑闪失，以致骨缝开错，气血郁滞，为肿为痛宜用按摩法。”筋与骨相辅相成，“筋出槽”与“骨错缝”相互关联，二者往往同时出现，如踝扭伤后踝周的韧带等软组织因损伤而错位，可称之为筋伤出槽，此时骨骼就没有了筋的正常束缚，或是有较为细微的错位，称之为骨错缝[15]。清宫正骨手法源于清代宫廷上驷院绰班处历代传人的正骨经验，满语“绰班”即是“正骨医生”之意，“绰班处”则意为“正骨科”[16-17]。孙树椿教授在传承清宫正骨手法的基础上发展而成“清宫外踝理筋手法”，此手法治疗选点通常为患者感觉最难受、最明显或者局部已形成痉挛、条索、筋结等部位，这些病变一般是因患处组织充血、水肿、炎性物质浸润，产生“无菌性炎症”，而随着软组织修复、纤维组织增生而逐渐形成的[18]。在清宫外踝理筋手法治疗时，需注重“手摸心会”。《医宗金鉴·正骨心法要旨》云：“盖一身之骨体，既非一致，而十二经筋之罗列序属，又各不同，故必素知其体相，识其部位，一旦临证，机触于外，巧生于内，手随心转，法从手出。”在此手法的具体操作过程中，强调轻巧柔和、筋骨并重[19]。并且要根据病患的体质强弱、病变的轻重程度，关注其耐受情况，有针对性地调整力道，如面对的是新鲜损伤、病位尚较浅者，手法用

(C)1994-2023 China Academic Journal Electronic Publishing House. All rights reserved. http://www.cnki.net

力应轻缓，若是陈旧性损伤、病位较深者，需逐渐增加力道，以求力能渗透，到达病所；对于体质较弱者在行手法时应徐徐用力，对于身体强壮者，发力时应使患处有轻度疼痛感、酸胀或麻木感，但均是以病患可以耐受为度。

此外，手法的治疗时间不宜过长，本研究中每次摇拔戳手法操作5遍，每次的手法治疗时间不超过5 min，操作后大部分患者感觉到踝关节较治疗前灵活舒适、疼痛减轻。临床中发现本手法更适合功能性不稳定的踝扭伤患者，可改善患踝局部本体感受器的功能，可降低当行走于不平地面上时病患出现的恐惧感。然而，本研究发现在治疗前后患者患踝内翻应力位X线片上距骨倾斜角的改变不明显，差异无统计学意义。有学者[20]指出，当患侧踝的距骨倾斜角与健侧踝的该角差值≥15°时，提示有至少两束外踝韧带断裂。虽然本研究入组的距骨倾斜角<15°，但不能明确外踝韧带损伤的具体情况，且2组治疗前后此角度无明显变化，但患者的疼痛感觉等得到了改善，由此可见本研究采用的2种干预方法对功能性不稳定及部分机械性不稳定的踝关节均有效，但对患踝局部的骨性解剖结构有无影响尚不清楚，需要进一步研究。

总之，清宫外踝理筋手法治疗陈旧性踝关节扭伤上可缓解疼痛、改善踝关节功能，临床疗效较为确切，并有较高的安全性。值得临床借鉴并进一步研究。

参考文献

[1] FONG DT, HONG Y, CHAN LK, et al. A systematic review on ankle injury and ankle sprain in sports[J]. Sports Med, 2007, 37(1): 73-94.

[2] 陈兆军，常青，吴俊德，等. 肌骨超声观察外踝理筋手法治疗陈旧性踝关节扭伤39例[J]. 中国中医骨伤科杂志，2018，26(7)：42-46.

[3] MIKLOVIC TM, DONOVAN L, PROTZUK OA, et al. Acute lateral ankle sprain to chronic ankle instability: a pathway of dysfunction[J]. Phys Sportsmed, 2017, 46(1): 116-122.

[4] 国家中医药管理局. 中医病证诊断疗效标准：ZYT0011～0019-94[S]. 南京：南京大学出版社，1994:64-65.

[5] 黄法森，马玉峰，吴俊德，等. 苏红汤联合艾瑞昔布片治疗膝关节骨性关节炎[J]. 吉林中医药，2020，40(10)：1327-1330.

[6] 吴俊德，林彬，马占华，等. 中药熏洗结合孙氏手法治疗跟痛症临床观察[J]. 北京中医药，2020，39(6)：611-614.

[7] 何伟华，李珂，独建库，等. 肌肉功能锻炼对慢性外踝关节不稳的治疗效果[J]. 实用医学杂志，2014，30(9)：1514-1515.

[8] 姜拯坤，许佳一. "踝三针"配合专项肌力功能锻炼治疗踝关节扭伤临床研究[J]. 湖北中医杂志，2016，38(5)：56-57.

[9] 何灿. 踝关节扭伤专项功能锻炼护理的临床研究[J]. 国际护理学杂志，2016，35(20)：2870-2873.

[10] 孙树椿. 清宫正骨手法图谱[M]. 北京：中国中医药出版社，2012:209-217.

[11] BAIRDRD RA, JACKSON ST. Fractures of the distal part of the fibula with associated disruption of the deltoid ligament. Treatment without repair of the deltoid ligament[J]. J Bone Joint Surg Am, 1987, 69(9): 1346-1352.

[12] 林志斌. 陈旧性踝关节扭伤临床研究进展[J]. 亚太传统医药，2014，10(22)：33-34.

[13] 施忠民，陈城，马燕红，等. 中国慢性踝关节外侧不稳定术后康复专家共识[J]. 中华骨与关节外科杂志，2019，12(10)：747-753.

[14] ROSENBAUM AJ, TARTAGLIONE J, ABOUSAYED M, et al. Musculoskeletal health literacy in patients with foot and ankle injuries: A cross-sectional survey of comprehension[J]. Foot Ankle Spec, 2016, 9(1): 31-36.

[15] 陈兆军. 孙树椿教授外踝理筋手法治疗陈旧性踝关节扭伤临床观察及机理初探[D]. 北京：中国中医科学院，2016.

[16] 唐志岚，牛志军，朱鹏展，等. 宫廷正骨在临床教学中的实践与探索[J]. 北京中医药，2020，39(12)：1314-1317.

[17] 阿伍提·艾克木，李俊海，林留洋. 宫廷正骨手法治疗陈旧性踝关节扭伤疗效观察[J]. 现代中医临床，2016，23(1)：44-46.

[18] 高景华，张军. 孙树椿筋伤疾病诊治经验[M]. 北京：中国中医药出版社，2014：12-13.

[19] 周可林，董硕，国生，等. 宫廷推拿法治疗松弛性跖痛症的疗效观察[J]. 北京中医药，2022，41(1)：47-50.

[20] GAEBLER C, KUKLA C, BREITENSEHER MJ, et al. Diagnosis of lateral ankle ligament injuries. Comparison between talar tilt, MRI and operative findings in 112 athletes[J]. Acta Orthop Scand, 1997, 68(3): 286-290.

Curative effect observation on treatment of old ankle sprain by tendon-regulating manipulation on lateral malleolus of Qinggong combined with fumigation and washing with Chinese herbs

WU Jun-de, MA Zhan-hua, PAN Xu-yue, QI Yin-ze, CHEN Zhao-jun

（收稿日期：2022-05-22）

(C)1994-2023 China Academic Journal Electronic Publishing House. All rights reserved. http://www.cnki.net
