# Supplementary material for: Efficacy and safety of Tuina (Chinese Therapeutic Massage) for chronic ankle instability: A systematic review and meta-analysis of randomized controlled trials
Source: PLoS One. 2025 Jun 6;20(6):e0321771. doi: 10.1371/journal.pone.0321771 (PMC12143534; doi:10.1371/journal.pone.0321771)
Supplement: S2 File — (ZIP) [file pone.0321771.s004.zip › 10.清宫手法治疗陈旧性踝关节扭伤的疗效观察_李慧文.pdf]

分类号 R274　　　　学校代号 10572

UDC 610　密级 公开　　　　学　　号 20187102100

广州中医药大学

Guangzhou University of Chinese Medicine

硕士学位论文

清宫手法治疗陈旧性踝关节扭伤的疗效观察

学位申请人　李慧文

指导教师姓名　陈海云

专业名称　中医骨伤科学

申请学位类型　专业学位

论文提交日期　2021年 6 月

中国知网 https://www.cnki.net

中国知网 https://www.cnki.net

广州中医药大学研究生学位（毕业）论文

答辩委员会名单及评定意见

论文题目：清宫手法治疗陈旧性踝关节扭伤的疗效观察

<table>
<tr><td colspan="6">答辩委员会名单</td></tr>
<tr><td rowspan="6">答辩委员会组成</td><td rowspan="2">主席</td><td>姓名</td><td>职称</td><td>博导/硕导</td><td>所在单位</td></tr>
<tr><td>林定坤</td><td>主任医师</td><td>博导</td><td>广东省中医院</td></tr>
<tr><td rowspan="4">委员</td><td>吴少鹏</td><td>主任医师</td><td>硕导</td><td>广东省第二中医院</td></tr>
<tr><td>王海洲</td><td>副主任医师</td><td>/</td><td>广东省中医院</td></tr>
<tr><td></td><td></td><td></td><td></td></tr>
<tr><td></td><td></td><td></td><td></td></tr>
<tr><td colspan="2">答辩委员会秘书</td><td>侯宇</td><td>主治医师</td><td>答辩时间及地点</td><td>2021 年 5 月 18 日 08:30-12:00 广东省中医院研修楼 2402 房</td></tr>
<tr><td colspan="6">答辩委员会评定意见</td></tr>
<tr><td colspan="6">该论文研究的清宫手法配合双氯芬酸二乙胺乳胶剂外涂及功能锻炼改善陈旧性踝关节的临床效果。具有一定的临床价值及社会价值，数据资料详实，分析方法得当，结论可信。文献研究较为丰富，论文设计合理方法可行，可体现作者的科研能力，写作规范，逻辑性好。答辩时，思路清晰，问题回答流利。答辩委员会一致同意通过论文答辩，并建议授予中医硕士专业学位。</td></tr>
</table>

中国知网 https://www.cnki.net

答辩成绩：　通过（√）　不通过（　）
决议：同意（ √ ）　不同意（　）授予硕士学位

中国知网　https://www.cnki.net

摘　要

目的：

陈旧性踝关节扭伤是踝关节急性扭伤后不加以重视，未及时得到有效治疗，兼以日常生活及运动劳损，致使病情迁延不愈的临床常见疾病，以踝关节功能受限、酸痛无力，无法久行为主要症状，影响生活质量。防治陈旧性踝关节扭伤，有助于促进康复，改善患者生活质量，提高患者生活满意度。本课题通过探讨清宫手法配合双氯芬酸二乙胺乳胶剂外涂及功能锻炼的方法治疗陈旧性踝关节扭伤的临床疗效，为临床医师治疗陈旧性踝关节扭伤提供参考。

方法：

本研究通过前瞻性研究，选择自 2020 年 11 月至 2021 年 1 月在广东省中医院大德路总院骨科门诊的外踝损伤的陈旧性踝关节扭伤患者，按照纳入、排除标准将研究对象随机分成试验组和对照组，共纳入 60 例病例，其中试验组 30 例，对照组 30 例。对照组：采用双氯芬酸二乙胺乳胶剂外涂扭伤部位，频率为每天 3 次，连续使用四周。试验组在对照组的基础上加清宫手法对扭伤部位进行手法治疗，频率为每周 2 次，每次摇拔戳 7 次。具体手法包括：1）手摸心会，用拇指指腹在外踝处寻找“筋结”；2）手法理筋，采用轻、巧、柔、和的手法对“筋结”进行揉按，待“筋结”变软后以清宫手法对踝关节摇、拔、戳手法进行理筋；3）“摇”法是指医者与助手在相对拔伸下摇晃踝部6~7次，同时拇指在“筋结”处揉捻；“拔”法是在拔伸下内翻踝部；“戳”法是紧接“拔”法后，在拔伸下外翻踝部，同时拇指在伤处轻微戳按；4）轻捋收功，最后沿着肌腱韧带走行方向捋顺筋脉。试验组与对照组患者均进行功能锻炼：1）足背伸，身体直立，以对侧脚为支撑，让患肢足背伸至疼痛可耐受处或背伸至最大限度保持 20 秒；2）足跖屈，身体直立，以对侧脚为支撑脚，让患肢足跖屈至疼痛可耐受处或跖屈至最大限度保持 20 秒；3）踝关节内翻运动，身体直立，对侧脚为支撑脚，让患侧踝关节内翻至疼痛处或内翻至最大限度保持 20 秒；4）提踵练习：身体直立，双脚并拢，以脚尖为着力点，原地连续提踵，同时双手可扶墙避免摔倒。功能锻炼每次锻炼各动作做 5 遍，每天锻炼 1 次。记录病例治疗前、治疗 2 周后、治疗 4 周后的 AOFAS 踝-后足评分系统评分、视觉模拟疼痛评分，以及治疗前、后的距骨倾斜度，采用 SPSS 24.0 统计学软件分析试验组与对照组之间各指标的差异。

结果：

1. 基线资料方面，两组在性别、年龄、损伤部位、病史分布的差异均无统计学意义（$P>0.05$），两组在治疗前 AOFAS 踝-后足评分系统评分、视觉模拟疼痛评分、距骨倾斜度的差异均无统计学意义（$P>0.05$）；两组具有可比性。

2. 对两组不同时间点 AOFAS 踝-后足评分系统评分、视觉模拟疼痛评分进行重复测量方差分析，结果提示时间对两组各指标的变化均有影响（$P<0.05$）；对比治疗

中国知网 https://www.cnki.net

前与治疗 4 周后的视觉模拟疼痛评分，差异有统计学意义（P=0.000）；对比治疗前、治疗 2 周后、治疗 4 周后的 AOFAS 踝-后足评分系统评分，差异有统计学意义（P=0.000）。

3. 距骨倾斜度变化情况：治疗前两组患者距骨倾斜度无统计学差异，具有可比性(P>0.05)；治疗后试验组与对照组距骨倾斜度组内差异无统计学差异（P>0.05）；治疗结束后试验组与对照组距骨倾斜度组间无明显差异（P>0.05）。

4. 两组受试者在试验阶段均未出现不适，生命体征未出现较大波动，未报告局部过敏反应、疼痛加重或全身性反应等不良反应。

结论：

1. 清宫手法配合双氯芬酸二乙胺乳胶剂外涂及功能锻炼的方法治疗陈旧性踝关节扭伤在缓解疼痛、改善患者踝关节功能方面临床疗效明显，并且优于双氯芬酸二乙胺乳胶剂外涂+功能锻炼组。2.清宫手法和功能锻炼均无法改变踝关节解剖关系，对功能性踝关节不稳疗效显著。

关键词： 陈旧性踝关节扭伤；清宫手法；功能锻炼；双氯芬酸二乙胺乳胶剂

中国知网 https://www.cnki.net

Curative effect of the Gong Ting Manipulation treatment on old ankle sprain

Specialty: Chinese medicine
Author: Li huiwen
Tutor: Chen haiyun

Abstract

Objective

Old ankle sprain is a sprain which is not paid attention to and treated effectively in time after acute sprain of ankle joint. It is also caused by daily life and sports strain, resulting in prolonged illness. The main clinical symptoms are limited ankle function, pain and lacking in strength, unable to walk for a long time, which affects the quality of life. Prevention and treatment of the adverse effects of old ankle sprain can promote rehabilitation, improve the quality of life of patients and improve their life satisfaction. This paper discusses the clinical observation of the Gong Ting Manipulation treatment with diclofenac diethylamine emulsion for external use and functional exercise in treating old ankle sprain, so as to provide reference for clinicians in the clinical treatment of old ankle sprain.

Methods

In this prospective study, from November 2020 to January 2021, patients with old ankle sprain due to lateral ankle injury were selected from the bone outpatient service in Guangdong Hospital of traditional Chinese Medicine on Dade road.According to the inclusion and exclusion criteria, the subjects were randomly divided into the experimental group and the control group. A total of 60 cases were included, including 30 cases in the experimental group and 30 cases in the control group. The control group was treated with diclofenac diethylamine emulsion, 3 times a day for four weeks. On the basis of the control group, the experimental group was treated with the Gong Ting Manipulation treatment, twice a week, 7 times each time. The specific manipulation include: 1) Touch by hand and apprehend intentionally:use the thumb pulp to find the "tendon tubercle" in the lateral ankle; 2) Straighten out the tendon with light, skillful, soft and gentle manipulation.The method of "shaking" refers to that the doctor and assistant shake the ankle 6-7 times under the relative stretching, while the thumb knead at the "tendon tubercle"; the method of "pulling" refers to that the ankle is turned inward after stretching; the method of "poking" refers to that the doctor and assistant shake the ankle 6-7 times under the relative stretching, and the thumb is rolled at the "tendon tubercle"; the method of "pulling" refers to that the ankle is turned inward after stretching; the method of "poking" refers to that the ankle is turned outward after stretching, and the thumb is turned inward at the same time 4) stroke gently to finish the work, and finally smooth the tendons and veins along the direction of tendon and ligament. The patients in the experimental group and the control group were given

中国知网 https://www.cnki.net

functional exercise: 1) dorsiflexion,the body upright, with the intact foot as the strut, let the affected limb dorsiflexion to the pain tolerable place or back extension to the maximum for 20 seconds; 2) plantar flexion,the body upright, with the intact foot as the strut, let the affected limb plantar flexion to the pain tolerable place or plantar flexion to the maximum for 20 seconds; 3) Ankle varus training, the body upright, with the intact foot as the strut, so that the affected side of the ankle varus to pain or varus to the maximum to maintain 20s; 4) heel lifting exercise: the body upright, feet together, with the toes as the focus point, continuous heel lifting in situ, while hands can help the wall to avoid falling. Functional exercise: do each exercise five times every day. The AOFAS ankle hind foot score, the Visual Analogue Score were recorded before treatment, after 2 weeks treatment and after 4 weeks treatment. The talus inclining angles were recorded before treatment and after treatment. The differencess of each index between the experimental group and the control group were analyzed by SPSS 24.0 statistical software.

Results

1. In terms of baseline data, there were no significant differencess in gender, age, injury site and distribution of disease course between the two groups ($P > 0.05$), and there were no significant differencess in the AOFAS ankle hind foot score, the Visual Analogue Score and talus inclination angle between the two groups before treatment ($P > 0.05$); the two groups were comparable.

2.The the AOFAS ankle hind foot score and the Visual Analogue Score of the two groups at different time points were analyzed by repeated measurement ANOVA. The results showed that time had an impact on the changes of each index of the two groups ($P < 0.05$); the Visual Analogue Score before treatment and after 4 weeks of treatment was compared, and the differences was statistically significant ($P = 0.000$);There was significant differences in the AOFAS ankle hind foot score before, 2 weeks and 4 weeks of treatment ($P = 0.000$).

3.The talus inclination angle changes: before treatment, there was no statistical differences between the two groups, with comparability ($P > 0.05$); there was no statistical differences between the result before treatment and after 4 weeks treatment,both the experimental group and the control group ($P > 0.05$); after treatment, there was no significant differences between the experimental group and the control group ($P > 0.05$).

4.There was no discomfort, no big fluctuation of vital signs, no local allergic reaction, aggravation of pain or systemic reaction in the two groups.

Conclusion

1. The Gong Ting Manipulation treatment with diclofenac diethylamine emulsion for external use and functional exercise in treating the old ankle sprain has obvious clinical effect in relieving pain and improving ankle function, and it is better than diclofenac diethylamine emulsion + functional exercise group. 2. The Gong Ting Manipulation treatment and functional exercise can not change the anatomical relationship of ankle joint, which has significant effect on functional ankle instability.

中国知网 https://www.cnki.net

Key words: old ankle sprain; Gong Ting Manipulation treatment; functional exercise; diclofenac diethylamine emulgel

中国知网 https://www.cnki.net

目　　录

摘　　要 I
Abstract III
目　　录 VI
引　　言 1
第一章　文献研究 3
1.1 陈旧性踝关节扭伤的流行病学特征 3
1.2 现代医学对陈旧性踝关节扭伤的认识 3
1.3 现代医学对陈旧性踝关节扭伤的治疗方法 5
1.3.1 非手术治疗方法 5
1.3.2 手术治疗方法 5
1.4 祖国医学对陈旧性踝关节扭伤的认识 8
1.5 祖国医学对陈旧性踝关节扭伤的治疗方法 8
1.5.1 单纯手法治疗 8
1.5.2 小针刀治疗 9
1.5.3 针灸治疗 9
1.5.4 中药治疗 10
1.5.5 结合现代技术的治疗 10
1.6 清宫手法对陈旧性踝关节扭伤的治疗 11
第二章　临床研究 14
2.1 研究资料 14
2.1.1 研究对象 14
2.1.2 诊断标准 14
2.1.3 病例选择 14
2.2 研究方法 15
2.2.1 治疗前准备 15
2.2.2 治疗方法 15
2.3 观察指标 16
2.3.1 主要观察指标 16
2.3.2 次要观察指标 17
2.4 安全性观察指标 18
2.5 统计学方法 18
第三章　结果与分析 19
3.1 两组术前基线资料对比 19

中国知网　https://www.cnki.net

3.2 各组间不同时间点各指标的对比分析 20
3.2.1 试验组和对照组不同时间点 VAS 评分 20
3.2.2 试验组和对照组不同时间点 AOFAS 评分 22
3.2.3 试验组和对照组治疗前后距骨倾斜度比较 24
3.2.4 试验组和对照组治疗前后最大步行距离比较 25
3.2.5 试验组和对照组治疗 2 周与治疗 4 周后改善率比较 26
3.3 安全性分析 27
第四章 讨论分析 28
4.1 中医手法治疗陈旧性踝关节扭伤的中医基础理论 28
4.2 清宫外踝理筋手法治疗陈旧性踝关节扭伤的特色 28
4.4 基于本研究分析清宫手法治疗对陈旧性踝关节扭伤患者 VAS 评分的影响 29
4.5 清宫手法治疗对陈旧性踝关节扭伤患者 AOFAS 踝-后足评分系统评分的影响 30
4.6 清宫手法治疗对陈旧性踝关节扭伤患者距骨倾斜度的影响 30
4.7 清宫手法治疗对陈旧性踝关节扭伤患者最大步行距离的影响 30
4.8 研究的不足及展望 31
结 语 32
参考文献 33
附 录 36
致 谢 38

中国知网 https://www.cnki.net

引　言

外侧踝关节扭伤是一种常见的肌肉骨骼损伤，多发生在高低不平的路面上行走、下楼梯及日常跑跳运动中偶然出现的踝关节内翻以及重力作用导致脚踝受力不均匀或受力过大超出关节的承载能力，从而引发踝关节一系列肌腱，筋膜，神经，血管组织的疾病，其中以外侧副韧带损伤最常见，以前距腓韧带、跟腓韧带、距腓后韧带损伤为主。临床上以急性踝关节扭伤多见，而陈旧性踝关节扭伤多由急性踝关节扭伤误治、错失最佳治疗时机或过度劳累所致。陈旧性踝关节扭伤最主要的危害是后期的踝关节不稳。

在人体众多负重关节中，踝关节在维持人体平衡中发挥着尤为重要的作用。踝关节活动度不大，但承受了人体绝大部分的体重，容易在关节稳定性不足或者地面不凹凸不平以及日常体育运动时发生损伤，且该损伤与性别、年龄并不存在相关性。若在出现较严重损伤后未进行系统治疗，容易导致踝关节不稳定，在往后日常生活中较常人更容易出现踝关节扭伤，损伤结构无法较好修复，又添新伤，踝关节的肿胀疼痛等症状无法缓解，患者踝关节关节囊或者踝关节韧带上机械感受器缺乏或损伤，出现感觉功能障碍，自觉患侧踝关节力量远不及对侧，这在影像学上目前是无法被侦测出来的，更多的是患者的主观感受，这其实是功能性的踝关节不稳定；在部分严重的踝关节扭伤或反复扭伤后，踝关节结构异常，踝关节活动度较健侧增大，踝关节松动，影像学上见距骨明显倾斜，韧带出现松弛，即踝关节机械性不稳定，从而容易导致关节软骨磨损，局部慢性炎症形成，关节腔积液，踝关节肿胀疼痛症状迁延不愈，最终导致创伤性关节炎或骨性关节炎。[1-2]

对于韧带部分损伤的陈旧性踝关节扭伤，现代医学上多以止痛、消肿等对症治疗为主，往往疗效并不确切。中医上认为陈旧性踝关节扭伤属“伤筋”、“痹证”范畴，乃“骨错缝、筋出槽”，踝关节损伤后经脉受损，血溢脉外，离经之血聚于患踝发为血瘀，气血不通，不通则痛，踝部筋脉失于濡养，则关节屈伸不利，治疗上当以舒筋活络，行气活血止痛为法，这样才能达到良好的治疗效果。国内已有不少临床研究报道手法治疗在陈旧性踝关节扭伤上取得一定的效果。清宫正骨手法源于上驷院蹿班处，以“手摸心会”、“轻巧柔和”为核心理念，常于谈笑间将病治好，病人常常不知所苦，疗效独特。但目前患者对踝关节扭伤重视程度较低，且清宫手法在陈旧性踝关节扭伤的机理尚未进一步研究。因此本研究将通过观察清宫手法对陈旧性踝关节扭伤治疗前后的疼痛及踝关节功能的影响，评价清宫手法治疗陈旧性踝关节扭伤的临床效果。

中国知网　https://www.cnki.net

中国知网 https://www.cnki.net

第一章　文献研究

在日常生活的行走或跑跳活动中，踝关节都扮演着最重要的角色。踝关节主要负责下肢与地面的接触，同时负荷了全身大多数重量，由于地面的不平整性，踝关节难免发生扭伤，但是由于距下关节辅助分担了部分扭转力量，更有踝关节周围韧带的包绕对踝关节起到保护作用，因此并不是每一次的踝关节扭伤都会发生损伤。在踝关节周围的韧带当中，起到保护作用的又以内外侧副韧带为主。但当扭伤力度足够大或应力集中于某一处时，则容易发生踝关节急性损伤。损伤一旦发生，踝关节周围的韧带首先受到伤害，踝关节周围的韧带的损伤中，以外侧副韧带损伤最为常见，这与踝关节生理结构关系密切，主要是因为踝关节外踝较内踝低，并且不在同一冠状面上，加之外踝韧带强度较内踝韧带强度低，所以在日常生活中内翻损伤远远多于外翻损伤，当快速行走、跑跳时，容易造成内翻跖屈位着地，足受到内翻应力，加之重力作用，使外侧副韧带受到牵拉引起撕裂或者断裂。在外侧副韧带中由于踝关节跖屈位内翻损伤最多见，此时距腓前韧带最为紧张，所以临床发生距腓前韧带损伤的患者最多。

1.1 陈旧性踝关节扭伤的流行病学特征

踝关节扭伤在体育运动以及在日常生活中时有发生。研究发现，在美国每年大约有 200 万人发生踝关节扭伤，其中发生反复扭伤的人群竟达 73% [3]。踝关节的稳定性与其解剖形态特有着点密切的关联。踝关节扭伤时的应力方式是主要有负重和扭转，而踝关节的稳定性对负重及运动功能的维持具有极其重要的意义。而陈旧性踝关节扭伤多由急性踝关节扭伤疾病失治、误治或久伤劳损所致[4]，其最主要的危害是后期的踝关节不稳，相关文献报道，约 20%-40%的踝关节扭伤最终会演变成慢性踝关节不稳定[5,6]。大部分学者认为，慢性踝关节不稳定主要是由于陈旧性踝关节扭伤后踝关节内外侧韧带在松弛位愈合造成的，其中以踝关节外侧不稳最常见[7,8]。陈旧性踝关节扭伤患者临床表现多为在不平路面上行走时的恐惧感或不稳定感、久行后出现的关节酸痛及酸胀感，严重时可出现踝关节活动受限[9]。

1.2 现代医学对陈旧性踝关节扭伤的认识

陈旧性踝关节扭伤患者多以踝关节肿胀疼痛、活动受限、久行后的酸痛或酸胀感、踝关节乏力感甚至踝关节活动受限为主诉就诊。在临床上，部分踝关节损伤患者，X 线检查无明显改变,而踝前外侧 、前内侧长期肿胀疼痛症状无法缓解，影响日常生活活动，其实质为踝关节急性旋前、旋后、背伸等活动损伤后，周围韧带（下胫腓韧带、距腓前韧带、三角韧带等）撕裂，后期韧带瘢痕化，嵌入到关节间隙从而引踝起关节软组织损伤及滑膜炎形成，从而导致踝关节肿痛。排除了骨折、脱位，这种踝关节的长期肿痛称为踝关节软组织撞击综合征。根据损伤部位不同，又可分为踝关节前外侧 、前内侧和踝前软组织撞击综合征[10]。在踝关节扭伤患者中，约 70%的急性、偶发踝关节扭伤，可能发展为慢性踝关节不稳定[11]。因而，有过急性扭伤的患者再次扭

中国知网　https://www.cnki.net

伤的风险是常人的4.5倍。在踝关节扭伤中，外踝扭伤占90%以上，内踝扭伤则不足10%[12]，究其原因，是因为外踝水平较内踝低，外侧副韧带强度不及内侧副韧带，外侧肌群力量低于内侧肌群力量。陈旧性踝关节扭伤后期的主要危害是慢性踝关节不稳[13]。慢性踝关节不稳（Chronic Ankle Instability,CAI）可分为功能性踝关节不稳（Functional Ankle Instability,FAI）与机械性踝关节不稳（Mechanical Ankle Instability,MAI），功能性踝关节不稳是指扭伤后反复发作的"乏力感"，关节运动的随意控制失常，查体见踝关节韧带无松弛，踝关节抽屉试验及X光检查阴性，B超或磁共振检查未见韧带损伤，导致功能性不稳踝关节反复扭伤的具体原因尚未确定，其原因可能与踝关节扭伤后出现的神经肌肉功能缺陷，包括肌力不足、本体感觉缺失、肌肉激活时间延迟以及平衡控制能力下降等相关。机械性踝关节不稳的踝关节关节活动度则超过正常范围。部分研究认为踝关节关节囊或者踝关节韧带上存在机械感受器，踝关节不稳定就是机械感受器缺乏或损伤所致[14]，然而这种机械感受器的存在与否在踝关节韧带上尚未得到证实。

踝关节的稳定是由相关的骨骼、韧带及肌肉共同维持的，完整的踝关节结构是维持踝关节稳定的必要条件，共同构成踝关节的静态稳定，另外，动态稳定则有赖于重力作用、相关的肌肉活动及足与地面相互作用等的综合因素[15]。

为了解析外踝韧带的功能及其在外踝不稳定中的作用，许灿[16]使采用MRI对正常人的踝关节进行连续扫描后将图像导入三维仿真建模软件Mimics并进行相关优化，再将形成的模型导入大型通用有限元分析软件ANSYS12.0中，在ANSYS中对模型网格赋予特定的材料属性，再利用解剖学资料，在ANSYS中构建踝关节周围的主要韧带后形成模型。最后通过该有限元模型建立踝关节外侧韧带损伤模型模拟外踝损伤的研究，最后得出结论：1.距腓前韧带能够限制距骨向前移位，即前抽屉运动,而跟腓韧带和距腓前韧带在维持踝关节前向稳定性当中作用不明显。2.在踝关节中立位下进行前抽屉试验时,距骨前抽屉运动最明显。当胫骨纵向压力大于300N后,关节面的吻合成为维持踝关节稳定的最主要因素。3.内翻应力试验对检查距腓前韧带损伤不敏感，跟腓韧带是阻止距骨内翻的主要韧带；跟腓韧带断裂合并距腓前韧带损伤时则会出现明显的踝关节内翻运动时不稳。4.内旋应力试验提示：距腓前韧带是维持踝关节内旋稳定性的主要韧带，跟腓韧带和距腓后韧带在维持踝关节内旋稳定性中作用不明显。该有限元模型模拟计算得到的数据与以往文献尸体标本试验的数据基本一致,证实了模型具有良好的有效性。

对于陈旧性踝关节扭伤及踝关节不稳定的诊断，需要从外伤史、临床表现、体格检查、辅助检查方面综合考虑。在临床上陈旧性踝关节扭伤的主要表现为踝关节疼痛、局部肿胀、皮下瘀斑或伴跛行以及踝关节的反复扭伤。查体局部压痛明显，外踝扭伤者将足做内翻动作时，外踝前下方疼痛明显，距骨前移试验被用作初步判断距腓前韧带有无损伤的重要手段,前抽屉应力位下距骨前移大于5 mm或与健侧相比大于3 mm

中国知网 https://www.cnki.net

考虑存在韧带损伤，距骨倾斜应力下倾斜角大于 15°或与健侧相比大于 3°。在磁共振中如果存在韧带不显影，韧带内信号增强，出现波浪形不规则的韧带轮廓择考虑存在韧带损伤。在超声检查中，中立位与跖屈位距腓前韧带长度比值大于 1.2 则考虑存在韧带损伤。[17]。若距腓前韧带合并跟腓韧带损伤，则距骨倾斜实验为阳性。诊断陈旧性踝关节扭伤的辅助检查包括应力位 X 片、肌骨超声和磁共振检查等。在这些检查中应力位 X 片价格最低，在应力位正位 X 片上测量胫骨下关节面与距骨上关节面的成角，一般认为>10°即有临床意义[18]。角度越大，说明韧带损伤越严重。然而医用踝关节应力位摄影辅助器在国内很少有，应力位 X 线片检查也很少开展。

1.3 现代医学对陈旧性踝关节扭伤的治疗方法

在现代医学中，对陈旧性踝关节扭伤的治疗，可分为非手术治疗与手术治疗两个方面。

1.3.1 非手术治疗方法

陈旧性踝关节扭伤患者若症状较轻微，对日常生活活动影响不大的可考虑采用康复训练、踝关节护具[19]、矫形鞋等保守治疗的方法。根据研究发现踝关节功能性不稳与平衡控制能力有重要关系[20,21]，康复训练主要通过加强踝部肌肉力量，增强本体感觉，提高平衡能力来达到治疗陈旧性踝关节扭伤的目的。目前康复训练主要有肌力锻炼和本体感觉训练，而联合两种训练比单独训练更有效[22]。相关研究发现[23]，等速力量训练，PNF 练习，超等长向心—离心收缩训练等针对性力量训练对踝关节不稳有很好的康复作用,也可以作为积极预防踝关节不稳的有效手段。吴贵根[24]等研究提示：手法刺激可调整患者的心理状态，使脑内致痛物质含量下降，从而提高痛阀，缓解疼痛；使体内产生组胺和类组胺，促使毛细血管扩张，加快血液循环和淋巴循环，促进了水肿的吸收，使肿胀消退。

1.3.2 手术治疗方法

踝关节韧带部分撕裂或出现功能性踝关节不稳定，当以非手术治疗为主，保守治疗效果不佳且影响患者日常生活者可考虑行手术治疗。若出现机械性踝关节不稳时，往往踝关节稳定结构出现异常，应考虑行手术干预[25]。手术应尽可能做到解剖复位，恢复损伤韧带的功能，而康复锻炼的标准需兼顾运动及神经肌肉功能康复[26]。

目前针对陈旧性踝关节扭伤出现外侧踝关节不稳的手术术式繁多，大体可以归纳为三类：（1）非解剖重建，即使用自身腓骨肌腱等肌腱重建外侧韧带，代表的手术方式有 Evans，Watson-Jones，Chrisma-Snook，Lee 及其改良术式；（2）解剖重建，即踝关节自身韧带的解剖重建，代表术式有 Broström 术及其改良术式；（3）使用异体肌腱或人造材料移植的解剖重建，代表术式有碳素纤维重建术；（4）综合应用，如 Broström-Evans-Gould 术[27]。

Evans 手术是将腓骨短肌肌腹肌腱结合处切断，然后从腓骨尖由前下向后上钻一骨隧道，再将腓骨短肌远端沿骨隧道钻入方向穿过骨隧道，再将腓骨短肌缝合固定在

中国知网 https://www.cnki.net

腓骨腓骨短肌肌腹上，改良术式则是将腓骨短肌缝合固定在腓骨骨膜上。这种术式手术步骤简单，但只重建了距腓前韧带，而没有很好地重建跟腓韧带。Waleed El Tohamy 等[28]对 14 例 15 岁至 50 岁（平均 26.6 岁）的慢性侧位不稳患者进行 Evans 术并术后后随访 2 年，术后采用 Kaikkonen 踝关节评分量表对患者踝关节进行评估，并用应力位 x 线片评估距骨倾斜和距骨前移情况，结果优良率 79%，可 14%，差 7%，从而得出结论：Evans 重建术是治疗复发性和复杂的慢性外侧踝关节不稳的一种有价值的方法，能提供良好的踝关节稳定。Hossam Diab[29]对 10 例慢性踝关节外侧韧带不稳患者采用改良 Evans 肌腱固定术，术后随访 1 年，Karlsson 评分平均值从术前的 61±3.94 增加到 88.2±3.97，Tegner 活动指数从术前平均值 3.3±0.82 显著升高到平均值 6.1±0.57。得出结论：改良 Evans 手术治疗慢性外侧踝关节不稳具有较好的临床疗效。牢靠的肌腱固定允许早期活动和更快的康复，早期恢复踝关节功能。

Watson-Jones 术式则是自在外踝尖上 2.5 cm 处钻一前后方向横形隧道，第二个隧道通过距骨颈外侧部分并与小腿纵轴一致，在腓骨尖上 1cm 处钻横行隧道，即为第三个隧道，将腓骨短肌肌健从后向前穿第一个隧道，向下下穿第二个隧道，再向后穿第三个隧道，最后将腓骨短肌肌腱缝合到腓骨短肌起点处，改良术式则是将腓骨短肌肌腱与外踝后方距腓后韧带缝合。改良 Watson-Jones 方法可有效修复踝关节距腓前韧带、跟腓韧带、三角韧带，恢复踝关节稳定，修复正常的生理结构。王建华等[30]采用改良 Watson-Jones 术式行外侧副韧带重建术治疗 21 例慢性踝关节不稳患者，术后随访 2-3 年，踝关节功能按 Good 评分标准：1 级 14 例，2 级 4 例，3 级 2 例，4 级 1 例，认为改良 Watson-Jones 术式治疗慢性外侧踝关节 不稳具有良好效果。邱士庆等[31]采用改良 Watson-Jones 方法治疗 26 例创伤性踝关节外侧不稳定，随访 3 个月～6 个月，21 例患者疼痛消失,，5 例仍有轻度疼痛，但无踝关节不稳定，效果满意。

Chrisman-Snook 术式是从外踝尖上方 2cm 处腓骨上钻一前后方向的隧道，再在跟骨外侧缘腓骨肌腱鞘处钻两相隔 1.5cm 的小孔，并用小刮匙将两孔连接成一完整的隧道。将腓骨短肌从止点处向近端切开，至腱腹交界处切断，肌腹与残余肌腱缝合。将腓骨短肌肌腱向后穿过腓骨隧道，再向前穿过跟骨隧道将足放置于中立轻度外翻位，末端与腓骨短肌止点缝合，肌腱穿过隧道处与周围软组织瓣缝合固定。周建刚[32]运用改良 Chrisman-Snook 手术治疗 14 例慢性踝关节外侧不稳定患者，术后随访 6-56 个月，平均随访 27.5 个月，按踝-后足评分标准，优 8 例，良 5 例，差 1 例，优良率达 92.9%。因此得出结论：改良 Chrisman-Snook 手术治疗外侧慢性踝关节不稳定的近中期疗效良好。周一飞等[33]采集 18 具冰冻下肢标本平均分为 3 组，A 组为对照组，B、C 组切断距腓前韧带和跟腓韧带，造成外侧副韧带II度损伤，并采用 Evans 术式和 Chrisman-Snook 术式重建外侧副韧带后进行生物力学测试，观察胫距关节和距下关节侧方应力加载下的位移情况，得出结论：踝关节外侧副韧带损伤造成机械性踝关节不稳的手术治疗方法中，Chrisman-Snook 术式比 Evans 术式在踝关节重建术后初期稳定

中国知网 https://www.cnki.net

性更好，认为 Chrisman-Snook 术式更符合生物力学原理。较同类的非解剖重建术式，Chrisman-Snook 术式具有以下优点：(1)重建了距腓前韧带和跟腓韧带，(2)使得踝关节和距下关节同时获得稳定，(3)手术操作简单。

外侧踝关节韧带解剖重建的代表术式有 Broström 术及其改良术式。而由于该术式常用的改良术式为 Broström-Gould 术。Broström 术是在腓骨远端上方约 1cm 处置入第一颗锚钉，在第一颗锚钉上方约 1cm 处置入第二颗锚钉。使用针头将锚钉上的缝线从踝关节前外侧，距离腓骨远端约 1.5 cm 处穿出，以同样的方法将缝线从前外侧向内侧依次穿出。4 根缝线相距约 5mm，再将所有缝线从外侧入路穿出，同时将踝关节放置于中立位，术者进行收紧打结。王国强等[34]对 23 例患者均采用关节镜下 Broström Gould 术修复踝关节外侧副韧带，术后随访 13～28 个月（平均 19.5 个月），AOFAS 踝关节评分从术前（52.8±14.3）分增加到末次随访（91.2±6.4）分，取得良好疗效。陈明亮等[35]对 40 例踝关节外侧副韧带损伤患者进行开放（22 例）与关节镜下（18 例）改良 Broström 术式治疗，术后随访开放组出现 2 例神经损伤，关节镜组出现 1 例，所有患者术后 12 周均能达到受伤前的运动水平，提示关节镜下改良 Broström 术式短期疗效优于开放术式，长期疗效相似。

在人工合成材料替代重建术中，碳素纤维是最常用的人工合成材料，然而，对碳素纤维重建踝关节外侧韧带的长期疗效尚未明确。Becker H P 等[36]采用改良 Evans 肌腱固定术治疗 30 例踝关节外侧韧带断裂患者，对其中的 23 例患者行踝关节外侧韧带碳素纤维替代术。结果显示：与碳素纤维替代术相比，肌腱固定术后的踝关节背伸和内翻明显受限。虽然从影像学上看两组术后稳定性均得到改善，但均无法减缓踝关节退行性病变的进展。肌腱固定术后，足底压力分布测试显示中足负荷较对侧足增加 20%，脚内侧的负荷显著增加，而碳素纤维替代术后发现双脚负荷对称。最终得出结论：肌腱固定术和踝关节韧带的解剖重建碳素纤维替代术在主观上是相似的。然而，肌腱固定术对足功能和活动范围的影响较大。

由于不同术式具有不同的优缺点，有学者提出将不同术式组合而成的综合型手术方式。Ng Z D[27]采用改良 Broström-Evans-Gould 手术治疗慢性踝关节外侧韧带不稳定，术后疗效达优 17 例（81%），良 4 例（19%），提示改良 Broström-Evans-Gould 手术对治疗慢性踝关节外侧韧带不稳定有效，由于关节过度松弛在亚洲人群中的患病率较高，因此认为改良 Broström-Evans-Gould 手术在亚洲人群中效果更佳。

综上所述，对于陈旧性踝关节扭伤后出现踝关节外侧机械性不稳的患者，主张首选解剖修复，解剖修复能减少创伤的同时，恢复韧带的正常解剖结构，这有助于重建关节本体感觉，更就有手术操作简单、并发症少、近期及远期效果均较好的优点；非解剖重建则需要取自体组织，手术创伤较大，操作相对复杂，手术时间较长，同时难以恢复踝关节正常的生物力学特性，近期效果虽可，远期效果较差，只有在解剖重建术不适用时才选择。人造替代材料虽然可以恢复关节稳定性，但牺牲了本体感觉，因

中国知网 https://www.cnki.net

此并不能预防踝关节再次损伤，且远期疗效仍有待考证。另外，综合型术式的文献报道较少，无法很好判断其疗效。

1.4 祖国医学对陈旧性踝关节扭伤的认识

祖国传统医学认为陈旧性踝关节扭伤属“伤筋”、“痹证”范畴，证候属“筋脉失养”，乃“骨错缝、筋出槽”。《素问·痹论》提到：“风寒湿三气杂至，合而为痹。”踝关节扭伤后局部筋脉损伤，血溢脉外，气滞血瘀，经脉不通，不通则痛；同时痛性活动受限致气血运行减缓，而久病必虚，乃形成本虚标实之证，瘀阻踝关节而致踝关节肿胀迁延。若血瘀未除，反复损伤，局部经脉失于濡养，风寒湿邪乘虚而入，内瘀及外邪痹阻肌肉筋脉。则关节肿胀、疼痛，活动不利，遇风寒湿加重。治当祛风散寒，宣痹除湿，行气活血止痛为则。

1.5 祖国医学对陈旧性踝关节扭伤的治疗方法

1.5.1 单纯手法治疗

在中医骨伤科学“筋骨并重”的治疗原则指导下，手法在治疗陈旧性踝关节扭伤中有其积极作用。手法因为其中的揉、按、推挤及拔伸牵引等方法，从而起到松解关节、行气活血、通络止痛的作用，从而可以纠正踝关节“筋出槽”的情况，这也是在中医治疗陈旧性踝关节扭伤中比较常见的治疗方法之一。陈兆军等[37]使用清宫外踝理筋手法治疗陈旧性踝关节扭伤 52 例，对比功能疗法锻炼治疗 37 例，两组患者治疗 1 个疗程，疼痛评分均有降低, AOFAS 评分也同步提高, 治疗组较对照组改善明显，提示清宫手法治疗陈旧性外踝扭伤对缓解疼痛、改善踝关节功能上具有良好的疗效, 且优于功能疗法锻炼组, 但并不能改变踝关节的异常解剖结构。林世豪[38]采用理筋正骨手法治疗陈旧性踝关节扭伤 40 例，对比特定电磁波治疗器（TDP 灯）照射治疗 40 例，经 2 个疗程治疗后，治疗组总有效率达 95%，对照组总有效率为 37.5%，治疗后两组患者压痛程度和 Baird-Jackson 踝关节评分较治疗前均有明显改善，且治疗组疗效明显优于对照组，且治疗过程中,理筋正骨手法操作简单,患者痛苦少,容易被接纳。付文博[39]采用摇拔戳手法治疗陈旧性踝关节扭伤，试验组予摇拔戳手法治疗，手法操作为 3 次，疗程为 1 周，对照组采用肌内效贴扎贴，每日 1 次，单次贴扎维持约 24h 左右，疗程 1 周，以治疗前、治疗后 1 天、治疗后 7 天、治疗后 1 个月为时间节点采集资料并分析后发现：（1）两种治疗方法在治疗陈旧性踝关节扭伤上均可达到缓解疼痛、减轻肿胀、改善踝关节的功能的目的；（2）试验组与对照组在减轻疼痛、肿胀症状上没有差异；（3）在 Takakura 功能评分中，试验组与对照组的总有效率接近。阿伍提·艾克木等[40]采用宫廷正骨手法对 54 例陈旧性踝关节扭伤患者进行治疗，治疗前后对患侧踝关节进行 Baird-Jackson 踝关节评分，治疗后有效率达 100%。根据 Baird-Jackson 踝关节评分，治疗后优良率为 92.6%，从而得出结论：宫廷正骨手法对陈旧性踝关节扭伤患者踝关节疼痛、稳定性、行走能力、跑步能力、工作能力、活动范围等方面均有很好的改善。

中国知网 https://www.cnki.net

1.5.2 小针刀治疗

小针刀疗法是一种微创闭合性松解术，具有伤口小、无须缝合及治愈率高等特点，但是对操作者在踝关节解剖知识方面要求较高。小针刀治疗陈旧性的踝关节扭伤，大部分患者筋脉受损，瘀血内阻，导致踝关节筋脉失养，踝关节周围粘连严重，踝关节活动明显受限，因此通过针刀皮下切割的方法可以将踝关节周围粘连的组织剥离，降低筋膜内的压力，从而改善局部的血液循环，使得关节恢复正常[41]。也有专家认为小针刀治疗表皮伤口虽小，皮下软组织剥离较大，短期疗效可，但不能防止软组织粘连再发，因此对小针刀治疗方法抱怀疑态度。王冠军[42]报道用小针刀治疗陈旧性踝关节扭伤 132 例，观察组和治疗组同时给予小针刀治疗，观察组额外给予中药熏蒸治疗，经过治疗，观察组有效率为 69.8%，对照组有效率为 50%，观察组 VAS 评分、Kofoed 踝关节功能评分均优于对照组。秦民安[43]报道使用小针刀治疗陈旧性踝关节扭伤 25 例，治疗结果显示优 18 例，良 4 例，中 3 例，得出结论：小针刀治疗陈旧性踝关节扭伤，从治疗结果来看，对于合并踝关节不稳，或有骨关节炎改变者也有效，但长期疗效不佳。黄伟[44]采用小针刀联合封闭治疗陈旧性内翻型踝关节扭伤，将 60 例患者平均分为三组，治疗组（小针刀+激素封闭治疗）、对照组I（小针刀治疗），对照组II（激素封闭治疗），记录治疗前、治疗后 1 周、8 周、6 个月的 VAS 评分、Mazur 踝关节功能评分、踝关节内翻、跖屈活动角度结果，治疗后 1 周:治疗组总有效率为 94.7%,而对照组I总有效率为 84.2%,对照组II总有效率是 85.0%。治疗后 6 个月:治疗组总有效率为 94.7%,而对照组 I 总有效率为 73.6%,对照组II总有效率则为 65.0%。因此认为小针刀联合封闭对陈旧性内翻型踝关节扭伤后的慢性疼痛等症状的缓解有良好疗效，且能有效改善踝关节活动度，值得临床推广。

1.5.3 针灸治疗

针法是指在中医理论的指导下在特定穴位处把针按照一定的角度刺入并运用捻转、提插等手法来刺激局部从而达到治疗疾病的目的。灸法是以预制的灸炷或灸草在体表特定穴位上烧灼、熏熨，利用热刺激达到治疗疾病的效果。临床上对于陈旧性踝关节扭伤患者运用针灸治疗能够达到疏通经络，行气止痛的效果。陈壮娜[45]分别使用常规针刺法电温针、齐刺法电温针治疗陈旧性踝关节扭伤患者 80 例，治疗后 VAS 评分均降低且，齐刺法电温针治疗组踝关节能力评分、患者满意度优于常规针刺法电温针治疗组，因此认为实施齐刺法电温针治疗陈旧性踝关节扭伤对疼痛和关节功能改善更佳，临床效果显著。李军[46]采用常规针灸、电针灸、温针灸治疗陈旧性踝关节扭伤患者各 40 例，对比三组患者的治疗效果、疼痛程度、SF-36 评分和 Karlsson-perterson 评分，结果提示电针灸、温针灸组治疗有效率均高于常规针灸组，疼痛评分更低、SF-36 评分更高，Karlsson-perterson 评分常规针灸＞电针灸＞温针灸，得出结论：使用电针灸与温针灸治疗陈旧性踝关节扭伤可有效缓解疼痛、改善症状，其中温针灸治疗时关节活动度改善效果更优。周彬[47]采用电针、火针联合疗法治疗陈旧性踝关节内翻扭伤

中国知网 https://www.cnki.net

患者，其中治疗组 33 例采用电针结合火针的联合治疗法，对照组的 31 例采用电针治疗，结果提示联合治疗组优 26 例，良 7 例，可 0 例，差 0 例；电针对照组优 4 例，良 4 例，可 19 例，差 4 例，治疗组疗效明显优于对照组，疗效显著，因此考虑电针结合火针治疗陈旧性踝关节内翻损伤疗效更理想，值得临床推广。

1.5.4 中药治疗

中药治疗踝关节陈旧性扭伤可分为两类：（1）中药内服（汤剂或中成药内服），（2）中药外用（外敷、熏蒸或外洗），中药治疗外伤患者，主要由活血化瘀，行气通络止痛的药物组成为主。在治疗慢性踝关节扭伤的中药中，桃仁、红花、牛膝、伸筋草、透骨草、当归、三七等出现频率较高。外用中药中，也有使用经验方和地方独特药物，例如四黄散、祛瘀止痛散、柏油膏等，均对慢性踝关节扭伤一病有较好的临床疗效。中药熏洗疗法是利用中药煮沸后的温热透达作用以及中药自身的药物作用来刺激损伤的部位，从而达到疏经通络，行气止痛，调和气血的作用。但临床中为了获得更好的疗效，大都会与其他的治疗方法相结合使用。另外，中药治疗常常配合其他疗法来达到更好的治疗效果。毛庆友[48]将 111 例陈旧性踝关节扭伤患者根据不同治疗方式分为两组，治疗组 56 例，对照组 55 例，治疗组予以关节松动术、运动疗法及中药熏洗联合治疗，对照组仅予中药熏洗治疗，治疗 1 个月后统计两组患者治疗后 MBI 评分均高于治疗前评分，且治疗组的 MBI 评分明显高于对照组，治疗组的 VAS 评分也明显低于对照组，结果显示：中西医结合治疗陈旧性踝关节扭伤疗效显著，且优于单纯中药熏蒸治疗。张阳[49]将 84 例陈旧性踝关节扭伤患者对照组和观察组，两组病例数相同。对照组给予踝关节功能锻炼，观察组在对照组基础上给予温针结合中药熏洗治疗，治疗 3 个月后统计结果显示：治疗后两组 VAS 疼痛评分均下降，且与对照组相比，观察组降低程度更明显，治疗后两组 Baird-Jackson 踝关节评分及 AOFAS 踝与足功能评价均明显改善，且观察组评分明显优于对照组。因此得出结论：温针结合中药熏洗可有效缓解陈旧性踝关节扭伤的临床症状，减轻患者痛苦，值得临床推广。薛彬[50]等将 78 例陈旧性踝关节扭伤的患者分为治疗组 39 例和对照组 39 例，治疗组采用魏氏手法结合特色中药外洗治疗，对照组采用主动功能锻炼结合扶他林软膏治疗，经过六周的治疗，治疗组的优良率为 83.7%，对照组则为 71.1%，且治疗组 AOFAS 评分明显比对照组高，因此认为与对照组相比，治疗组更好的远期治疗效果更好。在许多中药治疗陈旧性踝关节扭伤的文献中，大多数都以踝关节功能评分、疼痛评分等评价治疗效果，主观性较强，缺乏影像学资料支持，无法判断所运用的治疗方法对踝关节功能的影响。

1.5.5 结合现代技术的治疗

随着医学科技的发展和推广，目前有许多临床治疗方法运用到于骨关节疾病的治疗中，这些现代的治疗的方法常配合传统的中医药治疗方法联合治疗，在某种程度上可以取得良好的疗效。陆亚丽[51]采用超声波结合中药熏洗治疗陈旧性踝关节扭伤患者

中国知网 https://www.cnki.net

178 例平均分为治疗组和对照组，对照组采用超声波进行踝关节局部治疗，治疗组在对照组治疗基础上结合中药熏洗治疗，超声波和中药熏洗均每日 1 次, 10 次为 1 疗程。结合踝关节功能锻炼和踝两侧的肌肉力量加强训练，观察组有效率 94.4%，对照组有效率 83.1%，治疗效果差异有统计学意义，提示超声波结合中药熏洗治疗陈旧性踝关节扭伤临床疗效显著。余雷[52]纳入单侧陈旧性踝关节扭伤患者 68 例，随机分为低温等离子刀成形术组（治疗组）和小针刀组（对照组）各 34 例,两组患者均予以中药物熏洗患踝关节，15 天一个疗程，2 个治疗疗程后结果提示治疗组痊愈 8 例，痊愈率为 23.53%，总有效率为 97.06%；对照组痊愈 6 例，痊愈率为 17.65%，总有效率为 94.12%。低温等离子刀成形术组在 AOFAS 踝与足功能评分、Baird-Jackson 踝关节评分、踝关节功能、运动工作能力等均优于小针刀组，低温等离子刀成形术能够有效缓解患者陈旧性踝关节扭伤症状，在临床上具有安全性能好、创伤小、副作用少等特点，有效改善患者的症状。王敏[53]将 60 例陈旧性踝关节扭伤患者随机分为 2 组，治疗组采用中药熏蒸及手法联合关节腔内注射臭氧治疗，对照组采用中药熏蒸和双氯芬酸钠凝胶治疗，治疗 2 个疗程后，治疗组治愈率为 97%，对照组治愈率为 80%，2 组比较有显著性差异，提示中药熏蒸及手法联合关节腔内注射臭氧治疗陈旧性踝关节扭伤疗效满意，且操作安全。王筱锋[54]将 38 例陈旧性踝关节扭伤患者，均采用体外发散式冲击波踝关节局部冲击治疗，每周治疗 2 次，8 次为 1 个疗程，治疗后治愈 27 例(71.05%)，显效 8 例（21.05%），无效 3 例（7.89%），总有效率达 92.1%，疗效显著。冲击波作为一种非侵入性治疗方法, 既安全无创性, 又具有一定的有效性，在临床治疗疼痛方面已取得了广泛的应用。此外，根据研究认为冲击波疗法对人体组织肌肉的作用有：（1）机械效应：通过机械作用对人体产生按摩，促进新陈代谢，加强血液循环和淋巴回流。（2）空化效应：气体在冲击波的应力作用下以极高速度膨化，改善局部血液循环，松解软组织粘连。（3）代谢激活效应：改善治疗区域的新陈代谢，松解钙质沉着，减轻炎性反应及水肿。（4）镇痛效应：对神经末梢组织产生超强刺激而引起细胞周围自由基的改变，释放抑制疼痛的物质，提高大脑对疼痛的阈值从而缓解疼痛[55]。

1.6 清宫手法对陈旧性踝关节扭伤的治疗

清宫外踝理筋手法为清宫正骨派治疗踝关节扭伤的代表手法，手法以“手摸心会”、“轻巧柔和”为核心，名老中医孙树椿为清宫正骨手法的传人。现代医学的发展和解剖学的丰富，为“手摸心会”提供更有力的解剖学依据。清宫外踝理筋手法是针对“筋结”进行治疗的，“筋结”是肌腱、韧带损伤以后, 由于损伤部位的出血、渗出、炎症细胞浸润，以及损伤的韧带部分撕裂后短缩，局部形成的炎性反应，随着组织的修复，纤维组织自我修复及增生，形成“筋结”。孙老在长期临床实践中, 探索并总结：不同部位的损伤后形成的“筋结”形状不一但存在一定的规律性。对于陈旧性踝关节扭伤的治疗, 孙老主张临床查体一定要和解剖知识相结合。踝关节内翻扭伤最容易伤及踝关

中国知网 https://www.cnki.net

节距腓前韧带，但常常累及其他外侧副韧带，更严重的会出现肌腱断裂、软骨损伤、骨折等情况，这需要临床查体与影像学检查相结合，避免误诊、漏诊。对于陈旧性踝关节扭伤患者，查体时一般在距腓前韧带周围可以触及豆粒大小的"筋结"，在按压这些"筋结"时患者会出现不同程度的的疼痛，这往往就是治疗该病的关键点所在。这就是所谓的"手摸心会"。

在踝关节扭伤的手法治疗中，清宫手法不主张运用用力按压、揉等对局部软组织强刺激的手法，而主张轻揉"筋结"后运用别具一格的"摇、拔、戳"手法进行松解与复位（具体见图 1-3），此手法也因"轻巧柔和"的特点而广为患者接受。"摇"法是指医者与助手在相对拔伸下摇晃踝部 6~7 次，同时拇指在"筋结"处揉捻；"拔"法是在拔伸下内翻踝部；"戳"法是紧接"拔"法后，在拔伸下外翻踝部，同时拇指在伤处轻微戳按。"戳"法是清宫正骨的独特手法，"摇"法则可以放松踝关节周围软组织，从而解除踝关节"交锁"状态；拔法通过拔伸、牵引的方法促进踝部血液循环及淋巴回流，达到消肿的效果；"戳"法可以使轻微移位的踝关节复位，恢复正常的踝关节解剖结构，解决"筋出槽"的问题；同时，轻巧柔和的手法特点也能在尽可能减少患者痛苦甚至在无痛的情况下达到良好的治疗效果，提高患者满意度。

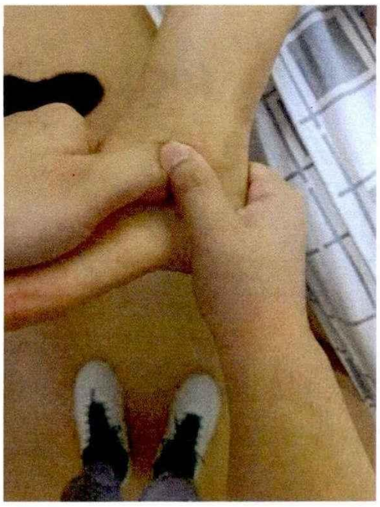

图 1 "摇"法示意图

中国知网 https://www.cnki.net

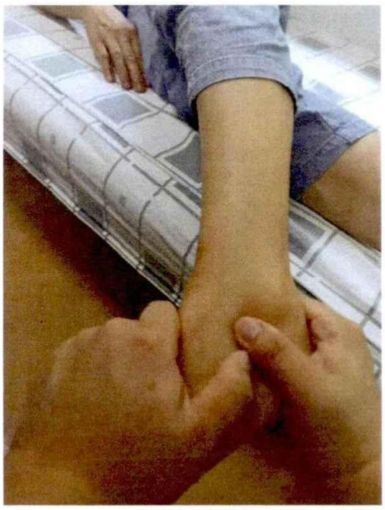

图 2 "拔"法示意图

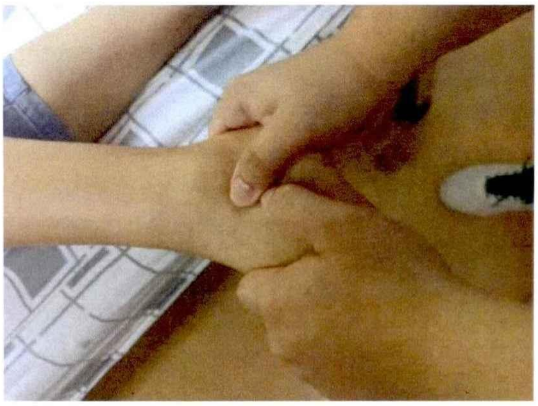

图 3 "戳"法示意图

综上所述，以上文献报道可作为进一步研究陈旧性踝关节扭伤治疗的参考与借鉴。医者与患者都应加强对踝关节扭伤的重视，医者更要加强宣传，提高患者对踝关节扭伤的重视程度。对于已经发生的踝关节扭伤，医者需进一步探索能够提高患者依从性、疗效确切、减轻患者痛苦的治疗方法。在做相关临床观察试验时，研究者当进一步提高研究设计严谨与完善程度，需要更全面更具体的疗效评价，并从有效率、症状缓解、功能改善、影像学变化等方面客观地进行疗效评价。

中国知网 https://www.cnki.net

第二章　临床研究

2.1 研究资料

2.1.1 研究对象

选择 2020 年 10 月-2021 年 1 月期间在广东省中医院大德路总院骨科门诊因陈旧性外侧踝关节扭伤就诊的患者 60 例，患者对本研究知情并同意入组进行临床试验观察。

分组方法：随机数字表法将病例分为试验组（在对照组的基础上加用清宫手法治疗）和对照组（双氯芬酸二乙胺乳胶剂外涂+功能锻炼）。患者按照就诊时间顺序进行编号，然后查阅随机数（见附录 2）字表产生随机数字，将得到的随机数字与患者的就诊序号相互对应，把末位数为奇数的随机数字对应的患者分配至试验组，末位数为偶数的随机数字对应的患者分配至对照组，平衡各组例数。计划试验组、对照组各 30 例，共 60 例。

样本量估计：采用定量数据成组设计的样本含量估计研究所需要的样本量：其中 α=0.05，β=0.10，q1=q2=0.5，采用双侧检验。σ 为两总体得分水平标准差的估计值，假设其相等；δ 为两样本均数之差；AOFAS 踝关节功能评分量表得分为计算指标，根据预实验结果，δ=8.31，σ=8.05，代入公式计算得出总样本量为 42 例，干预组 21 例，对照组 21 例。

$$N=\frac{(q_1^{-1}+q_2^{-1})(t_{\alpha/2}+t_\beta)^2\sigma^2}{\delta^2}=42,$$

考虑 20%的病例脱落率，N 总数=52.5≈53，因此样本最低样本量为 53；因此本研究纳入 60 例，按照 1:1 分配比例，其中试验组、对照组各有 30 例。

2.1.2 诊断标准

诊断标准参考 1994 年国家中医药管理局《中医病证诊断疗效标准》。

陈旧性踝关节扭伤

①有明确的踝部外伤史；

②扭伤时间在 3 周以上；

③踝关节疼痛、无力，不能久行，影响生活、工作和运动；

④内踝或外踝前下方处有不同程度的肿胀和压痛；

⑤X 线片未见骨折和脱位。

2.1.3 病例选择

2.1.3.1 入选标准

①符合上述诊断标准且为功能性外侧踝关节不稳的踝关节扭伤。

②外踝局部压痛明显，内翻扭伤者将足做内翻动作时，外踝前下方剧痛。

③患者为外踝韧带损伤的陈旧性踝关节扭伤，抽屉试验阴性，需影像学排除骨折，

中国知网 https://www.cnki.net

查体无法判断肌腱是否断裂时需进一步行 MRI 或彩超排除。

④年龄 18-65 岁。

⑤愿意接受清宫手法、双氯芬酸二乙胺乳胶剂外涂及功能锻炼治疗的。

⑥能积极配合,完成临床观察者。

2.1.3.2 排除标准

①相关检查提示骨折以及韧带断裂者。

②更换或兼用了其它治疗方法,无法判断疗效者。

③存在影响治疗的合并情况，如恶性肿瘤、妊娠等

④精神病等无法配合的患者。

⑤拒绝使用清宫手法以及双氯芬酸二乙胺乳胶剂外涂治疗者，不同意配合完成临床治疗者。

⑥对双氯芬酸二乙胺乳胶剂过敏者。

2.1.3.3 脱落标准

①产生重大的不良事件或并发症，并不适合完成后续的试验程序。

②治疗或用药程序中表示抗拒延续试验。

③按计划完成全部研究而数据缺失者。

2.2 研究方法

2.2.1 治疗前准备

研究所选取的病例均需签署临床研究的知情同意书。术前需进行相关体格检查，完善踝关节应力位 X 光检查，无法排除外侧韧带是否断裂者需进一步完善彩超或磁共振排除。排除外踝韧带断裂后将患者按照随机数表进行分组并完善治疗前 AOFAS 踝-后足评分系统评分、视觉模拟疼痛评分。患者治疗过程中操作与管理均由同一操作者按照治疗方案完成。本研究已通过医院伦理委员会审批。

2.2.2 治疗方法

对照组：双氯芬酸二乙胺乳胶剂外涂治疗+功能锻炼：采用双氯芬酸二乙胺乳胶剂外涂扭伤部位，频率为每天 3 次，连续使用四周。功能锻炼：1）足背伸，身体直立，以对侧脚为支撑，让患肢足背伸至疼痛可耐受处或背伸至最大限度保持 20 秒；2）足跖屈，身体直立，以对侧脚为支撑脚，让患肢足跖屈至疼痛可耐受处或跖屈至最大限度保持 20 秒；3）踝关节内翻运动，身体直立，对侧脚为支撑脚，让患侧踝关节内翻至疼痛处或内翻至最大限度保持 20 秒；4）提踵练习：身体直立，双脚并拢，以脚尖为着力点，原地连续提踵，同时双手可扶墙避免摔倒。功能锻炼每次锻炼各动作做 5 遍，每天锻炼 1 次。

试验组：在对照组的基础上，加清宫手法对扭伤部位进行手法治疗，频率为每周 2 次，每次摇拔戳 7 次。具体手法包括：1）手摸心会，用拇指指腹在外踝处寻找“筋结”；2）手法理筋，采用轻、巧、柔、和的手法对“筋结”进行揉按，待“筋结”变软后

中国知网 https://www.cnki.net

以清宫手法对踝关节摇、拔、戳手法进行理筋；3）“摇”法是指医者与助手在相对拔伸下摇晃踝部 6~7 次，同时拇指在“筋结”处揉捻；“拔”法是在拔伸下内翻踝部；“戳”法是紧接“拔”法后，在拔伸下外翻踝部，同时拇指在伤处轻微戳按；4）轻捋收功，最后沿着肌腱韧带走行方向捋顺筋脉。

本研究由同一医生进行治疗。

2.3 观察指标

2.3.1 主要观察指标

2.3.1.1 AOFAS 踝-后足评分系统评分

记录治疗前、治疗 2 周、治疗 4 周的 AOFAS 踝-后足评分系统评分，此评分包括①疼痛，②功能和自主活动、支撑情况，③最大步行距离，④地面步行情况，⑤反常步态，⑥前后活动，⑦后足活动，⑧踝-后足稳定性，⑨足部对线。从这 9 个方面对踝关节功能评价从而获得更客观可靠的疗效分析。具体见表 1。

表 1 AOFAS 踝-后足评分系统（AOFAS Ankle Hindfoot Scale）

	评分
疼痛（40 分）	
无	40
轻度，偶尔	30
中度，常见	20
严重，持续	0
功能和自主活动、支撑情况（10 分）	
不受限，不须支撑	10
日常活动不受限，娱乐活动受限，需扶手杖	7
日常和娱乐活动受限，需扶手杖	4
日常和娱乐活动严重受限，需扶车、扶拐、轮椅、支架	0
最大步行距离（街区数）（5 分）	
>6 个	5
4 ～ 6 个	4
1 ～ 3 个	2
<1 个	0
地面步行（5 分）	
任何地面无困难	5
走不平地面、楼梯、斜坡、爬梯时有困难	3
走不平地面、楼梯、斜坡、爬梯时有困难	0
反常步态（8 分）	
无、轻微	8
明显	4
显著	0
前后活动（屈曲加伸展）（8 分）	
正常或轻度受限（>30°）	8
中度受限（15°-29°）	4
重度受限（<15°）	0

中国知网 https://www.cnki.net

后足活动（内翻加外翻）（6 分）	
正常或轻度受限（75%-100%正常）	6
中度受限（25%-74%正常）	3
重度受限（<25%）	0
踝-后足稳定性（前后，内翻-外翻）（8 分）	
稳定	8
明显的不稳定	0
足部对线（10 分）	
优：跖行足，踝-后足排列正常	10
良：跖行足，踝-后足明显排列成角，无症状	5
差：非跖行足，严重排列紊乱，有症状	0

优：90 ～ 100 分；良：75 ～ 89 分；可：50 ～ 74 分；差：50 分以下

2.3.1.2 视觉模拟疼痛评分

记录治疗前、治疗 2 周、治疗 4 周的视觉模拟疼痛评分，0 分表示无痛；3 分以下表示有轻微的疼痛，患者能忍受；4 分-6 分表示患者疼痛较重，并影响睡眠，但尚能忍受；7 分-10 分患者有逐渐强烈的疼痛疼痛难忍；10 分最痛。

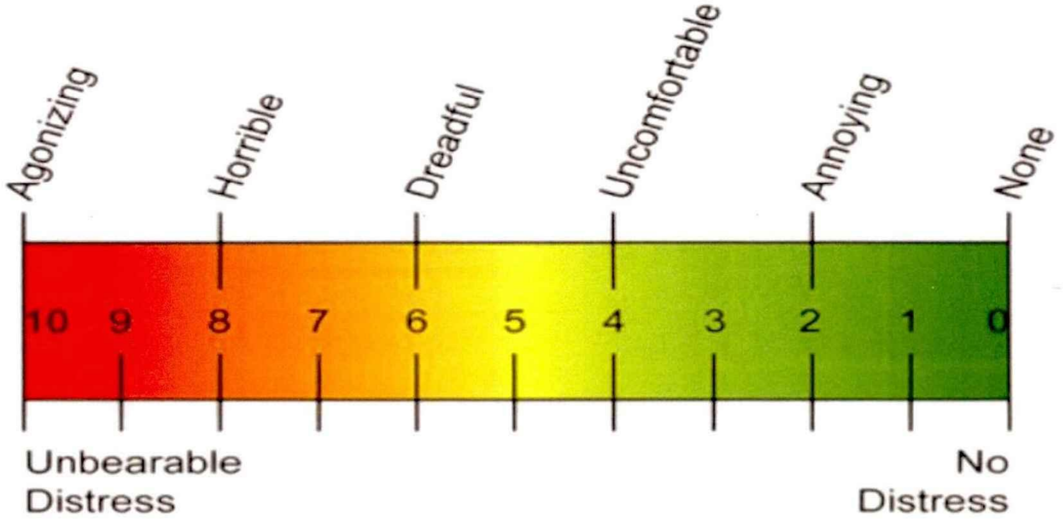

图 4 视觉疼痛模拟评分法（Visual Analogue Scale，简称 VAS）

2.3.2 次要观察指标

2.3.2.1 距骨倾斜度测量

本研究中患者全部在相同条件下由一名放射科医生进行摄片操作，自制踝关节应力位固定设备下让患者尽力内翻踝关节，以患者可承受的最大疼痛为度。测量角度由研究员在我院阅片软件上进行测量。具体拍摄方法：站立位，髋、膝关节屈曲 90°，踝关节跖屈 30°摄片，辅助内翻设备具体见下图。拍摄治疗前、治疗 4 周后踝关节内翻应力位片进行比较。

中国知网 https://www.cnki.net

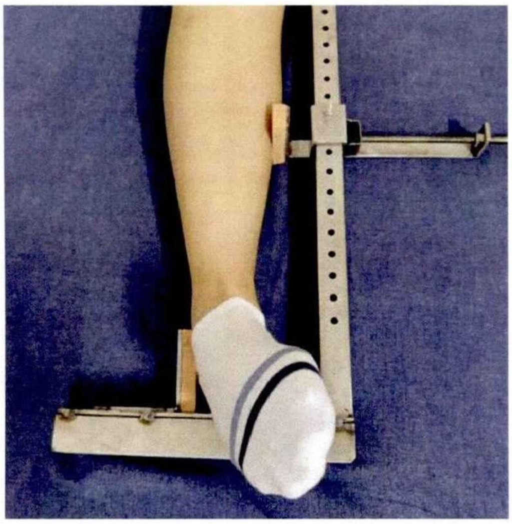

图5 内翻应力位拍片设备

2.3.2.1 改善率

记录患者治疗前、治疗4周后AOFAS评分，根据公式进行改善率计算并统计对比。

注：改善率=（治疗后AOFAS评分-治疗前AOFAS评分）/（100-治疗前AOFAS评分）×100%

2.4 安全性观察指标

观察患者生命体征：如心率、呼吸、脉搏、血压等；注意观察入选研究者有无出现过敏，若出现过敏，立即停止实验。记录是否出现治疗后不良反应，包括有局部过敏反应、疼痛加重或全身性反应等，判断是否需要停药或处理。

2.5 统计学方法

本研究运用SPSS 24.0统计软件。利用软件对收集的数据进行统计分析。计量资料用均数±标准差表示，组内比较用配对t检验，组间比较采用两独立样本t检验，对于多次测量数据采用重复测量方差分析。计数资料用频数、构成比（P）、平均秩和（$\bar{R}$）等表示，组间比较采用Fisher卡方检验。比较中医临床的有效率采用独立样本Mann-Whitney U非参数检验。检验水准α=0.05。

中国知网 https://www.cnki.net

第三章 结果与分析

3.1 两组术前基线资料对比

共有 60 例病例纳入本研究中，其中试验组 30 例，对照组 30 例，无数据缺失情况。其中试验组：男性 9 例，女性 21 例，左侧踝关节扭伤 13 例，右侧踝关节扭伤 17 例，平均年龄 29.27±9.44 岁。对照组：男性 15 例，女性 15 例，左侧踝关节扭伤 9 例，右侧踝关节扭伤 21 例，平均年龄 31.67±9.01 岁。两组研究对象一般情况中，采用 P-P 图检验年龄是否符合正态分布，结果显示，年龄变量的各点基本呈一条直线，符合正态分布，具体见图 6。符合正态分布的计量资料用均数±标准差进行统计描述，采用独立样本 t 检验进行统计推断。计数资料用构成比和率进行统计描述，采用卡方检验进行统计推断。本研究共纳入患者 60 例，试验组和对照组各 30 例。通过比较两组术前年龄、性别、扭伤部位等基线资料，发现差异无统计学意义，具有可比性。见表 2.3。

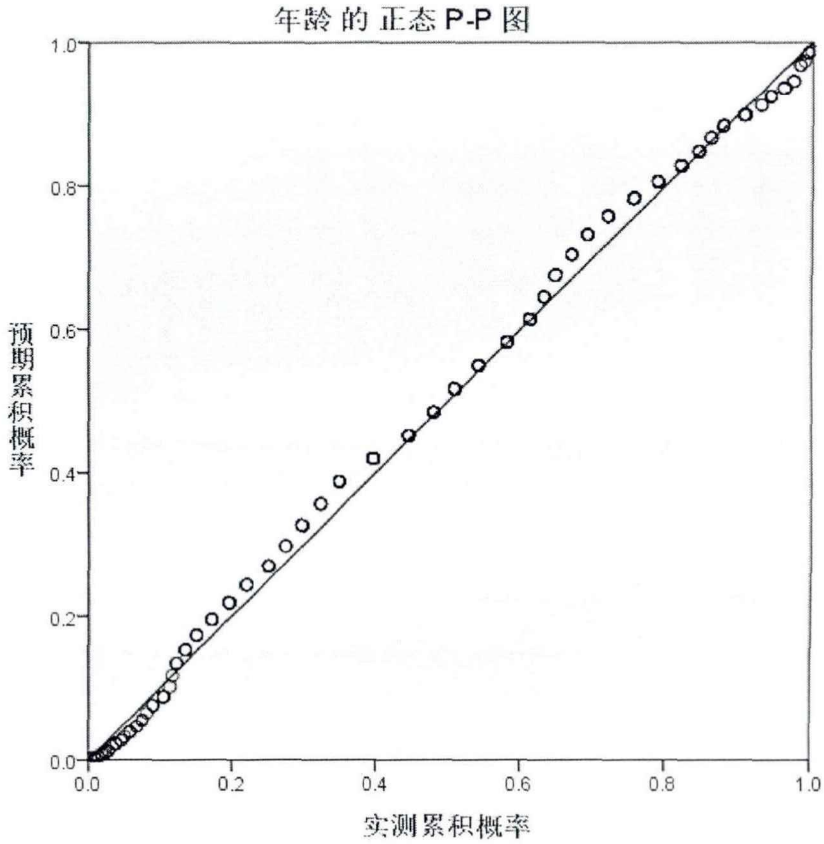

图6 年龄变量P-P图

中国知网 https://www.cnki.net

表 2 两组患者一般资料比较

项目	分类	试验组	对照组	χ^2/t	P
年龄	-	29.27±9.44	31.67±9.01	-1.008	0.318
性别	女	21（70%）	15（50%）	2.500	0.114
	男	9（30%）	15（50%）		
损伤部位	右侧	17（56.7%）	21（70%）	1.148	0.284
	左侧	13（43.3%）	9（30%）		

注：对比两组性别、损伤部位，采用卡方检验，P值均大于0.05，差异无统计学意义。对比两组年龄，采用独立样本t检验，$P>0.05$，差异无统计学意义。说明两组具有可比性。

表 3 两组患者病史分布比较（月）

组别	N	＜3	3-6	6-12	＞12	χ^2/t	P
试验组	30	14	12	3	1	1.762	0.623
对照组	30	16	9	2	3		

注：对比两组患者病史分布，采用卡方检验，P 值均大于 0.05，差异无统计学意义。

3.2 各组间不同时间点各指标的对比分析

3.2.1 试验组和对照组不同时间点 VAS 评分

采用 P-P 图检验 VAS 评分是否符合正态分布，结果显示，VAS 得分变量的各点基本呈一条直线，符合正态分布，具体见图 7。符合正态分布的计量资料重复测量数据采用重复测量方差分析进行统计推断，某一时间点的组间比较采用独立样本 t 检验进行统计分析。Mauchly 球形检验显示 VAS 评分符合球形数据要求（χ^2=2.239, P=0.326），故读取假设球形度的结果。结果显示，时间因素与分组因素之间存在交互效应（F=15.112，P=0.000），表示时间因素的作用随分组的不同而不同。交互图如图 8。两组患者 VAS 评分分均呈下降趋势，但试验组随时间下降趋势更明显。

对各时间点的数据进行组间比较，检验固定时间因素于某一水平时两组研究对象 VAS 评分的差异，采用两独立样本 t 检验进行两两比较。调整的名义检验水平为 0.05/5=0.01，需将两两比较所得 P 值与 0.01 比较，从而得出统计结论。各测量时间点 VAS 评分的组间比较结果如表 4 所示，入组当日两组得分差异无统计学意义（P=0.062＞0.01）,治疗 4 周后试验组 VAS 得分低于对照组，差异有统计学意义（P＝0.000）。

中国知网 https://www.cnki.net

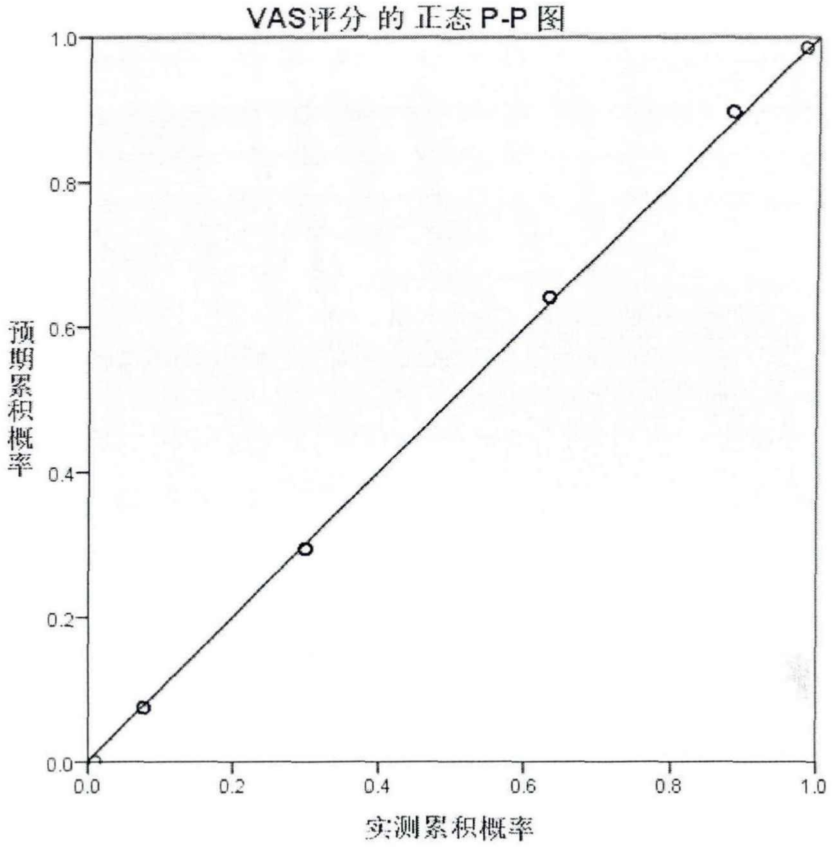

图7 VAS得分变量P-P图

表 4 试验组和对照组不同时间点 VAS 评分比较

组别	时间点			合计	F	P
	治疗前	治疗 2 周	治疗 4 周			
试验组	7.87±1.25	4.63±1.22	2.23±1.04	4.91±2.58	367.430[a]	0.000[a]
对照组	7.33±0.88	4.90±1.12	3.30±0.83	5.18±1.91	200.231[a]	0.000[a]
合计	7.60±1.11	4.77±1.17	2.77±1.08	5.04±2.27	557.087[b]	0.000[b]
F	1.906	-0.881	-4.377	1.476[b]	15.112[c]	
P	0.062	0.382	0.000	0.229[b]	0.000[c]	

注：a　时间因素重复测量方差分析的 F 统计量和 P 值

b　主效应的 F 统计量和 P 值

c　交互效应的 F 统计量和 P 值

中国知网 https://www.cnki.net

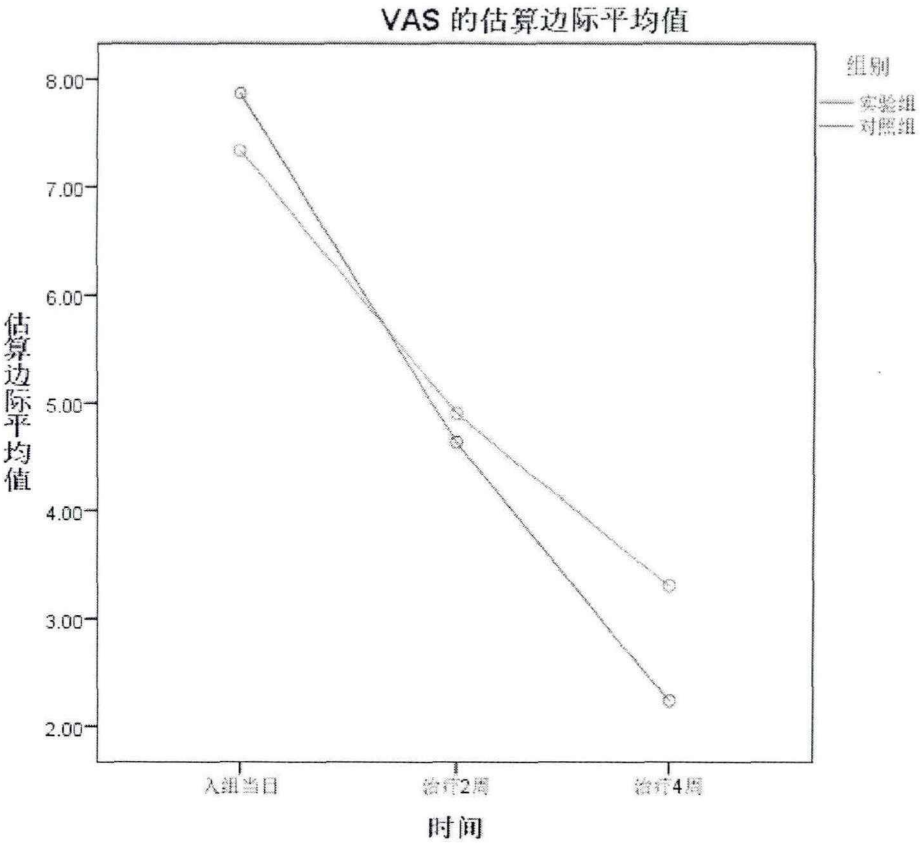

图 8 VAS 评分与时间的交互图

3.2.2 试验组和对照组不同时间点 AOFAS 评分

采用 P-P 图检验 AOFAS 评分是否符合正态分布，结果显示，AOFAS 评分变量的各点基本呈一条直线，符合正态分布，具体见图 9。Mauchly 球形检验显示 AOFAS 评分不符合球形数据要求（χ^2=29.113, P=0.000），故读取 Greenhouse-Geisser 的校正结果。结果显示，时间因素与分组因素之间存在交互效应（F=10.059，P=0.001），表示时间因素的作用随分组（即对照组和试验组）的不同而不同。交互图如图 10。两组患者 AOFAS 评分均呈上升趋势，但试验组随时间上升趋势更明显。

对各时间点的数据进行组间比较，检验固定时间因素于某一水平时两组研究对象AOFAS评分的差异，采用两独立样本t检验进行两两比较。调整的名义检验水平为0.05/5=0.01，需将两两比较所得P值与 0.01比较，从而得出统计结论。各测量时间点AOFAS评分的组间比较结果如表5所示，入组当日两组得分差异无统计学意义（P=0.718＞0.01）,治疗2周后试验组AOFAS评分高于对照组，差异有统计学意义（P＜0.05），治疗4周后试验组AOFAS评分高于对照组，差异有统计学意义（P=0.000）。

中国知网 https://www.cnki.net

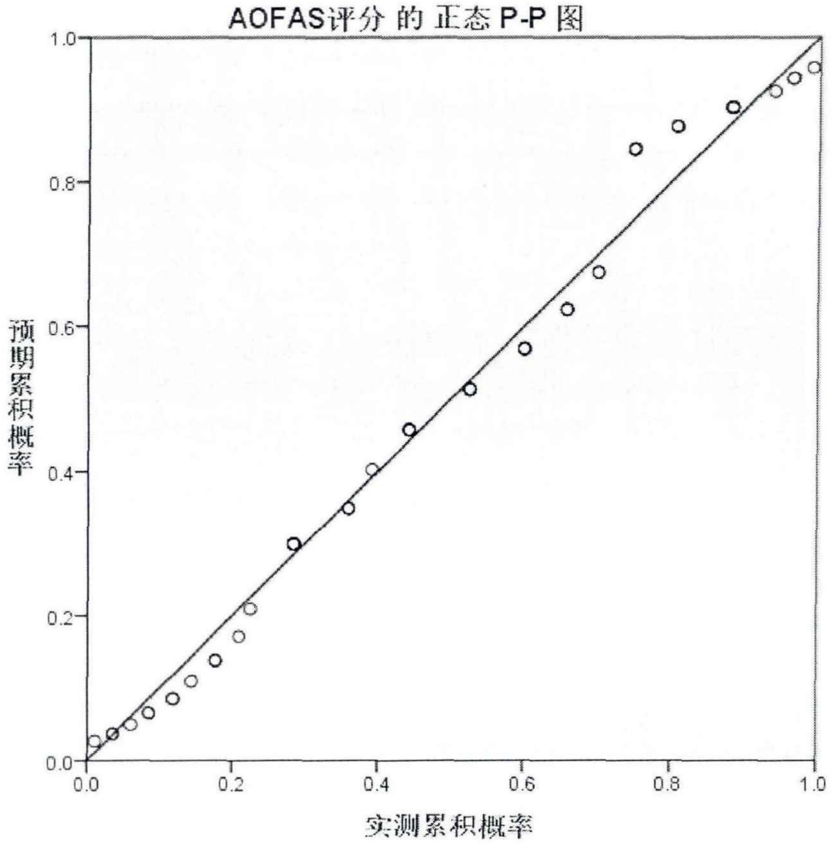

图9 AOFAS得分变量P-P图

表 5 试验组和对照组不同时间点 AOFAS 评分比较

组别	时间点			合计	F	P
	治疗前	治疗 2 周	治疗 4 周			
试验组	60.87±5.90	77.80±8.37	92.60±5.20	77.09±14.51	336.042[a]	0.000[a]
对照组	61.57±8.75	72.37±9.76	82.93±5.30	72.29±11.85	26.099[a]	0.000[a]
合计	61.22±7.41	75.08±9.42	87.77±7.13	74.69±13.47	523.486[b]	0.000[b]
F	-0.363	2.314	7.130	8.261[b]	20.162[c]	
P	0.718	0.024	0.000	0.006[b]	0.000[c]	

注：a 时间因素重复测量方差分析的 F 统计量和 P 值

b 主效应的 F 统计量和 P 值

c 交互效应的 F 统计量和 P 值

中国知网 https://www.cnki.net

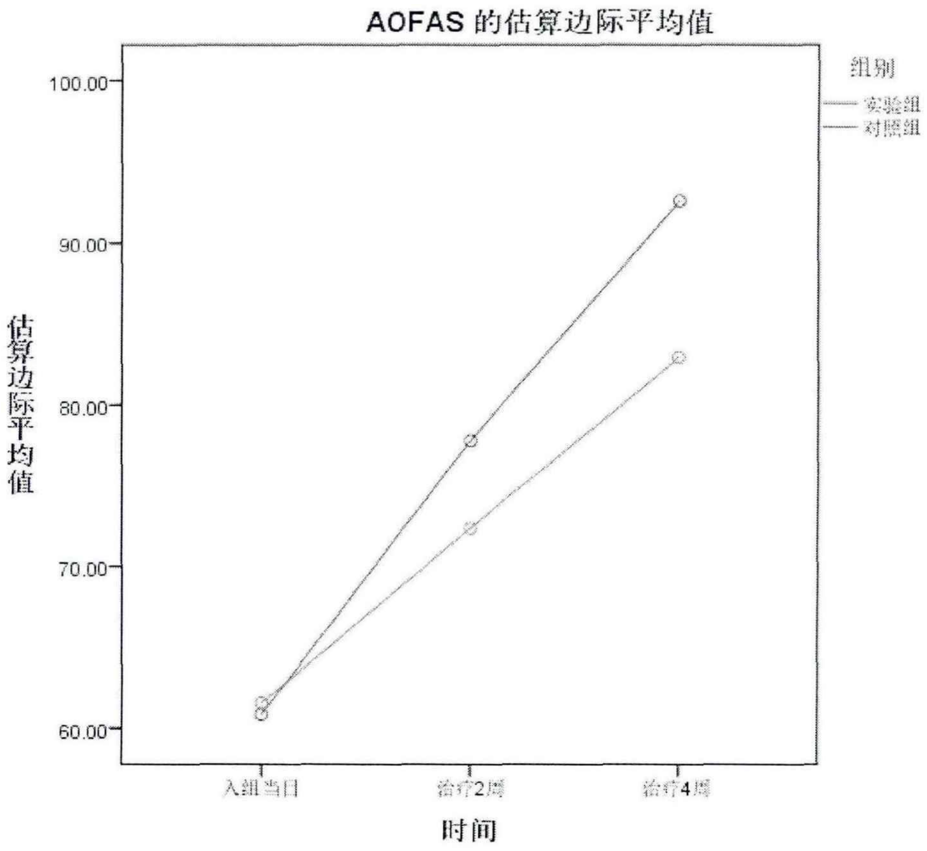

图 10 AOFAS 评分与时间的交互图

3.2.3 试验组和对照组治疗前后距骨倾斜度比较

采用P-P图检验距骨倾斜度是否符合正态分布，结果显示，距骨倾斜度变量的各点基本呈一条直线，符合正态分布，具体见图11。治疗前两组患者距骨倾斜度差异无统计学意义（P>0.05），具有可比性；试验组与对照组分别进行组内治疗前后比较，结果显示，治疗后试验组与对照组距骨倾斜度与治疗前对比，差异没有统计学意义（P>0.05）；治疗结束后试验组与对照组距骨倾斜度组间无明显差异（P>0.05），详见表6。

中国知网 https://www.cnki.net

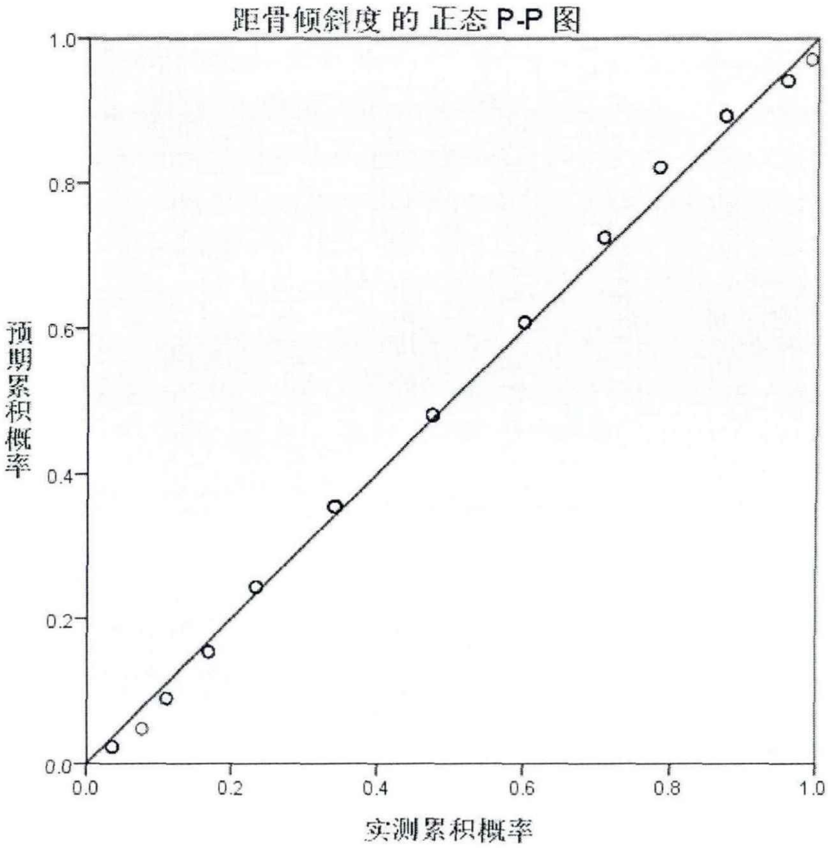

图11 距骨倾斜度变量P-P图

表 6 试验组和对照组治疗前后距骨倾斜度比较

组别	治疗前	治疗后	*t*	*P*
试验组（n=30）	6.13±3.31	6.43±2.98	-1.201	0.240
对照组（n=30）	6.17±2.91	6.50±2.27	-1.011	0.362
t	-0.041	-0.097		
P	0.967	0.923		

注：$^*P<0.05$

3.2.4 试验组和对照组治疗前后最大步行距离比较

采用P-P图检验最大步行距离是否符合正态分布，结果显示，最大步行距离变量的各点基本呈一条直线，符合正态分布，具体见图12。治疗前两组患者最大步行距离差异无统计学意义（$P>0.05$），具有可比性；试验组与对照组分别进行组内治疗前后比较，结果显示，治疗结束后试验组与对照组最大步行距离与治疗前对比，差异没有统计学意义（$P>0.05$）；治疗结束后试验组与对照组最大步行距离组间无明显差异，详见表7。

中国知网 https://www.cnki.net

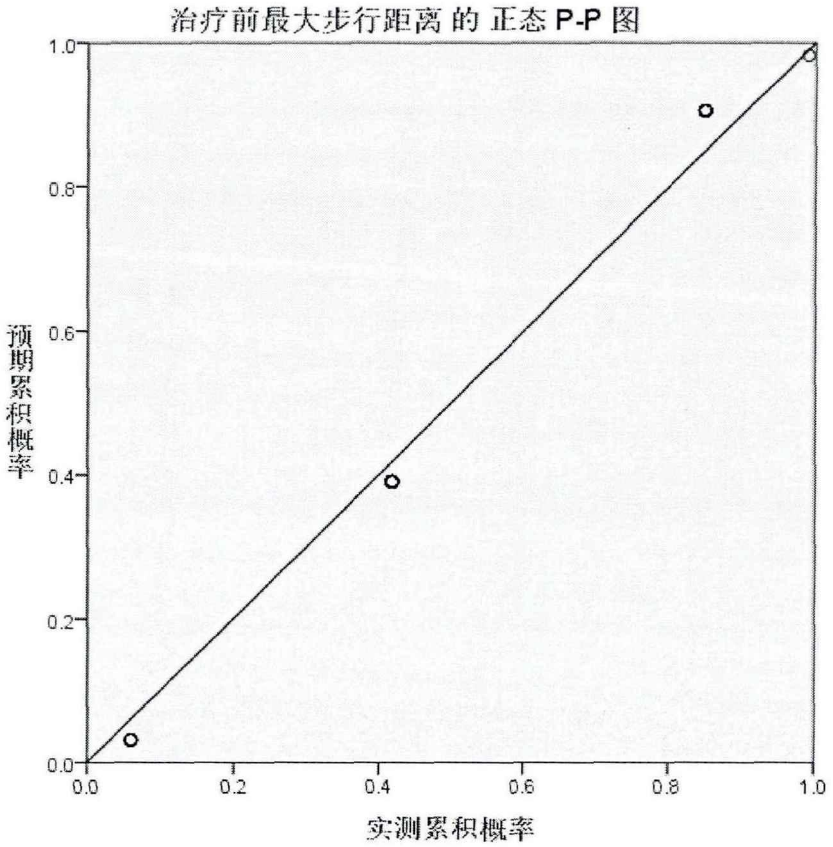

图12 最大步行距离变量P-P图

表 7 试验组和对照组治疗前后最大步行距离比较

组别	治疗前	治疗后	*t*	*P*
试验组（n=30）	2.07±1.23	4.60±0.50	-11.082	0.000*
对照组（n=30）	2.63±1.25	4.33±0.66	-7.899	0.000*
t	-1.773	1.765		
P	0.081	0.083		

注：*P<0.05

3.2.5 试验组和对照组治疗 2 周与治疗 4 周后改善率比较

采用P-P图检验改善率是否符合正态分布，结果显示，改善率的各点基本呈一条直线，符合正态分布，具体见图13。比较两组患者治疗2周和治疗4周后的改善率，结果发现，治疗2周，试验组的改善率明显高于对照组，治疗4周后也是试验组改善率更高，差异均具有统计学意义。对比治疗2周和治疗4周后试验组和对照组组内变化，结果显示，无论是试验组还是对照组，治疗4周后的疗效指数均明显高于治疗2周后的改善率，详见表8。

中国知网 https://www.cnki.net

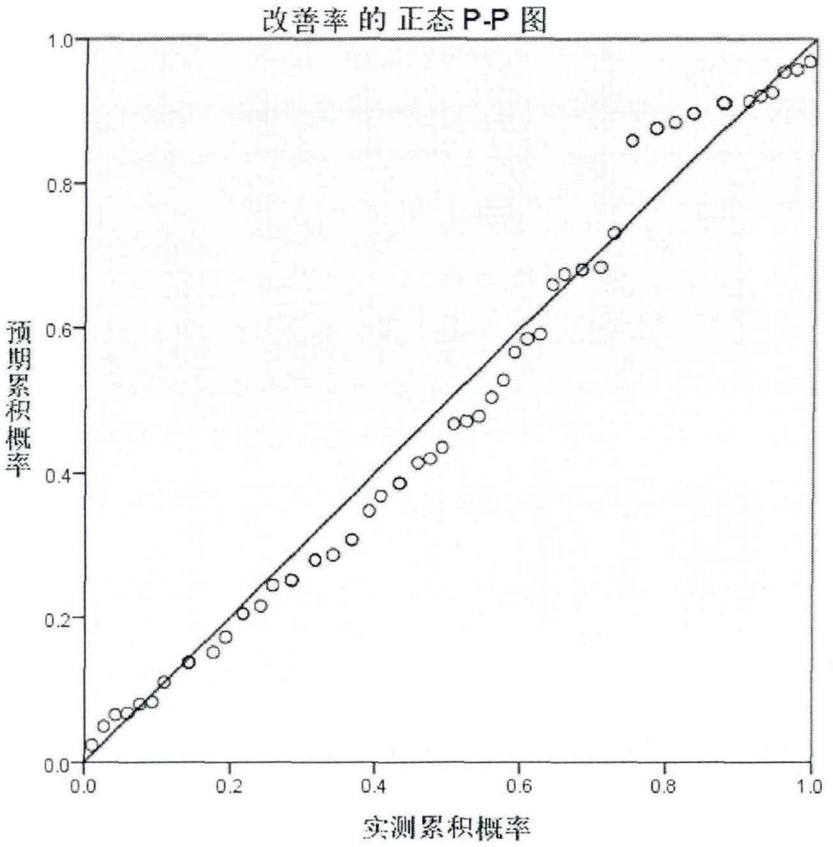

图13 改善率变量P-P图

表 8 试验组和对照组治疗 2 周与治疗 4 周后改善率比较

组别	治疗 2 周	治疗 4 周	t	P
试验组（n=30）	43.80±18.27	81.45±12.50	-13.763	0.000*
对照组（n=30）	29.47±17.08	55.55±9.95	-11.736	0.000*
t	3.139	8.878		
P	0.003*	0.000*		

注：*P<0.05

3.3 安全性分析

两组受试者在试验阶段均未出现不适，生命体征未出现较大波动、未报告局部过敏反应、疼痛加重或全身性反应等不良反应。

中国知网 https://www.cnki.net

第四章　讨论分析

4.1 中医手法治疗陈旧性踝关节扭伤的中医基础理论

手法是中医治疗陈旧性踝关节扭伤最常用方法之一，因其无创、疗效显著而受到患者青睐。在传统医学上，大多数学者认为陈旧性踝关节扭伤多因踝关节扭伤后局部筋脉损伤，血溢脉外，气滞血瘀，经脉不通，不通则痛。在中医手法治疗中，能通过按、揉法调和气血、促进血肿吸收，弹拨手法能够松筋散结、解筋通脉、松解局部粘连的软组织，拔伸手法能促进踝关节正常的解剖结构的恢复，牵张手法能扩大关节间隙，从而改善踝关节功能[56]。另外，"骨错缝，筋出槽"理论[57]也广为骨伤科医师所接受。在踝关节扭伤中，小关节在外力的作用下出现了微细的移位，即"骨错缝"，这种细微的移位并不一定能在影像学检查中显示出来，而在复杂的踝关节结构当中，这种微细的移位常常导致踝关节活动时疼痛、关节活动度减小等症状频发。急性的踝关节扭伤后，踝关节周围的韧带、肌腱、关节囊出现不同程度的损伤，导致撕裂的韧带、肌腱、关节囊在松弛位愈合、粘连变性，使得断裂的韧带、肌腱收缩、失去弹性，即"筋出槽"，筋出槽后不能维持踝关节的正常解剖结构，"筋束骨而利关节"，筋无以束骨时，关节活动异常，常常出现关节活动度下降或超过正常生理活动度。"筋骨并重"的理念使得中医手法通过缩筋回槽、敛气入骨，在治疗陈旧性踝关节扭伤中取得良好的效果。然而，中医手法治疗存在一定局限性。一方面，中医手法治疗并不能治疗所有的"筋出槽"，对于韧带、肌腱的断裂，出现踝关节机械性不稳定时，中医手法疗效不佳，因已经断裂的韧带无法修复，无法恢复踝关节正常生理功能，常常需要外科手术治疗；另一方面，中医手法对操作者要求较高，需要操作者对手法治疗部位具有良好的解剖结构知识储备，同时对运用的手法治疗具有较高的熟悉程度，从而在治疗中达到事半功倍的效果。

4.2 清宫外踝理筋手法治疗陈旧性踝关节扭伤的特色

在陈旧性踝关节扭伤的手法治疗中，清宫外踝理筋手法主张在轻揉"筋结"的同时运用独特的"摇、拔、戳"手法进行治疗。对于陈旧性踝关节扭伤，因疼痛、粘连等因素导致的踝关节周围肌腱、韧带紧张，踝关节活动受到限制，清宫外踝理筋手法中的"摇"法能在无痛或少痛的情况下放松踝关节，"拔"法与"戳"法的结合，"欲合先离,离而复合"，从而解除踝关节交锁，纠正"骨错缝"，同时使"筋回槽"，恢复踝关节正常解剖结构。陈兆军[58]、李永恒[59]、付文博[39]运用清宫外踝理筋手法治疗陈旧性踝关节扭伤均获得了良好的效果，有效减轻踝关节疼痛，提高踝关节功能。

秉承"机触于外、巧生于内、手随心转、法从手出"的正骨手法要旨，手法运用上讲求轻柔和缓、外柔内刚。使患者在没有痛苦感觉或尽可能减轻疼痛的情况下缓解乃至治愈疾病。轻：主要指动作要轻，使患者在心理上易于接受，同时过重的手法易加重损伤以及患者疼痛感，不利于康复。巧：巧妙，指手法技巧以及运用手法治疗时的

中国知网 https://www.cnki.net

“巧劲”，如此才能有效地治疗疾病，达到事半功倍的效果。柔：是手法用力要柔和，不能粗暴、生硬，强调刚柔相济。运用手法的力量要根据患者病情、患者接受程度，并结合医生自身功力运用。对新伤用力要轻，动作要缓，幅度要小，而陈旧伤则可随着治疗的进程徐徐用力，使患者感到患处有沉重感或酸痛，但能忍受即可。和：就是心、手相合。医者用手触摸，了解病患损伤的具体情况，并用“心”体会，用“心”指导双手实施手法达到治疗的目的。筋伤手法不是简单重复的机械运动，而是在“心”的指导下通过双手在患者无痛或者少痛的情况下纠正“骨错缝，筋出槽”，从而达到改善、恢复踝关节功能的效果。

4.3 双氯芬酸二乙胺乳胶剂药理作用及优势

双氯芬酸NSAIDs中具代表性的一类药物，目前临床应用的双氯芬酸钠，其作用机制是通过选择性阻断花生四烯酸代谢中环氧合酶的作用，阻断前列腺素(PGs)的合成。在消炎、镇痛、抗风湿方面效果较好，能够代替吗啡类镇痛药物，其药理学特点鲜明，药代动力学特征显著。双氯芬酸是非甾体类抗炎药中具有抗炎止痛的主要品种，较其他三环类非甾体抗炎药的效果更强，具有良好的安全性与耐受性，是应用最广泛的非甾体药物之一[60]。它具有以下临床应用特点[61]：①药物效力强：在抗炎、镇痛及解热方面，其作用效果是阿司匹林的26~50倍；②分布快：口服双氯芬酸后，机体即可将药物迅速吸收，服药后的1~2h血药浓度即可达到峰值；③体内消除快④使用剂量小：双氯芬酸的服用量为每日三次，每次25mg。将使用方法改成肌注后只需每日一次，一次75mg即可。然而，口服剂型容易刺激胃粘膜，服用后不良反应发生率高达19.3%，然而，将双氯芬酸制成乳胶剂后可有利于患者接受，同时能减少对皮肤正常功能的影响，易于用水洗，对骨关节炎疗效明显，不良反应较少，

4.4 基于本研究分析清宫手法治疗对陈旧性踝关节扭伤患者 VAS 评分的影响

清宫手法以“手摸心会”、“轻巧柔和”[64]为核心理念，常于谈笑间将病治好，恰如《刘寿山正骨经验集》 所言：“法之所施，使患者不知其苦，方称为手法也”，同时结合“筋喜柔不喜刚”的生理特点，清宫手法在运用上强调轻柔绵软、外柔内刚，将患者痛苦降到最低的同时达到治疗的作用。根据本研究结果，治疗前两组 VAS 评分无明显差异（P=0.062＞0.01），排除时间对研究结果的影响，治疗 4 周后试验组 VAS 得分低于对照组，差异有统计学意义（P=0.000）。这与翟东旺研究[65]结果一致，翟东旺将 84 例陈旧性踝关节扭伤患者分为观察组和对照组，对照组使用中药熏洗治疗，观察组在对照组基础上采用外踝理筋手法治疗，治疗 2 周后，观察组 VAS 评分明显低于对照组。清宫手法在治疗陈旧性踝关节扭伤中，通过手法的治疗可以改善踝关节局部的血液循环和淋巴回流，消除肿胀，与双氯芬酸二乙胺乳胶剂联用减轻踝关节局部炎症反应，从而减轻疼痛。

中国知网 https://www.cnki.net

4.5 清宫手法治疗对陈旧性踝关节扭伤患者 AOFAS 踝-后足评分系统评分的影响

清宫手法在尽可能降低患者痛苦的情况下达到治疗的效果，同时手法的力量由轻渐重，由外而内，阴柔之力与刚强之力相结合，舒筋活动、滑利关节，能有效改善踝关节功能。根据本研究结果，治疗前两组 AOFAS 差异无统计学意义（P=0.718＞0.01），排除时间变量对研究结果的影响，治疗 4 周后 试验组 AOFAS 评分高于对照组，差异有统计学意义（P=0.000），此外，根据 AOFAS 踝-后足评分系统评分进行改善率比较，治疗 2 周及治疗 4 周后试验组的改善率均高于对照组（P＜0.05，P＜0.05）。这与陈兆军研究[58]结果一致，陈兆军对内翻损伤型的陈旧性踝关节扭伤患者进行外踝理筋手法治疗和功能锻炼治疗对比，结果提示两组患者治疗后 AOFAS 评分均较治疗前提高（P＜0.05，P＜0.01），治疗后外踝理筋手法治疗组 AOFAS 评分高于对照组（P＜0.05）。踝部扭伤后，局部筋骨关节、脉络受损，必累及气血，气滞血瘀，为肿为痛，从而影响肢体关节的活动。《医宗余鉴·正骨心法要旨》中指出："因跌仆闪失，以致骨缝开错，气血郁滞，为肿为痛，宜用按摩法。按其经络，以通郁闭之气，摩其壅聚，以散瘀结之肿，其患可愈。"说明手法具有理筋整复、滑利关节的作用，这表现在三个方面：一是手法作用于踝关节损伤局部，促进气血运行，消肿祛瘀，理血止痛；二是手法可以通过力学的直接作用来纠正筋出槽、骨错缝，达到理筋整复的目的；三是轻巧柔和的被动运动手法可以起到松解粘连、滑利关节的作用。

4.6 清宫手法治疗对陈旧性踝关节扭伤患者距骨倾斜度的影响

在中医"筋骨并重"的中心思想指导下，清宫手法在对陈旧性踝关节扭伤的治疗中，对踝关节功能性不稳具有良好的疗效，但是对机械性不稳疗效不明显[64]。本研究通过测量两组治疗前后的应力位距骨倾斜度，结果发现，组内比较中试验组和对照组的距骨倾斜度在治疗前后并无显著改变，组间比较也没有统计学差异，因此考虑两组治疗均不能改变踝关节的解剖关系。这与李永恒的研究[59]结果一致，同时还指出，清宫手法治疗陈旧性踝关节扭伤过程中，患者症状的改善多是改善了踝关节功能性不稳定，而并没有改善机械性不稳定，当踝关节韧带断裂、踝关节骨折等机械性因素导致踝关节疼痛、活动受限时，应选择手术治疗。

4.7 清宫手法治疗对陈旧性踝关节扭伤患者最大步行距离的影响

在 AOFAS 踝关节功能评分中，抽取最大步行距离子项数据进行分析，结果显示，治疗结束后两组最大步行距离与治疗前对比具有统计学意义差异（P＜0.05）；而试验组与对照组分别进行组间治疗前后比较结果没有统计学差异（P＞0.05）。由此可见，试验组和对照组治疗陈旧性踝关节扭伤均能提高患者最大步行距离，且提高程度没有统计学差异，考虑以下因素：1.纳入病人数量偏少，不能很好比较出两组患者最大步行距离改善程度的差异；2.AOFAS 踝关节功能评分中最大步行距离分数占比较低

中国知网 https://www.cnki.net

（5 分），使用该评分进行步行功能评价时难以分析出两组差距；3.双氯芬酸二乙胺乳胶剂的止痛效果的干扰，这需要空白对照进行排除，4.试验观察期较短，试验组与对照组长期疗效。

4.8 研究的不足及展望

1. 本研究使用了未使用盲法，且由于时间和经费问题，所纳入得病例数偏少，可能影响本研究的客观性和准确性，还需高质量的大样本量临床研究；

2. 本研究仅仅对比了一些早期疗效指标，治疗前、治疗 2 周、治疗 4 周后的 VAS 评分、AOFAS 评分，治疗前、治疗 4 周后的内翻应力位距骨倾斜度测量；对于清宫手法联合双氯芬酸二乙胺乳胶剂外涂、功能锻炼治疗的中长期疗效尚不明确，还需进一步做到长期密切随访观察；

3. 临床观察的客观指标不足，未来研究中应考虑增加必要的影像学检查以及相关量表进行评估，以提高研究的准确性。

中国知网 https://www.cnki.net

结　语

由于踝关节扭伤的高发性与普遍性而常常不被重视，错失早期治疗的机会，导致后期陈旧性踝关节扭伤，慢性的踝关节疼痛、活动受限、乏力感等症状严重影响患者生活质量，大大增加患者治疗费用及治疗周期，对许多骨科医师来说是一种巨大的挑战。

清宫手法治疗陈旧性踝关节扭伤中，具有安全、无创、有效的特点，结合双氯芬酸二乙胺乳胶剂外涂，能有效减轻踝关节疼痛，并配合功能锻炼康复，“动静结合”，总体上认为有益于陈旧性踝关节扭伤患者踝关节功能的康复，值得临床推广。

中国知网　https://www.cnki.net

参考文献

[1]马德刚,赵秀华.中药熏洗配合手法治疗陈旧性踝关节扭伤 55 例报告[J].齐齐哈尔医学院学报,2008(11):1346.

[2]刘畅,张海森,裴宝静,苏珂,王庆海,白玉明,李龙杰.跟骨前突置钉外固定架治疗踝关节骨折的临床疗效分析[J].中国医药导报,2017,14(27):105-108.

[3]Waterman BR, Owens BD, Davey S, Zacchilli MA, Belmont PJ Jr. The epidemiology of ankle sprains in the United States. *J Bone Joint Surg Am*. 2010;92(13):2279-2284.

[4]李永恒,陈兆军.踝关节陈旧性损伤的诊断和治疗进展[J].中华骨与关节外科杂志,2017,10(02):173-177.

[5]裴子文,孟宪梅,杨建强,陈建.慢性踝关节不稳患者下肢肌肉激活特征研究现状[J].中国康复理论与实践,2018,24(06):678-681.

[6]段圆慧,田孟强,孙云波,张东亮,田峥巍,孙和军,王琦.关节镜手术治疗踝关节前方撞击综合征的疗效观察[J].中国微创外科杂志,2017,17(06):534-537.

[7]敬沛嘉,李箭.慢性踝关节不稳的临床治疗新进展[J].华西医学,2017,32(12):1939-1942.

[8]王琳珏,郑昆仑,金鸿宾,王爱国.神效散联合理筋手法治疗慢性踝关节不稳的临床疗效观察[J].中国中西医结合外科杂志,2018,24(01):43-46.

[9]华英汇,陈世益.慢性踝关节不稳定的外科治疗进展[J].中国医学前沿杂志(电子版),2013,5 (03):8-11.

[10]Melanie A. Hopper,Philip Robinson. Ankle Impingement Syndromes[J]. Radiologic Clinics of North America,2008,46(6).

[11]Jackson W , Mcgarvey W . Update on the treatment of chronic ankle instability and syndesmotic injuries[J]. Current Opinion in Orthopaedics, 2006, 17(2):97-102.

[12]Meacham BP, Granata JD, Berlet GC. Tenodesis reconstruction for chronic ankle instability: graft considerations and structures at risk with tunnel placement. *Foot Ankle Spec*. 2012;5(6):378-381.

[13]徐欣, 姜劲挺, 王强强,等. 中医骨伤科对慢性踝关节不稳定的认识及治疗进展[J]. 中国医药科学, 2019, 009(019):55-58.

[14]施晓剑,韩甲,刘宇,王雪强,陈佩杰.慢性踝关节不稳的病理机制和评估诊断研究进展[J].中国运动医学杂志,2019,38(09):816-824.

[15]于涛,俞光荣.踝关节外侧不稳定的生物力学研究进展[J].中国骨与关节损伤杂志,2010,25(01):94-96.

[16]许灿. 人踝关节外侧韧带损伤和慢性不稳定重建生物力学的三维有限元分析[D].中南大学,2012.

[17]陈明亮,谷成毅,徐留海,周游.踝关节外侧副韧带损伤诊断治疗研究进展[J].中国运动医学杂志,2019,38(02):152-158.

[18]Jarde O , Havet E , Gabrion A , et al. [Long-term outcome following surgical repair of ruptures of the fibular collateral ligament of the ankle. A report of 50 cases][J]. Acta Orthopaedica Belgica, 1999, 65(3):340.

[19]吴金龙,陆阿明.不同类型踝关节护具对功能性踝关节不稳者静态姿势稳定性的影响[J].中国运动医学杂志,2017,36(03):232-235.

[20]张阳,张秋霞.功能性踝关节不稳者的静态平衡能力[J].中国组织工程研究,2013,17(35):6287-629

中国知网 https://www.cnki.net

2.
[21]于惠贤,杨纯生,张冉,胡志伟,何蕾,陈亚平.完善慢性功能性关节不稳定康复疗效评估体系[J].解剖学报,2017,48(02):165-169.
[22]Kim K J , Kim Y E , Jun H J , et al. Which Treatment is More Effective for Functional Ankle Instability: Strengthening or Combined Muscle Strengthening and Proprioceptive Exercises?[J]. Journal of Physical Therapy Science, 2014, 26(3):385-388.
[23]黄昀,冯强.功能性踝关节不稳与力量训练[J].广西医学,2015,37(07):960-962.
[24]吴贵根,李军,刘川,赵信用,荣红亮,朱明双,汪亚强.推拿按摩治疗踝关节损伤的研究进展[J].光明中医,2010,25(08):1536-1537.
[25]王成伟. 踝关节外侧不稳定功能重建的生物力学及临床研究[D].新疆医科大学,2018.
[26]Sammarco, James V . Complications of lateral ankle ligament reconstruction.[J]. Clinical Orthopaedics & Related Research, 2001, 391(391):123.
[27]Ng Z , De S D . Modified Brostrom-Evans-Gould technique for recurrent lateral ankle ligament instability.[J]. Journal of Orthopaedic Surgery, 2007, 15(3):306.
[28]Tohamy W E , Mahboub N E . The results of surgical treatment of chronic lateral ankle instability with the Evans technique[J]. The Egyptian Orthopaedic Journal, 2016, 51(1):54-.
[29]Diab H . Modified Evans technique for treatment of chronic lateral ankle instability[J]. The Egyptian Orthopaedic Journal, 2014, 49(2):146.
[30]王建华,佟颖彪,桑园田.采用改良 Watson-Jones 术式重建外侧副韧带治疗慢性踝关节不稳的疗效分析[J].中国医师进修杂志,2013,36(15):44-45.
[31]邱士庆,邱运芬,刘洁,刘金云.改良 Watson-Jones 方法治疗创伤性慢性踝关节外侧不稳定 26 例报告[J].实用医技杂志,2005(13):1813-1814.
[32]周建刚.改良 Chrisman-Snook 手术治疗慢性踝关节不稳定[J].现代医药卫生,2005(20):2792-2793.
[33]周一飞,卢晓郎,赖红燕,左海强,叶超,洪建军.Evans 和 Chrisman-Snook 术式治疗踝关节外侧副韧带II度损伤的生物力学比较[J].中国骨伤,2012,25(08):654-657.
[34]王国强,屈建国,渠海波,乔绍文.关节镜下 Brostr?m-Gould 术治疗慢性踝关节外侧不稳[J].中国内镜杂志,2021,27(01):28-33.
[35]陈明亮,周耀君,谷成毅,丁松,周游.开放与关节镜下改良 Brostr?m 术式治疗慢性踝关节外侧副韧带损伤的对比研究[J].中国运动医学杂志,2020,39(02):104-110.
[36]Becker H P , Rosenbaum D , Zeithammel G , et al. Tenodesis versus carbon fiber repair of ankle ligaments: a clinical comparison.[J]. Clin Orthop Relat Res, 1996, 325:194-202.
[37]陈兆军,常青,吴俊德,李永恒,张晓亮,张岩峰,潘旭月.肌骨超声观察外踝理筋手法治疗陈旧性踝关节扭伤 39 例[J].中国中医骨伤科杂志,2018,26(07):42-46.
[38]林世豪. 理筋正骨手法治疗陈旧性踝关节扭伤的临床观察[D].安徽中医药大学,2017.
[39]付文博. 摇拔戳手法治疗陈旧性踝关节扭伤的病例对照研究[D].中国中医科学院,2016.
[40]阿伍提·艾克木,李俊海,林留洋.宫廷正骨手法治疗陈旧性踝关节扭伤疗效观察[J].现代中医临床,2016,23(01):44-46.
[41]杨春花.针刺配合小针刀治疗陈旧性踝关节扭伤 30 例[J].浙江中医杂志,2012,47(03):198.
[42]王冠军.中药熏蒸配合小针刀治疗陈旧性踝关节扭伤疗效观察[J].新中医,2015,47(05):265-266.
[43]秦民安,陈建鸿. 小针刀治疗陈旧性踝关节扭伤 25 例临床体会[A]. 孙丛晓、王琦、郭闽红、夏志辉.针刀医学论文精选[C].:山西省针刀医学会,1999:2.
[44]黄伟. 小针刀联合封闭治疗陈旧性内翻型踝关节扭伤的临床疗效观察[D].福建中医药大

中国知网 https://www.cnki.net

学,2017.
[45]陈壮娜,熊峻,黄石龙.不同针灸疗法治疗陈旧性踝关节扭伤的疗效观察[J].内蒙古中医药,2020,39(02):91-92.
[46]李军.不同针灸疗法治疗陈旧性踝关节扭伤临床效果研究[J].足踝外科电子杂志,2019,6 (04):39-42.
[47]周彬,金瑛,曹莉.电针结合火针治疗陈旧性踝关节内翻扭伤的临床疗效观察[J].时珍国医国药,2018,29(01):121-122.
[48]毛庆友,徐兆勇.中西医结合治疗陈旧性踝关节扭伤的临床效果[J].中医临床研究,2019,11(32):85-87.
[49]张阳.温针结合中药熏洗治疗陈旧性踝关节扭伤 42 例疗效观察[J].中国民族民间医药,2018,27(04):79-81.
[50]薛彬,万世元,李飞跃,奚小冰.魏氏伤科法治疗陈旧性踝关节扭伤的临床分析[J].中成药,2014,36(08):1612-1615.
[51]陆亚丽,蔡水奇.超声波中药熏洗治疗陈旧性踝关节伤的疗效观察[J].浙江创伤外科,2018,23(03):468-469.
[52]余雷. 低温等离子刀成形术联合中药熏洗治疗陈旧性踝关节扭伤的临床疗效观察[D].黑龙江中医药大学,2016.
[53]王敏,卢振和,陈来,陈筱.中医药联合臭氧治疗陈旧性踝关节扭伤[J].现代中西医结合杂志,2012,21(24):2669-2671.
[54]王筱锋,朱倩.发散式冲击波治疗陈旧性踝关节扭伤 38 例临床观察[J].中国民间疗法,2015,23(12):72-73.
[55]王刚,李莉,支世宝.体外冲击波治疗末端病的研究进展[J].中国医药指南,2018,16(14):27-28.
[56]孙琦,王丹,刘海兵,傅瑞阳.复合手法结合中药熏蒸治疗陈旧性踝关节扭伤疗效观察[J].浙江中医杂志,2015,50(04):281.
[57]李俊海,王庆甫,黄沪.正骨手法与中药熏洗治疗陈旧性踝关节扭伤的病例对照研究[J].中国骨伤,2012,25(02):113-115.
[58]陈兆军. 孙树椿教授外踝理筋手法治疗陈旧性踝关节扭伤临床观察及机理初探[D].中国中医科学院,2016.
[59]李永恒. 外踝理筋手法治疗陈旧性踝关节扭伤临床疗效观察[D].北京中医药大学,2017.
[60]王兴宇. 双氯芬酸盐、双氯芬酸衍生物的合成工艺及生物活性研究[D].吉林大学,2015.
[61]王雪洁,高彩芹,曲知芳,等. 双氯芬酸的药理及临床应用[J]. 山东医药工业,2001,20(3):17-18. DOI:10.3969/j.issn.1672-7738.2001.03.019.
[62]王磊,于天源,鲁梦倩,潘璠. 推拿手法"轻巧柔和"的探讨[A]. 中华中医药学会.第十二次全国推拿学术年会暨推拿手法调治亚健康临床应用及研究进展学习班论文集[C].中华中医药学会:中华中医药学会,2011:2.
[63]翟东旺.外踝理筋手法联合中药熏洗治疗陈旧性踝关节扭伤 42 例临床观察[J].中国民族民间医药,2019,28(08):94-95.
[64]李永恒,陈兆军.踝关节陈旧性损伤的诊断和治疗进展[J].中华骨与关节外科杂志, 2017,10 (02):173-177.

中国知网 https://www.cnki.net

附　录

附 录 1：评分量表

AOFAS 踝-后足评分系统（AOFAS Ankle Hindfoot Scale）

	评分
疼痛（40 分）	
无	40
轻度，偶尔	30
中度，常见	20
严重，持续	0
功能和自主活动、支撑情况（10 分）	
不受限，不须支撑	10
日常活动不受限，娱乐活动受限，需扶手杖	7
日常和娱乐活动受限，需扶手杖	4
日常和娱乐活动严重受限，需扶车、扶拐、轮椅、支架	0
最大步行距离（街区数）（5 分）	
>6 个	5
4 ～ 6 个	4
1 ～ 3 个	2
<1 个	0
地面步行（5 分）	
任何地面无困难	5
走不平地面、楼梯、斜坡、爬梯时有困难	3
走不平地面、楼梯、斜坡、爬梯时有困难	0
反常步态（8 分）	
无、轻微	8
明显	4
显著	0
前后活动（屈曲加伸展）（8 分）	
正常或轻度受限（>30°）	8
中度受限（15°-29°）	4
重度受限（<15°）	0
后足活动（内翻加外翻）（6 分）	
正常或轻度受限（75%-100%正常）	6
中度受限（25%-74%正常）	3
重度受限（<25%）	0
踝-后足稳定性（前后，内翻-外翻）（8 分）	
稳定	8
明显的不稳定	0
足部对线（10 分）	
优：跖行足，踝-后足排列正常	10
良：跖行足，踝-后足明显排列成角，无症状	5
差：非跖行足，严重排列紊乱，有症状	0

中国知网 https://www.cnki.net

优：90 ～ 100 分；良：75 ～ 89 分；可：50 ～ 74 分；差：50 分以下

视觉模拟疼痛评分

记录治疗前、治疗 2 周、治疗 4 周的视觉模拟疼痛评分，0 分：无痛；3 分以下：有轻微的疼痛，患者能忍受；4 分-6 分：患者疼痛并影响睡眠，尚能忍受；7 分-10 分患者有渐强烈的疼痛疼痛难忍；10 分最痛。

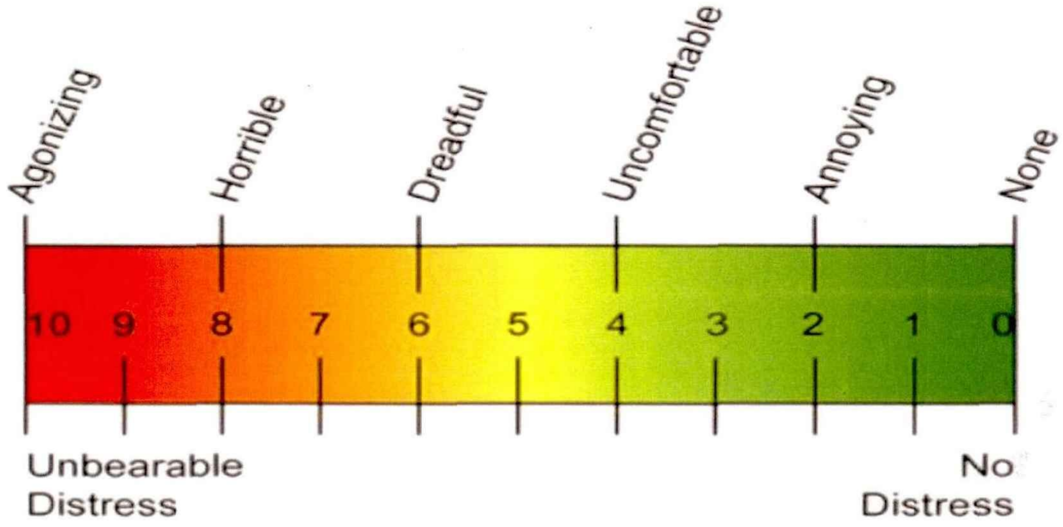

附 录 2：随机数表

24	12	18	41	7	44
19	59	15	54	28	43
57	49	20	35	4	11
48	22	13	53	52	10
16	46	56	31	2	6
17	27	38	3	39	9
21	30	32	37	40	42
55	58	14	25	26	29
33	36	47	51	5	8
23	45	50	34	60	1

附 录 3：

缩略语	英文全称	中文全称
AOFAS	Ankle Hindfoot Scale	踝-后足评分系统
VAS	visual analogy score	视觉模拟疼痛评分
CAI	Chronic Ankle Instability	慢性踝关节不稳
FAI	Functional Ankle Instability	功能性踝关节不稳
MAI	Mechanical Ankle Instability	机械性踝关节不稳

中国知网 https://www.cnki.net

致　谢

五年的本科加上三年的硕士研究生学习已然接近尾声，自己已不再是当初的青葱懵懂，脸上褪去了幼稚，走向了成熟，也走向了社会。回想八年的学习生活，总是感慨万千，也感谢相遇。

首先，感谢敬爱的父母，感谢父母给予我生命并抚养长大；感谢父母的支持，感谢父母提供了二十多年的物质生活保障和优秀的学习生活环境；感谢父母的包容，包容我的任性和不成熟，为了让我以后的人生更顺利，少走弯路，真的很感谢你们！

然后要感谢尊敬的陈海云老师，感谢陈海云老师的传道授业，还有解惑，老师的治学严谨、对患者的耐心和负责的态度、对手术操作的严格要求，都深深地影响着我，老师与我们亦师亦友的师生关系，让我们能与老师更好地交流与学习。同时，本研究也是在陈海云老师的悉心指导下完成课题的选题、实施、论文的书写和修改，给我提供了很多宝贵的意见和建议，感谢与陈海云老师的相遇！

接着，要感谢老年骨折科的喻秀兵老师、陈平老师、王海洲老师、杨文斌老师、杨冰师兄，创伤足踝科的谢杰伟老师、管华老师、李晓初老师、刘毅老师、黄伟明老师，大学城骨科吴江林老师、梁以豪师兄等的谆谆教诲，让我学会了很多临床知识与技能操作。

再要感谢同门冯恩辉师兄、何倩伟师兄、魏力师兄、吕阳师兄、李希文师兄、高世华师兄、何祥忠、王养发、管建豪、周冠斌、林梓涵、陈家镇等的照顾与学习，感谢女友、舍友和朋友们的支持，还有临床轮科遇到的师兄、师姐、师弟、师妹给予的帮助，让我学习到很多为人处世的道理，顺利完成硕士研究生学业。

如今，硕士研究生的学习即将迎来结束，但知识学习的历程仍在继续，我将怀揣着自己的梦想，不忘初心，继续努力学习，在未来的工作学习中再创佳绩！

中国知网 https://www.cnki.net

统计学审核证明

学号：20187102100

广州中医药大学研究生学位论文统计学审核证明

兹有 硕士研究生 李慧文 （导师 陈海云 ）的学位论文《清宫手法治疗陈旧性踝关节扭伤的疗效观察》中有关统计学方面的内容，经我部门审定合格，特此证明。

广州中医药大学华南针灸研究中心

负责人：[signature]

2021 年 4 月 5 日

中国知网 https://www.cnki.net
