# Supplementary material for: Efficacy and safety of Tuina (Chinese Therapeutic Massage) for chronic ankle instability: A systematic review and meta-analysis of randomized controlled trials
Source: PLoS One. 2025 Jun 6;20(6):e0321771. doi: 10.1371/journal.pone.0321771 (PMC12143534; doi:10.1371/journal.pone.0321771)
Supplement: S2 File — (ZIP) [file pone.0321771.s004.zip › 11.理筋正骨手法治疗陈旧性踝关节扭伤的临床观察_林世豪.pdf]

单位代码：10369

学　　号：2015212212005

安徽中医药大学

2017 届外籍硕士研究生学位论文

理筋正骨手法治疗陈旧性踝关节扭伤的临床观察

CLINICAL OBSERVATION ON MANIPULATION OF REGULATING JINGJIN IN THE TREATMENT OF THE OLD ANKLE SPRAIN

学科专业：中医骨伤科学

研究方向：中医药防治筋伤疾病

导　　师：陈朝晖 副教授

硕 士 生：林世豪

论文完成单位：安徽中医药大学

2017 年 6 月·合肥

中国知网 https://www.cnki.net

密　　级：

学　　号：2015212212005

安徽中医药大学

2017 届硕士研究生学位论文

理筋正骨手法治疗
陈旧性踝关节扭伤的临床观察

CLINICAL OBSERVATION ON MANIPULATION OF REGULATING JINGJIN IN THE TREATMENT OF THE OLD ANKLE SPRAIN

作者姓名：林世豪　　申请学位级别：硕士
指导教师姓名：陈朝晖　　职　　称：副教授
学科专业：中医骨伤科学　　研究方向：中医药防治筋伤疾病
学习时间：自 2015 年 10 月　起至 2017 年 6 月止
论文提交日期：2017.06　　论文答辩日期：2017.05.19
学位授予单位：安徽中医药大学　学位类型：医学硕士

中国知网　https://www.cnki.net

中国知网 https://www.cnki.net

目　录

中文摘要……1
英文摘要……3
正文
前言……4
1 临床研究……6
1.1 临床资料……6
1.1.1 受试者来源……6
1.1.2 中医诊断标准……6
1.1.3 纳入标准……6
1.1.4 排除标准……7
1.1.5 病例剔除与脱落……7
1.2 治疗方案……7
1.2.1 治疗组……7
1.2.2 对照组……8
1.2.3 观察指标……8
1.2.4 疗效评定标准……8
1.3 统计学分析……8
2 治疗结果分析……9
2.1 两组临床疗效比较况……9
2.2 两组患者治疗前后压痛程度比较……9
2.3 两组患者治疗前后症状评分比较……10
2.4 不良反应……11
3 讨论……11
3.1 陈旧性踝关节扭伤的解剖探究及病理变化……11
3.2 西医学对陈旧性踝关节扭伤的认识……14
3.3 中医学对陈旧性踝关节扭伤的认识……19
3.4 理筋正骨手法机制……22

中国知网 https://www.cnki.net

3.5 本实验不足之处..24
4 结论..24
参考文献..25
附录..28
综述..30
个人简介..36
致谢..37

中国知网 https://www.cnki.net

中文摘要

1 目的

应用理筋正骨手法治疗陈旧性踝关节扭伤，观察理筋正骨手法的临床疗效，为理筋正骨手法治疗陈旧性踝关节扭伤提供临床依据。

2 方法

本研究的研究对象是从马来西亚当地医院选取的陈旧性踝关节扭伤患者，共80人，符合我们的纳入标准。将这80位患者按照随机化原则分组，分为治疗组和对照组各40例，治疗组采用理筋正骨手法治疗，对照组采用特定电磁波治疗器（TDP灯）照射治疗。两组患者每周治疗3次，7次为1个疗程，共治疗2个疗程。于治疗前后采用压痛仪评估两组患者疼痛程度，并参考Biard-Jackson踝关节评分标准对患者的疼痛、关节活动度、关节稳定性、行走能力、工作能力和放射学结果进行评分，并观察临床疗效。

3 结果

（1）两组疗效性观察：治疗组总有效率95%，对照组总有效率37.5%，两组比较差异有统计学意义（P=0.000）；

（2)治疗后两组患者压痛程度和Biard-Jackson踝关节评分标准较本组治疗前明显改善（P<0.05），且治疗组以上各评价指标均明显优于对照组（P<0.05）。

4 结论

理筋正骨手法治疗陈旧性踝关节扭伤可明显减轻患者局部疼痛，临床效果明显。在治疗过程中，操作简单，患者痛苦少，容易被接纳，值得在临床推广。

关键词： 陈旧性踝关节扭伤；理筋正骨手法；特定电磁波治疗仪；

中国知网 https://www.cnki.net

Clinical Observation on Manipulation of Regulating Jingjin in the Treatment of the chronic ankle sprain

Abstract

1 Objective

To evaluate the clinical efficacy of manipulation of regulating Jingjin-(soft tissue) for the treatment of chronic ankle sprain.

2 Methods

This study will be included in the diagnostic criteria of 80 patients with the old ankle sprain patients were randomly divided into treatment group and the control group of 40 cases， the treatment group was treated with manipulation of regulating Jingjin， the control group was treated with TDP lamp irradiation treatment， the two groups lasted for30 minutes each time， 3 times treatment a week， 7 times for a course of treatment， 2 courses， respectively. The pain degree of the two groups was evaluated by using pressure instruments before and after treatment; The pain degree， joint activity， joint stability， walking ability， working ability and radiological results were scored by the Biard-Jackson ankle scoring criteria， and observe the clinical efficacy.

3 Results

(1) The efficacy of the two groups: the total effective rate of 95% in the treatment group， the total effective rate of 37.5% in the control group， the difference between the two groups was statistically significant， and the evaluation indexes of the treatment group were significantly better than those of the control group (X^2=29.574， P =0.000).

(2) After treatment， the degree of tenderness and Biard-Jackson ankle score were significantly improved (P <0.05)， and the treatment group were significantly better than those of the control group(P <0.05) .

4 Conclusion

中国知网 https://www.cnki.net

Manipulation of Regulating Jingjin treatment of old ankle sprain can significantly reduce the local pain， improve clinical efficacy. And in the course of treatment， the treatment is simple， the patient less pain， easy to be accepted， it is worth in the clinical promotion.

Key words：old ankle sprain; Manipulation of regulating Jingjin(soft tissue)

中国知网 https://www.cnki.net

前言

踝关节扭伤是最为常见的软组织损伤疾病之一，在生活中尤其在各种较为激烈的运动中发生率高达全身关节损伤的80%[1]。急性踝关节扭伤发病原因多与急性运动损伤有关，常由于下楼踏空楼梯，篮球、排球、足球、现代舞、芭蕾舞等运动中跳起落地不稳或脚被踩绊等引起足内翻、内旋或过度的外翻、外旋，导致踝关节外侧或内侧韧带损伤[2]。

踝关节是十分稳固的屈戌关节，其静态稳定性的很大来源是关节周围韧带，比如内侧的副韧带，外侧的副韧带，连接胫腓骨远端的下胫腓韧带。内侧副韧带是踝关节周围最坚强的韧带，其像一个扇形连接着足骨和胫骨，且和踝关节囊紧密结合在一起，它的作用是防止踝关节在外翻时产生过度运动造成损伤。与此相对应的是更为薄弱的外侧副韧带，也更容易在暴力下遭受损害。踝关节的扭伤包括两种情况，一是在足关节内翻时扭伤，而是在外翻情况下遭受损害。距腓前韧带、跟腓韧带、距腓后韧带以及下胫腓韧带在第一种情况下会出现损伤，三角韧带损伤常见于后者，临床上三角韧带损伤不多见，更为普遍的是坚韧的三角韧带牵拉附着的骨面造成骨或者软骨的撕裂性伤害，甚则严重者会造成韧带断裂。

踝关节的结构与功能特点导致踝关节周围韧带损伤内侧韧带比较少见，基本上以内侧韧带损伤为主，最常见的是距腓前韧带损伤。胫骨腓骨远端和距骨以及周围的肌肉肌腱韧带关节囊滑囊等结构共同组成了踝关节，踝关节是典型的屈戌关节，上方是前宽后窄的距骨，下方是由内外踝和胫骨后缘构成的踝穴，处在踝穴中的距骨在跖屈时较窄的面进入踝穴，因此保证了关节除了在矢状面上的屈伸外还有小范围的内外翻，因此跖屈位的损伤更为普遍；踝关节外侧腓骨较长，内侧胫骨较短，内外侧长短不一致是造成踝关节更易发生内翻扭伤的另一因素。距腓前韧带与跟腓韧带在外翻时受到撕裂的可能性最大，而说起踝关节的外翻扭伤，并不轻易发生，但是一旦发生，造成的损伤程度都会比较严重，甚至在后期还会进一步引起踝关节动静态稳定性的破坏以及增加踝关节创伤性关节炎的发生率。

当踝关节扭伤发生时，韧带收到的力首先作用在其附着的骨面上，被牵拉的骨膜像被拎起的塑料薄膜，而后下方的血管破裂出血充填在其中，与此同时发生的是渗出于局部的组织液，和血液一同增加了局部的压力，造成关节内外的力量失衡，首先这会导致踝关节的疼痛的产生，其次是踝关节的活动障碍，最后是踝

中国知网 https://www.cnki.net

关节周围在一种长期不均衡的力量作用下，关节稳定性被打破，继而关节面会产生磨损，无菌性炎症会发生，关节边缘由于骨质重建会引起骨质增生，长期的结果就是关节囊的挛缩僵硬，关节周围软组织的短缩，肌肉的张力增高，力量减弱，甚至萎缩。踝关节疼痛，关节僵硬，活动不灵活是病人在临床上最主要的主诉，并且说明这种现象的发生与天气寒冷潮湿与否有着极为密切的关联，更为严重的是影响患者的日常生活活动能力也被影响，连正常的行走，慢跑，跳跃都遭到限制。

陈旧性踝关节扭伤是由急性踝关节扭伤迁延不愈或未治疗或治疗不当等原因发展而来的一种慢性踝关节疾病。患者常主诉踝关节有弥漫性定位不清的疼痛或酸痛感，关节僵硬，自觉日常如行走、慢跑等活动受限。国外学者 Freeman 研究发现这一比率高达 40％[3]。在中医学中，踝关节扭伤被纳入筋伤疾病之中，踝关节在遇闪挫后，经络气机不畅，经络受损，气滞血瘀等，既伤于气又损于血，血溢脉外而滞于局部，渐成血瘀气滞之证[4]，随后若又治疗不当且风寒湿之邪侵袭，就会导致在相应部位出现体表可以触及的筋结、条索等。在西医学中所持的观点是急性踝关节扭伤如果没有及时治疗或治疗不当，或者未充分休息加上过早的负重活动会使损伤部位长期处于一种失于修复的状态，久而久之就会转变为陈旧性的损伤，且症状缠绵不愈，治疗难度也大大增加。

临床上治疗踝关节扭伤主要在于以下几个方面:减轻或消除踝关节疼痛症状，改善关节僵硬症状，促进关节活动度的改善，使患者的日常生活活动尽量不受影响，可以更好的进行生活工作和学习。踝关节陈旧性扭伤的治疗方法取决于患者的具体情况，需要因人而异，随着不同年龄、不同性别、病变程度等都有可能导致治疗方法或多或少的差异，总的来说，首选非手术治疗，即以非药物和药物治疗为主，严重的患者进行手术治疗。非药物治疗是药物治疗及手术治疗等的基础，对于初次就诊且症状不严重的患者非药物治疗是其第一选择，且让患者更容易在心理上接受。治疗方式如手法治疗，物理治疗，运动疗法，针灸疗法，熏蒸疗法等。手法治疗在运用时可以使用较大力度较长时间操作，目的是促使关节粘连僵硬松开；常见的理疗方式如热疗、水疗、超声波、牵引、经皮神经电刺激(TENS)等，目的是减轻疼痛，松解粘连；除此之外，可以让患者使用手杖、拐杖、助行器等辅助用具，其可以改变下肢力线，使关节负重减少；医者需告诫症状急性发

中国知网 https://www.cnki.net

作的患者注意休息，减轻或避免关节活动，少做或者不做一些促使关节疼痛的动作，如下蹲、走路、跳跃、跑步、爬楼梯。非药物治疗可以配合药物治疗同时进行，具体用药和用量参考病人的具体情况。可以采用甾体类消炎药或者非甾体类消炎药来进行局部外用，轻中度的关节疼痛即可被缓解且副作用较小，且在采用口服药前，可选择局部药物治疗，局部药物治疗可使用非甾体抗炎药的乳胶剂、膏剂、贴剂和非甾体类消炎药擦剂。对于中重度疼痛可局部药物与口服非甾体类消炎药合用，以提高疗效。当一切保守治疗的效果并不尽如人意，可以采用手术外科治疗的方法来矫正关节畸形，防止关节被进一步破坏，减轻关节疼痛，改善或提高关节功能。关节镜和关节手术是常用的方式。

本课题采用理筋正骨手法治疗陈旧性踝关节扭伤，探讨其临床治疗的有效率，进而为在临床上推广此种疗法使更多病人获益提供更有力的数据支撑。

中国知网 https://www.cnki.net

1 临床研究

1.1 临床资料

1.1.1 受试者来源

本研究对象选取病例来源于2015年11月至2016年10月马来西亚当地诊所收治陈旧性踝关节扭伤患者共80例，根据统计学中随机化原则将研究对象分为两组：即治疗组和对照组各40例。治疗组男21例，女19例，外踝扭伤33例，内踝扭伤7例；年龄22~54岁，平均(35.16±8.33)岁；病程1~9个月，平均(4.05±1.87)个月。对照组男19例，女21例，外踝扭伤32例，内踝扭伤8例；年龄24~55岁，平均（35.73±7.69）岁；病程1~10个月，平均（4.41±1.83）个月。两组患者的一般资料在统计学上具有可比性。各项资料 $P>0.05$。

1.1.2 中医诊断标准

参照国家中药管理局1994年颁布的《中医病症诊断疗效标准》[5]，①有明确的踝部扭伤史；②扭伤时间在1个月以上；③踝关节酸痛或无力，不能久行，生活、工作及运动均受影响；④内踝或外踝前下方可触及微小“筋结”，有按压痛，周围有不同程度的肿胀；⑤踝关节X线片未见骨折、脱位及其他骨病。

1.1.3 纳入标准

（1）符合中医陈旧性踝关节扭伤的诊断标准；

（2）签署知情同意书并能按要求接受治疗者；

（3）若先前接受其他治疗，于其他治疗停止后一个星期开始治疗。

1.1.4 排除标准

（1）不符合上述诊断标准和纳入标准者；

（1）曾经进行手术或关节镜治疗；

（2）合并有严重心、脑血管、肝肾疾病、传染性疾病及精神病患者；

（3）局部有皮肤破损或皮肤病者；

（4）不能坚持本实验的治疗方法者。

1.1.5 病例剔除与脱落

（1）不符合上述诊断标准和纳入标准者；

中国知网 https://www.cnki.net

(2)治疗过程中患者出现不能耐受或不良反应,根据医生判断应停止临床试验者;
(3)患者在临床试验过程中不愿继续进行临床试验，向医生提出中止临床试验要求者。

1.2 治疗方案

本研究采用随机、对照试验方法，所有入选病人签署知情同意书后，以入选顺序随机分配至理筋正骨手法配合治疗组和对照组，每组各 40 例。

1.2.1 治疗组

治疗组：理筋正骨手法：患者体位，取仰卧位，将患侧踝关节暴露并置于床边，医者先用拇指指腹按揉踝足部以放松踝关节周围软组织，在有痛性结节的位置用指端进行点揉以松筋解节；再用拿捏揉按的方式处理腓骨长短肌、胫骨前肌和小腿三头肌，放松这些肌肉，以通经活络、消肿解痛；最后医者一手握住足跟，另一手握住足背，双手拇指按在患者最为疼痛的地方，嘱助手握住患者小腿远端进行相对拔伸，环转摇晃踝部 6 ~ 7 次，在维持牵引的情况下，进行踝关节的分离对合运动，在对合的同时用拇指戳按伤处，使出槽的经筋回复其原来的解剖位置[6]。上述手法操作可重复操作数次。对于陈旧性踝关节损伤的患者，手法可以重一些，以松解粘连，恢复关节功能。以上治疗每周治疗 3 次，间隔 1~2 天治疗 1 次，每次治疗 30min，7 次为 1 个疗程，共治疗 2 个疗程[7]。

1.2.2 对照组

采用 TDP 神灯照射治疗。

器材：研究中对照组采用照射法治疗的神灯是来自重庆国人医疗器械有限公司生产的 L-I-2 型电磁波谱仪。

方法：患者坐于床上，下肢选舒适位置放置，暴露脚踝，使红外线灯垂直照射患者自觉疼痛点和筋结点，距离患者皮肤约 30~100cm，以患者感觉温热舒适为宜，以上治疗每周治疗 3 次，每次间隔 1~2 天，每次治疗 30min，7 次为 1 个疗程，共治疗 2 个疗程。

1.2.3 观察指标

中国知网 https://www.cnki.net

1.2.3.1 压痛采用美国 Wagnerinstruments 生产的 Wagner Force Ten™ - Model FDX 手控制数字力计对患者自述最痛的部位进行压痛测定。

1.2.3.2 参考 Biard-Jackson 踝关节评分标准对患者的疼痛，关节活动度，关节稳定性，行走能力，工作能力和放射学结果进行评分[8]。

1.2.4 疗效评定标准

疗效评价可以反映出患者对于治疗后状态的主观感受，有一定的参考价值，我们采用《中医病症诊断疗效标准》[5]设立了疗效判断标准。①治愈：踝关节疼痛和肿胀消失，功能恢复正常②显效：踝关节疼痛明显减轻，力量，活动度和本体感觉等基本恢复正常③有效：踝关节疼痛、僵硬症状稍微改善，但长时间或用力活动仍然感觉踝关节内有不适感④无效：踝关节症状没有改善，患者对治疗结果不满意。

1.3 统计学分析

对所有的实验数据采用 SPSS23.0 统计软件进行分析，其中计量资料采用 t 检验或方差分析，计数资料采用 x^2 检验或校正 x^2检验，均以均数 ± 标准差（$\bar{x} \pm s$）表示。条件不满足时采用秩和检验比较组间的差异是否有统计学意义；$P<0.05$ 或 $P<0.01$ 表示实验数据差异有统计学意义。

中国知网 https://www.cnki.net

2.治疗结果与分析

2.1 两组临床疗效比较 表1示，两组均于经治疗2个疗程后临床疗效比较，采用上述标准评定疗效，结果显示治愈15例，占37.5%；显效14例，占35%；有效9例，占22.5%；无效2例，占5%。总有效率95%。治疗组有效率优于对照组，差异有统计学意义（X^2=29.574，P=0.000）。

表1 两组患者疗效比较[例（%）]

组别	例数	治愈	显效	有效	无效	总有效率
治疗组	40	15（37.5）	14（35）	9（22.5）	2（5）	38（95）
对照组	40	1（2.5）	8（20）	6（15）	25（62.5）	15（37.5）

2.2 两组患者治疗前后压痛程度比较 表2示，治疗前两组疼痛值比较P>0.05，差异无统计学意义；治疗后两组均有改善（P<0.05），但组间比较差异有统计学意义（P<0.05）。

表2 两组患者治疗前后压痛程度比较(kg，$\bar{x}\pm s$)

组别	例数	治疗前	治疗后
治疗组	40	4.10±0.87	5.11±0.87*△
对照组	40	3.89±0.78	4.57±0.82*

注：与本组治疗前相比较，*P<0.05；与对照组治疗后相比，△ P<0.05

2.3 两组患者治疗前后症状评分比较 表3示，两组患者治疗前后各项症状评分均较治疗前明显改善（P<0.05）。治疗组对活动度、力量、稳定性、疼痛的改善均优于对照组（P<0.05）。

中国知网 https://www.cnki.net

表 3　两组患者治疗前后症状评分比较（分，$\bar{x}\pm s$）

组别	时间	例数	疼痛	行走能力	跑步能力	工作能力
治疗组	治疗前	40	2.41±1.19	2.20±1.86	2.40±1.58	2.67±1.52
	治疗后	40	12.23±2.45*△	11.07±2.27*△	8.80±1.20*△	7.93±2.33*△
对照组	治疗前	40	2.21±1.46	2.18±1.70	2.80±1.07	2.80±1.63
	治疗后	40	10.51±2.32*	9.60±2.49*	6.40±1.05*	5.20±2.60*

组别	时间	组数	稳定性	关节活动度	放射学结果
治疗组	治疗前	40	2.30±1.76	2.58±1.92	4.20±2.86
	治疗后	40	10.18±2.35*△	8.20±1.01*△	21.20±2.86*△
对照组	治疗前	40	2.18±1.86	2.27±2.02	2.32±1.65
	治疗后	40	9.20±1.14*	7.20±1.25*	19.20±2.16*

注：与本组治疗前相比较，*P<0.05；与对照组治疗后相比，△ P<0.05

2.4 不良反应

在治疗期间，两组治疗患者没有出现任何不良反应如皮肤损伤或皮下出血等事件发生，这表明理筋正骨手法治疗陈旧性踝关节扭伤患者是安全的。

中国知网 https://www.cnki.net

3 讨论

3.1 陈旧性踝关节扭伤的解剖探究及病因病理

由距骨近端关节面和胫骨、腓骨远端共同组成踝关节的基本解剖结构。踝关节是屈戌关节，只能在一个面内进行单方向的活动，踝关节是可在矢状面上行屈伸活动的屈戌关节。其中在体表能够触摸到的踝关节周围的体表标志即内踝、外踝和后踝，内踝是指胫骨下端向内下的骨突，后踝是后缘下方的骨突，外踝是指腓骨远端向外下的骨突，其中内踝和后踝都较外踝粗壮，内踝高于细长的外踝上0.5cm，前约1cm。距骨被这三踝构成的踝穴包含容纳其中。距骨体是呈前宽后窄的一骨块，包含距骨头、距骨体和距骨尾三段，胫骨远端内踝的内侧关节面与腓骨远端的外踝的外侧关节面卡在距骨体的两端，与之相吻合。踝关节近端即胫腓骨的下段的稳定性由周围的韧带保证，如骨间韧带，下胫腓前韧带、下胫腓后韧带及下胫腓横韧带，这些韧带坚强而有弹性，使踝关节能够自如的进行各项活动而不至于导致关节的分离。除了上述韧带外，踝关节前后方的韧带以及关节囊较两侧的韧带和关节囊都更为松弛，薄弱，这是踝关节能够完成矢状面上的屈伸活动以及冠状面上不多的内翻外翻动作的解剖基础保证。因此临床上踝关节扭伤主要发生在内翻和外翻状态下，损伤内侧和外侧副韧带，其中又因为外踝比内踝低，所以内翻导致的外侧副韧带损伤在临床更为常见。

踝关节的内侧副韧带是覆盖于近端的胫骨与远端的足楔骨包括距骨内侧、跟骨内侧和舟骨内侧的纤维结缔组织，呈三角形或呈扇形，保证踝关节外翻的稳定性和不至过度外翻拉伤。该韧带经断层解剖发现分为浅深两层，浅层包括稍短稍薄的胫舟韧带，平均长18mm，厚1.5mm以及更为丰厚的胫跟韧带，平均长30.8mm，厚2.7mm，及深层的胫距前韧带（平均长14.8mm，厚2.2mm）和胫距后韧带（平均长10.8mm，厚6.4mm）构成，十分坚韧，不易损伤，其主要生理作用是从内踝加强踝关节。当踝关节受到由内向外的暴力时，其内侧前部附着点可发生损伤，胫距后韧带与外侧的距腓后韧带相对应，其靠近踝关节的运动轴，正常运动时维持紧张状态。

中国知网 https://www.cnki.net

踝关节外侧的韧带相比较内侧更为薄弱软弱易受伤，可在一定程度上保证内翻活动时的关节稳定以及不至于过度内翻内旋时拉伤关节囊和韧带。其从前往后分别为距腓前韧带，跟腓韧带和距腓后韧带。最前方的腓距前韧带覆盖在腓骨外踝和距骨颈上，几乎水平从后方外踝向前方走行，较中间和后方的韧带也更为细长，平均长 20mm，厚 2.2mm，在踝关节跖屈内翻位时，距腓前韧带被拉得最为紧张，所以在此时可以有效的限制关节的过度跖屈内翻活动。除此之外，由于距腓前韧带附着在距骨的距骨颈，因此在踝关节处于中立位时能够防止踝关节活动而伴随的少量距骨前移活动[9][10][11]；中间的韧带为覆盖在腓骨外踝和跟骨外侧凸起的腓跟韧带，较前侧的距腓前韧带更为坚韧丰厚，在外侧副韧带当中其次坚强，其强度约为距腓前韧带的 2 倍[13]，跟腓韧带在踝关节背伸内翻位时，被拉得最为紧张，在跖屈内翻位时却保持松弛状态，所以在此时可以有效的限制关节的过度背伸内翻活动，除此之外，当踝关节在中立位时，跟腓韧带也能发挥部分防止关节过度内翻作用[14-15]，在承重中约占 30%的作用[16]，经解剖发现此韧带平均长 25mm，厚 3mm。后束是一条较为坚强坚韧的韧带，覆盖在外踝后缘至距骨后方骨突的腓距后韧带，其强度约为距腓前韧带的两倍，表面有小腿肌肉的肌腱越过，如腓骨长肌、腓骨短肌，拇长屈肌肌腱等，这些肌腱与上述韧带也有紧密的联系，这些也保证了距腓后韧带在临床上很少因踝关节的扭伤而损伤距腓后韧带保证了踝关节的背伸以及踝关节在负重时的内翻内旋的稳定性以及不至于过度活动[17-18]，在承重稳定中约占 10%的作用[19]。

踝关节是处在人体下肢远端的铰链关节，承担人体在运动或行走时与各种类型地面接触而保持平衡的功能，这离不开踝关节的屈伸活动和内外翻活动的调整适应，踝关节承受的重量来自于人体的重力，或人体在走路、跑步、跳跃过程中与地面接触而释放的运动能量。所以踝的功能包括两个方面，一是承重，二是屈伸活动。踝关节的解剖特点是表面脂肪和肌肉少，主要是一些来自小腿的肌腱通过，形成重要的内外踝管结构以及足底的足弓。小腿后方浅层是小腿三头肌，向下止于跟腱形成踝关节后方的结构；后方深层是胫骨后肌和屈踝屈趾肌，向远端从内踝后方通过，形成踝关节内侧的重要结构；小腿前方是胫骨前肌和伸踝伸趾肌群，向下在踝的前方加固关节，小腿外侧是腓骨长、短肌，在踝的外侧保护关

中国知网 https://www.cnki.net

节。在踝的生物力学研究当中，踝关节通常被放到一个足关节联合体当中看待，而使得踝关节虽然只有一个方向活动度，但是整个足关节复合体却有三个方向的活动，包括沿着横向轴进行屈伸活动，沿着纵向轴进行内收外展（此种活动只在膝关节弯曲且发生轴向旋转时才产生）和沿着一根水平方向且位于矢状面的轴进行的旋前旋后活动，因此足踝部可以变换各种各样的姿势来应对各种不平整的地面，这有些类似于上肢的腕关节可以联合手掌在空间里完成很多姿势，不同的是，足的活动空间范围远远小于腕部。

当足底垂直于纵向的长轴时，足处于标准的解剖位置，踝关节屈曲正常在20° ~30° ，平均 20° ，背伸正常在 20° ~50° ，平均为 30° ，因为距骨滑车后半部分关节面的弧长大于前半部分，因此，踝关节跖屈远大于背伸。当踝关节极度背屈时，受到以下几种因素影响，一是骨性因素，极度背屈时，距骨颈会与胫骨前缘相接触，若继续背屈，距骨颈可能面临被折断的风险，这阻碍进一步背屈的发生，且在此时，背屈踝关节的肌纤维会收缩牵拉起踝关节囊的前部分，使其不受挤压；二是极度背屈时，后部关节囊和韧带会被牵拉阻止进一步背屈的发生，三是肌肉的限制，即小腿后方三头肌的限制，这种限制早于前两者甚至可将足部固定在马蹄足的现状。同样的踝关节的跖屈也受到同样的因素影响，一是骨性因素，距骨结节与胫骨远端后方的接触限制，二是前部关节囊和韧带的牵拉限制，三是小腿前方肌肉的肌性限制，也是最早发生的限制因素。当踝关节的屈伸活动超过正常的范围时，可损伤关节的某个部分，如踝关节过度背屈可导致前脱位或者胫骨前缘骨折，踝关节过度跖屈可导致后脱位。在踝的末端极限运动时，还有跗骨间关节的少量参与，作用虽小，但是也不可忽视。当踝关节背伸时，距骨较宽的部分进入踝穴，此时踝穴必须扩大来容纳距骨，方式是外踝上升后移，但同时踝关节的稳定性也需要保证，这离不开下胫腓韧带相应变得紧张的作用，踝穴通过骨性与软组织的限制将距骨较宽的部分紧紧包裹住，这也是踝关节背屈位置如此稳定的原因。当踝关节跖屈时，相对应的外踝下降并且向前内旋转从而来适应此时距骨体较窄的部分，与背屈不同的是，此时下胫腓韧带是松弛的，这说明了踝关节此时处在相对不稳定的状态，也解释了踝关节易在跖屈位发生扭伤的原因。

中国知网 https://www.cnki.net

从上述对于踝关节的解剖特点和运动特点描述，可得知临床上最易发生的是踝关节在跖屈内翻发生的损伤。此时遭到撕裂的是腓侧副韧带，而在腓侧副韧带中又以解剖位置更为靠前，更为纤细的距腓前韧带更易受到损伤。相对应的，坚强的三角韧带很少受损，一是因为外旋外翻损伤的几率较小，二是因为其自身的坚韧性导致即使发生外翻，受损伤的也是内踝的骨质，即由于三角韧带的牵拉而产生撕脱的独立骨质。有研究表明，外翻损伤仅占踝关节扭伤的 5%，而内翻损伤伤及距腓前韧带则高达 65%[20]。在踝关节进行非正常范围内的活动时，损伤再所难免，当没有第一限制即肌肉的作用时，首先遭受损害的就是周围韧带，周围韧带发生不成程度的撕裂，或对附着点的急性牵扯而造成水肿或血肿的产生，或卡压在关节间隙中，造成关节内的压力过大和关节内疼痛的产生。如果韧带足够坚韧，关节周围骨质、软骨板或关节囊就面临着被撕裂的风险，局部充血，组织液渗出，水肿合并血肿，加剧局部的炎症进展，随着损伤的进展，这些炎症渗出物会转变为纤维结缔组织，这也是造成局部粘连的重要原因，更为严重的踝关节扭伤还面临着关节的脱位，关节内压力一直处于较高状态，关节周围力量不均衡，也会加重关节内软骨的进一步磨损破坏，软骨下骨质的代偿性增生，关节周围软组织粘连短缩，关节囊挛缩，关节周围肌肉粘连萎缩，肌力下降，关节活动也变得僵硬活动不灵活。也有研究表明炎症的迁延不愈对于韧带的修复产生明显的阻碍作用[21]。并且损伤的韧带无法确保关节的平衡也是踝关节的慢性不稳定的重要原因之一[22]。

踝关节扭伤在生活中很常见，多因在高低不平的地面上行走、跑跳或下楼梯时不慎或一些运动中导致脚踝受力不均匀或受力过大超出关节的承载能力而引发的一系列肌腱，筋膜，神经，血管等组织发生疾病[23]。临床上急性踝关节扭伤很普遍，而由急性发展而来的陈旧性也是我们在临床上十分常见却又经常被忽视的。陈旧性踝关节扭伤具体包括踝关节不稳，踝关节外侧韧带钙化，局部韧带或瘢痕组织卡顿和韧带断端挛缩引起的腱鞘炎四种[24]。

陈旧性踝关节的患者常主诉自觉关节沉重，酸胀，无力感，弥漫性的关节内定位不清的疼痛[25]，踝关节的这种无力感好发于在上下楼梯或者行走运动于不平

中国知网 https://www.cnki.net

整的道路上[26]。关节僵硬，活动度下降，并常随负重和阴雨天气变化而加重，关节可有肿胀，影响正常步行和运动。在查体中，可在外踝及内踝前下方触及肿胀、压痛或者“筋节”存在，小腿前后肌群可伴有不同程度的肌紧张和压痛[27]。

踝关节陈旧性扭伤的判断除了靠患者的自觉症状外，必要的体格检查必不可少，如用于判断是否存在距腓前韧带的损伤的前抽屉试验，在临床上最常用[28]，另外如踝内翻与外翻应力试验、后抽屉试验等也是常用来判断踝关节侧副韧带损伤程度的常用办法[29]。

3.2 西医学对陈旧性踝关节扭伤的认识

踝关节位于人体各个关节的远端，活动度并不大，但承受了自上而下较多的体重，容易在关节周围稳定结构不够坚韧或者地面不够平整时发生损伤，不分性别和年龄段都容易发生损伤。关节周围韧带是最先遭受损害的周围稳定结构。如果忽略当时的症状而置之不理就会导致关节持续不稳定的存在，损伤结构未得到充分修复，新伤又至，则踝关节的疼痛肿胀等症状持续存在，患者主观感觉肌肉力量下降，实际上踝关节真正的损伤在影像学上无法识别，更多的是患者的主观感受，患者的感觉功能障碍也会影响运动功能，神经功能障碍也会影响运动功能，这种其实是功能性的踝关节不稳定；与此相对应的是踝关节真正结构上出现的问题，如影像学表明距骨有实质性的倾斜或前移出现，或者显示踝关节周围具体的稳定结构出现损害，即踝关节机械结构性失稳定，这种长期会导致关节软骨的持续磨损，以至于踝关节创伤性关节炎的发生，这种问题统称踝关节不稳综合征，或称为慢性踝关节不稳定，临床上最容易出现的是腓侧关节副韧带的不稳定综合症。

确保良好的治疗效果在于准确的诊断疾病，第一点是病史采集，当病人前来就诊时，会主诉踝关节过往有扭伤史，且是反复扭伤，以外踝扭伤更为常见，现遗留外侧韧带处隐隐疼痛。第二点是体格检查局部有压痛点，包括韧带和相应的肌腱附着点处。前后抽屉试验可能阳性，关节活动僵硬，活动度下降。第三点是踝关节 x 线片无明显症状，但超声，磁共振等检查提示踝关节可能有器质上的不稳定存在。还有一种重要的影像学指标即应力位拍摄踝关节，可在踝关节内翻位

中国知网 https://www.cnki.net

和前抽屉位进行不同的测试。如内翻＞15° 则提示距腓前韧带损伤，＞30° 提示外侧 3 条韧带均有损伤[30]；再如当距骨位移超过 3mm 时，则判断为前抽屉试验阳性，提示距腓前韧带可能有损伤。陈兆军[31]等提出摄片应力位踝关节 X 线片对判断踝周韧带的损伤程度有重要价值，并且应以健侧作为比较，除此之外双侧对比的 CT 或 MRI 也有较高的应用价值。除了 x 线以外，CT 和 MRI 作为更先进的诊疗手段，也频繁的用于踝关节陈旧新扭伤的准确诊断中，可以确诊损伤的韧带究竟是哪一条或者是哪条韧带中的某一部分。周捷[32]等认为 MRI 是检查外侧副韧带有无断裂的好方法，并且对于职业运动员伴有内踝压疼的病例，应使用 MRI 排除关节软骨是否有损伤。但不可否认的是 MRI 的临床普及性以及必要性还是有待考察，因为许多患者在 MRI 检查结果是阴性的情况下出现了 CAI 的症状。

损伤程度各有不同，表现方式也各有差异，了解各种程度的表现方式，可以为初步判断踝关节损伤程度以及初步选择治疗方案制定依据。轻度扭伤，疼痛和肿胀都不明显，可能有或无明显压痛点，稳定结构损伤轻微；中度损伤，局部压痛明显，可触及肿胀，关节运动疼痛，僵硬，活动度下降；重度扭伤，局部损伤严重，甚则完全撕裂周围稳定的软组织，局部疼痛肿胀难忍，淤血较多，局部青紫明显，活动不能，关节松弛感明显。

Hintermann 等发现超声检查对诊断踝关节韧带损伤有较高的敏感性和特异性，是诊断韧带损伤的一种准确又简便的检查方式。超声检查的优点是可以对踝关节的韧带损伤做出较为直接的诊断，对踝关节韧带的急性损伤敏感性较高。但超声检查无法对 CAI II 度的韧带损伤情况做出量化结果。临床需要有经验的医师通过双侧踝关节超声结果进行比较得出诊断结果。Guillodo 等认为超声检查可以明确 CAI 患者中有无距腓前韧带损伤。踝关节镜检查可明确踝关节内部结构及病理改变，有助于解释 CT 及 MRI 等影像学检查结果所见。

Rubenstein 等认为，如果踝关节外侧副韧带完整，即使内侧副韧带和内踝破坏，距骨也不会发生内、外侧偏移。由此可见，外侧副韧带在维持踝关节稳定性方面起着重要的作用。外侧副韧带的损伤可按解剖学分为三级：I 级，距腓前韧带拉长。临床主要表现为外踝中度肿胀，踝关节活动不受限或轻、中度受限，无

中国知网 https://www.cnki.net

关节松弛。II级，距腓前韧带完全断裂合并跟腓韧带部分撕裂。临床表现为局部肿胀伴活动受限，可有轻、中度关节松弛。III级，距腓前韧带和跟腓韧带完全撕裂，伴有关节囊和距腓后韧带撕裂，临床表现为踝关节前外侧和足跟部弥漫性肿胀，距腓前韧带和跟腓韧带起止点或走行处有明显压痛。现代医学认为急性踝关节扭伤如果没有及时治疗或适当休息，负重活动会使损伤的韧带不能修复，踝关节周围韧带，筋膜以及关节囊等损伤导致的陈旧性的踝关节损伤持续存在，无菌性炎症反应长期存在，就会发生增生粘连，踝关节活动遗留经常疼痛，而形成陈旧性踝关节扭伤由于其常引起患者踝关节出现疼痛及功能障碍，而且症状缠绵不愈。目前临床上对于I级踝关节韧带损伤大多主张保守治疗，而对于II、III级踝关节韧带损伤或保守治疗无效的I级损伤则行手术治疗。根据踝关节韧带损伤的情况不同，治疗CAI所选择的手术方式也有所不同，总体上可大致分为解剖重建和非解剖修复两种。解剖重建是指直接对受损韧带进行修复的一种手术方式，以Brostrom术式最具代表性。手术方式为：采用外踝前方切口，切口起自外踝前方，绕过外踝尖，弧形向后，长约5CM，依次切开皮肤及皮下，注意保护足背外侧皮神经，显露出距腓前韧带切断，在维持足外翻5°～8°的情况下，将断端重叠缝合;切开腓骨长短肌腱鞘，拉开腓骨长短肌腱，切开内侧鞘壁，显露跟腓韧带，探查张力，如有松弛，亦行中间切断，断端重叠缝合;逐层关闭伤口。该手术主要适用于残留韧带有足够的长度进行叠瓦状缝合的患者。该术式的优点有：手术方法简单，操作简便，不牺牲腓骨短肌腱，可恢复正常解剖结构并保留距下关节活动度;手术切口小，术后切口感染发生率低。但是该手术也有一定局限性，主要受限于患者局部软组织的损伤情况。对于某些CAI患者，如存在外侧副韧带挛缩、缺失或瘢痕体质，或因患者过度肥胖、从事体力劳动以及对关节功能要求较高的人士如运动员等，则此手术效果欠佳。

经典术式在非解剖修复术式中Watson-Jones和Chrisman-Snook是两大经典的手术方式，也被广大临床医生所认可。Watson-Jones和Chrisman-Snook术式均可为胫距关节与距下关节提供良好的稳定性，术后获得优秀的生物力学性能，在治疗慢性踝关节不稳的过程中可取得较好的疗效。但是，由于这两种术式是非解剖修复手术，也存在很多缺点，如手术过程中移植物的等长性和踝关节的活动度均

中国知网 https://www.cnki.net

受到影响，而且术后踝关节的活动受限一直是一个比较突出的问题，主要表现在踝关节内翻受限，有文献报道与健侧相比内翻受限可达 20°，此外还有踝关节背伸受限以及距下关节活动受限等，从而出现踝关节功能受限进而导致下肢功能不良。

改良术式鉴于传统术式的缺点，所以很多学者都在以往经典术式的基础上进行了改良。Colville 等采用部分腓骨短肌腱来重建距腓前韧带和跟腓韧带，取得了很好地生物力学测试结果，但是仍无法解决手术切口过大带来的切口感染、皮缘坏死等并发症。陈前博等在踝关节外侧副韧带的修复过程中，尝试通过一种新的小切口获取部分腓骨短肌腱修复损伤韧带。该术式可以根据肌腱移植物的长短来选择移植物的固定方式，如果肌腱长度足够则可以直接将肌腱缝合，如果长度不够，则可以使用 3.5mm 锚钉将肌腱固定于距骨上。只游离部分腓骨短肌腱并且在游离的过程中保持腓骨肌支持带不切断，这就减少了对踝关节解剖结构的破坏，更多地保持了腓骨短肌腱的功能。由于传统切口变成了 3 个部位不同的小切口，极大地减少了手术切口过大所导致的并发症发生率，也减轻了术后瘢痕痛，为康复运动提供了更好的软组织条件。

近年来，原位解剖重建已被越来越多的临床医生所接受并在临床治疗中得以应用，所用的材料多为自体或异体肌腱，国内外研究也证实了异体材料重建韧带的可行性。应用不同的移植物解剖重建法相对于非解剖重建可以降低术后复发率，并且获得更好的术后疗效。自体移植物选择有半腱肌肌腱、股薄肌、髌腱。有些学者采用自体股薄肌腱解剖重建距腓前韧带及跟腓韧带取得了很好的临床疗效。该术式的优势在于手术切口小，避免了大切口带来的神经损伤、皮缘坏死等术后并发症的发生，同时该术式保留腓骨短肌腱维持了踝关节外侧的解剖结构稳定性。但股薄肌腱的取出可能会对下肢功能有一定的影响，由于未在腓骨隧道内采用内固定，术后移植物是否会发生滑动，其对手术疗效有无影响还没有明确的证据证实。有学者则采用自体半腱肌腱重建外侧副韧带，该手术优势在于自体半腱肌容易获取，并且半腱肌有足够的长度和强度来重建踝关节外侧副韧带，在提高了治疗效果的同时缩短了手术时间，并且术后发热时间短，无排斥反应。有些学者采用同种异体肌腱修复重建踝关节外侧副韧带，也取得很好的疗效。采用异体肌腱

中国知网 https://www.cnki.net

进行重建，满足了重建韧带的长度需要，避免了取腱过短导致的移植后功能无法满足。但是异体肌腱的排异反映，愈合时间较自体肌腱长，都是目前已知的一些不足。异体材料重建术后早期功能恢复理想，在一定程度上功能恢复要优于自体肌腱重建。异体肌腱重建踝关节外侧副韧带具有手术创伤小、恢复时间短、固定可靠、可早期进行功能锻炼等优点。

3.3 中医学对陈旧性踝关节扭伤的认识

在人体的所有附属运动结构之一——韧带中踝关节韧带损伤最容易遭到损伤，占据约 80％的比例[34]。踝关节扭伤无论是急性还是慢性在中医学骨伤科学分支的辨证中都归属“伤筋”。在中医骨伤科学中，筋的含义丰富多元，包罗万象，其可代指运动系统中所有的软组织，除了骨骼外。从现代解剖学角度看，肌肉、肌腱、韧带、滑囊、关节软骨，甚至神经和血管一类，都是所谓的“筋”。《素问·痿论》[35]里有相关论述，“宗筋主束骨而利机关”；清代医家高士赚曾说过“大筋连于骨内，小筋络于骨外”[36]；以及《素问·五脏生成》里记载：“诸筋者，皆属于节”，告知我们在古人眼中，筋是将骨节部分紧密联系在一起的重要组织结构，筋的作用是连属关节，络缀形体，司关节运动。综上，筋的生理功能是联络骨骼，组成关节，维持关节运动稳定，促进关节运动。中医学中的观点，外在筋骨与内在脏腑关系紧密，包括肝、肾、心、脾、肺。筋属于肝，血藏于肝，肝血濡养一身之筋，肝血盈满筋得以润养，才能充分发挥其连属关节，运动四肢的作用[37]，当肝血不足或对于经筋润养不够，则经筋失养而痿软无力，四肢痉挛，麻木，活动拘挛僵硬等症状，其生理功能也无法得到发挥。肾有主骨生髓的功效，若肾精充盈，则骨髓得以健壮生养，若肾精不足，则骨髓的生长，发育，修复都会受到极大的影响[38]。患者可能出现腰膝酸软，无力行走，活动困难等症状。除此之外，中医认为脾胃为后天之本，且肌肉属于脾脏管辖，即脾主肉，脾主统血，后天的脾胃功能强大，能从外界物质中充分吸收所需营养物质，则人体肌肉丰满，腠理致密，反之，脾胃运化功能不佳，则人体肌肉松软，腠理疏松，四肢无力，人体也会表现出虚胖，活动无力的症状。中医学中心主一身之血脉，心不收纳统摄一身之血脉，则气血无法滋养全身上下各个部位的筋骨。肺主一身呼吸之气，也对筋骨肌肉系统有着重要的作用。综上所述，对于陈旧性踝关节扭伤的患者不

中国知网 https://www.cnki.net

能只注重局部的病情，还应针对患者的五脏六腑，进行整体的调整，尤其要注重对于患者肝肾脾三脏的调整修养。《灵枢 · 本脏》[35]曰："经脉者，所以行气血而营阴阳，濡筋骨，利关节也"。《灵枢 · 本脏》亦指出："是故血和则经脉流行，营复阴阳，筋骨劲强，关节清利矣"，经脉通利，在正常道路之上循行——关节才能滑利通畅。经脉中的气与血才能顺畅，就像道路通畅，人才能从路上通过，那么路的转折处则关节通利。当遭受外伤或内因损伤，经脉破损致气血外溢或者经脉无法收摄其中气血，那么阴阳失调，筋骨得不到正常的气血供应来源，则筋骨痿软失养，关节僵硬，活动不利，《圣济总录 · 伤折恶血不散》[39]中记载："若因伤折，内动经络，血行之道不得宣通，瘀结不散，则为肿为痛"也证明了上述观点。急性踝关节扭伤的病因是外界暴力的直接打击，或者是人体无法适应外界而导致的关节不稳而扭伤，患者会表现出踝关节处的疼痛，淤血，肿胀，活动障碍等症状，等时间渐久，且未做任何处理，局部出现气血壅滞，经络阻滞，气机不畅，淤血阻络，淤血化热等症。中医看陈旧性踝关节扭伤病机在气滞血瘀，相应的在治疗上以活血化瘀为治则，同时结合中医整体观念，辩证论治观念，总结并形成一套内治脏腑，外治筋骨，综合治疗，手法为先，动静结合，辩证用药的治疗体系[40]。其中手法治疗踝关节扭伤独特的角度和疗效。在中医筋伤学中，踝关节扭伤属于"筋出槽"与"骨错缝"的范畴，这是骨伤科学体系的特有的概念，"筋出槽"是筋的解剖位置发生异常，使人体肢体发生功能障碍，如常见的筋歪，筋走，筋翻，筋卷等等，《医宗金鉴 · 正骨心法要旨》中说"筋之弛、纵、卷、挛、翻、转、离、合"其实都是"筋出槽"的不同表现，关于筋在其槽，这个槽该如何理解，正常情况下，筋在槽中，骨正筋柔，槽无法触摸，但当筋不在槽中，就可以通过触摸感知局部肌肉张力高，且与周围皮肤肌肉相比稍隆起，甚至可以触摸到较为凹陷的凹槽。置于骨错缝，比较容易理解，骨缝是指两骨之间的微小缝我们平常说的关节脱位，虽然都是由于关节的解剖位置发生变化引起肢体功能障碍，但前者比起后者，移位的程度太小，在影像学中看不到明显变化，但却能给人体带来可以轻易感知的不适。《医宗金鉴 · 正骨心法要旨》又说"或因跌扑闪失，致骨缝开错，气血郁滞，为肿为痛，宜用按摩法，按其经络，以通郁闭之气，摩其壅聚，以散瘀闭之肿，其患可愈"也指出了早在几百年前前人就

中国知网 https://www.cnki.net

以提出骨错缝的概念和病机以及相应的治则治法[41]。两者之间大部分情况是合并存在的，少量情况下单独出现。或者是筋出槽，筋不处在原来的解剖位置上，长期牵拉周围附着的骨头，则时间一长，必然出现骨错缝甚至关节脱位。或者是先由于长期姿势不正或外伤等先出现骨错缝的情况，关节无法正确对线对位，则周围附着在其上的韧带，关节囊，滑囊，关节软骨，肌肉等可能会遭受撕裂的风险，即面临着筋出槽的局面。如陈旧性踝关节扭伤就是由于关节扭伤的错缝导致的筋出槽，从局部看，踝关节骨和筋是整体，从大的看，全身的筋与骨也是一个密不可分的整体，以及外在的筋骨与内在的脏腑之间。相应的，在诊断时要注意有整体观念，把握全局，治疗时也应当筋骨同治，哪怕局部当时只表现出单独的筋或骨的症状，也要有未病先防的预防观念，这样同时也可以增加治疗效果。

本病在中医筋伤学中被称为“内外踝缝伤筋”，筋骨辩证为“筋出槽”“骨错缝”，气血辩证证属“气滞血瘀”，忽遇闪挫后，既伤于气又损于血，血溢脉外而滞于局部，渐成血瘀气滞之证。 明张介宾提出：“十二经脉之外而复有经筋者，何也？盖经筋营行表里，故出入脏腑，以次相传；经筋联缀百骸，故围络周身，各有定位……”，经筋结构受创或慢性劳损后，局部当即出现肿痛等情况，局部气滞血瘀，经络阻滞，早期未正确处理，加之受寒湿之邪侵袭，久而久之就会出现筋结、筋束等。在临床处理这一类问题时，除了要进行筋骨辩证，脏腑辨证，还要辨别损伤部位，然后根据损伤部位的病变程度，采取相应的治疗手法。中医骨伤科中处理筋伤手法包括四个手段，手法，药物，固定和功能锻炼，在这其中，手法为先且以手法为重，即所谓“七分手法，三分药物”和“手法者，诚正骨之首务哉”，使用手法的目的就在于使损伤的部位恢复到原来的状态，“使仍复于旧也”，理筋正骨手法的效果的保障在于正确诊断，这需要治疗者对于治疗部位的解剖生理病理情况都把握的很好才行，“盖正骨者，须心明手巧，既知其病情，夫善用手法，然后治之多效”，理筋正骨手法还强调手法的应用要轻巧柔和，“使患者不知其苦”，在最小痛苦的状况下，就可以消除病变，让患者容易接受，效果良好，减少后遗症。本研究可知，通过治疗后治疗组与对照组的压痛值及疼痛值明显改善，且治疗组优于对照组（$P<0.05$）。

中国知网 https://www.cnki.net

根据中医学理论，本病属于“筋出槽，骨错缝”，所以相应的治疗准则即为理筋整复，使“痛则不通”转换为“通顺则不痛”，因“筋喜柔不喜刚”，手法的力度更柔和、轻巧持久，渗透性好，才可达到舒经活络，调理筋骨，恢复活动功能，促进血液循环，恢复组织功能，减少病人痛苦，提高治愈率，降低病情复发，而这一切取决于能否正确把握清宫正骨的精髓，即将调气、守神与内力相结合。综上所述，理筋正骨手法可明显缓解患者疼痛肿胀，改善关节僵硬，提高关节活动范围，且经研究表明，患者主要症状及压痛程度均较对照组更好，差异在统计学上有意义（$P<0.05$）。

3.4 理筋正骨手法机制

中医学认为，踝关节扭伤时势必由于外力作用下导致骨错缝和筋出槽的情况出现，因此治疗的原则是“理筋正骨”，而当急性扭伤持续存在，未得到根治，炎症持续存在，损伤结构无法彻底修复，而是长期处在修复后又被损伤的状态，则又会生出新的病灶，新旧混合，使病情更为复杂，形成旧伤，即陈旧性踝关节扭伤。从本病的病机筋骨同病可推断本病治疗原则为纠正筋出槽和骨错缝,使“骨正筋顺”。为了纠正筋的解剖位置异常，松解软组织粘连，采用揉捻、指按等手法，以促进气血运行、舒通经络的目的。除此之外，对踝关节进行摇晃拔伸对合等手法虽然直接作用于关节，但对于周围软组织也有一定的牵引作用，可以改善肌肉痉挛和软组织粘连，增大关节活动度，改善关节僵硬程度。除了筋出槽，针对踝关节部位难以被影像学所检查到的骨错缝也有相应的手法促使其解剖位置恢复正常，如拔伸、摇晃、松动等，通过上述手法操作，使踝关节解剖位置对线对位，关节内外保持平衡状态，则可以很容易的治愈此类疾病，如果不纠正这种失衡状态，长期以往，则情况会愈演愈烈，至反复出现损伤，甚至关节炎症出现。因此松解踝关节软组织粘连，缓解周围肌肉痉挛和关节囊挛缩，纠正踝关节错缝即理筋正骨手法治疗此类伤科疾病的作用机制。

在治疗中，最大限度的环转摇晃踝关节是为了牵伸踝关节周围软组织，可以松解粘连，缓解小肌肉的痉挛，改善关节活动能力；拔伸法的目的是牵开挛缩的关节，使关节缝中卡顿的软组织得以恢复到正常位置，从而达到消肿止痛，改善气血运行的作用。揉捻局部筋结和经络上的腧穴可以达到舒筋通络，通利关节的

中国知网 https://www.cnki.net

作用。最后是对合戳按手法，先于受损侧反方向的被动活动再进行受损侧的被动活动，同时对受伤部位进行戳按使其复位，此手法根据中医学中“欲合先离”的理论所创，先拉开受伤侧挛缩扭转的软组织，再用戳按手法使出槽的筋归位，且戳按手法还可消除局部淤血水肿，促进局部疼痛肿胀症状的消除。手法操作时要时刻牢记“轻巧柔和，筋骨并重”。

3.5 本实验不足之处

由于本人研究水平及临床研究时间仓促、条件有限等原因，本研究还不堪完善，还存在着一些问题，如本课题主要以临床研究为主，缺乏较为客观的实验室检查数据，今后在条件允许的情况下，可加入相关实验室检查指标使之更加完善，结果更具说服力。理筋正骨手法临床疗效显著，但对其治疗机制的研究仍很缺乏，因此需要在以后工作中通过的动物实验加以验证。

4 结论

（1）理筋正骨手法治疗陈旧性踝关节扭伤患者有显著的治疗效果。

（2）理筋正骨手法陈旧性踝关节扭伤可明显减轻患者局部疼痛，改善临床疗效。而且在治疗过程中，治疗操作简单，患者痛苦少，容易被接纳，值得在临床推广。

中国知网 https://www.cnki.net

参考文献:

[1] 毛宾尧. 足外科[M]. 北京: 人民卫生出版社, 1992: 198-199.

[2] 张长杰.肌肉骨骼康复学 [M].北京:人民卫生出版社,2013:154-155.

[3] Freeman MA.Instability of the foot after injuries to the lateral ligament of the ankle.J Bone Joint Surg Br.1965 Nov;47(4):145-156.

[4] 丁洪磊. 摇拔戳手法治疗急性外踝关节扭伤的临床研究[D]. 北京:北京中医药大学, 2013.

[5] 中医病证诊断疗效标准编审委员会.中医病证诊断疗效标准[M]南京:南京大学出版社,1994:198-199.

[6] 孙树椿,孙之镐.中医筋伤学[M].北京:人民卫生出版社,2001:153.

[7] 北京中医药大学东直门医院. 刘寿山正骨经验[M]北京:人民卫生出版社,2006:361-363.

[8] BAIRD RA, JACKSONST. Fracture soft the distal part of the fibula with associated disruption of the deltoid ligament.[J]. J Bone Joint Surg Am, 1987,69(9):1346-1352.

[9] Renstrom P, Wertz M, Incavo S, et al. Strain in the lateral ligaments of the ankle[J]. Foot Ankle, 1998, 9(2):59-63.

[10] Stephens MM, Sammarco GJ. The stabilizing role of the lateral ligament complex around the ankle and subtalar joints[J].Foot Ankle.1992,13(3):130-136.

[11] Cass JR, Settles H. Ankle instability: in vitro kinematics in response to axial load[J]. Foot Ankle Int. 1994,15:134-140.

[12] Zhao J, Huangfu X. The biomechanical and clinical application of using the anterior half of the peroneus longus tendon as anautograft source. Am J Sports Med. 2012;40(3):662-671.

[13] 付文博.摇拔戳手法治疗陈旧性踝关节扭伤的病例对照研究[D].中国中医科学院,2016.

[14] 苑振峰,韩士章,杨晓飞等.腓骨骨膜瓣翻转解剖学重建踝关节外侧韧带[J].中国矫形外科杂志,2007,15(18):1421-1422.

[15] Hollis JM, Blasier RD, Flahiff CM Simulated lateral ankle ligamentous injury: change in ankle stability[J]. Am J Sports Med, 1995, 23(6):672-677.

[16] Kjaersgaard-Andersen P, Wethelund JO, Helmig P, et al. Effect of the calcaneofibular ligament on hindfoot rotation in amputetionspecimens[J].Acta Orthop Scand, 1987,58(2):135-138.

[17] 李光胜,李克舟,金利新,夏玉军. 踝关节周围韧带的解剖学研究及临床应用[J]. 齐鲁医学

中国知网 https://www.cnki.net

杂志,2011,(05):433-437.

[18] Stormont DM, Morrey BF, An KN, et al. Stability of the loaded ankle: relation between articular restraint and primary and secondary static restraints[J].Am J Sports Med,1985, 13(5):295-300.

[19] Burks RT, Morgan J. Anatomy of the lateral of the ankle ligaments[J].Am J Sports Med, 1994,22(1):72-77.

[20] Kerhoffs GM, Rowe BH, Assendelft WJ, et al. Immobilisation and functional treatment for acute lateral ankle ligament injuries in adults[DB]. Cochrane Database Syst Rev:2002, 32(1):3762-3763.

[21] Cruz-Diaz D, Hita-Contreras F, Lomas-Vega R, eral.Cross-cultural adaptation and validation of the Spanishversion of the CumberlandAnkle Instability Tool (CAIT):Aninstrument to assess unilateral chronic ankle instability[J].. ClinRheumatol, 2013,32(1):91-98.

[22] 朱渊,徐向阳,刘津浩等.踝关节本体感受器损伤与慢性踝关节不稳定关系的初步研究[J].中华创伤杂志,2011,27(5):446-450.[23] 毛宾尧. 足外科[M]. 北京: 人民卫生出版社, 1992:198-199.

[24] 陈进,覃爱军.陈旧性踝关节内侧运动性扭伤的分类及治疗[J].临床和实验医学杂志,2008,7(5):155.

[25] 阿伍提·艾克木,李俊海,林留洋等.官廷正骨手法治疗陈旧性踝关节扭伤疗效观察[J].现代中医临床,2016,(1):44-46.

[26] 高景华,高春雨,孙树椿等.摇拔戳手法治疗陈旧性踝关节扭伤 34 例[J].世界中医药,2011,06(3):214-215.

[27] 欧阳忠南,杨承荣,张覃泉,张文明. 踝关节前距腓韧带断裂的实验研究和手术验证[J]. 解放军医学杂志,1984,(04):287-290.

[28] 田伟.实用骨科学.北京:人民卫生出版社,2008:1033-1035.

[29] 王亦斑.骨关节与损伤.北京:人民卫生出版社,2007.1498-1514.

[30] 陈兆军. 孙树椿教授外踝理筋手法治疗陈旧性踝关节扭伤临床观察及机理初探[D].中国中医科学院,2016.

[31] 陈兆军,唐凡启,林顺福等. 踝关节韧带损伤的早期诊治[J]. 中国骨伤. 2007, (05):330-331.

中国知网 https://www.cnki.net

[32] 周捷,曲绵域,田得祥,等.急性外踝韧带和关节囊撕裂[J].中国运动医学杂志.1998(17): 307-308.

[33] 白万山,赵辉, 邱晓华等.磁共振成像在踝关节外侧副韧带损伤诊断中的作用J. 国外医学: 骨伤学分册, 2005, 26(2):112-115.

[34] 王正义.足踝外科学[M].北京:人民卫生出版社,2006,205-210.

[35] 程士德.黄帝内经素问[M].北京:人民卫生出版社,2006,207-208.

[36] 姜鹏飞,王培民.急性软组织损伤中医外治的研究现状[J]..中国医药导报,2009,6(1): 86-87

[37] 孙树椿,孙之镐.临床骨科学(第二版).北京:人民卫生出版社,2014,29-40.

[38] 王庆其.内经选读.北京.中国中医药出版社,2007, 76-77.

[39] 赵佶.(郑金生整理).圣济总录.北京.人民卫生出版社,2013,1627-1628.

[40] 黄桂成, 王庆甫.中医正骨学.北京:人民卫生出版社,2012,1-3.

[41] 吴谦.医宗金鉴(第六册).北京:人民卫生出版社,1957,278.

中国知网 https://www.cnki.net

附录

Biard-Jackson 踝关节功能评分

1. 疼痛:

A 无痛（15）

B 剧烈活动时轻微疼痛（12）

C 日常活动时轻微疼痛（8）

D 负重时疼痛（4）

E 静息时疼痛（0）

2. 踝关节稳定性:

A 无临床不稳定（15）

B 体育运动时不稳定（5）

C 日常活动时不稳定（0）

3. 行走能力:

A 能行走，距离不受限制，无行、无疼痛（15）

B 能行走，距离不受限制，有轻度跛行或疼痛（12）

C 行走能力中度受限（8）

D 仅能行走较短距离（4）

E 不能行走（0）

4. 跑步能力:

A 能跑步，距离不受限制，无疼痛（10）

B 能跑步，距离不受限制，有轻微疼痛（8）

C 跑步能力中度受限，有轻度疼痛（6）

D 仅能跑较短距离（3）

E 不能跑步（0）

5. 工作能力:

A 能完成一般职业工作（10）

B 能完成一般职业工作，但剧烈活动时受限（8）

C 能完成一般职业工作，但明显受限（6）

中国知网 https://www.cnki.net

D 部分残疾，仅能选择性工作（3）
E 不能工作（0）
6. 踝关节活动范围:
A 低于正常踝关节的 10 度以内（10）
B 低于正常踝关节的 15 度以内（7）
C 低于正常踝关节的 2O 度以内（4）
D 小于正常踝关节的 50%，或背屈小于 5 度（0）
7. 放射学结果
A 踝关节恢复解剖对位关系，内侧关节间隙正常，踝穴上关节间隙正常，无距骨倾斜（25）
B 基本结果与 A 一致，但关节边缘有轻度增生反应性改变（15）
C 踝穴上关节间隙轻度变窄，但踝穴上关节间隙仍大于 2mm，或距骨倾斜大于 2mm（10）
D 踝穴上关节间隙中度变窄，在 1-2mm 之间（5）
E 踝穴上关节间隙重度变窄，小于 1mm; 内侧关节间隙变宽，有重度增生反应性改变(软骨下骨质硬化、骨赘形成)（0）
8. 最高评分数值: 100 分
优: 96~100 分
良: 91~95 分
可: 81~90 分
差: 0~80 分

中国知网 https://www.cnki.net

综述
陈旧性踝关节扭伤临床治疗进展

摘要：陈旧性踝关节扭伤是临床最常见的慢性损伤疾病之一，因其病因发病机制多因患者在急性扭伤后未得到治疗和休养或因治疗不当转变而来。本文归纳了陈旧性踝关节扭伤的中西医发病机制及治疗。从中医及西医两个方面分别从发病及治疗方法进行阐述，指出了目前常见采用的中西医治疗方法，为陈旧性踝关节扭伤患者的治疗及康复提供了新思路。

关键词：陈旧性踝关节扭伤；发病机制；中西医治疗

Advances in clinical treatment of old ankle sprain

Abstract: Older ankle sprain is one of the most common chronic diseases of the disease， because the pathogenesis of the disease because of patients after acute sprain without treatment and rest or improper treatment due to change. This article summarizes the pathogenesis and treatment of humeral epicondylitis in Chinese and western medicine. From the two aspects of Chinese medicine and Western medicine from the incidence and treatment methods were described，pointed out that the current common use of Chinese and Western medicine treatment for patients with old ankle sprain treatment and rehabilitation provides a new way of thinking.

Key words: old ankle sprain; pathogenesis; Integrated Traditional Chinese and Western Medicine

踝关节扭伤是筋伤科常见的踝足部疾病，而陈旧性踝关节扭伤则是由于患者在急性扭伤后未得到治疗和休养或因治疗不当转变而来。国外学者 Freeman 研究发现这一比率高达 40%[1]。患者表现为踝足深部隐痛，肿胀，阴雨寒冷天气以及久行后加重，影响患者步行功能和日常生活[2]。陈旧性踝关节扭伤近年来逐渐被临床医务工作者和患者所重视，治疗方法也越来越多样化，且取得了较好的治疗效果。学者检索近十年来治疗陈旧性踝关节扭伤的文献，做以下综述，以期对临

中国知网 https://www.cnki.net

床上此病治疗方法的选择提供更多借鉴。

1.病因与病理分析

踝关节是由胫腓骨下端与距骨构成的以背伸跖屈为主的关节，是下肢重要的承重关节 。踝关节的解剖特点是：外踝比内踝长，内侧韧带比外侧韧带坚韧，阻止踝外翻的力量较大，而阻止踝内翻的力量较小，所以踝关节扭伤以内翻损伤最为常见。此外，距骨体前宽后窄，当踝关节背伸时，其宽部进入踝穴，同时下胫腓韧带紧张，踝关节稳定；而当踝关节跖屈时，距骨的窄部进入踝穴，下胫腓韧带松弛，踝关节不稳定，因此踝关节易在跖屈位发生扭伤。踝关节扭伤多因在高低不平的地面上行走、跑跳或下楼梯时不慎，踝关节跖屈位突然内翻或外翻而引起。根据踝部扭伤时足所处的位置不同，可分为内翻损伤和外翻损伤两类，但以内翻损伤最常见。内翻损伤者，一般损伤外侧副韧带中的距腓前韧带和跟腓韧带；外翻损伤者，则损伤内侧的三角韧带，但由于三角韧带坚韧，不易撕裂而常常发生内踝的撕脱骨折。当踝关节的翻转活动度超过了踝关节的正常活动范围和韧带的维持能力，则首先造成韧带的撕裂伤或韧带附着部的撕脱骨折以及关节囊撕裂，可使关节附近的脂肪组织及断裂的韧带嵌入关节间隙，使关节腔内的皮下发生瘀血，韧带全部撕裂时可合并踝关节的脱位。陈旧性踝关节扭伤主要是由于外伤后经久不愈或反复性损伤，或是早期较粗暴的方法整复而引起关节内外血肿机化，发生关节囊和周围组织纤维粘连，以及关节周围软组织损伤或关节错缝未得到妥善整复治疗，因而造成踝关节肿胀、疼痛难消，活动或负重时出现上述症状加重，所以说陈旧性踝关节扭伤是由于急性踝足部扭伤失养、失治、误治发展而来的慢性踝关节疾病。患者大多数是足内翻所致的外侧韧带损伤，而外侧的韧带中起稳定踝关节的韧带有前距腓韧带、腓跟韧带、后距腓韧带以及跟距骨间韧带和跟距外侧韧带。因为人在不平的路面上行走、跑步或者下楼梯的时候因为踝关节突然的跖屈内翻，导致踝关节外侧韧带发生强烈拉力，而出现挨伤或者是部分断裂，甚至是完全断裂，而在这些个损伤中尤以距腓前韧带的损伤最为多见。

2.治疗方法

2.1 手法治疗

手法治疗是陈旧性踝关节扭伤常见的一种治疗方法，现有的研究多以正骨手

中国知网 https://www.cnki.net

法和普通推拿手法为主。手法的组合应用相辅相成，可以松解粘连，改善局部血运，促进瘢痕组织吸收，同时还可拨乱反正，整复错缝，舒筋理筋，达到通则不痛、 恢复踝关节功能活动。高景华等[3]通过正骨手法中的摇拔戳法治疗 34 例陈旧性踝关节扭伤的患者，取得了较为满意的结果，治疗三次后有效率可达到 91.2％，同时研究发现正骨手法治疗疗效不受病人病程以及年龄影响，都可取得较好的疗效。阿伍提·艾克木等[4]对临床 54 例陈旧性踝关节扭伤的患者采用正骨手法治疗，优良率达 92.6％，参考 BAIRD JACKSON 踝关节评分标准，除影像学检查外的疼痛、稳定性、行走能力、跑步能力、工作能力和活动范围均较前显著提高，且差异具有统计学意义。吴山等[5]以 47 名陈旧性踝关节扭伤患者为例，评价挤压法与常规推拿治疗的疗效，研究得出手法治疗组总治愈率和有效率都高于常规推拿治疗组，且差异具有统计学意义。作者认为挤压法于常规推拿法的优势在于其可以对分离的下胫腓关节和半脱位的距骨进行整复，且操作简便，疗效更好。薛彬等[6]以“魏氏手法+特色中药外洗”比较“主动功能锻炼+扶他林软膏”，针对 78 例陈旧性踝关节扭伤的患者，对比其疗效。结果显示于疗程结束之后两组评分无差异，而在治疗半年后治疗组相较于对照组的差异具有统计学意义，说明了魏氏手法+特色中药外洗手法具有良好的远期治疗效果；除此之外，治疗组的优良率也高于对照组，且差异具有统计学意义。

2.2 针刀针灸治疗

陈旧性的踝关节扭伤，大部分是因为合并了筋脉受损，淤血内滞，导致踝关节周围筋脉失养所致，因此通过针灸或者是针刀的方法可以将踝关节周围粘连的组织剥离，降低筋膜内的压力，从而改善局部的血液循环，使得关节恢复正常的活动翻。王冠军将 132 名患者随机分为观察组和治疗组各 66 例，2 组同时给予小针刀治疗，观察组额外给予中药熏蒸治疗，经过治疗观察组总的有效率为 69. 8％，对照组有效率为 50％，且观察组 VAS 评分、Kofoed 踝关节功能评分均优于对照组。刘保新等[7]将 70 例陈旧性踝关节扭伤的患者随机平均分为两组，治疗组采用小针刀加运动理筋疗法，对照组采用小针刀加局部推拿理筋治疗，经过治疗四周后，Baird-Jackson 踝关节评分无差异，但随访 3-6 个月后的患者，治疗组复发情况及进一步的恢复情况由于对照组。因此应用小针刀配合运动理筋疗法 治疗陈旧

中国知网 https://www.cnki.net

性踝关节扭伤临床疗效满意。王宋鑫[8]将 67 例患者随机分为单纯使用针刀组 34 例和针刀配合针刺两组 33 例，结果联合治疗组治愈率明显优于单纯治疗组，但两组的有效率无明显的差异。阮炳炎[9]将 62 例患者随机分为毫火针针刺治疗组 32 例，以及采用毫针针刺对照组 30 例，经过两个疗程治疗后，根据 Ridit 统计分析结果，毫火针治疗组的 Ridit 平均值 R 治疗组>R 对照组，故治疗组疗效较好。

2.3 中药熏洗治疗

陈夏燕将 84 例患者按自愿平均分为 3 组，A 组采用电针配合中药熏洗治疗，B 组采用单纯电针治疗，c 组采用单纯中药熏洗治疗，经过两个疗程治疗后，A 组的有效率明显高于其他两组。通过电针联合中药熏洗，可以促进药物渗透，局部炎症物计临床试验，将 76 例患者分为治疗组的 39 例和对照组的 37 例，治疗组采用正骨手法治疗，每周两次，对照组采用中药熏洗治疗，每日 1 次。治疗 3 周后，治疗组的 Baird-Jackson 踝关节评分为 93. 44±4. 91 分，而对照组为 85. 81±6. 57 分。因此作者认为正骨手法和中药熏洗在治疗陈旧性踝关节扭伤方面都是有一定的疗效的，但是前者的疗效要优于后者。

2.4 西医治疗方法

西医治疗陈旧性踝关节扭伤的报道主要采用封闭、制动、锚钉及韧带重建等方法，临床可根据患者的严重程度选用适合疗法，若损伤严重且久治不愈，则应放弃保守方法而选用手术治疗。王庆来等[10]采用封闭加有限制动方法治疗陈旧性踝关节扭伤 162 例，治愈 98 例，有效 55 例，无效 9 例，总有效率达 94. 4%，治愈率为 60. 5%。王锼[11]等采用锚钉治疗踝外侧副韧带陈旧性损伤 20 例，术后 10 周复查，患者可全部完全行走，内外翻试验阴性，抽屉试验阴性；术后 12 月复查所有患者恢复正常生活，未诉踝关节不适，患者踝关节功能良好。李海东[12]等采用关节镜探查联合韧带重建治疗慢性踝关节不稳 23 例，术后随访，患者术后第 3、6、12 个月返院复查，结合患者主诉采用专科查体结合影像学检查进行评分；术后 3 个月，痊愈 14 例，有效 8 例；术后 6 个月，痊愈 19 例，有效 3 例；术后 1 年，痊愈 19 例，有效 3 例。与术前比较，关节功能评分差异均有统计学意义 (P<0.01)，术后 6 个月、1 年与术后 3 个月评分比较差异均有统计学意义(P<0.01)

中国知网 https://www.cnki.net

3.讨论

中医对急性踝关节扭伤的治疗方法丰富多样，包括：(1)中药组方外用法治疗(熏蒸、外洗、外敷等)；(2)针灸为主的治疗(毫针、火针等)；(3)推拿为主的治疗(定点按压正骨、三步推拿等)；(4)其他综合疗法。结果均能获得满意的疗效。但就目前的研究而言，尚存在一定不足，主要体现在实验样本量过小，缺乏大规模数据支撑，以致数据可信度较低；部分研究设计不够合理，缺乏随机对照，结果判定主观性强；对于采用何种治疗方法治疗何种踝关节扭伤，尚无明确定论；系统的作用机制研究相对薄弱，多为消肿止痛、行气通脉等中医理论验证。因此，为了更好地让中医外治法在踝关节扭伤治疗中服务于临床，我们有必要根据循证医学原则，科学合理地设计研究方案(如采用随机、双盲的方法)，增加实验样本收集量，使用更为客观、可量化的疗效评价指标，融合其他学科更为先进的理论和工具，开展前瞻性实验研究，阐明机理。

对于是现代医学认为陈旧性的踝关节扭伤是因为踝关节周围韧带，筋膜以及关节囊等损伤持续存在，从而演变成慢性的踝关节周围无菌性炎症刺激。在中医中，其属于“筋伤”的范畴，中医学认为急性损伤之后，出现了局部的经络损伤，从而导致气滞血瘀，气血不通，经络阻塞等，早期未正确处理，久而久之就会出现筋结、筋束等。

文献中对于此疾病的诊断也有待商榷的。一般多是认为此病是因为急性的踝关节扭伤失治、误治导致的，但很少有人关注对于陈旧性的踝关节扭伤其实需要跟很多的类似的疾病鉴别才能明确诊断，比如说跗骨窦综合征，慢性踝关节不稳，还有胫距关节前方撞击综合征等等。对于上述的疾病而言，需要特殊的x片拍摄和详细的体格检查才能确诊，比如说慢性踝关节不稳，需要距屈内翻应力下拍摄，以明确距骨上关节面是否与胫骨远端平行，跗骨窦综合征需要行踝关节的MRI检查，才能确诊是否有跗骨窦区域内有无异常的滑膜增生或者滑膜炎症刺激。因此，针对陈旧性的踝关节扭伤，单单靠病史、症状以及体征难以确诊。所以，现在的临床中，对于陈旧性踝关节扭伤是亟待明确统一的诊断标准。同时大部分文献研究并未设有对照组，治疗效果难以明确对比，且综合疗法文献报道偏多，不能明确综合治疗过程中各种疗法的具体疗效。

中国知网 https://www.cnki.net

综上所述，需更多规范的临床研究和循证医学证据完善陈旧性踝关节扭伤的治疗原则，为临床治疗提供理论依据。

参考文献:

[1] Freeman MA.Instability of the foot after injuries to the lateral ligament of the ankle.J Bone Joint Surg Br.1965;47(4):120.

[2] 朱守应.三棱针放血、按摩加针刺治疗慢性踝关节扭伤25例[J].中国 灸,2008,28(9):634-634.

[3] 高景华,高春雨,孙树椿等.摇拔戳手法治疗陈旧性踝关节扭伤 34 例[J].世界中医药,2011,06(3):214-215.

[4] 阿伍提·艾克木,李俊海,林留洋等.宫廷正骨手法治疗陈旧性踝关节扭伤疗效观察[J].现代中医临床,2016,(1):44-46.

[5] 吴山,马友盟,林应强等.挤压法治疗陈旧性踝关节扭伤 47 例[J].新中医,2000,32(7):31.

[6] 薛彬,万世元,李飞跃等.魏氏伤科法治疗陈旧性踝关节扭伤的临床分析[J].中成药,2014,36(8):1612-1615.

[7] 张进,刘保新,解娟等.小节穴针刺配合运动理筋疗法治疗Ⅰ、Ⅱ度急性踝关节扭伤的研究[J].现代中西医结合杂志,2015,(19):2074-2077.

[8] 王宋鑫,曾建文,王素华等.针刀配合针刺治疗陈旧性踝关节损伤 33 例[J].中医外治杂志,2014,23(6):42-43.

[9] 阮炳炎.毫火针治疗陈旧性踝关节扭伤 32 例[J].中国医药科学,2013,(19):107-108.

[10] 王庆来,张帆.封闭加有限制动治疗陈旧性踝关节扭伤[J].中医正骨,2003,15(1):47-48.

[11] 王弢,冯世庆.利用锚钉治疗踝外侧副韧带陈旧性损伤[J].中国伤残医学,2014,(1):34-34,35.

[12] 李海东,邬建明,陈燕才等.关节镜探查联合韧带重建治疗慢性踝关节不稳疗效观察[J].西南国防医药,2014,24(2):170-172.

中国知网 https://www.cnki.net

中国知网 https://www.cnki.net

致谢

两年研究生生涯如白驹过隙，转瞬即逝。选择继续学习深造对于自己来说是个自愿且必须的过程，世界变化太快，事物更新速度太快，唯有尽全力以奔跑的速度去学习才能不被落下，但在这过程中也需要静下心来好好去思考和沉淀。非常庆幸能有这样一个机会来到中国进行手法的学习，这对于从事这项工作数年的我来说是一次打破固有思维，融汇新知识的过程，跟随陈师进行清宫正骨手法的学习使我对于临床上手法操作有了新的感悟和体验，研究生时期的其他学习也让我初步了解了做研究的流程与步骤。

感谢这两年陈老师对我的指导与教诲，不仅在于学业的指导，更包括生活和工作上的不吝指正！

感谢同门的师兄弟姐妹对我的鼓励和帮助，让我在研究生期间能够如此顺利通过！

感谢在中国遇到过帮助我的老师和同学，以后我将以更加积极和认真的态度在工作生涯中进行探索！

中国知网 https://www.cnki.net
