# Supplementary material for: Efficacy and safety of Tuina (Chinese Therapeutic Massage) for chronic ankle instability: A systematic review and meta-analysis of randomized controlled trials
Source: PLoS One. 2025 Jun 6;20(6):e0321771. doi: 10.1371/journal.pone.0321771 (PMC12143534; doi:10.1371/journal.pone.0321771)
Supplement: S2 File — (ZIP) [file pone.0321771.s004.zip › 12.杜氏拔伸摇踝手法配合香独活...疗陈旧性踝关节扭伤疗效观察_舒文韬.pdf]

杜氏拔伸摇踝手法配合香独活血散外敷治疗陈旧性踝关节扭伤疗效观察

舒文韬，欧阳松，罗建平，刘波△，王超，石江龙，淦建帮，吴佳航
（四川省第二中医医院，四川 成都 610031）

摘要：目的：观察杜氏拔伸摇踝手法配合香独活血散外敷治疗陈旧性踝关节扭伤的临床疗效。方法：选取我院杜氏骨伤科诊断为陈旧性踝关节扭伤的患者74例，随机分为试验组和对照组各37例，试验组选用杜氏拔伸摇踝手法配合香独活血散外敷治疗，对照组给予TDP照射配合香独活血散外敷治疗，共计治疗2疗程（2周），2周后比较两组疗效及VAS评分、Biard-Jackson踝关节评分。结果：试验组总有效率为95%，对照组为78%，两组间比较差异有统计学意义（$P<0.05$）；两组患者治疗后VAS评分均低于治疗前，并且试验组评分低于对照组（$P<0.05$）；治疗后Biard-Jackson踝关节评分均高于治疗前，并且试验组评分高于对照组（$P<0.05$）。结论：杜氏拔伸摇踝手法配合香独活血散外敷治疗陈旧性踝关节扭伤疗效显著，值得临床推广。

关键词：杜氏拔伸摇踝手法；陈旧性踝关节扭伤；疗效观察
中图分类号：R 274　文献标志码：A　文章编号：1000-3649（2020）11-0158-03

陈旧性踝关节扭伤多是由于急性踝关节扭伤疾病失治、误治或积劳成疾所致[1]，主要症状为踝关节酸痛无力、不能久行、功能受限等[2]，属于骨伤科的常见病、多发病。踝关节是下肢主要负重关节之一，迁延不愈会严重影响患者的学习、工作、生活，研究表明发现多达40%的踝关节急性扭伤会遗留某些并发症[3]。我科采用杜氏拔伸摇踝手法配合香独活血散外敷治疗陈旧性踝关节扭伤，疗效满意，现报道如下。

1 资料与方法

1.1 一般资料　选择四川省第二中医医院杜氏骨伤科门诊及住院部2017年4月~2019年4月收治的陈旧性踝关节扭伤的患者74例，随机分为试验组与对照组各37例。试验组中男18例，女19例；年龄22~61岁，平均40.89±8.83岁；病程3月~7年，平均2.34±1.74年。对照组中男19例，女18例；年龄27~66岁，平均41.81±8.21岁；病程6月~6年，平均2.45±1.61年。两组性别、年龄、病程差异无统计学意义，具有可比性。

1.2 诊断标准　参照国家中医药管理局颁布的《中医病证诊断疗效标准》[4]制定：①有明确的踝部扭伤史；②扭伤时间在3周以上；③踝关节疼痛、无力，不能久行，影响生活、工作和运动；④内踝或外踝前下方处可有不同程度的肿胀和压痛；⑤X线片未见骨折和脱位。

1.3 纳入标准　①符合陈旧性踝关节扭伤诊断标准的患者；②年龄25~70岁之间，性别不限；③患者及家属均签署知情同意书，同意本研究。

1.4 排除标准　①合并心脑血管及造血系统等严重疾病者；②严重骨质疏松者、痛风性关节炎、对外用药物治疗过敏者；③精神病患者；④妇女妊娠期、月经期及哺乳期者；⑤治疗不配合、不签署知情同意书者。

1.5 治疗方法

1.5.1 试验组　采用杜氏拔伸摇踝手法配合香独活血散外敷治疗。（1）杜氏拔伸摇踝手法：杜氏拔伸摇踝手法操作要点[5]：①准备手法：患者屈膝坐于床上，术者用手掌推摩小腿前、后、内、外肌群，从腿至踝约2min；用拇指揉拨踝关节周围肌腱、韧带，重点在内外侧损伤部韧带，力度稍重，但以不加重损伤为度；用拇指在踝部顺肌腱、韧带走向捋筋，重点在筋结处。②拔伸摇踝：患者仰卧，屈膝屈髋，一助手固定股骨下端，术者一手握住跟部，拇指压在损伤韧带处，另一手握足部，相对用力牵引，持续1min后，跖屈踝关节，然后再背伸，往复5遍。最后跖屈踝关节，并内翻或外翻（翻向伤侧），旋转

△通讯作者：刘波，1572258004@qq.com。

[14] Lee A. Richter, Jim Han, Sarah Bradley, *et al*. Topical estrogen prescribing patterns for urogenital atrophy among women with breast cancer: results of a national provider survey [J]. Menopause, 2019, 26 (7): 1.
[15] 李羽禾. 乳腺癌术后内分泌药物治疗对子宫内膜影响及监测的研究进展 [J]. 肿瘤学杂志，2019，25（3）：261-263.

（收稿日期　2020-04-08）

(C)1994-2022 China Academic Journal Electronic Publishing House. All rights reserved. http://www.cnki.net

关节至正位并背伸，后期可适当加大旋转幅度，用手抵住伤侧韧带，拇指揉、捋伤侧韧带。③结束手法：用拇指（或食指或中指）深点患足之昆仑、丘墟、足临泣、太溪、照海、商丘等穴位，同时行按法或摩法，约 2min。以上治疗每天治疗 1 次，每次治疗 15 分钟，7 次为 1 个疗程，共计治疗 2 个疗程。（2）香独活血散外敷：选用我院院内制剂香独活血散（批准文号：川药制字 Z20080461）30g，用鲜开水调成糊状，外敷伤处，每日 1 次，每次 12h，7 次为 1 疗程，共计 2 个疗程。

1.5.2 对照组 对照组采用 TDP 照射配合香独活血散外敷治疗。（1）TDP 照射：患者卧于床上，下肢选舒适位置放置，暴露脚踝，使红外线灯垂直照射患者自觉疼痛点，距离患者皮肤约 30cm，以患者感觉温热舒适为宜，以上治疗每天治疗 1 次，每次治疗 30min，7 次为 1 个疗程，共计治疗 2 个疗程。（2）香独活血散外敷：方法同试验组。

1.6 观察指标 比较治疗前后试验组、对照组 VAS 评分、Biard-Jackson 踝关节评分[6]等评估患者疼痛以及功能的改善。（1）VAS 评分：取刻度为 10cm 一条直线，评分前先向患者解释此意义：直线的两端为 0 和 10，0 表示无痛，0~10 之间表示逐渐加重的疼痛程度，10 表示剧痛，由患者根据平时日常活动的情况，指出自身疼痛程度的分值。分值越大表明疼痛越重。（2）Biard-Jackson 踝关节评分：此表由 7 个条目构成，分别为疼痛 15 分、踝关节稳定性 15 分、行走能力 15 分、跑步能力 10 分、工作能力 10 分、踝关节活动范围 10 分，及影像学改变 25 分等指标对踝关节功能进行全面评价，总分为 100 分，分值越大表明功能越好。

1.7 疗效标准 参照国家中医药管理局制定的《中医病症诊断疗效标准》[7]的标准进行评定：临床治愈：患者经治疗后踝关节肿胀、疼痛及无力感消失，踝关节活动功能正常；显效：患者经治疗后踝关节肿胀消失，疼痛、无力感基本消失，踝关节活动功能基本正常；有效：患者经治疗后踝关节肿胀、疼痛及无力感有所改善，踝关节活动功能有所改善；无效：患者经治疗后踝关节肿胀、疼痛及无力感无改善，踝关节活动功能无改善。

总有效率=（临床痊愈+显效+有效例数）/总例数×100%。

1.8 统计学方法：所有试验数据均规范录入电脑，运用 SPSS 23.0 统计软件对其进行计算、分析。计量资料采用均数±标准差表示，采用 t 检验；计数资料采用卡方检验。具体方法为：性别采用卡方检验，年龄、病程采用独立样本 t 检验；组内比较采用配对样本 t 检验，组间比较采用独立样本 t 检验；疗效比较采用卡方检验。显著性检验水准 $\alpha=0.05$，P 取双侧值。

2 结 果

2.1 VAS 评分 由表 1 可知，经 t 检验，两组治疗后 VAS 评分均低于治疗前，并且试验组 VAS 评分低于对照组。其中组内比较，试验组治疗前后 $t=13.31$，$P=0.00$（$P<0.05$）；对照组治疗前后 $t=10.54$，$P=0.00$（$P<0.05$）。组间比较，2 个疗程以后，经 t 检验，$t=-3.055$，$P=0.003$（$P<0.05$）。由此表明 2 个疗程后，在改善疼痛方面，试验组疗效优于对照组。

表 1 两组治疗前后 VAS 评分比较（$\bar{x}\pm s$）

组别	治疗前	治疗后
试验组	6.51±1.35	2.65±1.40*#
对照组	6.62±1.16	3.62±1.34*

注：与治疗前相比，*$P<0.05$；与对照组相比，#$P<0.05$

2.2 Biard-Jackson 踝关节评分 由表 2 可知，经 t 检验，两组治疗后 Biard-Jackson 踝关节评分均高于治疗前，并且试验组 VAS 评分高于对照组。其中组内比较，试验组治疗前后 $t=-12.60$，$P=0.00$（$P<0.05$）；对照组治疗前后 $t=-9.94$，$P=0.00$（$P<0.05$）。组间比较，2 个疗程以后，经 t 检验，$t=3.18$，$P=0.002$（$P<0.05$）。由此表明 2 个疗程后，在改善踝关节方面，试验组疗效优于对照组。

表 2 两组治疗前后 Biard-Jackson 踝关节评分比较（$\bar{x}\pm s$）

组别	治疗前	治疗后
试验组	62.19±10.96	86.35±7.32*#
对照组	63.27±9.33	79.97±9.77*

注：与治疗前相比，*$P<0.05$；与对照组相比，#$P<0.05$

2.3 疗效比较 由表 3 可知，试验组总有效率为 95%，对照组总有效率为 78%；试验组临床痊愈率 51.4%，对照组临床痊愈率为 16.2%。试验组临床疗效明显高于对照组，经卡方检验 $\chi^2=11.927$，$P=0.008<0.05$，差异有统计学意义。

表 3 两组疗效比较（n）

组别	n	临床痊愈	显效	有效	无效	总有效(%)
试验组	37	19	7	9	2	94.59
对照组	37	6	8	15	8	78.39

3 讨 论

踝关节由距骨滑车和胫骨、腓骨的下端踝关节面组成，并由胫腓下端韧带、三角韧带（内侧副韧带）、外侧副韧带等重要的韧带连接。踝关节作为人体使用频繁、负重最大、活动范围较大的关节，其运动损伤十分常见。因踝关节解剖特点，外踝比内踝长，并且内侧副韧带较外侧副韧带坚强，临床多见伤及外侧副韧带的内翻损伤[8]。因踝部血供较差，若损伤后得不到及时、有效治疗，则容易使无菌性

(C)1994-2022 China Academic Journal Electronic Publishing House. All rights reserved. http://www.cnki.net

炎症长期存在，损伤处组织水肿吸收不畅以及血液循环障碍等，使肌肉痉挛、滑膜嵌顿以及韧带、肌腱等软组织增生粘连。中医认为本病属于“筋痹”、“骨错缝、筋出槽”范畴，踝部扭伤后，由于局部筋脉受损，血离经脉，血瘀气滞，迁延日久致络脉闭阻，局部损伤组织粘连、挛缩、瘢痕化，导致关节酸痛、功能障碍等[8]。在治疗方面，主要针对踝关节疼痛、关节僵硬、关节活动度等进行改善，以保守治疗为主，西医方面如口服止痛药物、佩戴支具、肌力训练等，必要时可行手术治疗；中医方面有针灸、推拿、熏洗等多种疗法，有操作简便，费用较低，安全性高等优点。

杜氏拔伸摇踝手法为杜氏骨伤第四代传承人李先樑主任医师在吸取杜氏手法精华后改良而成，以“筋骨并重”为治疗原则，临床上效果良好。“筋”、“骨”共同维持踝关节的力学平衡，筋柔则骨正，肌肉韧带等软组织功能正常，则有利于骨以及关节正常解剖位置的维持；反过来，骨与关节处于其正常的解剖位置，联系它们的韧带、肌肉等，才不会受到牵拉、刺激，其功能才能正常发挥，即骨正则筋柔。所以治疗不应偏废一方，‘柔筋’、‘正骨”相辅相成，缺一不可。孙氏[9]也认为，如果先以轻柔的手法缓解筋的痉挛，则骨关节的稳定与对合才能真正长治久安。足踝部因其周围肌肉、韧带比较复杂，故触诊检查十分重要，需仔细检查患者痛点，并且注重手法力度轻重适度，运摇关节手法循序渐进，避免造成二次损伤。

手法治疗上首先针对踝部及小腿部位的肌肉、韧带等软组织——即筋，进行治疗，运用推法、揉法、理筋等手法，调和气血，放松局部肌肉，改善局部血液循环，促进软组织的修复及瘀血的消散；在“筋结”部位，即痛点部位，可行稍重之手法，松解软组织粘连，可起到舒筋行气止痛的效果。然后行拔伸摇踝的手法，操作时拇指需压在损伤韧带处，便于后面的施术。首先持续牵引 1min 左右，以加大关节间隙，牵拉伤处挛缩之筋；然后在牵引下跖屈、背伸踝关节数次，以改善关节活动度，松解局部肌肉僵硬、紧张的情况；最后跖屈踝关节，并翻向伤侧，旋转关节至正位并背伸，同时拇指揉、捋伤侧韧带，目的是在逆扭伤机制下，通过拇指的按揉手法的配合，在运动中纠正踝部的骨错缝、筋出槽，使踝关节恢复正常的生理解剖位置。通过该手法的操作，可起到行气活血、滑利关节、消肿止痛之功效，并可使“骨复位、筋归槽”，使“筋骨平衡”。这也符合中医“欲合先离”的理论，与手法整复骨折、脱位异曲同工。最后行局部点穴手法配合按摩，杜自明老先生在《中医正骨经验概述》[10]中提到“久伤主用按摩”，作用为“通开关窍，以通定痛”。因踝关节疼痛必然会对其站立、行走姿势造成影响，久而久之会使下肢肌肉力学平衡遭到破坏，从而加重踝关节的疼痛，故而治疗不应当局限于踝关节部位，小腿部也应适当照顾。刘氏[11]也认为踝关节的疼痛对小腿肌肉及臀部肌肉产生渐进性的影响，从而可形成疲劳性损伤，继而加重了踝关节的病情。并且，需嘱患者尽量正常行走，并逐步进行足踝部功能锻炼，如背伸、跖屈等，但避免行与受伤姿势相同的动作，这样才能逐步改善局部肌肉的废用性萎缩，重建踝及小腿部的力学平衡，增强踝部稳定性，避免出现反复“崴脚”的情况。

香独活血散为杜氏骨伤流派特色制剂，主要成分为川木香、独活、川芎、乳香、没药等，有行气止痛、温经散寒、活血化瘀的功效，现为我院的院内制剂，广泛运用于骨伤科跌打损伤，瘀血疼痛。香独活血散外敷患处，使药物透皮吸收，与推拿手法相配合，更能起到有效改善受伤处软组织的疼痛，改善局部循环，促进瘀血以及炎性物质的吸收等作用，使临床疗效得以进一步提高。

本研究显示，杜氏拔伸摇踝手法配合香独活血散治疗陈旧性踝关节扭伤优于 TDP 照射配合香独活血散外敷治疗，能有效改善患者踝关节的疼痛及功能，并且操作简便、费用较低，具有较好的临床应用前景，值得临床推广。

参考文献

[1] 林志斌. 陈旧性踝关节扭伤临床研究进展 [J]. 亚太传统医药，2014，10 (22)：33-34.
[2] 孙树椿，孙之镐. 中医筋伤学 [M]. 北京：人民卫生出版社，1994：140-141.
[3] Freeman MA. Instability of the foot after injuries to the lateral ligament of the ankle. J Bone Joint Surg Br，1965Nov；47 (4)：669-677.
[4] 国家中医药管理局. 中医病证诊断疗效标准 [M]. 南京：南京大学出版社，1994，64-65.
[5] 李先樑，陈学忠. 实用推拿治疗 [M]. 成都：天地出版社，2000：209-211.
[6] 刘志雄. 骨科常用诊断分类方法和功能结果评定标准 [M]. 北京：科学技术出版社，2005：298-299.
[7] 国家中医药管理局. 中医病证诊断疗效标准 (2012 版) [M]. 北京：中国医药科技出版社，2012：57.
[8] 袁帅，廉杰，张向东. “筋滞骨错”手法配合温针治疗陈旧性踝关节扭伤的临床疗效分析 [J]. 世界复合医学，2016，2 (4)：10-12.
[9] 邓素玲. 孙氏手法在脊柱病治疗中的平衡观 [J]. 中国中医骨伤科杂志，2007，15 (11)：75-76.
[10] 杜自明. 中医正骨经验概述 [M]. 北京：人民卫生出版社，1960：30.
[11] 刘照富，张振南. 中药洗药治疗陈旧性踝关节扭伤的临床疗效观察 [J]. 中医临床研究，2014，6 (20)：46-47.

(收稿日期 2020-04-14)

(C)1994-2022 China Academic Journal Electronic Publishing House. All rights reserved. http://www.cnki.net
