# Supplementary material for: Efficacy and safety of Tuina (Chinese Therapeutic Massage) for chronic ankle instability: A systematic review and meta-analysis of randomized controlled trials
Source: PLoS One. 2025 Jun 6;20(6):e0321771. doi: 10.1371/journal.pone.0321771 (PMC12143534; doi:10.1371/journal.pone.0321771)
Supplement: S2 File — (ZIP) [file pone.0321771.s004.zip › 13.Manual therapy in joint and nerve structures combined with exercises in the treatment of recurrent ankle.pdf]

Manual Therapy 26 (2016) 141–149

Contents lists available at ScienceDirect

Manual Therapy

journal homepage: www.elsevier.com/math

Original article

Manual therapy in joint and nerve structures combined with exercises in the treatment of recurrent ankle sprains: A randomized, controlled trial

Gustavo Plaza-Manzano [a, 1], Marta Vergara-Vila [b, c], Sandra Val-Otero [c], Cristina Rivera-Prieto [c], Daniel Pecos-Martin [c, 2], Tomás Gallego-Izquierdo [c, 2], Alejandro Ferragut-Garcías [d, 3], Natalia Romero-Franco [d, *]

[a] Departamento de Medicina Física y Rehabilitación, Facultad de Medicina, Universidad Complutense de Madrid, Instituto de Investigación Sanitaria del Hospital Clínico San Carlos (IdISSC), Spain
[b] Community Health Centre Maria Wolff, Madrid, Spain
[c] Physiotherapy Department, University of Alcalá, E-28871, Madrid, Spain
[d] Physiotherapy and Nursery Department, University of the Balearic Islands, E-07122, Mallorca, Spain

ARTICLE INFO

Article history:
Received 29 March 2016
Received in revised form
6 July 2016
Accepted 22 August 2016

Keywords:
Manual therapy
Proprioception
Exercises
Ankle sprain

ABSTRACT

Background: Recurrent ankle sprains often involve residual symptoms for which subjects often perform proprioceptive or/and strengthening exercises. However, the effectiveness of mobilization to influence important nerve structures due to its anatomical distribution like tibial and peroneal nerves is unclear.
Objetives: To analyze the effects of proprioceptive/strengthening exercises versus the same exercises and manual therapy including mobilizations to influence joint and nerve structures in the management of recurrent ankle sprains.
Study design: A randomized single-blind controlled clinical trial.
Method: Fifty-six patients with recurrent ankle sprains and regular sports practice were randomly assigned to experimental or control group. The control group performed 4 weeks of proprioceptive/strengthening exercises; the experimental group performed 4 weeks of the same exercises combined with manual therapy (mobilizations to influence joint and nerve structures). Pain, self-reported functional ankle instability, pressure pain threshold (PPT), ankle muscle strength, and active range of motion (ROM) were evaluated in the ankle joint before, just after and one month after the interventions.
Results: The within-group differences revealed improvements in all of the variables in both groups throughout the time. Between-group differences revealed that the experimental group exhibited lower pain levels and self-reported functional ankle instability and higher PPT, ankle muscle strength and ROM values compared to the control group immediately after the interventions and one month later.
Conclusions: A protocol involving proprioceptive and strengthening exercises and manual therapy (mobilizations to influence joint and nerve structures) resulted in greater improvements in pain, self-reported functional joint stability, strength and ROM compared to exercises alone.
© 2016 Elsevier Ltd. All rights reserved.

1. Introduction

Lateral ankle sprain is the most common musculoskeletal injury among the physically active population (Gribble et al., 2016b), as well as the most prevalent ankle sprain type (85% of all ankle sprains) (Doherty et al., 2014). Although the residual pain often occurs between 5 and 25% even three years later (van Rijn et al., 2008), the development of chronic ankle instability (CAI) means a significant global healthcare burden that is associated to this injury between 20 and 41% of all lateral ankle sprains(Hubbard and Hicks-

* Corresponding author.
E-mail addresses: gustavo@fisioterapiamanual.es (G. Plaza-Manzano), ver.vergara90@gmail.com (M. Vergara-Vila), Val.sandra@hotmail.com (S. Val-Otero), cris309cr@gmail.com (C. Rivera-Prieto), daniel.pecos@uah.es (D. Pecos-Martin), tomas.gallego@uah.es (T. Gallego-Izquierdo), alejandro_sft@yahoo.es (A. Ferragut-Garcías), narf52@gmail.com (N. Romero-Franco).
[1] Fax: +34 913941518.
[2] Fax: +34 918855008.
[3] Fax: +34 971172810.

http://dx.doi.org/10.1016/j.math.2016.08.006
1356-689X/© 2016 Elsevier Ltd. All rights reserved.

Little, 2008). In this sense, the main primary manifestation is a "giving away" subjective feeling of the ankle joint that often ends up in the recurrence of the ankle sprain.

Along with the subjective instability, patients also exhibit reductions in the sense of the ankle joint position, the range of motion of the ankle, and the strength of the ankle eversion muscles that may also facilitate this recurrence and thus, the CAI (Sizer et al., 2003; Holmes and Delahunt, 2009; Munn et al., 2010; Han and Ricard, 2011). Considering this symptomatology, an exercise and manual therapy based approach is needed to manage the CAI patients.

Manual therapy approaches are often included to benefit the range of motion, the residual pain and the consequent quality of life of these patients. The joint mobilization techniques in regions such as the astragalus and talocrural joint are often included reporting increases in ankle dorsiflexion range of motion and postural control, which may help restore the functional stability (Hubbard et al., 2005; Truyols-Dominguez et al., 2013; Hoch et al., 2014; Cruz-Diaz et al., 2015).

Exercise therapy approach is also considered through proprioceptive and strengthening exercises to improve the main postural control strategies during body perturbations and decrease the recurrence of the ankle sprain. The balance-perturbation training in stable and unstable platforms, anticipatory postural adjustments and/or elastic tube exercises are often included reporting excellent results in terms of pain, range of motion, function, and postural control strategies even after an only training session (Han et al., 2009; Santos et al., 2016).

Despite the benefits from manual therapy and exercise approaches both as separated therapies and combined programs (Truyols-Dominguez et al., 2013; Lubbe et al., 2015; Cleland et al., 2013) residual symptoms and recurrence persisted after several weeks of treatment. In this regards, it is important to note that neural structures in the ankle like the tibial and peroneal nerves may play a role in the residual symptoms due to its possible affectation during the plantar flexion with inversion (PFI) ankle sprain mechanism (Hunt, 2003). In fact, Nitz et al. (1985) referred to a possible neural damage in a high percentage of patients with ankle sprain grade III that could prolong the rehabilitation process (Nitz et al., 1985). However, no studies to date have considered the treatment of these neural structures, remaining unknown its effectiveness on the CAI residual symptoms. We hypothesis that including specific manual therapy mobilizations to influence nerve structures during a program combining manual therapy and exercise approaches may results on higher benefits for CAI symptoms.

Based on the aforementioned arguments, this study aimed to analyze and compare the effects of proprioceptive and strengthening exercises versus the same protocol of exercises plus manual therapy which included mobilizations to influence joint and nerve structures, on the management of CAI symptoms.

2. Material and methods

The present research refers to a single-blinded, randomized study, with two groups. Level of pain, self-reported functional ankle instability, pressure pain threshold (PPT), muscle strength and active range of motion were considered as outcome measures. This study was guided by the CONSORT statement and included the CONSORT checklist of information/items to include when reporting a randomized trial.

2.1. Subjects

Fifty-six subjects (39 men, 17 women) aged 20–38 years (24.3 ± 2.5) with recurrent PFI ankle sprains voluntary participated in this study. Participants were randomly recruited from a sample of 70 subjects of the University Hospital of the city, referred by medical practitioners (who were Orthopaedists experts in the ankle joint) to Physiotherapy. Participants were recruited in May 2014 and the study took place from May 2014 to June 2014, in the University Hospital of the city.

The eligibility criteria were based on those endorsed by the International Ankle Consortium (Gribble et al., 2016a). Inclusion criteria: a) previous initial PFI ankle sprain graded I, II or III (I, stretching; II, partial rupture; III, complete rupture of the ligament) (Amundson, 1991) at least 12 months prior to the study beginning, diagnosed by a medical practitioner expert in the ankle joint associated with inflammatory symptoms such as swelling and pain with at least one interrupted day of desired physical activity; b) recurrence of previous PFI ankle sprains (at least one recurrence); c) had not sprained the affected ankle in the last three months; c) regular sports practice (recreational activity at least three times a week). Exclusion criteria: a) surgical treatments or b) previous fractures in either lower extremity; c) adjacent pathologies that disturbed joint integrity or function (i.e. sprains) and required ad least one interrupted day of desired physical activity.

The sample size calculation was performed considering the Visual Analogical Scale (VAS) as the primary outcome measurement. The effect size for the VAS was considered at 0.25. The correlation between repeated measures was assumed in 0.5. Considering three measures (pre, post and one month later) in two treatment groups, the sphericity correction was determined at 1. We estimated a sample size of 44 participants with a statistical power of 0.95 and level alpha of 0.05. Since we considered a 30% drop out rate, the necessary sample size was of 56 participants (28 in each intervention group). We employed the software Gpower v.3.0.18.

2.2. Ethical considerations

According to the standards of the Declaration of Helsinki, all subjects provided written informed consent before data collection. Approval was obtained from the ethical committee from the University of the city: M2013/031/20131120.

2.3. Procedures

All participants were randomly classified into the Experimental I Group (n = 28) or the Experimental II Group (n = 28). For the randomization process, an external clinical assistant randomized the intervention to each participant using computer-generated random numbers with the Epidat software v.3.1. (EPIDAT, 2014) Assessors and therapists were blind to the group assignment. Experimental I Group performed proprioceptive and strengthening exercises over 4 weeks (two times per week); Experimental II Group performed the same exercises and manual therapy over 4 weeks. Participants were not informed about the true objective of the study to avoid bias. Also, the participants did not know about the existence of another intervention group to avoid a major engagement because of the treatment. In this way, participants were blinded.

2.3.1. Proprioceptive and strengthening exercises

The protocol consisted of four sessions of six exercises that were repeated twice a week and progressed every week (Table 1). The participants were always supervised by two physiotherapists with at least six years of experience in Sports Physiotherapy and physically active populations with musculoskeletal injuries (mainly ankle joint injuries), while performing all the exercise program sessions based on previous studies, having been previously shown to be successful (Mattacola and Dwyer, 2002; McKeon and Hertel, 2008).

2.3.2. Manual therapy

The protocol consisted of talocrural joint mobilization in distraction, postero-anterior talocrural joint mobilization, antero-posterior talocrural joint mobilization, anteroposterior and posteroanterior distal tibiofibular joint mobilization, and superficial peroneal nerve neurodynamic mobilization (Fig. 1). For the neurodynamic mobilization, participants were positioned in PFI of ankle and straight leg raise, because PFI selectively stresses the peroneal division of the sciatic nerve (Sunderland, 1990). Therefore, previous researchers have used this position, combined with straight leg raise to assess neurodynamic function following ankle sprain (Pahor and Toppenberg, 1996). All these joint and nerve mobilizations were included to improve the general mobilization of these joints contributing to the neutral zone and easier movements in both directions. To this term, every mobilization was applied at grade 3 (similar to those described by Maitland) including large amplitude passive movements and respecting the participants' tolerance. With this mobilizations, we pretended to improve the mechanical sensitivity of the joint and the soft tissue adaptation to the load (Hengeveld and Banks, 2013). The duration of techniques was 20–30 s, with 2 min of resting between techniques. Each technique was repeated 10 times by two physiotherapists who are experts in manual therapy (they received a specific formation that lasted 1200 h). Similar protocol has been included for PFI ankle sprain in previous studies, where some types of joint mobilizations were similar to those included in the present study (Truyols-Dominguez et al., 2013).

2.4. Outcomes and follow-up

All the subjects reported their levels of pain, self-reported functional ankle instability, PPT, active range of motion in the ankle joint, and strength in ankle flexion and extension. Assessors measured the outcomes before and after the 4 weeks of treatment. One follow-up measurement was conducted one month later. The assessors who collected the data were external to the investigation.

2.4.1. Pain

All of the subjects rated their pain level from 0 (no pain) to 10 (maximum pain) on a visual analog scale (VAS). The VAS was reported by a provider external to the investigation. The validity of this scale has been reported in previous studies (ICC from 0.79 to 0.96) (Ferreira-Valente et al., 2011).

2.4.2. Self-reported functional ankle instability

The Cumberland Ankle Instability Tool (CAIT) was used. This scale has nine items and the maximum possible score is 30 (a score below 15 points indicates chronic instability of the ankle) (Cruz-Diaz et al., 2013). The CAIT was reported by a provider external to the investigation. As previous studies showed, this tool is valid and reliable to measure severity of self-reported functional ankle instability (ICC = 0.96) (Hiller et al., 2006).

2.4.3. Pressure pain threshold (PPT)

A digital algometer (Pain Test FPI 10-WA Algometer, Wagner Instruments; Greenwich, CT) was used to determine the PPTs in the anterior talofibular ligament, the calcaneofibular ligament, tibial malleolus and fibular malleolus. The pressure was applied perpendicularly to each structure with a stimulation of 1 cm^2 while patients were positioned lying on the unaffected side with the knee and hip in semiflexion (López-Rodríguez et al., 2012). We obtained the variable in kg/cm^2. The reliability and validity have been proved previously (ICC = 0.91) (Nussbaum and Downes, 1998; Chesterton et al., 2007).

2.4.4. Active range of motion in the ankle joint

A standard goniometer (NexGen Ergonomics; Quebec, Canada) was employed. The patients were positioned in prone position with their knees at 90° flexion and their ankle in a neutral position. The goniometer fulcrum was placed over the lateral malleolus with its proximal arm over the fibular diaphysis and its distal arm over the fifth metatarsal bone. The patients were asked to actively perform flexion and extension of the ankle. The validity and reliability of this test has been reported in previous studies (ICC from 0.62 to 0.82) (Krause et al., 2011).

Table 1
Proprioceptive and strengthening exercises protocol.

First week:
• Unipedal maintenance on stable platform (2 sets, 30 s)
• Unipedal maintenance on stable platform while drawing a five-points stars (5 times)
• Sustained single leg calf raise on stable platform (2 sets, 30 s)
• Unipedal maintenance on stable platform while catching a ball from the floor (5 times)
• Unipedal maintenance while throwing a ball (10 times)
• Eccentric contractions of eversor muscles against a light resistance (3 sets, 15 times)
Second week:
• Unipedal maintenance on stable platform with closed eyes (2 sets, 30 s)
• Unipedal maintenance on stable platform while drawing a five-points stars (5 times)
• Sustained single leg calf raise on stable platform (2 sets, 30 s)
• Unipedal maintenance on stable platform while catching a ball from the floor (5 times)
• Unipedal maintenance on stable platform while throwing a ball with different directions and increasing the velocity (10 times)
• Eccentric contractions of eversor muscles against a medium resistance (3 sets, 15 times)
Third week:
• Unipedal maintenance on unstable platform (2 sets, 30 s)
• Unipedal maintenance on unstable platform while drawing a five-points stars (5 times)
• Unipedal maintenance on stable platform while catching a ball from the floor (5 times)
• Unipedal maintenance on stable platform while throwing a ball with different directions and increasing the velocity (10 times)
• Unipedal maintenance on unstable platform with closed eyes (2 sets, 30 s)
• Eccentric contractions of eversor muscles against a medium resistance (3 sets, 15 times)
Forth week:
• Unipedal maintenance on unstable platform (2 sets, 30 s)
• Unipedal maintenance on unstable platform while drawing a five-points stars (5 times)
• Unipedal maintenance on unstable platform while catching a ball from the floor (5 times)
• Unipedal maintenance on stable platform while throwing a ball with different directions and increasing the velocity (10 times)
• Unipedal maintenance on unstable platform with closed eyes (2 sets, 30 s)
• Eccentric contractions of eversor muscles against a moderate resistance (3 sets, 15 times)

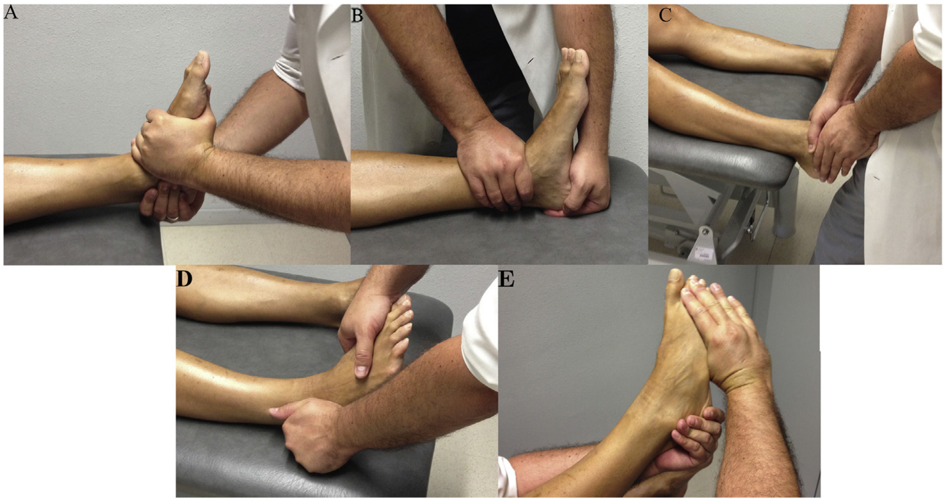

Fig. 1. Manual therapy protocol. A: Talocrural joint mobilization in distraction; B: postero-anterior talocrural joint mobilization; C: antero-posterior talocrural joint mobilization; D: antero-posterior and postero-anterior distal tibiofibular joint mobilization; E: superficial peroneal nerve mobilization.

2.4.5. *Strength in ankle flexion and extension*

Dynamic dynamometry was performed with a MicroFet-2 (Prohealthcare Products; Utah, USA) while the patients were in the supine position with their lower limbs on the therapeutic table. From this position, the participants performed ankle flexion and extension. The test-retest reliability of this tool has previously been demonstrated ($ICC = 0.97$) (Bohannon, 2006).

2.5. *Statistical analysis*

The software SPSS v.21 for Windows was used in this study. Statistical significance was set at $P < 0.05$ and the confidence intervals were considered at 95%. Structuring of statistical analysis was performed according the purpose of the study. We analyzed the effectiveness of interventions that were applied to the participants on intention to treat.

The mean and standard deviations were reported to describe all the variables. Kolmogorov–Smirnov test with the signification correction of Lillieford indicated that the quantitative values were normally distributed.

The homogeneity of the groups was evaluated with Student's t-tests for independent samples, except for the variable of gender, which was analyzed with Chi-Square of Pearson.

The between-groups and within-group differences we used a mixed model with linear procedures because we designed a repeated-measures study with unequal intervals between measurements. Analysis included within-subject differences (the time of measurement with three levels: before, immediately after the intervention period, four week after the intervention period) and between-subjects differences (the intervention with two levels: group control and group experimental).

The effect size between groups was calculated with Cohen coefficients (d) and interpreted as follows: large effect sizes, $d \geq 0.8$; moderate effect sizes, $0.8 > d > 0.2$; and small effect sizes, $d \leq 0.2$.

3. Results

Sixty-eight participants were screened in the study, but only fifty-six met the inclusion criteria and voluntary participated in the investigation (Fig. 2). No control or experimental subjects dropped out of the trial. Any participant showed harms or unintended effects during the period of study. No significant difference was found between the two groups in terms of the demographic characteristics and baseline measures (Table 2).

Table 3 shows the pain and functional instability data derived from CAIT acquired before the intervention, just after the intervention period and one month after the intervention period. The mixed model linear analysis revealed significant group-by-time interactions for VAS ($F = 18.96$ $P < 0.001$) and CAIT ($F = 24.85$, $P < 0.001$), in which the Experimental II Group exhibited significant lower levels of pain and higher scores in the CAIT immediately after the intervention and one month later compared to Experimental I Group ($P < 0.001$). The effect sizes of both VAS and CAIT variables were strong ($d = 1.23$ and $d = 1.45$, respectively). The within-group differences revealed that both groups exhibited significantly decreased VAS and increased CAIT compared to baseline ($P < 0.001$).

Table 3 also shows the PPT values for the four anatomic points (i.e., the anterior talofibular ligament (ATL), calcaneofibular ligament (CFL), fibular malleolus (FM) and tibial malleolus (TM) and strength of ankle flexion and extension. The mixed-model linear analysis revealed a significant group-by-time interactions for PPT and strength of the flexion and extension of the ankle, in which the Experimental II Group exhibited higher values of PPT for the four points (ATL: $F = 27.82$, $P < 0.001$; CFL: $F = 22.70$, $P < 0.001$; FM: $F = 27.82$, $P < 0.001$; TM: $F = 26.72$, $P < 0.001$) and higher flexion and extension strength values in the ankle joint ($F = 202.81$, $P < 0.001$ and $F = 164.64$, $P <. 001$, respectively). The effect sizes were moderate ($d = 0.65$, $d = 0.60$, $d = 0.65$, $d = 0.59$, $d = 0.90$ and $d = 0.88$, respectively). The within-group differences revealed that both groups exhibited significantly decreased PPTs at all of the pressure points and increased the ankle muscle strength compared to the baseline ($P < 0.001$).

Table 4 shows the values for the active range of motion in the ankle joint. The mixed-model linear analysis revealed a significant group-by-time interaction for the active range of motion in the ankle that indicated that the Experimental II Group exhibited higher ankle flexion ($F = 21.93$, $P < 0.001$) and ankle extension ($F = 38.79$, $P < 0.001$) values with moderate effect sizes ($d = 0.58$ and $d = 0.68$, respectively) compared to Experimental I Group. The within-group differences indicated that both groups exhibited significant increases in the active range of motion compared to baseline ($P < 0.001$).

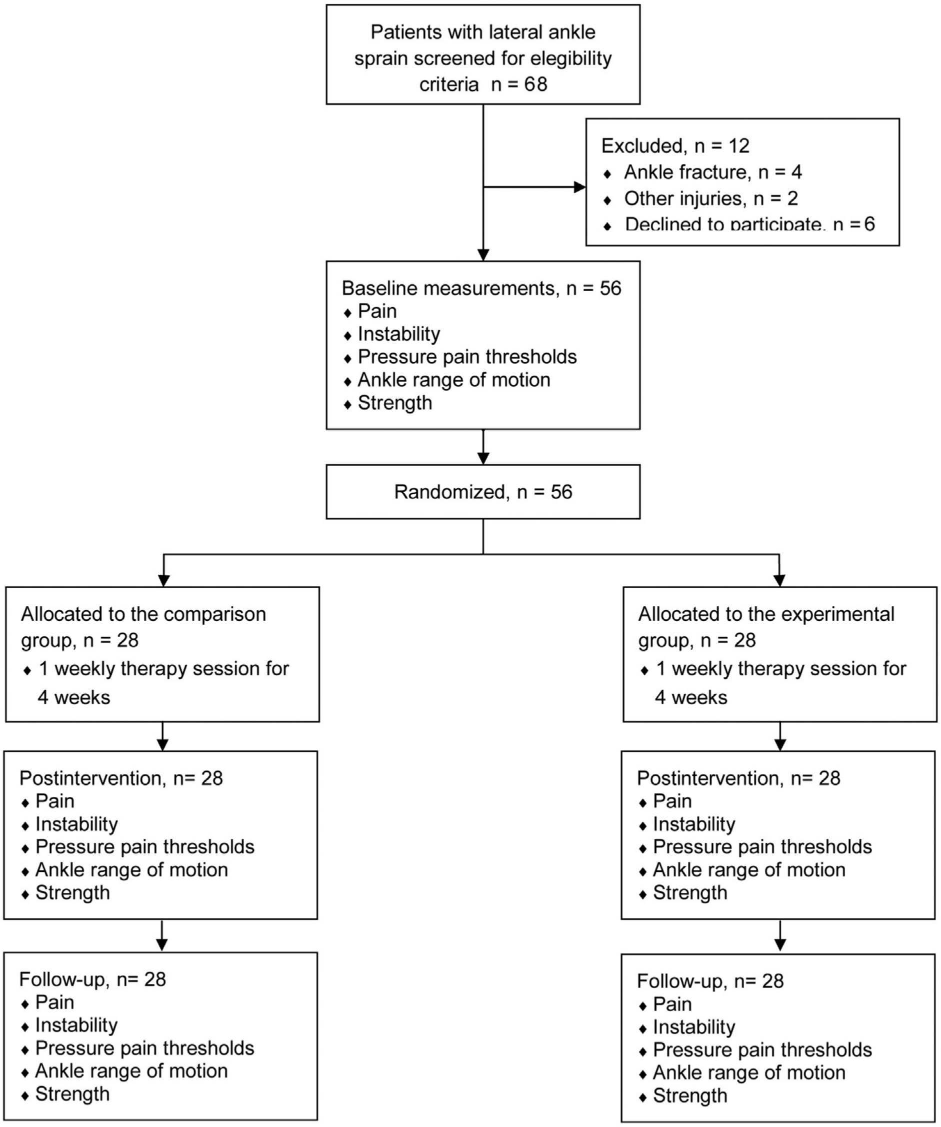

Fig. 2. Participants inclusion flow diagram.

Table 2
Baseline and demographics for both groups[a].

	Experimental I group (n = 28)	Experimental II group (n = 28)	*P* Value
Gender (male/female), n	20/8	19/9	1.00
Age, y	24.1 ± 2.4	24.4 ± 2.4	0.906
Height (m)	176.7 ± 8.9	176.5 ± 8.8	0.822
Weight (kg)	74. ± 10.8	73.7 ± 13.1	0.929
BMI	23.6 ± 1.8	23.0 ± 1.4	0.150
Pain (0–10)	5.0 ± 1.7	5.2 ± 2.0	0.728
Instability (CAIT:0–30)	15.4 ± 3.9	16.4 ± 4.9	0.435
**Presssure Pain Threshold:**			
ATL (kg/cm2)	3.6 ± 0.6	3.4 ± 0.8	0.318
CFL (kg/cm2)	5.1 ± 0.3	5.1 ± 0.4	0.973
Tibial Malleolus (kg/cm2)	6.0 ± 0.4	5.8 ± 0.1	0.446
Fibular Malleolus (kg/cm2)	5.5 ± 0.8	5.3 ± 1.3	0.875
**Muscle strength:**			
Flexion (newton)	181.8 ± 4.5	180.2 ± 4.6	0.124
Extension (newton)	133.4 ± 3.4	133.7 ± 4.1	0.559
**Active Range of Motion**			
Flexion (degrees)	27.5 ± 3.9	29.2 ± 3.8	0.103
Extension (degrees)	12.9 ± 1.8	13.7 ± 5.1	0.462

[a] *Values are mean ± SD (95% confidence interval), except for gender*; ATL = Anterior talofibular ligament; BMI = Body Mass Index; CFL = Calcaneofibular ligament.

4. Discussion

The main findings in this study indicated that a protocol involving manual techniques that influenced joint and nerve structures combined with proprioceptive and strengthening exercises induced lower levels of pain and functional ankle instability in the ankle joint and greater PPTs and strengths of the ankle muscles; however, the proprioceptive and strengthening exercises alone also induced benefits in all of the measured variables.

In functional ankle instability, we found that participants who received the combined protocol had considerably higher CAIT scores compared to those who received only the exercises. In this sense, previous studies have correlated the functional ankle instability with sensorimotor changes in hip, knee and ankle musculature, which would explain the benefits after performing proprioceptive and strengthening exercises (Kagaya et al., 2015). These authors reported high correlation between a dynamic hip mal-alignment and the heel-floor impact test. In this same line, a recent systematic literature review and meta-analysis found that the addition of proprioceptive exercises to rehabilitation programs improves self-reported functional instability and functional outcomes following ankle ligament injury (Postle et al., 2012). However, in this review, the effects on range of motion, joint position sense, postural sway, and the recurrence of the injuries remained unclear (Postle et al., 2012). On the other hand, CAIT refers to a reliable and valid tool to determine the severity of the functional ankle instability (Hiller et al., 2006). Many previous studies used CAIT score to classify "CAI participants" when CAIT is $\leq$ 25 as optimal cutoff score (Wright et al., 2014), or even discriminate participants with and without previous ankle sprain in CAIT < 27.5 (sensibility 82.9%; specificity 74.7%) (Hiller et al., 2006). However, its capacity as ankle injury predictor is unclear. While Henderson et al. determined that CAIT was not good to predict ankle re-injury in athletes (Henderson, 2015), Hiller et al., 2006 considered it a good predictor of resprain in participants with previous ankle sprain and low CAIT scores (Hiller et al., 2006). In our study, participants were not classified according CAIT score and this information was only taken into account to determine the self-reported functional ankle stability. If we considered these punctuations, participants receiving the combined protocol would have reached the CAIT score of "no CAI participants" immediately after the intervention (CAIT = 26.6) and even would have been out of "participants with previous ankle sprain" classification one month later (CAIT = 29.0). In contrast, participants receiving only

Table 3
Outcome data for pain, instability and pressure pain threshold.

	Baseline	Post-intervention	1-wk follow-up
**Pain intensity (0–10)**			
Experimental I group[a]	5.0 ± 1.7	4.0 ± 2.0	3.0 ± 1.8
Experimental II group[a]	5.2 ± 2.0	2.0 ± 0.8	0.8 ± 1.1
**Within-group change score from baseline**[b]			
Experimental I group[a]		−1.0 (−1.6,−0.8)[d]	−2.0 (−2.9,−1.7)[d]
Experimental II group[a]		−3.2 (−3.8,−2.6)[d]	−4.4 (−5.0,−3.5)[d]
**Between-group difference in change score**[c]		2.0 (1.3,2.7)[d]	2.2 (1.0,2.8)[d]
**CAITS (0–30)**			
Experimental I group[a]	15.4 ± 3.9	19.7 ± 4.4	21.3 ± 4.9
Experimental II group[a]	16.4 ± 4.9	26.6 ± 3.2	29.0 ± 0.7
**Within-group change score from baseline**[b]			
Experimental I group[a]		4.3 (2.7,5.9)[d]	5.9 (4.1,7.7)[d]
Experimental II group[a]		10.2 (8.6,11.8)[d]	12.6 (10.7,14.5)[d]
**Between-group difference in change score**[c]		6.9 (1,4,–3.3)[d]	7.6 (5.7,9.6)[d]
**Pressure Pain Threshold (kg/cm^2)**			
ATL Experimental I group[a]	3.6 ± 0.6	5.4 ± 1.1	5.9 ± 0.9
ATL Experimental II group[a]	3.4 ± 0.8	6.3 ± 0.6	7.2 ± 0.3
**Within-group change score from baseline**[b]			
ATL Experimental I group[a]		1.8 (1.4,2.2)[d]	2.3 (1.9,2.7)[d]
ATL Experimental II group[a]		2.9 (2.6,3.3)[d]	3.8 (3.5,4.2)[d]
**Between-group difference in change score**[c]		0.9 (0.4,1.4)[d]	1.3 (1.0,1.7)[d]
CFL Experimental I group[a]	5.1 ± 0.3	6.2 ± 0.8	6.4 ± 0.7
CFL Experimental II group[a]	5.1 ± 0.4	7.2 ± 0.2	7.5 ± 0.5
**Within-group change score from baseline**[b]			
CFL Experimental I group[a]	1.1 (0.8,1.5)[d]		1.3 (1.0,1.6)[d]
CFL Experimental II group[a]	2.1 (2.0, 2.3)[d]		2.5 (2.4,2.6)[d]
**Between-group difference in change score**[c]	1.0 (0.7, 1.3)[d]		1.1 (0.8, 1.4)[d]
Tibial Malleolus Experimental I group[a]	6.0 ± 0.4	7.1 ± 0.2	7.3 ± 0.4
Tibial Malleolus Experimental II group[a]	5.1 ± 0.1	7.8 ± 0.2	8.1 ± 0.1
**Within-group change score from baseline**[b]			
Tibial Malleolus Experimental I group[a]		1.1 (0.7,1.3)[d]	1.3 (0.9,1.5)[d]
Tibial Malleolus Experimental II group[a]		2.7 (2.2,3.8)[d]	2.0 (1.9,2.4)[d]
**Between-group difference in change score**[c]	0.7 (0.4,1.4)[d]		0.8 (0.5,1.1)[d]
Fibular Malleolus Experimental I group[a]	5.5 ± 0.8	6.7 ± 0.5	6.9 ± 0.6
Fibular Malleolus Experimental II group[a]	5.3 ± 1.3	7.3 ± 0.6	7.5 ± 0.3
**Within-group change score from baseline**[b]			
Fibular Malleolus Experimental I group[a]		1.2 (0.8,1.5)[d]	1.4 (0.9,1.6)[d]
Fibular Malleolus Experimental II group[a]		2.0 (1.5,2.5)[d]	2.2 (1.8,2.7)[d]
**Between-group difference in change score**[c]		0.6 (0.2,1.4)[e]	0.9 (0.2.1.5)[e]

VAS = Visual Analogical Scale, CAITS = Cumberland Ankle Instability Toll Score, ATL = Anterior Talofibular Ligament, CFL = Calcaneofibular Ligament.
[a] Values are mean ± SD.
[b] Compared to pretreatment.
[c] Values are mean (95% confidence interval).
[d] Statistically significant differences (P < 0.01).
[e] Statistically significant differences (P < 0.05).

Table 4
Outcome data for muscle strength and active range of motion.

	Baseline	Postintervention	1-wk follow-up
**Muscle strength flexion (Newton)**			
Experimental I group[a]	181.8 ± 4.5	192.3 ± 4.2	209.8 ± 3.6
Experimental II group[a]	180.2 ± 4.6	213.4 ± 5.3	228.4 ± 1.7
**Within-group change score from baseline**[b]			
Experimental I group[a]		10.5 (8.5,14.5)[d]	28.0 (25.8,30.2)[d]
Experimental II group[a]		33.2 (31.6,34.8)[d]	48.2 (46.3,49.8)
**Between-group difference in change score**[c]		21.1 (18.9,23.4)[d]	18.6 (17.0, 20.1)[d]
**Muscle strength extension (Newton)**			
Experimental I group[a]	133.4 ± 3.4	145.1 ± 4.3	149.6 ± 3.9
Experimental II group[a]	133.7 ± 4.1	159.9 ± 5.3	167.4 ± 5.0
**Within-group change score from baseline**[b]			
Experimental I group[a]		11.6 (10.0,13.2)[d]	16.2 (14.0, 18.3)[d]
Experimental II group[a]		26.2 (25.0, 27.1)[d]	33.7 (32.2, 35.1)[d]
**Between-group difference in change score**[c]		14.8 (12.3,17.5)[d]	17.8 (15.4,20.2)[d]
**Active range of motion flexion (degrees)**			
Experimental I group[a]	27.5 ± 3.9	32.2 ± 5.0	36.2 ± 5.2
Experimental II group[a]	29.2 ± 3.8	42.1 ± 5.3	46.2 ± 3.5
**Within-group change score from baseline**[b]			
Experimental I group[a]		4.7 (2.6, 6.9)[d]	8.7 (6.4, 11.1)[d]
Experimental II group[a]		12.9 (10.6, 15.2)[d]	17.0 (17.9, 19.1)[d]
**Between-group difference in change score**[c]		9.9 (7.1,12.7)[d]	10.0 (7.6,12.4)[d]
**Active range of motion extension (degrees)**			
Experimental I group[a]	12.9 ± 1.8	15.1 ± 3.3	16.9 ± 4.4
Experimental II group[a]	13.7 ± 5.1	24.6 ± 4.0	27.1 ± 3.0
**Within-group change score from baseline**[b]			
Experimental I group[a]		2.2 (0.9, 3.3)[d]	4.0 (2.3, 5.6)[d]
Experimental II group[a]		10.9 (8.5, 13.1)[d]	13.4 (11.1, 15.5)[d]
**Between-group difference in change score**[c]		9.5 (7.5,11.4)[d]	10.2 (8.1,12.2)[d]

[a] Values are mean ± SD.
[b] Compared to pretreatment.
[c] Values are mean (95% confidence interval).
[d] Statistically significant differences ($P < 0.01$).

the proprioceptive and strengthening exercises would have not reached any of two classifications.

Referring level of pain, we considered the VAS and the PPT levels in different regions, in which participants receiving the combined treatment always exhibited improved values with large effect sizes compared with participants receiving the proprioceptive and strengthening exercises as only treatment.

In the variable of ankle range of motion, despite the importance of strengthening and proprioceptive exercises in the ankle joint, our findings showed that the rehabilitation reported greater results when combined with manual techniques that influenced joint and nerve structures. In this sense, it is important to highlight the limited ankle flexion of ankle as a predictor of ankle injuries as well as functional restrictions (Tabrizi et al., 2000; Denegar et al., 2002; Terada et al., 2013). The main reason is that an ankle with limited dorsiflexion often shows external rotation of the talus near the maximum dorsiflexion, which suggests a medially limited gliding motion of the talocrural joint. This situation may decrease the bony conformity during the maximal dorsiflexion, which has been meant in a higher risk for plantar flexion with inversion ankle sprain (Kobayashi et al., 2013). Our combined rehabilitation program adding talocrural and neural mobilization could explain the greater results compared with the program involving only proprioceptive and strengthening exercises. The addition of manual techniques that also influenced nerve structures might explain the active range of motion improvements due to the superficial peroneal nerve distribution in the ankle joint and its role in the active joint mobility (Pahor and Toppenberg, 1996; Hunt, 2003). In this sense, superficial peroneal nerve passes between peroneal muscles and the lateral side of extensor digital longus, where it pierces the deep fascia and is divided in cutaneous nerves that enter the foot to innervate the dorsal surface. Therefore, the plantar flexion with inversion combined with straight leg raise would specifically stress this neural structure. Supporting our results, Pahor and Toppenberg demonstrated that patients who suffered plantar flexion with inversion ankle sprains have greater restrictions of knee extension on the injured side during the slump test. These authors suggested that the common peroneal tract play an important role due to the greater restriction in ankle extension and inversion of the foot position (Pahor and Toppenberg, 1996). Also, the frequent sequels of pain after ankle sprains seemed to be explained by an increased mechanical sensibility of peroneal nerve (Hunt, 2003) In this term, although the manual techniques that also influenced nerve structures could have help decrease this pain because these studies showed the engagement of the neural structure, more studies are needed to clear the influence of the neural mobilization. However, future researches are needed in order to analyze the importance of the manual techniques influencing nerve structures alone during the management of the residual symptoms of recurrent ankle sprain.

Linking with the ankle range of motion improvements and the influence of the nerve structures, the strength variables considered in the present study showed that participants receiving the combined treatment exhibited considerably higher values of strength in both extension and flexion movements, with large effect sizes compared with participants who only performed the exercises protocol. In this term, although all participants carried out strengthening exercises that improved the strength values, the aforementioned influence of manual techniques on ankle joint limitation consequently improved the strength restrictions more than the exercises as isolated approach.

Although the addition of manual therapy to the proprioceptive and strengthening exercises elicited better results than the isolated exercise program, both groups improved in terms of all of the variables examined in this study. This finding suggests that proprioceptive and strengthening exercises are useful in the management of the ankle sprains, but the inclusion of manual therapy might maximize treatment efficacy. This result agrees with those of Schiflan et al., who suggested that proprioception and strengthening are key parameters in the ankle sprain rehabilitation in terms of the prevention of recurrent injuries (Schiftan et al., 2015) particularly in the sporting population (Nurse, 2011; Ben Moussa Zouita et al., 2013).

4.1. Study limitations

Because the participants performed regular sports practice, they continued their normal physical activities in addition to the exercises and manual therapy program. This feature of the sample will hamper the extrapolation of the results to sedentary subjects.

Referring to the intervention, it should be taken into account that the manual therapy protocol was a set of six techniques with the same duration and standardization, not based on individual clinical reasoning approach. On the other hand, it is also possible that attention bias occurred, because the session of treatment duration was longer in those subjects receiving the combined treatment of manual therapy and exercises, which could influence the patients.

Moreover, this study included one month of follow-up, which is a short time period for examining the recurrence of the pathology and considering its chronic character.

For future studies, we recommend longer follow-ups to examine the recurrence of ankle sprain after the application of the intervention protocol and the consideration of other populations to facilitate the generalization of the results. Future studies are also needed to analyze the role of the neural restriction and the effects of neural mobilization.

5. Conclusions

The addition of manual mobilization on the ankle, which may also influence the dorsolateral peripheral nerves of the foot, to the proprioceptive and strengthening exercises elicited lower pain levels, reduced self-reported functional ankle instability, greater ankle strength, lower pressure pain thresholds and greater active ranges of motion in patients with recurrent ankle sprains compared to proprioceptive and strengthening exercises alone, although more studies with a longer follow-up are needed for the long-term results.

Regarding the practical applications of these results, health and sports professionals should consider manual therapy that may influence the joint and neural structures, as well as proprioceptive and strengthening exercises as an appropriate intervention to include in patients with recurrent inversion (plantar flexion with inversion) ankle sprain. This clinical approach may be useful to decrease the residual symptoms that often persist even months after the injury event.

Conflict of interests

None of authors have conflicts of interest (i.e., personal associations or involvement as director, officer, or expert witness) with respect to this work.

Statement of institutional review board approval of the study protocol

The study was approved by the Ethical Committee of Príncipe de Asturias Hospital in Alcalá de Henares M2013/031/20131120 (Madrid). The number registration is NCT02252276.

Funding

None declared.

References

Amundson LH. Sports medicine: functional management of ankle injuries. Kansas City. 1991.

Ben Moussa Zouita A, Majdoub O, Ferchichi H, Grandy K, Dziri C, Ben Salah FZ. The effect of 8-weeks proprioceptive exercise program in postural sway and isokinetic strength of ankle sprains of Tunisian athletes. Ann Phys Rehabil Med 2013;56:634–43.

Bohannon RW. Test-retest reliability of the MicroFET 4 hand-grip dynamometer. Physiother Theory Pract 2006;22:219–21.

Cruz-Diaz D, Hita-Contreras F, Lomas-Vega R, Osuna-Perez MC, Martinez-Amat A. Cross-cultural adaptation and validation of the Spanish version of the cumberland ankle instability tool (CAIT): an instrument to assess unilateral chronic ankle instability. Clin Rheumatol 2013;32:91–8.

Cruz-Diaz D, Lomas Vega R, Osuna-Perez MC, Hita-Contreras F, Martinez-Amat A. Effects of joint mobilization on chronic ankle instability: a randomized controlled trial. Disabil Rehabil 2015;37:601–10.

Chesterton LS, Sim J, Wright CC, Foster NE. Interrater reliability of algometry in measuring pressure pain thresholds in healthy humans, using multiple raters. Clin J Pain 2007;23:760–6.

Cleland JA, Mintken PE, McDevitt A, Bieniek ML, Carpenter KJ, Kulp K, Whitman JM. Manual physical therapy and exercise versus supervised home exercise in the management of patients with inversion ankle sprain: a multicenter randomized clinical trial. J Orthop Sports Phys Ther 2013;43:443–55.

Denegar CR, Hertel J, Fonseca J. The effect of lateral ankle sprain on dorsiflexion range of motion, posterior talar glide, and joint laxity. J Orthop Sports Phys Ther 2002;32:166–73.

Doherty C, Delahunt E, Caulfield B, Hertel J, Ryan J, Bleakley C. The incidence and prevalence of ankle sprain injury: a systematic review and meta-analysis of prospective epidemiological studies. Sports Med 2014;44:123–40.

EPIDAT: Epidemilogical Data Analysis, version 4.0. October 2014. Department of Health of the Autonomous Government of Galicia, Spain; Pan-American Health Organization (PAHO); (Center of Eu- ropean Studies) CES University, Colombia. Available at: http://dxsp.sergas.es. (Accessed 29.11.14.).

Ferreira-Valente MA, Pais-Ribeiro JL, Jensen MP. Validity of four pain intensity rating scales. Pain 2011;152:2399–404.

Gribble PA, Bleakley CM, Caulfield BM, Docherty CL, Fourchet F, Fong DT. 2016 consensus statement of the International Ankle Consortium: prevalence, impact and long-term consequences of lateral ankle sprains. Br J Sports Med 2016. http://dx.doi.org/10.1136/bjsports-2016-096188.

Gribble PA, Bleakley CM, Caulfield BM, Docherty CL, Fourchet F, Fong DT. Evidence review for the 2016 International Ankle Consortium consensus statement on the prevalence, impact and long-term consequences of lateral ankle sprains. Br J Sports Med 2016. http://dx.doi.org/10.1136/bjsports-2016-096189.

Han K, Ricard MD. Effects of 4 weeks of elastic-resistance training on ankle-evertor strength and latency. J Sport Rehabil 2011;20:157–73.

Han K, Ricard MD, Fellingham GW. Effects of a 4-week exercise program on balance using elastic tubing as a perturbation force for individuals with a history of ankle sprains. J Orthop Sports Phys Ther 2009;39:246–55.

Henderson EM. Evaluation of the Cumberland Ankle Instability Tool as a predictor of ankle re-injury in collegiate athletes. University of Delaware; 2015.

Hengeveld E, Banks K. Maitland's peripheral manipulation – elsevieron Vital Source: management of neuromusculoskeletal disorders. Elsevier Health Sciences UK; 2013.

Hiller CE, Refshauge KM, Bundy AC, Herbert RD, Kilbreath SL. The Cumberland ankle instability tool: a report of validity and reliability testing. Arch Phys Med Rehabil 2006;87:1235–41.

Hoch MC, Mullineaux DR, Andreatta RD, English RA, Medina-McKeon JM, Mattacola CG, et al. Effect of a 2-week joint mobilization intervention on single-limb balance and ankle arthrokinematics in those with chronic ankle instability. J Sport Rehabil 2014;23:18–26.

Holmes A, Delahunt E. Treatment of common deficits associated with chronic ankle instability. Sports Med 2009;39:207–24.

Hubbard TJ, Hicks-Little CA. Ankle ligament healing after an acute ankle sprain: an evidence-based approach. J Athl Train 2008;43:523–9.

Hubbard TJ, Olmsted-Kramer LC, Hertel J, Sherbondy P. Anterior–posterior mobility of the talus in subjects with chronic ankle instability. Phys Ther Sport 2005;6:146–52.

Hunt GC. Injuries of peripheral nerves of the leg, foot and ankle: an often unrecognized consequence of ankle sprains. Foot 2003;13:14–8.

Kagaya Y, Fujii Y, Nishizono H. Association between hip abductor function, rear-foot dynamic alignment, and dynamic knee valgus during single-leg squats and drop landings. J Sport Health Sci 2015;4:182–7.

Kobayashi T, Yoshida M, Yoshida M, Gamada K. Intrinsic predictive factors of noncontact lateral ankle sprain in collegiate athletes: a case-control study. Orthop J Sports Med 2013;1.

Krause DA, Cloud BA, Forster LA, Schrank JA, Hollman JH. Measurement of ankle dorsiflexion: a comparison of active and passive techniques in multiple positions. J Sport Rehabil 2011;20:333–44.

López-Rodríguez MdM, Castro-Sánchez AM, Fernández-Martínez M, Matarán-Penarrocha GA, Rodríguez-Ferrer ME. Comparación entre biodanza en medio acuático y stretching en la mejora de la calidad de vida y dolor en los pacientes con fibromialgia. Aten Primaria 2012;44:641–9.

Lubbe D, Lakhani E, Brantingham JW, Parkin-Smith GF, Cassa TK, Globe GA, Korporaal C. Manipulative therapy and rehabilitation for recurrent ankle sprain with functional instability: a short-term, assessor-blind, parallel-group randomized trial. J Manipulative Physiol Ther 2015;38:22–34.

Mattacola CG, Dwyer MK. Rehabilitation of the ankle after acute sprain or chronic instability. J Athl Train 2002;37:413–29.

McKeon PO, Hertel J. Systematic review of postural control and lateral ankle instability, part II: is balance training clinically effective? J Athl Train 2008;43:305–15.

Munn J, Sullivan SJ, Schneiders AG. Evidence of sensorimotor deficits in functional ankle instability: a systematic review with meta-analysis. J Sci Med Sport 2010;13:2–12.

Nitz AJ, Dobner JJ, Kersey D. Nerve injury and grades II and III ankle sprains. Am J Sports Med 1985;13:177–82.

Nurse M. Proprioceptive training to prevent ankle injuries in basketball. Clin J Sport Med 2011;21:277–8.

Nussbaum EL, Downes L. Reliability of clinical pressure-pain algometric measurements obtained on consecutive days. Phys Ther 1998;78:160–9.

Pahor S, Toppenberg R. An investigation of neural tissue involvement in ankle inversion sprains. Man Ther 1996;1:192–7.

Postle K, Pak D, Smith TO. Effectiveness of proprioceptive exercises for ankle ligament injury in adults: a systematic literature and meta-analysis. Man Ther 2012;17:285–91.

Santos MJ, Conceicao J, de Araujo FG, Santos GM, Keighley J. Changes in postural control after a ball-kicking balance exercise in individuals with chronic ankle instability. J Athl Train 2016;51:480–90.

Schiftan GS, Ross LA, Hahne AJ. The effectiveness of proprioceptive training in preventing ankle sprains in sporting populations: a systematic review and meta-analysis. J Sci Med Sport 2015;18:238–44.

Sizer Jr PS, Phelps V, James R, Matthijs O. Diagnosis and management of the painful ankle/foot part 1: clinical anatomy and pathomechanics. Pain Pract 2003;3:238–62.

Sunderland S. The anatomy and physiology of nerve injury. Muscle Nerve 1990;13:771–84.

Tabrizi P, McIntyre WM, Quesnel MB, Howard AW. Limited dorsiflexion predisposes to injuries of the ankle in children. J Bone Jt Surg Br 2000;82:1103–6.

Terada M, Pietrosimone BG, Gribble PA. Therapeutic interventions for increasing ankle dorsiflexion after ankle sprain: a systematic review. J Athl Train 2013;48: 696–709.

Truyols-Dominguez S, Salom-Moreno J, Abian-Vicent J, Cleland JA, Fernandez-de-Las-Penas C. Efficacy of thrust and nonthrust manipulation and exercise with or without the addition of myofascial therapy for the management of acute inversion ankle sprain: a randomized clinical trial. J Orthop Sports Phys Ther 2013;43:300–9.

van Rijn RM, van Os AG, Bernsen RM, Luijsterburg PA, Koes BW, Bierma-Zeinstra SM. What is the clinical course of acute ankle sprains? A systematic literature review. Am J Med 2008;121:324–31. e6.

Wright CJ, Arnold BL, Ross SE, Linens SW. Recalibration and validation of the Cumberland Ankle Instability Tool cutoff score for individuals with chronic ankle instability. Arch Phys Med Rehabil 2014;95:1853–9.
